# Comparative study of advanced reasoning versus baseline large-language models for histopathological diagnosis in oral and maxillofacial pathology

**DOI:** 10.1371/journal.pone.0340220

**Published:** 2025-12-31

**Authors:** Viet Anh Nguyen, Van Hung Nguyen, Thi Quynh Trang Vuong, Quoc Thanh Truong, Thi Trang Nguyen

**Affiliations:** 1 Faculty of Dentistry, Phenikaa University, Hanoi, Viet Nam; 2 Faculty of Medicine, Vinh Medical University, Nghe An, Vietnam; 3 Private Practice, Viet Anh Orthodontic Clinic, Hanoi, Vietnam; 4 Center of Pathology, Medlatec, Hanoi, Vietnam; University of the Republic Uruguay: Universidad de la Republica Uruguay, URUGUAY

## Abstract

Large language models (LLMs) are increasingly explored as diagnostic copilots in digital pathology, but whether the newest reasoning-augmented architectures provide measurable benefits over earlier versions is unknown. We compared OpenAI’s o3 model, which uses an iterative planning loop, with the baseline GPT-4o on 459 oral and maxillofacial (OMF) cases drawn from standard textbooks. Each case consisted of two to five high-resolution haematoxylin-and-eosin micrographs, and both models were queried in zero-shot mode with an identical prompt requesting a single diagnosis and supporting microscopic features. Overall, o3 correctly classified 31.6% of cases, significantly surpassing GPT-4o at 18.7% (Δ = 12.9%, P < 0.001). The largest gain was recorded for the heterogeneous “other conditions” category (37.2% versus 20.2%). For correctly diagnosed cases, o3 generated more detailed descriptions (median Likert score 9 versus 8, P = 0.003). These benefits were offset by longer mean response time (98 s versus near-instant) and lower reproducibility across repeated queries (40.2% versus 57.6%). A board-certified general pathologist achieved 28.3% accuracy on the same image set, underscoring the difficulty of the task. Ground truth was established by two board-certified OMF pathologists with high inter-rater reliability, ensuring the reliability of the reference standard. The general pathologist served only as a non-OMF difficulty benchmark. The findings indicate that advanced reasoning mechanisms materially improve diagnostic performance and explanatory depth in complex histopathology, but additional optimisation is required to meet clinical speed and consistency thresholds. Clinically, such models are adjunctive ‘copilots’ for preliminary descriptions and differential diagnoses; expert OMF pathologists retain full responsibility for sign-out.

## 1. Introduction

Artificial intelligence (AI), particularly large language models (LLMs), is playing an increasingly prominent role in pathology. Recent LLMs can synthesize medical knowledge and even integrate visual data to assist in diagnosis [1–3]. In pathology practice, deep learning image-analysis systems have already improved diagnostic accuracy for specific tasks, such as classifying whole-slide images, and helped reduce pathologist workload [4]. However, such task-specific models require large, high-quality datasets and can struggle with variability in staining or rare conditions [5]. In contrast, LLMs offer a more generalized reasoning approach. By leveraging vast training data and advanced neural architectures, they can potentially interpret diverse pathology inputs and generate diagnostic impressions across different contexts [6]. This capability is especially relevant given the global shortage of experienced pathologists and the need for tools to aid diagnostic decision-making in understaffed settings [7].

The past two years have seen the emergence of LLMs with markedly enhanced reasoning abilities [8]. Notably, OpenAI’s GPT-4 introduced in 2023 is an order of magnitude larger than its predecessors and demonstrated significantly improved contextual understanding and medical reasoning skills [2]. Unlike earlier models, these new-generation LLMs can handle complex, multi-step diagnostic problems and even accept image inputs, enabling direct interpretation of histopathological images in text form [1]. Early reports have highlighted GPT-4’s impressive performance on medical benchmarks and case challenges, approaching or exceeding human experts in some instances [9]. For example, GPT-4 matched experienced clinicians on certain pathology exam questions and even outperformed another Google’s Bard in a liver histology grading task [10]. At the same time, GPT-4’s diagnostic accuracy on image-based pathology questions remains slightly below that of pathology residents, indicating that human experts still hold an edge in nuanced image interpretation [11].

Several recent studies illustrate both the promise and the current limitations of LLMs in histopathological diagnosis. In general pathology, Ding et al. [1] found that GPT-4 could generate appropriate microscopic descriptions and diagnoses for a variety of organ tissues, performing comparably to pathology residents in many cases. In liver pathology, Zhang et al. [10] demonstrated that ChatGPT-4 achieved high accuracy (87.5%) in identifying non-alcoholic steatohepatitis and fibrosis stages from liver biopsy images, significantly outperforming Google Bard. In contrast, Li et al. [12] found that while Claude 3 Opus exhibited strong language fluency in renal pathology, its clinical accuracy and completeness were limited, highlighting ongoing challenges in producing clinically valuable pathology interpretations. Additionally, a recent study by Laohawetwanit et al. [7] assessing GPT-4’s performance in detecting and classifying colorectal adenomas revealed high sensitivity (74%) but low specificity (36%), with variable accuracy across adenoma subtypes and generally poor diagnostic consistency. Focused evaluations in the oral and maxillofacial (OMF) domain have yielded mixed results. For instance, Cuevas-Nunez et al. [13] reported that ChatGPT-4.0 correctly diagnosed roughly 60% of oral biopsy cases based on the histopathology descriptions. The accuracy varied widely by lesion type, as the model fared well on mucocele cases, likely due to their consistent and distinctive histopathological features, but struggled with inflammatory or reactive conditions.

OpenAI recently released their most advanced reasoning models, o3, in April 2025. This model is trained to think longer and reason more deeply before responding, enabling more accurate, detailed, and tool-assisted analyses, capabilities that are critical for complex histopathological diagnosis. While AI applications in pathology are expanding rapidly, there remains a critical gap in understanding how the latest reasoning-augmented LLMs compare to baseline models in real diagnostic scenarios. Most published work to date has examined either single-model performance or human-versus-AI accuracy, with relatively few studies directly contrasting an advanced LLM against a prior-generation model on the same histopathology cases, especially within the OMF pathology field. To address this gap, the present study provides a comparative analysis of an advanced reasoning LLM versus a baseline LLM in histopathological diagnosis. The study hypothesis was that whether the newest generation of LLMs can meaningfully outperform earlier models in pathology diagnosis, thereby informing how such AI tools might be best integrated into pathology practice. We focus on OMF pathology cases as a test domain, evaluating each model’s ability to generate accurate microscopic descriptions and final diagnoses. By quantifying differences in diagnostic accuracy and reasoning between the new model and its predecessor, we aim to clarify the extent of improvement offered by enhanced reasoning capabilities.

## 2. Methods

### 2.1. Study design

This cross-sectional investigation complies with the Strengthening the Reporting of Observational Studies in Epidemiology (STROBE) standards, alongside a specialized reporting checklist tailored for studies involving artificial intelligence [14,15]. Because the study relied solely on publicly available, fully anonymized textbook images and involved no human participants or animal subjects, formal ethical approval was not required. Due to the lack of prior data to estimate effect size, we assumed a medium effect size based on Cohen’s criteria (h = 0.3). With a significance level of 0.05 and a statistical power of 95%, the calculated sample size was approximately 289 images per group. The images were sourced from two well-established textbooks on OMF pathology [16,17]. These textbooks were chosen due to their comprehensive coverage of histopathological features, wide acceptance in the field, and inclusion of high-quality, well-annotated images that are essential for accurate diagnostic training and evaluation [18,19].

### 2.2. Data sources and image selection

**Fig 1:**
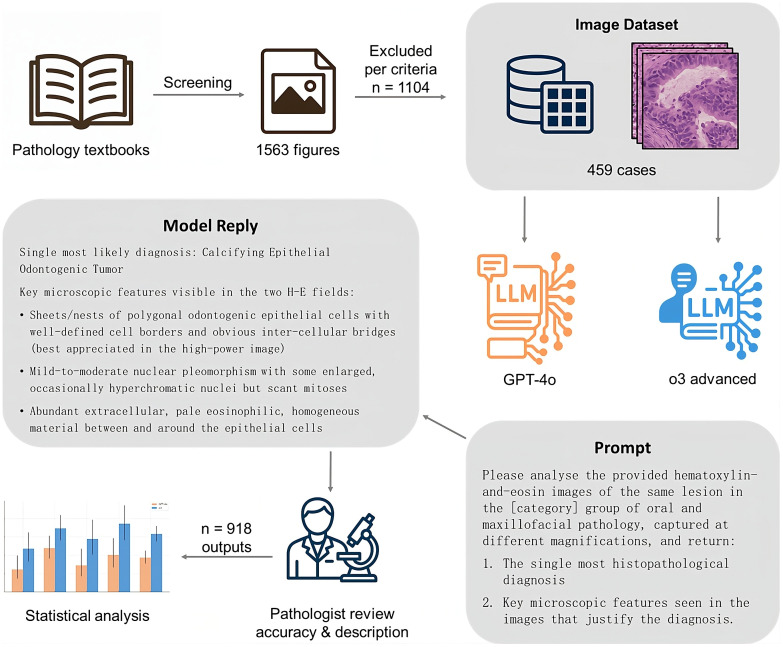
Study flowchart. LLM, large language models. All icons and schematic elements, including the stylized histology tile, were created de novo by the corresponding author in Adobe Photoshop (Adobe Inc., San Jose, CA, USA). No third-party artwork, clip art, stock icons, or adapted materials were used..

All hematoxylin-and-eosin (HE) stained figures from the reference textbooks were systematically screened for diagnostic terminology and clinical relevance based on their accompanying captions (Fig 1). Figures were included if their captions described a recognized histopathologic diagnosis or referenced a formally classified disease entity in pathology, such as “benign melanocytic nevi” and “squamous cell carcinoma subtypes”. Captions that contained sufficient terminology to imply a distinct diagnostic category, even when preceded by modifying descriptors, such as “HPV-associated oral dysplasia” and “secondary syphilis”, were also accepted. In addition, only figures that consisted of at least two images at different magnifications were included. Figures were excluded if captions were purely descriptive of microscopic features without diagnostic significance, such as “subepithelial cleft” and “perineural invasion”, or if they depicted physiologic, reactive, or artifact-related findings, such as “leukoedema”, “reactive keratosis”, and “postradiation changes”. Additional exclusions included secondary histopathologic alterations unrelated to a defined disease entity, such as “organizing thrombus” and “keratin-filled cavity”.

### 2.3. Input preparation for AI models

Each histopathological case, represented by a set of HE-stained images at different magnifications, was input into one of two AI models, either GPT-4o as the baseline model or o3 as the advanced model, using a dedicated input interface that simulated a diagnostic dialog box. The data entry into the AI models took place in May 2025. Each case was presented in a single dialog box containing the prompt and the corresponding images, without any additional diagnostic context. To ensure consistent performance across all cases and minimize limitations related to token constraints, model availability, or computational resource allocation, both models were accessed via the highest-tier subscription plan (Pro), providing full-capacity inference and unrestricted access to advanced model features. The temperature was maintained at the default value of 1.0 to reflect real‐world use by clinicians unfamiliar with API or coding; although setting the temperature to 0 may improve accuracy, it is not practical in everyday clinical practice. All images were digitally extracted directly from licensed e-book versions of the OMF pathology textbooks using the copy function of a PDF reader (Adobe Acrobat, Adobe Inc., San Jose, CA, USA). No scanning of printed pages or manual photography was involved. The images were pasted directly into the LLM chat interface without further editing or color manipulation. The input prompt for each case followed a standardized format, where [category] corresponded to the chapter in the OMF pathology textbooks where the images were sourced:

“Please analyse the provided hematoxylin-and-eosin images of the same lesion in the [category] group of oral and maxillofacial pathology, captured at different magnifications, and return:

1. The single most histopathological diagnosis
2. Key microscopic features seen in the images that justify the diagnosis.“

Once the new advanced reasoning model o3 or the older baseline model GPT-4o processed the case and generated a diagnostic output along with descriptive justification, the dialog box was cleared before inputting the next case. This approach ensured that each diagnostic session was independent, without any contextual carryover from previous cases, thereby mimicking real-life diagnostic conditions where each case is evaluated separately.

### 2.4. Outcome assessment and scoring

Two board-certified OMF pathologists independently assessed diagnostic accuracy. Since the input images do not specify the anatomical location of the specimens, all grading was based solely on histological features visible in routine HE stains; anatomical site was not taken into account. For example, diagnoses such as oral lymphoepithelial cyst and cervical (branchial-cleft-type) lymphoepithelial cyst were both considered correct. Additionally, descriptive terms that are legitimately shared by a single entity, such as “lentigo simplex” and “oral melanotic macule”, or terms applicable to two entities that are histologically indistinguishable on HE staining, such as “epidermoid cyst” and “orthokeratinized odontogenic cyst”, were also accepted as correct.

Furthermore, when the diagnosis provided by the AI model was correct, the descriptive explanations accompanying the diagnosis were evaluated and scored by the two pathologists to assess the accuracy and quality of the underlying reasoning. If the AI diagnosis was incorrect, a score of zero was assigned. For cases with correct diagnoses, microscopic descriptions were rated on a Likert scale from 1 to 10, where 10 indicated fully accurate histological descriptions, scores between 5 and 9 represented partially accurate descriptions, and scores from 1 to 4 reflected mostly inaccurate descriptions. The 1–10 Likert scale was adopted to capture finer gradations of partially correct microscopic descriptions and to reduce ceiling and floor effects that can occur with 1–5 scales.

The two board-certified OMF pathologists served as the gold standard for both the binary diagnostic endpoint (correct/incorrect) and the 1–10 descriptive scoring. Disagreements, if any, were resolved by consensus before locking the reference labels. The OMF pathologists did not participate in any accuracy comparisons, their role was solely to establish the reference standard.

To assess intra-model reproducibility, 20% of all histopathological cases were randomly re-entered into each model twice using identical input formats and images, allowing evaluation of the consistency of diagnostic outputs generated by the same model under repeated conditions. For each duplicated case, the two corresponding diagnoses generated by the model were rated for agreement by an OMF pathologist.

Additionally, a board-certified general pathologist (non-OMF) independently evaluated 20% of the cases and provided diagnoses to determine the overall difficulty level of the question set. The reason for selecting a general pathologist as a comparator to provide a difficulty benchmark was that (i) it avoided incorporation bias, because OMF pathologists had already defined the gold standard; (ii) it mirrored real-world deployment, where LLMs may assist non-specialists; and (iii) it provided a non-specialist baseline rather than a circular comparison to the reference raters.

### 2.5. Statistical analysis

All analyses were performed using SPSS Statistics version 26.0 (IBM Corp., Armonk, NY, USA). Inter-rater agreement between the two certified OMF pathologists was assessed in two domains. For binary judgments of diagnostic accuracy (correct vs. incorrect), inter-rater reliability was assessed using Cohen’s kappa coefficient, which quantifies agreement beyond chance for categorical outcomes. For ordinal ratings of the descriptive explanations (scored on a 1–10 Likert scale), inter-rater agreement was evaluated using the intraclass correlation coefficient (ICC), specifically the ICC(3,1) model, which is based on a two-way mixed-effects approach with single measures and absolute agreement, to assess the level of concordance between raters. For intra-model reproducibility, consistency in repeated diagnostic outputs was compared using the χ^2^ test. For subgroup analysis, cases were retrospectively categorized into four diagnostic groups based on their underlying pathology: (1) odontogenic tumors and cysts; (2) non-odontogenic tumors, cysts, and neoplasms (non-salivary); (3) salivary gland disorders; and (4) other conditions, including developmental-congenital conditions, infectious conditions, inflammatory and immune-mediated conditions, physical injuries, pigmentations, or systemic manifestations. Diagnostic accuracy between the two AI models was compared using the χ^2^ test on the proportions of correct diagnoses. Microscopic description scores were compared between models using the Mann-Whitney U test. Holm’s method was applied to adjust for multiple comparisons, with a significance level of 0.05 set for all statistical tests.

## 3. Results

A total of 459 histopathological cases were evaluated across the two AI models. Inter-rater reliability between the two OMF pathologists was excellent. The reference standard demonstrated perfect agreement (Cohen’s κ = 1.00), and descriptive scoring demonstrated very high concordance, with an intraclass correlation coefficient of ICC = 0.992 (95% CI: 0.992–0.993) when all cases, including those assigned a score of 0, were included, and ICC = 0.874 (0.840–0.901) when restricted to correctly diagnosed cases. This high inter-rater agreement supported the reliability of the gold-standard labels. The o3 model exhibited a markedly longer response latency, with a mean thinking time of 97.9 ± 76.0 seconds, whereas GPT-4o returned outputs almost instantaneously.

The board-certified general pathologist (non-OMF) correctly diagnosed 26 out of 92 cases (28.3%, 95% CI: 20.1–38.2%), underscoring the overall difficulty level of the selected case set. In intra-model reproducibility, GPT-4o produced identical diagnostic outputs in 53 of 92 repeated cases (57.6%; 95% CI: 47.4–67.2%), whereas o3 was consistent in 37 of 92 cases (40.2%, 95% CI: 30.8–50.4%), a difference that was statistically significant (χ² = 5.57; P = 0.018).

**Fig 2:**
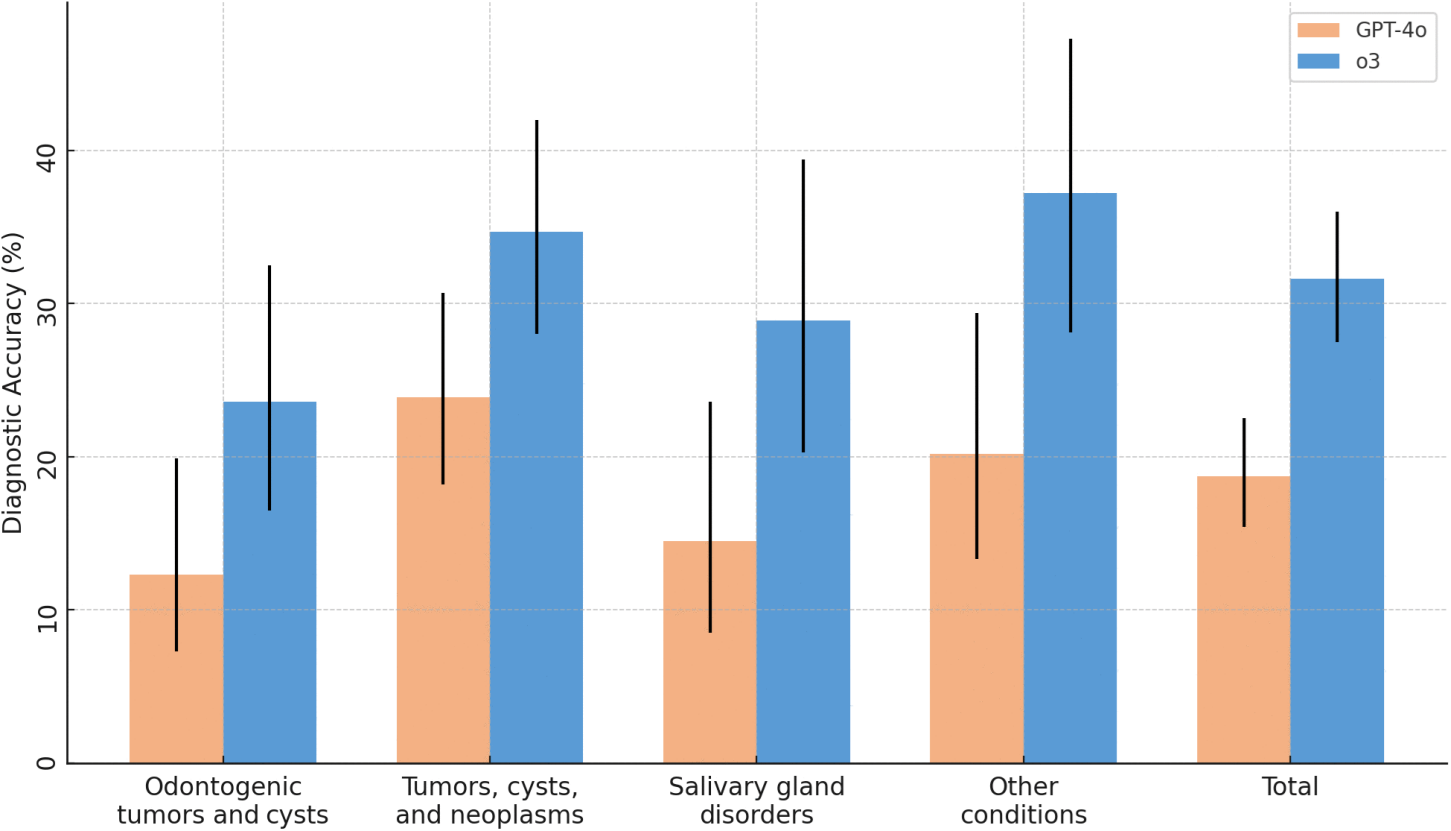
Grouped bar chart showing diagnostic accuracy by category and model.

**Fig 3:**
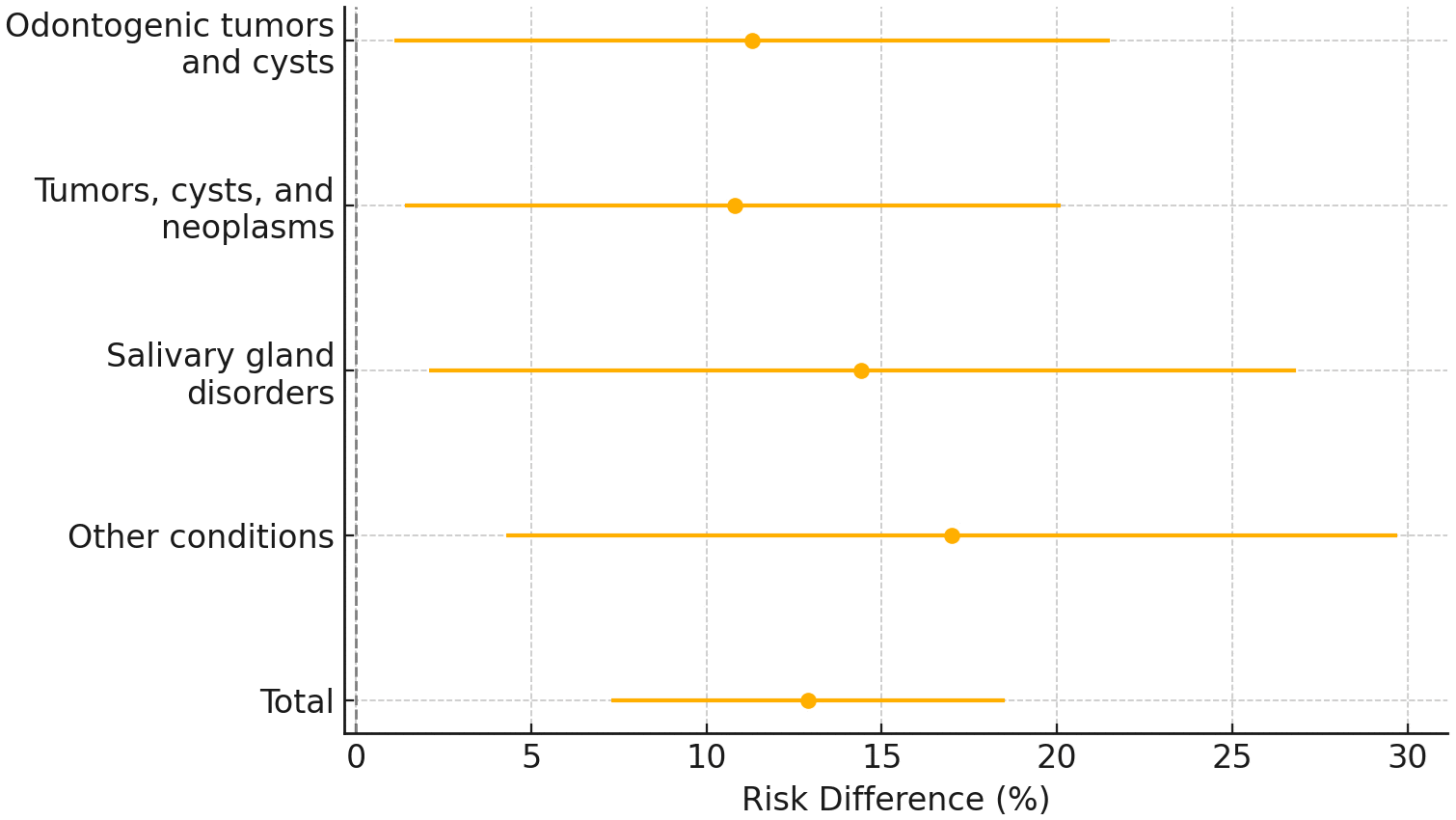
Forest plot showing risk difference between o3 and GPT-4o with 95% confidence intervals.

Across the full sample, the advanced reasoning o3 model achieved a diagnostic accuracy of 31.6% (95% CI 27.5–36.0%), significantly exceeding that of the baseline GPT-4o model at 18.7% (15.4–22.5%), corresponding to an absolute improvement of 12.9 percentage points (χ² = 20.1, P < 0.001; Table 1). The greatest absolute gain was observed for the heterogeneous other-condition category, where accuracy increased from 20.2% (95% CI: 13.3–29.4, Fig 2) to 37.2% (28.1–47.3), yielding a 17.0 percentage point improvement (P = 0.040, Holm-adjusted, Fig 3). Salivary gland disorders likewise showed a notable increase, from 14.5% (8.5–23.6) to 28.9% (20.3–39.4), an absolute gain of 14.4 points (P = 0.072). For tumours, cysts and neoplasms, accuracy rose from 23.9% (18.2–30.7) to 34.7% (28.0–42.0), corresponding to a 10.8-point improvement (P = 0.072). Odontogenic tumours and cysts improved from 12.3% (7.3–19.9) to 23.6% (16.5–32.5), with an absolute gain of 11.3 points (P = 0.072). No diagnostic group favoured the baseline model. In general, diagnostic accuracy was lowest for odontogenic tumors and cysts, followed by salivary gland disorders.

**Table 1:** Diagnostic performance of baseline large‐language model GPT‐4o and advanced reasoning model o3 across six categories of oral and maxillofacial pathology.

Category	n	GPT-4o	o3	Risk difference% (95% CI)	P Value
Odontogenic tumors and cysts	106	12.3 (7.3–19.9)	23.6 (16.5–32.5)	11.3 (1.1–21.5)	0.072
Tumors, cysts, and neoplasms	176	23.9 (18.2–30.7)	34.7 (28.0–42.0)	10.8 (1.4–20.1)	0.072
Salivary gland disorders	83	14.5 (8.5–23.6)	28.9 (20.3–39.4)	14.4 (2.1–26.8)	0.072
Other conditions	94	20.2 (13.3–29.4)	37.2 (28.1–47.3)	17.0 (4.3–29.7)	0.040
Total	459	18.7 (15.4–22.5)	31.6 (27.5–36.0)	12.9 (7.3–18.5)	<0.001

Data are expressed as the number of cases (n) and the corresponding percentage of correctly diagnosed cases, with 95% confidence intervals calculated using the Wilson method. χ^2^ test with Holm-adjusted P values was used to compare diagnostic accuracy between models.

**Fig 4:**
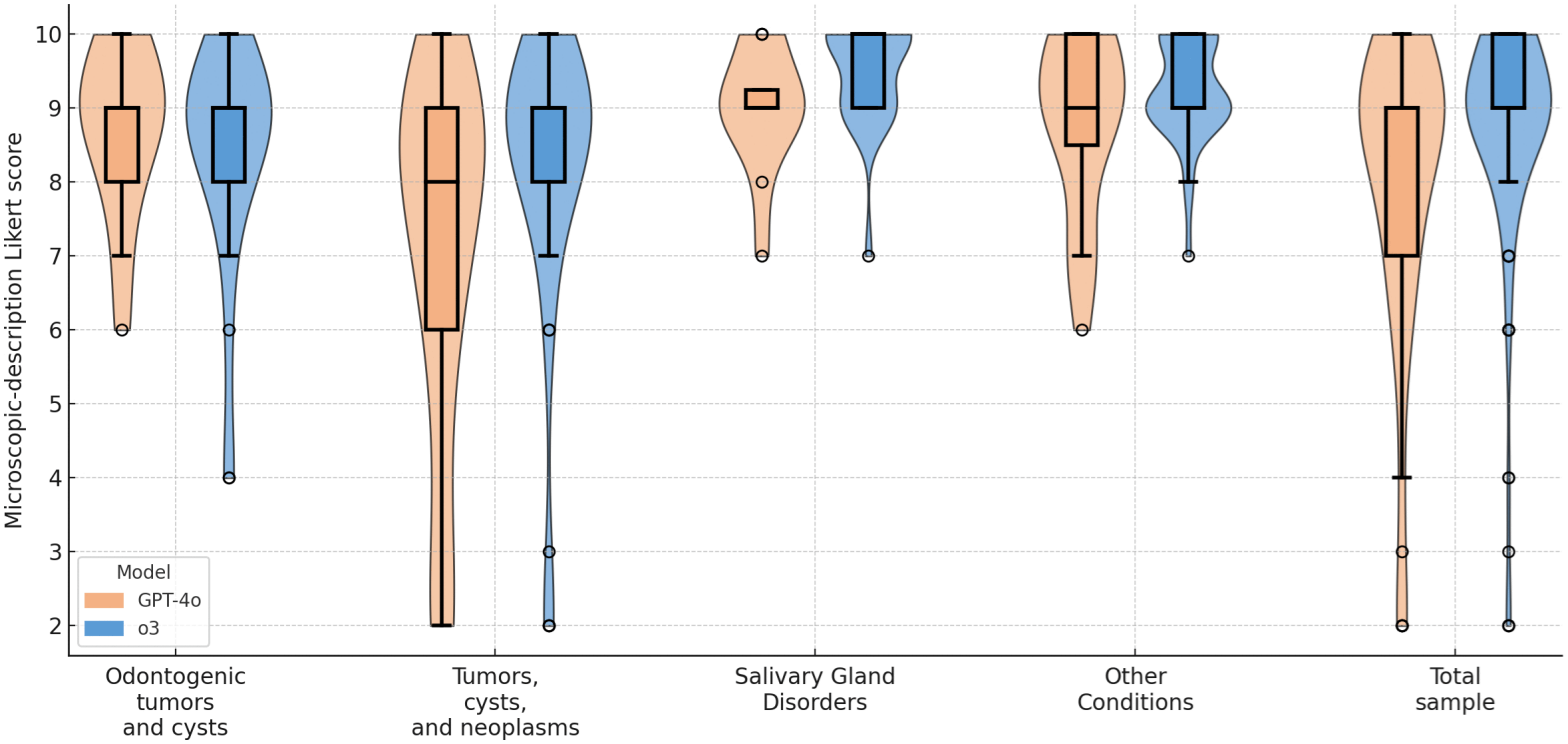
Box-and-violin plot showing microscopic-description Likert scores.

When including all cases (with incorrect diagnoses scored as 0), GPT-4o achieved a mean score of 1.49 ± 3.24 (95% CI: 1.19–1.79), significantly lower than the score of 2.74 ± 4.14 (2.36–3.12) obtained by o3 (Mann-Whitney, *P* < 0.001). When considering only correct diagnoses, GPT-4o continued to yield lower scores, averaging 7.95 ± 2.10 (7.50–8.40) compared with 8.67 ± 1.63 (8.40–8.94) for o3 (Mann-Whitney, *P* = 0.003, Table 2). When stratified by diagnostic category, o3 continued to yield higher scores than GPT-4o across all subgroups (Fig 4). The difference in the non-odontogenic tumor-cysts-neoplasms group was statistically significant (median 9 vs. 8; IQR 8–9 vs. 6–9; mean 8.13 ± 2.03 vs. 7.05 ± 2.49; P = 0.036, Holm-adjusted), while comparisons in odontogenic tumors and cysts (P = 0.593), salivary-gland disorders (P = 0.144), and other conditions (P = 0.498) remained non-significant.

**Table 2:** Microscopic-description Likert scores for correctly diagnosed cases by diagnostic category and total sample.

	GPT-4o				o3				P Value
	n	Median (Range)	IQR (Q1–Q3)	Mean±SD	n	Median (Range)	IQR (Q1–Q3)	Mean±SD	
Odontogenic tumors and cysts	13	9 (6–10)	8–9	8.69 ± 1.18	25	9 (4–10)	8–9	8.36 ± 1.58	0.593
Tumors, cysts, and neoplasms	42	8 (2–10)	6–9	7.05 ± 2.49	61	9 (2–10)	8–9	8.13 ± 2.03	0.036
Salivary Gland Disorders	12	9 (7–10)	9–9.25	9.00 ± 0.85	24	10 (7–10)	9–10	9.50 ± 0.72	0.144
Other Conditions	19	9 (6–10)	8.5–10	8.79 ± 1.23	35	9 (7–10)	9–10	9.26 ± 0.70	0.498
Total	86	9 (2–10)	7–9	7.95 ± 2.10	145	9 (2–10)	9–10	8.67 ± 1.63	0.003

IQR, interquartile range; Q1, first quartile; Q3, third quartile; SD, standard deviation. Scores of 0 (assigned to incorrect diagnoses) were excluded. P values derive from two-sided Mann–Whitney U tests between models and are Holm-adjusted for multiple comparisons.

## 4. Discussion

We observed that the reasoning‐enhanced o3 model consistently outperformed the baseline GPT-4o model across all diagnostic categories, yielding roughly a 13 percentage-point increase in overall accuracy and generating more detailed microscopic descriptions for correctly diagnosed cases. These findings suggest that the advantages of advanced reasoning extend beyond incremental improvements in classification metrics. By producing richer explanatory outputs, o3 has the potential to support pathologists in cases where nuanced image features are critical. In turn, this could help mitigate the growing demand for expert review in underserved settings. Although diagnostic performance remains limited in cases with intricate histological features, our results underscore a meaningful step forward, as o3’s multi‐step reasoning and deeper image‐comparison mechanisms distinctly benefit scenarios with overt morphological patterns. These strategic enhancements to LLM architectures can bridge gaps in automated histopathological analysis and pave the way for more effective AI-pathologist collaboration.

While absolute accuracy appears modest, the reference standard is reliable, and the general pathologist (non-OMF) benchmark indicates that this case set is genuinely difficult for non-specialists. The task also required zero-shot, multi-image reasoning across magnifications, which is known to depress absolute accuracy while still allowing meaningful relative gains between model variants.

GPT-o3 processes histopathologic images through a deliberate two-stage pipeline. A high-resolution Vision Transformer encoder first converts each tile into patch tokens and compares them with a cached library of visual prototypes. The language module then enters an iterative reasoning loop: it drafts a latent “plan,” evaluates that plan against the matched exemplars, revises it, and finally produces a narrative enriched with pathology-specific ontology terms [20,21]. The cost of this rigor is latency, because the model keeps its chain of thought private until planning converges, users experience a brief “thinking” pause. In contrast, GPT-4o removes that pause by fusing vision and language in a single-pass decoder that streams tokens as soon as they are generated. Image features remain inside the shared decoder, no external planning loop is invoked, and lighter safety filtering avoids post-processing delays. The result is real-time interaction and fluent prose, but the absence of prototype verification makes GPT-4o somewhat more prone to hallucinating low-frequency lesions when the histology is borderline or under-represented in training [20–22].

An important observation from our study is that the advanced reasoning model o3, despite its enhanced diagnostic accuracy and richer interpretive outputs, exhibits lower reproducibility compared to the baseline GPT-4o model. This discrepancy likely arises from o3’s multi-stage, chain-of-thought reasoning process. Although this process enables it to generate more nuanced and detailed interpretations, the inherent variability in each iteration can lead to different outputs for the same case when run multiple times. In contrast, GPT-4o employs a more streamlined, single-pass approach that fuses visual and linguistic information in real time, thereby enhancing output consistency. This trade-off between deeper, more detailed reasoning and reproducibility illustrates a critical area for further optimization in AI-driven diagnostic tools, underscoring the need to balance advanced reasoning capabilities with consistent performance in clinical settings.

Prior evaluations of LLMs in histopathology typically fed the model a single image per case, yielding substantially higher accuracy, often between 60–87% on organ‐specific tasks, such as liver, colon, and kidney, and approximately 60% on mixed oral biopsy descriptions [1,7,10–13]. In contrast, our study presented 2–5 high‐resolution, textbook‐sourced HE images at varying magnifications in a single prompt, yet achieved only 18.7% accuracy for GPT-4o and 31.6% for the newer o3 model. Three main factors likely explain this discrepancy. First, feeding multiple images, and especially at different zoom levels, requires the model to integrate multi‐scale visual information, which prior work did not test. For example, Ding et al. [1] found GPT-4’s performance actually declined when more than one image was provided at a time. Second, our textbook images are novel to the model, earlier studies often used web‐derived or publicly available slides that LLMs had effectively “seen” during pretraining. These familiar, single‐image inputs are easier for GPT-4 to recognize than high‐quality, curated textbook photomicrographs. Third, the inherent difficulty of our OMF case set, featuring rare entities such as odontogenic tumors and specialized salivary gland neoplasms, was underscored by an accuracy rate of approximately 28% among non‐specialist pathologists on the same images. Thus, although o3’s 31.6% accuracy remains lower than the 60–88% reported in simpler, organ‐focused tasks, this gap primarily reflects the complexity of integrating multiple high‐resolution views and the niche nature of our lesion set, rather than a fundamental limitation of LLM technology.

Moreover, Ferber et al. [23] demonstrated that vision-enabled LLMs such as GPT-4V can approach similarly high accuracy (83−88%) on colorectal tissue and lymph node metastasis classification with using ten‐shot prompts handful of in-context examples, whereas zero-shot performance remains substantially lower (40−60%). Similarly, Ono et al. [24] directly compared GPT-4V with a YOLOv8 CNN on neurodegenerative disease histopathology and found that GPT-4 V’s zero-shot accuracy was approximately 40% but increased to approximately 90% with a 20-shot prompt, matching the YOLOv8 CNN, which required 100 labelled examples to achieve equivalent performance. However, these benchmarks were obtained using relatively uniform open-source datasets in which images were acquired under fixed protocols with consistent HE staining, patch size, and resolution.

Regarding microscopic descriptions, GPT-4o consistently scored lower than o3, even when analyses were limited to correctly diagnosed cases, indicating that GPT-4o often omitted key histopathological details compared to o3’s more thorough explanations. This discrepancy mirrors findings by Apornvirat et al. [11], where ChatGPT’s descriptions achieved roughly half the quality of those produced by junior and senior residents, despite including some relevant features. Similarly, Li et al. [12] reported that Claude 3 Opus, while linguistically fluent, ranked poorly on accuracy and clinical relevance when describing renal pathology slides. Although o3’s description scores demonstrate a clear improvement over GPT-4o, occasional omissions of subtle diagnostic features suggest that further refinement, through domain-specific fine-tuning or optimized prompting, could help ensure consistently comprehensive explanations.

On the other hand, several convolutional neural network (CNN) models and ViT models have achieved very high accuracy in histopathological classification. For example, Emegano et al. [25] showed that a ResNet-50-based model demonstrated 98% accuracy on prostate biopsy images. Elazab et al. [26] found that combining ResNet-50 with YOLOv5 for tumor localization and an XGBoost classifier achieved 97% accuracy and a Dice coefficient of 0.97 for glioma grading using The Cancer Genome Atlas whole slide images. EfficientNet variants likewise perform robustly, with Anjum et al. [27] reporting that EfficientNet-B2 reached 97% accuracy on a 25000-image lung and colorectal dataset, and Albalawi et al. [28] finding that EfficientNet-B3 distinguished normal mucosa from oral squamous cell carcinoma with 99% accuracy and nearly perfect precision and recall. Li et al. [29] developed a ViT-based ViT-WSI model that predicted IDH1 mutation status with an area under the curve of 0.960, TP53 mutation with an area under the curve of 0.874, and MGMT methylation with an area under the curve of 0.845 directly from 6173 glioma whole slide images. Finally, Rong et al. [30] reported that HD-YOLO outperformed existing methods by 5% to 12% in nuclear detection accuracy across lung, liver, and breast cancer whole slide images while processing each patch in 0.01 seconds. Although these models deliver consistent performance on in‐distribution images, they depend on extensive annotations, uniform staining-scanning protocols, and substantial computational resources, often impractical in routine pathology labs. Furthermore, unlike CNNs and ViTs that produce only categorical labels and depend on post hoc tools such as Gradient-weighted Class Activation Mapping for interpretability, GPT‐4o and o3 generate human‐readable descriptions of morphological features [31]. Moreover, although somewhat less accurate on pure image‐classification tasks, GPT‐4o and o3 can accept heterogeneous inputs (multiple magnifications, varied stains, and rare lesions) without retraining. Additionally, they are accessible via cloud‐based interfaces and provide rapid diagnostic impressions along with explanatory rationale, all with minimal setup.

In evaluating lesions, the model demonstrated systematic reasoning flaws that go well beyond simply reporting a percentage of errors. For instance, when attempting to differentiate between acinic cell carcinoma and secretory carcinoma, o3 focused primarily on superficial high-power features, such as the presence of clear or bubbly cytoplasm, without adequately correlating these with low-power architectural details like lobular preservation or the pattern of infiltration. Similarly, in the salivary gland file, the model misclassified lesions by overemphasizing keratinization, frequently labeling an apical radicular cyst as a keratinizing odontogenic tumor and dismissing key contextual clues such as a nonkeratinized, inflamed lining. In the odontogenic domain, the chain of thought was often incomplete, while the model sometimes detected a thin, uniform epithelial lining at low power, it would neglect critical high-power features (for example, the presence or absence of basal cell palisading) that distinguish an odontogenic keratocyst from a dentigerous cyst. Moreover, the model showed inconsistency in evaluating ghost cells, amyloid-like extracellular material, and calcific deposits, including Liesegang rings, which are pathognomonic for lesions like calcifying epithelial odontogenic tumors. Another failure mode was the overreliance on isolated morphological findings. In several cases, it interpreted a solitary attribute, such as a thin epithelial lining, as diagnostic of a dentigerous cyst without integrating the essential clinical context, such as the association with an impacted tooth, or other architectural cues. Finally, a lack of domain-specific fine-tuning led to further degradation of performance, as the model’s reasoning did not consistently integrate multi-scale information from both low- and high-power examinations. These examples underscore that the errors are not merely quantitative but arise from an incomplete and inconsistent chain of reasoning that fails to merge subtle architectural patterns with detailed cytological features, a shortcoming that must be addressed with more robust, domain-focused training.

By contrast, GPT-5–a unified multimodal model supporting both rapid responses and extended reasoning–postdates this study, with reported state-of-the-art visual-perception performance and substantially fewer hallucinations than GPT-4o and o3 [32]. It also reports improved instruction-following and agentic tool use (including ecuting longer chains of tool calls) and adds API controls for steerability. While these advances may plausibly benefit zero-shot, multi-image histopathology, GPT-5 was unavailable at the time of data collection, and our results should be interpreted as conservative for the current model family. A like-for-like replication using GPT-5 is warranted to quantify any delta under identical task and scoring conditions.

From a clinical standpoint, the current performance profile suggests that o3 and GPT-4o are best positioned as adjunctive tools rather than autonomous diagnosticians. With overall accuracies of approximately 32% and 19%, respectively, and limited reproducibility, neither model can be safely used to sign out cases or to replace expert OMF pathologists. Instead, potential near-term applications include generating preliminary microscopic descriptions, suggesting differential diagnoses for non-specialists, and supporting teaching or self-directed learning in settings where subspecialty expertise is scarce. In such scenarios, human pathologists remain responsible for integrating clinical, radiologic, and histologic findings, with LLM outputs serving only as a structured second opinion or educational aid.

Despite offering novel insights into the potential of reasoning-augmented large language models (LLMs) for OMF histopathology, our study is subject to several interconnected limitations. First, all analyses relied on high-resolution, textbook-sourced hematoxylin-and-eosin images that lack the variability and artefacts inherent in routine clinical specimens; this may have overestimated model generalizability to real-world practice. Second, diagnostic prompts incorporated multiple magnification levels to mimic realistic challenges, but this multi-scale framework likely accentuated the models’ difficulties in integrating visual information across scales, contributing to the overall low accuracy observed. Third, both o3 and GPT-4o were evaluated strictly in a zero-shot setting, without in-context examples or pathology-specific fine-tuning, which underrepresents their achievable performance and limits interpretive nuance. Fourth, we did not include any clinical metadata, such as patient history or lesion site, precluding true multimodal reasoning that is critical in everyday diagnostics. Fifth, the single-center, cross-sectional design and modest case numbers within each diagnostic subgroup, especially for rare entities, constrain statistical power and external validity, and they prevent assessment of these models’ performance within dynamic clinical workflows. Sixth, although the advanced reasoning model generated richer explanatory outputs, its substantially longer inference latency may impede seamless integration into high-throughput environments, and its lower reproducibility underscores a trade-off between detailed interpretive depth and consistency. Additionally, we did not evaluate GPT-5; therefore, the generalizability of our findings to the newest model is unknown and should be tested prospectively. Finally, broader ethical, regulatory, and cost-benefit considerations remain unaddressed, as neither model underwent domain-adapted training for OMF pathology, and we did not explore data governance or potential bias in decision-making. Collectively, these limitations highlight both the promise and the current gaps that future studies must address, through multicenter, prospective designs, inclusion of diverse clinical specimens and metadata, targeted fine-tuning, and rigorous evaluation of workflow integration and ethical implications, to fully realize the utility of reasoning-augmented LLMs in histopathological diagnostics.

## 5. Conclusions

In summary, our comparative evaluation shows that the reasoning-enhanced large-language model o3 outperforms baseline GPT-4o in OMF histopathology, achieving a 13-percentage-point absolute gain in diagnostic accuracy and producing more granular microscopic explanations. Although these gains come at the cost of slower inference and reduced reproducibility, they illustrate how structured, multi-step reasoning within vision–language architectures can narrow the gap between LLMs and task-specific convolutional networks and point toward broadly deployable, annotation-light tools. Clinically, the current performance profile suggests that such models are best positioned as adjunctive “copilots” for generating preliminary descriptions, differential diagnoses, and educational feedback, while expert OMF pathologists retain full responsibility for case sign-out. Future work should couple these advanced models with domain-tuned training, multimodal clinical metadata, and workflow-level prospective validation to improve robustness and generalizability. Collectively, our findings support next-generation reasoning LLMs as promising collaborators, rather than substitutes, in the pursuit of more accurate, transparent, and accessible histopathological diagnostics.

## Supporting information

S1 File Reference diagnoses and AI model outputs. (DOCX)

S2 File Dataset. (XLSX)
