# Supplementary material for: Comparative study of advanced reasoning versus baseline large-language models for histopathological diagnosis in oral and maxillofacial pathology
Source: PLoS One. 2025 Dec 31;20(12):e0340220. doi: 10.1371/journal.pone.0340220 (PMC12755752; doi:10.1371/journal.pone.0340220)
Supplement: S1 File — (DOCX) [file pone.0340220.s001.docx]

**Supplementary Information file S1: Reference diagnoses, key microscopic features, and AI model outputs – text adapted for copyright compliance**

This supplemental file lists, for each case, the reference diagnosis and its defining microscopic features as adapted from the source textbooks (all text rewritten to ensure copyright compliance), alongside the corresponding diagnostic responses generated by GPT-4o and o3.

Developmental Defects of the Oral and Maxillofacial Region

| **Fig_no** | **Ground truth** | **GPT 4o answer** | **o3 answer** |
| --- | --- | --- | --- |
| 1.69 | Diagnosis: A cystic lesion of the epidermoid type; Key Microscopic Features: On low magnification, a keratin-filled cystic space is evident, and on high magnification, the lining is composed of stratified squamous epithelium producing orthokeratin. | Single Most Likely Histopathological Diagnosis:  Oral lymphangioma (also known as lymphatic malformation)  Key Microscopic Features Supporting the Diagnosis:  Low Magnification Image:  Multiple, irregularly dilated spaces in the subepithelial connective tissue, many of which appear empty or contain scant eosinophilic fluid—these are characteristic of dilated lymphatic vessels.  The lumina are lined by thin, flattened endothelial cells, and the overall architecture appears lobular and non-encapsulated.  The overlying epithelium is intact, without signs of dysplasia or ulceration, further supporting a benign developmental lesion.  High Magnification Image:  Thin-walled, interconnected vascular spaces located just beneath the stratified squamous epithelium.  The lumina are devoid of red blood cells, distinguishing lymphangiomas from hemangiomas.  The epithelium is parakeratinized stratified squamous epithelium, which is typical for oral mucosa and often found overlying such malformations.  Supporting stroma shows minimal inflammation, which is consistent with a developmental rather than reactive or inflammatory lesion. | Histopathological diagnosis (single best answer)  Orthokeratinised odontogenic cyst (OOC) – the orthokeratinised developmental cystic analogue of the odontogenic keratocyst.  Key microscopic features supporting the diagnosis  Field‑of‑view Histological detail Diagnostic value  Low magnification (first image) • A well‑circumscribed cystic space filled with thick, eosinophilic, laminated material that floats free in the lumen.  • The material forms flat “shreds” and whorled plates – typical of orthokeratin flakes that have sloughed from the surface lining.  • Cyst wall is thin and fibrous, with scant inflammation. Confirms a cystic lesion and shows profuse keratin desquamation, the hallmark of OOC.  High magnification (second image) • Cyst lining is uniformly thin (≈4–8 cell layers).  • Surface shows a basket‑weave pattern of anucleate orthokeratin.  • Immediately beneath the orthokeratin is a well‑developed granular (keratohyaline) cell layer.  • Basal cells are low cuboidal/flattened and non‑palisaded, without nuclear hyperchromatism.  • No budding or daughter cyst formation. The combination of an orthokeratinised surface, prominent granular layer and bland, non‑palisaded basal cells distinguishes OOC from the parakeratinised odontogenic keratocyst and other cysts. |
| 1.80 | Diagnosis: A lymphoepithelial cyst of the oral mucosa; Key Microscopic Features: Under low power, a keratin-filled cyst lies beneath the mucosal surface with lymphoid tissue incorporated into its wall, and under high power, lymphoid aggregates are observed adjacent to the cystic lining. | Based on the provided hematoxylin-and-eosin (H&E) stained images at low and high magnification, the single most likely histopathological diagnosis is:  Dermoid Cyst  Key Microscopic Features Justifying the Diagnosis:  Cystic Structure:  Both images show a well-circumscribed cystic lesion with a clear lumen surrounded by a fibrous capsule.  Lumen Contents:  The cystic cavity contains keratinaceous material, visible as eosinophilic, lamellated debris in the lumen – a hallmark of keratinizing cysts.  Epithelial Lining:  The cyst is lined by orthokeratinized stratified squamous epithelium (seen clearly in the second image). The lining has a granular cell layer and flattened surface keratin, consistent with epidermoid-type differentiation.  Skin Appendages:  Within the cyst wall (especially in the low magnification image), there are sebaceous glands and possibly hair follicles – a defining feature of dermoid cysts (as opposed to epidermoid cysts, which lack adnexal structures).  Surrounding Connective Tissue:  Dense fibrous connective tissue stroma with chronic inflammatory infiltrate can be seen, especially at the base of the epithelial lining. | Histopathological diagnosis  → Oral (branchial) lympho‑epithelial cyst  Key microscopic features supporting this diagnosis  Microscopic finding How it appears in the submitted slides Diagnostic relevance  Lamellated keratin‐filled lumen Central cavity packed with concentric, eosinophilic, anucleate keratin whorls (first image, pink core) Indicates a keratinising epithelial‑lined cyst rather than a mucous or odontogenic cyst  Very thin stratified squamous epithelial lining (often parakeratinised) A 2 – 4‑cell‑layer‑thick epithelium seen bordering the keratin (second image, just above the white cleft) Characteristic of lympho‑epithelial cysts; thicker, rete‑ridge epithelium would suggest another entity  Dense subepithelial lymphoid tissue with germinal centres Broad rim of dark‑purple lymphocytes with follicular architecture surrounding the entire cyst wall (both images) The defining feature that couples the epithelium to organised lymphoid tissue, giving the lesion its name  Sharp epithelial–connective tissue interface with no adnexal appendages Clear separation between epithelium and lymphoid stroma; absence of hair follicles, sebaceous or sweat glands Helps exclude dermoid / epidermoid cysts, which show skin adnexal structures  No odontogenic epithelial rests or calcifications No ghost cells, no dystrophic calcification, no palisaded basal layer Rules out odontogenic keratocyst and calcifying odontogenic cyst |

Bacterial Infections

| **Fig_no** | **Ground truth** | **GPT 4o answer** | **o3 answer** |
| --- | --- | --- | --- |
| 5.16 | Diagnosis: Secondary Syphilis, Condyloma Lata; Key Microscopic Features: On low-power examination, the biopsy reveals papillary overgrowth of the epithelium accompanied by a dense plasma cell–predominant infiltrate within the connective tissue; on high-power view, there is marked movement of neutrophils into the epithelial layer. | 🔬 Single Most Likely Histopathological Diagnosis  Secondary Syphilis (Oral Mucous Patch or Condyloma Lata Stage)  🧠 Key Microscopic Features Supporting the Diagnosis:  Epithelium:  Markedly acanthotic stratified squamous epithelium with elongated rete ridges.  Superficial epithelial erosion and spongiosis—common in mucosal lesions of secondary syphilis.  Inflammatory Infiltrate:  Dense lichenoid and perivascular infiltrate predominantly composed of plasma cells (hallmark of syphilitic lesions).  Mixed with lymphocytes and histiocytes.  Infiltrate extends deep into the connective tissue papillae and perivascular areas.  Vascular Changes:  Evidence of endothelial swelling and vascular proliferation, consistent with vasculitis, which is often seen in syphilis.  Surface Changes:  Ulceration or desquamation of surface epithelium in high-power field, sometimes covered with fibrin or inflammatory exudate. | Histopathological diagnosis  Condyloma lata (secondary syphilitic papillary lesion) – an oral manifestation of secondary syphilis.  Key microscopic features that support this diagnosis  Area examined Diagnostic findings Significance  Surface epithelium • Stratified‑squamous epithelium with pronounced papillomatosis/psoriasiform hyperplasia.  • Broad, club‑shaped rete ridges extend deeply and irregularly. Typical architecture of condyloma lata; resembles psoriasiform changes produced by Treponema pallidum–induced epithelial irritation.  Intra‑epithelial changes • Heavy exocytosis of inflammatory cells with well‑formed intra‑epithelial lympho‑plasmacytic micro‑collections (“micro‑abscess‑like” foci). Exocytosis and micro‑collections are characteristic for secondary syphilitic lesions.  Surface exudate / ulceration • Fibrinopurulent crust and epithelial erosion in focal areas. Common in condyloma lata and mucous patches of secondary syphilis.  Lamina propria • Dense, band‑like infiltrate rich in plasma cells and lymphocytes occupying papillary and deeper connective tissue. A plasmacyte‑predominant infiltrate is the classic clue to syphilis; helps distinguish it from other papillary/inflammatory lesions.  Absence of dysplasia & viral cytopathic effect • No koilocytosis, viral inclusions or epithelial atypia. Helps rule out HPV‑related condyloma acuminatum and verrucous carcinoma. |

Allergies and Immunologic Diseases

| **Fig_no** | **Ground truth** | **GPT 4o answer** | **o3 answer** |
| --- | --- | --- | --- |
| 9.23 | Diagnosis: Orofacial Granulomatosis; Key Microscopic Features: Granulomatous inflammatory clusters surround scattered vessels, and the inset highlights histiocytes and multinucleated giant cells within those granulomas | Histopathological Diagnosis:  Sarcoidosis  Key Microscopic Features Justifying the Diagnosis:  Non-caseating Granulomas:  Both images clearly show tight clusters of epithelioid histiocytes (macrophages with elongated, pale-staining nuclei), forming granulomas without central necrosis.  Multinucleated giant cells are also present within the granulomas, a hallmark of granulomatous inflammation.  Lack of Caseation:  Unlike infections such as tuberculosis, which show caseating necrosis in granulomas, these granulomas appear non-necrotizing, supporting a diagnosis of sarcoidosis.  Sparse Peripheral Lymphocytes:  The granulomas are relatively "naked," meaning there is a sparse lymphocytic cuff around them—another classic feature of sarcoid granulomas.  Distribution in the Connective Tissue:  The low-power image shows these granulomas are distributed throughout the connective tissue beneath the oral mucosal epithelium, which is a common site for oral manifestations of sarcoidosis.  Sarcoidosis is a multisystem granulomatous disorder of unknown etiology, and oral involvement—though less common—can present with lesions on the gingiva, lips, or buccal mucosa.  Would you like a comparison with other granulomatous diseases in the oral cavity for differential diagnosis? | Most likely histopathological diagnosis  → Sarcoidosis (non‑caseating granulomatous inflammation)  Key microscopic features visible in the two H‑E sections  Field What is seen Why it supports sarcoidosis  Low‑power overview Numerous well‑circumscribed, discrete nodules scattered throughout the lamina propria/submucosa, immediately beneath an intact stratified squamous epithelium. Granulomas in sarcoidosis are typically multiple, tightly grouped and “naked” (scant surrounding lymphocytic cuff), producing a peppered appearance throughout the connective tissue.  Cellular composition of each nodule Aggregates of epithelioid histiocytes with abundant eosinophilic cytoplasm; peripheral scant lymphocytes; occasional multinucleated giant cells. Sarcoid granulomas are composed predominantly of epithelioid macrophages with few accompanying lymphocytes and scattered Langhans‑type or foreign‑body‑type giant cells.  Absence of necrosis No central caseation, fibrinoid necrosis or suppuration is present. Non‑caseating (non‑necrotizing) granulomas are characteristic of sarcoidosis and help exclude infectious granulomatous diseases such as tuberculosis.  Intranodular inclusions (high‑power image) Within some giant cells/histiocytes there are rounded, eosinophilic cytoplasmic inclusions (Schaumann‑type calcific bodies / asteroid bodies). Schaumann or asteroid bodies are classic—but not pathognomonic—cytoplasmic inclusions frequently encountered in sarcoid granulomas. Their identification further supports the diagnosis.  Taken together, the pattern of multiple “naked” non‑caseating granulomas composed of epithelioid histiocytes and giant cells, without necrosis, and containing Schaumann/asteroid bodies is textbook for sarcoidosis in the oral and maxillofacial region. |

Epithelial Pathology

| **Fig_no** | **Ground truth** | **GPT 4o answer** | **o3 answer** |
| --- | --- | --- | --- |
| 10.8 | Diagnosis: Verruca Vulgaris; Key Microscopic Features: At low magnification, multiple papillary projections are enveloped by a hyperkeratotic, stratified squamous epithelium with elongated rete ridges at the lesion’s periphery converging centripetally; at higher magnification, distinct koilocytes are observed within the superficial epithelial layers. | Histopathological Diagnosis:  Verrucous carcinoma  Key Microscopic Features Justifying the Diagnosis:  Exophytic growth pattern (Low magnification, Image 1):  The lesion shows broad-based, pushing rete ridges extending downward.  The surface is papillary or verrucous, characteristic of a warty, cauliflower-like growth.  Hyperkeratosis and parakeratosis (Both images):  Prominent thickening of the keratin layer is evident, particularly over the surface projections.  Bulbous rete ridges with pushing borders (Low magnification):  Rather than infiltrative or destructive, the rete ridges are broad and push into the underlying stroma in a well-demarcated manner.  This is a hallmark of verrucous carcinoma distinguishing it from conventional squamous cell carcinoma.  Minimal cytologic atypia (High magnification, Image 2):  Despite the thick epithelial proliferation, the individual cells show bland nuclei with minimal pleomorphism and low mitotic activity.  Absence of marked dysplasia, yet with clear architectural disturbance.  Keratin plugging (High magnification):  The second image demonstrates keratin-filled invaginations or crypts, commonly found in verrucous lesions.  Summary:  The combination of verrucous surface architecture, pushing epithelial downgrowths, minimal cytological atypia, and absence of infiltrative behavior supports the diagnosis of verrucous carcinoma, a well-differentiated variant of squamous cell carcinoma. | Histopathological diagnosis – Verruca vulgaris (common wart, HPV‑related).  Key microscopic features that support this diagnosis  Field Findings Significance  Low‑power architecture • Exophytic, verruciform surface with sharply pointed papillary projections.  • Down‑growing rete ridges are long, thin and converge toward the centre of each papilla, producing a characteristic “cupping” pattern. Typical overall silhouette of verruca vulgaris rather than the broader, blunted fronds of a condyloma or the bulbous pushing fronts of a verrucous carcinoma.  Surface & keratin layer • Marked hyper‑ and parakeratosis (“church‑spire” keratin).  • Prominent granular cell layer containing coarse keratohyalin granules. Heavy keratin production and granular‑layer thickening are hallmarks of a common wart.  Spinous‑layer cytology • Numerous koilocytes – enlarged squamous cells with clear perinuclear halos and pyknotic, raisinoid nuclei – best appreciated in the higher‑power image. Koilocytosis is a classic cytopathic effect of HPV infection, seen in verruca vulgaris.  Atypia & invasion • Little or no cytologic atypia; basement membrane remains intact; no invasive irregular cords or buds. Confirms a benign HPV‑induced proliferation rather than dysplasia/carcinoma.  Stromal core • Each papillary projection contains a thin fibrovascular connective‑tissue core with only mild chronic inflammation. Supports a benign papillary epithelial lesion.  The combination of a pointed, verruciform architecture, elongated “cupping” rete ridges, granular‑layer hyperplasia with coarse keratohyalin granules, and conspicuous koilocytosis is diagnostic of verruca vulgaris in the oral cavity. |
| 10.24 | Diagnosis: Verruciform Xanthoma; Key Microscopic Features: Under low power, there is mild papillary formation due to hyperparakeratosis with uniformly elongated rete ridges and parakeratin plugging between the papillae; at high power, the connective tissue papillae are almost entirely composed of foamy, lipid-laden macrophages (xanthoma cells). | Based on the provided hematoxylin and eosin (H&E) stained histopathological images at low and high magnification, the single most likely histopathological diagnosis is:  Verrucous Carcinoma  Justification with Key Microscopic Features:  Low Magnification Image:  Papillary/verrucous architecture: Broad, pushing rete ridges extending into the connective tissue rather than showing infiltrative behavior.  Well-differentiated epithelium: There is minimal cytological atypia, maintaining a relatively orderly maturation pattern.  Hyperkeratosis: Surface appears thickened, consistent with a verrucous or warty appearance.  Parakeratin plugging: Keratin appears to fill the invaginations between papillary projections.  High Magnification Image:  Bland cytology: Cells show minimal pleomorphism and mitotic activity, indicating low-grade malignancy.  Abundant keratin production: Eosinophilic cytoplasm and keratin pearls in the superficial layers.  Pushing borders: Epithelial projections extend downward but without a destructive invasion of the stroma (a hallmark of verrucous carcinoma vs. conventional squamous cell carcinoma).  Summary:  Verrucous carcinoma is a well-differentiated variant of squamous cell carcinoma, often seen in the oral cavity, characterized by slow growth, local aggressiveness, and a verrucous surface. The key features of broad pushing rete ridges, bland cytology, hyperkeratosis, and a lack of deep invasion support this diagnosis. | Histopathological diagnosis  Verruca vulgaris (common wart) – an HPV‑induced exophytic squamous epithelial lesion.  Key microscopic features in the two H‑E sections that support this diagnosis  Low‑power view High‑power view Diagnostic relevance  1. Exophytic, papillary architecture – multiple tall, finger‑like projections separated by clefts and covered by hyper‑ ortho/para‑keratin. — Classic wart morphology; distinguishes it from flat epithelial hyperplasias.  2. “Cupping” rete pattern – elongated, pointed rete ridges on either side of each papilla bend toward the centre, producing a deep V‑shaped or cup‑like outline. — Characteristic of verruca vulgaris and rarely seen in other oral papillary lesions.  3. Hypergranulosis – thick granular layer with coarse keratohyalin granules. — Another typical feature of common warts.  — 4. Koilocytosis – numerous enlarged prickle‑cell nuclei surrounded by a clear perinuclear halo, giving a vacuolated “fried‑egg” appearance. Represents HPV‑induced cytopathic change; confirms a viral papillary lesion.  — 5. No epithelial dysplasia – orderly maturation from basal to surface layers; mitoses restricted to basal/parabasal zones. Helps separate verruca from verrucous carcinoma or dysplastic leukoplakia.  The combination of an exophytic papillary surface, converging pointed rete ridges (“cupping”), prominent hypergranulosis, and abundant koilocytes is diagnostic for verruca vulgaris in the oral mucosa. |
| 10.41 | Diagnosis: Oral Melanotic Macule; Key Microscopic Features: At low power, there is increased melanin pigment distributed along the basal epithelial layer; under high magnification, granular brown melanin pigment is evident within the basal keratinocytes. | Based on the two provided hematoxylin and eosin (H&E) stained histological images of the same lesion at different magnifications, the single most likely histopathological diagnosis is:  Epithelial Dysplasia (most consistent with Moderate to Severe Dysplasia)  Key Microscopic Features Supporting the Diagnosis:  1. Architectural Disturbance:  Loss of normal maturation and stratification of the epithelium.  Drop-shaped rete ridges seen in the low magnification image, with hyperplasia and irregular epithelial proliferation into the connective tissue.  Basal cell hyperplasia and expansion of immature cells into suprabasal layers.  2. Cytological Atypia:  Nuclear pleomorphism – variation in nuclear size and shape.  Nuclear hyperchromatism – darkly stained nuclei suggesting increased DNA content.  Increased nuclear-to-cytoplasmic ratio.  Abnormal mitotic figures can be observed, especially in the upper half of the epithelium (suggesting a progression beyond mild dysplasia).  3. Basal Cell Layer Changes (as seen in the high magnification image):  Loss of polarity and crowding of basal cells.  Prominent nucleoli, indicating increased metabolic and proliferative activity.  Mitotic activity in the suprabasal layers, which is abnormal.  Conclusion:  These histopathologic features are consistent with a diagnosis of oral epithelial dysplasia, most likely in the moderate to severe range. This is a premalignant lesion with the potential to progress to oral squamous cell carcinoma if not managed appropriately. | Most likely histopathological diagnosis  Oral melanotic macule (focal oral mucosal melanosis)  Key microscopic features that point to this diagnosis  Feature (seen in the photomicrographs) Diagnostic significance  Heavy, finely‑granular brown pigment confined to the basal cell layer (second, high‑power image) Marks increased melanin production within basal keratinocytes without the formation of nests or upward (pagetoid) spread that would suggest a nevus or melanoma.  Normal (or only slightly increased) number of melanocytes; nuclei appear bland, with no atypia or mitotic activity Rules out melanocytic nevus and malignant melanoma, which show proliferation and/or atypia of melanocytes.  Acanthotic but orderly stratified squamous epithelium with elongated rete ridges yet no architectural or cytologic dysplasia (first, low‑power image) Indicates a benign, reactive mucosal alteration rather than epithelial dysplasia or carcinoma.  Melanin‑laden macrophages (melanophages) in the superficial lamina propria Common “spillage” phenomenon beneath a melanotic macule; supports a benign hypermelanosis rather than a true melanocytic proliferation.  Absence of dendritic melanocytes scattered through the full epithelial thickness Helps distinguish the lesion from oral melanoacanthoma, which shows numerous dendritic melanocytes throughout all epithelial layers.  Together, the combination of intense basal‑layer pigmentation, lack of melanocytic nesting or atypia, orderly epithelium, and subepithelial melanophages is diagnostic of an oral melanotic macule. |
| 10.53 | Diagnosis: Blue Nevus; Key Microscopic Features: Numerous spindle-shaped melanocytes containing abundant melanin are located deep within the lamina propria, aligned parallel to the surface epithelium at low power; at higher magnification, these spindle cells are seen to be intensely pigmented. | Histopathological Diagnosis:  Amalgam Tattoo (Localized Argyrosis)  Key Microscopic Features Supporting the Diagnosis:  Black/Brown Granular Pigmentation:  Seen within the connective tissue stroma, often concentrated along collagen bundles or around blood vessels.  This pigmentation is consistent with deposited amalgam particles (metallic silver or mercury from dental restorations).  Absence of Inflammatory Reaction or Melanocytic Proliferation:  The surrounding tissue shows no significant inflammatory infiltrate.  There is no evidence of atypical melanocytes or melanocytic nests, helping exclude melanocytic lesions such as melanomas or nevi.  Subepithelial Location:  Pigment is localized in the lamina propria/submucosa, beneath an intact stratified squamous epithelium.  The overlying epithelium is unremarkable, which is common in amalgam tattoos.  Metallic Nature of the Deposits (based on morphology):  The pigmented material appears opaque, irregular, and refractile in H&E stains—characteristics of metallic deposits rather than melanin or hemosiderin.  These features, particularly the pattern and appearance of pigmentation in the absence of cellular atypia or inflammation, are pathognomonic for amalgam tattoo, a benign iatrogenic lesion due to implantation of dental amalgam particles into soft tissue. | Histopathological (definitive) diagnosis  Amalgam tattoo – focal exogenous metallic pigmentation of the oral mucosa  Key microscopic features that point to this diagnosis  Area What is seen on these H‑E sections Why it supports an amalgam tattoo  Surface epithelium Stratified squamous epithelium of normal thickness and architecture; no acanthosis, dysplasia, or junctional proliferation. Rules out a true melanocytic lesion (e.g., nevus, melanoma) whose changes would begin in the epithelium or epithelial–connective‑tissue interface.  Superficial lamina propria Numerous coarse, jet‑black to dark‑brown granular/filamentous particles scattered between collagen bundles and around small vessels and nerve sheaths; the deposits run in linear streaks that follow the connective‑tissue architecture. This “pepper‑and‑strings” pattern is classic for exogenous metallic particles (silver, mercury, tin) that leach from dental amalgam and are carried along connective‑tissue ­planes.  Cellular response Pigment largely extracellular; occasional macrophages contain fine black particles; no significant inflammatory or foreign‑body giant‑cell reaction. Amalgam fragments are usually inert and evoke little or no inflammation, unlike graphite tattoo (often shows a more pronounced macrophage response) or other foreign‑body reactions.  Depth and distribution Pigment extends only within the subepithelial connective tissue, tapering off in deeper tissues; no involvement of basal keratinocytes. Endogenous melanin disorders (oral melanotic macule, smoker’s melanosis) show increased melanin in basal cells and melanophages directly beneath them, not dispersed deeper as seen here.  Optical properties (noted by routine microscopy) Deposits are intensely opaque, refractile, and may be weakly birefringent/polarizable. Metallic amalgam particles exhibit these optical characteristics, distinguishing them from melanin (brown, non‑refractile) or hemosiderin (golden‑brown, Prussian‑blue‑positive).  Taken together, the completely normal overlying epithelium plus the linear, jet‑black extracellular particulate material dispersed along collagen bundles with minimal host reaction is diagnostic of an amalgam tattoo, the most common exogenous pigmented lesion encountered in the oral cavity. |
| 10.82 | Diagnosis: Tobacco Pouch Keratosis; Key Microscopic Features: Low-power examination reveals mild hyperkeratosis and acanthosis with linear deposits of amorphous eosinophilic material in the lamina propria above minor salivary glands; higher-power view highlights the amorphous eosinophilic deposits. | Based on the two hematoxylin and eosin (H&E) stained histological images you provided (at low and higher magnifications), the single most likely histopathological diagnosis is:  Ameloblastoma  Key Microscopic Features Supporting This Diagnosis:  Follicular Pattern (best appreciated in the lower magnification image):  The tumor is composed of multiple discrete islands (or follicles) of odontogenic epithelium within a fibrous stroma.  These epithelial islands resemble the enamel organ.  Peripheral Palisading of Columnar Cells:  At higher magnification, the periphery of the epithelial islands shows a single layer of tall columnar cells with nuclei polarized away from the basement membrane, resembling preameloblasts.  Central Stellate Reticulum-like Areas:  The centers of the epithelial islands consist of loosely arranged angular cells with a star-shaped appearance, mimicking the stellate reticulum of the developing enamel organ.  Lack of Significant Atypia or Mitotic Activity:  Despite being locally aggressive, ameloblastomas are benign tumors and typically show bland cytology.  These features are characteristic of conventional (solid/multicystic) ameloblastoma, most commonly found in the mandible and arising from remnants of the dental lamina or enamel organ. | Histopathological diagnosis (single best):  Irritation (traumatic) fibroma – focal fibrous hyperplasia  Key microscopic features in the two H‑E images that support this diagnosis  Level of magnification Distinguishing features How the feature fits irritation fibroma  Low‑power overview (first image) • A dome‑shaped, well‑delineated nodular mass sits beneath an intact oral stratified squamous epithelium.  • The epithelium is hyperparakeratinised with elongated, blunted “strap‑like” rete ridges that dip only slightly into the lesion.  • The underlying connective tissue is uniformly eosinophilic and collagen‑rich, forming broad, interlacing bundles with very few cells.  • Residual, slightly compressed minor salivary glands and ducts are visible deep to the fibrous mass, confirming a reactive rather than neoplastic process. ► Reactive mucosal overgrowths almost always retain an intact surface epithelium; mild hyperkeratosis reflects chronic trauma.  ► A dense, paucicellular collagen core is the hallmark of an irritation fibroma.  ► Lack of capsule and gradual blending with adjacent tissues is typical for a reactive hyperplasia rather than a true tumour.  Higher power (second image) • The collagen bundles are thick, glassy‑eosinophilic and run in whorled or haphazard directions (“scar‑like” stroma).  • Fibroblasts are sparse, spindle‑shaped and evenly scattered; mitoses are absent.  • Only a scant chronic inflammatory infiltrate is present.  • Salivary ductal / acinar elements at the periphery are compressed but otherwise unremarkable. ► A paucicellular, densely sclerotic stroma with few fibroblasts and negligible inflammation is classic for a long‑standing irritation fibroma.  ► Absence of atypia, mitotic activity, or infiltrative growth excludes fibrosarcoma and other true neoplasms.  ► Compression (rather than invasion) of adjacent minor gland tissue again favours a benign, reactive fibrous hyperplasia.  Synthesis  Taken together, the images show a nodular, collagen‑dense, paucicellular lesion with overlying mildly hyperkeratinised oral epithelium and no cytologic atypia or active inflammation. These features typify an irritation (traumatic) fibroma, the most common reactive soft‑tissue enlargement in the oral cavity. |
| 10.92 | Diagnosis: Actinic Keratosis; Key Microscopic Features: There is a pronounced accumulation of parakeratin on the epidermal surface, and at high magnification, the epidermal cells display hyperchromatic nuclei and pleomorphic morphology. | Histopathological Diagnosis:  Squamous Cell Carcinoma (SCC)  Key Microscopic Features Supporting the Diagnosis:  From the low-magnification image (first image):  Epithelial dysplasia with invasion: There is a clear loss of the normal epithelial stratification and continuity. The epithelium invades into the underlying connective tissue in an irregular, finger-like pattern.  Keratin pearls: Swirls of keratin seen deep within the epithelium or connective tissue — a hallmark of well-differentiated SCC.  Irregular rete ridges: The downward proliferation of rete pegs is disordered and bulbous, often extending beyond the basal membrane.  From the high-magnification image (second image):  Cellular pleomorphism: Marked variation in nuclear size and shape.  Hyperchromatism: Deeply stained nuclei suggesting increased DNA content.  Abnormal mitotic figures: Presence of mitoses, some atypical — indicative of high proliferative activity.  Loss of normal maturation pattern: The normal orderly arrangement of keratinocytes from basal to superficial layers is lost.  Dyskeratosis: Premature keratinization of individual cells within the epithelial layer.  These features — especially invasion into the underlying connective tissue and the presence of keratin pearls — confirm a diagnosis of invasive squamous cell carcinoma, a common malignant epithelial neoplasm in the oral cavity. | Histopathological diagnosis (single best answer)  Oral squamous cell carcinoma in situ / severe epithelial dysplasia  Key microscopic features seen in the provided H‑E sections  Feature How it appears in the images Diagnostic significance  Full‑thickness loss of epithelial maturation From the basal layer up to the superficial keratin layer the cells look cytologically “immature,” and the normal orderly transition from basal → spinous → granular → keratin layers is virtually absent. Fulfilment of the criterion for severe dysplasia / carcinoma in situ (CIS).  Marked cytologic atypia Large, pleomorphic, hyper‑chromatic nuclei, irregular nuclear contours, coarse chromatin, occasional nucleoli, cellular and nuclear pleomorphism scattered throughout the epithelium (see high‑power image). Indicates a high‑grade intra‑epithelial lesion.  Suprabasal and abnormal mitotic figures Mitotic figures—not confined to the basal layer—are readily found several cell layers above it, some are atypical in form. Hallmark of high‑grade dysplasia / CIS.  Loss of basal‐cell polarity & bulbous “drop‑shaped” rete ridges The basal cells are no longer arranged in a single, palisaded row; the rete ridges are enlarged and irregular. Architectural dysplasia supporting CIS.  Surface hyper‑ (para/ortho)‑keratosis A thick corrugated keratin layer caps the lesion on low‑power view. Frequent accompaniment of dysplastic oral epithelium.  Intact basement membrane – no stromal invasion Although the rete ridges project downward, no islands or cords of atypical epithelial cells breach into the underlying lamina propria. Confirms the process is in situ rather than invasive squamous‑cell carcinoma.  The constellation of severe architectural disturbance plus marked cytologic atypia involving the entire epithelial thickness while the basement membrane remains intact is diagnostic of carcinoma in situ (severe epithelial dysplasia) of the oral mucosa. |
| 10.120 | Diagnosis: Squamous Cell Carcinoma; Key Microscopic Features: Tumor cells have invaded along a nerve fiber, breaching the perineurium, and there is also angioinvasion as malignant cells occupy the lumen of a blood vessel. | Based on the provided hematoxylin and eosin (H&E) stained images, here is the analysis:  Single Most Likely Histopathological Diagnosis:  Perineural Invasion by Oral Squamous Cell Carcinoma (OSCC)  Key Microscopic Features Supporting the Diagnosis:  Low-Magnification Image (First Image):  Invasive Nests and Islands of Malignant Squamous Cells:  Irregularly shaped epithelial nests infiltrating the underlying stroma.  Individual cells exhibit nuclear pleomorphism, hyperchromasia, and keratinization.  Formation of keratin pearls within tumor nests is suggestive of squamous differentiation.  Desmoplastic Stromal Reaction:  Dense fibrotic stroma surrounding the tumor islands is a typical feature of invasive squamous cell carcinoma.  High-Magnification Image (Second Image):  Perineural Invasion:  A clearly visible nerve (central round structure) surrounded or infiltrated by malignant squamous cells.  The tumor cells are encircling and sometimes invading the nerve bundle, a hallmark of perineural invasion.  This is an important prognostic feature in oral squamous cell carcinoma, associated with more aggressive behavior and increased risk of recurrence.  Nuclear Atypia and Increased Mitotic Activity:  Malignant cells show pleomorphic nuclei, some with prominent nucleoli.  High nuclear-to-cytoplasmic ratio and abnormal mitotic figures are visible.  Conclusion:  The combination of keratinizing squamous epithelial nests, stromal invasion, and perineural involvement strongly indicates oral squamous cell carcinoma with perineural invasion, an aggressive subtype of OSCC. | Histopathological diagnosis (single best):  Invasive (well‑differentiated) oral squamous cell carcinoma  Microscopic features visible in the two fields that support this diagnosis  Field Feature Diagnostic significance  Low‑power image • Irregular nests, islands and cords of epithelium infiltrating a fibrous (desmoplastic) connective‑tissue stroma.  • Central, concentrically laminated keratin pearls within several tumour islands.  • Individual‑cell keratinisation and intercellular bridges in peripheral tumour cells. Confirms a keratinising squamous epithelial malignancy and demonstrates frank stromal invasion.  High‑power image • Cohesive cluster of atypical squamous cells with pleomorphic, hyperchromatic nuclei and abundant eosinophilic cytoplasm occupying a clear vascular/perineural space (tumour embolus).  • Mitotic figures, some abnormal. Shows marked cytologic atypia characteristic of carcinoma and evidences lymphovascular / perineural invasion, a hallmark of invasive disease.  Key take‑home points from both sections  Keratin‑producing epithelial tumour islands → points specifically to a squamous phenotype rather than salivary or mesenchymal origin.  Infiltrative growth beyond the basement membrane with desmoplastic host response → separates carcinoma from epithelial dysplasia/carcinoma in situ.  Tumour embolus in a vascular or neural channel → further proof of invasiveness and malignant potential.  Taken together, these findings are diagnostic of an invasive, well‑differentiated squamous cell carcinoma arising in the oral–maxillofacial region. |
| 10.121 | Diagnosis: Well-differentiated Squamous Cell Carcinoma; Key Microscopic Features: At low magnification, nests of malignant squamous epithelium invade into the lamina propria; at high magnification, dysplastic squamous cells are forming keratin pearls. | Based on the provided hematoxylin and eosin (H&E) stained histopathological images taken at low and high magnification, the single most likely diagnosis is:  Keratoacanthoma  Key Microscopic Features Justifying the Diagnosis:  1. Low Magnification (First Image):  Exophytic lesion with central keratin-filled crater: The architecture suggests a dome-shaped lesion, a hallmark of keratoacanthoma.  Symmetric, circumscribed borders: The lesion appears well-demarcated and not deeply infiltrative, which helps distinguish it from more aggressive malignancies like squamous cell carcinoma.  Lobulated epithelial proliferation: The lesion consists of lobules of squamous epithelium extending into the dermis.  2. High Magnification (Second Image):  Well-differentiated squamous epithelium: Cells exhibit minimal atypia with eosinophilic cytoplasm.  Keratin pearl formation: Seen centrally within lobules, indicating squamous differentiation.  Mild nuclear pleomorphism and mitotic figures: But not markedly atypical or frequent—suggestive of a benign or low-grade nature.  Inflammatory stroma: The surrounding connective tissue shows a mixed inflammatory infiltrate, a common finding in keratoacanthoma.  Differential Diagnosis Considered but Less Likely:  Squamous Cell Carcinoma (SCC): Ruled out due to lack of deep invasion, significant cytologic atypia, and aggressive features.  Pseudoepitheliomatous Hyperplasia: Although reactive, it typically lacks keratin pearls and the overall lobular architecture seen here. | Histopathological diagnosis  → Well‑differentiated (keratinising) invasive squamous cell carcinoma of the oral mucosa  Microscopic features that support this diagnosis  Field What is seen in the plates Diagnostic value  Architecture (low‑power image) • Multiple, irregular nests, cords and sheets of epithelial cells have breached the mucosal basement membrane and infiltrate the underlying connective tissue.  • The infiltrating islands contain numerous round, eosinophilic “whorls” scattered throughout the lesion. Invasion beyond the epithelium confirms malignancy; the whorls hint at keratin‑pearl formation typical of keratinising SCC.  Cytology (high‑power image) • Tumour cells are polygonal with dense eosinophilic cytoplasm.  • Nuclei are enlarged, pleomorphic and hyperchromatic; occasional atypical mitotic figures are present.  • Intercellular bridges can be appreciated in places. Nuclear atypia, abnormal mitoses and intercellular bridges indicate malignant squamous differentiation.  Keratin production • Concentric lamellated keratin pearls are clearly visible within tumour nests (centre of the high‑power field).  • Foci of individual‑cell keratinisation are also present. Abundant keratin pearl formation is the hallmark of a well‑differentiated keratinising SCC.  Stromal response • Desmoplastic fibrous stroma with mixed inflammatory infiltrate surrounds invading nests.  • Focal vascular congestion and haemorrhage are present. Common stromal reaction to infiltrating carcinoma.  Together, the invasive growth pattern, pronounced squamous cytologic atypia, and prolific keratin‑pearl production leave little doubt that the lesion represents a well‑differentiated (keratinising) squamous cell carcinoma rather than a benign keratinising process or a less‑differentiated epithelial malignancy. |
| 10.126 | Diagnosis: Verrucous Carcinoma; Key Microscopic Features: Low-power view shows marked epithelial hyperplasia with a rough, papillary surface and keratin plugging, while high-power view reveals broad, bulbous rete ridges without significant dysplasia. | Based on the analysis of the provided hematoxylin-and-eosin stained histological sections of an oral lesion (low and high magnification), the single most likely histopathological diagnosis is:  Papilloma (Squamous Papilloma)  Key Microscopic Features Justifying the Diagnosis:  Exophytic Growth Pattern:  The lesion exhibits a papillary, exophytic (outward-growing) architecture, which is a hallmark of papillomatous lesions.  Finger-like Projections:  Numerous papillary projections lined by stratified squamous epithelium are evident, particularly in the low magnification image.  These projections contain a central fibrovascular core — a classic feature of squamous papilloma.  Hyperkeratosis and Acanthosis:  The high magnification image shows thickening of the epithelium (acanthosis) and surface keratin buildup (hyperkeratosis), common in benign squamous proliferations.  Koilocytosis May Be Present (not clearly visible here):  Although not distinctly seen in the images, squamous papillomas often show koilocytosis (indicative of HPV infection), which would further support this diagnosis if present.  Lack of Dysplasia or Invasion:  The basal layer appears intact without signs of cellular atypia or invasion into the underlying connective tissue, supporting a benign diagnosis. | Histopathological diagnosis – Verrucous carcinoma (Ackerman tumour)  Microscopic hallmarks seen in the two H‑E fields  Feature What you can see in the photographs Why it points to verrucous carcinoma  Exo‑endophytic, verruciform architecture Low‑power slide shows a wavy, “church‑roof” surface with thick papillary folds and clefts full of keratin. At the same time the epithelium pushes deeply downward. Classical growth pattern of a verrucous carcinoma: a broad, wart‑like surface and bulbous downward extension.  Broad, bulbous, pushing rete pegs At high power the rete ridges are uniformly wide, rounded at their tips and descend farther than the neighbouring normal mucosa, but the epithelial–connective‑tissue interface remains smooth. Verrucous carcinoma grows endophytically by expansion rather than by sharp, infiltrative cords seen in conventional SCC.  Heavy hyper‑/parakeratosis with keratin plugs Surface and crypts are packed with thick eosinophilic keratin; plugs fill the invaginations between papillae. The exuberant keratin production that fills surface clefts is typical and gives the lesion its pale, verrucous clinical appearance.  Minimal cytological atypia Epithelium is thick but the cells mature normally from basal to superficial layers; nuclei are uniform, mitoses are rare and confined to the basal/parabasal layers. Verrucous carcinoma is well‑differentiated—it looks deceptively “benign” cytologically despite its malignant behaviour.  Intact basement membrane / pushing border No jagged tongues or single‑cell infiltration; connective tissue beneath the pegs shows only a mild chronic inflammatory infiltrate. Confirms the tumour is the verrucous variant rather than a conventional invasive SCC.  Taken together—an exophytic–endophytic verrucous surface, broad pushing rete pegs deeper than adjacent mucosa, abundant keratin with plugs, but little or no dysplasia—the lesion is diagnostic of verrucous carcinoma. |
| 10.139 | Diagnosis: Basal Cell Carcinoma; Key Microscopic Features: Low-power examination demonstrates ulceration of the epidermal surface overlying an invasive tumor of hyperchromatic epithelial cells, and higher-power view shows islands of basophilic epithelial cells exhibiting peripheral palisading. | Histopathological Diagnosis:  Mucoepidermoid Carcinoma (MEC) – a malignant salivary gland tumor.  Justification with Key Microscopic Features:  From the low-power image (Image 1):  Lobular architecture: The tumor appears lobulated and infiltrative, extending into the underlying connective tissue.  Cystic and solid areas: Suggestive of a neoplasm with variable cellular patterns.  Ulcerated surface epithelium: Often seen in aggressive lesions involving the mucosa.  From the high-power image (Image 2):  Mixed cellular components:  Mucous cells: Large cells with foamy cytoplasm (mucin production).  Epidermoid (squamoid) cells: Polygonal cells with eosinophilic cytoplasm and intercellular bridges.  Intermediate cells: Small to medium-sized cells with less distinct cytoplasm—common in MEC and help in grading.  Cystic and glandular differentiation: Some gland-like structures are evident.  Mitoses and nuclear atypia: Indicating malignant potential.  These features together point toward mucoepidermoid carcinoma, a common salivary gland malignancy of varying grades based on the proportion of mucous, intermediate, and epidermoid cells. | Histopathological diagnosis  → Adenoid cystic carcinoma ( “cylindroma” ) of a minor salivary gland  Key microscopic features visible in the two H‑E sections  Low‑power view High‑power view How the feature supports the diagnosis  • Ulcerated surface oral epithelium with an underlying, sharply infiltrative tumor extending deep into the connective tissue — Confirms a malignant salivary‑type lesion rather than an in‑situ surface process  • Numerous rounded and oval epithelial islands and cords separated by a fibrous stroma, giving a “Swiss‑cheese / cribriform” architecture • Islands show multiple punched‑out pseudocystic spaces filled with lightly eosinophilic to basophilic hyaline/mucinous material Classic cribriform pattern of adenoid cystic carcinoma  • Intermixed tubular structures: small true ducts with an inner cuboidal/columnar layer and an outer flattened/basal/myoepithelial layer • Dual‑cell population is evident: inner duct‑type cells with slightly more cytoplasm, and outer small basaloid cells with hyperchromatic angulated nuclei and scant cytoplasm Tubular‑cribriform combination is the commonest histologic pattern of ACC  • Irregular infiltration between normal salivary acini and along fibrous septa — Infiltrative growth is typical of ACC and helps distinguish it from benign cribriform adenomas  — • Occasional small necrotic plugs and mitotic figures but no keratin pearls or intercellular bridges Rules out squamous‑cell carcinoma; cytology matches adenoid cystic carcinoma  (The hallmark triad—basaloid cells, cribriform/tubular patterns, and hyaline pseudocysts—seen in these images is diagnostic of adenoid cystic carcinoma.) |
| 10.149 | Diagnosis: Nodular Melanoma; Key Microscopic Features: At low magnification, a nodular mass of malignant melanocytes infiltrates the dermis without radial growth in the adjacent overlying epidermis; at high magnification, atypical spindle-shaped melanocytes are visible. | Based on the provided hematoxylin and eosin (H&E) stained histopathological images at low and high magnification, the most likely diagnosis is:  Irritational Fibroma (Focal Fibrous Hyperplasia)  Key Microscopic Features Supporting the Diagnosis:  Well-circumscribed, dome-shaped lesion (visible in the low-power image):  The lesion is confined and separated from the surrounding tissue, consistent with a benign, reactive growth.  Dense, collagenous stroma:  The lesion is composed of interlacing bundles of dense collagen fibers, characteristic of fibrous hyperplasia.  Spindle-shaped fibroblasts:  Scattered throughout the stroma are bland, elongated fibroblasts, with no cytologic atypia or abnormal mitoses, supporting a benign/reactive nature.  Overlying stratified squamous epithelium:  Appears hyperplastic and hyperkeratotic in some areas, reflecting chronic irritation. There’s no evidence of dysplasia or malignancy.  Lack of encapsulation yet demarcated from surrounding tissues:  Although not encapsulated, the lesion’s clear demarcation is typical of a reactive fibrous lesion rather than an infiltrative neoplasm.  Summary:  These features are consistent with an irritational fibroma, a common benign soft tissue lesion in the oral cavity, typically arising in response to chronic irritation or trauma. | Histopathological diagnosis  Traumatic (irritation) fibroma – also called focal fibrous hyper‑plasia.  Key microscopic features that support this diagnosis  Level of magnification Observations Significance  Scanning / low power (first image) • A solitary, dome‑shaped, well‑circumscribed nodule lying immediately beneath an intact stratified‑squamous surface epithelium.  • Broad “pushing” (non‑infiltrative) margin and a uniformly pink (eosinophilic) stroma that contrasts with the darker epithelium.  • No lobulation, cystic spaces or epithelial tumor islands. Indicates a benign, reactive fibrous mass rather than an epithelial neoplasm or an infiltrative soft‑tissue tumour.  Intermediate / high power (second image) • Stroma composed of dense, thick, interlacing bundles of mature collagen with scant vascularity.  • Sparse, evenly dispersed spindle‑shaped fibroblasts; nuclei are bland, elongated and without pleomorphism or increased mitotic figures.  • Overlying epithelium shows mild hyper‑para/orthokeratosis and acanthosis—changes typical of chronic surface irritation.  • Absence of myxoid change, calcifications, duct‑like structures, Verocay bodies, atypia or inflammatory granulation tissue. Classic picture of a collagenised “scar‑like” connective‑tissue proliferation produced by chronic mechanical irritation. The lack of atypia, the paucicellular collagen bundles and the surface hyperkeratosis together distinguish it from neoplastic spindle‑cell lesions, salivary or odontogenic tumours, and from reactive inflammatory polyps.  Taken together, the circumscribed, hypocellular, collagen‑rich nodule covered by slightly hyperplastic mucosa is diagnostic of a traumatic/irritation fibroma of the oral mucosa. |

Salivary Gland Pathology

| **Fig_no** | **Ground truth** | **GPT 4o answer** | **o3 answer** |
| --- | --- | --- | --- |
| 11.11 | Diagnosis: Salivary Duct Cyst; Key Microscopic Features: On low magnification, a cyst is seen beneath the mucosal surface, and on higher magnification, the cystic chamber is lined by a thin cuboidal epithelial layer with an adjacent excretory salivary gland duct bordered by columnar epithelium. | Based on the provided hematoxylin-and-eosin (H&E) stained histopathology images of a lesion from the salivary gland region, here is the analysis:  Histopathological Diagnosis:  Mucocoele (Mucous Retention Cyst or Extravasation Cyst)  Key Microscopic Features Supporting the Diagnosis:  Cystic Cavity:  Both images show a well-defined cystic cavity, often lined by granulation tissue or compressed connective tissue. There is no true epithelial lining, which is typical of a mucous extravasation phenomenon (rather than a retention cyst).  Mucin Pool:  The lumen appears to contain pale, eosinophilic material consistent with extravasated mucin, a hallmark of mucocoeles.  Inflammatory Response:  Surrounding the mucin pool is a granulation tissue response with numerous macrophages, many of which may appear foamy due to phagocytosed mucin.  Overlying Minor Salivary Gland Tissue:  The periphery may show remnants of minor salivary gland lobules, although not prominent in these particular fields.  Absence of True Epithelial Lining:  The absence of a lining epithelium distinguishes it from a mucous retention cyst and suggests an extravasation type of mucocoele, the most common variant in minor salivary glands.  These histologic findings are characteristic of a mucocoele, commonly arising in the lower lip due to trauma or blockage of minor salivary gland ducts. | Histopathological diagnosis  Mucocele – mucous‑extravasation type (extravasation phenomenon of a minor salivary gland)  Key microscopic features that support this diagnosis  Feature seen in the photomicrographs Significance  A single, irregular, unilocular cyst‑like space occupying the submucosa Represents the pool of extravasated mucin that has collected after rupture of an excretory duct.  The cavity is not lined by an epithelium – its “wall” is formed only by compressed granulation / fibrous tissue Absence of a true epithelial lining distinguishes an extravasation mucocele from a mucous‑retention (duct) cyst or other true cystic neoplasms.  Granulation‑tissue wall populated by numerous foamy macrophages (muciphages) and scattered chronic inflammatory cells Macrophages phagocytose spilled mucin; the associated mild chronic inflammatory infiltrate is typical for an extravasation phenomenon.  Overlying oral mucosa consisting of stratified squamous epithelium remains intact and arches over the lesion Indicates the lesion is submucosal and of salivary origin rather than arising from the surface epithelium.  Adjacent minor salivary‑gland lobules and dilated or ruptured ducts may be seen at the periphery Provides the anatomic source of the leaked mucin, corroborating the diagnosis.  The constellation of (1) a mucin‑filled cavity, (2) lack of an epithelial lining, and (3) a wall composed of granulation tissue laden with muciphages is diagnostic of a mucocele (mucous‑extravasation phenomenon). |
| 11.55 | Diagnosis: Sialadenoma Papilliferum; Key Microscopic Features: At low power, a papillary surface neoplasm is evident with associated ductal structures in the superficial lamina propria, and at high power, cystic areas are lined by papillary oncocytic epithelium. | Based on the provided hematoxylin-and-eosin stained histopathological images (low and high magnification), the most likely diagnosis is:  Pleomorphic Adenoma (Benign Mixed Tumor)  Justification and Key Microscopic Features:  Architectural Pattern:  The low magnification image shows a well-circumscribed, lobulated lesion with papillary and glandular architecture protruding into cystic spaces. This lobulated growth pattern is typical of pleomorphic adenoma.  The lesion shows mixed epithelial and mesenchymal (stromal) elements, a hallmark feature of pleomorphic adenoma.  Epithelial Components:  The high magnification image demonstrates ductal and myoepithelial cells forming tubular and papillary structures.  Ducts are lined by cuboidal to columnar epithelial cells, which may show some degree of stratification but lack significant atypia or mitotic activity.  Mesenchymal (Stromal) Elements:  There is an abundant chondromyxoid stroma (though not very prominent in this particular field, often seen in other areas of pleomorphic adenoma).  The stroma may appear mucoid, myxoid, or cartilaginous, reflecting the “pleomorphic” nature.  Cellularity and Cytology:  Bland cytology with uniform nuclei and absence of mitotic figures or necrosis, supporting a benign process.  Summary:  Pleomorphic adenoma is the most common benign salivary gland tumor and typically presents with a combination of epithelial and myxochondroid stromal components. The images show classical features of this tumor type, with a lobulated architecture, ductal structures, and benign cytologic appearance. | Histopathological diagnosis (single best answer)  Papillary cystadenoma (salivary‑gland type) – a benign papillary–cystic epithelial neoplasm of minor or major salivary glands.  Key microscopic features visible in the two H‑E sections  Field Observations that point to papillary cystadenoma  Overall architecture (low‑power view) • Well‑circumscribed lesion composed of multiple cystic cavities that communicate with one another.  • Each cavity is filled by numerous, slender papillary fronds projecting into the lumen, producing a villous or “sea‑anemone” outline.  • Papillae are supported by delicate fibro‑vascular cores, not by lymphoid tissue (helps exclude Warthin tumour).  Epithelial lining (high‑power view) • Papillae are covered by a single to bilayered cuboidal‑to‑low‑columnar ductal epithelium with finely granular, lightly eosinophilic cytoplasm.  • Nuclei are uniform, round‑to‑oval, basally placed; mitoses or cytologic atypia are absent → indicates a benign process.  Stroma / supporting tissue • Cores show fibrovascular connective tissue without germinal centres or dense lymphoid tissue (again arguing against Warthin tumour).  • No myxoid, chondroid or osseous stroma (would be typical for pleomorphic adenoma).  Lack of malignant features • No infiltrative borders, perineural invasion, cribriform growth or comedo‑type necrosis that would suggest salivary duct carcinoma or intraductal carcinoma.  • Overall bland cytology and orderly architecture confirm a benign cystadenomatous lesion.  These combined features—multiloculated cystic architecture with profuse, delicate fibrovascular papillae that are uniformly lined by bland ductal epithelium—are diagnostic of papillary cystadenoma of salivary origin. |
| 11.56 | Diagnosis: Inverted Ductal Papilloma; Key Microscopic Features: A papillary intraductal proliferation is located beneath the mucosal surface, and at higher magnification, both squamous cells and mucous cells are present. | Histopathological Diagnosis:  Pleomorphic Adenoma (Benign Mixed Tumor)  Key Microscopic Features Supporting the Diagnosis:  Ductal and Myoepithelial Components (Biphasic Pattern):  The second, higher-magnification image clearly shows duct-like structures lined by epithelial cells and surrounded by plasmacytoid or myoepithelial cells, a hallmark of pleomorphic adenoma.  The cellular arrangement in islands, cords, and trabeculae is characteristic.  Chondromyxoid Stroma:  In the low-power image (first image), there are regions of loose, myxoid to chondroid stroma, which appear pale and are interspersed between epithelial components.  This mesenchymal-like stroma, despite being derived from myoepithelial cells, is a diagnostic clue for pleomorphic adenoma.  Well-Circumscribed, Lobulated Architecture:  The lesion is well-demarcated and exhibits a lobulated configuration, as seen in the low-power view. This feature supports its benign nature.  Absence of Atypia or Invasion:  There is no cytologic atypia, no mitotic figures, and no evidence of invasive growth, favoring a benign process.  Summary:  These findings — biphasic epithelial and myoepithelial cell population, presence of chondromyxoid stroma, and well-circumscribed lobulated growth pattern — are diagnostic of pleomorphic adenoma, the most common benign salivary gland neoplasm. | Histopathological diagnosis  → Warthin tumour (papillary cystadenoma lymphomatosum)  Microscopic features in the two H‑E sections that point to this diagnosis  Low‑power survey image High‑power view Diagnostic significance  • Sharply circumscribed, partly encapsulated nodule with one dominant cystic space and innumerable delicate papillary infoldings that almost fill the lumen • Each papillary frond is built around a thin fibro‑vascular/lymphoid core A cystic tumour that is essentially papillary in architecture is the hallmark starting point for Warthin tumour  • Cores of many papillae are densely basophilic, representing organised lymphoid tissue • Reactive‑looking lymphoid aggregates with germinal centres are easily recognised immediately beneath the epithelium A lymphoid stroma that is integral to every papilla (“lymphomatosum”) is unique among salivary tumours  • The epithelial lining is very orderly: the luminal surface is smooth, with no exophytic squamous projections • Epithelium is bilayered: ⬩ Inner (luminal) row of tall columnar oncocytic cells packed with finely granular, intensely eosinophilic cytoplasm ⬩ Outer row of smaller cuboidal/basaloid cells hugging the basement membrane The constant two‑cell‑thick oncocytic lining is pathognomonic; other papillary–cystic lesions either have a single layer or a thick, irregular multilayered epithelium  • No chondromyxoid, hyalinised or myxoid stroma; no cribriform or solid invasive foci • Cells are bland, mitoses are rare/absent Excludes pleomorphic adenoma, basal‑cell adenoma, intraductal carcinoma, etc.; confirms the lesion is benign  Putting the features together  Cystic tumour almost completely filled by branching papillae.  Every papilla contains prominent lymphoid tissue.  The lining epithelium is strictly bilayered and oncocytic.  This triad is diagnostic of Warthin tumour and explains why the entity was historically called papillary cystadenoma lymphomatosum. |
| 11.73 | Diagnosis: Carcinoma ex Pleomorphic Adenoma; Key Microscopic Features: On medium power, the benign portion of the tumor shows sheets of plasmacytoid myoepithelial cells within a myxoid background, whereas the malignant component demonstrates epithelial cells with pleomorphic nuclei. | Histopathological Diagnosis:  Mucoepidermoid Carcinoma (MEC) of the salivary gland.  Key Microscopic Features Supporting the Diagnosis:  Image A (Low to Intermediate Magnification):  Mucin-producing cells (pale-staining cytoplasm) interspersed throughout the tumor—these are indicative of mucous cells.  Intermediate cells with a polygonal shape and more eosinophilic cytoplasm.  Cystic and solid areas: The tumor shows architectural heterogeneity, a hallmark of MEC.  Some epidermoid (squamoid) cells are present with more eosinophilic, dense cytoplasm.  Image B (Higher Magnification):  Mucous cells with foamy, vacuolated cytoplasm are more clearly discernible.  Intermediate cells predominate and show variable nuclear size and shape (pleomorphism), consistent with a low- to intermediate-grade tumor.  Duct-like structures and solid nests of cells within a fibrous stroma.  Lack of keratinization, which helps differentiate from squamous cell carcinoma.  Conclusion:  These histological findings of mucous, intermediate, and squamoid (epidermoid) cells in a mixed cystic and solid pattern are diagnostic of Mucoepidermoid Carcinoma, the most common malignant salivary gland tumor. | Based on the provided hematoxylin-and-eosin (H&E) images of the same lesion in a salivary gland, here is the histopathological analysis:  Single Most Likely Diagnosis:  The histopathological features are suggestive of a pleomorphic adenoma, which is a common benign mixed tumor of the salivary glands.  Key Microscopic Features Seen in the Images:  Cellular Composition:  The images show a mixture of epithelial cells and myoepithelial cells, which are characteristic of pleomorphic adenomas. The glandular structures are mixed with stroma, showing a variety of cell types.  Architecture:  The tumor exhibits a biphasic architecture, with both epithelial cells (arranged in ducts or glandular patterns) and myoepithelial cells (scattered around the glands).  Stroma:  The stroma appears myxoid and chondroid, which is another hallmark of pleomorphic adenomas. The stroma may appear variable, with areas resembling cartilage or mucous tissue.  Cellular Pleomorphism:  There is notable cellular pleomorphism with varying shapes and sizes of the cells, but not as severe as in malignant lesions, confirming the benign nature of the tumor.  Infiltration and Encapsulation:  The lesion is typically well-circumscribed with minimal invasion into surrounding tissues, which is indicative of the benign nature of the pleomorphic adenoma.  Conclusion:  The lesion shows features typical of a pleomorphic adenoma, including a biphasic pattern of epithelial and myoepithelial cells, mixed stroma, and cellular pleomorphism. This confirms the benign nature of the lesion, and the well-formed encapsulation seen in the images supports the diagnosis. |
| 11.77 | Diagnosis: Adenoid Cystic Carcinoma; Key Microscopic Features: Islands of hyperchromatic cells form cribriform and tubular structures, and on high power, a small cribriform island is apparent. | Histopathological Diagnosis:  Mucoepidermoid Carcinoma (MEC) of the salivary gland  Justifying Key Microscopic Features:  The diagnosis of Mucoepidermoid Carcinoma is supported by the following histological features observed in the two H&E-stained images:  1. Mixed Cell Population:  There is a heterogeneous mix of mucous, epidermoid, and intermediate cells, which is a hallmark of MEC.  The mucous cells are large and pale with foamy cytoplasm (seen in clusters and lining cystic spaces).  Epidermoid cells have more eosinophilic cytoplasm and prominent cell borders.  2. Cystic and Solid Architecture:  Multiple cyst-like spaces of varying sizes are evident, some containing mucin (pale bluish content).  Solid nests and cords of tumor cells are interspersed between these cystic spaces.  This pattern suggests low- to intermediate-grade MEC.  3. Mucin Production:  Presence of mucin-filled vacuoles in mucous cells is clearly visible.  Mucin production is a key diagnostic clue—mucicarmine or PAS stains would confirm this further.  4. Mild Nuclear Atypia:  At higher magnification, minimal pleomorphism and low mitotic activity can be appreciated, supporting a lower grade tumor.  These features collectively point toward Mucoepidermoid Carcinoma, the most common malignant salivary gland tumor, especially affecting the parotid gland, but also seen in minor salivary glands. | The provided hematoxylin-and-eosin (H&E) images depict a lesion within the salivary gland, showcasing some typical histopathological features of a specific benign salivary gland tumor.  Single Most Likely Histopathological Diagnosis:  Pleomorphic Adenoma (Benign Mixed Tumor)  Key Microscopic Features Seen in the Images:  Cellular Composition:  The first image shows a mixture of ductal structures and stromal components. There are areas of epithelial cells forming duct-like structures, alongside a more cellular stromal component. The stroma can be myxoid or hyalinized, which is characteristic of pleomorphic adenomas.  Epithelial and Stromal Features:  In the second image, the lesion shows a more distinct appearance with cells arranged in cystic formations. The epithelial components are seen forming both ductal structures and myoepithelial cell layers. The surrounding stroma consists of a combination of fibrous and myxoid tissue.  Cystic Spaces:  The presence of numerous cystic spaces filled with eosinophilic material is a common feature of pleomorphic adenomas, contributing to their characteristic appearance.  Encapsulation:  Although not fully shown in the provided images, pleomorphic adenomas are usually well-circumscribed and often have a capsule that distinguishes them from other malignant salivary gland tumors.  These histopathological features align with the diagnosis of pleomorphic adenoma, which is the most common benign tumor of the salivary glands. The presence of mixed epithelial and stromal components with cystic spaces supports this diagnosis. |

Soft Tissue Tumors

| **Fig_no** | **Ground truth** | **GPT 4o answer** | **o3 answer** |
| --- | --- | --- | --- |
| 12.11 | Diagnosis: Giant Cell Fibroma; Key Microscopic Features: At low magnification, there is a nodular proliferation of fibrous connective tissue topped by stratified squamous epithelium with elongated rete ridges, and at higher magnification, numerous large, star-shaped, multinucleated fibroblasts are observed. | Based on the provided hematoxylin and eosin (H&E) stained histopathology images of a soft tissue tumor in the oral and maxillofacial region, captured at low and high magnification:  Single Most Likely Histopathological Diagnosis:  Pleomorphic Adenoma (Benign Mixed Tumor)  Key Microscopic Features Supporting the Diagnosis:  Low-Power Image:  Lobulated growth pattern with well-circumscribed borders.  The tumor shows extensive epithelial and myxoid stromal components, with multiple glandular or duct-like structures scattered throughout.  The architecture resembles a mix of epithelial and mesenchymal elements, which is characteristic of pleomorphic adenoma.  High-Power Image:  Presence of plasmacytoid or myoepithelial cells within a myxochondroid stroma.  Cells with eccentric nuclei and abundant eosinophilic cytoplasm, embedded in a background of loose, myxoid, or chondroid matrix.  Scattered duct-like structures lined by cuboidal to columnar epithelial cells.  These features are diagnostic of pleomorphic adenoma, the most common benign salivary gland tumor, typically arising in the parotid gland or minor salivary glands, including the palate in the oral cavity. | The two images you've provided appear to show histopathological sections of a lesion, likely from the Soft Tissue Tumors category in oral and maxillofacial pathology. Based on the features presented in these hematoxylin-and-eosin (H&E) stained slides, here is the analysis:  Single Most Likely Histopathological Diagnosis:  Fibromatosis (Desmoid Tumor)  Key Microscopic Features:  First Image (Low Magnification):  Pushing Borders: The lesion exhibits a well-circumscribed mass with infiltrative, irregular borders. This suggests a tumor that grows expansively, but without a significant capsule.  Fibrous Stroma: The lesion is composed of dense collagenous tissue, which is a key feature of fibromatosis.  Strands of Fibroblasts: There are strands or bundles of fibroblasts dispersed throughout the stroma. The pattern of fibrosis with fibroblastic infiltration is a hallmark of this tumor type.  Second Image (High Magnification):  Dense Collagen and Fibroblastic Proliferation: The high magnification reveals more detailed fibroblastic activity with elongated, spindle-shaped cells interspersed within a dense collagen matrix.  No Significant Cytological Atypia: The fibroblasts appear relatively uniform, with no pronounced atypical features, which supports the diagnosis of a benign fibroblastic lesion rather than a malignant one.  Mild to Moderate Cellularity: The lesion shows a moderate number of fibroblasts, which are not highly pleomorphic or mitotically active, consistent with the behavior of fibromatosis.  Conclusion:  The presence of a dense, fibrous stroma with a pattern of fibroblastic proliferation and infiltrative growth, without atypia or mitotic activity, points strongly towards fibromatosis (desmoid tumor). This type of tumor can occur in the head and neck region and is characterized by its locally aggressive, but typically benign, nature. |
| 12.25 | Diagnosis: Solitary Fibrous Tumor; Key Microscopic Features: At low magnification, “staghorn” vascular channels are surrounded by randomly distributed cells, while at higher magnification, a moderately cellular fibrous proliferation with a patternless architecture shows conspicuous blood vessels, focal myxoid change, and scattered thick collagen bundles. | Based on the two provided hematoxylin-and-eosin (H&E) stained images of the same lesion, which falls under the category of soft tissue tumors in oral and maxillofacial pathology:  Most Likely Histopathological Diagnosis:  Myxofibrosarcoma  Key Microscopic Features Supporting This Diagnosis:  Myxoid Stroma:  Both images show extensive pale myxoid areas, particularly in the first image, suggesting a mucinous extracellular matrix—characteristic of myxofibrosarcoma.  Curvilinear Blood Vessels:  The first image shows prominent, curvilinear, thin-walled blood vessels, a hallmark feature of myxofibrosarcoma.  Spindle Cell Proliferation:  There is a proliferation of spindle-shaped cells, seen in both low- and higher-power images, displaying mild to moderate nuclear atypia.  Pseudolipoblasts and Pleomorphism:  While pseudolipoblasts may not be distinctly visible in these particular images, the second image shows moderate pleomorphism and cellularity, supporting a low- to intermediate-grade lesion.  Storiform Pattern:  The second image reveals areas of storiform or swirling pattern formed by the spindle cells, which is another feature sometimes seen in myxofibrosarcoma.  Summary:  The presence of a myxoid background, curvilinear vasculature, spindle cells with variable atypia, and storiform architecture aligns well with myxofibrosarcoma, a malignant soft tissue tumor with a predilection for older adults and known for its infiltrative growth. | Based on the provided hematoxylin-and-eosin (H&E) images of the same lesion, I will now provide a histopathological diagnosis and explain the key microscopic features observed in the images.  Please allow me a moment to analyze them.  I've reviewed the provided images and will now proceed with the histopathological analysis. Based on the visual inspection of the H&E-stained tissue sections, the lesion seems to be indicative of a fibrous tissue neoplasm, with possible characteristics of a Fibromatosis or Desmoid tumor, considering the patterns of cell arrangement and tissue features observed.  Key Microscopic Features Supporting This Diagnosis:  Fibrous Stroma: The predominant feature in both images is the presence of a dense fibrous stroma, with a mix of spindle-shaped cells. This suggests a fibrous nature of the lesion, commonly seen in fibromatosis.  Spindle-shaped Cells: There are numerous elongated, spindled cells within the stroma, which are characteristic of fibroblasts, supporting the fibrous nature of the tumor.  Infiltrative Growth Pattern: The lesion demonstrates an infiltrative pattern with cells extending into the surrounding tissues. This feature is often associated with desmoid tumors and fibromatosis.  Lack of Significant Atypia: There is no overt atypia or mitotic activity seen in these images, which would further suggest that the lesion is a benign fibrous lesion, likely a fibromatosis or a desmoid tumor.  Vascular Pattern: The presence of small, irregular blood vessels can also be noted in the stroma, which is typical in soft tissue tumors like fibromatosis.  Conclusion:  Based on the above microscopic features, the diagnosis most likely points toward Desmoid Tumor (also known as aggressive fibromatosis) or a Fibromatosis, both of which are benign, but locally invasive fibrous tumors. |
| 12.48 | Diagnosis: Peripheral Ossifying Fibroma; Key Microscopic Features: Low-power imaging reveals a nonulcerated fibrous gingival mass containing central ossification, and at higher magnification, bony trabeculae are seen adjacent to fibrous connective tissue. | Histopathological Diagnosis:  Peripheral Ossifying Fibroma (POF)  Key Microscopic Features Justifying the Diagnosis:  Fibrous Proliferation:  Both images show a dense fibrous connective tissue stroma, a hallmark of POF.  The stroma consists of spindle-shaped fibroblasts with varying cellularity and collagen content.  Presence of Mineralized Material:  The higher magnification image clearly shows multiple foci of mineralized tissue — including:  Trabeculae of woven bone  Cementoid-like material  Osteoid formation  These calcified components are embedded within the fibrous stroma and vary in shape and size.  Overlying Epithelium:  The low-power image demonstrates a surface stratified squamous epithelium, possibly ulcerated or hyperplastic, typical in reactive gingival lesions.  Well-Circumscribed but Not Encapsulated Lesion:  The lesion appears relatively well-demarcated from surrounding tissues, though not encapsulated — a feature often seen in peripheral ossifying fibromas.  Summary:  These histological findings — a cellular fibrous stroma with embedded mineralized material (bone and/or cementum-like deposits) beneath a stratified squamous epithelial surface — are characteristic of Peripheral Ossifying Fibroma, a common reactive gingival lesion of the oral cavity. | Upon reviewing the provided hematoxylin-and-eosin (H&E) stained images of the lesion in the Soft Tissue Tumors group of oral and maxillofacial pathology, the histopathological diagnosis is likely keratocystic odontogenic tumor (KCOT), also known as the odontogenic keratocyst.  Key microscopic features observed:  Epithelial Lining:  The lesion exhibits a thin, stratified squamous epithelium lining, which is characteristic of the KCOT.  The epithelium appears parakeratotic, with flattened basal cells and columnar cells in the superficial layers, which is consistent with the keratin-producing nature of KCOT.  Basal Layer Configuration:  The basal layer of the epithelium is prominent and exhibits palisading (stacked arrangement of cells), which is one of the hallmark features of KCOT.  Cystic Lumen:  The presence of a well-defined cystic lumen filled with keratinaceous material (suggested by the appearance of the cystic space in the images) is also typical of this tumor type.  Keratinized Surface:  The surface of the epithelial lining in the higher magnification shows evidence of parakeratinization (retention of nuclei in the superficial layer), a distinguishing feature of KCOT.  No Inflammatory Cells:  There is a lack of significant inflammatory infiltrates, which distinguishes it from other odontogenic cysts, such as the dentigerous cyst or radicular cyst, where inflammation may be more prominent.  These features collectively support the diagnosis of keratocystic odontogenic tumor (KCOT), which is a developmental cyst with aggressive growth potential and a tendency to recur if not managed appropriately. |
| 12.58 | Diagnosis: Palisaded Encapsulated Neuroma; Key Microscopic Features: At low magnification, there is a well-circumscribed, nodular neural proliferation, and at higher magnification, spindle-shaped cells with wavy nuclei are evident. | Histopathological Analysis of Provided H&E-Stained Images  Diagnosis:  Neurofibroma – a benign peripheral nerve sheath tumor.  Key Microscopic Features Supporting the Diagnosis:  Low Magnification (First Image):  Well-circumscribed but non-encapsulated lesion: The lesion is clearly demarcated from the surrounding connective tissue but lacks a true capsule, which is typical of neurofibroma.  Expansion within the dermis/submucosa: The lesion appears to push up the overlying epithelium, consistent with a dermal/submucosal origin.  Loose myxoid stroma: The overall pale appearance indicates a loose connective tissue matrix often seen in neurofibromas.  High Magnification (Second Image):  Spindle-shaped cells with wavy nuclei: These are characteristic of Schwann cells or fibroblast-like cells in neurofibroma.  Low mitotic activity and no atypia: Supports a benign nature.  Presence of collagen bundles interspersed with tumor cells: A common feature in neurofibromas.  Lack of a biphasic pattern: Unlike schwannomas, neurofibromas do not exhibit Antoni A and B areas.  These features are consistent with a neurofibroma, a benign tumor of neural origin that often presents as a solitary lesion in the head and neck region, particularly in the oral cavity. | The two images you've provided show histological sections stained with hematoxylin and eosin (H&E) that are relevant for soft tissue tumors in oral and maxillofacial pathology.  Histopathological Diagnosis:  The most likely diagnosis based on these images is Desmoid-type fibromatosis (also known as aggressive fibromatosis).  Key Microscopic Features:  First Image (Low Magnification):  The lesion appears well-circumscribed, with irregular borders and a fibrous appearance.  There is a dense fibrocollagenous stroma that envelops the lesion. The fibrous tissue shows a somewhat haphazard arrangement.  The lesion may exhibit thick collagen bundles, which are characteristic of fibromatosis.  Second Image (Higher Magnification):  At higher magnification, spindle-shaped fibroblasts are seen, which are characteristic of fibromatosis.  The fibroblasts are arranged in long fascicles with occasional intersecting bundles.  The nuclear atypia is minimal, and there are no clear signs of mitotic activity, further supporting a benign tumor.  Justification for Diagnosis:  Desmoid-type fibromatosis is characterized by the proliferation of fibroblasts in a dense collagenous stroma. The absence of significant nuclear atypia and mitotic figures, along with the dense collagenous background, is consistent with this diagnosis.  The haphazard growth pattern and irregular fascicular arrangement of the fibroblasts are notable features that match with fibromatosis. |
| 12.61 | Diagnosis: Schwannoma; Key Microscopic Features: Low-power view demonstrates well-organized Antoni A regions beside myxoid, less organized Antoni B areas, and at high power, Schwann cells in the Antoni A region form palisades around acellular Verocay body zones. | Histopathological Diagnosis:  Leiomyoma (a benign smooth muscle tumor)  Key Microscopic Features Supporting the Diagnosis:  Interlacing Fascicles of Spindle Cells:  Both images show bundles of spindle-shaped cells arranged in interlacing fascicles and whorled patterns, characteristic of smooth muscle origin.  Cigar-Shaped Nuclei:  At higher magnification (second image), the nuclei are elongated and blunt-ended (described as “cigar-shaped”), a classic feature of smooth muscle cells.  Minimal Atypia and Mitoses:  There is an absence of nuclear pleomorphism and no significant mitotic activity, which helps distinguish leiomyoma from leiomyosarcoma (its malignant counterpart).  Eosinophilic Cytoplasm:  The cells exhibit eosinophilic (pink-stained) cytoplasm, typical of smooth muscle differentiation under H&E stain.  Well-Circumscribed Lesion:  The low-magnification image (first image) shows a well-demarcated lesion, suggesting a benign, slow-growing nature.  These features are strongly indicative of leiomyoma, a benign smooth muscle tumor occasionally occurring in the oral and maxillofacial region, typically originating from vascular smooth muscle. | To provide an accurate histopathological diagnosis and analysis based on the provided hematoxylin-and-eosin (H&E) stained images, I will first interpret the key features in these images.  Please give me a moment to analyze the images.  Based on the H&E-stained images provided, the lesion appears to be a fibrous connective tissue lesion, likely indicative of a fibromatosis or a benign fibrous tumor (such as a desmoid fibromatosis or nodular fasciitis), given the arrangement and appearance of the cells.  Key Microscopic Features Justifying the Diagnosis:  Cellularity:  The images display a high cellularity with spindle-shaped fibroblasts and myofibroblasts. These cells are arranged in a haphazard or fascicular pattern, which is characteristic of desmoid tumors or nodular fasciitis.  Fibrous Stroma:  There is a dense fibrous stroma around the spindle cells, and this is prominent in the lower magnification (first image). The stroma consists of collagen bundles, which is typical of fibrous tissue tumors.  Mitotic Activity:  The images show some degree of mitotic figures, which suggests cellular turnover and the proliferative nature of the lesion. However, mitotic activity is not excessively high, pointing to a benign behavior.  Absence of Pleomorphism:  The spindle cells appear relatively uniform with no significant nuclear pleomorphism, which rules out malignancy.  Vascularity:  Vessels appear to be more numerous than in normal tissues, indicating increased vascularity, which is another characteristic of lesions like nodular fasciitis.  Conclusion:  Based on these histopathological features, the most likely diagnosis is Nodular Fasciitis or a Benign Fibromatosis. However, the arrangement of the cells and the lesion's infiltrative growth pattern point more towards desmoid fibromatosis if the lesion is deeper and has more invasive characteristics. |

Hematologic Disorders

| **Fig_no** | **Ground truth** | **GPT 4o answer** | **o3 answer** |
| --- | --- | --- | --- |
| 13.36 | Diagnosis: Mycosis Fungoides; Key Microscopic Features: In this high-power view of an oral biopsy, one can observe atypical, malignant lymphoid cells showing the characteristic cerebriform nuclear contour of mycosis fungoides (inset). | Histopathological Analysis of the Provided H&E Images  Most Likely Diagnosis:  Burkitt Lymphoma  Key Microscopic Features Supporting the Diagnosis:  High Mitotic Activity:  Seen in the second (higher magnification) image: Numerous mitotic figures, including atypical ones, are evident. This is characteristic of Burkitt lymphoma, which exhibits one of the highest proliferation rates among human tumors.  Monomorphic Population of Medium-sized Lymphoid Cells:  In the lower magnification image (first image), there is a dense sheet-like proliferation of intermediate-sized lymphocytes with round nuclei, coarse chromatin, and multiple small nucleoli.  "Starry Sky" Appearance:  While not strikingly obvious due to image limitations, some areas suggest interspersed macrophages with ingested apoptotic bodies creating pale spaces against the dark tumor cell background — a hallmark of Burkitt lymphoma.  Scant Cytoplasm and Basophilic Nature:  Cells exhibit a high nuclear-to-cytoplasmic ratio, and cytoplasm appears deeply basophilic, consistent with Burkitt lymphoma cells.  Summary:  The histopathological features — monomorphic intermediate lymphoid cells, extremely high mitotic rate, and suggestion of a “starry sky” pattern — strongly support a diagnosis of Burkitt lymphoma, a highly aggressive B-cell non-Hodgkin lymphoma often involving the jaw or maxilla in endemic cases. | Based on the provided hematoxylin-and-eosin (H&E) images and the context of hematologic disorders in oral and maxillofacial pathology, here is an analysis:  Most Likely Histopathological Diagnosis:  The lesion appears to be consistent with multiple myeloma, a hematologic disorder that can affect the oral and maxillofacial region, particularly in the form of a plasmacytoma.  Key Microscopic Features Supporting the Diagnosis:  Plasma Cell Proliferation:  In both images, there is a clear presence of plasma cells. Plasma cells are characterized by an eccentric nucleus, abundant cytoplasm, and a perinuclear clearing (known as "hof").  These plasma cells are seen in sheets and clusters, which is consistent with the monoclonal proliferation seen in multiple myeloma or plasmacytoma.  Characteristic Nuclear Features:  The nuclei of these plasma cells appear eccentric, and some show a peripheral condensation of chromatin.  The nuclear membrane is irregular, and the nucleolus is prominent, which is typical for plasma cell neoplasms.  Disordered Architecture:  The tissue exhibits a disorganized architecture with sheets of atypical plasma cells, and there is a disruption of normal tissue structure. This is often seen in malignant plasma cell conditions like multiple myeloma.  Increased Nuclear-to-Cytoplasmic Ratio:  Many of the plasma cells show an increased nuclear-to-cytoplasmic ratio and prominent mitotic figures, which suggest an aggressive and proliferative lesion.  These histological findings are in line with a diagnosis of multiple myeloma or its extramedullary counterpart, plasmacytoma, which can manifest in the oral cavity. |

Bone Pathology

| **Fig_no** | **Ground truth** | **GPT 4o answer** | **o3 answer** |
| --- | --- | --- | --- |
| 14.5 | Diagnosis: Osteopetrosis; Key Microscopic Features: A low‐power photomicrograph demonstrates abnormally dense bone supplanting normal cancellous architecture, with an inset highlighting a nodular arrangement of compact bone that obliterates the marrow spaces. | Histopathological Diagnosis:  Chondrosarcoma  Key Microscopic Features Supporting the Diagnosis:  Lobulated Growth Pattern:  The lesion demonstrates a lobular architecture with nodules of cartilage separated by fibrous septa, a classic feature of chondrosarcoma.  Abundant Cartilaginous Matrix:  Both images show a significant amount of bluish-purple cartilaginous matrix (basophilic), typical of cartilaginous tumors.  Chondrocytes with Nuclear Atypia:  The higher magnification image reveals chondrocytes in lacunae with:  Enlarged nuclei  Hyperchromasia  Occasional binucleation  These are cytological hallmarks of malignancy in a cartilaginous tumor.  Irregular and Permeative Growth:  The neoplastic cartilage invades surrounding bone and soft tissue, indicating a malignant process.  Lacunar Arrangement with Variable Cellularity:  Some lacunae appear crowded or contain more than one cell, which is atypical and supports malignancy.  Ruling Out Benign Lesions:  Chondroma lacks cellular atypia and mitotic activity.  Osteochondroma typically has a well-organized cartilage cap and no infiltration of surrounding bone.  Osteosarcoma with chondroid differentiation may appear similar but usually shows more aggressive features like lace-like osteoid. | Upon analyzing the hematoxylin-and-eosin stained images of the bone lesion in the oral and maxillofacial pathology group, the following points can be deduced:  Most Likely Histopathological Diagnosis:  The lesion depicted in the images is most consistent with Central Giant Cell Granuloma (CGCG).  Key Microscopic Features Justifying the Diagnosis:  Multinucleated Giant Cells: Both images show a characteristic presence of multinucleated giant cells, which are scattered throughout the lesion. These giant cells are a hallmark of CGCG and are seen in the first image, located around a central area.  Bone Resorption: The second image highlights the presence of areas with resorption of bone material, which is typical in CGCG. The giant cells are often involved in the resorptive process, leading to the characteristic bone lysis.  Loose Fibrous Stroma: In both images, a loose, vascular fibrous stroma is observed surrounding the giant cells. This stroma is often richly vascular, which is a key feature in CGCG.  Well-defined Lesion Boundaries: The lesion is often well-circumscribed, and this is evident in the first image, where there is a defined border around the lesion, helping differentiate CGCG from other bone lesions that may have ill-defined borders.  These microscopic features are consistent with a diagnosis of Central Giant Cell Granuloma, a benign but locally aggressive lesion that commonly affects the jaws and may present in patients with a history of pain, swelling, or expansion of the bone. |
| 14.24 | Diagnosis: Cherubism; Key Microscopic Features: Scattered multinucleated giant cells are set within a cellular, hemorrhagic mesenchymal background, and at higher magnification, there is eosinophilic cuffing surrounding the blood vessels. | Histopathological Diagnosis:  Central Giant Cell Granuloma (CGCG)  Key Microscopic Features Justifying the Diagnosis:  Multinucleated Giant Cells:  Scattered throughout the stroma, the first image shows numerous multinucleated giant cells with nuclei that are centrally clustered — a hallmark of CGCG.  These cells are typically osteoclast-like and located within a fibrous stroma.  Cellular Fibroblastic Stroma:  The background is composed of spindle-shaped fibroblasts in a vascular and collagenous matrix.  High cellularity is evident in the first image, consistent with the more active form of CGCG.  Areas of Hemorrhage and Hemosiderin Deposition:  The second image highlights foci of hemorrhage and possible hemosiderin-laden macrophages.  This is characteristic of CGCG and contributes to its often reddish-brown gross appearance.  Reactive Bone or Osteoid Formation:  Some areas suggest immature bone or osteoid production, although not prominently shown, which can occasionally occur in CGCG.  These features collectively support the diagnosis of Central Giant Cell Granuloma, a non-neoplastic but potentially aggressive lesion found most commonly in the anterior mandible of younger individuals. | Based on the provided hematoxylin-and-eosin images of the lesion in the Bone Pathology group of oral and maxillofacial pathology, here is the histopathological analysis:  Histopathological Diagnosis:  The most likely diagnosis based on the histological appearance of these lesions is Osteosarcoma.  Key Microscopic Features:  Cellular Pleomorphism: The presence of atypical and pleomorphic cells with variation in size and shape, particularly in the first image. This is characteristic of malignant lesions like osteosarcoma.  Increased Mitotic Activity: The images show a high rate of mitosis, including some abnormal mitotic figures. This is indicative of malignancy and suggests the aggressive nature of the tumor.  Lack of Normal Bone Formation: Osteosarcomas are characterized by the production of abnormal bone or osteoid by the tumor cells. The absence of well-formed bone and presence of osteoid matrix formation seen in some areas of these images supports this diagnosis.  Dense and Disorganized Stroma: The second image reveals a dense and somewhat disorganized fibroblastic stroma, another feature typical of osteosarcomas, which often exhibit both cellularity and a loose, fibrous background.  Spindle-Shaped Cells: The spindle-shaped cells in the lesion are suggestive of a high-grade osteosarcoma, where the tumor cells are less differentiated and more aggressive.  These histological features, especially cellular pleomorphism, mitotic figures, osteoid matrix, and the fibrous stroma, are typical of high-grade osteosarcoma in the bone. |
| 14.42 | Diagnosis: Fibrous Dysplasia; Key Microscopic Features: Irregular trabeculae of woven bone are embedded in a fibrous stroma, and a medium‐power view reveals peripheral osteoid without any osteoblastic rimming. | Based on the two provided hematoxylin and eosin (H&E) stained histological images of the same bone lesion at different magnifications, the most likely histopathological diagnosis is:  Ossifying Fibroma  Key Microscopic Features Supporting the Diagnosis:  Well-demarcated lesion with fibro-osseous architecture (more evident in the lower magnification image):  The lesion is sharply demarcated from surrounding bone, a classic hallmark of ossifying fibroma.  Mixed cellular fibrous stroma interspersed with mineralized material.  Presence of mineralized components resembling bone or cementum:  The higher magnification image shows trabeculae of woven or lamellar bone-like material.  The mineralized areas are rimmed by osteoblast-like cells in some foci.  Fibroblastic stroma:  The connective tissue is cellular with spindle-shaped fibroblasts, typically arranged in a storiform or whorled pattern.  Lack of atypia or mitotic activity:  There is no cytological atypia, supporting the benign nature of the lesion.  Differential Diagnosis Considered but Less Likely:  Fibrous dysplasia – ruled out due to the well-circumscribed nature and presence of osteoblastic rimming, which is usually absent in fibrous dysplasia.  Cemento-ossifying fibroma – sometimes considered a subtype of ossifying fibroma, especially in the jaws; however, it shares identical features histologically. | Based on the hematoxylin-and-eosin-stained histopathological images provided, here is the analysis:  Single Most Histopathological Diagnosis:  The images are consistent with Osteosarcoma, a malignant bone tumor that is characterized by the production of osteoid or immature bone by malignant cells.  Key Microscopic Features Seen in the Images:  First Image (Lower Magnification):  Disorganized and Pleomorphic Cell Patterns: The image shows a proliferation of spindle-shaped cells with a disorganized architecture. There are areas of bony matrix formation (osteoid), and some regions exhibit a more chaotic arrangement of cells.  High Cellular Density: The field appears densely cellular, with irregular bone trabeculae, indicative of malignant osteoblasts producing abnormal bone.  Second Image (Higher Magnification):  Irregular Osteoid Formation: The presence of irregular and immature bone (osteoid) being produced by the malignant cells is more evident at higher magnification.  Pleomorphic Malignant Cells: The cells show significant variation in shape and size (pleomorphism), with hyperchromatic and irregular nuclei, a hallmark of malignancy.  Mitotic Figures: There are several mitotic figures observed, which are indicative of rapid cell division and malignancy.  Lack of Mature Bone: The bone matrix seen is unmineralized (osteoid), which is typically seen in osteosarcoma and is a distinguishing feature from benign bone lesions.  Conclusion:  These features point toward a diagnosis of Osteosarcoma, a common primary malignant bone tumor, especially in the context of the oral and maxillofacial region. The combination of pleomorphic cells, disorganized bone formation, and the presence of osteoid confirms this diagnosis. |
| 14.43 | Diagnosis: Mature Fibrous Dysplasia; Key Microscopic Features: Within this long‐standing lesion, broad, discrete trabeculae of bone lie in fibrous connective tissue, showing clear lamellar maturation of the bone. | Histopathological Diagnosis:  Cemento-ossifying fibroma (COF)  Key Microscopic Features Supporting the Diagnosis:  Well-demarcated lesion with fibrous stroma:  The lesion in both images is encapsulated or well-circumscribed, a hallmark of cemento-ossifying fibroma, distinguishing it from fibrous dysplasia.  The stroma is composed of cellular fibrous connective tissue.  Presence of mineralized material:  Both images reveal round to irregular areas of mineralized tissue embedded within the fibrous stroma.  These mineralized areas resemble bone (lamellar or woven) and cementum-like structures (round, acellular, eosinophilic basophilic bodies), typical of COF.  Concentric lamellar bony trabeculae:  Especially visible in the second image, showing lamellar bone with osteocytes in lacunae and concentric growth patterns, consistent with mature bone formation.  Lack of infiltration into surrounding bone:  Unlike fibrous dysplasia, there is no blending of the lesion with adjacent bone, reinforcing the diagnosis of a well-demarcated benign neoplasm like COF.  Summary:  The combination of fibrous stroma, presence of both cementum-like and bony components, and a well-circumscribed growth pattern seen in the provided H&E sections strongly supports the diagnosis of Cemento-ossifying fibroma—a benign fibro-osseous lesion commonly occurring in the jawbones. | Based on the provided histopathological images, here is the analysis:  Histopathological Diagnosis:  The images suggest a diagnosis of Central Giant Cell Granuloma (CGCG). This lesion typically occurs within the jawbones and is commonly seen in the oral and maxillofacial region.  Key Microscopic Features:  Presence of Multinucleated Giant Cells:  Both images display multinucleated giant cells scattered throughout the lesion. These are large cells with multiple nuclei, which is a hallmark of CGCG.  Fibrous Stroma:  The surrounding tissue appears to have a fibrous stroma, which is indicative of a connective tissue matrix commonly seen in CGCG.  Well-Defined Lesional Borders:  The lesion has well-defined borders, often seen in CGCG, and the bone resorption is visible in the first image.  Bone Resorption:  There is evidence of bone resorption and the presence of bone lacunae (spaces where osteocytes reside), particularly in the first image. This is a characteristic feature of CGCG as it often leads to bone destruction.  Conclusion:  The combination of multinucleated giant cells, fibrous stroma, and evidence of bone resorption points to Central Giant Cell Granuloma as the most likely diagnosis. |

Odontogenic Cysts and Tumors

| **Fig_no** | **Ground truth** | **GPT 4o answer** | **o3 answer** |
| --- | --- | --- | --- |
| 15.44 | Diagnosis: Lateral Periodontal Cyst; Key Microscopic Features: The photomicrograph reveals a delicate epithelial lining with localized nodular thickenings that often display a swirling cell pattern. | Histopathological Analysis  Single Most Likely Diagnosis:  Calcifying Odontogenic Cyst (COC) (also known as Gorlin cyst)  Key Microscopic Features Supporting the Diagnosis:  1. Ghost Cells:  Description: Pale eosinophilic anucleate cells (seen in the second image) within the epithelial lining or extending into the connective tissue.  Significance: A hallmark feature of COC, ghost cells represent aberrant keratinization.  2. Cystic Structure:  Description: The first image at lower magnification shows a cystic cavity lined by ameloblastoma-like epithelium with basal cells showing palisading and reverse nuclear polarity.  Significance: The ameloblastoma-like epithelial lining supports an odontogenic origin, typical of COC.  3. Basal Cell Palisading and Hyperchromatic Nuclei:  Seen in both images: Particularly in the lower half of the epithelium, consistent with odontogenic epithelium.  4. Fibrous Connective Tissue Wall:  Description: Surrounding the cyst is a fibrous wall with sparse inflammation.  Significance: Characteristic of odontogenic cysts.  5. Possible Calcifications:  Although not prominent in these sections, calcifications often accompany ghost cells in COC and may be visible in other sections. | The images you provided show histopathological slides with hematoxylin-and-eosin (H&E) staining. These belong to the Odontogenic Cysts and Tumors group of oral and maxillofacial pathology.  Based on the appearance of the lesions in the provided images, the single most likely diagnosis is Keratocystic Odontogenic Tumor (KCOT).  Key Microscopic Features Justifying the Diagnosis:  Cyst Lining:  The first image demonstrates a cystic structure with a lining consisting of a uniform layer of epithelium that is keratinized. This is characteristic of the keratocystic odontogenic tumor.  The lining epithelium appears stratified squamous, and the basal layer has a palissading arrangement, where cells are tall and columnar.  Presence of Orthokeratin or Parakeratinized Epithelium:  The keratinized layer in the cystic wall can appear as either orthokeratinized or parakeratinized epithelium, which is common in KCOT.  Focal Hyperchromatic Basal Layer:  The basal layer of the epithelium is hyperchromatic, which refers to cells with dark-staining nuclei, an important feature of the KCOT's epithelium.  Chronic Inflammation in the Connective Tissue:  In both images, you can observe chronic inflammatory cells (such as lymphocytes and plasma cells) within the surrounding connective tissue, which is typical in cystic lesions like KCOT.  Thin Fibrous Capsule:  The connective tissue stroma surrounding the cyst is typically thin, and this is another distinguishing feature of KCOT.  These features, seen in the provided histopathological images, are consistent with a keratocystic odontogenic tumor (KCOT). |
| 15.94 | Diagnosis: Adenomatoid Odontogenic Tumor; Key Microscopic Features: At low magnification, a dense capsule encases the tumor, while at higher magnification, duct‐like epithelial structures are apparent with columnar cell nuclei oriented away from the luminal spaces. | Based on the provided hematoxylin and eosin (H&E) stained histological images of a lesion within the odontogenic cysts and tumors category, viewed at both low and high magnifications, the single most likely histopathological diagnosis is:  Ameloblastoma (Multicystic/Follicular type)  Key Microscopic Features Supporting the Diagnosis:  Low Power View (Image 1):  Multicystic architecture: The lesion shows multiple cystic spaces, characteristic of a multilocular tumor, which is a hallmark of multicystic ameloblastoma.  Expansile growth: The lesion expands into the surrounding connective tissue without encapsulation, which is typical for ameloblastomas.  Lobulated configuration: The tumor islands are separated by fibrous stroma, giving a lobulated appearance.  High Power View (Image 2):  Follicular pattern: Numerous epithelial islands resembling the enamel organ, with a clear peripheral palisading of columnar basal cells.  Reverse polarity of nuclei: Nuclei of the peripheral columnar cells are displaced away from the basement membrane.  Central stellate reticulum-like cells: Loosely arranged central cells mimic the stellate reticulum seen in the enamel organ.  No significant atypia or mitotic figures, supporting a benign but locally aggressive nature.  Summary:  These histological features are characteristic of follicular ameloblastoma, a common variant of ameloblastoma, which is a benign but locally aggressive odontogenic tumor. | The images you provided show histopathological sections of a lesion, and after examining the provided slides, it appears to be consistent with an Odontogenic Keratocyst (OKC). Here are the key microscopic features supporting this diagnosis:  Diagnosis:  Odontogenic Keratocyst (OKC)  Key Microscopic Features:  Layering of the Epithelium:  The first image (low magnification) shows a cystic cavity lined by a thin epithelium. Upon zooming in (second image), the epithelium is seen to exhibit prominent palisading along the basal layer, with a characteristic corrugated surface.  Keratinization:  The cyst lining in OKC demonstrates orthokeratinization (in some areas) with a smooth keratinized surface, which is seen in the first image as well.  Thin Cyst Wall:  The second image reveals a relatively thin cyst wall and the lack of significant connective tissue, a feature that differentiates OKC from other cysts which have thicker walls.  Satellite Cysts:  Satellite cysts, which are small cystic spaces within the surrounding connective tissue, are seen in the first image. These are a hallmark of OKC and contribute to its aggressive behavior and recurrence potential.  No Inflammatory Infiltrate:  There is a lack of significant inflammatory infiltrate in the wall, which is another key feature distinguishing OKC from other odontogenic cysts that tend to show more inflammation.  Justification for Diagnosis:  The presence of the characteristic palisaded basal layer and corrugated epithelium, along with keratinization, thin cyst walls, and the absence of inflammation or significant fibrous tissue, are the defining microscopic features that suggest the lesion is an Odontogenic Keratocyst (OKC).  This diagnosis is supported by the distinct histological appearance seen in the provided images. |
| 15.98 | Diagnosis: Calcifying Epithelial Odontogenic Tumor; Key Microscopic Features: Broad sheets of neoplastic epithelial cells surround pools of amorphous eosinophilic amyloid containing focal calcifications; at high power, polygonal cells with eosinophilic cytoplasm and intercellular bridges are noted alongside adjacent amyloid deposits. | Based on the two provided hematoxylin and eosin (H&E) stained histopathological images of a lesion in the odontogenic cysts and tumors category, the most likely diagnosis is:  Calcifying Odontogenic Cyst (COC), also known as Gorlin cyst.  Key Microscopic Features Supporting the Diagnosis:  Ghost Cells (Visible in both images, especially the first one):  These are eosinophilic (pink), enlarged, keratinized epithelial cells that have lost their nuclei.  Ghost cells are a hallmark of COC and may calcify, as seen in the large dark (basophilic) calcified areas within ghost cell clusters in the first image.  Calcifications:  Seen prominently in the first image: a large area of dystrophic calcification, likely associated with ghost cells. This is common in COC.  Ameloblastoma-like Epithelium:  The second image highlights epithelial islands that have peripheral palisading and hyperchromatic nuclei—resembling ameloblastoma—but with central stellate reticulum-like cells.  This ameloblastoma-like epithelial component is a known feature of COC.  Cystic and Solid Areas:  The lesion shows areas of cystic degeneration (clear spaces), often associated with COC.  There’s also a mix of solid epithelial proliferations in a fibrous stroma.  These features—especially the ghost cells, calcifications, and ameloblastoma-like epithelial islands—strongly support the diagnosis of Calcifying Odontogenic Cyst. | Single most likely diagnosis  → Calcifying Epithelial Odontogenic Tumor (CEOT, “Pindborg tumor”)  Key microscopic features visible in the two H‑E fields  # Feature seen Why it points to CEOT  1 Sheets/nests of polygonal odontogenic epithelial cells with well‑defined cell borders and obvious inter‑cellular bridges (best appreciated in the high‑power image) CEOT is composed of polyhedral epithelial cells that resemble squamous/stellate‑reticulum‑type odontogenic epithelium rather than palisaded ameloblastic cells.  2 Mild‑to‑moderate nuclear pleomorphism with some enlarged, occasionally hyperchromatic nuclei but scant mitoses CEOT typically shows nuclear variability yet behaves as a low‑grade neoplasm, matching this cytology.  3 Abundant extracellular, pale eosinophilic, homogeneous material between and around the epithelial cells This material represents the amyloid‑like protein that is characteristic of CEOT (Congo‑red positive, apple‑green birefringence on polarised light).  4 Foci of calcification—classical concentric “Liesegang rings”; in the low‑power field they appear as a dark basophilic/blackish mass within the amyloid pool Deposition of calcium within the amyloid produces the pathognomonic Liesegang‑ring calcifications of a Pindborg tumour.  5 Fibrous connective‑tissue stroma separating epithelial islands without a capsule CEOT grows in solid epithelial masses set in a fibrous stroma, rather than a cystic cavity or duct‑like structures (which would favour AOT).  The combination of polygonal epithelial sheets, amyloid‑like material, and Liesegang‑ring calcifications is diagnostic for a calcifying epithelial odontogenic tumor and distinguishes it from other entities in the odontogenic cyst/tumor spectrum. |
| 15.102 | Diagnosis: Squamous Odontogenic Tumor; Key Microscopic Features: The low‐power view shows islands of bland squamous epithelium set in a fibrous stroma, while the high‐power image highlights the innocuous epithelial cells forming small cystic spaces. | Histopathological Analysis of the Provided H&E Images  Most Likely Diagnosis:  Ameloblastoma (Follicular Type)  Key Microscopic Features Supporting This Diagnosis:  Low-Power Image (First Image):  Multiple epithelial islands are seen scattered within a fibrous connective tissue stroma.  These islands are well-demarcated, suggesting a neoplastic process.  The follicular pattern is prominent — islands of tumor cells resemble developing enamel organs.  High-Power Image (Second Image):  The epithelial islands show:  Peripheral palisading of columnar cells with reverse nuclear polarity (nuclei away from the basement membrane), characteristic of ameloblast-like cells.  Central stellate reticulum-like cells, mimicking the star-shaped cells found in the enamel organ.  The connective tissue is mature and fibrous, with no signs of inflammation or cystic degeneration in this specific view.  Summary of Diagnostic Features Consistent with Follicular Ameloblastoma:  Islands of odontogenic epithelium.  Palisaded peripheral columnar cells with reverse polarization.  Central stellate reticulum-like cells.  Follicular architectural arrangement. | Histopathological diagnosis  → Conventional (solid / multicystic) ameloblastoma – acanthomatous variant  Key microscopic features visible in the two fields  Field Feature seen Significance for diagnosis  Low‑power overview (first image) • Numerous, variably‑sized epithelial follicles/nodules and cystic spaces scattered through a fibrous connective‑tissue stroma. Multicystic / follicular growth pattern that is typical of the conventional solid ameloblastoma.  • Each follicle is delimited by a thin ribbon of epithelium; many show central cystic degeneration. The follicular pattern with secondary cyst formation is characteristic and helps separate it from a single‑lumen odontogenic cyst.  High‑power view (second image) • Peripheral tall columnar cells arranged in a palisade with hyperchromatic, basally aligned nuclei that show reverse polarity (nuclei away from the basement membrane) and occasional sub‑nuclear vacuoles. Classic “ameloblast‑like” lining of an ameloblastoma follicle.  • Central squamous metaplasia with laminated keratin (keratin pearls) replacing the usual loosely arranged stellate‑reticulum‑like cells. Defines the acanthomatous variant of ameloblastoma. Presence of orderly keratin pearls without cytologic atypia distinguishes it from squamous‑cell carcinoma or a calcifying odontogenic cyst.  • No significant cellular atypia or invasion beyond the follicular borders; stroma is mature and fibrous. Confirms a benign but locally aggressive odontogenic tumour rather than a malignant counterpart.  Taken together, the multicystic/follicular architecture with peripheral palisading ameloblast‑like cells and prominent squamous/keratinising change in the follicle centres is diagnostic of an acanthomatous ameloblastoma. |
| 15.105 | Diagnosis: Ameloblastic Fibroma; Key Microscopic Features: Elongated, narrow cords of odontogenic epithelium lie within a richly cellular primitive connective tissue, and basophilic epithelial islands exhibit peripheral nuclear palisading. | Histopathological Diagnosis:  Adenomatoid Odontogenic Tumor (AOT)  Justification and Key Microscopic Features:  The images provided, captured at different magnifications, show classic features of an Adenomatoid Odontogenic Tumor, a benign epithelial odontogenic tumor. Here's how the images support this diagnosis:  Low-power View (Image 1):  Multinodular/plexiform architecture: Islands, cords, and nests of odontogenic epithelium are set in a fibrous stroma.  Rosette-like and duct-like structures: Pseudoductal patterns are visible, a hallmark of AOT.  The overall growth pattern is non-invasive, indicating a benign lesion.  High-power View (Image 2):  Columnar/cuboidal epithelial cells: Arranged in a duct-like or tubular pattern, sometimes forming rosettes, mimicking glandular differentiation.  Polarized nuclei: The nuclei are often aligned away from the luminal surface in the duct-like spaces.  Scant cytoplasm and minimal mitotic activity, consistent with benign nature.  Some areas may show eosinophilic material in lumina, representing amyloid-like or hyaline material.  Summary:  These histological features — duct-like epithelial structures, rosette formations, and the benign appearance of odontogenic epithelial cells — are diagnostic of Adenomatoid Odontogenic Tumor (AOT). | Histopathological diagnosis  Ameloblastic fibroma (benign mixed odontogenic tumour)  Microscopic features in the two fields that lead to this diagnosis  Field Key findings Significance for diagnosis  Low‑power view • Numerous thin, branching and anastomosing cords/strands and small follicles of odontogenic epithelium scattered through the lesion  • Cords lie in a loose, cell‑rich, myxoid connective‑tissue stroma that resembles primitive dental papilla Classic two‑component (“mixed”) pattern of an ameloblastic fibroma: epithelial component + immature ectomesenchyme  High‑power view • Epithelial islands show a peripheral rim of columnar to cuboidal cells; the nuclei are basophilic, often oriented away from the basement membrane (ameloblast‑like)  • Central cells are more loosely arranged, mimicking stellate reticulum; occasional tiny lumen‑like spaces may be present  • Surrounding mesenchyme is plump, fibroblast‑rich and myxoid, not mature fibrous tissue Reproduces the histology of the early enamel organ and dental papilla, confirming a mixed odontogenic tumour rather than a purely epithelial lesion  Entire lesion (both images) • No enamel, dentin or calcified material is seen → rules out ameloblastic fibro‑odontoma / odontoma  • No extensive duct‑like structures or calcifications → rules out adenomatoid odontogenic tumour  • Islands are small and set in abundant ectomesenchyme, unlike the broad epithelial sheets of conventional ameloblastoma Helps narrow the differential specifically to ameloblastic fibroma  The combination of (1) proliferating odontogenic epithelial strands/follicles with ameloblast‑like peripheral cells, (2) a primitive, cell‑rich myxoid mesenchyme, and (3) absence of hard‑tissue formation is diagnostic of ameloblastic fibroma. |
| 15.109 | Diagnosis: Ameloblastic Fibro-Odontoma; Key Microscopic Features: The soft tissue component is indistinguishable from an ameloblastic fibroma, accompanied by areas of disorganized tooth‐like structure formation. | Histopathological Diagnosis:  Glandular Odontogenic Cyst (GOC)  Key Microscopic Features Supporting the Diagnosis:  From both low- and high-power hematoxylin and eosin (H&E) stained images, the following characteristic histological features are observed:  Multicystic architecture with epithelial lining:  The low-power view shows a cystic lesion with variable-sized locules, consistent with a multilocular cystic structure.  The lining is non-uniform in thickness, typical of GOC.  Epithelial lining with glandular (duct-like) structures:  The high-power image shows epithelial cells forming duct-like or glandular spaces, which are pathognomonic for GOC.  These pseudo-glandular spaces contain eosinophilic material and sometimes mucous cells.  Presence of mucous (goblet) cells:  Mucous cells are evident within the epithelium, often producing mucin—a hallmark of GOC.  Hobnail (surface cuboidal) cells:  The epithelium occasionally displays surface hobnail or cuboidal cells with eosinophilic cytoplasm.  Clear cells in the basal and suprabasal layers:  Clear cells may be noted, supporting the diagnosis, although not always prominent.  Epithelial spheres or plaque-like thickenings:  Focal nodular thickenings or plaques of epithelial proliferation can be seen within the cyst lining.  Summary:  The combination of glandular structures, mucous cells, hobnail cells, and multicystic growth within an odontogenic lesion strongly points to a Glandular Odontogenic Cyst (GOC)—a rare but locally aggressive developmental cyst of odontogenic origin. | Histopathological diagnosis  → Dentinogenic ghost‑cell tumor (DGCT) — the solid/neoplastic counterpart of the calcifying odontogenic cyst  Microscopic hallmarks visible in the two fields  # Key feature in the images Diagnostic significance  1 Ameloblastoma‑like epithelial component – inter‑anastomosing cords and islands of odontogenic epithelium with peripheral palisading and stellate‑reticulum‑like cells (best appreciated on the low‑power view). Shows that the lesion is a solid odontogenic tumour rather than a simple cyst lining.  2 Ghost cells – groups of enlarged, uniformly eosinophilic epithelial cells that have lost their nuclei (central cluster in the high‑power view). Ghost‑cell production is the signature change of the calcifying/ghost‑cell odontogenic lesions.  3 Dystrophic calcification of ghost cells – granular or basophilic foci within or around the ghost‑cell masses. Further supports a ghost‑cell lesion; calcification is frequent in DGCT.  4 Dentinoid/osteoid‑like material – broad bands of homogeneous, pink, tubular matrix laid down adjacent to epithelial nests (right side of high‑power image). “Dentinogenic” element that distinguishes DGCT from the purely cystic calcifying odontogenic cyst.  5 Fibro‑cellular stroma without atypia surrounding the epithelial nests. Typical benign supportive tissue of DGCT.  The combination of ameloblastomatous epithelial proliferation plus ghost‑cell formation plus dentinoid deposition in a solid tumour mass is diagnostic of a dentinogenic ghost‑cell tumour. |
| 15.132 | Diagnosis: Odontogenic Myxoma; Key Microscopic Features: A loose myxomatous tumor fills the marrow space between bony trabeculae, and at higher power, loosely arranged spindle cells with fine collagen fibrils are seen along with a small odontogenic epithelial rest centrally. | Histopathological Diagnosis:  Odontogenic Myxoma  Key Microscopic Features Supporting the Diagnosis:  Low-power image (first image):  Lobulated architecture: The lesion appears poorly circumscribed with a lobulated pattern.  Myxoid stroma: The connective tissue is loose and myxoid (pale-staining), characteristic of abundant ground substance and minimal collagenization.  Entrapped bone trabeculae: There is evidence of residual or entrapped bone along the periphery, which can often be seen in intraosseous odontogenic tumors like odontogenic myxoma.  High-power image (second image):  Stellate and spindle-shaped fibroblastic cells: Scattered within the myxoid matrix, these are hallmark cells of odontogenic myxoma.  Loose, basophilic extracellular matrix: Abundant ground substance with minimal cellularity and few capillaries.  Lack of encapsulation: Consistent with the infiltrative growth pattern typical of myxomas.  These features together point strongly toward odontogenic myxoma, a benign but locally aggressive neoplasm of odontogenic mesenchymal origin. | Histopathological diagnosis  Desmoplastic fibroma of the jaw (intra‑osseous counterpart of desmoid‑type fibromatosis)  Key microscopic features visible in the two H&E fields  Magnification What you can see How it supports the diagnosis  Low‑power overview (first image) • A solid, unencapsulated but well‑circumscribed mass completely filling the medullary cavity and pushing against a shell of pre‑existing cortical bone (the intensely eosinophilic plates at the superior margin).  • The tumour itself is pale‑pink, uniformly collagenised and obviously hypocellular. A fibrous lesion that originates within bone and replaces cancellous trabeculae is the classic growth pattern of desmoplastic fibroma. The dense collagen and paucicellularity immediately argue against the common odontogenic tumours that contain either epithelial rests or calcified matrix.  Higher magnification (second image) • Scattered, bland, spindle‑shaped fibroblasts with thin, tapered nuclei embedded in thick, wavy collagen bundles.  • Cells are arranged in long sweeping fascicles and subtle whorls around small capillary‑sized vessels (centre of the field).  • Virtually no mitotic figures, no nuclear atypia, no ghost cells, no enamel/dentinoid, and no odontogenic epithelial strands. The combination of an extremely hypocellular, densely collagenous stroma with bland spindle cells in sweeping / whorled bundles, plus the absence of any epithelial component or calcifications, is diagnostic for desmoplastic fibroma and rules out: central odontogenic fibroma (would show odontogenic epithelium), ossifying/cemento‑ossifying fibroma (would show bone or cementum‑like deposits), odontogenic myxoma (would be myxoid rather than densely collagenous), and any odontogenic cyst (would require an epithelial lining).  Bottom line:  A monotonous, hypocellular, densely fibrous intra‑osseous tumour composed of bland spindle fibroblasts with sweeping fascicles and no odontogenic epithelium or mineralised product is characteristic of desmoplastic fibroma of the jaw. |

Dermatologic Diseases

| **Fig_no** | **Ground truth** | **GPT 4o answer** | **o3 answer** |
| --- | --- | --- | --- |
| 16.10 | **Diagnosis:** Hereditary Benign Intraepithelial Dyskeratosis (HBID); **Key Microscopic Features:** Under moderate magnification, the epithelium exhibits pronounced hyperparakeratosis, acanthosis, and dyskeratosis, and at higher magnification, individual dyskeratotic cells are identified. | Based on the histopathological examination of the provided hematoxylin and eosin (H&E) stained sections of the same lesion at low and high magnifications, the single most likely diagnosis is Lichen Planus.  🔍 Key Microscopic Features Justifying the Diagnosis:  Low Magnification Image:  Saw-toothed rete ridges: The epithelial projections into the connective tissue have a pointed, irregular appearance.  Hyperkeratosis: Thickening of the keratin layer on the surface.  Band-like lymphocytic infiltrate: Dense band of chronic inflammatory cells (primarily lymphocytes) hugging the epithelial-connective tissue interface.  Acanthosis: Thickening of the spinous layer of the epithelium.  High Magnification Image:  Civatte bodies (colloid bodies): Eosinophilic, round apoptotic keratinocytes in the lower epithelial layers or superficial connective tissue.  Basal cell degeneration: The basal layer shows hydropic (vacuolar) degeneration, typical of interface dermatitis.  Lymphocytic infiltrate: Lymphocytes closely associated with the basal layer, often obscuring the dermoepidermal junction.  These features are classic for oral lichen planus, particularly the reticular or erosive type commonly seen in the oral mucosa. | Histopathologic diagnosis — Oral lichen planus (lichenoid mucositis)  Key microscopic features in the two fields  Image Field‑of‑view Features that support the diagnosis  1 Low‑power, epithelium + lamina propria • Surface hyper‑ortho/para‑keratosis  • Epithelium is thinned in places but shows sharply pointed, “saw‑tooth” rete ridges rather than broad or blunted pegs  • Immediately beneath the epithelium a dense, band‑like, predominantly lymphocytic infiltrate hugs the epithelial–connective tissue interface (“lichenoid” pattern)  2 High‑power, intra‑epithelial • Liquefaction (hydropic) degeneration of the basal cell layer with loss of the normal basal keratinocyte row  • Numerous Civatte/colloid bodies – round, eosinophilic, apoptotic keratinocytes scattered in the lower epithelium and interface  • Spinosum shows minimal acanthosis and no true acantholysis or intra‑epithelial clefting (helps exclude pemphigus)  Together, the saw‑tooth rete ridges, interface lymphocytic (lichenoid) band, basal‑cell liquefaction, and Civatte bodies are the classic quartet for oral lichen planus, distinguishing it from other dermatologic/interface conditions of the oral cavity (e.g., lupus erythematosus, pemphigoid, erythema multiforme). |
| 16.101 | **Diagnosis:** Lichen Planus; **Key Microscopic Features:** On low-power, the lesion displays hyperkeratosis, sawtooth rete ridges, and a continuous band of lymphocytes beneath the epithelium, while on high-power, lymphocytes infiltrate the lower epithelial layers causing basal cell interface degeneration. | Based on the provided hematoxylin-and-eosin stained histopathological images (low and high magnification) of a lesion from the oral mucosa, the most likely diagnosis is:  Lichen Planus (Oral Lichen Planus)  Key Microscopic Features Justifying the Diagnosis:  Saw-tooth Rete Ridges:  The rete ridges in the epithelium show a characteristic "saw-tooth" pattern, which is highly suggestive of lichen planus.  Dense Band-like Lymphocytic Infiltrate:  At the interface between the epithelium and connective tissue, there is a dense, band-like infiltrate composed predominantly of T-lymphocytes.  Basal Cell Degeneration:  There is evidence of liquefaction degeneration (hydropic degeneration) of the basal cell layer, with damage to basal keratinocytes.  Civatte (Colloid) Bodies:  Though not clearly visible in these images, in lichen planus you may find eosinophilic apoptotic keratinocytes known as Civatte bodies, particularly in the basal layer or upper lamina propria.  Hyperkeratosis and Hypergranulosis:  There is a thickened keratin layer, which is often orthokeratotic or parakeratotic, and granular cell layer thickening, typical of the hyperkeratotic variant of lichen planus.  These histological findings are characteristic of oral lichen planus, a chronic immune-mediated mucocutaneous disorder. | Most likely histopathological diagnosis  → Oral lichen planus (lichenoid/interface mucositis pattern).  Key microscopic features that support this diagnosis  Feature seen in the photomicrographs How it points to lichen planus  1. Intact but hyper‑keratinised stratified squamous epithelium with a thick, compact surface keratin layer Hyper‑ortho/parakeratosis is typical in the reticular/erosive forms of oral lichen planus (OLP).  2. “Saw‑tooth” (pointed, wedge‑shaped) rete ridges projecting into the connective tissue This altered epithelial architecture is classic for OLP and uncommon in most other interface reactions.  3. Liquefactive (hydropic) degeneration of the basal cell layer; scattered eosinophilic colloid/Civatte bodies may be present Basal‑cell degeneration is the hallmark of an interface/lichenoid mucositis such as OLP.  4. Dense, continuous, band‑like inflammatory infiltrate hugging the epithelial–connective‑tissue junction and obscuring the dermal–epidermal interface (predominantly small lymphocytes) A sharply band‑shaped, subepithelial T‑cell infiltrate is the single most characteristic microscopic feature of lichen planus.  5. Relative paucity of plasma cells, neutrophils, or eosinophils Reinforces that this is a lichenoid (T‑cell–mediated) rather than a plasmacytic or neutrophil‑rich process.  The constellation of a smooth, ribbon‑like lymphocytic infiltrate, basal‑cell degeneration with Civatte bodies, and the saw‑toothed rete architecture in an oral mucosal biopsy is essentially diagnostic of oral lichen planus. |
| 16.102 | **Diagnosis:** Lichen Planus; **Key Microscopic Features:** At high magnification, normal mucosa shows an intact basal layer without inflammation; in contrast, lichen planus features degeneration of the basal epithelium and a dense lymphocytic infiltrate in the superficial lamina propria. | Histopathological Diagnosis:  The single most likely diagnosis based on the provided hematoxylin and eosin-stained images is oral lichen planus (OLP).  Key Microscopic Features Supporting This Diagnosis:  Saw-tooth Rete Ridges:  In the first image (lower magnification), the epithelial rete pegs appear irregular and may show a "saw-tooth" configuration, which is a classic architectural change in OLP.  Hyperkeratosis and Acanthosis:  There is thickening of the keratin layer (hyperkeratosis) and thickening of the spinous layer (acanthosis), common in lichen planus.  Basal Cell Degeneration:  Both images show vacuolar degeneration of the basal cell layer. The basal keratinocytes appear damaged, which is a hallmark of lichenoid mucositis.  Civatte (Colloid) Bodies:  Eosinophilic, round apoptotic keratinocytes, also known as Civatte bodies, are visible, particularly near the basal layer.  Band-like Lymphocytic Infiltrate:  In the second image (higher magnification), a dense, band-like infiltrate of lymphocytes is present in the subepithelial connective tissue, which closely hugs the epithelial-connective tissue junction — a defining feature of OLP.  These histological characteristics — especially the band-like lymphocytic infiltrate with basal cell liquefaction degeneration and the presence of Civatte bodies — are pathognomonic for oral lichen planus. | Histopathologic diagnosis (single best answer)  Oral lichen planus – lichenoid interface mucositis  Key microscopic features that support this diagnosis  Feature What is seen in the provided sections Significance  Hyper-orthokeratosis / parakeratosis with “saw-tooth” epithelial ridges The surface epithelium is covered by a thick eosinophilic keratin layer; the rete processes are short, pointed and angulated, giving a serrated outline. Characteristic architectural change in lichen planus.  Liquefaction (hydropic) degeneration of the basal cell layer Basal keratinocytes appear swollen/vacuolated and many are indistinct or lost, producing a ragged, pale band immediately above the connective tissue. Hallmark basal cell injury produced by a cell-mediated immune attack.  Civatte (colloid) bodies Eosinophilic, round, anucleate bodies are scattered within the lower epithelium and at the epithelial-connective tissue interface. Represent apoptotic keratinocytes typical of lichenoid reactions.  Dense, band-like subepithelial lymphocytic infiltrate A continuous sheet of small, darkly staining lymphocytes hugs the undersurface of the epithelium, obscuring the normal interface; exocytosis of lymphocytes into the epithelium is also present. Classic “lichenoid” pattern; predominance of T-cells in lichen planus.  Absence of epithelial dysplasia or true acantholysis / subepithelial clefting Although the spinous layer is hyperplastic, cellular maturation is preserved and there is no suprabasilar split or tomb-stone basal layer. Helps exclude pemphigus vulgaris, mucous-membrane pemphigoid or dysplastic leukoplakia.  Taken together, the combination of a lichenoid band of lymphocytes, basal cell degeneration with Civatte bodies, and saw-tooth rete ridges beneath a hyperkeratotic surface is diagnostic of oral lichen planus in the setting of dermatologic diseases affecting the oral mucosa. |
| 16.106 | **Diagnosis:** Chronic Ulcerative Stomatitis; **Key Microscopic Features:** Under low-power, there is epithelial atrophy with a heavy chronic inflammatory infiltrate in the superficial lamina propria; at high-power, the basal epithelium shows interface degeneration with inflammation that, unlike lichen planus, contains numerous plasma cells in addition to lymphocytes. | Histopathological Analysis of the Provided H&E Images  Single Most Likely Diagnosis:  Lichen Planus  Key Microscopic Features Supporting the Diagnosis:  Saw-tooth Rete Ridges:  The lower-power image (first image) shows irregular, saw-toothed elongation of the rete ridges, a characteristic feature of lichen planus.  Band-like Lymphocytic Infiltrate:  A dense, band-like infiltrate of lymphocytes is present at the interface between the epithelium and connective tissue (seen clearly in both low- and high-power images). This pattern of inflammation is a hallmark of lichenoid mucositis.  Basal Cell Degeneration (Hydropic Degeneration):  The high-power image (second image) reveals degeneration of basal keratinocytes, often referred to as Civatte or colloid bodies, which appear as eosinophilic apoptotic cells in the basal layer.  Hyperkeratosis and Hypergranulosis:  The surface epithelium exhibits hyperkeratosis (thickened keratin layer), commonly seen in oral lichen planus, especially in the reticular form.  "Interface Dermatitis" Pattern:  The destruction at the epithelial-connective tissue junction with a lichenoid interface pattern is indicative of lichen planus.  These features—particularly the band-like lymphocytic infiltrate, basal cell degeneration, and saw-tooth rete ridges—are classic for oral lichen planus, a chronic mucocutaneous condition frequently seen in the oral cavity. | Histopathological diagnosis (single most likely):  Oral lichen planus – lichenoid/interface mucositis  Key microscopic features that support this diagnosis  Level Feature How it appears in the submitted sections  Epithelium • Hyper-keratinised/parakeratinised surface with mild acanthosis  • Irregular “saw-tooth” shaping of the rete ridges Low-power image shows a slightly thickened surface epithelium with pointed, angulated rete processes.  Basal cell layer • Hydropic (vacuolar) degeneration and loss of the normal basal cell polarity  • Civatte/colloid bodies (eosinophilic apoptotic keratinocytes) High-power view demonstrates numerous basal keratinocytes with pale vacuolated cytoplasm; scattered round, eosinophilic apoptotic bodies are interspersed.  Lamina propria / interface • Dense, continuous “band-like” (lichenoid) infiltrate hugging the epithelial–connective-tissue junction At low magnification a dark, narrow lymphocytic band sits immediately beneath the epithelium, sharply delimiting the interface.  Inflammatory cell composition • Predominantly small lymphocytes (T-cells), with occasional plasma cells or histiocytes; neutrophils are scarce High-power image shows a sheet of monotonous, dark-staining round nuclei with very little cytoplasm, characteristic of mature lymphocytes.  Deeper connective tissue • Relative sparing of the deeper lamina propria; collagen bundles appear normal Deeper half of the field becomes pale-pink, with only scattered chronic inflammatory cells. No deep perivascular or periadnexal aggregate is seen.  Negative clues • No subepithelial clefting/bullae (rules out pemphigoid)  • No follicular/periadnexal inflammation or abundant dermal mucin (argues against discoid lupus)  • No epithelial dysplasia Observed sections lack blister formation, adnexal involvement, and cytologic atypia.  Taken together, the classic lichenoid (interface) infiltrate tightly opposed to an epithelium showing basal-cell vacuolar change and apoptotic bodies is diagnostic of oral lichen planus. |

Oral Manifestations of Systemic Diseases

| **Fig_no** | **Ground truth** | **GPT 4o answer** | **o3 answer** |
| --- | --- | --- | --- |
| 17.5 | Diagnosis: Lipoid Proteinosis; Key Microscopic Features: At medium magnification, perivascular areas exhibit deposition of a layered, acellular substance, and application of the periodic acid–Schiff (PAS) stain accentuates these vascular deposits. | Histopathological Diagnosis:  Scleroderma (Systemic Sclerosis)  Key Microscopic Features Supporting the Diagnosis:  Extensive Collagen Deposition:  Both images show dense, eosinophilic (pink-staining) collagen bundles replacing normal tissue architecture.  The collagen appears thickened and hyalinized, typical of scleroderma's fibrotic changes in connective tissue.  Atrophic or Degenerated Minor Salivary Glands:  The glandular elements, visible in the first image, appear shrunken and surrounded by dense collagen, reflecting glandular atrophy and replacement by fibrous tissue.  Diminished Cellular Infiltrate:  There's a lack of significant inflammatory infiltrate, which is consistent with the chronic fibrotic nature of systemic sclerosis.  Entrapment and Compression of Structures:  Small ducts and residual acini are entrapped within the fibrotic stroma, showing signs of compression and degeneration.  These histologic features align with scleroderma, a connective tissue disease with well-documented oral manifestations, including loss of salivary gland function, mucosal fibrosis, and perivascular changes. | Histopathologic diagnosis (single best):  Invasive well‑differentiated (keratinising) squamous cell carcinoma – desmoplastic (sclerosing) variant.  Key microscopic features visible in the two H‑E fields that support this diagnosis  Feature What is seen in the images Diagnostic importance  1. Infiltrative irregular islands & cords of squamous epithelium Multiple round, oval and elongated epithelial nests are dispersed through the connective‑tissue stroma, entirely detached from the surface epithelium Confirms an invasive epithelial malignancy rather than an in‑situ or reactive process  2. Concentric lamellated keratin (“keratin pearls”) inside many tumour nests Central, brightly eosinophilic whorls with an onion‑skin appearance are present in several islands (best appreciated in the higher‑power image) Hallmark of a well‑differentiated squamous phenotype  3. Individual‑cell keratinisation and intercellular bridges Within the nests the cells show dense cytoplasm and faint prickles; occasional solitary keratinised cells are noted Further evidence of squamous differentiation  4. Marked desmoplastic (sclerotic, collagen‑rich) stroma Tumour islands are surrounded by densely hyalinised, paucicellular pink collagen, producing a “sclerosing” background Characteristic of the desmoplastic variant of SCC and explains the deceptively low cellularity  5. Cytologic atypia of the tumour epithelium Nuclei in the peripheral tumour cells are hyperchromatic and enlarged (though somewhat obscured by the low magnification); architecture is disorderly Confirms malignancy rather than a benign keratinising adnexal lesion  The combination of invasive keratin‑pearl‑forming squamous islands and a pronounced desmoplastic host reaction precisely fits a well‑differentiated (keratinising) squamous cell carcinoma, desmoplastic type occurring in the oral/dermatologic region. |

DEVELOPMENTAL AND CONGENITAL CONDITIONS

| **Fig_no** | **Ground truth** | **GPT 4o answer** | **o3 answer** |
| --- | --- | --- | --- |
| 2.17 | Diagnosis: Ectopic thyroid tissue in the tongue; Key Microscopic Features: A mass of thyroid tissue overlain by oral epithelium, displaying normal follicles filled with colloid. | Based on the provided hematoxylin-and-eosin (H&E) stained histological images at different magnifications, the most likely histopathological diagnosis is:  Thyroglossal Duct Cyst (TGDC)  Key Microscopic Features Justifying the Diagnosis:  Cystic spaces lined by respiratory epithelium (pseudostratified columnar) or stratified squamous epithelium:  In your higher magnification image, some cysts are lined by ciliated epithelium, consistent with respiratory epithelium, which is typical for thyroglossal duct remnants.  Some areas may show squamous metaplasia.  Presence of thyroid follicles:  Scattered within the cyst wall are round to oval follicles filled with colloid — a hallmark of ectopic thyroid tissue.  This is the most diagnostic feature of a TGDC.  Fibrous cyst wall with chronic inflammation:  The stroma surrounding the cysts shows chronic inflammatory cell infiltrate (lymphocytes and plasma cells), which is common in TGDCs due to recurrent infections or irritation.  Fibrosis may also be present in the cyst wall.  Location beneath stratified squamous epithelium resembling skin or oral mucosa:  In the low magnification image, the lesion lies below a surface mucosa resembling that of the oral cavity or skin, consistent with the midline neck location of TGDCs.  Summary:  The combination of thyroid follicles, cystic spaces lined by respiratory/squamous epithelium, and chronic inflammation in a lesion beneath the oral mucosa is highly characteristic of a Thyroglossal Duct Cyst, a common congenital developmental anomaly in the midline neck/oral region. | Histopathological diagnosis  Lymphangioma (cystic / cavernous lymphatic malformation) ‒ a developmental hamartomatous lesion of lymphatic vessels.  Microscopic features that support this diagnosis  Field Key observations Significance  Low-power view • Numerous, variably-sized cystic spaces occupy the sub-epithelial connective tissue.  • Spaces are separated by delicate fibrous septa that contain dense lymphoid aggregates.  • Overlying oral mucosa shows normal stratified squamous epithelium. A lobulated mass of dilated lymphatic channels is the architectural hallmark of a lymphangioma.  Higher magnification • Each cavernous space is lined by a single layer of flattened endothelial cells (no epithelial, myoepithelial or odontogenic lining).  • Lumina contain protein-rich, eosinophilic fluid with scattered lymphocytes and occasional erythrocytes; no true blood column is present.  • Septa are collagenous and show prominent collections of lymphocytes and plasma cells. Endothelial lining plus proteinaceous lymph fluid (rather than blood) distinguishes lymphatic from vascular malformations; the lymphoid tissue in the septa is characteristic.  Additional clues • Absence of keratin lamellae (rules out epidermoid/dermoid cyst).  • Absence of thyroid follicles, salivary acini or muscle bundles within the cyst walls (rules out thyroglossal duct cyst, hamartoma, etc.). Helps exclude other developmental cysts or hamartomas that can appear in the oral/maxillofacial region.  Together, the constellation of large irregular endothelial-lined channels, proteinaceous lymph content, and lymphoid-rich septa beneath intact oral mucosa is diagnostic of a lymphangioma (cystic/cavernous type). |
| 2.18 | Diagnosis: Smooth muscle hamartoma of the tongue; Key Microscopic Features: An unencapsulated collection of spindle-shaped cells within the tongue surrounding salivary glands, composed of spindle-shaped smooth muscle cells with abundant pale eosinophilic cytoplasm. | Based on the provided hematoxylin-and-eosin stained histological images at low and high magnification, the single most likely histopathological diagnosis is:  Congenital Epulis (also known as Congenital Granular Cell Tumor)  Key Microscopic Features Supporting the Diagnosis:  Low Magnification (First Image):  Polypoid/Exophytic growth pattern: The lesion appears to be well-circumscribed and elevated from the mucosal surface.  Overlying stratified squamous epithelium: The surface epithelium is relatively unremarkable and not ulcerated, which is typical.  Subepithelial proliferation: The lesion consists of a lobular proliferation in the subepithelial connective tissue.  High Magnification (Second Image):  Granular eosinophilic cytoplasm: The tumor cells have abundant granular eosinophilic cytoplasm, a hallmark feature.  Small, uniform nuclei: The nuclei are round to oval, bland, and located centrally.  Lack of mitotic activity or atypia: The cells show no pleomorphism or increased mitotic figures, suggesting a benign process.  No pseudoepitheliomatous hyperplasia: This differentiates congenital epulis from adult granular cell tumor (which often shows this feature).  Contextual Relevance:  Congenital epulis is a rare benign lesion seen almost exclusively in newborns, typically on the alveolar ridge. It is part of the developmental and congenital conditions group in oral and maxillofacial pathology, aligning with the user's categorization. | Histopathological diagnosis  Solitary neurofibroma of the oral mucosa.  Key microscopic features supporting this diagnosis  Low-power findings High-power findings  • Unencapsulated, poorly demarcated proliferation expanding the lamina propria and submucosa beneath intact, slightly hyperplastic stratified squamous epithelium. • Interlacing bundles and haphazard sheets of spindle-shaped cells with characteristic wavy/serpentine nuclei.  • Lesion infiltrates around and between native structures (fat lobules/minor salivary tissue are partially entrapped), a typical pattern for neurofibroma rather than a well-circumscribed fibroma. • Delicate, eosinophilic fibrillary cytoplasm and pale myxoid-collagenous matrix between the cells, giving a loose, “shredded carrot” appearance.  • Overall architecture lacks a true capsule (distinguishing it from schwannoma) and shows diffuse growth. • Scattered small vessels and occasional mast cells (not obvious in every field but commonly present) without significant atypia or mitotic activity, consistent with a benign neural lesion.  The combination of an unencapsulated, infiltrative spindle-cell proliferation with wavy nuclei, delicate collagen / myxoid stroma, and entrapment of surrounding tissues is diagnostic of neurofibroma in the developmental–congenital category of oral and maxillofacial pathology. |
| 2.19 | Diagnosis: Ectopic brain tissue; Key Microscopic Features: Pale epithelioid cells situated within the tongue’s lamina propria above skeletal muscle, accompanied by a syncytium of astrocytes. | Based on the provided hematoxylin and eosin (H&E) stained histological images at different magnifications, the single most likely histopathological diagnosis is:  Central Giant Cell Granuloma (CGCG)  Key Microscopic Features Justifying the Diagnosis:  Multinucleated Giant Cells:  Seen prominently in the second (higher magnification) image.  These multinucleated cells are dispersed in a background of ovoid to spindle-shaped mononuclear stromal cells, which is characteristic of CGCG.  Vascular and Hemorrhagic Stroma:  The stroma is highly vascular, with numerous capillaries and often areas of hemorrhage. These features are evident particularly in the lower half of the first image.  Fibrous Connective Tissue Background:  The lesion is set in a fibroblastic stroma, with scattered hemosiderin-laden macrophages and possibly areas of osteoid or reactive bone formation (less clear in these images but often seen in CGCG).  Overlying Epithelium:  The overlying surface shows stratified squamous epithelium, which appears unremarkable and non-dysplastic—supporting the intraosseous and non-epithelial origin of the lesion.  Non-encapsulated, Locally Infiltrative Pattern:  The lesion does not show a capsule and blends into the surrounding tissue, a common pattern for CGCG.  Contextual Consideration:  CGCG is a benign but potentially aggressive intraosseous lesion of the jaws, more commonly seen in the anterior mandible of young individuals, and is classified under Developmental and Congenital Conditions in oral and maxillofacial pathology. | Histopathological diagnosis  → Solitary neurofibroma (benign peripheral-nerve sheath tumour)  Key microscopic features that support this diagnosis  Level of magnification What is seen How it points to neurofibroma  Scanning/low power (first image) • Diffuse, poorly circumscribed, non-encapsulated lesion expanding the lamina propria and infiltrating between skeletal-muscle bundles in the deep connective tissue.  • Overlying stratified squamous epithelium is intact but shows elongated, slender rete ridges that “dip” around the lesion. Neurofibromas typically blend imperceptibly with adjacent tissues rather than forming a capsule; their mass effect can secondarily stretch epithelium.  Intermediate–high power (second image) • Proliferation of spindle to stellate cells arranged in short, interlacing fascicles and whorls, set in a delicate, fibrillar (“shredded-carrot”) collagenous / myxoid stroma.  • Wavy, serpentine nuclei with tapered ends; cytoplasm scant, ill defined.  • Lesional stroma shows scattered small blood vessels and occasional mast cells (pink‐purple granulated cells).  • No nuclear palisading or Verocay bodies (helps exclude schwannoma); no significant atypia or mitotic activity. The combination of unencapsulated growth, mixture of Schwann-cell-type spindle cells and fibroblastic cells with wavy nuclei, plus the characteristic loose collagen/myxoid background and mast cells, is classic for a neurofibroma.  Summary of the pattern: an unencapsulated spindle-cell proliferation with wavy nuclei, delicate collagen, and mast cells that infiltrates surrounding connective tissue and muscle—while leaving the surface epithelium intact—matches a solitary neurofibroma arising in the oral soft tissues. |
| 2.22 | Diagnosis: Lymphoepithelial cyst of the oral cavity; Key Microscopic Features: A keratin-filled cyst partially bordered by lymphoid tissue, lined by parakeratotic stratified squamous epithelium with ganglion-like cells encircled by neural tissue characteristic of a subgemmal neurogenous plaque. | Based on the two hematoxylin-and-eosin stained histological images of the same lesion taken at different magnifications, the most consistent histopathological diagnosis is:  Odontogenic Keratocyst (OKC)  Key Microscopic Features Supporting the Diagnosis:  Cystic Architecture:  The low-power image shows a unilocular cystic lesion with a well-defined fibrous capsule. This is consistent with the architecture of an odontogenic keratocyst.  Parakeratinized Stratified Squamous Epithelium:  The high-power image clearly shows a uniform epithelial lining that is parakeratinized (retaining nuclei in the keratin layer) with a corrugated surface — a hallmark feature of OKC.  Palisaded Basal Layer:  The basal layer of the epithelium displays nuclear palisading (columnar and aligned nuclei), another classic feature of OKC.  Thin, Uniform Epithelial Lining:  The epithelial lining is thin (typically 6–8 cells thick) and consistent in thickness, another key diagnostic trait.  Lack of Inflammatory Infiltrate (in most cases):  The fibrous capsule appears relatively free of chronic inflammatory infiltrate, which is commonly seen in non-inflamed OKCs.  Contextual Relevance:  Odontogenic keratocysts are developmental cysts that arise from the dental lamina and are part of the developmental and congenital conditions group in oral and maxillofacial pathology. They are known for their aggressive behavior and high recurrence rate. | Histopathological diagnosis  → Cervical (branchial‐cleft-type) lymphoepithelial cyst  Key microscopic features in the two H-E sections that lead to this diagnosis  # Feature seen Diagnostic significance  1 Well-defined cystic space beneath the surface oral mucosa, partly collapsed in the low-power view Confirms a cystic rather than solid developmental lesion  2 Lining epithelium is stratified squamous, 4–8 cells thick, generally smooth, occasionally parakeratinised (high-power inset) Typical epithelial lining of a branchial-cleft/lymphoepithelial cyst  3 Sharp cleft (retraction artefact) between epithelium and underlying tissue in several foci Common in thin, non-rete-pegged cyst linings of this entity  4 Dense lymphoid tissue forming prominent follicles with germinal centres immediately beneath the lining (dark-purple lobulated areas in the low-power slide) The defining hallmark—epithelium intimately associated with organised lymphoid tissue  5 Lumen and deeper folds show flakes/strands of eosinophilic keratin debris (seen as pale-pink material with occasional slit-like spaces) Keratin desquamation is expected in a squamous-lined cyst that is not inflamed  6 Surrounding fibrous capsule with scattered minor salivary acini and vessels but no atypia Supports a developmental cyst; excludes neoplasm  The coexistence of a thin squamous lining and a robust lymphoid follicular wall is diagnostic of a lymphoepithelial (branchial-cleft–type) cyst, a classic developmental/congenital lesion in the head-and-neck region. |
| 2.27 | Diagnosis: Epidermoid cyst; Key Microscopic Features: A cyst lined by uniformly thin stratified squamous epithelium filled with keratin, featuring an orthokeratinized stratified squamous epithelial lining that is uniformly thin and exhibits a prominent granular cell layer. | Histopathological Diagnosis:  Dentigerous Cyst  Key Microscopic Features Supporting the Diagnosis:  Cystic Structure:  The low-power image (first image) reveals a well-circumscribed, unilocular cystic lesion with a fibrous connective tissue wall enclosing a lumen, which is characteristic of odontogenic cysts.  Non-keratinized Stratified Squamous Epithelium:  In the high-power view (second image), the cyst is lined by a uniform, thin (2–4 cell layers thick), non-keratinized stratified squamous epithelium, a hallmark of dentigerous cysts.  The basal cell layer appears flat to cuboidal and lacks palisading, and there is no surface keratinization.  Fibrous Connective Tissue Wall:  The connective tissue wall underlying the epithelium is loosely collagenized and vascular, often with some chronic inflammatory infiltrate (though not prominent in this example).  Association with Developing Tooth (Implied Context):  While not seen in these histologic images directly, dentigerous cysts are typically associated radiographically and clinically with the crown of an unerupted or impacted tooth — usually third molars or maxillary canines — supporting the clinical diagnosis in developmental lesions. | Histopathological diagnosis  Odontogenic keratocyst (parakeratinised variant)  Key microscopic features that support this diagnosis  Feature (seen in the submitted H-E sections) Diagnostic significance  Uniformly thin epithelial lining, only ~6–8 cell layers thick Developmental odontogenic cysts may have variable thickness, but OKC classically shows a very thin, even lining.  Luminal surface covered by a corrugated (wavy) layer of parakeratin The corrugated parakeratin layer is the hallmark of the parakeratinised OKC and helps distinguish it from orthokeratinised odontogenic cysts or dentigerous/radicular cysts.  Prominent, palisaded basal layer: nuclei are hyperchromatic, elongated and arranged in a “picket-fence”/“tombstone” fashion, with reverse polarity Palisaded basal cells are characteristic of OKC and are rarely as striking in other odontogenic cysts.  Flat epithelial–connective-tissue interface with little or no rete-ridge formation Unlike inflammatory cysts, OKC epithelium does not interdigitate with the capsule, so the epithelial front is flat and sharply demarcated.  Lumen and focal whorls of loose keratin shreds (visible on the low-power image) Supports a keratin-producing cyst lining rather than a mucous or serous cyst.  Fibrous cyst wall generally thin, with occasional satellite/daughter cysts (suggested by small epithelial islands in the wall on the low-power view) Satellite cysts are a well-known feature of OKC and contribute to its high recurrence rate.  The constellation of a corrugated parakeratin surface, thin uniform lining, and palisaded basal cells is diagnostic for a parakeratinised odontogenic keratocyst. |
| 2.28 | Diagnosis: Dermoid cyst; Key Microscopic Features: A cyst wall containing pilosebaceous units, lined by uniformly thin orthokeratinized stratified squamous epithelium with a granular layer and the presence of pilosebaceous units within the wall. | Based on the provided hematoxylin and eosin (H&E) stained histological images, taken at different magnifications, the single most likely histopathological diagnosis is:  Dermoid Cyst  Key Microscopic Features Supporting the Diagnosis:  Cystic Architecture:  Both images reveal a cystic cavity lined by stratified squamous epithelium, resembling skin, which is a hallmark of dermoid cysts.  Presence of Skin Adnexal Structures:  In the second, higher magnification image, sebaceous glands and hair follicle-like structures are clearly visible in the cyst wall. These adnexal structures are diagnostic and distinguish a dermoid cyst from an epidermoid cyst.  Keratinization and Cyst Lumen:  The cyst lining shows keratinization, and the lumen appears to contain keratinaceous debris, commonly seen in dermoid cysts.  Fibrous Connective Tissue Wall:  The cyst is surrounded by dense connective tissue, consistent with the fibrous capsule often seen in dermoid cysts.  Additional Notes:  Dermoid cysts are considered developmental lesions and are included in the category of developmental cysts of ectodermal origin.  In the oral and maxillofacial region, they commonly occur in the midline floor of the mouth, though this information is not evident from the image alone. | Histopathological diagnosis  → Dermoid cyst (developmental “true” dermoid cyst of the oral cavity).  Key microscopic features supporting this diagnosis  Microscopic finding How it appears in the provided slides  Cystic space lined by orthokeratinised stratified squamous epithelium with a well-formed granular layer The lumen is bordered by a thick squamous lining whose superficial cells show keratohyaline granules; the lumen contains flaky keratin (best appreciated in the higher-power image).  Abundant keratin debris in the lumen Central eosinophilic, anucleate material is present, characteristic of orthokeratin accumulation.  Dermal/skin appendages in the cyst wall – sebaceous glands ± hair-follicle structures Multiple lobules of pale, vacuolated sebaceous cells (“grapelike” clusters) are embedded in the fibrous wall on both sides of the cyst; a few follicular structures are also suggested. Their presence distinguishes a dermoid cyst from a simple epidermoid cyst.  Dense fibrous connective-tissue capsule without inflammation or dysplasia The cyst is surrounded by a collagenous capsule with scattered fibroblasts and minimal chronic inflammatory infiltrate.  Overlying normal oral mucosa At low power, normal stratified squamous epithelium of the oral mucosa is seen superficial to the lesion, confirming its submucosal/deep location typical of developmental cysts.  Together, the combination of an orthokeratinised epithelial lining plus definite adnexal (sebaceous) structures in the cyst wall is diagnostic of a dermoid cyst as opposed to an epidermoid or teratoid variant. |
| 2.29 | Diagnosis: Dermoid tumor (hairy polyp); Key Microscopic Features: A nodule covered by epidermis containing pilosebaceous units and abundant mature adipose tissue, with pilosebaceous units, eccrine glands, and smooth muscle present. | Based on the hematoxylin and eosin (H&E) stained histopathological images provided, the single most likely diagnosis is:  Lymphangioma  Key Microscopic Features Justifying the Diagnosis:  Multiple Dilated Lymphatic Channels:  The low-power image shows numerous large, thin-walled, endothelium-lined vascular spaces throughout the connective tissue.  These spaces are irregular and variably sized, some appearing almost cystic, and they are more prominent toward the center.  Endothelial Lining:  The high-power image demonstrates these spaces lined by a single layer of flattened endothelial cells, typical of lymphatic vessels.  Eosinophilic Proteinaceous Fluid in Lumina:  Some of the spaces contain pale eosinophilic material, likely lymph, consistent with lymphatic origin.  Surrounding Loose Connective Tissue:  The stroma appears loose and collagenous, consistent with a lesion involving submucosal tissue.  No Evidence of Atypia or Invasive Behavior:  No cellular atypia or mitotic activity is observed, favoring a benign congenital lesion.  Background Information:  Lymphangioma is a benign hamartomatous lesion of lymphatic vessels, often present at birth or noticed in early childhood. In the oral cavity, it commonly affects the tongue, lips, and buccal mucosa. Histologically, the lesion is characterized by large, dilated lymphatic channels in the subepithelial tissue, which matches the histopathologic features seen here. | Histopathological diagnosis  Dermoid cyst (skin-appendage type) of the oral cavity  Key microscopic features visible in the two fields  # Feature How it appears in the images Diagnostic relevance  1 Keratinising stratified squamous epithelial lining A thin bluish‐purple band circumscribing the lesion beneath the oral mucosa (best appreciated in the low-power image) Indicates an epidermoid-type cyst wall  2 Dermal adnexal structures inside the cyst wall Multiple lobules made up of: • Sebaceous glands – clusters of pale, foamy cells with centrally placed nuclei  • Hair-follicle–like structures – round/oval epithelial nests with central keratin, surrounded by an outer root-sheath–like layer Presence of cutaneous appendages upgrades an epidermoid cyst to a dermoid cyst  3 Mature adipose tissue within the wall Abundant empty, round vacuoles separated by delicate fibrous septa occupy most of the central field in both magnifications Adipocytes are a common mesodermal component of dermoid cyst walls and contribute to the lobulated appearance on low power  4 Dense collagenous connective tissue Coarse, eosinophilic, wavy collagen bundles form the background stroma surrounding adnexal islands (high-power image) Typical supportive wall of dermoid cyst; differentiates it from simple surface epithelium-lined cysts  5 Well-circumscribed, domed architecture The low-power view shows a round, sharply demarcated lesion sitting immediately beneath the surface mucosa Favors a benign developmental cyst rather than an infiltrative neoplasm  The combination of an epidermoid cyst lining plus clearly developed sebaceous glands/hair-follicle structures and supporting fibrofatty tissue is pathognomonic for a dermoid cyst in the oral and maxillofacial region. |
| 2.33 | Diagnosis: Nasolabial cyst; Key Microscopic Features: A cyst lined by uniformly thin epithelium that incorporates muscle within its wall and mucous cells, with areas of ciliated columnar and cuboidal epithelium containing mucous cells. | Histopathological Diagnosis:  Papillary cystadenoma lymphomatosum (Warthin tumor)  Key Microscopic Features Supporting the Diagnosis:  The provided hematoxylin and eosin (H&E) stained images at different magnifications collectively show classic histopathological characteristics of a Warthin tumor, a benign salivary gland neoplasm, commonly affecting the parotid gland.  1. Papillary and Cystic Architecture (Seen prominently in the low-power image):  Numerous papillary projections into cystic spaces.  The cystic spaces are lined by a bilayered epithelium and contain eosinophilic material or proteinaceous debris.  2. Bilayered Epithelium (More evident at medium and high power):  Inner luminal layer: Tall columnar cells with eosinophilic cytoplasm (oncocytic appearance).  Outer basal layer: Cuboidal or polygonal cells.  This dual-layered lining is very characteristic of Warthin tumor.  3. Dense Lymphoid Stroma with Germinal Centers:  The connective tissue stroma beneath the epithelium shows prominent lymphoid aggregates, often with germinal centers, a hallmark of Warthin tumor.  This lymphoid component gives rise to the alternative name: papillary cystadenoma lymphomatosum.  4. Oncocytic Change:  Cells lining the cystic spaces have abundant granular eosinophilic cytoplasm, consistent with oncocytic metaplasia, indicative of abundant mitochondria.  Conclusion:  The combination of cystic and papillary architecture, bilayered oncocytic epithelium, and dense lymphoid stroma is diagnostic of Warthin tumor. This lesion is almost exclusively seen in the parotid gland and typically affects older adults, often with a history of smoking. | Histopathological diagnosis  Nasolabial (naso-alveolar / Klestadt) cyst – a developmental, non-odontogenic cyst of the nasal‐alar region.  Key microscopic features visible in the three H-E sections  # Feature in the slide Why it points to a nasolabial cyst  1 Cystic space lined by a thin, respiratory-type epithelium – 2-3 layers of tall columnar cells with basally placed nuclei, apical mucin/blebs and scattered goblet cells (high-power image). The nasolabial cyst arises from embryonic remnants of the nasolacrimal duct; its lining is almost always pseudostratified ciliated columnar epithelium with occasional goblet cells.  2 Long, branching papillary or arborising folds of the lining into the lumen (best seen on the low- and medium-power pictures). Papillary infoldings are very characteristic for this lesion and are only rarely seen in other developmental cysts of the region.  3 Loose, oedematous fibro-vascular connective-tissue wall containing many thin-walled, dilated blood vessels and areas of recent haemorrhage. A vascular, slightly inflamed wall is typical; it lacks the prominent lymphoid tissue of branchial cysts and the large neuro-vascular bundles of a nasopalatine duct cyst.  4 Foci of minor sero-mucous salivary gland acini in the deeper wall (visible at the edge of the low-power field). Minor salivary glands are often entrapped in the soft-tissue wall of a nasolabial cyst because the lesion sits entirely in the soft tissue of the upper lip/nasal vestibule.  5 Absence of bone, cartilage or keratinised squamous epithelium. Helps exclude a nasopalatine duct cyst (usually within bone and may show cartilage) and epidermoid / dermoid cysts (keratinising squamous lining).  Taken together, the combination of a soft-tissue cyst with respiratory-type epithelium, exuberant papillary infoldings, a vascular fibrous wall and adjacent minor glands is diagnostic of a nasolabial cyst. |
| 2.34 | Diagnosis: Heterotopic gastrointestinal cyst; Key Microscopic Features: A cyst lined by uniformly thin squamous epithelium containing mucous cells, showing transitions between stratified squamous and mucous cells resembling gastric mucosa, and frequently exhibiting ciliated epithelium. | Based on the provided hematoxylin and eosin (H&E) stained histopathology images of the same lesion at varying magnifications, the single most likely histopathological diagnosis is:  Glandular Odontogenic Cyst (GOC)  Key Microscopic Features Supporting the Diagnosis:  Epithelial Lining with Variable Thickness:  The cystic lining is non-uniform in thickness, typical of GOC. Some areas show a thin, cuboidal to columnar epithelium, while others are thicker with more stratification.  Hobnail (Surface Columnar) Cells:  In the higher magnification images, cells at the surface of the lining epithelium show eosinophilic cytoplasm and appear columnar or cuboidal with apical nuclei – these are the classic “hobnail” cells seen in GOC.  Mucous (Goblet) Cells:  Mucin-producing goblet cells are interspersed within the lining epithelium, which is a hallmark feature of GOC and not commonly seen in other odontogenic cysts.  Intraepithelial Microcysts or Gland-like Spaces:  Multiple small, cyst-like or duct-like spaces are visible within the epithelial lining, consistent with the “glandular” or “duct-like” differentiation.  Clear (Pale) Cells:  Presence of clear cells scattered in the epithelium is noted, another feature supporting GOC.  Flat Interface with Connective Tissue Wall:  The epithelium connects smoothly with the underlying connective tissue wall without significant inflammation or dysplasia.  Summary:  The combination of hobnail cells, mucous cells, intraepithelial microcysts, and a non-uniform epithelial lining with duct-like spaces is pathognomonic for Glandular Odontogenic Cyst. These features distinguish it from other cysts like the lateral periodontal cyst or mucoepidermoid carcinoma. | Histopathological diagnosis (single best):  Nasolabial (naso-alveolar) cyst  Key microscopic features visible in the three photomicrographs  Feature Where it is seen Significance for the diagnosis  1. Large unilocular cystic space with smooth-to-papillary folds Low-power image (first slide) Confirms a true cyst rather than a solid tumour; the broad, smooth lumen with occasional projecting folds is typical of nasolabial cysts.  2. Lining of pseudostratified, ciliated columnar “respiratory-type” epithelium with plentiful goblet (mucous) cells Medium- and high-power images (second & third slides) The characteristic respiratory lining is the single most useful clue. Goblet cells are abundant and cilia can be appreciated on the apical surface – classic for fissural cysts of the naso-alveolar region and uncommon in odontogenic cysts.  3. Variable epithelial thickness (2 – 8 cell layers) without keratinisation or rete ridges High-power view (third slide) Helps exclude keratinising odontogenic cysts and epidermoid/dermoid cysts.  4. Fibro-vascular cyst wall with scattered chronic inflammatory cells and numerous thin-walled vessels Medium-power image (second slide) Chronic but mild inflammation is common because the cyst opens into the nasal vestibule; richly vascular connective tissue supports respiratory mucosa.  5. Skeletal muscle bundles and minor salivary gland lobules in the supporting soft tissue; no bone present Lower half of the medium-power image (cross-sectioned pink/red fibres) Placement within the soft tissues of the upper lip/alar base (outside bone) is typical for a nasolabial cyst and contrasts with nasopalatine duct cysts, which lie within the incisive canal and often show bone or neurovascular bundles in their wall.  Taken together, the respiratory-type lining with goblet cells, the soft-tissue location evidenced by skeletal muscle in the wall, and the absence of odontogenic or bony elements point squarely to a nasolabial (naso-alveolar) cyst, a developmental, non-odontogenic fissural cyst of the nasolabial fold region. |

BACTERIAL,VIRAL, FUNGAL,AND OTHER INFECTIOUS CONDITIONS

| **Fig_no** | **Ground truth** | **GPT 4o answer** | **o3 answer** |
| --- | --- | --- | --- |
| 4.6 | **Diagnosis:** Infection by herpes simplex virus (HSV); **Key Microscopic Features:** Ulceration on the dorsal surface of the tongue due to HSV, with multinucleated epithelial cells at the ulcer margin exhibiting indistinct Cowdry inclusions and nuclear molding. | Based on the provided hematoxylin and eosin (H&E) stained histopathological images taken at different magnifications, here is the analysis:  Single Most Likely Histopathological Diagnosis:  Mucormycosis (Zygomycosis)  Key Microscopic Features Justifying the Diagnosis:  Low Magnification (First Image):  Ulcerated stratified squamous epithelium with necrosis and underlying granulation tissue.  Dense inflammatory infiltrate, predominantly neutrophilic, indicating acute inflammation.  Areas of necrosis with hemorrhage, which are typical of tissue destruction by angioinvasive fungi.  High Magnification (Second Image):  Presence of broad, non-septate to sparsely septate hyphae with irregular branching at right angles (90°), which is characteristic of Mucor species.  The fungal elements are seen invading blood vessels (angioinvasion), leading to thrombosis and tissue necrosis — a hallmark of mucormycosis.  Surrounding necrotic debris and intense acute inflammation, consistent with aggressive fungal invasion.  Conclusion:  The combination of broad, ribbon-like, non-septate hyphae, right-angle branching, and vascular invasion seen in a necrotic and inflamed background strongly supports a diagnosis of mucormycosis, a life-threatening fungal infection common in immunocompromised individuals, especially those with uncontrolled diabetes or immunosuppression. | Histopathological diagnosis (single most likely):  Rhinosporidiosis (infection by Rhinosporidium seeberi)  Key microscopic features that support this diagnosis  Field Observation Diagnostic implication  Epithelium (low-power image) Papilliform / pseudoepitheliomatous hyperplasia with focal surface ulceration and fibrinous exudate Characteristic reaction pattern of mucosa overlying rhinosporidial polyps  Subepithelial stroma (both magnifications) Dense mixed inflammatory infiltrate (neutrophils, lymphocytes, plasma cells) with scattered micro-abscesses Typical host response to rupturing sporangia  Organisms (high-power image) Numerous round-to-oval, thick-walled sporangia of greatly variable size (maturing trophocytes 10–30 µm → fully developed sporangia ≥100 µm) Size spectrum is classic for the developmental stages of R. seeberi  Each mature sporangium contains multiple basophilic endospores; some are collapsed or empty after rupture, producing “ghost” cystic spaces Endospore-filled, thick-walled sporangia are pathognomonic  Ruptured sporangia surrounded by neutrophils and occasional multinucleated giant cells Rupture and release of endospores elicit acute and granulomatous inflammation  These large, thick-walled, endospore-filled sporangia set rhinosporidiosis apart from other infectious lesions encountered in the oral/maxillofacial region (e.g., the narrower hyphae of mucormycosis/aspergillosis, the smaller intracellular yeasts of histoplasmosis, or the broad-based budding yeast of blastomycosis).  Hence, the constellation of pseudoepitheliomatous mucosal hyperplasia, a mixed chronic–suppurative stroma, and myriad variably sized, thick-walled sporangia packed with endospores unequivocally supports rhinosporidiosis. |
| 4.8 | **Diagnosis:** Oral hairy leukoplakia; **Key Microscopic Features:** The oral epithelium reveals shaggy parakeratosis, acanthosis, and keratinocyte swelling, accompanied by candidal colonization without spongiotic pustules, and keratinocytes show peripheral chromatin condensation encircling indistinct central Cowdry inclusions. | Based on the hematoxylin and eosin (H&E) stained histopathology images you provided, the single most likely histopathological diagnosis is:  Condyloma acuminatum (HPV-induced squamous epithelial proliferation)  This lesion belongs to the viral infectious conditions group, and is most commonly associated with low-risk human papillomavirus (HPV) types 6 and 11.  Key Microscopic Features Justifying the Diagnosis:  Papillomatous and Acanthotic Epithelium:  There is marked acanthosis (thickening of the spinous layer) and papillary projections, which are characteristic of HPV-associated epithelial lesions.  Koilocytosis:  Seen particularly in the superficial layers of the epithelium, these cells show:  Enlarged, hyperchromatic nuclei  Clear perinuclear halos  Perinuclear vacuolization  These changes are hallmark features of HPV cytopathic effect.  Parakeratosis and Hypergranulosis:  Mild parakeratin layering is evident on the surface.  Some foci may show thickened granular layers.  Minimal Inflammation:  There is no significant inflammatory infiltrate or ulceration, which helps differentiate from infectious ulcerative lesions or fungal infections.  No Evidence of Fungal Organisms or Bacterial Colonies:  No hyphal forms (as in candidiasis) or bacterial colonies (as in actinomycosis or syphilitic mucositis) are identified.  This lesion is consistent with condyloma acuminatum, a benign but transmissible lesion commonly found on the oral mucosa, anogenital region, or perioral areas, often due to oral-genital contact in HPV-infected individuals. | Most likely histopathological diagnosis  Verruca vulgaris (common wart) of the oral mucosa – an HPV-associated viral infection  Key microscopic features in the two H-E sections that support this diagnosis  # Feature seen Diagnostic significance  1 Pronounced hyper-orthokeratosis / parakeratosis forming a thick, wavy surface coat (best appreciated in the low-power field). Warts characteristically build up a thick keratin layer.  2 Acanthosis with bulbous, elongated rete ridges that “cup” toward the centre of the lesion and sometimes show internal whorling of keratin (“squamous eddies”). The downward-growing, converging rete pegs are typical of verruca vulgaris and help distinguish it from the broader, blunted ridges of condyloma acuminatum.  3 Koilocytosis in the upper spinous / granular layers – rounded cells with enlarged, wrinkled, hyperchromatic nuclei surrounded by a clear perinuclear halo (clearly visible in the high-power image). Classical viral cytopathic effect of HPV infection.  4 Surface papillomatosis (short papillary projections with a vascular connective-tissue core) although sectioning partially truncates the papillae. Gives the wart its exophytic, verruciform architecture.  5 Absence of epithelial dysplasia or true invasion – despite the pseudo-epitheliomatous hyperplasia, the basement membrane remains intact and there is no stromal desmoplasia. Supports a benign HPV-induced proliferation rather than carcinoma.  Taken together, the combination of thick keratin, acanthotic epithelium with cupped rete ridges, abundant koilocytes, and papillary surface architecture is diagnostic of verruca vulgaris, an HPV-driven viral lesion of the “Bacterial, Viral, Fungal and Other Infectious Conditions” category in oral & maxillofacial pathology. |
| 4.9 | **Diagnosis:** Oral hairy leukoplakia; **Key Microscopic Features:** The oral mucosa displays parakeratosis with a subjacent band of edematous, ballooned cells; keratinocytes exhibit peripheral chromatin condensation, coarse chromatin clumping, and indistinct Cowdry inclusions; in situ hybridization demonstrates nuclear positivity for Epstein–Barr virus–encoded RNA. | Histopathological Analysis of Provided H&E Images  Single Most Likely Diagnosis:  Oral Mucormycosis (Zygomycosis)  Key Microscopic Features Justifying the Diagnosis:  Image 1 (Low magnification):  Ulcerated epithelium with necrosis and underlying dense inflammatory infiltrate.  Marked tissue necrosis extending into submucosa and possibly muscle layer.  Suggestive of aggressive and angioinvasive infection.  Image 2 (High magnification):  Presence of broad, non-septate or pauci-septate hyphae with right-angle (90°) branching, seen in necrotic tissue.  These are classic morphological features of Mucorales fungi, differentiating them from septate hyphae of Aspergillus (which branch at acute angles).  Image 3 (Intermediate magnification):  Angioinvasion: fungal hyphae invading blood vessels, with resultant vascular occlusion and tissue ischemia.  This angioinvasion is characteristic and often leads to extensive necrosis.  Supporting Diagnostic Points:  Mucormycosis is typically seen in immunocompromised patients, particularly with diabetes mellitus or those receiving corticosteroids.  The extensive necrosis and broad, ribbon-like fungal hyphae with sparse septations and wide-angle branching strongly support Mucor species.  Differential diagnoses like Aspergillosis or Candidiasis are ruled out based on fungal morphology (septation and branching angles). | Histopathological diagnosis  ► Cryptococcosis (infection by Cryptococcus spp.)  Key microscopic features in the three fields that support this diagnosis  Field What is seen Why it points to Cryptococcus  Low‑power overview (image 1) •  Surface stratified squamous epithelium shows marked pseudo‑epitheliomatous (papillary/irregular) hyperplasia with downward‑growing rete ridges.  •  Underlying lamina propria is pale and only lightly inflamed. This pattern is typical of mucosal reactions to a deep fungal infection in which the bulk of organisms lie in the submucosa rather than within the epithelium.  Intermediate magnification (image 2) •  Numerous round‑to‑oval bodies, 4‑10 µm in diameter, scattered through a pale eosinophilic (“gelatinous”) stroma.  •  Each body has a central basophilic yeast cell that has shrunk away from the surrounding tissue, leaving a wide unstained clear halo. The clear halo is the polysaccharide capsule that characterises Cryptococcus species. On H‑E it fails to take up dye, producing the classic “soap‑bubble” appearance.  High‑power (image 3) •  The encapsulated yeasts are seen in greater detail: thick, refractile capsule, central basophilic nucleus; many exhibit narrow‑based budding.  •  Inflammatory response is relatively sparse; no hyphae, septae or endosporulating sporangia are present. Size, encapsulation, narrow‑based budding and paucity of associated inflammation together are diagnostic for Cryptococcus and help exclude other fungal or protozoal organisms (e.g., Candida, Blastomyces, Rhinosporidium).  Taken together, the combination of pseudoepitheliomatous surface hyperplasia and a submucosal proliferation of numerous encapsulated, “haloed” yeast cells with narrow‑based budding is pathognomonic for cryptococcal infection of the oral mucosa. |
| 4.13 | **Diagnosis:** Infection with cytomegalovirus (CMV); **Key Microscopic Features:** The oral mucosa shows ulceration at its base, with cells exhibiting prominent “owl’s eye” nuclei characterized by large size and red nucleoli, as well as mildly granular amphophilic cytoplasm; endothelial cells are also infected by CMV. | Based on the analysis of the provided hematoxylin and eosin (H&E) stained histopathological images of an oral lesion at low and high magnifications, the single most likely histopathological diagnosis is:  Herpes Simplex Virus (HSV) Infection  Key Microscopic Features Supporting the Diagnosis:  Acantholysis and Ulceration (Low Power View):  The first image shows ulcerated stratified squamous epithelium with pseudoepitheliomatous hyperplasia.  There is necrosis of the epithelial surface, and the underlying connective tissue appears inflamed and granulated.  Multinucleated Giant Cells with Viral Cytopathic Effect (High Power Views):  Prominent multinucleated epithelial cells are visible with:  Ground-glass chromatin  Nuclear molding (nuclei of adjacent cells appear to indent each other)  Margination of nuclear chromatin (peripheral distribution)  These changes are classical for HSV cytopathic effect.  Occasional Cowdry type A intranuclear inclusions may be seen—eosinophilic inclusions surrounded by a clear halo.  Dense Mixed Inflammatory Infiltrate:  Subepithelial connective tissue contains a dense inflammatory infiltrate, including lymphocytes, plasma cells, and occasional neutrophils.  Evidence of vascular congestion is also visible, indicating active inflammation.  Differential Diagnoses Considered but Less Likely:  Cytomegalovirus (CMV): Typically involves endothelial and stromal cells with large “owl’s eye” intranuclear inclusions.  Fungal infections (e.g., Histoplasmosis): Would show granulomas or identifiable fungal organisms on special stains.  Bacterial infections (e.g., Syphilis): Usually show plasma cell-rich infiltrates with vascular changes, but not viral cytopathic effect.  Varicella-Zoster Virus (VZV): Can look similar but clinical correlation (dermatomal pattern) helps distinguish. | Histopathologic diagnosis – Oral/gingival SARCOIDOSIS (non-caseating granulomatous inflammation)  Key microscopic features that point to sarcoidosis  Field What is seen in the photographs Why it matters  Granuloma architecture Discrete, tight granulomas made up of epithelioid histiocytes that coalesce through most of the lamina propria; they lack a necrotic (caseating or suppurative) centre. Well-formed non-caseating granulomas are the sine-qua-non of sarcoidosis and help exclude tuberculosis, cat-scratch disease and deep fungal infections, all of which usually show necrosis or neutrophilic micro-abscesses.  Giant-cell component Numerous Langhans-type and foreign-body–type multinucleated giant cells scattered through the granulomas. Sarcoid granulomas almost always contain giant cells; their presence with no organisms on H-E further supports a “clean” (sarcoid-type) granuloma.  Intra-cytoplasmic inclusions in giant cells Eosinophilic, concentrically laminated bodies (Schaumann bodies) and occasional faint stellate inclusions (asteroid bodies) are visible inside several giant cells (best appreciated in the high-power images where the laminated “bull’s-eye” structures are seen). Although not pathognomonic, Schaumann and asteroid bodies are classic cytologic hallmarks of sarcoidosis and are rarely encountered together in other oral granulomatous diseases.  Overlying surface epithelium Marked pseudo-epitheliomatous hyperplasia with elongated rete ridges (low-power image). Reactive epithelial thickening commonly overlies sarcoidal granulomas in the oral cavity and may mimic a verrucous or dysplastic process unless the sub-epithelial changes are appreciated.  Inflammatory milieu A tight cuff of small lymphocytes surrounds each granuloma; plasma cells and neutrophils are scant to absent; no fungi, bacteria or parasites are seen on H-E. The “naked granuloma” appearance—granulomas with only a thin rim of lymphocytes—is characteristic of sarcoidosis and distinguishes it from infections that evoke heavier mixed inflammation or visible organisms.  Vascular changes Small vessels are engulfed by, but not destroyed by, granulomas; no fibrinoid necrosis or leukocytoclastic vasculitis is present. Helps rule out granulomatosis with polyangiitis (Wegener) and other vasculitic mimics.  Together, the constellation of non-caseating epithelioid granulomas with multinucleated giant cells, intracytoplasmic Schaumann and asteroid bodies, scant peripheral lymphocytes, and an absence of identifiable microorganisms or necrosis is diagnostic of sarcoidosis in an oral biopsy. |
| 4.15 | **Diagnosis:** Papillomas of nonkeratinized squamous epithelium; **Key Microscopic Features:** Pedunculated papillary epithelial growths typically lacking significant keratin, with keratinocytes demonstrating edema alongside dilated capillaries, and fibrovascular cores showing hyalinization. | Histopathological Evaluation:  📌 Most Likely Diagnosis:  Condyloma acuminatum (oral wart) – a lesion typically caused by human papillomavirus (HPV), especially types 6 and 11.  🔬 Key Microscopic Features (from both low and high magnification images):  1. Papillary/Verrucous Architecture (Low Power - Image 1):  Exophytic growth pattern with papillary projections supported by fibrovascular cores.  Finger-like projections are lined by stratified squamous epithelium, which is a hallmark of a benign papillomatous lesion.  2. Acanthosis and Hyperkeratosis (High Power - Image 2):  Significant acanthosis (thickening of the spinous layer).  Hyperkeratosis and parakeratosis visible, typical for HPV-related lesions.  3. Koilocytosis (High Power - Image 2):  Koilocytes are present – these are squamous epithelial cells with:  Perinuclear clearing (halos)  Irregular, hyperchromatic nuclei  Koilocytosis is a strong indicator of HPV infection.  4. Minimal/No Cytologic Atypia or Invasion:  No evidence of dysplasia or invasive growth, supporting a benign, HPV-related etiology.  🦠 Etiologic Consideration:  Viral: Human papillomavirus (HPV) – low-risk subtypes (especially HPV 6 and 11), causing oral mucosal warts and condylomas.  Often sexually transmitted but may also spread through autoinoculation or non-sexual contact. | Histopathological diagnosis (single best):  Squamous papilloma – an HPV-associated oral papillomatous lesion  Key microscopic features visible in the provided H-E sections  # Feature How it appears in the images Diagnostic significance  1 Pedunculated, exophytic architecture At low power the lesion is attached by a narrow fibrous stalk and expands into a bush-like mass of projections (Image 1) Favours a papilloma; verruca vulgaris is usually sessile and focal epithelial hyperplasia is flat  2 Multiple slender finger-like papillary fronds Numerous thin, branching projections radiate outward, each discrete from its neighbour (Image 1) Characteristic of squamous papilloma; condyloma acuminatum shows broader, blunted papillae  3 Central fibro-vascular cores Every papillary digit contains loose connective tissue with small vessels (Image 1 & 2) Essential criterion for a true papilloma  4 Stratified squamous epithelium with acanthosis but bland cytology Epithelium is thickened, yet nuclei are uniform and mitoses are basal/normal (Image 2) Excludes dysplastic or malignant processes  5 Surface hyper- / para-keratosis Eosinophilic keratin layer caps many fronds (best seen at higher magnification, Image 2) Common surface change in HPV-induced papillomas  6 Koilocytosis (HPV effect) Scattered superficial keratinocytes show perinuclear clearing with wrinkled, pyknotic nuclei (Image 2, especially outer epithelial layers) Confirms viral (HPV) aetiology and supports papilloma diagnosis  7 Absence of “cupping” rete ridges or mitosoid bodies Rete ridges do not converge centrally (rules out verruca vulgaris); no mitosoid nuclei (rules out focal epithelial hyperplasia) Helps narrow the differential  Taken together, the classical papillary architecture with fibrovascular cores, koilocytotic change, and lack of significant atypia most convincingly points to squamous papilloma of the oral mucosa. |
| 4.16 | **Diagnosis:** Papilloma of squamous epithelium; **Key Microscopic Features:** Papillary epithelial overgrowth with parakeratosis and axially oriented peripheral rete ridges, and fibrovascular cores containing dilated capillaries. | Based on the provided hematoxylin and eosin (H&E) stained histopathological images at different magnifications, the single most likely histopathological diagnosis is:  Condyloma acuminatum (Oral genital wart caused by HPV infection)  Key Microscopic Features Justifying the Diagnosis:  Papillary Architecture:  The low-power image shows exophytic, papillary projections with broad-based, blunted rete ridges.  This papillary growth pattern is a hallmark of condyloma acuminatum.  Acanthosis and Hyperkeratosis:  Marked thickening of the stratified squamous epithelium (acanthosis).  Surface hyperkeratosis is evident (bright pink surface keratin layer).  Koilocytosis:  At higher magnification, koilocytes (squamous epithelial cells with perinuclear clearing and irregular, raisinoid nuclei) are seen in the upper layers of the epithelium.  This is a classic cytopathic effect of human papillomavirus (HPV) infection, typically types 6 and 11.  Minimal Cytologic Atypia:  The epithelial cells show mild to moderate atypia, without features suggestive of malignancy.  There is no evidence of invasion through the basement membrane.  Inflammatory Stroma:  The underlying connective tissue (stroma) may exhibit a mild chronic inflammatory infiltrate, although not prominent in this case.  Summary:  These features—papillary architecture, acanthosis, hyperkeratosis, and especially koilocytosis—are characteristic of HPV-induced condyloma acuminatum. This lesion is considered benign but can be contagious and may recur. It's important to differentiate it from verruca vulgaris and squamous papilloma, which can have overlapping features but differ in histologic detail and clinical presentation. | Single most likely histopathologic diagnosis  Verruca vulgaris (common wart) – an HPV-induced papillary lesion of the oral mucosa  Key microscopic features in the two H-E sections that point to this diagnosis  Feature What you can see in the images Why it matters  Exophytic, papillary (“verruciform”) growth pattern Multiple, finger-like projections of epithelium extend above the mucosal surface; each projection has a central fibro-vascular core (best appreciated in the low-power image). Classic architecture of a wart; distinguishes it from flat epithelial hyperplasia.  Pronounced acanthosis with elongated rete ridges that “cup” around the connective-tissue cores The spinous layer is markedly thickened, and the rete ridges curve inward toward the centre of each papilla. The cupping configuration is typical of verruca vulgaris and less prominent in condyloma acuminatum.  Vertical columns/“church-spire” plugs of parakeratin and orthokeratin between papillae Bright pink keratin cores project vertically within the epithelial clefts (white linear spaces in the high-power view). These keratin columns are a signature finding of common warts.  Hypergranulosis and koilocytosis in the superficial layers Patchy cells with perinuclear clearing and pyknotic nuclei can be seen near the surface; the granular layer is thickened beneath the keratin. Koilocytes and a prominent granular layer reflect HPV cytopathic effect, confirming a viral wart rather than a purely reactive lesion.  Lack of epithelial dysplasia Nuclear polarity and maturation are preserved; no atypical mitoses. Helps rule out verrucous carcinoma or papillary squamous cell carcinoma.  Taken together, the constellation of papillary architecture, keratin “church-spire” plugs, koilocytosis, and cupping rete ridges is diagnostic of verruca vulgaris in the oral cavity. |
| 4.19 | **Diagnosis:** Common wart (verruca vulgaris); **Key Microscopic Features:** Marked hyperkeratosis and papillary epithelial overgrowth with axially tilted rete ridges, and large keratohyaline granules. | Histopathological Diagnosis:  Condyloma acuminatum (Venereal wart) — typically caused by Human Papillomavirus (HPV), most commonly types 6 and 11.  Key Microscopic Features Supporting the Diagnosis:  Low-Magnification Image (1st Image):  Papillary architecture: Multiple exophytic, finger-like projections.  Acanthosis: Thickening of the spinous layer of the epithelium.  Hyperkeratosis: Thickened keratin layer on the surface.  Parakeratosis: Retained nuclei within the stratum corneum.  Koilocytic changes visible even at this level (enlarged cells with clear perinuclear halos).  High-Magnification Image (2nd Image):  Koilocytosis: Classic hallmark of HPV infection — keratinocytes with:  Perinuclear clearing (halo)  Enlarged, irregular, hyperchromatic nuclei.  Binucleation or multinucleation: A common cytopathic effect of HPV.  Hypergranulosis: Thickened granular cell layer.  No evidence of dysplasia: Suggests a benign lesion, consistent with condyloma.  Summary:  These findings—particularly the papillary epithelial hyperplasia, koilocytosis, and hyperkeratosis—are classic for condyloma acuminatum, a benign HPV-associated lesion often found on mucosal surfaces, including the oral cavity. | Histopathological diagnosis (single best):  Verruca vulgaris (common wart) – an HPV-induced verrucous epithelial proliferation  Key microscopic features that support this diagnosis  Level of magnification What is seen Diagnostic implication  Low-power (whole lesion) • Exophytic, verruciform architecture with multiple pointed papillary projections.  • Elongated, slender rete ridges that bend inward toward the centre of each papilla, producing a characteristic “cupping” effect.  • Each papillary projection contains a central fibro-vascular connective-tissue core. Typical gross silhouette of a common wart; the converging rete ridges help distinguish verruca vulgaris from broader, blunted projections of condyloma acuminatum.  High-power • Marked acanthosis with a very thick granular cell layer packed with coarse basophilic keratohyalin granules.  • Numerous koilocytes – keratinocytes showing perinuclear vacuolisation with pyknotic, irregular nuclei – concentrated in the upper spinous and granular layers.  • Surface hyper- and parakeratosis forming rugged keratin flakes (“church-spire” hyperkeratosis).  • No cytologic atypia or invasion. Coarse keratohyalin granules plus koilocytosis are classic HPV-related changes in verruca vulgaris; together with the hyper/parakeratosis they confirm a benign HPV-driven wart rather than dysplasia or neoplasia.  Taken together, the exophytic spiky papillomatosis, converging rete ridges, abundant keratohyalin granules and widespread koilocytosis are textbook for verruca vulgaris in the oral mucosa. |
| 4.28 | **Diagnosis:** Molluscum contagiosum infection; **Key Microscopic Features:** Endophytic epithelial proliferation containing numerous viral inclusions, with abundant large purple-pink molluscum bodies occupying the cytoplasm and displacing the nucleus to the periphery. | Based on the provided hematoxylin and eosin (H&E) stained histopathological images at low and high magnifications, the most likely single histopathological diagnosis is:  Condyloma acuminatum (oral condyloma)  This is a benign epithelial proliferation caused by human papillomavirus (HPV), typically types 6 and 11.  Key Microscopic Features Supporting the Diagnosis:  Papillary architecture (seen in low power image):  The lesion shows papillary or verrucous epithelial hyperplasia with multiple blunt or rounded projections.  There is acanthosis (thickening of the spinous layer of the epithelium).  Elongated rete ridges extending into the connective tissue in a uniform, often club-shaped pattern.  Koilocytosis (seen in high power image):  Koilocytes are hallmark cells of HPV infection. These cells show:  Perinuclear halos (clearings around the nuclei).  Nuclear enlargement, irregularity, and hyperchromasia.  Cytoplasmic vacuolization.  These features are diagnostic of HPV-related epithelial lesions.  Parakeratosis and hypergranulosis:  Parakeratin layer on the surface may be present.  Sometimes, there is a thickened granular cell layer, although this can vary.  Summary:  The combination of papillary epithelial hyperplasia, koilocytosis, and the typical architectural pattern in the oral mucosa is characteristic of oral condyloma acuminatum, a lesion associated with HPV infection. | Histopathological diagnosis (single best):  Molluscum contagiosum (poxvirus infection) of the oral mucosa/skin.  Key microscopic features that support this diagnosis  Level What is seen in the pictures Significance  Low-power view Endo-exophytic, cup-shaped epithelial proliferation with broad, club-shaped rete ridges growing downward around a central invagination Typical overall architecture of a molluscum lesion  Epithelium is markedly acanthotic but orderly, without cytologic atypia Suggests a reactive/viral lesion rather than dysplasia  High-power view Large clusters of swollen keratinocytes whose cytoplasm is packed with homogeneous, eosinophilic inclusion material; the nucleus is small and pushed to the periphery (signet-ring appearance) These are the classic Henderson–Patterson (molluscum) bodies, pathognomonic for poxvirus infection  Inclusions are most numerous in the upper spinous/superficial layers and eventually get released into the central crater Explains the contagious nature of the lesion  No significant dermal/lamina propria inflammatory or granulomatous response Fits the usual bland host reaction seen in molluscum contagiosum  The combination of a cup-shaped epithelial lesion and the presence of intracytoplasmic Henderson-Patterson bodies is diagnostic for molluscum contagiosum and distinguishes it from other infectious or verrucal conditions of the oral and maxillofacial region. |
| 4.36 | **Diagnosis:** Coccidioidomycosis infection; **Key Microscopic Features:** Pseudoepitheliomatous epithelial hyperplasia with granulomatous inflammation extending into muscle, necrotizing and suppurative granulomatous reaction, and granulomas containing thick-walled spherules filled with numerous endospores. | Histopathological Diagnosis:  Deep Fungal Infection – Most likely Histoplasmosis  Key Microscopic Features (from low to high magnification):  Low Magnification (1st image):  Pseudoepitheliomatous hyperplasia (PEH) of the overlying epithelium – a reactive pattern often seen in chronic fungal infections.  Dense granulomatous inflammation in the underlying connective tissue.  Formation of granulomas composed of histiocytes and lymphocytes.  Intermediate Magnification (2nd image):  Multiple granulomas with central collections of histiocytes.  Mixed inflammatory infiltrate with multinucleated giant cells.  Areas of necrosis within granulomas may be seen.  High Magnification (3rd image):  Numerous small, round-to-oval yeast forms (2–5 microns in size), often found within the cytoplasm of macrophages.  These yeasts have a clear halo surrounding them (suggestive of a capsule or retraction artifact).  Minimal budding seen, consistent with Histoplasma capsulatum morphology.  Accompanying chronic inflammation with numerous plasma cells and neutrophils.  Summary:  The combination of pseudoepitheliomatous hyperplasia, granulomatous inflammation, and numerous small intracellular yeasts within histiocytes strongly suggests a diagnosis of Histoplasmosis, a deep fungal infection often seen in immunocompromised individuals or endemic regions. | Most likely histopathological diagnosis  → Coccidioidomycosis (coccidioidal granuloma / “Valley-fever”-type fungal infection)  Key microscopic features in the three H-E images that point to this diagnosis  Magnification level What is seen Why it matters  Low-power (image 1) • Irregular pseudo-epitheliomatous hyperplasia of the surface mucosa  • A well-circumscribed, nodular inflammatory mass that replaces the lamina propria and extends into the submucosa  • Granulomatous architecture with scattered foci of necrosis Fungal infections of the deep mycoses often provoke florid granulomatous inflammation and reactive epithelial hyperplasia resembling a verrucous/leukoplakic lesion clinically.  Intermediate power (image 2) • Confluent granulomas composed of epithelioid histiocytes and multinucleated giant cells  • Numerous large, round, refractile structures sprinkled through the granulomas; some appear empty, others filled with granular basophilic material Suggests a dimorphic fungus that forms spherules rather than budding yeasts; points away from Histoplasma, Blastomyces, Cryptococcus.  High-power (image 3) • Spherical organisms measuring roughly 20-60 µm, each with a double-contoured thick wall (eosinophilic outer rim) and an internal collection of many tiny endospores (fine granular basophilic material)  • Occasional ruptured spherules spilling endospores into the surrounding neutrophil-rich exudate These structures are the pathognomonic spherules of Coccidioides spp. No budding is seen; instead each spherule matures, cleaves internally, and releases numerous 2-5 µm endospores on rupture—exactly the appearance shown.  Putting the features together  The combination of thick-walled, endospore-filled spherules of uniform size and a necrotizing granulomatous reaction is characteristic of Coccidioides immitis/posadasii.  Other deep mycoses differ morphologically:  Blastomyces ⇒ broad-based budding yeasts, single nuclei.  Histoplasma ⇒ tiny intracellular yeasts (2-4 µm) inside macrophages.  Cryptococcus ⇒ narrow-based budding yeasts with a clear mucicarmine-positive capsule.  Rhinosporidium ⇒ sporangia far larger (often >150 µm) with a much thicker hyaline wall and coarse daughter spores; not seen here.  Taken together, the sections depict a classic oral coccidioidal granuloma. |
| 4.39 | **Diagnosis:** Mucormycosis infection; **Key Microscopic Features:** Sinus mucosa showing necrotic and ulcerated regions, ribbon-like fungal hyphae present within necrotic tissue, and arterial wall infiltrated by fungi appearing as clear spaces. | Based on the provided hematoxylin and eosin (H&E)-stained histopathological images of the same lesion at different magnifications, the most likely diagnosis is:  Histopathological Diagnosis:  Histoplasmosis  Key Microscopic Features Supporting the Diagnosis:  Low Power (Image 1):  Diffuse granulomatous inflammation involving the submucosa.  Areas of necrosis and aggregates of inflammatory cells.  Evidence of pseudoepitheliomatous hyperplasia (reactive epithelial proliferation mimicking squamous carcinoma), commonly seen in deep fungal infections.  Intermediate Magnification (Image 2):  Numerous small, oval, basophilic yeast-like organisms (2–5 µm), often seen within macrophages (histiocytes), which is classic for Histoplasma capsulatum.  Dense inflammatory infiltrate composed of lymphocytes, plasma cells, and histiocytes.  Areas of caseating or non-caseating granulomatous inflammation.  High Magnification (Image 3):  Well-formed granulomas with central necrosis and surrounding epithelioid histiocytes and multinucleated giant cells.  Within the necrotic zones and histiocytes, small intracellular organisms (yeast forms of Histoplasma) are discernible.  Vascular invasion and surrounding fibrosis, suggestive of chronic infection.  Conclusion:  The constellation of intracellular small yeast forms within histiocytes, granulomatous inflammation, necrosis, and pseudoepitheliomatous hyperplasia points toward histoplasmosis, a deep fungal infection often affecting the oral and maxillofacial region, particularly in immunocompromised patients or those from endemic regions. | Histopathologic diagnosis (single best answer)  ➜ Mucormycosis (zygomycotic fungal infection) of the oral-maxillofacial tissues.  Key microscopic features supporting this diagnosis  Field Observations Diagnostic significance  Low-power architecture • Ulcerated surface mucosa with an underlying zone of extensive coagulative necrosis.  • Islands of necrotic tissue punctuated by dense acute and chronic inflammatory infiltrates and granulomas.  • Multiple thrombosed / inflamed blood vessels within the necrotic bed. Fleshy, infarct-like necrosis and angiocentric destruction are hallmarks of mucormycosis.  Medium- to high-power fungal morphology • Numerous broad (5 – 15 µm), ribbon-like hyphae that are pauciseptate or non-septate and have irregular, predominantly right-angle (>45°) branches.  • Hyphae are pale-eosinophilic / gray, often folded or collapsed. The size, lack of regular septation, and wide-angle branching distinguish mucorales from Aspergillus (which shows narrow, uniform, septate, 45°-branching hyphae).  Angio-invasion & thrombosis • Fungal hyphae seen invading and occluding vascular lumina with associated fibrin thrombus and neutrophilic debris (suppurative vasculitis). Vascular tropism explains the rapid tissue necrosis typical of mucormycosis.  Suppurative granulomatous response • Central collections of neutrophils and necrotic debris surrounded by palisading epithelioid histiocytes and multinucleated giant cells (“suppurative granulomas”). Common host response in deep fungal infections; supports an infectious (rather than purely vasculitic) etiology.  Surrounding tissue reaction • Concentric rings of reactive fibro-vascular tissue encasing necrotic foci (as in the third image). Represents post-infarct reparative change, further emphasizing angio-invasion.  Collectively, the bread-slice, aseptate, wide-branching hyphae within necrotic, angio-invasive lesions create the classic picture of mucormycosis in oral-maxillofacial pathology. |
| 4.46 | Diagnosis: Secondary syphilis; Key Microscopic Features: Ulceration accompanied by a dense chronic inflammatory infiltrate at the interface in a lichenoid pattern and encircling blood vessels; parakeratosis with both acute and chronic inflammatory cells within the epithelium, sporadic necrotic keratinocytes, and plasma cells in the lamina propria; plasma cells also infiltrating adipose tissue and surrounding vascular structures. | sed on the provided hematoxylin-and-eosin (H&E) stained images of an oral lesion at progressively increasing magnifications, the most likely histopathological diagnosis is:  Deep Fungal Infection – Histoplasmosis  Key Microscopic Features Justifying the Diagnosis:  Granulomatous Inflammation (seen prominently in image 2 and 3):  Presence of well-formed granulomas with epithelioid histiocytes.  Dense infiltrates of lymphocytes, plasma cells, and multinucleated giant cells.  Seen in deep fungal infections, especially in chronic infections like histoplasmosis.  Small Intracytoplasmic Yeast Forms (image 3, high magnification):  Numerous tiny, round to oval organisms (2–5 µm), often within macrophages.  These yeasts have a clear halo suggesting a capsule, characteristic of Histoplasma capsulatum.  Best visualized with special stains (e.g., GMS or PAS), but visible on H&E in high magnification.  Surface Ulceration and Pseudoepitheliomatous Hyperplasia (image 1):  The epithelium appears hyperplastic and ulcerated, commonly seen in fungal infections involving the mucosa.  Underlying heavy chronic inflammatory infiltrate with microabscesses.  Differential Diagnosis Considerations (but less likely):  Leishmaniasis – Similar histiocytic pattern but typically shows Leishman-Donovan bodies (not seen).  Tuberculosis – Granulomas are present but acid-fast bacilli required for confirmation; organisms not seen.  Paracoccidioidomycosis – Yeasts are much larger with characteristic “pilot wheel” appearance.  Cryptococcosis – Larger yeasts with thick capsule; often seen with mucicarmine stain. | Most likely histopathological diagnosis  → Sporotrichosis (infection by Sporothrix schenckii)  Key microscopic features that point to this diagnosis  Field of view What is seen Why it matters  Low-power (1st image) Ulcerated/eroded stratified squamous mucosa overlying a band-like, very neutrophil-rich granulomatous infiltrate that extends deeply Sporotrichosis produces suppurative (neutrophilic) granulomas rather than the purely epithelioid granulomas of e.g. tuberculosis or sarcoidosis  Intermediate-power (2nd image) Extension into submucosal connective tissue and marrow fat with multiple micro-abscesses rimmed by histiocytes & occasional giant cells The pattern of micro-abscesses embedded in a granulomatous background (so-called “suppurative granulomas”) is classic for deep mycoses—sporotrichosis is the prototype  High-power (3rd image) In and around the abscesses are scattered 2–6 µm, oval-to-elongated “cigar-shaped” yeast forms; some show narrow-based budding, some are surrounded by a thin eosinophilic coat (Splendore-Hoeppli material) The small size, distinctive cigar / fusiform shape and narrow-based budding are virtually pathognomonic for Sporothrix schenckii; other deep fungi have different silhouettes (round thick-walled yeasts of blastomycosis, 2–4 µm intracellular dot-like yeasts of histoplasmosis, broad non-septate hyphae of mucormycosis, etc.)  Putting the features together  The triad of (1) ulcerated mucosa, (2) suppurative granulomatous reaction with micro-abscesses, and (3) scattered cigar-shaped budding yeasts is characteristic of sporotrichosis and rules out the other bacterial, viral, fungal and parasitic infections that occur in the oral & maxillofacial region. |

NODULES FIBROUS AND GINGIVAL

| **Fig_no** | **Ground truth** | **GPT 4o answer** | **o3 answer** |
| --- | --- | --- | --- |
| 5.2 | Diagnosis: Fibrous tumor exhibiting reactive hyperkeratosis; Key Microscopic Features: Compact collagen bundles containing enlarged capillary vessels. | Histopathological diagnosis  Irritation fibroma ( a.k.a. traumatic fibroma / focal fibrous hyper-plasia)  Key microscopic features that support this diagnosis  Level Finding Significance  Low-power (first image) • Pedunculated/ polypoid mass covered by stratified squamous epithelium.  • Epithelium is hyper-parakeratinised and acanthotic with elongated, blunt rete ridges. Typical architecture of a reactive mucosal fibrous nodule produced by chronic irritation.  Stromal core (first & second images) • Broad central mass of very dense, eosinophilic, mature collagen bundles arranged in haphazard / swirling (scar-like) fashion.  • Stroma is hypo-cellular; only scattered, bland spindle-shaped fibroblasts are present, with no atypia or mitoses. Dense fibrous connective tissue with paucicellular appearance is characteristic of an irritation fibroma rather than neoplastic or inflammatory lesions.  Vascularity & inflammation • Few, thin-walled slit-like blood vessels scattered through the collagen.  • Only minimal chronic inflammatory infiltrate. Low vascularity and minimal inflammation distinguish it from pyogenic granuloma or other vascular/reactive lesions.  Absence of other diagnostic clues • No multinucleated stellate fibroblasts (rules out giant-cell fibroma).  • No mineralised material (rules out peripheral ossifying fibroma).  • No peripheral giant cells, no ulceration, no dysplasia. Helps narrow the diagnosis to a simple reactive fibrous hyperplasia.  Taken together, the exophytic mucosal nodule with hyperplastic epithelium overlying a hypocellular, densely collagenised, avascular stromal core is classic for an irritation fibroma in the fibrous/gingival category of oral soft-tissue lesions. | Histopathological diagnosis  Irritation fibroma ( a.k.a. traumatic fibroma / focal fibrous hyper-plasia)  Key microscopic features that support this diagnosis  Level Finding Significance  Low-power (first image) • Pedunculated/ polypoid mass covered by stratified squamous epithelium.  • Epithelium is hyper-parakeratinised and acanthotic with elongated, blunt rete ridges. Typical architecture of a reactive mucosal fibrous nodule produced by chronic irritation.  Stromal core (first & second images) • Broad central mass of very dense, eosinophilic, mature collagen bundles arranged in haphazard / swirling (scar-like) fashion.  • Stroma is hypo-cellular; only scattered, bland spindle-shaped fibroblasts are present, with no atypia or mitoses. Dense fibrous connective tissue with paucicellular appearance is characteristic of an irritation fibroma rather than neoplastic or inflammatory lesions.  Vascularity & inflammation • Few, thin-walled slit-like blood vessels scattered through the collagen.  • Only minimal chronic inflammatory infiltrate. Low vascularity and minimal inflammation distinguish it from pyogenic granuloma or other vascular/reactive lesions.  Absence of other diagnostic clues • No multinucleated stellate fibroblasts (rules out giant-cell fibroma).  • No mineralised material (rules out peripheral ossifying fibroma).  • No peripheral giant cells, no ulceration, no dysplasia. Helps narrow the diagnosis to a simple reactive fibrous hyperplasia.  Taken together, the exophytic mucosal nodule with hyperplastic epithelium overlying a hypocellular, densely collagenised, avascular stromal core is classic for an irritation fibroma in the fibrous/gingival category of oral soft-tissue lesions. |
| 5.5 | Diagnosis: Fibrous growth located on the front part of the palatal mucosa; Key Microscopic Features: A nodular lesion composed of hyaline cartilage intermingled with nerve and blood vessel bundles and areas of fully developed hyaline cartilage. | Histopathological Analysis of Provided H&E-Stained Lesion Images (Nodules Fibrous and Gingival Group):  Single Most Likely Diagnosis:  Peripheral Ossifying Fibroma (POF)  Key Microscopic Features Justifying the Diagnosis:  Fibrous Proliferation:  The low-power image shows a well-circumscribed nodular lesion composed predominantly of a cellular fibrous stroma, which is characteristic of a reactive fibrous overgrowth.  Ossification:  The higher magnification image highlights a central area of mineralized material, appearing as trabeculae or globules of ossified tissue (cementum-like or bone-like), surrounded by fibroblastic stroma.  This mineralized component is the hallmark of a POF and helps distinguish it from other reactive gingival lesions like fibromas or pyogenic granulomas.  Overlying Epithelium:  Stratified squamous epithelium with elongation of rete ridges can be observed at the periphery, often seen in reactive gingival lesions.  Lack of Significant Inflammatory Infiltrate:  Minimal inflammatory cells suggest a more fibrous/reactive nature rather than a highly inflamed lesion such as a pyogenic granuloma.  Summary:  The combination of a fibrous stroma, mineralized ossified component, and location in the gingival nodule category strongly supports a diagnosis of Peripheral Ossifying Fibroma, a common reactive gingival lesion. | Histopathological diagnosis (single best):  Peripheral ossifying fibroma (POF) – a reactive gingival lesion arising from the periodontal-ligament zone.  Key microscopic features that support this diagnosis  Field Observations in the provided H&E sections Diagnostic significance  Surface epithelium Intact, keratinised stratified squamous epithelium with elongated/pointed rete ridges (low-power image) Typical of gingival mucosa repeatedly traumatised by an exophytic nodule  Stromal component Fibro-cellular connective tissue that is more collagenous toward the base and more cellular toward the centre The fibrous proliferation is the “fibroma” part of POF  Mineralised material Large, well-circumscribed spherical mass of eosinophilic mineralised tissue in the core of the lesion. At higher power it shows lacunae with entrapped osteocyte-like cells and peripheral osteoblastic rimming → mature lamellar/woven bone; smaller basophilic globules resembling cementum-like calcifications are also scattered Reactive metaplastic production of bone and/or cementum is the defining feature of a peripheral ossifying fibroma  Lesion architecture Polypoid, confined to soft tissue, with no evidence of multinucleated giant cells or exuberant capillary proliferation Rules out peripheral giant-cell granuloma and pyogenic granuloma  Inflammation/vascularity Mild chronic inflammatory infiltrate and patchy vascularity; no haemorrhagic areas filled with giant cells Consistent with POF rather than other common gingival “lump” entities  Putting these together: a purely soft-tissue, fibrous gingival mass that contains centrally placed ossified (bone/cementum-like) trabeculae is characteristic of a peripheral ossifying fibroma. |
| 5.6 | Diagnosis: Fibroma characterized by giant cells; Key Microscopic Features: A fibrous mass exhibiting pointed rete ridges and compact collagen, containing star-shaped and binucleate fibroblasts. | Histopathological Analysis of the Lesion (H&E stained, different magnifications)  📌 Single Most Likely Diagnosis:  Irritation Fibroma (Focal Fibrous Hyperplasia)  🔬 Key Microscopic Features Justifying the Diagnosis:  Surface Epithelium:  Hyperplastic stratified squamous epithelium with elongated, pointed rete ridges, which often appear club-shaped.  Hyperkeratosis may be present due to chronic friction or irritation (though this isn't distinctly shown here).  Underlying Connective Tissue Stroma:  Dense, collagenous fibrous connective tissue: Appears pink and homogenous under H&E stain, as seen in both low and high magnifications.  Scattered fibroblasts with elongated nuclei are visible in the fibrous stroma.  Minimal chronic inflammatory infiltrate, supporting a reactive (rather than inflammatory or neoplastic) lesion.  Architectural Pattern:  The lesion has a nodular architecture and a well-circumscribed yet non-encapsulated appearance.  Absence of features like cellular atypia, mitoses, or infiltrative growth rules out malignancy.  🧠 Clinical Correlation:  Irritation fibromas are benign, reactive lesions commonly seen on the buccal mucosa, tongue, or gingiva in response to local trauma or chronic irritation (e.g., from dental appliances or sharp tooth edges). | Histopathological diagnosis – Giant cell fibroma  Key microscopic features that support this diagnosis  Feature How it appears in the provided slides Why it points to giant cell fibroma  Hyperplastic, papillary/pebbly surface epithelium Low-power view shows parakeratinised stratified squamous epithelium forming long, slender, pointed rete ridges that “drop” between fibrous papillae. Giant cell fibroma characteristically has a papillary or lobulated surface produced by epithelial hyperplasia overlying the fibrous mass.  Fibro-cellular lamina propria with numerous large, stellate-shaped fibroblasts In the higher-magnification image the connective-tissue cores are occupied by plump, angular or stellate cells with pale cytoplasm scattered among loosely arranged collagen bundles. The presence of these distinctive stellate fibroblasts is the hallmark of a giant cell fibroma and distinguishes it from an irritation fibroma (which shows mainly dense collagen with bland spindle fibroblasts).  Multinucleation / “giant” fibroblasts Several of the stellate cells show two or more vesicular nuclei within the same cytoplasm. Binucleated and multinucleated fibroblasts give the lesion its name; they are uncommon in most other routine fibrous gingival growths.  Sparse inflammatory infiltrate and scant vasculature Both sections show only occasional small vessels and very few inflammatory cells. This relative paucity of inflammation is typical and separates the lesion from reactive inflammatory nodules such as a pyogenic granuloma or fibrous hyperplasia secondary to chronic irritation.  Absence of calcifications, odontogenic rests or giant cells of macrophage lineage No bone, cementum-like material, odontogenic epithelium or foreign-body/osteoclastic-type giant cells are present. Helps rule out peripheral ossifying fibroma, peripheral odontogenic fibroma and peripheral giant-cell granuloma, respectively.  Taken together, the papillary epithelial architecture plus the fibrous stroma populated by characteristic stellate and multinucleated fibroblasts provide a classic picture of giant cell fibroma. |
| 5.7 | Diagnosis: Fibroma characterized by giant cells; Key Microscopic Features: A fibrous nodule displaying a papillary surface, composed of large and binucleate fibroblast-like cells within dense collagen. | Based on the provided hematoxylin and eosin (H&E) stained images of a lesion from the nodules, fibrous and gingival category in oral and maxillofacial pathology, the single most likely histopathological diagnosis is:  Peripheral Ossifying Fibroma (POF)  Key Microscopic Features Justifying the Diagnosis:  Low-power view (first image):  Lobulated, exophytic, and well-circumscribed lesion arising from the mucosa.  Surface covered by stratified squamous epithelium, often ulcerated or showing hyperplasia.  The lesion is composed predominantly of cellular fibrous connective tissue.  Multiple areas show mineralized material—appears as irregular basophilic (purple) structures suggestive of bone or cementum-like calcifications.  High-power view (second image):  Highly cellular fibrous stroma with plump, spindle-shaped fibroblasts.  Presence of mineralized material: eosinophilic, osteoid-like or cementoid structures.  No significant atypia or high mitotic activity, consistent with a benign reactive process.  Scattered inflammatory cells may be present, typical in reactive lesions.  Summary:  These features are classic for Peripheral Ossifying Fibroma, a reactive gingival growth often arising from the periodontal ligament, commonly seen in adolescents and young adults. It should be distinguished from other fibrous gingival lesions like fibromas, pyogenic granulomas, or peripheral giant cell granulomas. | Most likely histopathological diagnosis  → Giant cell fibroma (oral mucosa type)  Key microscopic features supporting the diagnosis  Level of magnification What is seen Significance  Low-power overview • Pedunculated/polypoid nodule with lobulated, papillary surface projections.  • Covered by relatively thin stratified squamous epithelium that forms elongated, slender rete ridges between the papillary “lobes”. Giant-cell fibromas classically present as small, papillary or sessile nodules of gingiva or mucosa rather than a smooth-domed fibroma.  High-power stroma • Fibro-collagenous connective tissue that is hypo-cellular overall but harbours scattered, very large, stellate- or spindled fibroblasts with ample eosinophilic cytoplasm.  • Many of these fibroblasts are bi- or multinucleated (“giant” fibroblasts); nuclei are vesicular without atypia.  • Fibroblasts lie in a loose whorled collagen matrix with a delicate myxoid quality.  • Only a scant chronic inflammatory infiltrate; no calcifications, hemorrhage or giant-cell granuloma–type clusters. The hallmark of a giant-cell fibroma is the presence of isolated, bizarre-shaped, often multinucleated fibroblasts scattered through a collagenous stroma beneath otherwise ordinary surface epithelium. Other common gingival nodules (irritation fibroma, peripheral ossifying fibroma, peripheral giant-cell granuloma) lack this constellation of multinucleated stellate fibroblasts.  Taken together, the lobulated papillary architecture plus the distinctive population of large stellate/giant fibroblasts pinpoints the lesion as a giant cell fibroma of the gingiva/oral mucosa. |
| 5.8 | Diagnosis: Fibroma with sclerotic features; Key Microscopic Features: A sharply demarcated nodular lesion of thick, glassy collagen containing star-shaped and spindle-shaped fibroblasts separated by tissue clefts. | Based on the provided hematoxylin and eosin (H&E) stained histological images of a lesion from the nodules, fibrous and gingival group in oral and maxillofacial pathology, here is the analysis:  Most Likely Histopathological Diagnosis:  Irritation Fibroma (Focal Fibrous Hyperplasia)  Key Microscopic Features Supporting the Diagnosis:  Low Magnification (First Image):  Nodular proliferation beneath the epithelium, well-circumscribed but non-encapsulated.  Hyperplastic stratified squamous epithelium, often hyperkeratotic, overlying the lesion.  Dense collagenized connective tissue stroma, appearing eosinophilic and acellular in many areas.  Minimal inflammation (or occasional chronic inflammatory infiltrate) can be seen.  High Magnification (Second Image):  Abundant collagen fibers, densely packed and arranged in a swirling or haphazard fashion.  Scattered fibroblasts with elongated, spindle-shaped nuclei.  No evidence of atypia, mitoses, or neoplastic features—typical of reactive rather than neoplastic lesions.  The collagen appears intensely pink (eosinophilic), while the fibroblast nuclei stain basophilic (blue/purple).  Conclusion:  The histological features—nodular mass with dense fibrous connective tissue, overlying epithelial hyperplasia, and absence of cellular atypia—are consistent with an irritation fibroma, a common benign reactive lesion resulting from chronic irritation or trauma, frequently occurring on the gingiva or buccal mucosa. | Histopathological diagnosis  Irritation (traumatic) fibroma – a focal fibrous hyperplasia of the oral mucosa.  Key microscopic features supporting this diagnosis  Field Observations Significance  Overall architecture (low-power view) • Dome-shaped, well-circumscribed but unencapsulated submucosal nodule.  • Covered by stratified squamous oral epithelium that is slightly hyperkeratotic and flattened over the lesion. Pattern typical of a reactive fibrous nodule produced by chronic mechanical irritation.  Stromal composition • Mass is composed almost entirely of densely collagenised connective tissue that stains uniformly eosinophilic (pink).  • Collagen bundles are thick and arranged in swirling / interlacing patterns. Dense, mature collagen is characteristic of irritation fibroma; the swirling or radiating bundle pattern reflects long-standing fibrosis.  Cellularity • Scattered, slender spindle-shaped fibroblast nuclei (basophilic) are present but relatively sparse.  • No pleomorphism, mitotic figures, multinucleated giant cells or prominent inflammatory infiltrate. Low cellularity with bland fibroblasts distinguishes it from more cellular fibrous lesions (e.g., peripheral ossifying fibroma, giant cell fibroma, fibromatosis).  Absence of additional elements • No calcified/bone-like material, cementum-like globules or dystrophic calcifications.  • No multinucleated giant cells, hemorrhagic foci or hemosiderin.  • Minimal chronic inflammatory cells. Helps exclude peripheral ossifying fibroma (would show mineralised foci) and peripheral giant-cell granuloma (would show numerous osteoclast-type giant cells and hemorrhage).  Together, the combination of a mucosa-covered nodule composed of dense, paucicellular collagen with scattered inactive fibroblasts and no distinctive calcified or giant-cell component is diagnostic of an irritation (traumatic) fibroma. |
| 5.12 | Diagnosis: Fibrous gingival growth or inflammatory fibrous hyperplasia; Key Microscopic Features: A fibrous nodule accompanied by chronic inflammatory cells; surface epithelium showing keratin loss, epithelial thickening, intercellular edema, and persistent inflammation; and a prominent infiltrate of plasma cells. | Based on the three hematoxylin-and-eosin (H&E) stained histological images provided at low, medium, and high magnification, the most likely histopathological diagnosis is:  Lymphoid Hyperplasia (Reactive Lymphoid Hyperplasia)  Key Microscopic Features Justifying the Diagnosis:  1. Low Magnification (First Image):  Nodular architecture is clearly seen with well-circumscribed areas involving the subepithelial connective tissue.  The surface epithelium is intact, suggesting a non-ulcerated lesion.  Numerous discrete, well-defined lymphoid aggregates are visible throughout the section.  2. Medium Magnification (Second Image):  There is a dense lymphocytic infiltrate in the lamina propria/submucosa.  Overlying stratified squamous epithelium appears hyperplastic in some areas, but not dysplastic.  The lymphoid follicles are well-formed, with some demonstrating germinal centers, indicating a reactive rather than malignant process.  3. High Magnification (Third Image):  Germinal centers are clearly visible, with a mixed population of centroblasts and centrocytes.  A "starry-sky" appearance is evident due to tingible body macrophages.  Polymorphous infiltrate (mixture of small lymphocytes, immunoblasts, and macrophages) supports a reactive, non-neoplastic process.  Conclusion:  The presence of multiple lymphoid follicles with germinal centers, the absence of cytologic atypia, and the preserved architecture all support a diagnosis of reactive lymphoid hyperplasia, commonly found in lymphoid tissue-rich regions such as the gingiva or soft palate. This is a benign and self-limiting condition often seen in response to antigenic stimulation. | Histopathological diagnosis –  Reactive (benign) follicular lymphoid hyperplasia of the oral mucosa  Key microscopic features that support this diagnosis  Feature What is seen in the fields supplied Why it points to reactive follicular lymphoid hyperplasia  Polypoid mucosal architecture with acanthotic stratified squamous epithelium Low-power slide shows a broad-based, exophytic mucosal nodule; the epithelium is thickened with elongated/club-shaped rete ridges but remains orderly and lacks dysplasia Reactive lesions usually retain a normal but hyperplastic epithelium rather than showing neoplastic epithelial change  Numerous well-formed lymphoid follicles beneath the epithelium At medium power multiple round aggregates are present, each with a pale germinal centre and a darker mantle zone A nodular pattern with clearly demarcated germinal centres is characteristic of follicular lymphoid hyperplasia, not of fibrous overgrowths or giant-cell lesions  Reactive germinal-centre morphology High-power image demonstrates a polymorphous cell population in the centres (centrocytes, immunoblast-type lymphocytes) with interspersed tingible-body macrophages; peripherally a rim of small mature lymphocytes forms a mantle The mixed cell size, presence of macrophages “eating” apoptotic debris, and preservation of a mantle zone are hallmarks of reactive rather than clonal (lymphomatous) germinal centres  No cytologic atypia or destructive growth Cells have small, round to slightly irregular nuclei, smooth nuclear membranes, inconspicuous nucleoli, and scant cytoplasm; no necrosis, no atypical mitoses, and no invasion beyond the submucosa Absence of atypia and tissue destruction separates this benign process from malignant lymphoma  Fibro-collagenous stroma with scattered chronic inflammatory cells The interfollicular areas show delicate collagen bundles admixed with plasma cells and small lymphocytes Fits with a reactive mucosal lesion; supports placement in the “fibrous/gingival nodule” clinical group even though the dominant component is lymphoid  Taken together, the combination of an acanthotic but non-dysplastic surface epithelium, a subepithelial sheet of reactive lymphoid tissue forming numerous germinal centres, and the lack of cytologic atypia or invasive behaviour is diagnostic of reactive follicular lymphoid hyperplasia (benign lymphoid hyperplasia/pseudolymphoma) of the oral mucosa. |
| 5.13 | Diagnosis: Pregnancy-associated granuloma; Key Microscopic Features: An ulcerated, loosely lobular proliferation of capillaries; proliferating endothelial cells forming dilated capillaries with mild nuclear bulging; and focal areas with increased cellularity. | Based on the histopathological analysis of the three hematoxylin and eosin (H&E) stained images at increasing magnifications, the single most likely diagnosis is:  Peripheral Ossifying Fibroma (POF)  Key Microscopic Features Supporting the Diagnosis:  Low magnification (first image):  A well-circumscribed, nodular lesion originating from the gingival mucosa.  The lesion is composed of cellular fibrous connective tissue, showing a lobular or zonal growth pattern.  Intermediate magnification (second image):  Proliferation of plump fibroblasts within a collagenous stroma.  Presence of numerous capillary-sized blood vessels.  Scattered mineralized areas – likely representing early osteoid or cementoid formation.  High magnification (third image):  Clear evidence of mineralized material resembling bone or cementum.  Chronic inflammatory infiltrate in the stroma, likely due to irritation.  Vascular proliferation and occasional hemosiderin-laden macrophages may be present, consistent with a reactive gingival lesion.  Summary:  Peripheral Ossifying Fibroma is a reactive lesion that commonly arises on the gingiva, especially in the interdental papilla. The combination of a cellular fibrous stroma, presence of mineralized tissue (bone/cementum-like), and localization to the gingiva strongly supports this diagnosis. | Histopathological diagnosis (single best):  Pyogenic granuloma – lobular capillary haemangioma of the gingiva  Key microscopic features that support the diagnosis  Low → high-power findings How they point to pyogenic granuloma  Lobular architecture – At scanning power the lesion is divided into well-circumscribed lobules separated by thin fibrous septa beneath an intact oral mucosal epithelium. The “lobular” growth pattern is the signature feature of a lobular capillary haemangioma.  Proliferation of numerous small blood vessels – capillary-sized channels, many packed with erythrocytes, branch and anastomose throughout the lesion. A dense capillary network is typical; other fibrous gingival nodules (e.g., fibroma, peripheral ossifying fibroma) lack this prominent vascular component.  Plump endothelial cells lining the vascular spaces, often in a hob-nail/tufted arrangement but without significant cytologic atypia. Endothelial tufting with bland nuclei is characteristic and helps distinguish it from granulation tissue that shows flatter endothelial cells.  Fibro-myxoid stroma between the lobules with delicate collagen fibres. This loose, oedematous connective tissue is expected in the “early”/cellular phase of pyogenic granuloma.  Mixed inflammatory infiltrate – scattered neutrophils, lymphocytes and plasma cells permeate the stroma and sometimes extend into the vessel walls. The brisk inflammation reflects chronic irritation/trauma and is common in oral pyogenic granulomas.  Focally, the surface epithelium is flattened/attenuated (sometimes ulcerated in other cases). Surface ulceration or thinning is frequent because the lesion protrudes into the oral cavity and is easily traumatised.  Taken together, the lobular capillary proliferation with plump endothelial cells, the supporting fibro-myxoid stroma and mixed inflammation beneath oral mucosa are diagnostic of pyogenic granuloma (lobular capillary haemangioma) in the gingival/fibrous nodule category. |
| 5.14 | Diagnosis: Peripheral ossifying fibroma; Key Microscopic Features: Proliferation of spindle-shaped fibroblast-like cells laying down immature woven bone bordered by osteoid; formation of cementum-like droplets; and cementum droplets with lamellar, psammomatoid structure. | Histopathological Diagnosis:  Peripheral Giant Cell Granuloma (PGCG)  Key Microscopic Features Justifying the Diagnosis:  Multinucleated Giant Cells:  The second and third images (higher magnification) show numerous multinucleated giant cells scattered throughout the lesion. These cells are typically osteoclast-like and are a hallmark of PGCG.  Fibrovascular Stroma:  The background stroma is composed of plump spindle-shaped fibroblasts and small capillaries, often with extravasated red blood cells—features well demonstrated in the images.  Hemorrhagic and Hemosiderin Deposition:  Although not vividly pigmented in the images, areas with hemorrhage (especially the first image) are common in PGCG and may show hemosiderin-laden macrophages on higher magnification.  Cellularity and Inflammatory Infiltrate:  Moderate cellularity with occasional mononuclear inflammatory infiltrate is evident, a frequent feature in PGCG.  Lack of Atypia or Malignancy:  Despite the cellularity and presence of giant cells, there is no evidence of cellular atypia or mitotic figures suggestive of a malignant process.  Summary:  These features—especially the presence of numerous multinucleated giant cells within a fibrovascular stroma—are diagnostic of Peripheral Giant Cell Granuloma, a reactive lesion commonly seen on the gingiva or alveolar mucosa. | Histopathological diagnosis – Peripheral ossifying fibroma  ( also called peripheral cemento-ossifying fibroma, a reactive fibro-osseous gingival nodule )  Microscopic features in the provided H-E sections that lead to this diagnosis  Field Observations Diagnostic significance  Low-power architecture Nodular lesion made of highly cellular fibrous connective tissue separated by numerous, irregular dark-purple islands/plaques A reactive fibroblastic mass rather than an epithelial or vascular process  Nature of the fibrous stroma Proliferation of plump spindle-shaped fibroblasts arranged in short fascicles and whorls; collagen bundles delicate Characteristic active periodontal-ligament-type fibroblasts seen in POF  Calcified component Multiple foci of mineralised material:  • Irregular trabeculae and curvilinear spicules of woven/lamellar bone  • Concentric lamellated “cementoid” ossicles (round, intensely basophilic bodies with onion-skin lines) A mixed bone- and cementum-like matrix dispersed through a fibrous stroma is the hallmark of an ossifying (cementifying) fibroma  Osteoblastic activity Some ossicles/trabeculae show a thin rim of plump osteoblastic cells Confirms true bone formation rather than dystrophic calcification  Giant/ multinucleate cells Occasional multinucleated or bizarre-shaped fibroblasts at higher magnification Frequently encountered but not prominent in POF; helps exclude peripheral giant-cell granuloma where giant cells dominate  Inflammation / surface Sparse chronic inflammatory cells; surface epithelium not included in the cuts (common to sample only the core in excisional biopsies) Compatible with a reactive gingival nodule rather than an aggressive neoplasm  Why other lesions are unlikely  Traumatic/irritation fibroma – lacks the extensive, organised mineralised component.  Peripheral giant-cell granuloma – dominated by numerous osteoclast-type giant cells and haemorrhage, which are not present here.  Pyogenic granuloma – shows lobular capillary proliferation, absent in these slides.  Together, the cellular periodontal-ligament-type fibrous tissue plus mixed bone-/cementum-like calcifications at several stages of maturation are diagnostic of peripheral ossifying fibroma. |
| 5.15 | Diagnosis: Peripheral ossifying fibroma; Key Microscopic Features: A nodular lesion of cellular fibrous tissue containing abundant woven bone; spindle cell proliferation producing osteoid and woven bone; and osteoid bordered by layers of active osteoblasts housing large osteocytes. | Histopathological Diagnosis:  Peripheral Ossifying Fibroma (POF)  Key Microscopic Features Supporting the Diagnosis:  Fibrocellular Stroma:  All three images, especially the higher magnification one, show a highly cellular fibrous connective tissue stroma with plump fibroblasts and some degree of collagenization.  The stroma is characteristic of reactive fibrous proliferations seen in gingival lesions like POF.  Presence of Mineralized Components:  The central and lower magnification images show trabeculae and irregular calcified material within the fibrous stroma, consistent with bone or cementum-like material, a hallmark of POF.  This mineralization appears basophilic to eosinophilic and is variably shaped, indicating reactive bone formation.  Surface Epithelium (at lower magnification):  The low magnification image reveals the lesion is submucosal, with a relatively intact stratified squamous surface epithelium, consistent with the peripheral (extraosseous) nature of the lesion.  Lack of Atypia or Malignancy:  Despite the cellularity, there is no significant cytologic atypia, mitotic activity, or features to suggest malignancy. This supports a benign reactive process.  Summary:  Peripheral Ossifying Fibroma is a common reactive gingival lesion, typically arising from the periodontal ligament. The combination of fibrous proliferation and mineralized material (bone or cementum-like) in a submucosal location on the gingiva, without atypia, is characteristic and diagnostic. | Histopathological diagnosis (single best):  Trabecular-type juvenile ossifying fibroma (JOF) – a highly-cellular, benign fibro-osseous neoplasm that typically presents on the gnathic bones or, as in this case, as a peripheral gingival mass.  Key microscopic features that support this diagnosis  Field (magnification) Salient findings Diagnostic significance  Low power • Well-circumscribed, lobulated nodule separated from the surrounding tissue by a thin fibrous rim.  • Central mass composed of innumerable, branching, inter-anastomosing trabeculae of eosinophilic bone/osteoid (“Chinese-letter” pattern). A well-delineated, expansile fibro-osseous lesion argues for an ossifying fibroma rather than an ill-defined process such as fibrous dysplasia.  Intermediate power • Trabeculae are immature woven bone containing osteocytes in lacunae and lined by plump osteoblasts (prominent osteoblastic rimming).  • Inter-trabecular stroma is very cellular, made up of bundles and whorls of uniform spindle-shaped fibroblasts. The combination of abundant woven bone with continuous osteoblastic rimming in a highly cellular stroma is characteristic of the trabecular variant of JOF; conventional ossifying fibroma is usually less cellular, while fibrous dysplasia lacks osteoblastic rimming.  High power • Spindle-cell stroma shows minimal pleomorphism and low mitotic activity (benign).  • No epithelial islands, multinucleated giant cells, or cementicle-type calcifications that would suggest peripheral odontogenic fibroma or peripheral ossifying fibroma. Helps exclude other gingival fibrous nodules (e.g., peripheral ossifying fibroma, peripheral odontogenic fibroma) and malignant entities (e.g., osteosarcoma, which would show marked atypia and atypical mitoses).  Summary of the pattern  Sharply demarcated lesion composed of hyper-cellular fibrous tissue.  Copious irregular, branching trabeculae of immature woven bone/osteoid with continuous osteoblastic rimming.  Absence of malignancy (little cytologic atypia, no infiltrative growth).  These features together are diagnostic of trabecular juvenile ossifying fibroma. |
| 5.19 | Diagnosis: Peripheral giant cell granuloma; Key Microscopic Features: A cellular lesion touching the epithelial surface focally; sheets of multinucleated giant cells and mononuclear cells in a vascular stroma; and multinucleated giant cells with occasional mitotic figures in the mononuclear component. | Histopathological Analysis:  Single Most Likely Diagnosis:  Peripheral Giant Cell Granuloma (PGCG)  Justification & Key Microscopic Features:  Across the three images at increasing magnifications, the following consistent and defining histological features are observed:  1. Multinucleated Giant Cells:  Abundant multinucleated giant cells are scattered throughout the lesion, often surrounded by a background of mononuclear stromal cells.  These are a hallmark of PGCG and distinguish it from other fibrous or reactive gingival lesions.  2. Vascular Stroma:  The background stroma is highly vascular, with capillary proliferation and extravasated red blood cells visible, particularly in the second and third images.  This supports the reactive, vascular nature of PGCG.  3. Fibroblastic Proliferation:  Numerous plump spindle-shaped stromal cells, likely fibroblasts or myofibroblasts, are dispersed in a fibrous matrix.  The cellularity of the stroma is moderate to high, consistent with PGCG.  4. Overlying Epithelium:  In the low-power image (first one), there is parakeratinized stratified squamous epithelium overlying the lesion, which is typically hyperplastic due to chronic irritation or trauma.  This epithelium appears intact but shows slight rete ridge elongation, common in reactive gingival lesions.  5. Hemosiderin and Hemorrhage:  Scattered extravasated erythrocytes and possible hemosiderin deposition suggest prior hemorrhage, reinforcing the reactive/inflammatory nature.  Differential Diagnosis Considered:  Peripheral Ossifying Fibroma (lacks giant cells, more ossification)  Pyogenic Granuloma (more capillary proliferation, lacks giant cells)  Central Giant Cell Granuloma (intraosseous, not peripheral/gingival) | Histopathological diagnosis – Nodular fasciitis (oral / gingival variant)  Key microscopic features that point to nodular fasciitis  Field view What is seen Why it matters  Low-power overview - A dome-shaped, unencapsulated but well-circumscribed nodule immediately beneath an intact stratified squamous mucosa.  - No lobular architecture or mineralised foci. A reactive-type, subepithelial mass rather than a true neoplasm; absence of lobules rules out pyogenic granuloma, and lack of bone/cementum excludes peripheral ossifying fibroma.  Intermediate magnification - Uniformly cellular lesion composed of plump spindle / stellate cells set in a loose myxoid-to-collagenous matrix.  - Cells arranged in short, irregular fascicles giving a “tissue-culture” appearance.  - Numerous thin-walled, branching capillaries with prominent extravasated erythrocytes scattered throughout. The tissue-culture pattern of fibroblasts/myofibroblasts with conspicuous haemorrhage is classic for nodular fasciitis; the vascular pattern and RBC leakage help separate it from ordinary fibroma.  High-power view - Fibroblasts/myofibroblasts have oval-to-slightly elongated nuclei, delicate chromatin, small nucleoli; cytoplasm is pale eosinophilic.  - Mitotic figures may be present but are typical (not atypical).  - Scattered lymphocytes and occasional plasma cells pepper the stroma; no multinucleated giant cells. Cytologic blandness despite brisk mitoses, together with the mixed inflammatory infiltrate, fits a reactive fasciitis rather than a sarcoma; absence of osteoclast-type giant cells excludes peripheral giant-cell granuloma.  Putting the changes together  The combination of:  a rapidly formed, submucosal nodule,  plump spindle / myofibroblastic cells in a loose myxoid background with a pronounced “tissue-culture” growth pattern,  abundant extravasated red blood cells and thin-walled vessels, and  sparse chronic inflammation without significant atypia  is diagnostic of nodular fasciitis in the oral/gingival region. |
| 5.20 | Diagnosis: Peripheral giant cell granuloma; Key Microscopic Features: Hemosiderin-laden macrophages and siderophages are abundant at the lesion’s periphery adjacent to the grenz zone, accompanied by numerous hemorrhage-associated spindled monocytic cells and multinucleated giant cells. | Histopathological Diagnosis:  Kaposi Sarcoma  Key Microscopic Features Supporting the Diagnosis:  1. Low-power view (first image):  Epidermal atrophy: The overlying epithelium appears thinned.  Grenz zone: A clear, uninvolved area of collagen just beneath the epithelium separating the lesion from the epidermis.  Dermal proliferation: Spindle cell proliferation is seen in the dermis, forming nodular or plaque-like aggregates.  2. Medium- and high-power views (second and third images):  Spindle cell proliferation: Monomorphic spindle cells arranged in short fascicles, a hallmark of Kaposi Sarcoma.  Slit-like vascular spaces: Irregular, slit-like, or crescentic vascular spaces filled with erythrocytes among the spindle cells.  Erythrocyte extravasation and hemosiderin: Numerous red blood cells are found outside vessels, often accompanied by hemosiderin-laden macrophages.  Hyaline globules: Eosinophilic, round cytoplasmic inclusions seen in some of the spindle cells.  Mitotic figures: Though usually low to moderate, mitotic activity may be noted.  These histological findings are highly characteristic of Kaposi Sarcoma, a vascular neoplasm associated with Human Herpesvirus 8 (HHV-8), and often found in immunocompromised patients, particularly those with HIV/AIDS. | Histopathological diagnosis  Solitary oral myofibroma (myofibroma / myopericytoma-type lesion)  Key microscopic features supporting the diagnosis  Field Observation Significance  Low-power architecture Well-circumscribed, unencapsulated submucosal nodule. A broad, pale, hypocellular “caps” of hyalinised collagen lies immediately beneath the stratified squamous epithelium, while the deeper core is much more cellular. Classic biphasic (zoning) pattern of myofibroma – peripheral maturing fibrous zone and central proliferative myoid zone.  Cellular component (high power) Plump spindle-to-ovoid myofibroblasts arranged in short intersecting fascicles and whorls; nuclei oval with fine chromatin, small nucleoli; mitoses are present but typical; no atypia. Bland myofibroblastic cytology typical of myofibroma; mitotic activity without atypia helps rule out sarcoma.  Basicmedical Key  Vascular pattern Numerous thin-walled, branching (“staghorn”/hemangiopericytoma-like) vessels; frequent foci of extravasated erythrocytes surrounding them. Characteristic pericytomatous vasculature of myofibroma; the leaking vessels explain the patchy red cell extravasation seen throughout.  Stroma Alternating myxoid to collagenised areas; peripheral zone shows dense eosinophilic collagen, central zones looser. Reinforces the biphasic nature and distinguishes the lesion from uniformly myxoid nodular fasciitis or uniformly collagenous irritation fibroma.  Basicmedical Key  Host epithelium Overlying epithelium is intact but shows elongated rete ridges (“pseudoparakeratosis”) dipping toward the mass. A common reactive change over slow-growing submucosal nodules and supports a benign process.  Together, the biphasic zoning (peripheral hyaline / central cellular), hemangiopericytoma-like vasculature with red-cell extravasation, and a proliferation of bland myofibroblasts are diagnostic for a solitary myofibroma in the oral cavity, differentiating it from other gingival / fibrous nodules such as irritation fibroma, pyogenic granuloma, peripheral giant-cell granuloma, or nodular fasciitis. |
| 5.24 | Diagnosis: Gingival fibrous hyperplasia; Key Microscopic Features: Fibrous stroma shows acanthosis with pronounced chronic inflammation and spongiosis, alongside apoptotic cells and a prominent plasma cell infiltrate within the connective tissue. | Histopathological Diagnosis:  Peripheral Giant Cell Granuloma (PGCG)  Key Microscopic Features Observed in the Images:  Multinucleated Giant Cells:  The high-power image (third image) clearly reveals numerous multinucleated giant cells dispersed throughout the lesion, particularly within a background of spindle-shaped mononuclear stromal cells.  These giant cells are often clustered around hemorrhagic areas, a hallmark of PGCG.  Fibrovascular Connective Tissue Stroma:  The stroma is cellular, composed of plump spindle cells with prominent vasculature and interspersed inflammatory infiltrates, including lymphocytes and plasma cells.  There is often extravasated red blood cells, as noted here.  Overlying Stratified Squamous Epithelium:  The surface mucosa in the low- and intermediate-power images (first and second images) is composed of stratified squamous epithelium, which is intact but may be hyperplastic due to reactive changes.  Ulceration and Inflammatory Infiltrate (Focal):  Evidence of chronic inflammatory cells in the subepithelial connective tissue is seen, likely due to trauma or ulceration.  Lobulated Architecture and Nodularity:  The low-power view demonstrates a nodular architecture consistent with an exophytic lesion, as typically seen in PGCG.  Conclusion:  The combination of multinucleated giant cells within a fibrovascular stroma, chronic inflammatory infiltrate, hemorrhage, and association with the gingival epithelium is characteristic of Peripheral Giant Cell Granuloma, a reactive lesion commonly occurring on the gingiva or alveolar ridge. | Histopathological diagnosis  Inflammatory fibrous hyperplasia (-“irritation” fibroma / inflamed fibroepithelial polyp)  Key microscopic features that support the diagnosis  Level of magnification What is seen Why it points to inflammatory fibrous hyperplasia  Low-power (whole section) • Polypoid, well-circumscribed mucosal mass  • Intact, thickened surface squamous epithelium covering a central core of connective tissue that looks fibrous rather than lobular or mineralised A reactive fibrous nodule typically grows as a dome-shaped or pedunculated mass beneath hyperplastic epithelium.  Intermediate power • Epithelium is markedly acanthotic with elongated/club-shaped rete ridges (sometimes pseudo-epitheliomatous)  • Immediately below the epithelium a dense band of chronic inflammatory cells (lymphocytes ± plasma cells)  • Numerous small capillaries and scattered extravasated erythrocytes The chronic inflammatory “collar” and reactive vascularity indicate continual surface trauma/irritation – a hallmark of an inflamed fibrous hyperplasia rather than a true fibroma or a vascular/giant-cell/ossifying lesion.  High power • Core composed of interlacing and swirling bundles of collagen fibres with scattered plump fibroblasts  • No multinucleated giant cells, ossification, or lobular capillary architecture  • Inflammatory infiltrate mixed but dominated by plasma cells/lymphocytes Swirling mature collagen with fibroblasts is characteristic of a fibrous hyperplasia. Absence of giant cells (rules out peripheral giant-cell granuloma), bone/osteoid (rules out peripheral ossifying fibroma), and lobular capillary proliferation (rules out pyogenic granuloma) focuses the diagnosis on an inflamed fibrous hyperplasia.  These combined features—reactive epithelial hyperplasia, a fibrous connective-tissue core thrown into whorls, and a brisk chronic inflammatory infiltrate secondary to surface irritation—are diagnostic of inflammatory fibrous hyperplasia. |
| 5.28 | Diagnosis: Metastatic renal cell carcinoma to the gingiva; Key Microscopic Features: Neoplastic tissue within the gingival mucosa forms large lobules of nested tumor cells with extensive fresh hemorrhage, featuring nests of clear cells encircled by dilated, congested vascular channels. | Based on the provided hematoxylin-and-eosin (H&E) stained histological images taken at different magnifications of a lesion in the "nodules fibrous and gingival" group of oral and maxillofacial pathology, the single most likely histopathological diagnosis is:  Pyogenic Granuloma (Lobular Capillary Hemangioma)  Key Microscopic Features Supporting the Diagnosis:  Lobular Arrangement of Capillaries (Best seen in the 2nd and 3rd images):  There is a prominent lobular architecture of proliferating small blood vessels.  These capillaries are lined by flattened endothelial cells and filled with erythrocytes.  Edematous and Inflammatory Stroma (1st and 2nd images):  The lesion shows a loose, edematous stroma with a prominent inflammatory infiltrate, predominantly composed of neutrophils and lymphocytes.  Scattered hemorrhage is visible.  Ulcerated Surface Epithelium (1st image):  The overlying stratified squamous epithelium appears ulcerated or thinned, often seen in pyogenic granulomas due to trauma or secondary infection.  Mixed Vascular and Fibrous Tissue:  The stroma around the lobular capillary aggregates shows some degree of fibrosis, which is consistent with a maturing or older pyogenic granuloma.  Conclusion:  The characteristic lobular capillary proliferation, combined with surface ulceration, edematous inflamed stroma, and numerous blood-filled capillaries, is diagnostic of pyogenic granuloma. This lesion is a common benign vascular lesion of the oral mucosa, often reactive in nature. | Histopathological diagnosis  Pyogenic granuloma (lobular capillary hemangioma) – a common reactive/vascular gingival nodule.  Key microscopic features seen in the three fields  Image Magnification-level impression Salient findings that support the diagnosis  1 – superficial/medium power Surface mucosa & lesion shoulder • Hyperparakeratinised stratified squamous oral epithelium, focally thinned/ulcerated  • Abrupt transition to an underlying, cell-rich vascular proliferation  • Scattered neutrophils & fibrin on the surface – typical of a recently traumatised pyogenic granuloma  2 – low power Entire nodule • Well-circumscribed mass composed of lobules of tissue that stain deeper purple, separated by delicate pale fibrous septa (classic lobular architecture)  • Each lobule packed with numerous small calibre vascular spaces; deeper zones show more dilated, blood-filled channels  • Overall outline matches a lobular capillary hemangioma rather than a fibrous or giant-cell lesion  3 – high power Vascular core • Closely set capillary-sized vessels lined by plump but bland endothelial cells  • Many lumina packed with erythrocytes; marked extravasated RBCs between vessels → gives the intense red mottling  • Edematous granulation-tissue stroma with mixed inflammatory infiltrate (neutrophils, lymphocytes)  • No multinucleated giant cells, no mineralised material, and no significant atypia – rules out peripheral giant-cell granuloma, ossifying fibroma, true hemangiopericytoma, etc.  Together, the lobular capillary arrangement, profuse small vessels with erythrocyte-filled lumina, fibrous septation, surface ulceration and mixed inflammation are diagnostic of a pyogenic granuloma (lobular capillary hemangioma). |
| 5.35 | Diagnosis: Soft tissue myxoma; Key Microscopic Features: A non-encapsulated lesion composed of mucinous/myxoid matrix lacking collagen, populated by plump stellate-shaped cells. | Histopathological Analysis of the Provided H&E-Stained Images  Single Most Likely Histopathological Diagnosis:  Peripheral Ossifying Fibroma (POF)  Justifying Key Microscopic Features:  Low Magnification (First Image):  Well-circumscribed nodular lesion arising from the gingiva.  Fibrocellular stroma with lobulated architecture.  Multiple peripheral ossified areas appearing as basophilic, calcified foci — suggestive of bone or cementum-like material.  Surface stratified squamous epithelium, often ulcerated or with rete ridges extending into the lesion.  High Magnification (Second Image):  Proliferation of fibroblasts and plump spindle-shaped cells within a collagenous matrix.  Scattered mineralized deposits consistent with bone, cementum, or dystrophic calcifications.  Occasional chronic inflammatory cells, but not a dominant feature.  Presence of immature bone or cementum-like structures, sometimes rimmed by osteoblasts.  Conclusion:  These features strongly support a diagnosis of Peripheral Ossifying Fibroma, a common reactive gingival lesion believed to originate from the periodontal ligament. It typically presents as a firm, fibrous mass with areas of calcification or ossification, often in the anterior maxilla. | Histopathological diagnosis – Giant cell fibroma (oral fibrous gingival nodule)  Key microscopic features that support the diagnosis  Field Observation Diagnostic relevance  Overall architecture (low-power) • Well-circumscribed, pedunculated/exophytic nodule covered by stratified squamous mucosa.  • Surface epithelium shows elongated, papillary rete ridges projecting into the lesion. Polypoid, papillary profile typical of reactive fibrous gingival nodules; giant-cell fibroma commonly displays a papillary surface.  Connective-tissue stroma • Stroma is loose and myxoid, composed of delicate, haphazard collagen bundles rather than dense scar-type fibrous tissue.  • Only scant chronic inflammatory cells. Separates the lesion from a classic “irritation fibroma”, which is usually densely collagenised; the lightly collagenised, myxoid background is characteristic of giant-cell fibroma.  Fibroblastic cells (high-power) • Numerous large, stellate-shaped fibroblasts with long dendritic cytoplasmic processes spread through the stroma.  • Many of these cells contain two or more nuclei (true multinucleated “giant” fibroblasts). The presence of abundant stellate and multinucleated fibroblasts is the defining microscopic criterion for a giant-cell fibroma and is absent in other common gingival fibrous lesions.  Additional features • No significant vascular proliferation (rules out pyogenic granuloma).  • No calcified/ossified foci (rules out peripheral ossifying fibroma).  • No peripheral giant cells or hemorrhagic zones (rules out peripheral giant-cell granuloma). Helps narrow the differential diagnosis within the “nodules, fibrous and gingival” category.  The combination of a papillary mucosal nodule, a loose myxoid connective tissue background, and numerous stellate and multinucleated fibroblasts is diagnostic of giant cell fibroma. |
| 5.36 | Diagnosis: Soft tissue angiomyxoma of the oral mucosa; Key Microscopic Features: Mucinous stroma containing spindle cells and dilated vessels infiltrating skeletal muscle fibers, with spindled and stellate fibroblasts embedded in the myxoid matrix. | Based on the hematoxylin-and-eosin (H&E) stained histological images you provided, which are from different magnifications of the same lesion in the "Nodules, Fibrous and Gingival" category of oral and maxillofacial pathology, the single most likely histopathological diagnosis is:  Peripheral Ossifying Fibroma (POF)  Key Microscopic Features Supporting the Diagnosis:  Fibrous Stroma:  The lesion shows a highly cellular fibrous connective tissue stroma with plump spindle-shaped fibroblasts, consistent with a reactive fibroblastic proliferation.  Osteoid or Calcified Material:  At higher magnification (second image), there are scattered basophilic or eosinophilic calcified areas resembling bone or cementum-like material — a hallmark of Peripheral Ossifying Fibroma.  Vascularity and Inflammation:  The lesion shows areas of vascular proliferation and mild chronic inflammatory infiltrate, which are often present in reactive gingival lesions.  Surface Epithelium (low power image):  Although not the main focus, the left side of the low magnification image appears to show surface epithelium, possibly hyperplastic, which is consistent with a peripheral (gingival) lesion.  Well-Demarcated but Unencapsulated:  The lesion appears well-circumscribed yet not encapsulated, another feature typical of POF.  Summary:  Peripheral Ossifying Fibroma is a common reactive gingival lesion, particularly seen in younger individuals and females. The histologic presence of cellular fibrous tissue interspersed with mineralized material (bone or cementum-like) is diagnostic. | Histopathological diagnosis (single best):  Superficial (oral) angiomyxoma / soft-tissue myxoma  Key microscopic features that support this diagnosis  Feature seen in the photomicrographs Diagnostic implication  Abundant loose, pale-staining myxoid (muco-polysaccharide-rich) extracellular matrix occupying almost the whole lesion Hallmark of a myxomatous tumour  Hypocellular stroma populated by scattered spindle- or stellate-shaped fibro-/myofibroblasts with small, bland, hyperchromatic nuclei and inconspicuous nucleoli; mitoses absent Benign myxoid lesion rather than a cellular/reactive process  Numerous thin-walled, curvilinear and arborising blood vessels that ramify through the matrix (“angiomyxoid” pattern) Characteristic vascular component of superficial angiomyxoma  Delicate, wispy collagen bundles interspersed in the myxoid ground substance Typical of soft-tissue myxoma; differentiates from inflammatory/granulation tissue  Well-circumscribed but unencapsulated interface with surrounding fibrous tissue and minor salivary glands/skeletal muscle (left side of low-power field) Consistent with a superficially located, slow-growing benign nodule  Absence of lobules of adipocytes, giant cells, odontogenic epithelium, calcifications or marked cellular atypia/pleomorphism Helps exclude lipomatous, giant-cell, odontogenic, ossifying or malignant myxoid lesions  Together, the constellation of a hypocellular myxoid stroma, plentiful delicate vasculature and scattered bland spindle/stellate cells in a superficial gingival nodule is diagnostic of superficial angiomyxoma (oral soft-tissue myxoma) within the “fibrous & gingival nodule” category of oral and maxillofacial pathology. |

VASCULAR,NEURAL,ADIPOCYTIC,MUSCLE,AND MYOFIBROBLASTIC TUMORS

| **Fig_no** | **Ground truth** | **GPT 4o answer** | **o3 answer** |
| --- | --- | --- | --- |
| 6.2 | Diagnosis: Capillary hemangioma with lobular formation; Key Microscopic Features: demonstrates a lobular arrangement composed of numerous dilated capillary channels, along with proliferating endothelial cells lining these expanded vascular regions. | Based on the histopathological examination of the two provided H&E-stained images from a lesion in the category of vascular, neural, adipocytic, muscle, and myofibroblastic tumors of the oral and maxillofacial region, the single most likely histopathological diagnosis is:  Malignant Peripheral Nerve Sheath Tumor (MPNST)  Key Microscopic Features Justifying the Diagnosis:  Low-Power View (First Image):  Lobulated architecture with intersecting fascicles and areas of geographic necrosis.  The lesion shows hypercellular areas separated by fibrous septa, with infiltrative margins.  Evidence of perivascular accentuation of tumor cells, a feature common in high-grade sarcomas, including MPNST.  High-Power View (Second Image):  Spindle-shaped cells arranged in fascicles with wavy, hyperchromatic nuclei—a hallmark of nerve sheath differentiation.  High mitotic activity is observed, consistent with a malignant process.  Nuclear atypia and pleomorphism, further indicating a high-grade tumor.  Some areas may show necrosis and hemorrhage, common in MPNST.  Absence of epithelial differentiation or clear features of other mesenchymal tumors like liposarcoma or leiomyosarcoma.  Why MPNST is Most Likely:  The tumor displays neural differentiation (spindle cells with wavy nuclei).  It is malignant, given the mitotic activity, nuclear atypia, and necrosis.  It lacks features typical of muscle (e.g., cross striations), adipocytic (lipoblasts), or myofibroblastic (zonation, myxoid areas) tumors.  Location in oral/maxillofacial soft tissue is consistent with neural tumors, especially if associated with a nerve or neurofibroma background. | Diagnosis (single best answer)  Benign myofibroma (solitary myofibroma / infantile-type myofibromatosis)  Key microscopic features supporting the diagnosis (visible in the two H&E fields)  Field Feature seen Significance  Low-power overview • Sharply demarcated, multilobular mass divided by broad, pale-pink, collagenous septa  • Alternating dark (hyper-cellular) and light (more sclerotic) zones within the lobules  • Numerous branching, slit-like to “staghorn” thin-walled vascular channels coursing through the lesion Typical growth pattern of myofibroma: lobular architecture with a biphasic “light-and-dark” appearance and a prominent hemangiopericytoma-like vascular network  High-power detail • Uniform plump spindle-to-ovoid cells with scant, pale eosinophilic cytoplasm and indistinct cell borders  • Nuclei round/oval, finely chromatic, inconspicuous nucleoli, minimal pleomorphism  • Concentric whorling and tight streaming of tumor cells around small vessels (“perivascular myoid” pattern)  • Scattered extravasated red cells, scant mitoses, no necrosis Cytology and vascular wrapping pattern are characteristic of myofibroblastic (pericytic-myoid) differentiation and help distinguish myofibroma from other spindle-cell tumors of the oral–maxillofacial region  Together, the lobulated biphasic architecture, hemangiopericytoma-like vessels, and bland perivascular myofibroblasts are diagnostic of a myofibroma within the spectrum of vascular–myofibroblastic tumors encountered in oral and maxillofacial pathology. |
| 6.3 | Diagnosis: Lobular capillary hemangioma affecting the lip; Key Microscopic Features: exhibits a diffuse overgrowth of endothelial cells forming widened capillary spaces, shows capillary channels with varying degrees of dilation, and presents benign-appearing endothelial cells with a central mitotic figure. | Based on the three provided hematoxylin and eosin (H&E) stained histopathological images at low, intermediate, and high magnifications, the single most likely histopathological diagnosis is:  Myofibroma  Key Microscopic Features Supporting the Diagnosis:  1. Low Magnification (Image 1):  Well-circumscribed but unencapsulated dermal/submucosal lesion.  Overlying epithelium appears intact.  Tumor has a biphasic appearance with more cellular and less cellular (fibrous or myxoid) zones.  No infiltrative border—consistent with a benign process.  2. Intermediate Magnification (Image 2):  Biphasic pattern with alternating areas:  Cellular fascicles of spindle-shaped cells.  More myxoid or collagenous areas with reduced cellularity.  Presence of hemangiopericytoma-like vascular pattern (staghorn or branching vessels), which is characteristic of myofibroma.  3. High Magnification (Image 3):  Spindle to oval myoid cells with pale eosinophilic cytoplasm.  Bland, uniform nuclei without significant atypia.  Scattered mitotic figures may be present but are not atypical.  Plump perivascular myoid cells surrounding thin-walled branching vessels.  Occasional areas of hyalinization or collagen deposition.  Summary:  The histologic pattern seen across the magnifications is classic for myofibroma, a benign myofibroblastic tumor. The presence of a well-demarcated lesion, hemangiopericytoma-like vasculature, and spindle cell morphology with a biphasic growth pattern is diagnostic. These tumors are common in children and young adults and can occur in the oral cavity, especially in the mandible or soft tissue. | Proposed histopathological diagnosis  Lobular capillary hemangioma ( = “pyogenic granuloma” ) of the oral mucosa  Key microscopic features visible in the three H-E photographs  Low / medium / high power finding How it points to lobular capillary hemangioma  Subepithelial, well-circumscribed, dome-shaped nodule lying immediately beneath a focally ulcerated stratified squamous epithelium (first image) Typical growth pattern of pyogenic granuloma: a polypoid mass arising from the lamina propria, often covered by ulcer and fibrin because of repeated trauma/bleeding.  The lesion is divided into multiple lobules by delicate fibrous septa; each lobule is packed with numerous small, thin-walled capillary-type vessels (first & second images) “Lobular” architecture is the defining feature of lobular capillary hemangioma and separates it from ordinary granulation tissue or other vascular tumours.  Within each lobule, the vessels are lined by plump but bland endothelial cells; there is no cytologic atypia or infiltrative pattern (second & third images) Confirms a benign capillary proliferation rather than an angiosarcoma or malignant vascular lesion.  Abundant erythrocyte-filled lumina and extravasated red blood cells/hemosiderin scattered through the stroma (second & third images) Accounts for the clinically prominent bleeding of pyogenic granuloma and is expected in this lesion.  Background stroma is edematous to myxoid with scattered mixed inflammatory cells (mainly neutrophils) (second & third images) Reflects the surface ulceration and reactive nature of the lesion; inflammation is common in pyogenic granuloma but uncommon in many other vascular tumours.  Overall cellularity is high but mitoses are rare and normal, and there is no encapsulation but a pushing, non-infiltrative border (all images) Benign biologic behaviour consistent with pyogenic granuloma.  Taken together, the combination of a superficial ulcerated mass, a lobular arrangement of capillary-sized vessels, plump but bland endothelia, abundant RBCs, and a reactive inflammatory backdrop is diagnostic of a lobular capillary hemangioma (pyogenic granuloma) in the oral cavity. |
| 6.4 | Diagnosis: Capillary hemangioma with lobular architecture; Key Microscopic Features: shows an ulcerated polypoid lesion, contains a retiform network of vascular channels, and displays moderate atypical changes in the endothelial cells. | Based on the histological images provided at low, medium, and high magnification, the features are highly suggestive of:  Diagnosis:  Angiomatoid Fibrous Histiocytoma (AFH)  Key Microscopic Features Supporting the Diagnosis:  Low Magnification (First Image):  Well-circumscribed, lobulated lesion often with a fibrous pseudocapsule.  Presence of blood-filled cystic spaces or hemorrhagic areas, characteristic of the “angiomatoid” component.  Mixed solid and cystic growth pattern.  Intermediate Magnification (Second Image):  Tumor composed of spindle to ovoid cells arranged in solid sheets or fascicles.  Presence of pseudoangiomatous spaces (blood-filled spaces not lined by endothelium).  Areas of dense lymphoplasmacytic infiltrate, often forming a cuff around the lesion.  High Magnification (Third Image):  Round to spindle-shaped tumor cells with eosinophilic cytoplasm and vesicular nuclei.  Multinucleated and bizarre cells may be seen, but mitotic figures are usually low.  Prominent pericapsular chronic inflammation, sometimes with germinal center formation.  Corroboration with Tumor Group:  This lesion fits within the "myofibroblastic tumors" category among oral and maxillofacial tumors. AFH is rare in the oral cavity but recognized in the broader group of soft tissue tumors with overlapping myofibroblastic and histiocytic features. | Histopathological diagnosis (single best):  Pyogenic granuloma / Lobular capillary hemangioma  Key microscopic features that support this diagnosis  Field Observations in the submitted slides Diagnostic value  Low-power architecture • Pedunculated/ polypoid mass beneath oral squamous epithelium, with a focally ulcerated surface crater.  • Lesion is well-circumscribed and composed of innumerable small empty or blood-filled spaces that form discrete lobules separated by thin fibrous septa (“lobular” pattern). The lobulated capillary proliferation beneath (and often eroding) the mucosa is the signature of a lobular capillary hemangioma.  Vascular component • Capillary-sized, round-to-slit-like lumina.  • Lined by plump endothelial cells that bud into—and sometimes contain—erythrocytes (intracytoplasmic lumina). A dense, fast-growing capillary network with epithelioid endothelial cells is typical for pyogenic granuloma rather than ordinary capillary/cavernous hemangioma or smooth-muscle tumours.  Stroma • Delicate, oedematous / myxoid stroma between lobules in the superficial part; becoming more collagenised deeper down. “Maturing” fibromyxoid stroma is characteristic of older pyogenic granulomas.  Inflammation & haemorrhage • Extravasated red blood cells and scattered mixed inflammatory cells throughout the lesion. Frequent bleeding and secondary inflammation are hallmarks of this reactive vascular lesion.  Cytology / atypia • Endothelial nuclei are round-to-oval and uniform; mitoses may be present but no cytological atypia or multilayering. Confirms a benign, reactive lesion rather than an angiosarcoma or epithelioid hemangioendothelioma.  Taken together—the polypoid mucosal mass, focal ulceration, a sharply lobulated proliferation of capillary vessels lined by plump but bland endothelial cells, set in variably fibromyxoid stroma with haemorrhage—the findings are classic for pyogenic granuloma (lobular capillary hemangioma) in the oral cavity. |
| 6.7 | Diagnosis: Lip varices; Key Microscopic Features: shows markedly dilated venules; vascular spaces are lined by a single layer of endothelial cells with uneven thickness of the surrounding muscle coat. | Histopathological Diagnosis:  Hemangiopericytoma / Myopericytoma (spectrum of perivascular myoid cell neoplasms)  Key Microscopic Features Justifying the Diagnosis:  Prominent Staghorn-like Vascular Pattern:  The low-power image shows numerous thin-walled, branching blood vessels with a “staghorn” or antler-like configuration, which is characteristic of hemangiopericytoma-like vascular patterns.  These vessels are surrounded by concentric layers of spindle-to-oval cells.  Perivascular Myoid Cell Proliferation:  The higher magnification reveals bland, spindle-shaped cells arranged concentrically around blood vessels. These cells have eosinophilic cytoplasm and elongated nuclei with minimal atypia and low mitotic activity.  This perivascular arrangement is a hallmark of pericytic neoplasms (e.g., myopericytoma, hemangiopericytoma).  Lack of Significant Atypia or Necrosis:  The lesion appears cytologically bland, with no features suggestive of high-grade malignancy.  No necrosis, significant pleomorphism, or increased mitoses are identified.  Collagenous Stroma:  A variable amount of collagen is present between tumor cells, sometimes imparting a myxoid or hyalinized quality.  Differential Diagnosis Considerations (But Less Likely):  Solitary Fibrous Tumor (SFT): Also shows staghorn vessels but typically has a patternless architecture and may show “ropey” collagen.  Angioleiomyoma: Also perivascular but with more mature smooth muscle bundles.  Glomus Tumor: Shows round, uniform cells with a more solid growth pattern, not the perivascular concentric arrangement. | Histopathological diagnosis (single best):  Cavernous haemangioma (venous-type vascular malformation) of the oral mucosa  Key microscopic features supporting the diagnosis  Area of slide What is seen Diagnostic implication  Low-power overview • Intact, non-ulcerated stratified squamous epithelium overlying a well-circumscribed but non-encapsulated submucosal mass.  • Lesion is dominated by numerous, variably sized, large blood-filled spaces separated by thin fibro-collagenous septa. Large calibre vascular channels favour a cavernous form rather than a capillary haemangioma.  Vascular spaces • Lumina packed with erythrocytes; occasional organising thrombus.  • Walls are delicate, composed of a single layer of flattened endothelial cells with minimal smooth-muscle support. Confirms the lesion is vascular and benign; absence of multilayering, tufting or atypia excludes angiosarcoma and Kaposi sarcoma.  Intervening stroma • Scant, collagen-rich connective tissue containing bland, elongated spindle cells (fibroblasts/smooth muscle cells).  • Whorled arrangement in places, but no significant cytologic atypia or mitotic activity. Benign supporting stroma typical of haemangioma; rules out cellular myofibroblastic or neural neoplasms.  Additional findings • Focal extravasation of red cells and hemosiderin deposition.  • No adipocytes, Verocay bodies, Antoni patterning, or skeletal-muscle bundles. Helps exclude lipoma, schwannoma, leiomyoma or rhabdomyoma.  Collectively, the presence of large, blood-filled cavernous vascular channels lined by bland endothelium underneath intact oral epithelium—with only sparse fibrous stroma and no significant cellular atypia—points squarely to a cavernous haemangioma / venous malformation. |
| 6.8 | Diagnosis: Lip varices; Key Microscopic Features: demonstrates distended, congested venules; exhibits expanded, endothelium-lined spaces featuring valve leaflets and a thin muscular coat. | Histopathological Diagnosis:  Masson's Hemangioma (Intravascular Papillary Endothelial Hyperplasia, IPEH)  Key Microscopic Features Justifying the Diagnosis:  Intravascular Location:  Both low- and high-power views show a lesion entirely contained within a dilated vascular space, a hallmark of IPEH.  The periphery of the lesion is lined by flattened endothelium, consistent with a pre-existing vascular lumen.  Papillary Structures with Fibrin Core:  There are multiple papillary fronds projecting into the vascular lumen.  These fronds are composed of a fibrin core (pale eosinophilic) covered by a single layer of bland endothelial cells.  Lack of Atypia or Mitotic Activity:  Endothelial cells lining the papillary projections appear bland and uniform with no nuclear atypia or mitotic figures.  This supports a benign, reactive process rather than a malignant vascular neoplasm (e.g., angiosarcoma).  Association with Thrombus:  The lesion shows areas of organizing thrombus, which is a key feature of Masson’s tumor — often thought to arise as an organizing process within a thrombus.  No Infiltration into Surrounding Tissues:  The lesion remains well circumscribed, without evidence of infiltration into adjacent soft tissue, muscle, or fat — another benign indicator.  Summary:  These features point strongly to Masson’s Hemangioma (IPEH), a benign, reactive vascular lesion often mistaken for angiosarcoma histologically. Recognition of its intravascular, well-organized papillary architecture and bland cytology is key for accurate diagnosis. | Histopathological diagnosis  → Cavernous (venous-type) hemangioma of the oral mucosa  Key microscopic features that point to this diagnosis  # Feature seen in the photomicrographs Diagnostic significance  1 Well-circumscribed, lobulated mass immediately beneath an intact stratified squamous mucosa Typical location/pattern for a benign vascular lesion rather than an infiltrative malignancy.  2 Multiple, very large blood-filled spaces (“caverns”) that occupy almost the whole lesion; in several areas the erythrocytes gravitate to the periphery leaving pale central lumina Cavernous hemangiomas are defined by dilated vascular channels rather than the tiny capillary loops of a lobular capillary hemangioma/“pyogenic granuloma.”  3 Each cavern is lined by a single layer of flattened endothelial cells; there is no endothelial piling, multilayering, atypia or mitotic activity Confirms a benign vascular malformation, excluding angiosarcoma or Kaposi sarcoma.  4 Thin fibrous connective-tissue septa separate adjacent vascular spaces and form the lobulated outline seen at low power Helps distinguish the lesion from a simple vascular ectasia and from spindle-cell vascular tumours (e.g., angioleiomyoma, myopericytoma).  5 Scant, unremarkable stroma with only a mild chronic inflammatory infiltrate; no admixture of smooth/skeletal muscle, nerve, or adipose elements Rules out mixed hamartomatous lesions or myofibroblastic tumours.  Taken together, the combination of large, blood-packed, thin-walled vascular spaces, their benign endothelial lining, and the lobulated but non-infiltrative growth pattern is classic for a cavernous hemangioma (venous malformation) in the oral and maxillofacial region. |
| 6.12 | Diagnosis: Venous malformation; Key Microscopic Features: shows numerous dilated, congested venules; the vascular channels have a thin muscular layer; valve leaflets are identifiable within these spaces. | Histopathological Analysis of the Provided H&E-Stained Images  Single Most Likely Diagnosis:  Hemangioma (Capillary or Cavernous Type)  Key Microscopic Features Supporting the Diagnosis:  Numerous Vascular Spaces:  All three images consistently show multiple, variably sized, endothelial-lined vascular spaces throughout the lesion. These range from small capillary-like channels to larger, dilated, blood-filled cavernous spaces.  Endothelial Cell Lining:  The vascular channels are lined by a single layer of flattened endothelial cells, without significant cytologic atypia or mitotic activity—typical of benign vascular proliferations.  Blood Content:  The vascular spaces in images 2 and 3 are filled with erythrocytes, confirming their vascular nature.  Lobular Growth Pattern:  The low-power image (image 1) shows a lobular architecture, a hallmark of some types of hemangiomas (especially lobular capillary hemangiomas).  Lack of Infiltrative Behavior or Atypia:  There is no evidence of invasive growth, cellular atypia, or necrosis—important in differentiating this benign entity from angiosarcoma or other malignant vascular tumors.  Absence of Significant Stromal Components:  The lesion is almost entirely composed of vascular structures, with minimal fibrous or adipocytic stroma, arguing against other mixed mesenchymal tumors.  Conclusion:  The combination of lobular arrangement, dilated blood-filled spaces, and benign endothelial lining supports a diagnosis of hemangioma, likely of the cavernous or mixed capillary-cavernous subtype, depending on the relative proportion of large versus small vessels. | Histopathological diagnosis  → Cavernous haemangioma (venous-type vascular malformation)  Key microscopic features that support the diagnosis  Field What is seen How it points to cavernous haemangioma  Low-power architecture Subepithelial mass composed of innumerable, back-to-back vascular spaces that occupy almost the entire lamina propria and upper submucosa, pushing but not infiltrating surrounding tissue. A purely vascular proliferation without solid tumour stroma is characteristic of haemangiomas/vascular malformations. The broad, sponge-like arrangement of dilated channels is typical of the cavernous type.  Size & shape of channels Large, irregular, anastomosing, “cavernous” lumina of very variable calibre; many measure several red-cell diameters across. Capillary haemangiomas show small, uniform vessels; cavernous lesions show wide, sinus-like spaces.  Luminal content Spaces are packed with erythrocytes; some contain clotted blood or appear partly evacuated, producing empty or partially filled cavities. Blood-filled spaces confirm a vascular (rather than lymphatic) nature. The mixture of full and empty channels is common in cavernous haemangioma.  Endothelial lining Single layer of flattened, bland endothelial cells; nuclei are small, elongated and lack atypia or mitotic figures. Benign endothelial lining distinguishes the lesion from angiosarcoma or other malignant vascular neoplasms.  Intervening stroma/septa Very thin fibrous septa and delicate connective-tissue trabeculae; occasional slender, papillary projections extend into lumina (best seen on higher magnification). Fibrous strands and intraluminal bridging are typical supporting structures in cavernous haemangiomas.  Surface epithelium Intact stratified squamous oral mucosa with no dysplasia; lesion sits immediately beneath it. Location and lack of epithelial involvement support a submucosal vascular lesion rather than an epithelial tumour.  Collectively, the presence of numerous dilated, RBC-filled vascular spaces with flat, bland endothelial lining and thin fibrous walls, arranged in a sponge-like network beneath intact oral mucosa, is diagnostic of a cavernous haemangioma (venous malformation). |
| 6.13 | Diagnosis: Venous malformation; Key Microscopic Features: features venules of heterogeneous sizes occupying both superficial and deep lamina propria; vessels exhibit variable muscular wall thickness. | Based on the hematoxylin-and-eosin (H&E) stained histopathology images provided — a low-power overview and a higher magnification view — the most likely single histopathological diagnosis is:  Sialolipoma  Key Microscopic Features Supporting This Diagnosis:  Well-Circumscribed, Lobulated Lesion (seen in the low-power image):  The lesion appears to be well-demarcated and located in the submucosa.  The overlying epithelium is intact, suggesting a benign process.  Mature Adipose Tissue:  The bulk of the lesion consists of lobules of mature adipocytes, which are uniform in size and shape.  This is a hallmark of lipomatous tumors.  Entrapped Normal Salivary Gland Elements (seen in the higher-power image):  Numerous ductal and acinar structures resembling salivary glands are seen within the adipocytic component.  These epithelial structures are not atypical and are distributed within the fatty stroma.  This distinguishes sialolipoma from conventional lipomas, which lack salivary elements.  Delicate Fibrous Septa and Vascular Channels:  The lobules of fat are separated by thin fibrous septa.  Small vascular channels and myxoid stroma may be present.  Absence of Cytologic Atypia or Mitotic Activity:  The lesion lacks features of malignancy such as pleomorphism, necrosis, or high mitotic activity.  Summary:  Sialolipoma is a rare benign tumor composed of mature adipose tissue and normal salivary gland elements. It is typically well-circumscribed and found in the oral cavity, particularly the palate, buccal mucosa, and floor of mouth. | Histopathological diagnosis (single best fit)  Vascular leiomyoma (angioleiomyoma), venous-type  Key microscopic features that support the diagnosis  Level of magnification Findings Diagnostic relevance  Low power • Well-circumscribed, submucosal nodule lying immediately beneath intact stratified squamous epithelium.  • Lesion is composed of innumerable round-to-oval vascular profiles of fairly uniform calibre set in an eosinophilic stroma. Indicates a benign, non-infiltrative vascular-smooth-muscle tumour rather than a diffuse malformation or invasive sarcoma.  Intermediate / high power • Each vascular space is lined by a single layer of flattened endothelial cells and contains erythrocytes.  • Surrounding every lumen is a thick concentric mantle of spindle cells with brightly eosinophilic cytoplasm, arranged in smooth, interlacing bundles (“onion-skin” or cuff-like wall).  • The spindle cells have blunt, cigar-shaped nuclei, absent pleomorphism, and rare/absent mitoses.  • Between vascular profiles the same smooth-muscle bundles intersect, merging one vessel wall with the next. The concentric, compact smooth-muscle cuffs and inter-fascicular pattern are classic for a vascular leiomyoma (particularly the venous subtype) and separate it from:  – Myopericytoma (perivascular cells much thinner, tapering, with little intervening fasciculation)  – Hemangioma/angiomatous malformation (minimal or no muscular wall)  – Solitary fibrous tumour / “hemangiopericytoma” (staghorn branching vessels without true muscular collars).  Cytology / stroma • Bland spindle cells, no necrosis, no atypical mitotic figures.  • Collagenised but otherwise non-myxoid stroma. Confirms benign smooth-muscle phenotype and excludes leiomyosarcoma or myofibrosarcoma.  Collectively, the abundant thick muscular coats around patent vascular lumina, the interlacing smooth-muscle fascicles between vessels, and the overall circumscription point to a vascular leiomyoma (angioleiomyoma) as the single most appropriate diagnosis for this oral soft-tissue lesion. |
| 6.16 | Diagnosis: Lymphatic dilation (lymphangioma circumscriptum); Key Microscopic Features: comprises a distinct cluster of enlarged lymphatic spaces in both superficial and deep lamina propria that abut the overlying epithelium; spaces are lined by a single layer of endothelial cells and filled with pale, wispy lymph. | Histopathological Diagnosis:  Lymphangioma  Key Microscopic Features Justifying the Diagnosis:  Multiloculated Cystic Spaces:  Seen prominently across all magnifications, especially in the low-power view.  The cystic spaces are irregular, thin-walled, and interconnected—typical of a lymphangioma.  Endothelial Lining:  The spaces are lined by a single layer of flat endothelial cells, as seen clearly in the high-power image.  These cells are bland and lack atypia or mitotic activity.  Eosinophilic Proteinaceous Material and Lymph:  The cystic spaces contain pale, eosinophilic, proteinaceous fluid with scattered lymphocytes, consistent with lymphatic content.  Lack of Significant Blood Content:  Unlike hemangiomas, the vascular spaces do not appear to contain red blood cells, reinforcing a lymphatic origin.  Overlying Epithelium:  The overlying stratified squamous epithelium is intact and not dysplastic, common in oral mucosal lymphangiomas.  Loose Fibrous Stroma with Lymphocytic Aggregates:  The supporting connective tissue contains sparse inflammatory cells and fibrovascular stroma, which is a common background in lymphangiomas.  These features are diagnostic of lymphangioma, a benign lymphatic malformation frequently seen in the head and neck region, including the oral cavity. | Histopathological diagnosis  → Superficial lymphatic malformation (“lymphangioma circumscriptum”)  Key microscopic features supporting this diagnosis  Feature (with magnification reference) Diagnostic significance  1. Broad, sub-epithelial proliferation of multiple, irregular, empty-looking spaces (low-power panorama) Represents markedly dilated lymphatic channels occupying the papillary and upper reticular lamina propria.  2. Extremely thin, eosinophilic walls containing only scattered flattened nuclei (intermediate and high power) The attenuated endothelial lining is typical of lymphatic rather than blood-vascular spaces; there is virtually no smooth-muscle or elastic tissue in the walls.  3. Little or no intraluminal red blood cells; lumina contain pale proteinaceous fluid ± a few lymphocytes (high power) Absence of erythrocytes helps distinguish lymphatic channels from haemangiomatous vessels.  4. Overlying stratified squamous epithelium sends elongated rete ridges that dip between the dilated channels (intermediate magnification) “Wedge-shaped” epithelial extensions are characteristic of superficial lymphatic malformations involving the mucosa/skin.  5. Loose fibrous septa with small aggregates of lymphocytes between channels (high power) Lymphoid cuffing is frequently seen in lymphangiomas.  6. Lesion is unencapsulated and blends imperceptibly with the surrounding connective tissue Supports a developmental/hamartomatous vascular malformation rather than a true neoplasm.  The combination of numerous, superficially situated, thin-walled, bloodless vascular spaces lined by flattened endothelial cells directly beneath the mucosal epithelium is classic for a lymphangioma of the superficial (circumscribed) type and rules out adipocytic, neural, myogenic or hemangiomatous alternatives. |
| 6.17 | Diagnosis: Epithelioid hemangioma; Key Microscopic Features: appears as a well-circumscribed multilobular cellular nodule with centrally dilated large vascular channels; contains numerous capillaries, epithelioid cells, and lymphocytes; epithelioid endothelial cells exhibit large, benign nuclei with small nucleoli. | Based on your uploaded hematoxylin and eosin (H&E) stained images at low, intermediate, and high magnification, and considering the lesion falls within the category of vascular, neural, adipocytic, muscle, and myofibroblastic tumors of the oral and maxillofacial region, the most likely histopathological diagnosis is:  Schwannoma (Neurilemmoma)  Key Microscopic Features Supporting the Diagnosis:  1. Low magnification (first image):  Well-circumscribed, encapsulated lesion: Indicates a benign, slow-growing neoplasm.  Biphasic pattern: Presence of alternating hypercellular (Antoni A) and hypocellular (Antoni B) areas, characteristic of schwannomas.  2. Intermediate magnification (second image):  Antoni A areas: Densely cellular regions with spindle-shaped Schwann cells arranged in fascicles.  Verocay bodies: Nuclear palisading around eosinophilic, acellular zones — a hallmark of Antoni A areas.  Antoni B areas: Less cellular, more myxoid with a looser stroma, consistent with degenerative changes.  3. High magnification (third image):  Spindle cells with elongated, wavy nuclei: Typical Schwann cell morphology.  Lack of mitotic activity or atypia: Supports benign behavior.  Thick-walled hyalinized vessels: Often seen in schwannomas.  Foamy macrophages and vascular proliferation: Degenerative features sometimes referred to as “ancient” change.  Differential diagnosis (less likely based on features):  Neurofibroma: Typically lacks encapsulation and Verocay bodies.  Leiomyoma: Shows smooth muscle differentiation without nuclear palisading.  Myofibroma: More hemangiopericytoma-like vascular pattern, often lacks Antoni A/B regions. | Histopathological diagnosis  Solitary myofibroma ( myofibroma of the oral cavity) – a benign myofibroblastic tumour that belongs to the “muscle / myofibroblastic” subset of oral soft-tissue tumours.  Key microscopic features that support this diagnosis  Magnification Salient finding Diagnostic significance  Low-power (first slide) • A well-circumscribed, lobulated submucosal nodule sitting beneath intact stratified squamous epithelium.  • Lesion is composed of two to three tightly packed cellular lobules separated by delicate fibrous septa, giving a nodular / biphasic architecture. A sharply outlined but unencapsulated, lobulated growth is typical of myofibroma; the biphasic pattern (cell-rich centre, more collagenised periphery) is a hallmark.  Intermediate power (second slide) • Within each lobule the cells form whorled and concentric fascicles around numerous thin-walled branching (“staghorn”) vessels, creating a hemangiopericytoma-like pattern.  • Widespread extravasated erythrocytes and small foci of haemorrhage are present. Concentric perivascular proliferation of spindle / ovoid myofibroblasts with conspicuous RBC leakage is characteristic of myofibroma and helps separate it from smooth-muscle tumours (which show thick-walled vessels) and neural tumours (which lack the perivascular pattern).  High-power (third slide) • Cells are plump spindle to ovoid with pale amphophilic cytoplasm and tapered ends; nuclei are vesicular, uniform and lack atypia.  • Delicate collagen and occasional hyalinised matrix separate the cells.  • Mitoses are infrequent and normal in appearance. Cytomorphology of myofibroblasts (intermediate between fibroblast and smooth-muscle cell) with absence of significant atypia or necrosis confirms the benign myofibroblastic nature of the lesion. The mixture of immature round/ovoid cells centrally and more differentiated spindle cells peripherally produces the classic biphasic pattern.  Why other entities are unlikely  Angioleiomyoma / vascular leiomyoma – would show bundles of cigar-shaped smooth-muscle cells and thick muscular vessel walls, not the thin-walled pericytic vessels and RBC extravasation seen here.  Nodular fasciitis – usually poorly circumscribed, lacks the lobulated/biphasic architecture and hemangiopericytoma-like whorling pattern.  Neural tumours (schwannoma, neurofibroma) – show Antoni A/B areas, Verocay bodies or wavy collagen bundles, none of which are present.  Taken together, the nodular outline, central pericytic whorls of uniform myofibroblasts around branching vessels, peripheral spindle-cell collar, and abundant extravasated erythrocytes point squarely to solitary myofibroma. |
| 6.18 | Diagnosis: Kimura disease; Key Microscopic Features: shows fibrotic stroma accompanied by a dense lymphoid infiltrate containing numerous germinal centers; germinal centers are populated by abundant lymphocytes, plasma cells, and eosinophils. | Based on the histopathological evaluation of the provided H&E-stained images of the lesion (low and high magnification), and considering the context of vascular, neural, adipocytic, muscle, and myofibroblastic tumors in the oral and maxillofacial region, the most likely diagnosis is:  Single Most Likely Histopathological Diagnosis:  Angiolipoma  Key Microscopic Features Supporting the Diagnosis:  Low Magnification (First Image):  Presence of mature adipose tissue (clusters of adipocytes) predominantly in the lower left quadrant.  Scattered capillary-sized blood vessels, some congested, interspersed between fat lobules.  Well-circumscribed lesion, lacking overt infiltrative growth.  A central vessel with thrombotic material (white cleft-like appearance) is suggestive of vascular proliferation or organization.  High Magnification (Second Image):  Numerous small blood vessels intermingled with mature adipocytes.  Evidence of fibrin thrombi or occlusion in some capillaries.  Minimal cellular atypia, consistent with a benign lesion.  Scattered inflammatory cells in the stroma — typical for angiolipoma, which can have inflammatory and thrombotic elements.  Summary:  These features — a mix of mature fat and proliferative vascular elements with thrombi — are characteristic of angiolipoma, a benign lesion in the adipocytic category that shows a prominent vascular component. The lesion is well-circumscribed, non-infiltrative, and composed of benign adipose and vascular tissue, which aligns with the histology seen. | Single best diagnosis  → Kimura disease (eosinophilic lymphoid granuloma / angiolymphoid proliferative lesion of soft tissue).  Key microscopic features seen in the two H-E fields that support this diagnosis  Feature Where you can see it in the submitted figures Diagnostic significance  Lobulated mass composed of numerous reactive lymphoid follicles with well-formed germinal centres Low-power image: multiple round, dark-purple nodules separated by paler stroma Hall-mark of Kimura disease – far more prominent lymphoid component than in an epithelioid haemangioma  Thick mantle (onion-skin) and inter-follicular zones packed with cells Low- and high-power images: mantle zones of small lymphocytes encircling the germinal centres; inter-follicular areas cellular Reflects the exuberant reactive B-cell proliferation characteristic of the lesion  Heavy eosinophil-rich inflammatory infiltrate, sometimes forming micro-abscesses High-power image: numerous cells with bright eosinophilic granular cytoplasm and bilobed nuclei scattered between lymphocytes Dense tissue eosinophilia is a sine-qua-non for Kimura disease and helps separate it from simple lymphoid hyperplasia  Proliferation of thin-walled capillaries and venules lined by flat endothelium (no epithelioid “hob-nailing”) Both magnifications: small vascular lumina coursing through the stromal areas; central white vascular space in the low-power field Vascular proliferation is present, but the endothelial cells are bland and flattened – unlike the plump epithelioid cells of angiolymphoid hyperplasia with eosinophilia (ALHE)  Fibro-hyalinised stroma and extension into adjacent adipose tissue Low-power image: pale pink, sclerotic bands separating the lymphoid lobules; islands of mature fat along the left border Subcutaneous localisation with fibrosis is typical for Kimura disease of the head and neck region  Taken together, the triad of (1) florid reactive lymphoid follicles, (2) abundant eosinophils, and (3) accompanying capillary proliferation in a fibrosclerotic stroma centred in subcutaneous tissue is diagnostic of Kimura disease. |
| 6.20 | Diagnosis: Kaposi sarcoma; Key Microscopic Features: A proliferation of spindle-shaped cells forming dilated vascular channels accompanied by extensive fresh hemorrhage, with spindle cells encircling narrow slit-like spaces demonstrating mild nuclear variability and mitotic figures, and red blood cells present both between the spindle cells and within the lumina. | Histopathological Diagnosis:  Kaposi Sarcoma  Justification & Key Microscopic Features:  The provided H&E-stained histological images (low and high magnification) reveal classic findings consistent with Kaposi Sarcoma (KS), a vascular neoplasm. This tumor is categorized under vascular tumors in the classification of oral and maxillofacial soft tissue neoplasms.  Key Microscopic Features:  Low Magnification (Image 1):  Spindle Cell Proliferation:  Intersecting fascicles of spindle cells dominate the field.  These spindle cells are arranged in a haphazard pattern with a storiform or vaguely fascicular arrangement.  Slit-like Vascular Spaces:  Irregular, angulated, and slit-like vascular spaces are interspersed throughout the lesion.  These mimic neovascular channels and are lined by atypical endothelial cells.  Extravasated Erythrocytes:  Numerous red blood cells are seen outside vascular lumens, indicative of leaky neoplastic vasculature.  Hemosiderin Deposition:  Some foci exhibit hemosiderin-laden macrophages, indicating chronic vascular leakage.  High Magnification (Image 2):  Spindle Cells with Atypia:  The spindle cells have elongated nuclei with mild to moderate atypia.  Mitoses may be seen, though not typically numerous.  Prominent Extravasated RBCs:  Numerous red blood cells scattered between spindle cells—a hallmark of KS.  Mild Inflammatory Infiltrate:  Often includes lymphocytes and plasma cells, surrounding neoplastic areas.  Lack of Adipocytic, Neural, or Muscle Differentiation:  No mature adipocytes, nerve bundles, or striated muscle fibers, helping rule out tumors of those lineages.  Summary:  The presence of spindle cell proliferation with slit-like vascular spaces, extravasated RBCs, and hemosiderin, all within a vascular-rich neoplastic environment, points strongly to Kaposi Sarcoma—a vascular tumor often associated with HHV-8 infection. | Histopathological diagnosis – Kaposi sarcoma, nodular stage (vascular spindle-cell variant).  Key microscopic features visible in the two H-&-E sections  Field Observations How the feature supports Kaposi sarcoma  Low-power overview • Solid, vaguely nodular sheet of uniform spindle cells arranged in intersecting fascicles.  • Numerous jagged, slit-like and ectatic vascular spaces/clefts pepper the lesion; many are packed with erythrocytes, others are empty “ghost-like” lumina.  • Alternating hypercellular and hypocellular areas create a patch-/plaque- to nodular architecture. A spindle-cell proliferation permeated by irregular blood-filled slits is the classic architectural pattern of Kaposi sarcoma.  High-power detail • Spindle cells have elongated, oval nuclei with finely stippled chromatin and scant mitotic activity; cytologic atypia is mild.  • Red-blood-cell extravasation is prominent between tumor cells and within vascular clefts; scattered hemosiderin pigment (not all shown) usually accompanies this.  • Occasional “promontory sign” – small capillary buds protruding into larger ectatic channels.  • Sparse inflammatory cells (lymphocytes/plasma cells) are interspersed. Combination of bland spindle cells, slit-like vascular spaces containing RBCs, and extravasated blood/hemosiderin is virtually pathognomonic for Kaposi sarcoma and distinguishes it from other spindle-cell or vascular lesions (e.g., angiosarcoma shows marked atypia; spindle-cell hemangioma lacks the extensive slit-like pattern).  Together, these features typify Kaposi sarcoma of the oral soft tissues in its fully developed (nodular) stage within the vascular tumor category of oral & maxillofacial pathology. |
| 6.23 | Diagnosis: Traumatic neuroma; Key Microscopic Features: Nerve bundles of various calibers embedded in a fibrotic stroma, with some nerve fibers showing a thickened endoneurial sheath. | Histopathological Analysis of the Provided H&E-Stained Images  Diagnosis:  Perineurioma (soft tissue type)  Justification and Key Microscopic Features:  Spindle Cell Proliferation in Whorled Patterns:  Seen prominently in the second (higher magnification) image.  The lesional cells are elongated and arranged in concentric, whorled, or lamellar structures, a classic pattern of perineurial cell proliferation.  Palisading and Pseudo-onion Bulb Structures:  Cells form perivascular whorls and pseudo-onion bulb formations (reminiscent of perineurial cell layering around axons).  Bland Cytology:  Nuclei are uniform, without atypia or mitoses, suggesting a benign process.  Minimal cytoplasm and absence of pleomorphism also support the benign nature.  Well-Circumscribed Dermal/Submucosal Mass:  The low-power image shows a well-circumscribed but non-encapsulated lesion in the dermis/submucosa, characteristic of soft tissue perineurioma.  No Adipocytic, Vascular, or Muscle Differentiation:  The lesion lacks the features of lipomas, vascular tumors (e.g., hemangiomas), or muscle tumors (e.g., leiomyoma), ruling out other tumor types in this category.  Additional Notes:  Immunohistochemistry (if available) would support this diagnosis:  Positive for EMA (epithelial membrane antigen).  Negative for S-100 (helps differentiate from Schwannoma or neurofibroma). | Histopathological diagnosis (single best):  Vascular leiomyoma (angioleiomyoma) – solid/venous pattern  Key microscopic features that point to this diagnosis  Finding Description in the provided sections Significance  Well-circumscribed, sub-epithelial nodule At low power the lesion forms a dome-shaped mass in the lamina propria/submucosa, sharply demarcated from adjacent connective tissue. The surface stratified squamous epithelium is intact. Typical presentation of a benign soft-tissue tumour such as leiomyoma.  Numerous vascular channels with lumina, some containing erythrocytes Round to oval spaces, one of which clearly shows a cluster of red cells, are evident within the tumour. Defines the lesion as angio-leiomyoma rather than a purely solid smooth-muscle tumour.  Concentric layers (“onion-skin” appearance) of bland spindle cells around each vessel Each vascular space is encased by several smooth-muscle cell layers that are arranged circumferentially, producing a whorled pattern. Characteristic of the solid/venous subtype of vascular leiomyoma.  Uniform spindle cells with eosinophilic cytoplasm and cigar-shaped nuclei On higher magnification the tumour cells are elongated, have tapered ends, finely granular eosinophilic cytoplasm and central, blunt-ended nuclei without pleomorphism. Hallmark cytology of benign smooth-muscle differentiation.  Interlacing fascicles of smooth muscle between vessels Between the perivascular whorls, the same spindle cells form short intersecting bundles separated by collagenous stroma. Common architectural feature of leiomyoma.  Absence of mitoses, necrosis or significant atypia No mitotic figures or nuclear hyperchromasia are seen in the high-power field provided. Confirms benign nature, excluding leiomyosarcoma.  Taken together, the combination of thick-walled vascular channels intimately surrounded and compartmentalised by bland smooth-muscle bundles in a well-circumscribed, submucosal mass is diagnostic of vascular leiomyoma (angioleiomyoma). |
| 6.25 | Diagnosis: Neurofibroma; Key Microscopic Features: Spindle-shaped cells lying in a delicate collagenous matrix, with many of those cells exhibiting comma-shaped nuclei, surrounded by fine collagen fibers and an abundant population of mast cells. | Based on the provided hematoxylin and eosin (H&E) stained histological images of the same lesion, which are consistent with a tumor in the vascular, neural, adipocytic, muscle, and myofibroblastic tumors group in oral and maxillofacial pathology, the single most likely histopathological diagnosis is:  Desmoplastic Fibroma  Key Microscopic Features Supporting This Diagnosis:  Hypocellular to moderately cellular fibrous stroma (seen across low and higher magnification images):  The lesion demonstrates a sparsely to moderately cellular proliferation of spindle cells embedded in a dense collagenous/desmoplastic stroma.  This is a hallmark of desmoplastic fibroma.  Uniform spindle cells with bland, elongated nuclei:  The spindle cells are relatively uniform and lack significant atypia or pleomorphism.  No prominent nucleoli or mitotic figures are seen, consistent with a benign lesion.  Infiltrative growth pattern:  At low magnification, there appears to be entrapment of surrounding normal structures, such as vessels and possibly nerves or glands, indicating an infiltrative nature, despite the bland cytologic appearance.  Absence of lipoblasts or mature adipocytes:  Helps exclude lipomatous tumors.  No evidence of muscular differentiation:  There are no cross-striations, suggesting that a rhabdomyomatous tumor is unlikely.  Lack of prominent eosinophilic cytoplasm seen in smooth muscle tumors further supports this.  Lack of prominent vascular spaces:  Rules out vascular tumors like hemangiomas or angiosarcomas.  Summary:  These histological features—bland spindle cells in a densely collagenous matrix with infiltrative borders—are classic for desmoplastic fibroma, a benign but locally aggressive myofibroblastic tumor. | Histopathological diagnosis (single best):  Solitary neurofibroma  Key microscopic features supporting this diagnosis  Feature How it appears in the submitted H-E photomicrographs  1. Poorly circumscribed, un-encapsulated spindle-cell lesion Low-power image shows the lesion blending imperceptibly with the surrounding connective tissue without a capsule.  2. Paucicellular to moderately cellular stroma with “shredded-carrot” collagen Alternating loose myxoid areas and more collagenised zones; the wavy, eosinophilic collagen bundles create the classic shredded-carrot look (best appreciated in image 3, upper half).  3. Spindle cells with long, comma/wavy nuclei High-power view (image 2) demonstrates slender cells whose nuclei curve or taper at one or both ends—typical of Schwann-cell differentiation.  4. Delicate, thin-walled blood vessels and perivascular collagen cuffing Numerous small round or slit-like vascular lumina are scattered throughout all three magnifications; some are surrounded by concentric collagen.  5. Scattered mast cells Occasional granular, ovoid cells with centrally placed nuclei can be seen in the background stroma (best in image 2), a helpful clue to neurofibroma.  6. Absence of significant mitotic activity, pleomorphism or necrosis Cytology is bland throughout, supporting a benign neural tumour rather than a sarcoma or nodular fasciitis.  The combination of an unencapsulated, intraneural-type spindle-cell proliferation with wavy nuclei, myxoid-collagenous (“shredded carrot”) matrix, scattered mast cells and delicate vasculature is diagnostic of neurofibroma in the oral and maxillofacial region. |
| 6.26 | Diagnosis: Plexiform neurofibroma of tongue in a patient with neurofibromatosis-1; Key Microscopic Features: Tortuous nodular and cordlike aggregates of neural tissue infiltrating the lamina propria and muscle, alongside spindle cells embedded within a delicate collagenous and myxoid stroma, with more cellular regions representing neuritic processes. | Histopathological Diagnosis:  Myofibroma (Solitary Myofibromatosis)  Key Microscopic Features Justifying the Diagnosis:  Low-Power Image (First Image):  Biphasic Pattern: The lesion shows a characteristic biphasic appearance with alternating light and dark staining areas.  Lighter zones correspond to more myxoid and collagenous stroma.  Darker zones are more cellular and compact, showing fascicles or whorled areas.  Nodular growth pattern extending into the submucosa and deeper soft tissue layers.  Overlying mucosa appears intact, suggesting a well-circumscribed but non-encapsulated lesion.  High-Power Image (Second Image):  Spindle-shaped cells with elongated nuclei arranged in interlacing fascicles and whorls.  Minimal atypia and low mitotic activity, which favors a benign process.  Myofibroblastic differentiation: The spindle cells have pale eosinophilic cytoplasm and indistinct cell borders.  Perivascular accentuation: Commonly seen in myofibroma but not clearly visible in this cropped view (though often seen in adjacent higher magnification fields).  Conclusion:  The combination of a biphasic growth pattern, bland spindle cells in fascicular and whorled arrangements, and the involvement of myofibroblastic elements strongly supports the diagnosis of Myofibroma. This is a common lesion in the oral cavity and falls under the category of myofibroblastic tumors in the WHO classification of oral and maxillofacial pathology. | Likely histopathologic diagnosis  Soft-tissue (extraneural) perineurioma of the oral mucosa.  Key microscopic (H-E) features supporting this diagnosis  Low-power features High-power / cytologic details Significance  • A single, well-circumscribed but unencapsulated mass beneath an intact stratified squamous epithelium.  • The tumour is subdivided into multiple pale-pink nodules/whorls separated by delicate fibrous septa. • Lesion composed of uniform, slender, spindle cells with very thin, pale eosinophilic cytoplasm and tapering, “pencil-shaped” nuclei.  • Cells are arranged in concentric lamellae and short fascicles forming onion-skin / whorled or storiform patterns.  • Background stroma is collagenous to slightly myxoid; no keloid-like thick collagen bundles, necrosis, or significant pleomorphism. • A whorled/lamellar growth pattern of bland spindle cells separated by delicate collagen is characteristic of perineurial (not Schwann-cell) differentiation.  • Relative hypocellularity, absence of atypia, and paucity of mitoses favour a benign perineurioma over entities such as myofibroma, nodular fasciitis, or fibromatosis.  Correlative note: Perineurial cells typically express EMA, claudin-1 and GLUT-1 while lacking S-100 protein—immunostains that can be used to confirm the diagnosis if required, but the distinctive lamellar/whorled architecture with bland spindle cells in a collagenous matrix is usually diagnostic on H-E sections. |
| 6.27 | Diagnosis: Dendritic cell neurofibroma; Key Microscopic Features: A well-circumscribed nodule composed of spindle cells; pseudorosette formations featuring peripheral spindle cells and central large pale cells; pseudorosettes made of spindle-type I cells surrounding larger pale type II cells; and the larger pale cells showing positivity for CD57. | Based on the three provided hematoxylin and eosin (H&E) stained histopathological images taken at progressively higher magnifications, the most likely diagnosis is:  Schwannoma (Neurilemmoma)  Key Microscopic Features Supporting This Diagnosis:  Well-circumscribed, encapsulated lesion (Image 1 - low power):  The lesion is clearly demarcated from surrounding tissue and enclosed by a fibrous capsule, which is a hallmark of schwannomas.  It appears as a solitary, round to oval mass without evidence of infiltration.  Biphasic histologic pattern (Images 2 and 3 - medium to high power):  The lesion shows alternating Antoni A and Antoni B areas:  Antoni A: Densely cellular regions with spindle-shaped cells arranged in short fascicles or palisades. These may form Verocay bodies (though not distinctly visible here, the pattern is consistent).  Antoni B: Looser, hypocellular areas with a myxoid stroma.  Nuclear palisading (Image 3 - high power):  There are zones of nuclear alignment suggestive of palisading, typical of Antoni A areas.  Spindle-shaped Schwann cells with elongated nuclei can be appreciated.  Lack of atypia, mitotic activity, or necrosis:  No cytologic atypia or abnormal mitotic figures are seen, supporting a benign peripheral nerve sheath tumor.  Conclusion:  The histologic features align most consistently with a Schwannoma, a benign neural tumor arising from Schwann cells, commonly seen in the head and neck region, including oral and maxillofacial sites. | Histopathological diagnosis  → Soft-tissue myxoma (superficial / circumscribed myxoma of the oral mucosa)  Key microscopic features visible in the provided H-&-E sections  Field Observation Significance for the diagnosis  Low-power (whole nodule) • Sharply demarcated, almost spherical submucosal mass lying just beneath intact stratified squamous epithelium.  • Surrounded by a thin rim of compressed fibrous tissue that mimics a capsule. Myxomas are usually well-circumscribed but not truly encapsulated; the compressed fibrous rim seen here is typical of a pseudocapsule.  Overall cellularity • Lesion is strikingly hypocellular – only scattered nuclei are present in a vast amount of matrix. Paucicellularity is a hallmark of myxoma and helps exclude more cellular spindle-cell/myofibroblastic proliferations (e.g., nodular fasciitis, schwannoma Antoni A areas).  Stroma / matrix • Abundant loose, pale eosinophilic to slightly basophilic (“myxoid”) ground substance that separates the cells.  • Delicate wispy collagen/reticulin fibres coursing through the matrix. Matrix rich in hyaluronic-acid mucopolysaccharide is the defining feature of a myxoma.  Tumour cells • Sparse spindle-to-stellate fibroblastic / myofibroblastic cells with long, thin cytoplasmic processes (“hair-like” extensions).  • Nuclei are small, oval to slightly elongated, bland, without pleomorphism or mitoses. The bland, stellate fibroblastic cells embedded in myxoid matrix typify myxoma and distinguish it from neural tumours (which show wavy S-100-positive nuclei, palisading, or axons).  Vasculature & inflammation • Numerous delicate, thin-walled capillaries dispersed uniformly.  • No significant haemorrhage, perivascular hyalinisation or inflammatory infiltrate. Sparse capillaries are expected in myxoma; absence of thick-walled vessels, pericytic cuffing, or marked inflammation helps exclude vascular tumours and reactive myofibroblastic lesions.  Atypia / mitoses / necrosis • None identified in the high-power fields provided. Benign cytology supports a diagnosis of myxoma rather than a low-grade myxoid sarcoma.  Why the other common look-alikes are less likely  Schwannoma / neurofibroma – would show either antoni-A palisading (schwannoma) or wavy, S-shaped nuclei and no true capsule (neurofibroma).  Nodular fasciitis (myxoid phase) – is more cellular, with plump “tissue-culture” fibroblasts, mitotic figures and extravasated red cells.  Myofibroma / myofibromatosis – displays zonation with cellular spindle areas and hemangiopericytoma-like vessels, not seen here.  Taken together, the constellation of a well-circumscribed, extremely hypocellular nodule composed of scattered bland stellate cells in an abundant myxoid matrix with delicate capillaries is classic for soft-tissue myxoma. |
| 6.28 | Diagnosis: Mucosal neuroma (somewhat plexiform pattern) in a patient with MEN-2B; Key Microscopic Features: Rope-like proliferation of nerve fibers of varying diameters within the lamina propria; nerve fibers exhibiting thickened perineurial sheaths; and identifiable neurites and axons. | Based on the hematoxylin-and-eosin (H&E) stained images provided, examined at low, medium, and high magnifications, the most likely histopathological diagnosis is:  Schwannoma (Neurilemmoma)  Key Microscopic Features Supporting This Diagnosis:  Encapsulation and Well-Circumscription (Low Magnification):  The tumor is well-demarcated from the surrounding tissue, often encapsulated.  Lobulated architecture with a biphasic pattern of tissue density.  Antoni A and Antoni B Areas (Medium Magnification):  Antoni A areas are more cellular, organized, and consist of spindle cells arranged in fascicles with nuclear palisading (Verocay bodies).  Antoni B areas are less cellular, more myxoid, with loosely arranged cells and a vacuolated background.  Verocay Bodies (High Magnification):  Formed by two rows of palisaded nuclei separated by an anuclear zone; this is highly characteristic of Schwannoma.  Spindle cells with wavy, elongated nuclei and eosinophilic cytoplasm.  Lack of Atypia or Significant Mitotic Activity:  Cells appear benign with no marked pleomorphism or mitoses, supporting a benign nerve sheath tumor.  Differentiation from Other Entities:  Neurofibroma would show a more diffuse pattern with no encapsulation or Verocay bodies.  Leiomyoma or Myofibroma would show smooth muscle differentiation but lack Verocay bodies and biphasic Antoni patterns.  Lipoma would consist of mature adipocytes.  Hemangioma would have vascular spaces, which are not seen here. | Histopathological diagnosis  Angioleiomyoma (vascular leiomyoma) of the oral submucosa.  Key microscopic features that support this diagnosis  Magnification Findings Diagnostic significance  Low-power (first image) • Sharply circumscribed, lobulated mass within the lamina propria/submucosa, covered by intact stratified squamous epithelium.  • Multiple rounded to oval nodules that appear to emanate from or envelop vascular lumina. A well-demarcated, subepithelial spindle-cell tumour that is closely associated with blood vessels is characteristic of angioleiomyoma.  Intermediate (second image) • Numerous vascular channels—some slit-like, others rounded—lined by flattened endothelium.  • Smooth-muscle bundles arranged concentrically around and between these vessels, forming whorls and sweeping fascicles. Concentric smooth-muscle proliferation around vessels is the defining histology of an angioleiomyoma (distinguishes it from a solid leiomyoma or myofibroma).  High-power (third image) • Spindle cells with abundant, eosinophilic cytoplasm and blunt-ended “cigar-shaped” nuclei, arranged in interlacing fascicles.  • Uniform nuclear chromatin, inconspicuous nucleoli, scarce mitoses, and absence of necrosis or pleomorphism. Cytomorphology confirms a benign smooth-muscle lineage and rules out malignancy (leiomyosarcoma) or neural/myofibroblastic tumours, which lack the classic blunt nuclei and abundant eosinophilic cytoplasm.  Synthesis  The constellation of (1) a circumscribed mucosal/submucosal mass, (2) prominent vascular spaces intimately wrapped by smooth-muscle fascicles, and (3) bland smooth-muscle cytology with blunt nuclei is diagnostic of angioleiomyoma in the oral and maxillofacial region. |
| 6.29 | Diagnosis: Schwannoma; Key Microscopic Features: An encapsulated neoplasm with hypercellular Antoni A areas containing Verocay bodies and hypocellular Antoni B regions, along with elongated spindle cells showing only subtle nuclear palisading. | Based on the provided hematoxylin and eosin (H&E) stained histological images of the same lesion at different magnifications, the single most likely histopathological diagnosis is:  Schwannoma (Neurilemmoma)  Key Microscopic Features Supporting the Diagnosis:  Encapsulation (Low-power image):  The tumor appears well-circumscribed and encapsulated, a hallmark feature of schwannomas.  Antoni A and Antoni B Areas:  The lesion shows alternating hypercellular (Antoni A) and hypocellular (Antoni B) areas.  Antoni A regions: Densely packed spindle cells arranged in fascicles with nuclear palisading.  Antoni B regions: Loosely arranged spindle cells with myxoid stroma and microcystic changes.  Verocay Bodies (Seen in higher magnification):  These are palisading arrangements of nuclei around eosinophilic, acellular zones, typically found in Antoni A areas — classic for schwannomas.  Spindle Cell Morphology:  Uniform, elongated nuclei with tapered ends.  No significant atypia or mitotic activity is apparent, supporting a benign nature.  Lack of Infiltration:  The tumor is non-infiltrative, growing in a pushing manner, also supporting a benign peripheral nerve sheath tumor.  These features collectively support the diagnosis of schwannoma, which is a benign nerve sheath tumor often included in the neural tumors subgroup of oral and maxillofacial pathology. | Most likely diagnosis (single best fit)  Encapsulated schwannoma (a.k.a. neurilemmoma – cellular variant)  Key microscopic features in the three H-E fields that support this diagnosis  Field Feature seen Diagnostic significance  Low-power overview • Sharply circumscribed, encapsulated nodule with a thin fibrous capsule that cleanly separates the lesion from adjacent tissue.  • Subtle variation in cellularity producing lighter (hypocellular) and darker (hypercellular) zones. Encapsulation and biphasic architecture are characteristic of a benign peripheral-nerve-sheath tumour; neurofibroma lacks a true capsule.  Intermediate magnification • Thick collagenous capsule / perineurium visible as an eosinophilic band.  • Thick-walled, hyalinised blood vessel within the tumour.  • Adjacent areas of compact spindle cells abutting looser, more myxoid zones. Schwannomas commonly show a hyalinised capsule and hyalinised vessels; Antoni A (compact) and Antoni B (looser) regions create the biphasic pattern.  High magnification • Densely packed fascicles and whorls of uniform spindle cells with elongated, tapered (“comma-shaped”) nuclei and indistinct cytoplasm (Antoni A pattern).  • Focal nuclear palisading/Verocay-body-like arrangements and swirling storiform areas.  • Minimal pleomorphism, rare mitoses, no necrosis. The cytologic appearance, palisading, and absence of atypia favour schwannoma; the cellularity and whorled growth conform to the cellular schwannoma variant.  Together, these findings – a well-encapsulated lesion composed of uniformly bland Schwann-type spindle cells arranged in compact fascicles with scattered hyalinised vessels and biphasic architecture – are classic for an encapsulated (cellular) schwannoma of the oral and maxillofacial region. |
| 6.30 | Diagnosis: Ancient schwannoma; Key Microscopic Features: An encapsulated tumor displaying marked stromal edema and pseudocystic degeneration, with spindle cell proliferation forming pseudocysts, areas of perivascular hyalinization, chronic inflammatory infiltrates, predominantly Antoni B–type tissue, and palisaded nuclei arranged in an unusual rosette-like configuration. | Based on the provided hematoxylin and eosin (H&E) stained histopathological images of the same lesion from the vascular, neural, adipocytic, muscle, and myofibroblastic tumors category in oral and maxillofacial pathology, the single most likely histopathological diagnosis is:  Nodular Fasciitis  Key Microscopic Features Justifying the Diagnosis:  Overall Architecture (Low Magnification)  The lesion is well-circumscribed yet unencapsulated, showing a nodular growth pattern.  There is a tissue culture-like appearance with loosely arranged, plump spindle to stellate cells.  Areas of myxoid degeneration intermixed with denser fibrous zones are seen.  Cellular Composition (Intermediate Magnification)  The spindle cells are arranged in short fascicles with a feathery or storiform pattern.  These cells exhibit mild nuclear atypia but no significant pleomorphism or increased mitotic activity.  There are extravasated red blood cells and scattered lymphocytes, indicating a reactive process.  Vascular proliferation is present, but the vessels are benign-appearing and thin-walled.  Focal Myxoid Change and Inflammatory Component (High Magnification)  A notable presence of myxoid stroma around the spindle cells, with infiltrating inflammatory cells, especially lymphocytes.  Small areas of microhemorrhage and occasional multinucleated giant cells may be visible.  Summary:  Nodular fasciitis is a benign, self-limiting myofibroblastic proliferation often mistaken for a sarcoma due to its rapid growth and cellularity. The histological triad of plump myofibroblastic cells, myxoid stroma, and scattered inflammatory cells with extravasated RBCs is characteristic and matches the features observed in the images you provided. | Most likely histopathological diagnosis  → Angioleiomyoma (vascular leiomyoma) – solid/capillary type with marked hyalinisation‐calcification  Key microscopic features that point to this diagnosis  Field What is seen in the photographs Diagnostic significance  Low-power overview • A well-circumscribed, encapsulated nodule in the submucosa.  • Numerous irregular, pale-eosinophilic plates and shards scattered through the tumour, some showing central cracks or clefts (long-standing hyalinisation, dystrophic calcification/early ossification). Oral angioleiomyomata are usually discrete masses and often develop prominent degenerative change (hyaline fibrosis, calcification).  Intermediate magnification • Many oval to round vascular lumina; some contain erythrocytes, others are empty but have thick, brightly eosinophilic walls.  • Interlacing and radially oriented bundles of spindle cells that converge on or swirl around these vascular spaces. Thick-walled vessels and a star-burst or whorled array of smooth-muscle bundles around them is classic for the solid (capillary) variant of angioleiomyoma.  High magnification • Spindle cells possess abundant eosinophilic cytoplasm and blunt-ended, “cigar-shaped” nuclei—features of mature smooth-muscle cells.  • Concentric fascicles of these cells merge with homogeneous, glassy collagen at the vessel wall (long-standing hyalinisation).  • Occasional lumina with entrapped red blood cells. The cytomorphology confirms smooth-muscle differentiation, and its intimate relationship with vascular channels clinches the diagnosis of a vascular (angio-) leiomyoma.  Why alternative entities are less likely  Myofibroma/nodular fasciitis – lack the thick muscular vessel walls and cigar-shaped nuclei; usually show a more myxoid stroma and peripheral “zonal” pattern rather than a concentric star-burst of smooth muscle.  Schwannoma/neurofibroma – neural tumours display wavy or palisaded nuclei and S-100 positivity but do not form muscular vessel walls.  Calcifying fibrous tumour – heavily collagenised with psammomatous calcifications but is hypocellular, lacks smooth-muscle bundles and vascular architecture seen here.  Bottom line:  The combination of (1) circumscribed submucosal mass, (2) numerous thick-walled blood vessels, (3) interlacing and radially oriented bundles of smooth-muscle cells with blunt nuclei, and (4) prominent hyalinisation/calcification is diagnostic of an angioleiomyoma (vascular leiomyoma) in the oral and maxillofacial region. |
| 6.30 | Diagnosis: Plexiform schwannoma; Key Microscopic Features: A plexiform, multilobulated proliferation of Antoni A–type tissue containing numerous classic Verocay bodies. | Histopathological Diagnosis:  Neurofibroma  Key Microscopic Features Supporting the Diagnosis:  Low Magnification (First Image):  Well-circumscribed, non-encapsulated lesion in the subepithelial connective tissue:  This is a classic presentation of neurofibroma, which often appears as a relatively well-defined but unencapsulated mass.  Multiple lobules or nodular growth pattern:  The lesion is divided into lobular zones separated by thin fibrous septa, which is frequently seen in neurofibromas.  High Magnification (Second Image):  Spindle-shaped cells with wavy nuclei:  Characteristic of Schwann cell differentiation, these spindle cells have elongated, serpentine ("comma-shaped") nuclei.  Myxoid stroma with scattered collagen fibers:  The pale, loose, myxoid background with collagen bundles is typical of neurofibromas.  Low cellular atypia and mitotic activity:  The absence of nuclear pleomorphism and mitoses supports the benign nature of the lesion.  Presence of mast cells (not easily seen in these images but often present in neurofibromas under higher magnification or special stains).  Differential Diagnosis Considerations:  Schwannoma: Would show Antoni A and B areas and Verocay bodies—absent here.  Traumatic neuroma: Typically more disorganized and associated with a history of trauma.  Fibroma or myofibroma: These usually lack the wavy spindle cells and myxoid stroma.  Leiomyoma: Smooth muscle origin, with cigar-shaped nuclei and eosinophilic cytoplasm, unlike the pale spindle cells here. | Histopathological diagnosis  Solitary myofibroma (myofibroblastic tumour) of the oral mucosa / submucosa.  Key microscopic features that support this diagnosis  Low-power findings High-power findings  • Lobulated, well-circumscribed mass immediately beneath an intact stratified squamous epithelium. Two large nodules are separated by thin fibrous septa, creating a multinodular (sometimes called “plexiform”) appearance. • Spindle-shaped myofibroblasts with tapered, blunt-ended nuclei and scant pale eosinophilic cytoplasm.  • Biphasic (zoning) pattern – each nodule shows a darker, more cellular centre and a paler peripheral rim, a classic feature of myofibroma. • Cells are arranged in interlacing fascicles and whorls, producing a swirling or “storiform-like” pattern typical of this tumour.  • No infiltration of surrounding fat or muscle and no overlying epithelial dysplasia, favouring a benign process. • Numerous thin-walled, branching (“hemangiopericytoma-like”) vascular channels with concentric perivascular cuffing of tumour cells – a hallmark of myofibroma / myopericytic lesions.  • Minimal cytological atypia and very low mitotic activity; necrosis is absent, confirming a benign spindle-cell lesion.  The constellation of a lobulated, biphasic, perivascular whorling spindle-cell proliferation with minimal atypia in the oral submucosa is diagnostic of solitary myofibroma and distinguishes it from other benign spindle-cell tumours such as neurofibroma, schwannoma, leiomyoma, or solitary fibrous tumour. |
| 6.30 | Diagnosis: Cellular schwannoma; Key Microscopic Features: A dense proliferation of spindle cells with infiltrative borders, arranged in intersecting fascicles, exhibiting indistinct cell borders, pale cytoplasm, and benign tapered nuclei. | Histopathological Diagnosis:  Neurofibroma  Key Microscopic Features Supporting the Diagnosis:  Low-Power View (Image 1):  Poorly circumscribed lesion expanding the dermis and subcutaneous tissue.  The lesion merges imperceptibly with the surrounding tissue — a hallmark of non-encapsulated neurofibroma.  The overlying epithelium is intact and shows no dysplasia or ulceration.  Intermediate-Power View (Image 2):  Proliferation of spindle-shaped cells with wavy, elongated nuclei arranged in a loose, myxoid stroma.  Lack of significant nuclear pleomorphism or mitotic figures.  Scattered mast cells may be seen (though less prominent here, common in neurofibromas).  High-Power View (Image 3):  Spindle cells with elongated, serpentine (comma-shaped) nuclei, characteristic of Schwann cell origin.  The collagen fibers are interspersed in a delicate, wispy pattern.  No atypia, necrosis, or high mitotic activity—features that help rule out malignant peripheral nerve sheath tumor (MPNST).  Summary Justification:  The lesion's architecture and cytologic features are characteristic of a neurofibroma, a benign peripheral nerve sheath tumor. The non-encapsulated, diffuse growth pattern, spindle-shaped cells, and wavy nuclei in a myxoid background are diagnostic. This rules out other spindle cell tumors like schwannoma (which is typically encapsulated and shows Antoni A/B areas and Verocay bodies) and smooth muscle tumors (which have cigar-shaped nuclei and are more fascicular). | Most likely histopathological diagnosis  Solitary myofibroma (myofibroma of the oral mucosa) – a benign myofibroblastic tumour that commonly arises in the submucosa of the oral cavity.  Key microscopic features supporting this diagnosis  Field Observation Diagnostic significance  Low-power architecture • A single, well-circumscribed but unencapsulated nodule in the submucosa  • Overlying stratified squamous epithelium intact  • Obvious zonal (biphasic) pattern – pale peripheral areas and more basophilic, cellular central foci The circumscribed, lobular growth and biphasic “light–dark” zoning are classic for myofibroma.  Intermediate power • Intersecting fascicles and whorled/storiform arrangements of spindle cells  • Cellular nodules merge with less cellular, collagen-rich stroma The swirling fascicles of uniform spindle cells with alternating cellularity are characteristic and help separate myofibroma from smooth-muscle or neural tumours.  Vascular pattern • Numerous thin-walled, branching (“hemangiopericytoma-like”) capillaries coursing through the cellular zones (best appreciated at medium power) The prominent, staghorn-type vasculature is a hallmark of myofibroma and explains its occasional clinical pulsatility.  High-power cytology • Uniform spindle to plump myofibroblasts with tapered/blunt-ended nuclei and pale-eosinophilic cytoplasm  • Inconspicuous nucleoli, minimal pleomorphism, rare mitoses, no necrosis Cytologic blandness with low mitotic activity confirms a benign myofibroblastic lesion rather than sarcoma.  Stroma • Delicate collagen bundles and focal hyalinisation around vessels Collagenous matrix is typical, contrasting with the myxoid background of nodular fasciitis.  Collectively, the zonal architecture, whorled fascicles of bland spindle myofibroblasts, hemangiopericytoma-like vessels, and lack of significant atypia or mitoses point most convincingly to a solitary myofibroma. |
| 6.31 | Diagnosis: Solitary circumscribed neuroma with a unilobular pattern; Key Microscopic Features: A well-defined aggregation of spindle-shaped cells forming tissue clefts, with neuritic processes and spindle cells organized into short bundles, and the spindle cells display slender, elongated nuclei. | Histopathological Diagnosis:  Neurofibroma  Key Microscopic Features Justifying the Diagnosis:  1. Low-power view (First Image):  Well-circumscribed, non-encapsulated lesion located in the dermis/submucosa, pushing up the overlying epithelium.  The lesion blends with surrounding connective tissue without a true capsule—this is a hallmark of neurofibroma.  No signs of aggressive infiltration or necrosis.  2. Intermediate-power view (Second Image):  Shows a loose fascicular architecture with wavy, spindle-shaped cells that have elongated nuclei.  The stroma is relatively myxoid or collagenous, another typical feature.  Absence of significant cellular pleomorphism or mitotic activity.  3. High-power view (Third Image):  The spindle cells have pale, eosinophilic cytoplasm and elongated, wavy ("comma-shaped") nuclei, characteristic of Schwann cells and fibroblasts seen in neurofibromas.  Mild inflammatory infiltrate and small blood vessels are present, but no atypia, necrosis, or high mitotic rate—all of which support a benign nature.  Lack of Verocay bodies or Antoni A/B areas (which are seen in schwannomas), helping to distinguish this lesion.  Additional Considerations (to confirm diagnosis):  Immunohistochemistry for S100 protein (typically diffusely positive) and neurofilament protein (for axons within the lesion) would confirm the neural origin, consistent with neurofibroma. | Histopathological diagnosis  Vascular leiomyoma (angioleiomyoma) – a benign smooth-muscle tumour in which bundles of mature smooth-muscle cells are intimately associated with thick-walled blood vessels.  Key microscopic features that support the diagnosis  Field Observations Diagnostic implication  Low power (1st image) • A single, well-circumscribed, almost spherical nodule located entirely within the submucosa/dermis.  • A delicate fibrous capsule separates the lesion from the surrounding connective tissue.  • Numerous serpiginous, slit-like lumina course through the mass. A sharply circumscribed, encapsulated mass is a hallmark of leiomyoma; the conspicuous intralesional vascular channels point to the angio subtype.  Intermediate power (2nd image) • Interlacing / swirling fascicles of uniform spindle cells forming a whorled architecture.  • Fascicles are frequently oriented circumferentially around thick vascular channels. The orderly fascicular pattern is typical of mature smooth muscle; the perivascular arrangement is characteristic of vascular leiomyoma.  High power (3rd image) • Spindle cells with abundant eosinophilic cytoplasm and blunt (“cigar-shaped”) central nuclei; chromatin is fine, nucleoli inconspicuous, mitoses rare or absent.  • Thick-walled vessels lined by endothelium with concentric layers of smooth-muscle cells in their wall.  • Scattered lymphocytes/plasma cells but no significant atypia or necrosis. Cytomorphology confirms smooth-muscle differentiation; the conspicuous muscularized vessel walls secure the diagnosis of angio-leiomyoma, and the bland cytology rules out leiomyosarcoma or other spindle-cell sarcomas.  Taken together, the combination of a well-defined submucosal nodule composed of bland, eosinophilic spindle cells arranged in concentric fascicles around thick-walled vascular channels is diagnostic of vascular leiomyoma (angioleiomyoma), a benign smooth-muscle tumour frequently encountered in the oral and maxillofacial region.  2/2 |
| 6.31 | Diagnosis: Solitary circumscribed neuroma with a multilobular pattern; Key Microscopic Features: Numerous irregularly winding tumor lobules appear, alongside neuritic processes and spindle cells exhibiting undulating nuclei, mild nuclear palisading, and the presence of tissue clefts. | Based on the hematoxylin-and-eosin (H&E) stained histologic sections you’ve provided — one at low magnification and one at higher magnification — the lesion belongs to the neural category of oral and maxillofacial tumors.  Single Most Likely Histopathological Diagnosis:  Palisaded Encapsulated Neuroma (PEN)  Key Microscopic Features Supporting This Diagnosis:  Low Power (First Image):  Well-circumscribed, multinodular dermal lesion under intact squamous epithelium.  Appears partially or fully encapsulated by a thin fibrous capsule.  Multiple lobules of spindle-cell proliferation within the lesion.  High Power (Second Image):  Spindle-shaped cells with wavy nuclei arranged in interlacing fascicles or short bundles.  Lacks significant pleomorphism or mitotic activity.  Cells are S-100 protein positive (known from immunohistochemistry in practice, typical of Schwann cells).  There is no Verocay body formation, helping distinguish this from schwannoma.  Absence of a myxoid matrix or Antoni A/B areas, which would be seen in schwannoma or neurofibroma.  Minimal or no inflammatory infiltrate, and no necrosis.  Additional Diagnostic Considerations (Excluded by Features):  Neurofibroma: Less circumscribed, more collagenous stroma, lacks capsule.  Schwannoma: Shows Antoni A and B areas, Verocay bodies — not seen here.  Traumatic neuroma: Disorganized proliferation of nerve fibers and fibrosis — more chaotic appearance.  Leiomyoma / Myofibroma: Spindle cells but with eosinophilic cytoplasm and cigar-shaped nuclei, different arrangement. | Histopathological diagnosis  → Plexiform neurofibroma  Key microscopic features that support this diagnosis  Field Findings in these H&E sections Diagnostic significance  Overall architecture (low-power) • Multinodular/plexiform “bag-of-worms” configuration of several discrete, round-to-ovoid nodules expanding the submucosa beneath an intact stratified squamous epithelium Plexiform growth pattern is characteristic of neurofibroma arising from multiple nerve fascicles rather than a single encapsulated mass  Stroma • Each nodule is unencapsulated and separated by loose collagenous tissue  • Background shows delicate myxoid matrix with scattered small vessels Lack of a true capsule helps distinguish neurofibroma from schwannoma; myxoid matrix is typical of neurofibroma  Cell type and arrangement (high-power) • Interlacing bundles and whorls of bland spindle cells with elongated, wavy (“comma-shaped”) nuclei and tapered ends  • Cells have indistinct borders and scant cytoplasm Schwann-cell/nerve-sheath phenotype; wavy nuclei are a classic clue  Additional features • Occasional mast cells and thin collagen fibrils within nodules (noted as small, dark round nuclei between spindle cells)  • Absent Verocay bodies and Antoni A/B zoning (helps exclude schwannoma)  • No cytologic atypia or brisk mitoses Consistent with a benign neurofibroma rather than schwannoma or a myogenic/myofibroblastic lesion  The combination of a plexiform (multifascicular) architecture and the cytology of benign peripheral-nerve–sheath spindle cells without encapsulation points specifically to plexiform neurofibroma—the prototype lesion in the neural category of oral & maxillofacial soft-tissue tumours. |
| 6.32 | Diagnosis: Perineurioma; Key Microscopic Features: A widespread growth of spindle cells arranged in fascicles, with the cells bearing benign oval nuclei encircling organoid configurations, and hallmark pseudo-onion bulb structures. | Histopathological Diagnosis:  Myofibroma  Key Microscopic Features Supporting the Diagnosis:  Biphasic Growth Pattern:  The lesion exhibits a characteristic biphasic appearance. One area displays a more hypercellular region with spindle-shaped cells arranged in interlacing fascicles, while another region shows less cellular, more collagenized zones. This mixture is typical for myofibromas.  Whorled/Storiform Pattern:  Seen clearly in the medium and high magnification images, the spindle cells are arranged in whorled or storiform patterns, which is a hallmark feature of myofibroma.  Plump Spindle Cells with Pale Eosinophilic Cytoplasm:  The neoplastic cells are uniform, with bland, elongated nuclei and a moderate amount of cytoplasm, lacking atypia or significant pleomorphism.  Hemangiopericytoma-like Vascular Pattern:  While not very prominent in these particular fields, myofibromas often feature a rich vascular network, sometimes resembling the hemangiopericytoma-like pattern (staghorn vessels), especially at the periphery.  Lack of Significant Atypia or Mitoses:  The nuclei appear uniform, and no significant mitotic activity or atypical features are noted—this supports the benign nature of the lesion.  Correlative Notes:  Myofibroma is a benign myofibroblastic tumor commonly found in the head and neck region, especially in children and young adults.  These tumors are typically well-circumscribed but not encapsulated, and can show infiltrative growth in soft tissues. | Histopathological diagnosis  Solitary myofibroma (of the oral soft tissues) – a benign perivascular myoid / myofibroblastic tumour that is common in the head-and-neck region.  Key microscopic features visible in the submitted H-E sections  Field (image) What you can see Why it points to myofibroma  Low-power overview (first image) A well–circumscribed but unencapsulated nodule composed of spindle cells. There is a subtle “zoning” pattern – the central portion is more intensely basophilic (cell-rich) while the peripheral rim is paler and more collagenised. The biphasic “central cellular + peripheral myoid” architecture is characteristic of myofibroma.  Intermediate & high power (second & third images) Onion-skin / whorled fascicles of uniform spindle cells that wrap concentrically around small thin-walled vascular lumina or hyalinised collagen cores. The cells have tapered, blunt-ended (“cigar-shaped”) nuclei, fine chromatin and scant, pale eosinophilic cytoplasm. Concentric perivascular whorls are a hallmark of the pericytic/myofibroblastic family; in the oral cavity this pattern is most typical of myofibroma. The nuclei are bland, supporting a benign lesion.  Numerous delicate, branching (“hemangiopericytoma-like”) vessels and occasional extravasated red blood cells. A rich vascular component with hemangiopericytoma-like vessels forms the cell-rich central zone of myofibroma. Extravasated RBCs are frequently present.  Absence of significant pleomorphism, necrosis or brisk mitotic activity. Confirms the lesion is benign, helping exclude sarcomas with similar spindle-cell morphology.  Putting the features together  The combination of:  Biphasic zoning (cell-rich vascular centre and myoid/ collagenised periphery)  Perivascular concentric whorls of bland spindle cells (“onion-skin” pattern)  Delicate branching vessels with occasional RBC extravasation  Lack of significant cytologic atypia or mitoses  is diagnostic of solitary myofibroma in the spectrum of vascular-myoid / myofibroblastic tumours of the oral and maxillofacial region. |
| 6.34 | Diagnosis: Granular cell tumor; Key Microscopic Features: An unencapsulated neoplasm with a slightly nodular appearance infiltrating the underlying muscle, granular cells adjacent to epithelium demonstrating papillary atrophy and localized pseudoepitheliomatous hyperplasia, and cells featuring granular cytoplasm with small intracytoplasmic inclusions and innocuous nuclei. | Based on the provided hematoxylin and eosin (H&E)-stained images of a lesion from the oral and maxillofacial region—captured at low, intermediate, and high magnifications—and considering the diagnostic grouping of vascular, neural, adipocytic, muscle, and myofibroblastic tumors, the most likely histopathological diagnosis is:  Granular Cell Tumor (GCT)  Key Microscopic Features Supporting This Diagnosis:  1. Low Power View (First Image):  Well-circumscribed but non-encapsulated lesion within the submucosa.  Overlying stratified squamous epithelium with pseudoepitheliomatous hyperplasia (PEH)—a common feature in granular cell tumors.  No significant infiltration into adjacent structures (muscle, fat, or deep tissues), but the lesion integrates smoothly with surrounding stroma.  2. Intermediate Power View (Second Image):  The lesion is composed of uniform polygonal cells arranged in nests or sheets.  These cells have abundant granular eosinophilic cytoplasm.  The nuclei are small, central, and round to oval, often with inconspicuous nucleoli.  A characteristic lack of significant atypia or mitotic activity.  3. High Power View (Third Image):  The cytoplasm of tumor cells appears finely granular and eosinophilic, reflecting lysosomal accumulation.  Nuclei are bland with low nuclear-cytoplasmic ratio.  No evidence of lipoblasts, striations, neural fascicles, or vascular spaces that might suggest alternative diagnoses like liposarcoma, leiomyoma, schwannoma, or hemangioma.  Additional Supporting Diagnostic Considerations:  Granular cell tumors are thought to be of Schwann cell origin, typically positive for S-100 protein on immunohistochemistry.  PEH may mimic squamous cell carcinoma, but underlying bland granular cells help differentiate the two. | Histopathological diagnosis  Granular cell tumour (Abrikossoff tumour) of the oral mucosa.  Key microscopic features that support the diagnosis  Low → High power feature What is seen in the provided sections Why it points to a granular-cell tumour  Relationship to surface epithelium Intimate subepithelial location with pronounced pseudo-epitheliomatous hyperplasia of the over-lying stratified squamous epithelium (image 2) Granular-cell tumours commonly elicit marked reactive hyperplasia that can mimic SCC in biopsy material  Growth pattern / circumscription Ill-defined, infiltrative mass that permeates the lamina propria and extends between skeletal-muscle bundles (image 1) GCTs are usually not encapsulated and blend with adjacent soft tissues  Tumour cells – size & outline Sheets and nests of large polygonal cells with indistinct cell borders, giving a syncytial appearance (images 2-3) Characteristic for GCT; helps distinguish from spindle-cell lesions in the same category  Cytoplasm Abundant, pale-to-eosinophilic, coarsely granular cytoplasm; occasional vacuolated/foamy appearance (image 3) Coarse eosinophilic granules are diastase-resistant PAS-positive lysosomes – the defining feature of GCT  Nuclei Small, centrally placed, uniform round-to-oval nuclei with inconspicuous nucleoli; mitoses absent (image 3) Benign cytology typical of conventional GCT  Stroma & vessels Delicate collagenous stroma with scant vasculature; no thick-walled vessels or hemangiopericytic pattern (images 1-2) Helps rule out angioleiomyoma, myofibroma and other perivascular myoid tumours  Taken together, the combination of a subepithelial, non-encapsulated mass composed of large cells with granular cytoplasm and pseudo-epitheliomatous hyperplasia of the covering epithelium is diagnostic of a granular-cell tumour. |
| 6.35 | Diagnosis: Granular cell tumor; Key Microscopic Features: Prominent pseudoepitheliomatous hyperplasia, epithelial nests showing atypical cellular features and dyskeratosis, and granular cells containing large eosinophilic cytoplasmic inclusions. | Based on the provided hematoxylin and eosin (H&E) stained images at different magnifications, the lesion can be evaluated as follows:  Histopathological Diagnosis:  Granular Cell Tumor (GCT)  Key Microscopic Features Supporting the Diagnosis:  Overall Architecture (Low Power View):  The first image demonstrates a submucosal, non-encapsulated lesion with infiltration into surrounding connective tissue, typical of granular cell tumors.  There is pseudoepitheliomatous hyperplasia of the overlying epithelium, a common feature in GCTs that can mimic squamous cell carcinoma.  Cellular Morphology (Medium to High Power Views):  The second and third images show large polygonal cells with abundant granular eosinophilic cytoplasm, a hallmark of granular cell tumors.  The granularity is due to the presence of numerous lysosomes, and the cells have small, centrally located, vesicular nuclei.  The cells are arranged in nests or sheets, and infiltrate the surrounding tissues.  There is no significant mitotic activity or nuclear atypia.  Differential Considerations Excluded:  Absence of atypical mitotic figures or pleomorphism helps rule out malignancies.  The granular cell appearance helps differentiate it from neural or adipocytic tumors.  No evidence of vascular proliferation or muscle differentiation.  Conclusion:  These histopathological features—especially the granular cytoplasm, pseudoepitheliomatous hyperplasia, and lack of encapsulation—are diagnostic of a Granular Cell Tumor, a benign lesion commonly found in the oral cavity, particularly on the tongue. | Histopathologic diagnosis – Granular Cell Tumor (Abrikossoff tumor)  Key microscopic features that support the diagnosis  Field Diagnostic findings Why it points to a granular cell tumor  Low-power architecture • Unencapsulated, poorly circumscribed mass expanding the lamina propria/submucosa.  • Surface stratified squamous epithelium shows pronounced pseudo-epitheliomatous hyperplasia (deep, jagged rete ridges that may simulate invasive SCC). PEH of the overlying mucosa is seen in ≈50 % of oral granular cell tumors and can masquerade as carcinoma.  Surface epithelium (2nd image) • Down-growing epithelial nests containing concentric keratin pearls, but no significant cytologic atypia or destructive invasion. Confirms the PEH rather than a true primary epithelial malignancy; a classic “trap” with granular cell tumors.  Tumor cell cytology (3rd image) • Sheets and cords of large polygonal cells with:  – Abundant eosinophilic, finely granular cytoplasm (lysosome-rich).  – Small, uniform, centrally placed nuclei without mitotic activity. The hallmark granular cytoplasm produced by lysosome accumulation is diagnostic of a granular cell tumor.  Stroma & interface • Cells interdigitate between native collagen bundles and skeletal muscle fibers without a capsule. The infiltrative, non-capsulated pattern is typical; muscle permeation is frequent in oral lesions.  Ancillary tests (not pictured but classically positive): S-100, SOX-10, and NKI/C3 immunoreactivity confirming Schwann-cell origin; PAS-D positivity of cytoplasmic granules.  These combined histologic features—pseudo-epitheliomatous hyperplasia of the mucosa overlaying an unencapsulated proliferation of S-100–positive granular cells—are pathognomonic for a granular cell tumor within the neural/Schwannian category of oral soft-tissue tumors. |
| 6.35 | Diagnosis: Plexiform granular cell tumor; Key Microscopic Features: Numerous lobular aggregates of granular cells and tumor tissue enveloping a nerve fiber. | Based on the provided hematoxylin and eosin (H&E) stained histological images at low and high magnification, the most likely histopathological diagnosis is:  Nodular Fasciitis  Key Microscopic Features Supporting the Diagnosis:  Low-Magnification Image:  Lobulated architecture: A well-circumscribed yet non-encapsulated lesion with a lobular growth pattern within the submucosa.  Mild overlying mucosal hyperplasia without evidence of epithelial dysplasia or ulceration.  Spindle cell proliferation: The bulk of the lesion shows a loosely arranged spindle cell proliferation that blends into the adjacent tissue.  High-Magnification Image:  Plump spindle-shaped cells with vesicular nuclei and prominent nucleoli, arranged in short fascicles and a feathery, tissue culture–like pattern.  Myxoid to collagenous stroma with loosely arranged collagen fibers.  Scattered mitoses are present but no atypical mitotic figures, which helps to distinguish this lesion from sarcomas.  Extravasated red blood cells and a rich capillary network are often noted in nodular fasciitis.  Mild chronic inflammatory infiltrate may also be present.  Additional Notes:  Nodular fasciitis is a benign, self-limited myofibroblastic proliferation that often presents rapidly and can clinically mimic a malignancy due to its growth rate and histologic cellularity. In the oral cavity, it typically occurs on the buccal mucosa or tongue. | Histopathological diagnosis  Solitary myofibroma (oral myofibroma / infantile-type myofibromatosis, solitary form)  Key microscopic features supporting this diagnosis  Field Observations Diagnostic value  Low-power architecture • Well-circumscribed, dome-shaped nodule seated in the lamina propria/submucosa, beneath an intact stratified squamous epithelium.  • Lesion shows a pale, lightly collagenised peripheral zone and a slightly darker, more cellular central zone. A sharply outlined submucosal mass is characteristic of a benign myofibroblastic proliferation such as myofibroma. The subtle “biphasic” zoning is typical.  Cellular composition (high power) • Interlacing short fascicles and whorls of bland spindle- to ovoid myoid cells with pale eosinophilic cytoplasm and tapering nuclei.  • Nuclei uniform, inconspicuous nucleoli, rare mitoses, no atypia. Uniform, cytologically bland myofibroblasts are hallmarks of myofibroma and help exclude sarcoma.  Vascular pattern • Numerous thin-walled, branching (“staghorn”) vessels.  • Concentric onion-skin / hemangiopericytoma-like perivascular whorling of tumour cells around many vessels (clearly seen in the higher-magnification image). The distinctive perivascular concentric arrangement is the single most helpful clue to a myofibroma (and separates it from an irritation fibroma, neuroma, etc.).  Stroma • Delicate collagen fibres with focal hyalinisation; scant inflammatory infiltrate; no significant myxoid change or RBC extravasation. The collagenous, only mildly myxoid stroma – together with the vascular pattern – argues against nodular fasciitis and other fibrous lesions.  Growth behaviour • Pushing (rather than infiltrative) interface with adjacent tissue, no destruction of overlying epithelium. Correlates with the benign nature of a solitary myofibroma.  How these features point to myofibroma  Biphasic appearance – peripheral mature spindle cells and a more cellular central zone.  Characteristic vessels – thin-walled, branching vessels with a sleeve of concentrically arranged myoid cells.  Bland myofibroblastic cytology – uniform spindle/ovoid cells, scant mitoses, absence of atypia.  Well-circumscribed submucosal nodule – typical clinical–pathological presentation in the oral cavity.  Taken together, the combination of a circumscribed oral submucosal mass composed of bland myofibroblasts arranged in short fascicles and, most importantly, the hemangiopericytoma-like concentric perivascular pattern is diagnostic of solitary myofibroma. |
| 6.37 | Diagnosis: Angioleiomyoma; Key Microscopic Features: A proliferation of spindle-shaped cells intermingled with dilated, congested blood vessels, and benign spindle smooth muscle cells encircling vascular structures. | Based on the provided hematoxylin and eosin (H&E) stained histological images—captured at different magnifications—and your classification within the vascular, neural, adipocytic, muscle, and myofibroblastic tumors group of oral and maxillofacial pathology, the single most likely histopathological diagnosis is:  Angioleiomyoma (vascular leiomyoma)  Key Microscopic Features Justifying the Diagnosis:  1. Well-Circumscribed Lesion:  The low-power image shows a well-demarcated lesion surrounded by normal tissue, suggesting a benign neoplasm.  2. Interlacing Bundles of Smooth Muscle:  The high-power view highlights interweaving fascicles of spindle cells with elongated, blunt-ended ("cigar-shaped") nuclei—classic of smooth muscle differentiation.  3. Numerous Thick-Walled Blood Vessels:  Prominent numerous vascular channels are observed, often with thick muscular walls, a hallmark of angioleiomyoma.  These vessels are interspersed between the smooth muscle bundles, suggesting intimate incorporation into the tumor.  4. Lack of Atypia or Significant Mitotic Activity:  The spindle cells show bland nuclear features, with no significant atypia or mitotic figures, supporting a benign process.  Additional Supporting Points:  Location: Angioleiomyomas are not common but can occur in the head and neck region, including the oral cavity.  Histologic Variants: This appears to represent the solid type, which is the most common and is composed predominantly of smooth muscle with interspersed blood vessels. | Histopathological diagnosis  Angioleiomyoma (vascular leiomyoma) – venous/solid type  Key microscopic features supporting this diagnosis  Low-power findings High-power findings Diagnostic implication  Sharply circumscribed, lobulated nodule composed of intersecting, pink, fascicular tissue Fascicles are made of spindle cells with eosinophilic, fibrillary cytoplasm and blunt-ended (“cigar-shaped”) nuclei Classic architecture of a benign smooth-muscle tumour  Numerous round-to-oval and slit-like vascular lumina scattered throughout; some are cavernous and congested with erythrocytes Many lumina have a concentric cuff of the same smooth-muscle cells forming a thick muscular wall; others show a less-developed media (venous-type) Defines an angio-leiomyoma – a leiomyoma in which vessels form an integral part of the tumour  Stroma is variably hyalinised/collagenised but lacks necrosis Minimal cytologic atypia, absent/rare mitoses, no necrosis Confirms benign nature and distinguishes it from leiomyosarcoma  Lesion arises in the soft-tissue compartment of the oral–maxillofacial region, where angiomyoma is the commonest smooth-muscle neoplasm Immunohistochemistry (if performed) would show SMA & desmin positivity in tumour cells, CD34 only in endothelial lining Corroborative but not required when H-E features are typical  These features – a well-circumscribed proliferation of benign smooth-muscle bundles intimately associated with thick-walled and variably ectatic blood vessels – are characteristic for angioleiomyoma and differentiate it from ordinary hemangioma (thin-walled, endothelial proliferation), myofibroma (biphasic myoid–cellular pattern with hemangiopericytoma-like vessels), or other spindle-cell lesions in the same diagnostic group. |
| 6.39 | Diagnosis: Leiomyoma; Key Microscopic Features: An unencapsulated growth of spindle-shaped and more robust epithelioid cells, with increased cellularity showing plump, epithelioid morphology, and epithelioid cells possessing abundant eosinophilic, vacuolated cytoplasm and oval nuclei. | Histopathological Diagnosis:  Myofibroma  Key Microscopic Features Justifying the Diagnosis:  The series of hematoxylin and eosin (H&E) stained sections at low, intermediate, and high magnification reveal the classic features of a myofibroma, a benign myofibroblastic tumor commonly seen in the oral and maxillofacial region.  Low Power (First Image):  Well-circumscribed dermal/submucosal lesion: The tumor is sharply demarcated from surrounding tissue and located beneath an intact epithelium.  Nodular architecture: The lesion shows a biphasic growth pattern with alternating light and dark areas, typical of myofibroma.  Intermediate Power (Second Image):  Spindle cell proliferation: The lesion consists of interlacing fascicles of spindle-shaped cells with elongated nuclei.  Staghorn or hemangiopericytoma-like vascular pattern: Numerous thin-walled, branching blood vessels are evident, a hallmark of myofibroma.  High Power (Third Image):  Cellular heterogeneity: There is a mixture of:  Myoid-appearing spindle cells with eosinophilic cytoplasm.  Small, round-to-ovoid cells with hyperchromatic nuclei in the more cellular zones.  Minimal atypia and low mitotic activity: Supporting benign behavior.  Fibromyxoid stroma: With a relatively collagenous background.  Summary:  These images are diagnostic of myofibroma, supported by:  Biphasic pattern (light/dark zones),  Spindle and round cell mix,  Characteristic vasculature,  Lack of significant atypia or high mitotic activity. | Histopathological diagnosis  Cutaneous (pilo-)leiomyoma – solid type benign smooth-muscle tumour  Key microscopic features that support this diagnosis  Field Observation Diagnostic implication  Low-power (whole lesion) • Sharply circumscribed nodule occupying the full thickness of the dermis/submucosa.  • Overlying epithelium is intact and uninvolved.  • Tumour shows a broad, whorled/fascicular architecture. A well-defined, non-infiltrative mass with a sweeping fascicular pattern is typical of a leiomyoma arising from arrector-pili smooth muscle.  Intermediate-power • Interlacing bundles of spindle cells sweep at right-angles giving a “whorled” appearance.  • Numerous narrow vascular lumina lie between fascicles but lack thick muscular coats (distinguishes it from angioleiomyoma). The organised interlacing bundles are characteristic for solid/piloleiomyoma; absence of thick-walled vessels helps exclude angioleiomyoma.  High-power • Cells have abundant, eosinophilic fibrillary cytoplasm and blunt, cigar-shaped (truncated) nuclei, many with a juxta-nuclear clear vacuole (“perinuclear halo”).  • Nuclei are uniform, with inconspicuous nucleoli, minimal pleomorphism, and virtually no mitotic figures.  • Stroma is scant collagen without myxoid change. Classic cytological hallmarks of benign smooth-muscle differentiation confirm leiomyoma and exclude neural or fibrohistiocytic lesions.  Why other entities were ruled out  Myofibroma / solitary fibrous tumour – would show hemangiopericytoma-like branching (“staghorn”) vessels and a biphasic pattern, which are absent here.  Schwannoma / neurofibroma – would display wavy or palisaded nuclei and S-shaped bundles rather than blunt “cigar” nuclei.  Angioleiomyoma – requires thick, muscularised vessel walls that are not present in these sections.  Together, the circumscribed dermal nodule composed of intersecting fascicles of uniform smooth-muscle cells with blunt nuclei and no atypia is diagnostic of a solid cutaneous (piloleiomyoma). |
| 6.40 | Diagnosis: Rhabdomyoma; Key Microscopic Features: Sheets of polygonal cells containing vacuoles, with cells displaying abundant eosinophilic cytoplasm and cytoplasmic vacuolations, and featuring small nuclei with inconspicuous nucleoli. | Histopathological Diagnosis:  Hibernoma  Key Microscopic Features Supporting the Diagnosis:  Multivacuolated Adipocytes Resembling Brown Fat:  The hallmark of hibernoma is the presence of large polygonal cells with abundant, multivacuolated cytoplasm and small, centrally located nuclei.  These vacuolated cells do not compress the nucleus eccentrically like white fat cells but rather contain numerous finely divided lipid droplets reminiscent of brown fat.  Lobular Growth Pattern:  The lesion appears to have a lobulated architecture, separated by fibrous septa, a common architectural feature in hibernomas.  Highly Vascularized Stroma:  Abundant capillary-sized blood vessels scattered throughout the lesion, often seen in hibernomas due to the metabolic activity associated with brown fat.  Lack of Atypia or Mitotic Activity:  There is no cytological atypia, necrosis, or significant mitotic activity — consistent with the benign nature of this lesion.  Additional Notes:  Hibernomas are rare benign tumors composed of brown fat-like cells and typically arise in areas where brown fat persists, but can occur in the head and neck, including the oral and maxillofacial region.  Differentiation from other lipomatous tumors (like lipoblastoma or atypical lipomatous tumor) is critical; the lack of lipoblasts, atypia, and mitoses helps rule out malignancy. | Histopathological diagnosis (single best):  Hibernoma (benign brown-fat tumour)  Key microscopic features supporting this diagnosis  Feature How it appears in the provided sections Why it points to hibernoma  Lobulated, well-circumscribed mass Low-power overview shows a sharply demarcated, vaguely lobular lesion separated by delicate fibrous septa from surrounding tissue. Benign adipocytic tumours are usually circumscribed; hibernoma typically has a lobulated architecture.  Predominance of multivacuolated adipocytes (“hibernoma cells”) At medium and high power, the bulk of the tumour is composed of polygonal cells whose cytoplasm contains numerous variably sized, round clear vacuoles giving a bubbly appearance. Brown-fat cells characteristically contain many small lipid droplets rather than a single large one; this multivacuolated pattern is the hallmark of hibernoma.  Central, small, round nuclei without atypia Nuclei sit centrally in the cytoplasm, are uniform, lack hyperchromasia or mitoses. Benign nature; distinguishes the cells from lipoblasts of liposarcoma, which show nuclear indentation and atypia.  Granular, eosinophilic cytoplasm between vacuoles Between the droplets, the cytoplasm stains pink-granular on H&E. Reflects the high mitochondrial content of brown fat.  Rich, delicate capillary network Numerous thin-walled vascular channels with erythrocytes thread between tumour cells in all views. Brown fat is highly vascular; this vascularity is typical of hibernoma and helps separate the lobules.  Scant or absent mature univacuolated adipocytes Classical white-fat cells with peripheral nuclei are sparse or absent. Confirms the lesion is not a conventional lipoma but a brown-fat tumour.  Collectively, the combination of a circumscribed lobulated lesion, sheets of multivacuolated eosinophilic adipocytes with central bland nuclei, and abundant capillaries is diagnostic of hibernoma within the adipocytic tumour group of oral and maxillofacial pathology. |
| 6.41 | Diagnosis: Embryonal rhabdomyosarcoma; Key Microscopic Features: A cellular neoplasm containing myxoid regions, perivascular clusters of tumor cells offering a diagnostic clue, and more differentiated cells appearing polygonal or elongated with eosinophilic cytoplasm. | Based on the provided hematoxylin and eosin (H&E) stained histological images at varying magnifications, and considering the classification within the group of vascular, neural, adipocytic, muscle, and myofibroblastic tumors in oral and maxillofacial pathology, the most likely diagnosis is:  Single Most Likely Histopathological Diagnosis:  Leiomyoma (benign smooth muscle tumor)  Key Microscopic Features Supporting This Diagnosis:  Low-Power View (First Image):  The tumor appears well-circumscribed and unencapsulated.  There is a uniform architecture, lacking infiltrative borders or necrosis.  Densely cellular areas with interlacing fascicles.  Intermediate-Power View (Second Image):  Fascicles of spindle-shaped cells arranged in intersecting bundles or whorled patterns.  Perivascular arrangement of tumor cells (indicative of vascular leiomyoma variant).  No significant pleomorphism or atypical mitoses.  Presence of elongated nuclei with blunt ends ("cigar-shaped" nuclei), typical of smooth muscle origin.  High-Power View (Third Image):  Spindle cells with eosinophilic cytoplasm and centrally located blunt-ended nuclei.  No evidence of cellular atypia, high mitotic activity, or necrosis.  Some fibrous stroma interspersed among tumor cells.  Absence of lipoblasts (rules out adipocytic tumors), neural features (Antoni A/B patterns), or striations (which would suggest skeletal muscle origin).  Summary Justification:  The overall architecture, cytologic features, and growth pattern are classic for a leiomyoma, particularly the angioleiomyoma variant given the prominent vascular component. These features help distinguish it from other spindle cell tumors in the differential diagnosis such as neurofibroma, schwannoma, myofibroma, or lipoma. | Histopathological diagnosis – Solitary Myofibroma (myofibromatosis‐type lesion)  Key microscopic features visible in the three H&E fields  Image Magnification Salient findings that build the diagnostic picture  1 (low-power overview) ~×20 Solid, nodular proliferation that is fairly well-circumscribed but un-encapsulated, showing a biphasic, mottled architecture – darker, highly cellular zones alternating with paler, more collagenised areas. Several slit-like, branching (“staghorn”) vascular spaces are scattered throughout, hinting at a haemangiopericytomatous component.  2 (intermediate power) ~×100 A medium-sized vessel is surrounded by concentric whorls of spindle cells – the classic “onion-skin” perivascular pattern characteristic of myofibroma. The spindle cells are uniform, with tapering nuclei and scant eosinophilic cytoplasm; no appreciable cytologic atypia or atypical mitoses are present.  3 (high power) ~×200–400 The tumour is composed of bland myofibroblasts arranged in short fascicles and interlacing bundles. Between them are keloid-like, thick eosinophilic collagen fibres. Nuclei are ovoid–spindle, finely chromatic, and mitotic activity is very low – all features indicating a benign myofibroblastic process.  How these features point to myofibroma  Biphasic pattern with hemangiopericytoma-like vessels: Myofibroma typically shows alternating primitive cellular areas and more collagenous, mature myoid areas surrounding branching thin-walled vessels.  Perivascular “onion-skin” whorls: Concentric layering of spindle cells around vessels is highly characteristic and helps separate myofibroma from other spindle-cell lesions (e.g., leiomyoma or solitary fibrous tumour).  Uniform myofibroblasts with scant cytologic atypia and low mitotic count: Confirms a benign myofibroblastic proliferation rather than a sarcoma.  Keloidal collagen and short interlacing fascicles: A common stromal signature of myofibroma, not typically seen in neural, adipocytic, or pure smooth-muscle tumours.  Taken together, the constellation of a circumscribed biphasic spindle-cell mass, perivascular whorling, haemangiopericytomatous vasculature and bland myofibroblasts most convincingly supports the diagnosis of solitary myofibroma in the myofibroblastic-muscle-vascular tumour group of the oral and maxillofacial region. |
| 6.42 | **Diagnosis:** Alveolar-pattern rhabdomyosarcoma; **Key Microscopic Features:** Small, round, basophilic tumor cells form alveolar clusters with central cell drop-out, and the cells exhibit round shapes with atypical nuclei and occasional multinucleation. | Based on the two provided hematoxylin and eosin (H&E) stained histological images of the same lesion from the vascular, neural, adipocytic, muscle, and myofibroblastic tumors group in oral and maxillofacial pathology, here is the analysis:  🔍 Single Most Likely Histopathological Diagnosis:  Hemangiopericytoma / Myopericytoma spectrum tumor  (now often categorized under myopericytoma in WHO classification)  🔑 Key Microscopic Features Observed:  Numerous Thin-Walled, Staghorn-Shaped Vessels:  A prominent hallmark of hemangiopericytoma-like tumors.  These vessels have irregular lumens and are lined by a single layer of endothelial cells.  The vascular spaces are surrounded by a proliferation of spindle-shaped to oval pericytic/myoid-appearing cells.  Concentric Perivascular Cell Arrangement:  At higher magnification (second image), there's a clear onion-skin or whorled pattern of myoid cells encircling blood vessels.  This perivascular growth pattern is characteristic of tumors within the myopericytoma spectrum.  Uniform, Bland Spindle Cells:  The tumor cells show minimal atypia, with small, oval nuclei and eosinophilic cytoplasm.  Lack of significant pleomorphism or mitotic activity suggests a benign or low-grade lesion.  Fibrous Septa & Lobular Architecture:  Seen especially in the lower-power image (first image), the lesion appears to be divided into lobules by fibrous connective tissue.  Absence of Necrosis or High Mitotic Rate:  These features support a benign or intermediate-grade diagnosis rather than a malignant counterpart (e.g., malignant pericytic tumor or angiosarcoma).  🧠 Summary:  The characteristic vascular architecture (staghorn-shaped vessels), concentric perivascular arrangement of bland spindle/myoid cells, and lobulated architecture are most consistent with a myopericytoma, which lies within the hemangiopericytoma-like spectrum of tumors. | Histopathological diagnosis  Lobular capillary hemangioma (-“pyogenic granuloma”)  Key microscopic features that support this diagnosis  Low-power findings High-power findings Diagnostic significance  Distinct lobular (nodular) architecture – the lesion is broken up by delicate fibrous septa into multiple round-to-ovoid lobules. Closely packed, capillary-sized vascular channels within each lobule. Lumina are either slit-like or distended and often contain erythrocytes. The lobular pattern is the single most characteristic feature of a lobular capillary hemangioma and distinguishes it from ordinary capillary or cavernous hemangioma.  Prominent, variably cellular fibromyxoid septa separating the lobules. Plump but cytologically bland endothelial cells line the vascular spaces; nuclei are oval, uniform, with inconspicuous nucleoli and no significant atypia. Bland endothelium and lack of infiltrative growth exclude angiosarcoma and other malignant vascular tumours.  Overall lesion is well circumscribed but not encapsulated. Scattered inflammatory cells (mainly neutrophils and lymphocytes) can be present in septa and around vessels, reflecting the reactive/ulcer-prone nature of the lesion. A mild inflammatory background is typical for pyogenic granuloma and helps separate it from purely neoplastic vascular proliferations.  Taken together, the lobular pattern of tightly packed capillaries, bland endothelial lining, fibrous septa, and mild inflammation are classic for a lobular capillary hemangioma (pyogenic granuloma) of the oral cavity. |
| 6.43 | **Diagnosis:** Spindle cell and sclerosing variant of rhabdomyosarcoma; **Key Microscopic Features:** A highly cellular neoplasm composed of spindle-shaped and round cells intersected by thick, sclerotic collagen bands, with individual tumor cells displaying hyperchromatic nuclei in both spindle and round forms. | Based on the histopathological analysis of the provided H&E-stained sections at both low and high magnification, the most likely diagnosis is:  Myofibroma (Myofibromatosis)  Key Microscopic Features Supporting This Diagnosis:  Biphasic Growth Pattern (seen best in low magnification):  Alternating hypercellular areas with spindle cells and hypocellular zones with a more myxoid or collagenized stroma.  Areas with hemangiopericytoma-like vascular pattern, i.e., staghorn or branching blood vessels.  Cellular Composition (seen best in high magnification):  Spindle-shaped cells arranged in fascicles or whorled patterns.  Plump nuclei with fine chromatin, inconspicuous nucleoli, and eosinophilic cytoplasm.  Absence of significant nuclear atypia or high mitotic activity.  Vascular Features:  Numerous small to medium-sized thin-walled blood vessels, often surrounded by a cuff of tumor cells, typical of a pericytic tumor pattern.  No overt signs of malignancy:  Despite being cellular, the tumor does not show pleomorphism, atypical mitoses, or necrosis, distinguishing it from more aggressive spindle cell tumors (like leiomyosarcoma or MPNST).  Rationale for Tumor Group Classification:  This lesion fits into the myofibroblastic tumor group within oral and maxillofacial pathology due to the presence of spindle cells with myofibroblastic differentiation, fascicular growth, and the hemangiopericytoma-like vascular pattern. | Single most likely histopathological diagnosis  Angioleiomyoma (vascular leiomyoma) – a benign smooth-muscle tumour that arises from the tunica media of small- to medium-sized blood vessels.  Key microscopic features seen in the two H-E photomicrographs  Low-power field High-power field Diagnostic significance  • Sharply circumscribed, highly cellular nodule composed of interlacing and sweeping fascicles running in different directions.  • Fascicles are punctuated by many round to oval eosinophilic structures that represent thick-walled vascular channels; several are cut longitudinally (elongated pink bands) and others transversely (solid oval pink islands). • Tumour cells are uniform spindle cells with abundant eosinophilic cytoplasm and blunt-ended “cigar-shaped” nuclei typical of smooth-muscle differentiation.  • Nuclei show minimal pleomorphism, inconspicuous nucleoli and very low mitotic activity (no atypical mitoses).  • Smooth-muscle bundles are seen merging with or cuffing the vascular walls, a hallmark of angioleiomyoma.  • Scattered capillaries, a few extravasated red cells and an occasional adipocyte at the periphery may be present but are not part of the tumour proper. • A fascicular smooth-muscle pattern plus numerous thick, muscularized blood vessels is characteristic of angioleiomyoma and distinguishes it from other spindle-cell tumours in the vascular/neural/adipocytic/muscle/myofibroblastic group (e.g., schwannoma lacks muscular vessels; myofibroma shows hemangiopericytoma-like thin-walled vessels; leiomyosarcoma would display significant atypia and mitoses).  Taken together, the combination of interlacing smooth-muscle fascicles intimately associated with thick-walled vascular channels and the bland cigar-shaped nuclei with low mitotic activity supports the diagnosis of angioleiomyoma. |
| 6.45 | **Diagnosis:** Benign myofibromatous tumor; **Key Microscopic Features:** A proliferation of spindle-shaped cells beneath an ulcerated surface forming short fascicles, with zones of increased cellularity where the spindle cells show bland nuclear characteristics. | Based on the histopathological features observed across the three progressively magnified hematoxylin-and-eosin (H&E) stained images of the lesion, the most likely diagnosis is:  Leiomyosarcoma  Key Microscopic Features Supporting the Diagnosis:  1. Low-Power View (Image 1):  Ill-defined lesion within the submucosa, showing infiltrative growth.  Absence of a well-circumscribed capsule suggests a malignant process.  Overlying mucosa appears intact, often seen in deep-seated tumors.  2. Intermediate Magnification (Image 2):  Fascicular pattern of spindle-shaped cells arranged in interlacing bundles or whorls.  Suggestive of smooth muscle differentiation.  No clear evidence of adipocytes or nerve bundles, helping exclude lipomatous or neural tumors.  3. High-Power View (Image 3):  Hyperchromatic, pleomorphic nuclei with prominent nucleoli.  High mitotic activity, including atypical mitoses.  Eosinophilic cytoplasm in spindle cells, consistent with smooth muscle origin.  Absence of cross striations helps rule out skeletal muscle origin (e.g., rhabdomyosarcoma).  Differential Consideration Ruled Out:  Schwannoma/Neurofibroma: Lack of Verocay bodies or nerve features.  Liposarcoma: No lipoblasts or adipocytic differentiation.  Fibrosarcoma: Typically shows more herringbone pattern.  Myofibrosarcoma: Overlaps but often has more collagen and less eosinophilic cytoplasm.  If available, immunohistochemistry (IHC) would help confirm:  Positive for: SMA (Smooth Muscle Actin), desmin.  Negative for: S-100 (rules out neural origin), MyoD1 (rules out skeletal muscle origin). | Histopathological diagnosis (single best fit)  Nodular fasciitis (pseudosarcomatous myofibroblastic proliferation) of the oral submucosa  Microscopic hallmarks that support this diagnosis  Field Key observations Significance  Low-power view • Unencapsulated, poorly circumscribed nodule lying immediately beneath intact stratified squamous mucosa.  • Slight heterogeneity (“zonation”) with more cellular upper half and paler, more collagenised lower half. Typical for nodular fasciitis, which grows rapidly but remains confined to the superficial fascia / submucosa.  Intermediate magnification • Short, intersecting and swirling fascicles of spindle cells giving a “tissue-culture” pattern.  • Alternating myxoid and more fibrous areas. The haphazard, loose fascicular arrangement is characteristic; smooth-muscle tumours show longer, parallel bundles, and neural tumours have wavy nuclei.  High magnification • Plump spindle-to-oval myofibroblasts with vesicular chromatin and small nucleoli; cytoplasm pale-eosinophilic, not the deeply eosinophilic fibrillary cytoplasm of true smooth-muscle cells.  • Extravasated erythrocytes and numerous delicate, thin-walled capillaries scattered among the cells.  • Occasional mitotic figures, but no atypical mitoses or significant nuclear pleomorphism. The combination of tissue-culture-like myofibroblasts, plentiful RBC extravasation and thin-walled vessels is classic for nodular fasciitis. Presence of mitoses without atypia helps exclude sarcoma; lack of cigar-shaped nuclei excludes leiomyoma/leiomyosarcoma; absence of Verocay bodies or wavy nuclei excludes schwannoma/neurofibroma.  Together, these features pin the lesion as nodular fasciitis – a benign, self-limited myofibroblastic proliferation that often mimics a sarcoma histologically yet lacks significant atypia or infiltrative destruction. |
| 6.46 | **Diagnosis:** Cellular variant of myofibroma; **Key Microscopic Features:** A multinodular lesion predominantly uniphasic, composed of spindle cells arranged in fascicles and occasional nodules demonstrating early myoid differentiation. | Based on the provided hematoxylin and eosin (H&E) stained images at increasing magnifications, the most likely histopathological diagnosis is:  Schwannoma (Neurilemmoma)  Key Microscopic Features Supporting the Diagnosis:  Encapsulation:  Seen clearly in the lowest magnification image (first image), the lesion is well-circumscribed and encapsulated, typical of a benign peripheral nerve sheath tumor like schwannoma.  Antoni A and Antoni B Areas:  Higher magnification images (especially second and third) show alternating dense (Antoni A) and loose (Antoni B) areas.  Antoni A areas are hypercellular, with spindle cells arranged in palisades or interlacing fascicles.  Antoni B areas are more myxoid and hypocellular, giving a looser appearance.  Verocay Bodies:  Within the Antoni A areas (most visible in the second image), there are formations suggestive of Verocay bodies—areas where palisading nuclei are separated by anuclear zones.  This is a hallmark of schwannoma.  Spindle Cell Morphology:  Uniform spindle-shaped cells with elongated, wavy nuclei are abundant throughout the lesion.  Absence of Nuclear Atypia or Mitotic Activity:  No significant pleomorphism or atypical mitotic figures are visible, supporting a benign nature.  Conclusion:  The histologic appearance across all magnifications — encapsulation, Antoni A/B patterns, Verocay bodies, and spindle cell morphology — is diagnostic for Schwannoma, a neural tumor often found in the oral and maxillofacial region. | Histopathological diagnosis – Solitary (localized) neurofibroma  Key microscopic clues seen in the three H-E fields  Field What you can see Why it points to neurofibroma  Low-power overview (left-most image) • A well-circumscribed, but not truly encapsulated, spindle-cell nodule in the submucosa.  • At the right edge, a concentric, eosinophilic “ring” structure representing a residual nerve fascicle invested by perineurium and swallowed by the tumour. Neurofibromas grow within the parent nerve, so the parent fascicle is often found coursing through or caught inside the mass, unlike schwannoma in which it is displaced to the periphery and the lesion is fully encapsulated.  Intermediate magnification (centre image) • Uniform, interlacing fascicles of spindle cells blending into one another without the biphasic Antoni A / Antoni B pattern or Verocay bodies that characterise schwannoma.  • No distinct thick-walled vessels or vascular channels that would suggest an angioleiomyoma. The diffuse, pattern-less, intertwining bundles with a stream-and-swirl appearance are typical of neurofibroma. Absence of palisading or encapsulation makes schwannoma unlikely; absence of herring-bone pattern and mitotic activity makes fibrosarcoma unlikely.  High-power (right-most image) • Spindle cells with elongated, wavy “comma-shaped” (serpentine) nuclei, scant pale cytoplasm and tapered ends.  • Cells lie in a delicate collagenous / fibrillary stroma with scattered small vessels; mast cells are often present (not easy to appreciate at this resolution).  • Minimal pleomorphism, rare mitoses, no necrosis. The nuclear contour and background matrix are classic for Schwann-cell-rich neurofibroma. Lack of blunt “cigar-shaped” nuclei and dense eosinophilic cytoplasm argues against smooth-muscle tumours (leiomyoma/angioleiomyoma); lack of tissue-culture-like fibroblasts and myxoid zones argues against nodular fasciitis.  Bottom line  The combination of an intraneural, poorly encapsulated but circumscribed spindle-cell proliferation composed of uniform Schwann-type cells with wavy nuclei and delicate collagen, and a trapped parent nerve fascicle, is diagnostic of a neurofibroma in the neural category of spindle-cell tumours of the oral and maxillofacial region. |
| 6.47 | **Diagnosis:** Myopericytic neoplasm; **Key Microscopic Features:** Pale spindle cells encircle vascular channels with denser cellular regions at the periphery; cells vary in cytoplasmic volume, some displaying abundant pale cytoplasm while others with scant cytoplasm are associated with smaller vessels. | Histopathological Analysis of Provided Images:  📌 Final Diagnosis:  Neurofibroma  🧠 Key Microscopic Features Supporting the Diagnosis:  1. Low-Power View (Image 1):  Well-circumscribed but non-encapsulated lesion expanding the subcutaneous tissue.  The lesion is composed of a loosely arranged spindle cell proliferation.  There is wavy collagen in a myxoid matrix, which is classic for neurofibroma.  Entrapped structures (e.g., adnexal glands or fat) at the periphery, supporting its infiltrative (non-encapsulated) growth pattern.  2. Intermediate-Power View (Image 2):  Spindle cells with wavy, serpentine nuclei, characteristic of Schwann cell differentiation.  No significant nuclear atypia or mitotic activity, supporting a benign nature.  Occasional perivascular cuffing and involvement of nerve fascicles, a hallmark of neurofibroma.  Presence of fibromyxoid stroma with scattered mast cells, which is a common feature.  3. High-Power View (Image 3):  Closer look confirms the bland spindle cells with elongated, buckled ("comma-shaped" or "serpentine") nuclei.  Scattered mast cells are also visible, which are often abundant in neurofibromas.  No necrosis or atypical mitoses, further supporting a benign peripheral nerve sheath tumor.  🧬 Differential Diagnoses Considered (and ruled out):  Schwannoma: Ruled out due to lack of Verocay bodies and encapsulation.  Traumatic neuroma: Ruled out due to lack of haphazard nerve regeneration pattern.  Dermatofibrosarcoma protuberans: Would show storiform pattern and infiltrative dermal involvement.  Spindle cell lipoma or myofibroma: Would show adipocytic or myoid features not seen here. | Most likely histopathological diagnosis  Solitary myofibroma ( = oral/soft-tissue variant of infantile myofibromatosis)  Key microscopic features that support this diagnosis  Field Findings seen in the provided H-E sections Diagnostic significance  Overall architecture (low-power) • Well-circumscribed, oval nodule surrounded by a thin fibrous pseudo-capsule.  • Characteristic biphasic “zoning” pattern – a pale, less-cellular peripheral zone rims a darker, highly cellular core. The zonation phenomenon is classic for myofibroma and helps separate it from other smooth-muscle or fibrous lesions.  Vascular pattern • Numerous thin-walled, branching (“staghorn”/hemangiopericytoma-like) vessels in the dark core.  • Some medium-sized vessels with concentric whorls of tumour cells around the lumen (second picture, centre). A hemangiopericytomatous vascular tree in combination with myofibroblasts is a hallmark of myofibroma.  Cell morphology (high-power) • Two cell populations: ↳ Central/core: densely packed plump spindle-to-ovoid cells with scant cytoplasm and hyperchromatic nuclei (left half of the third image). ↳ Peripheral: more elongated myoid spindle cells with eosinophilic cytoplasm embedded in hyalinised collagen (right half of third image).  • Nuclei are tapering/blunt-ended; pleomorphism minimal; mitoses infrequent. Dual cytology (primitive-looking core + mature myoid periphery) is typical of myofibroma and distinguishes it from leiomyoma (uniform smooth-muscle) and nodular fasciitis (single cell type with “tissue-culture” appearance).  Stroma & additional clues • Interlacing fascicles and sweeping whorls of tumour cells around vessels.  • Interspersed islands of skeletal muscle fibres and small foci of haemorrhage/RBC extravasation (first image). Muscle entrapment is common when the lesion arises in the oral cavity/tongue; extravasated RBCs are another frequent feature.  Cytologic behaviour • No coagulative tumour necrosis, significant atypia or high mitotic index. Supports a benign myofibroblastic proliferation rather than leiomyosarcoma or malignant peripheral nerve-sheath tumour.  Correlative immunohistochemistry (not shown but expected): diffuse SMA +, muscle-specific actin +; desmin usually negative or only patchy; β-catenin negative – a profile consistent with a myofibroblastic rather than smooth-muscle lineage.  Taken together, the circumscription, biphasic zoning, hemangiopericytomatous vasculature, dual spindle-cell population, low cytologic atypia and oral soft-tissue location firmly point to solitary myofibroma. |
| 6.48 | **Diagnosis:** Synovial‐type soft tissue sarcoma; **Key Microscopic Features:** Dense fascicles of spindle-shaped cells characterized by a high nuclear-to-cytoplasmic ratio and intensely hyperchromatic nuclei. | Based on the provided hematoxylin and eosin (H&E) stained histological images of the same lesion (low and high magnification) from the group of vascular, neural, adipocytic, muscle, and myofibroblastic tumors in oral and maxillofacial pathology, the most likely histopathological diagnosis is:  Diagnosis: Leiomyosarcoma  Justification and Key Microscopic Features:  Spindle Cell Proliferation (Low Power Image):  The first image (low magnification) shows a fascicular arrangement of spindle-shaped cells, typical of smooth muscle origin.  There is a streaming or intersecting fascicle pattern with dense cellularity, supporting a sarcomatous process.  Cellular Atypia and Pleomorphism (High Power Image):  The second image (high magnification) reveals spindle cells with elongated, hyperchromatic nuclei, nuclear pleomorphism, and eosinophilic cytoplasm—hallmarks of smooth muscle differentiation.  Mitotic figures are present, and abnormal mitoses may be identifiable, indicating malignancy.  Necrosis or Increased Mitotic Activity (Implied):  Although necrosis isn’t clearly visible in the provided images, the cytologic atypia and high mitotic activity are sufficient to raise concern for malignancy in a smooth muscle tumor.  Exclusion of Other Entities:  No features of adipocytes (ruling out liposarcoma).  Lack of vascular channels rules out angiosarcoma or hemangiopericytoma.  Absence of neural whorling or palisading patterns (ruling out schwannoma or neurofibroma).  Myofibroblastic tumors are usually less pleomorphic and more collagenous.  Conclusion:  The overall histomorphology strongly supports leiomyosarcoma, a malignant smooth muscle tumor, which—though rare—can occur in the oral and maxillofacial region. | Most likely histopathological diagnosis  → Embryonal rhabdomyosarcoma (skeletal-muscle-type sarcoma)  Key microscopic features seen in the two H-&-E fields that support this diagnosis  Field What is seen Why it points to embryonal rhabdomyosarcoma  Low-power (first image) • A highly cellular, non-encapsulated lesion replacing the connective tissue  • Alternating more compact fascicular areas and slightly looser/myxoid zones  • Scattered thin-walled vascular channels coursing through the tumour The zonal pattern (“dark–light” areas) and fascicular growth are typical of embryonal RMS; abundant but delicate vasculature is common in muscle-derived sarcomas.  High-power (second image) • Mixture of cell types:  – Small round to oval primitive cells with hyperchromatic nuclei  – Spindle cells arranged in short intersecting bundles  – Eosinophilic “strap”/“tadpole” rhabdomyoblasts with eccentric nuclei and amphophilic-to-bright eosinophilic cytoplasm (some show faint cross-striations)  • Moderate nuclear pleomorphism and brisk mitotic activity The heterogenous population (primitive round cells + spindle cells + rhabdomyoblasts) is the signature triad of embryonal RMS. Strap cells with deeply eosinophilic cytoplasm are pathognomonic for skeletal-muscle differentiation. Frequent mitoses confirm malignancy.  Synopsis of the pattern  Skeletal-muscle differentiation is evidenced by strap/tadpole-shaped rhabdomyoblasts with abundant eosinophilic cytoplasm.  Embryonal architecture is indicated by the admixture of primitive round cells and spindle cells set in varying cellularity, rather than the fibrous-septa/alveolar spaces of alveolar RMS or the parallel fascicles of a leiomyosarcoma.  Location in the head-and-neck region further favours embryonal RMS, the commonest subtype in this site.  Together, these features make embryonal rhabdomyosarcoma the single most fitting diagnosis for the tumour shown. |
| 6.49 | **Diagnosis:** Nodular reactive fasciitis; **Key Microscopic Features:** A cellular proliferation of spindle myofibroblasts arranged in fascicles with chronic inflammation and recent hemorrhage, set within a feathery fascicular pattern in a myxoid stroma containing scattered lymphocytes, the cells demonstrating mild variation in size and shape with frequent normal mitotic figures. | Based on the provided hematoxylin and eosin (H&E) stained histological images at increasing magnifications, the single most likely histopathological diagnosis is:  Leiomyoma (Benign Smooth Muscle Tumor)  Key Microscopic Features Justifying the Diagnosis:  Fascicular Pattern of Spindle Cells:  Seen prominently in all magnifications, particularly in the low-power view.  The tumor is composed of interlacing bundles and fascicles of spindle-shaped cells with eosinophilic cytoplasm, arranged in a whorled or interlacing pattern—hallmarks of a smooth muscle origin.  Uniform, Blunt-Ended (Cigar-Shaped) Nuclei:  At higher magnification, the cells exhibit characteristic elongated, blunt-ended nuclei with minimal pleomorphism, which is highly suggestive of smooth muscle differentiation.  Lack of Significant Atypia or Mitotic Figures:  There is no appreciable cytologic atypia, necrosis, or abnormal mitoses—supporting the benign nature of the lesion.  Low Vascularity and Absence of Lipoblasts or Neural Features:  No adipocytic differentiation (rules out lipomatous tumors), no wavy nuclei or Verocay bodies (rules out neural tumors), and the vascularity is not dominant (argues against vascular tumors like hemangioma).  Conclusion:  The histopathological features—particularly the whorled fascicular pattern of bland spindle cells with eosinophilic cytoplasm and cigar-shaped nuclei—are most consistent with leiomyoma, a benign smooth muscle tumor. This tumor can occur in the oral and maxillofacial region, although it is relatively rare in that location. | Most likely histopathological diagnosis  → Benign smooth-muscle tumour (leiomyoma) – oral/maxillofacial angioleiomyoma subtype  Microscopic features in the three H-E fields that support this diagnosis  Field What is seen Why it points to leiomyoma  Low-power overview (first image) • A well-circumscribed mass composed of broad, sweeping inter-lacing fascicles that form whorled/spiral (“pin-wheel”) patterns.  • No infiltrative edges or necrosis. A characteristic architecture of benign smooth-muscle tumours; the orderly whorled pattern rules out nodular fasciitis, fibrosarcoma, etc.  Intermediate magnification (second image) • Fascicles are made of uniform spindle cells with pale-pink, fibrillary cytoplasm.  • Scattered small vascular lumina containing erythrocytes; several bundles appear to merge with or cuff these vessels (angioleiomyoma pattern). Uniformity and vascular association favour a benign leiomyoma; angioleiomyoma is the commonest oral variant.  High power (third image) • Each cell shows abundant eosinophilic cytoplasm and an elongated, blunt-ended “cigar-shaped” nucleus, oriented parallel to the long axis of the cell.  • Nuclei are bland, with finely granular chromatin; mitotic figures and pleomorphism are virtually absent.  • Occasional perinuclear vacuoles typical of smooth-muscle cells. The cytologic features are diagnostic for smooth-muscle differentiation and, in the absence of atypia or mitoses, establish a benign lesion.  Summarised key points  Interlacing, whorled fascicles of bland spindle cells.  Cells with eosinophilic cytoplasm and blunt “cigar” nuclei.  Scant mitoses, no atypia or necrosis → benign behaviour.  Scattered, often thick-walled vascular channels cuffed by the smooth-muscle bundles → angioleiomyoma pattern typical of oral leiomyoma.  Together these features most convincingly fit a benign oral leiomyoma (angioleiomyoma). |
| 6.50 | **Diagnosis:** Desmoid‐type fibromatosis; **Key Microscopic Features:** A spindle cell proliferation embedded in a dense collagenous stroma, with individual cells exhibiting tapering cytoplasmic extensions and bland nuclei showing minimal pleomorphism. | Histopathological Diagnosis:  Leiomyoma (a benign smooth muscle tumor)  Justification with Key Microscopic Features:  Across the three provided H&E-stained histological sections at increasing magnifications, the following characteristic features support the diagnosis of leiomyoma:  1. Spindle Cell Proliferation in Fascicles (Low to High Magnification):  The lesion demonstrates intersecting fascicles of uniform spindle-shaped cells, which is classic for smooth muscle tumors.  The cells have eosinophilic cytoplasm and elongated, blunt-ended ("cigar-shaped") nuclei, typical of smooth muscle differentiation.  2. Minimal Atypia and Mitosis:  No significant cytologic atypia or mitotic figures are observed, supporting a benign nature.  Nuclear pleomorphism is absent or minimal.  3. Vascular Channels (Low Magnification):  There is evidence of thin-walled blood vessels, but without proliferation or features of vascular neoplasms (e.g., hemangioma).  This supports a well-vascularized benign mesenchymal tumor rather than a vascular-origin lesion.  4. Absence of Other Lineage Differentiation:  No evidence of neural features (e.g., wavy nuclei of Schwann cells), lipogenic differentiation (no adipocytes), or skeletal muscle characteristics (e.g., cross striations).  Also, no prominent myxoid matrix or features to suggest myofibroblastic neoplasms.  Summary:  The spindle cell lesion with intersecting fascicles of bland smooth muscle cells, minimal mitotic activity, and absence of significant atypia is diagnostic of a leiomyoma. These features are consistent with a tumor from the muscle group of oral and maxillofacial pathology. | Histopathological diagnosis  Leiomyoma – solid/angioleiomyoma-type smooth-muscle tumour of the oral/maxillofacial region.  Key microscopic features that support the diagnosis  Feature What is seen in the slides Importance for diagnosis  Architecture Well-circumscribed nodule composed of tightly packed, interlacing fascicles that sweep and swirl, producing a whorled appearance (best appreciated on the low-power image). Classic growth pattern of a benign smooth-muscle tumour.  Cell morphology Uniform spindle cells with abundant eosinophilic, fibrillary cytoplasm and blunt, “cigar-shaped” nuclei; occasional perinuclear clear zones; nuclei parallel the long axis of each cell. The blunt-ended nucleus and eosinophilic cytoplasm distinguish smooth-muscle cells from fibroblasts or Schwann cells.  Vascular component Numerous small to medium calibre vascular channels, some with relatively thick muscular walls, are intimately invested by the tumour fascicles; scattered extravasated erythrocytes are present. Smooth-muscle cells arising from or wrapping around vessels typify an angioleiomyoma variant.  Cytologic blandness Minimal pleomorphism, inconspicuous nucleoli, and very rare/absent mitotic figures; no necrosis. Confirms a benign lesion and rules out leiomyosarcoma or other spindle-cell sarcomas.  Stroma Delicate collagen between fascicles; no myxoid change, storiform fibroblastic pattern, Verocay bodies, or shredded-carrot collagen. Helps exclude myofibroma, solitary fibrous tumour, neurofibroma, etc.  The combination of a whorled, fascicular spindle-cell proliferation composed of cytologically bland smooth-muscle cells tightly associated with vascular channels, and the absence of atypia or significant mitoses, is diagnostic of a benign smooth-muscle tumour – most consistent with a leiomyoma (angiomyoma/vascular leiomyoma) in the oral and maxillofacial setting. |
| 6.52 | **Diagnosis:** Benign lipomatous tumor; **Key Microscopic Features:** A well-circumscribed nodule of adipocytes separated by collagenous septa, where fat cells display uniform vacuolated cytoplasm and are free of nuclear atypia or fibrotic change. | Histopathological Analysis  Single Most Likely Diagnosis:  Spindle Cell Lipoma (SCL)  Key Microscopic Features Justifying the Diagnosis:  1. Gross Architecture (Low Magnification – First Image):  A well-circumscribed, unencapsulated lesion located in the subcutaneous tissue.  Lobulated architecture composed of adipose tissue separated by fibrous septa.  Peripheral fibrous capsule is seen partially, typical of a benign lesion.  2. Intermediate Magnification (Second Image):  Mixture of mature adipocytes and spindle cells arranged in parallel fascicles.  Spindle cells are bland, uniform, and embedded in a collagenous or myxoid stroma.  There is a lack of significant atypia or mitotic activity.  Ropy collagen bundles present between spindle cells, which is characteristic of spindle cell lipoma.  Sparse vascularity.  3. High Magnification (Third Image):  The spindle cells show elongated nuclei with inconspicuous nucleoli, and eosinophilic cytoplasm.  Mature adipocytes are present, varying in size but with no nuclear atypia.  Collagen fibers have a wiry or ropy quality, again reinforcing the diagnosis.  Scattered mast cells may be appreciated, a subtle but supportive feature.  Differential Consideration Excluded:  Liposarcoma: No lipoblasts or nuclear atypia; no mitotic figures or infiltrative borders.  Neurofibroma: Lacks the admixture with mature adipocytes and ropy collagen pattern.  Angiolipoma: No significant vascular proliferation or thrombi.  Myxoid tumors (e.g., myxoid liposarcoma): The myxoid matrix here is not prominent or abundant, and lacks characteristic vascular pattern ("chicken wire").  Conclusion:  The histological findings of a well-demarcated lesion composed of bland spindle cells, ropy collagen, and mature adipose tissue without atypia or mitoses are diagnostic of Spindle Cell Lipoma, a benign adipocytic neoplasm often found in the oral/maxillofacial region, particularly the neck and back but occasionally in the oral cavity. | Histopathological diagnosis  Fibrolipoma – the fibrous (sclerotic) variant of a conventional lipoma.  Key microscopic features visible in the three levels  Field Salient findings Diagnostic value  Low-power overview (Image 1) Sharply circumscribed, thinly encapsulated, submucosal nodule showing a lobulated architecture; thick, eosinophilic fibrous septa radiate from the capsule and divide the mass into multiple smaller lobules of pale cells. A well-defined, lobulated fatty lesion with a prominent fibro-collagenous framework is typical of a fibrolipoma rather than an infiltrative process or a liposarcoma.  Intermediate magnification (Image 2) Lobules are composed of numerous mature univacuolated adipocytes (empty “white” cells after lipid dissolution) that are intersected by broad, dense collagen bundles; no increased vascularity or myxoid matrix is present. Abundant dense collagen separating islands of mature fat is the defining histologic criterion for fibrolipoma.  Lippincott  High magnification (Image 3) * Uniform adipocytes with small, inconspicuous, eccentrically placed nuclei. * Dense, pink, rope-like collagen fibres containing only scattered bland spindle/fibroblast-like nuclei. * Absence of lipoblasts, cellular pleomorphism, mitoses or atypia. Confirms benign nature (no lipoblasts / atypia) and emphasises the disproportionately large fibrous component that distinguishes fibrolipoma from ordinary lipoma and from spindle-cell or pleomorphic lipoma.  Lippincott  How these features lead to the diagnosis  Mature adipocyte population → rules out myxoid, pleomorphic or atypical liposarcoma.  Prominent, sclerotic fibrous septa and trabeculae accounting for a sizable part of the tumour volume → separates the lesion from a simple lipoma (which shows only delicate septa) and from angiolipoma (which has numerous capillaries).  Scant bland spindle cells, no rope-y collagen whorls or floret-type giant cells → argues against spindle-cell/pleomorphic lipoma.  Well-circumscribed/encapsulated growth beneath intact oral mucosa → supports a benign, soft-tissue lipomatous tumour rather than an infiltrative fibromatosis or malignant neoplasm.  Taken together, the constellation of mature fat lobules plus abundant dense fibrous connective tissue septa in a sharply circumscribed oral submucosal nodule is diagnostic of fibrolipoma. |
| 6.53 | **Diagnosis:** Lipoma with osseous differentiation; **Key Microscopic Features:** A lipomatous neoplasm containing osteoid and immature woven bone with osteoclasts, all surrounded by prominent, plump spindle cells. | Histopathological Diagnosis:  Intravascular Papillary Endothelial Hyperplasia (IPEH) (also known as Masson's Tumor)  Key Microscopic Features Supporting the Diagnosis:  Papillary structures with endothelial lining within vascular spaces:  Both images clearly show intravascular proliferations forming papillary projections. These are lined by a single layer of bland endothelial cells.  The papillae often have a hyalinized fibrous core, which is typical of IPEH.  Well-circumscribed lesion within vascular lumen:  In the low-power image, the lesion appears well-circumscribed and confined within a dilated vascular space, without evidence of infiltrative growth into adjacent adipose tissue or muscle.  Lack of significant cytologic atypia or mitotic activity:  At higher magnification, the endothelial cells are bland, lacking nuclear pleomorphism or hyperchromasia, and mitotic figures are absent, which helps distinguish it from angiosarcoma.  Associated thrombus:  IPEH often arises in association with an organizing thrombus, which appears to be present in the images as eosinophilic material within the vascular lumen, acting as a scaffold for endothelial proliferation.  Conclusion:  The described features are classic for Intravascular Papillary Endothelial Hyperplasia (IPEH) — a benign, reactive vascular proliferation that must be distinguished from malignant vascular tumors like angiosarcoma. Its well-circumscribed intravascular nature, papillary architecture, and bland cytology are the key diagnostic clues. | Histopathological diagnosis  Osteolipoma (ossifying lipoma) – a variant of intramuscular / soft-tissue lipoma with extensive mature osseous metaplasia  Key microscopic features visible in the two H-E sections  Feature How it appears in the images Why it supports osteolipoma  Abundant mature adipose tissue The background of both fields is occupied by large, clear vacuolated cells with peripheral nuclei – classic mature adipocytes. Confirms the adipocytic (lipomatous) nature of the lesion.  Broad, anastomosing trabeculae of mature lamellar bone At low power the lesion shows pink, branching trabeculae coursing through the fat; at high power the trabeculae have parallel lamellae and osteocytes in lacunae. Indicates well-formed, metaplastic bone rather than dystrophic calcification or immature osteoid.  Osteoblastic rimming / fibrous peri-trabecular stroma The bony spicules are lined by a cuff of flattened to cuboidal osteoblast-like cells and delicate fibrovascular stroma with scattered spindle cells (seen best in the high-power image). Shows active but benign bone formation typical of an ossifying lipoma rather than malignant bone-forming tumors.  Absence of cytologic atypia or lipoblasts Neither the adipocytes nor spindle cells display pleomorphism, hyperchromasia, mitoses, or lipoblasts. Rules out well-differentiated / dedifferentiated liposarcoma and other sarcomas.  Lack of prominent smooth-muscle bundles or thick-walled vessels No interlacing smooth-muscle fascicles (would suggest myolipoma/angiomyolipoma) and vessels are unremarkable. Helps narrow the diagnosis to an ossifying variant of lipoma rather than other mixed soft-tissue tumors.  Together, the intimate admixture of mature fat and mature lamellar bone without atypia is diagnostic of osteolipoma. |
| 6.54 | **Diagnosis:** Traumatized lipoma; **Key Microscopic Features:** A lipoma in the tongue mucosa characterized by adipocytes of diverse sizes accompanied by fibrosis, with fat cells containing variably sized vacuoles. | Based on the provided hematoxylin-and-eosin stained images at low and high magnification, the most likely single histopathological diagnosis is:  Spindle Cell Lipoma  Key Microscopic Features Justifying the Diagnosis:  1. Presence of Mature Adipocytes:  Both images show numerous mature fat cells (adipocytes), which appear as clear vacuolated cells due to fat dissolution during tissue processing.  These adipocytes are interspersed within a collagenous stroma.  2. Bland Spindle Cells:  In the high-magnification image, you can see a population of bland-appearing spindle cells with elongated nuclei and no significant atypia or mitotic activity.  These cells are evenly distributed within the collagen-rich matrix.  3. Ropey Collagen:  A characteristic feature of spindle cell lipoma is the presence of thick, "ropey" collagen bundles, which are visible in the background stroma.  4. Myxoid to Collagenous Stroma:  The tumor matrix alternates between myxoid (loose) and more collagenized areas, a feature common in spindle cell lipomas.  5. Absence of Cytologic Atypia or Necrosis:  No atypical mitotic figures, necrosis, or pleomorphism is noted, ruling out liposarcoma and other malignant entities.  6. Location and Submucosal Involvement (Based on Context):  Spindle cell lipomas often occur in the head and neck region, including the oral cavity, and are submucosal, consistent with the anatomical site of this lesion. | Single best histopathological diagnosis  Spindle-cell lipoma (oral submucosal variant – an adipocytic tumour)  Key microscopic features that point to this diagnosis  Feature (seen in the submitted H&E photomicrographs) Why it supports spindle-cell lipoma  1. Well-circumscribed, non-encapsulated nodule just beneath an intact stratified squamous mucosa (low-power image) Typical location for an oral spindle-cell lipoma; lesion grows as a discrete submucosal mass without infiltrative borders.  2. Intimate admixture of two cell populations:  • Mature adipocytes of variable size, forming small lobules and single cells  • Uniform bland spindle cells with elongated nuclei and scant cytoplasm (high-power image) The defining biphasic pattern of spindle-cell lipoma – unlike a conventional lipoma (virtually all fat) or a pure fibrous lesion (no fat).  3. Characteristic thick, eosinophilic “ropey” collagen bundles weaving between the spindle cells (best appreciated at high power) “Ropey” collagen is a hallmark of spindle-/pleomorphic lipoma and is absent in most other fatty tumours.  4. Very scant mitotic activity, no nuclear atypia, no lipoblasts, no plexiform capillary network Helps exclude myxoid/atypical liposarcoma, lipoblastoma, or other sarcomas.  5. Relative paucity of vascular channels Rules against angiolipoma (which shows numerous small vessels, often with fibrin thrombi).  6. Skeletal muscle fibres at the deep edge are merely compressed, not invaded Supports a benign, pushing growth pattern typical of spindle-cell lipoma.  Taken together, these findings fit perfectly with spindle-cell lipoma, an uncommon but recognised adipocytic tumour in the oral and maxillofacial region that shows a mix of mature fat, bland spindle cells and ropey collagen in a myxoid back-ground. |
| 6.55 | **Diagnosis:** Traumatized lipoma; **Key Microscopic Features:** A nonencapsulated adipocytic neoplasm composed of adipocytes with lipid vacuoles of varying dimensions alongside spindle-shaped stromal cells embedded in delicate collagen fibers, also displaying vacuoles of assorted sizes. | Histopathological Diagnosis:  Spindle Cell Lipoma  Key Microscopic Features Justifying the Diagnosis:  Well-Circumscribed and Submucosal Lesion (first image, low magnification):  The lesion is clearly demarcated from the surrounding tissue and located beneath the squamous epithelium.  Absence of infiltrative margins suggests a benign neoplasm.  Mature Adipocytes Mixed with Bland Spindle Cells (second image, intermediate magnification):  Admixture of mature fat cells with uniform, bland spindle cells is a hallmark of spindle cell lipoma.  The spindle cells are arranged in short fascicles and embedded in a myxoid or collagenous stroma.  Ropey Collagen and Myxoid Background (third image, high magnification):  Presence of eosinophilic "ropey" collagen bundles interspersed among spindle cells.  The stroma is myxoid, a typical finding in spindle cell lipoma.  Low Cellular Atypia and Mitotic Activity:  There is no evidence of pleomorphism or increased mitotic figures, supporting a benign nature.  Absence of Vascular Proliferation, Neural Bundles, or Myogenic Differentiation:  These features help rule out angiolipoma, neurofibroma, and myofibroblastic tumors.  Spindle cell lipoma is a benign soft tissue tumor often occurring in the neck, back, or shoulder of older men but can also appear in the oral and maxillofacial region. The histologic triad of mature adipocytes, spindle cells, and ropey collagen is diagnostic. | Histopathological diagnosis  Fibrolipoma (a variant of lipoma that contains a conspicuous fibrous connective-tissue component)  Key microscopic features in the three H-E fields that support this diagnosis  Image Magnification Features seen Diagnostic significance  1 (low-power overview) Whole nodule • Well-circumscribed, dome-shaped, submucosal mass lying immediately beneath an intact stratified-squamous oral epithelium.  • The bulk of the lesion is pale-eosinophilic and hypocellular. A pushing border without infiltration and preservation of the overlying mucosa are in keeping with a benign mesenchymal tumour such as a lipoma.  2 (intermediate) ×100 • Countless round to oval clear vacuoles of various sizes representing fat droplets removed during processing.  • These vacuoles are embedded in broad, eosinophilic collagen bundles that weave through the tumour.  • Sparse, bland spindle nuclei (fibroblasts) and only an occasional thin-walled capillary; no atypia, mitoses or lipoblasts. The admixture of mature adipocytes and abundant fibrous stroma is the defining histology of a fibrolipoma.  3 (high-power) ×200–400 • Individual adipocytes with thin rims of cytoplasm and flattened, eccentrically placed nuclei (at this resolution the nucleus is sometimes obscured but its position is implied by the crescent-shaped cytoplasmic rim).  • Inter-adipocyte spaces are traversed by thick collagen fibres, confirming the fibrous component is at least equal in volume to the fat.  • No nuclear pleomorphism, hyperchromasia, necrosis or “chicken-wire” capillary network—features that would raise concern for myxoid or other liposarcoma. Mature adipocytes without atypia plus dense collagen distinguish fibrolipoma from other adipocytic or myxoid lesions and rule out malignancy.  Reasoning recap  A conventional lipoma would show lobules of almost pure mature fat with only delicate septa.  Here, thick, hyalinised collagen bundles occupy a large proportion of the tumour, interlacing between, and sometimes splitting, the adipocytes. When a benign lipomatous tumour contains ≥ 50 % fibrous tissue it is classified as a fibrolipoma. The lack of cellular atypia, mitotic activity, pleomorphism, or the characteristic vascular pattern of a myxoid liposarcoma further supports this benign fibrolipomatous diagnosis. |
| 6.57 | **Diagnosis:** Spindle cell lipoma; **Key Microscopic Features:** An admixture of mature adipocytes and spindle-shaped cells set within both thick, rope-like collagen bundles and fine collagen fibers, with scattered mast cells throughout. | Based on the analysis of the three hematoxylin and eosin (H&E)-stained histopathology images of the same lesion taken at increasing magnifications, the single most likely histopathological diagnosis is:  Spindle Cell Lipoma  Key Microscopic Features Supporting This Diagnosis:  Mature Adipocytes:  Numerous mature adipocytes (white, vacuolated round cells) are scattered throughout the lesion, particularly evident in the low-power image. This is characteristic of lipomatous tumors.  Spindle Cell Component:  Intermixed with the fat are bland spindle cells arranged in parallel fascicles. These cells have elongated nuclei with no significant atypia or mitotic activity, as seen more clearly in the intermediate- and high-power images.  Ropey Collagen:  The presence of thick, rope-like eosinophilic collagen bundles running between the spindle cells is a hallmark feature of spindle cell lipoma.  Myxoid Stroma:  There are areas with a loose, myxoid matrix, especially visible at higher magnification, which is commonly seen in spindle cell lipomas.  Low Cellular Atypia and Absence of Mitotic Figures:  The nuclei appear bland with minimal pleomorphism and no abnormal mitoses, which supports a benign nature.  No Evidence of Necrosis or Infiltrative Growth:  The lesion shows a well-circumscribed architecture without signs of tissue invasion, favoring a benign tumor.  Conclusion:  The constellation of mature fat, bland spindle cells, ropey collagen, and absence of atypia/mitotic activity is diagnostic of a spindle cell lipoma, a benign soft tissue tumor that can occur in the oral and maxillofacial region, typically in older adults. | Most likely histopathological diagnosis  → Spindle-cell lipoma (oral variant)  Key microscopic features that point to this diagnosis  Feature seen in the photomicrographs Diagnostic significance  Intimate admixture of two components – plentiful mature univacuolated adipocytes (white round vacuoles) interwoven with a cellular spindle-cell proliferation Classic biphasic pattern of spindle-cell lipoma; ordinary fibrolipoma would show broad fibrous septa separating fat rather than spindle cells intermingling with it  Bland spindle cells with long, slender, sometimes wavy nuclei, sparse cytoplasm and no significant pleomorphism, mitoses or necrosis Confirms a benign lesion and matches the fibro-/myxoid spindle cells of spindle-cell lipoma  “Ropey”, thick, eosinophilic collagen bundles coursing between the spindle cells (best appreciated in the mid-power image) Virtually pathognomonic for spindle-cell lipoma/pleomorphic lipoma  Loosely myxoid background stroma in parts of the field Another characteristic, especially in oral and parapharyngeal spindle-cell lipomas  Scattered delicate thin-walled capillaries and numerous mast cells (dark-purple, granular cytoplasm; best seen on high power) Frequently present supportive findings  Absence of atypical multivacuolated lipoblasts, marked cellular atypia, high mitotic rate, or staghorn-type branching vessels Helps exclude well-differentiated/pleomorphic liposarcoma, solitary fibrous tumour, etc.  The constellation of mature adipocytes + uniform bland spindle cells + ropey collagen in a well-circumscribed lesion is diagnostic of spindle-cell lipoma, a benign adipocytic tumour that, although classically found in the posterior neck/shoulder of older men, also occurs in the oral and maxillofacial region. |
| 6.58 | **Diagnosis:** Lipid granuloma; **Key Microscopic Features:** Marked fibrosis with chronic inflammatory cells containing vacuoles of various sizes, and lipid vacuoles of both small and large caliber, some located within multinucleated giant cells and surrounded by macrophages. | Based on the analysis of the two provided hematoxylin and eosin (H&E) stained histological images at different magnifications, from the group of vascular, neural, adipocytic, muscle, and myofibroblastic tumors in oral and maxillofacial pathology, the single most likely histopathological diagnosis is:  Spindle Cell Lipoma  Key Microscopic Features Supporting the Diagnosis:  Admixture of Mature Adipocytes and Spindle Cells:  The lesion is composed of a mixture of mature fat cells and uniform spindle-shaped cells arranged in parallel bundles or fascicles.  This is best appreciated in the lower magnification image, where lobules of adipocytes are intermixed with cellular fibrous areas.  Myxoid Background with Collagen Bundles:  The background stroma shows myxoid changes and ropey collagen fibers, a hallmark of spindle cell lipoma.  These collagen bundles are pale pink and wavy, interspersed between spindle cells.  Lack of Significant Atypia or Mitosis:  The spindle cells are bland, with no marked pleomorphism or atypical mitoses, which helps rule out sarcomatous processes.  Scattered Mast Cells:  On higher magnification, scattered mast cells (with granulated cytoplasm) may be observed, which is another common feature of spindle cell lipoma.  Vascularity:  Presence of small to medium-sized vessels without the prominent vascular pattern seen in vascular tumors like hemangiomas or angiolipomas.  This tumor is benign and typically occurs in the posterior neck, back, and shoulder regions, but when found in the oral cavity, it commonly involves the buccal mucosa and tongue. | Histopathological diagnosis (single best):  Angiomyolipoma (extrarenal/oral soft-tissue type – a PEComa)  Key microscopic features supporting this diagnosis  Feature seen in the photomicrographs Diagnostic significance  Intimate admixture of three tissue elements – numerous mature adipocytes (clear‐vacuo­lated cells), interlacing fascicles of smooth-muscle-type spindle cells (eosinophilic cytoplasm, cigar-shaped nuclei) and thick-walled, sometimes tortuous blood vessels The classic triad that defines an angiomyolipoma (“angio-” = vessel, “myo-” = smooth muscle, “lipoma” = fat)  Bundles of smooth-muscle cells appear to emanate from, or wrap around, the vascular walls Characteristic radial or concentric arrangement of myoid cells around dysplastic vessels found in AML  Thick, hyalinised vascular walls with variable calibre and occasional lumina containing erythrocytes Prominent, often dysplastic, vessels are one of the three compulsory components  Mature adipocytes of variable size scattered haphazardly through the myoid/vascular stroma with no capsule separating the components Confirms that the fatty component is part of the tumour rather than entrapped normal fat  Overall paucity of cytologic atypia and very low mitotic activity Corroborates a benign PEComa rather than a liposarcoma or other malignant adipocytic neoplasm  Whorled / swirling architecture produced by the interlacing smooth-muscle fascicles at low power Typical growth pattern of angiomyolipoma in soft tissue  The simultaneous presence, in the same lesion, of abundant mature fat, conspicuous thick-walled vessels and smooth-muscle bundles is essentially pathognomonic for angiomyolipoma and distinguishes it from other adipocytic (lipoma/liposarcoma), vascular (angioleiomyoma) or purely myogenic tumours encountered in the oral and maxillofacial region. |
| 6.59 | **Diagnosis:** Atypical lipomatous tumor; **Key Microscopic Features:** Lobules of fat cells that appear mature interspersed with strands of atypical cells bearing large, hyperchromatic nuclei; atypical adipocytes or lipoblasts that are ovoid to spindle in shape with pleomorphic nuclei; and lipoblasts demonstrating multiple vacuoles, multinucleation, and irregular, hyperchromatic nuclei. | Based on the three hematoxylin and eosin (H&E) stained histopathology images provided—taken at low, intermediate, and high magnification—this lesion is consistent with a pleomorphic lipoma, a benign adipocytic tumor that typically arises in subcutaneous tissues and may occasionally be seen in the oral and maxillofacial region.  Histopathological Diagnosis:  Pleomorphic lipoma  Key Microscopic Features Supporting the Diagnosis:  Low Magnification (Image 1):  Lobulated architecture: The lesion shows a lobulated pattern with alternating hypocellular and cellular areas.  Adipocytic component: Scattered mature adipocytes are present within fibrous stroma.  Intermediate Magnification (Image 2):  Spindle cell proliferation: There is a proliferation of bland spindle cells arranged in a myxoid or collagenous stroma.  Ropey collagen: Dense, eosinophilic “ropey” collagen bundles are evident, which are characteristic of pleomorphic lipoma.  High Magnification (Image 3):  Floret-like multinucleated giant cells: Classic feature of pleomorphic lipoma, where cells have multiple nuclei arranged in a wreath-like or floret pattern.  Lack of mitotic activity or atypia: Despite cellular pleomorphism, the nuclei are bland without evidence of significant atypia or mitotic figures.  Background adipocytes: The presence of mature adipocytes further supports the adipocytic lineage of this tumor.  Conclusion:  The constellation of mature adipocytes, spindle cells in fibrous stroma, ropey collagen, and floret-like multinucleated giant cells—especially in the absence of atypia or necrosis—confirms the diagnosis of pleomorphic lipoma, a benign entity in the adipocytic tumor family.  If you need immunohistochemical correlations or a differential diagnosis list (e.g., atypical lipomatous tumor, myxoid liposarcoma, etc.), I’d be happy to assist further. | Histopathological diagnosis  Atypical lipomatous tumor / well-differentiated liposarcoma (inflammatory-sclerosing variant).  Key microscopic features in the three fields that support this diagnosis  Field What is seen Why it matters  Low-power overview (image 1) • Lobulated mass dominated by mature adipocytes separated by broad fibrous septa.  • Septa are hypercellular, showing whorling bundles of spindle cells and a lymphoplasmacytic infiltrate. A benign lipoma lacks fibrous septa with cellular/atypical elements. Thick septa packed with cells and inflammation is typical of the inflammatory/sclerosing variant of well-differentiated (WD) liposarcoma.  Intermediate magnification (image 2) • Central fibromyxoid zone packed with bland-to-slightly pleomorphic spindle cells, admixed with numerous lymphocytes and plasma cells (“inflammatory” component).  • Mature adipocytes at the periphery vary moderately in size. The juxtaposition of cellular fibrous areas and mature fat, together with a prominent chronic inflammatory infiltrate, is characteristic of the inflammatory WD liposarcoma subset.  High-power detail (image 3) • Scattered hyperchromatic atypical stromal cells within fibrous septa, some multinucleated.  • True lipoblasts: multivacuolated cytoplasm with indented, hyperchromatic nuclei. Identification of lipoblasts and markedly atypical stromal cells is the sine-qua-non for a malignant adipocytic lesion and distinguishes atypical lipomatous tumor/WD-liposarcoma from spindle-cell or pleomorphic lipoma.  Putting the features together  Mature fat lobules with size variability – baseline adipocytic component.  Fibrosclerotic septa that are cellular and contain atypical hyperchromatic cells – hallmark of WD-liposarcoma.  Definite lipoblasts – diagnostic of a malignant adipocytic tumor.  Prominent chronic inflammatory infiltrate – places the lesion in the “inflammatory” variant of atypical lipomatous tumor / WD-liposarcoma seen in head–neck sites.  The constellation of these findings rules out benign lipoma (no atypia or lipoblasts), spindle/pleomorphic lipoma (floret-type giant cells but no lipoblasts), and myxoid liposarcoma (abundant myxoid matrix with delicate “chicken-wire” vessels, absent here). |
| 6.61 | **Diagnosis:** Solitary fibrous tumor, fibrous type; **Key Microscopic Features:** A lesion formed of densely collagenous fibrous tissue, featuring areas of hyalinized collagen adjacent to delicate, vascularized collagen zones populated by spindle cells, some of which exhibit pleomorphic, hyperchromatic nuclei. | Histopathological Analysis of the Lesion (H&E-stained sections)  Single Most Likely Histopathological Diagnosis:  Myxoma (Odontogenic Myxoma)  Key Microscopic Features Supporting the Diagnosis:  Low Power View (First Image):  The lesion is hypocellular and composed predominantly of myxoid stroma.  The stroma appears loosely arranged, with sweeping fascicles and few visible cells.  Absence of encapsulation but well-demarcated from adjacent tissue.  Intermediate Power View (Second Image):  More evident loose, mucoid extracellular matrix rich in ground substance.  Scattered stellate- to spindle-shaped fibroblastic cells embedded within the stroma.  No significant atypia or mitotic figures are observed.  Occasional delicate collagen fibers may be interspersed.  High Power View (Third Image):  The stellate/spindle-shaped cells have small, bland nuclei and scant cytoplasm.  Myxoid matrix is prominent, with light basophilic to amphophilic staining.  No evidence of necrosis, hemorrhage, or aggressive cellular features.  Diagnostic Justification and Differential Exclusion:  Neural tumors (e.g., neurofibroma) typically have wavy nuclei and more fibrous background, lacking the gelatinous stroma.  Adipocytic tumors (e.g., lipoma) would show mature adipocytes, which are not seen here.  Muscle tumors (e.g., leiomyoma) show more cellularity, cigar-shaped nuclei, and eosinophilic cytoplasm.  Myofibroblastic lesions (e.g., myofibroma) are typically more cellular and less myxoid.  Conclusion:  The constellation of features — hypocellularity, myxoid stroma, bland stellate cells, and absence of atypia — is diagnostic of myxoma, most likely odontogenic myxoma in the context of oral/maxillofacial pathology. | Histopathologic diagnosis  Desmoplastic fibroblastoma ( = collagenous fibroma) – a benign fibro-/myofibroblastic soft-tissue tumour.  Key microscopic features visible in the three H-E fields  Feature What you can see in the images How it supports the diagnosis  Paucicellular, well-circumscribed mass Low-power image shows a broad, sharply delimited nodule with a smooth outline and very few nuclei scattered through it. Desmoplastic fibroblastoma is classically a well-circumscribed but unencapsulated lesion that is strikingly hypocellular.  Abundant dense, eosinophilic “ropey” or hyalinised collagen Medium- and high-power views reveal thick, wavy, glassy-pink bundles of collagen occupying most of the tumour volume. The lesion’s hallmark is copious, collagen-rich matrix that dwarfs the cells; the collagen often has a rope-like or keloidal appearance.  Widely spaced bland fibroblastic / myofibroblastic cells High-power field shows spindle-to-stellate cells with small, dark, ovoid nuclei and inconspicuous cytoplasm set far apart in the collagen. Mitoses, pleomorphism, and hyperchromasia are absent. Desmoplastic fibroblastoma contains only scattered, cytologically bland fibroblasts or myofibroblasts without atypia or mitotic activity.  Interlacing / whorled pattern of collagen fascicles Low-power image demonstrates sweeping, interlacing fascicles that create a subtly whorled architecture. Although hypocellular, the collagen is laid down in intersecting fascicles, giving the characteristic pattern described for this tumour.  Entrapment of adjacent tissue without destruction In the medium-power photo the lesion insinuates between pre-existing muscle / fibrous tissue at the periphery but does not display infiltrative destruction. Desmoplastic fibroblastoma frequently interdigitates with surrounding structures in a pushing fashion rather than a frankly infiltrative one.  The combination of very low cellularity, bland fibroblastic cells, and abundant thick eosinophilic collagen laid down in sweeping fascicles is quintessential for desmoplastic fibroblastoma and effectively excludes more cellular entities such as fibromatosis, neurofibroma, nodular fasciitis or smooth-muscle tumours. |
| 6.62 | **Diagnosis:** Solitary fibrous tumor, cellular type; **Key Microscopic Features:** A spindle cell proliferation with alternating hypercellular and hypocellular regions and staghorn vascular patterns, including dilated vessels encircled by plump spindle cells, and spindle cells bearing rounded to ovoid nuclei that appear benign but are mildly hyperchromatic. | Based on the provided hematoxylin and eosin (H&E) stained histopathological images at low, intermediate, and high magnification, the features are consistent with a diagnosis of:  Diagnosis:  Kaposi Sarcoma  Key Microscopic Features Supporting the Diagnosis:  Low Power (Image 1):  Nodular architecture with a poorly circumscribed lesion extending through the dermis/submucosa.  Presence of slit-like vascular spaces in a vaguely lobular arrangement.  Epidermis or mucosal epithelium may appear relatively unremarkable or slightly thinned.  Intermediate Power (Image 2):  Proliferation of spindle cells arranged in interlacing fascicles.  Numerous irregular, angulated vascular spaces interspersed among the spindle cell proliferation.  Red blood cells are often seen extravasated within these vascular channels.  High Power (Image 3):  Spindle cell population shows moderate nuclear atypia with hyperchromatic, elongated nuclei.  Prominent vascular slit-like spaces with extravasated erythrocytes.  Hemosiderin-laden macrophages and plasma cells can be seen scattered in the stroma.  No significant mitotic activity, but mild pleomorphism is noted.  Additional Notes:  Kaposi sarcoma is a vascular neoplasm associated with HHV-8 (Human Herpesvirus 8) infection. It commonly occurs in immunocompromised patients (e.g., HIV/AIDS), although endemic and iatrogenic forms exist. Oral involvement often presents on the palate, gingiva, or tongue.  If immunohistochemical stains are performed, HHV-8 positivity in spindle cells would confirm the diagnosis.  Let me know if you need a differential diagnosis or comparison with similar vascular/myofibroblastic tumors like angiosarcoma or spindle cell hemangioma. | Histopathological diagnosis  → Solitary myofibroma (oral myofibromatosis)  Key microscopic features visible in the three H-E sections  Magnification What is seen Why it points to myofibroma  Low power (first slide) • Sharply demarcated, subepithelial nodule pushing into the underlying connective tissue.  • Central pale/necrotic-looking zone (“zonation”) surrounded by a more solid peripheral rim.  • Numerous small, slit-like to dilated vascular spaces throughout. Zonation and a hemangiopericytoma-like vascular pattern are classic for myofibroma; the lesion sits immediately beneath intact oral mucosa, as expected for a solitary oral presentation.  Intermediate power (second slide) • Closely packed round-to-spindle cells arranged in interlacing short fascicles and whorls.  • A prominent network of thin-walled, branching (“staghorn”) vessels coursing between the cells.  • Extravasated erythrocytes in the stroma, but no true blood-filled sinusoids. The combination of bland myofibroblasts and a staghorn vascular network reproduces the hemangiopericytoma-like pattern typical of myofibroma.  High power (third slide) • Tumour cells have oval nuclei, finely dispersed chromatin, inconspicuous nucleoli and modest, pale eosinophilic cytoplasm—features of myofibroblasts.  • Scant mitoses, no pleomorphism or atypical mitotic figures.  • Focally collagenised stroma between the cells. Cytologic blandness and myofibroblastic morphology separate this lesion from sarcomas; absence of thick perivascular smooth-muscle bundles rules out angioleiomyoma, and lack of HHV-8-type slit vessels excludes Kaposi sarcoma.  Putting the pattern together  Biphasic zonation (central cellular area ± ischaemic change and peripheral spindle-cell collar).  Hemangiopericytoma-like, branching thin-walled vessels coursing through the lesion.  Bland myofibroblastic cytology with minimal mitotic activity.  These three hallmarks, all present in the supplied images, are diagnostic of solitary myofibroma in the spectrum of vascular, neural, adipocytic, muscle and myofibroblastic tumours of the oral and maxillofacial region. |
| 6.63 | **Diagnosis:** Solitary fibrous tumor, cellular type within a minor salivary gland; **Key Microscopic Features:** Spindle cell proliferation with pronounced hyalinization around salivary ducts and hyalinized bands of collagen, accompanied by prominent multinucleated, pleomorphic tumor cells. | Based on the provided hematoxylin-and-eosin (H&E) stained histological images of the same lesion at various magnifications — within the context of vascular, neural, adipocytic, muscle, and myofibroblastic tumors of the oral and maxillofacial region — the most likely histopathological diagnosis is:  Malignant Peripheral Nerve Sheath Tumor (MPNST)  Key Microscopic Features Supporting This Diagnosis:  Spindle Cell Proliferation (seen in the second image):  Interlacing fascicles of spindle-shaped cells with hyperchromatic, wavy nuclei.  Atypia and nuclear pleomorphism are present.  The arrangement in a storiform to herringbone pattern is characteristic.  Necrosis and Mitotic Activity (clearly seen in the third image):  Evidence of geographic necrosis.  Numerous mitotic figures, some atypical.  Tumor giant cells and areas of hemorrhage can be noted, indicating high-grade malignancy.  Infiltrative Growth Pattern (more discernible in the first image):  Poorly circumscribed lesion invading surrounding soft tissue.  Tumor cells infiltrating in between other structures with no clear capsule.  Vascular Changes:  Scattered abnormal vessels are seen, which is often found in MPNST due to aggressive neoplastic growth and neoangiogenesis.  Lack of Differentiation Toward Other Lineages:  No clear features of adipocytic (e.g., lipoblasts), muscle (e.g., rhabdomyoblasts), or myofibroblastic (e.g., collagen rosettes) differentiation.  Supports the neural origin.  Supporting Differential Features (Ruling Out Others):  Hemangiopericytoma/solitary fibrous tumor: typically has "staghorn" vasculature, which is not prominent here.  Leiomyosarcoma: would show cigar-shaped nuclei and eosinophilic cytoplasm, which are not present.  Liposarcoma: lacks lipoblasts or fatty differentiation.  Myofibrosarcoma: tends to have a more uniform spindle cell population and lacks the degree of atypia and mitoses seen here.  Confirmatory Suggestions:  Immunohistochemistry:  S-100 protein (positive in ~50-60% of MPNSTs).  SOX10 (more sensitive and specific for neural crest origin).  Ki-67 proliferation index (likely high).  Loss of H3K27me3 expression is a newer and specific marker for high-grade MPNSTs. | Histopathological diagnosis (single best):  Solitary myofibroma / myofibromatosis-type myofibroblastic tumour of the oral–maxillofacial region.  Key microscopic features that support the diagnosis  Field Observations in the provided H-E sections Significance  Architectural “zoning” pattern On the low-power overview (first image) the lesion is lobulated and shows alternating dark, hyper-cellular nodules and pale, eosinophilic, hypocellular areas with cyst-like empty spaces. Classic biphasic pattern of a myofibroma: peripheral cellular zones and a central hyalinised/“necrotic” core.  Spindle-cell component Medium- and high-power views (second & third images) display plump, tapering spindle cells with oval to elongated nuclei and scant amphophilic cytoplasm, arranged in interlacing fascicles and whorls. Cytologic atypia is minimal and mitoses are typical. Cytomorphology of myofibroblasts; absence of significant atypia helps separate the lesion from sarcomas.  Hemangiopericytoma-like vasculature Numerous branching, thin-walled, staghorn-shaped vessels are seen; tumour cells frequently show concentric (perivascular) arrangement around them. A hallmark vascular pattern seen in myofibroma (and the reason it used to be called “infantile haemangiopericytoma”).  Central hyalinisation / collagen deposition The pale pink zones are composed of dense, acellular hyalinised collagen with irregular pseudo-cystic spaces. Represents ischaemic change typical of the central portion of a myofibroma nodule.  Extravasated erythrocytes & focal haemorrhage Scattered red blood cells are present between spindle cells (best appreciated in the third image). Common ancillary feature in myofibroma; also seen in nodular fasciitis but, in this case, accompanies the distinctive zoning pattern.  Lack of extensive inflammatory infiltrate or ropy collagen Only scattered inflammatory cells; collagen is not arranged in thick “ropy” bundles. Helps distinguish the lesion from inflammatory myofibroblastic tumour (with heavy plasma-cell infiltrate) and solitary fibrous tumour (prominent ropy collagen).  Together, the biphasic zoning pattern, whorled spindle-cell fascicles around hemangiopericytoma-like vessels, central hyalinisation, and minimal atypia/mitotic activity are diagnostic of a solitary myofibroma in the oral & maxillofacial soft tissues. |
| 6.64 | **Diagnosis:** Ectomesenchymal chondromyxoid tumor of the tongue; **Key Microscopic Features:** A well-defined, nonencapsulated proliferation of spindle cells in a myxoid stroma; a mixture of spindle and epithelioid cells within a chondromyxoid matrix featuring tissue clefts; and epithelioid cells with abundant eosinophilic cytoplasm and eccentrically placed, plasmacytoid nuclei. | Based on the provided hematoxylin and eosin (H&E) stained images of the same lesion at low, medium, and high magnifications, the most likely histopathological diagnosis is:  Granular Cell Tumor  🔍 Justification & Key Microscopic Features:  1. Low Magnification (1st Image):  Poorly circumscribed lesion in the subepithelial connective tissue.  Infiltrative borders with pseudoepitheliomatous hyperplasia of the overlying epithelium.  Tumor extends into the deeper connective tissue without a clear capsule.  2. Intermediate Magnification (2nd Image):  Sheets and nests of polygonal to fusiform cells with indistinct cell borders.  Abundant granular eosinophilic cytoplasm.  Relatively bland nuclei without significant pleomorphism or mitotic activity.  Scattered blood vessels in the background stroma.  3. High Magnification (3rd Image):  Cytoplasm filled with coarse eosinophilic granules — hallmark of lysosome accumulation.  Small, uniform, centrally-located nuclei.  No significant atypia or mitotic figures, supporting a benign nature.  Characteristic appearance of granular cells, with a “ground-glass” granular cytoplasm and indistinct cell boundaries.  📌 Additional Notes:  Granular cell tumors (GCTs) are believed to be of neural origin, particularly from Schwann cells.  Commonly seen in the tongue and oral cavity.  The pseudoepitheliomatous hyperplasia of the overlying epithelium can be mistaken for squamous cell carcinoma if not carefully assessed.  If immunohistochemistry were performed, you'd expect:  S100 positivity, confirming Schwannian origin.  NSE, CD68, and sometimes inhibin positivity.  Let me know if you'd like a labeled version of the images or an explanation comparing this entity to similar lesions (e.g., rhabdomyoma, neurofibroma, or schwannoma). | Most likely histopathological diagnosis  → Granular-cell tumour (Abrikossoff tumour) – a benign Schwann-cell–derived lesion that is common on the oral mucosa (especially tongue) and belongs to the “neural” subset of the vascular, neural, adipocytic, muscle and myofibroblastic tumours group.  Key microscopic features seen in the three H-E fields that support this diagnosis  Image & magnification Salient findings Diagnostic significance  Low-power overview • Solitary, well-circumscribed but unencapsulated submucosal nodule.  • Broad pseudo-epitheliomatous hyperplasia (PEH) of the overlying stratified squamous epithelium with elongated, downward-growing rete ridges.  • Lesion pushes aside but also interdigitates with adjacent skeletal muscle bundles. PEH is a classic reaction over granular-cell tumours and can mimic SCC. Submucosal, poorly encapsulated growth with skeletal-muscle entrapment is typical.  Intermediate power • Uniform, solid sheets and vague fascicles of polygonal to slightly spindled cells separated by delicate collagen.  • Scattered thin-walled vessels; occasional extravasated red cells.  • No appreciable nuclear pleomorphism, necrosis or brisk mitoses. The monotonous cellular pattern without atypia fits a benign neural lesion rather than a sarcoma or myofibroblastic proliferation.  High power • Cells have abundant finely granular, eosinophilic to pale cytoplasm (packed lysosomes).  • Small, round–oval, centrally or eccentrically placed nuclei with bland chromatin.  • Ill-defined cell borders give a syncytial appearance, but occasional clearer outlines are seen.  • No mitotic figures or atypical forms. The granular cytoplasm is pathognomonic for granular-cell tumour; the bland nuclei and absence of mitoses confirm benign behaviour.  Ancillary points (not pictured but characteristic):  Tumour cells are strongly positive for S-100 and SOX10, consistent with Schwann-cell origin.  PAS-D stain highlights the intracytoplasmic granules.  Taken together, the combination of PEH, a poorly encapsulated nodule of large polygonal cells with coarse granular cytoplasm, and bland nuclear features is diagnostic of a granular-cell tumour. |
| 6.65 | **Diagnosis:** Ectomesenchymal chondromyxoid tumor of the tongue; **Key Microscopic Features:** Lobular architecture showing both solid and pseudocystic patterns; a chondromyxoid stroma with tissue clefting; and microcystic areas containing epithelioid cells rich in eosinophilic cytoplasm with benign-appearing nuclei. | Histopathological Diagnosis:  Angiomyolipoma  Key Microscopic Features Justifying the Diagnosis:  Triphasic Composition:  All three images consistently show a triphasic pattern of tissue types, which is hallmark for angiomyolipoma:  Adipose tissue (fat): Seen as numerous round, clear vacuolated spaces due to fat extraction during processing (particularly evident in Image 1).  Thick-walled blood vessels: Seen in both low (Image 1) and intermediate (Image 2) magnification as irregular, sometimes ectatic vessels.  Smooth muscle bundles: Interlacing bundles of eosinophilic spindle cells, visible throughout all images, especially in Images 2 and 3.  Vascular Component:  Numerous prominent thick-walled vessels with irregular contours and absence of a well-defined internal elastic lamina (seen especially in Image 2), consistent with dysplastic vessels characteristic of angiomyolipoma.  Adipocytic Areas:  Clusters of mature adipocytes are visible, most prominent in Image 1. These appear as round clear cells with peripherally displaced nuclei.  Spindle and Epithelioid Smooth Muscle Cells:  The muscle component appears as bundles or fascicles of spindle-shaped cells with eosinophilic cytoplasm (Image 2 and 3).  In Image 3, some epithelioid cells with clear to eosinophilic cytoplasm can be appreciated around vessels, characteristic of the smooth muscle component.  Additional Notes:  Angiomyolipoma belongs to the PEComa family (Perivascular Epithelioid Cell tumors).  While most commonly found in the kidney, extrarenal angiomyolipomas can occur in the oral and maxillofacial region, although rare.  Immunohistochemistry would typically support the diagnosis, with markers such as HMB-45, Melan-A, and smooth muscle actin (SMA) positive.  Let me know if you’d like a diagram or IHC panel associated with this tumor type. | Histopathological diagnosis  Epithelioid hemangio-endothelioma (EHE) – an intermediate-grade vascular tumour of soft tissue that also occurs in the oral and maxillofacial region.  Key microscopic features that support this diagnosis  Field Feature Where it is seen in the provided slides  Architecture • Lobulated/ill-defined mass composed of cords, nests and short trabeculae of tumour cells embedded in a myxoid-to-hyaline stroma Low-power view (1st image): pale purple, slightly basophilic background with intermixed collagen bundles  Tumour cells • Epithelioid endothelial cells: round-to-polygonal shape, moderate eosinophilic cytoplasm, centrally placed round nuclei with inconspicuous nucleoli Medium- and high-power views (2nd & 3rd images)  Intracytoplasmic vacuoles / “blister” cells • Numerous clear round vacuoles, some containing erythrocytes, representing primitive vascular lumina; they impart the scattered “signet-ring” / punch-hole appearance at scanning magnification Scattered white circles in the low-power image; detail of RBC-containing vacuoles in the high-power image  Stroma • Pronounced myxohyaline matrix with eosinophilic (“glassy”) extracellular deposits surrounding and sometimes entrapping tumour nests High-power image shows dense pink hyaline material encircled by tumour cells  True vascular channels • Irregular, blood-filled spaces lined by the same epithelioid cells blend into the cell nests, confirming endothelial differentiation Medium-power image: branching, RBC-filled lumina continuous with tumour cords  Cytologic atypia & mitotic activity • Cells are relatively uniform with mild atypia and low mitotic rate – typical of the intermediate malignancy of EHE and helping to exclude high-grade angiosarcoma Best appreciated in the high-power image  Why other entities are less likely  Epithelioid haemangioma – usually forms well-formed small vessels with a florid eosinophil-rich inflammatory infiltrate, lacking the extensive myxohyaline stroma and intracytoplasmic lumina seen here.  Angiosarcoma – shows marked nuclear pleomorphism, brisk mitoses and destructive anastomosing vascular channels, all absent in these sections.  Metastatic signet-ring carcinoma or alveolar soft-part sarcoma – would not demonstrate the intimate connection of the vacuoles with vascular lumina or the distinctive myxohyaline stroma that typify EHE.  Taken together, the constellation of epithelioid endothelial cells with cytoplasmic vascular lumina, blood-filled spaces merging with cell nests, and a myxohyaline background is diagnostic of epithelioid hemangio-endothelioma. |
| 6.66 | Diagnosis: Juvenile xanthogranulomatous lesion; Key Microscopic Features: A nonencapsulated cellular proliferation composed of sheets of epithelioid cells, with clusters of epithelioid histiocytes intermixed with lymphocytes and multinucleated Touton-type giant cells, including characteristic Touton cells that display a central eosinophilic region, a peripheral ring of nuclei, and an outer vacuolated rim. | Histopathological Diagnosis:  Granular Cell Tumor (GCT)  Key Microscopic Features Supporting the Diagnosis:  The three H&E-stained images represent different magnifications of the same lesion and reveal the following critical features consistent with a Granular Cell Tumor, a neural tumor in the oral and maxillofacial region:  Low Magnification (Image 1):  The lesion is well-circumscribed yet unencapsulated.  It is located in the submucosa, beneath an intact overlying epithelium.  The tumor shows a homogeneous, eosinophilic appearance, suggesting uniform cell population with granular cytoplasm.  Intermediate Magnification (Image 2):  The cells are arranged in sheets or nests.  Individual cells show abundant granular eosinophilic cytoplasm.  The granular appearance is due to cytoplasmic lysosomes, a hallmark of granular cell tumors.  The nuclei are small, round to oval, and centrally placed.  High Magnification (Image 3):  Cells exhibit coarse eosinophilic cytoplasmic granules.  There are small, darkly staining nuclei with little pleomorphism.  Presence of pseudoepitheliomatous hyperplasia in the overlying epithelium is a classic finding (though not obvious here, it may be evident in some sections).  No significant mitotic activity or necrosis is noted, favoring a benign process.  Summary of Supporting Features:  Granular cytoplasm (lysosome-rich).  Unencapsulated, well-demarcated lesion in submucosa.  Sheets/nests of polygonal cells.  Small central nuclei, minimal atypia.  Lack of mitosis or necrosis.  If immunohistochemistry were performed, S-100 positivity would be expected, confirming the Schwann cell origin of this tumor.  Let me know if you’d like a comparison with other neural or soft tissue tumors in this region or to explore immunohistochemical confirmation. | Histopathological diagnosis  → Low-grade fibromyxoid sarcoma (LGFMS) – “hyalinising spindle-cell tumour with giant collagen rosettes.”  Key microscopic features supporting this diagnosis  Field Salient findings visible in the slides Diagnostic significance  Low-power view (slide 1) • Solitary, well-circumscribed but non-encapsulated nodule lying beneath an intact surface.  • Uniform, very pale-pink background with broad, sweeping whorls of slender cells. LGFMS typically grows as a deceptively circumscribed mass composed of bland spindle cells arranged in swirling or storiform patterns.  Intermediate magnification (slide 2) • Alternating zones of lightly myxoid stroma and more densely hyalinised (pink) fibrous stroma (“fibromyxoid” pattern).  • Bland spindle cells with scant cytoplasm and inconspicuous nucleoli lying in short fascicles and arcs. The alternating fibrous-myxoid background with bland spindle cells is a hallmark of LGFMS.  High-power magnification (slide 3) • Multiple giant collagen rosettes: large, round to oval, deeply eosinophilic acellular collagen cores surrounded concentrically by a single layer of tumour cells (rosette).  • Tumour cells remain cytologically bland (small round-to-oval nuclei, fine chromatin, very rare mitoses). The presence of hyaline/giant collagen rosettes is virtually pathognomonic; their recognition converts a generic “fibromyxoid spindle-cell lesion” into LGFMS/HSCT with giant rosettes.  Across all fields • Absence of significant pleomorphism, necrosis or brisk mitotic activity.  • Subtle haemangiopericytoma-like vessels in the myxoid zones. Reinforces the “low-grade” nature of the sarcoma and helps exclude higher-grade mimics.  These combined features – especially the alternating fibrous-myxoid stroma of bland spindle cells and the striking giant collagen rosettes – are characteristic of low-grade fibromyxoid sarcoma / hyalinising spindle-cell tumour with giant rosettes, a tumour included among the fibro-myofibroblastic neoplasms of the head-and-neck soft tissues. |

ULCERATIVE AND INFLAMMATORY CONDITIONS

| **Fig_no** | **Ground truth** | **GPT 4o answer** | **o3 answer** |
| --- | --- | --- | --- |
| 7.2 | **Diagnosis:** Aphthous ulceration; **Key Microscopic Features:** A fibrin-coated ulcer with inflammation confined to the superficial lamina propria, accompanied by epithelial spongiosis, hemorrhage, neutrophil infiltration, and spongiotic pustule formation. | Based on the histopathological analysis of the provided hematoxylin-and-eosin stained images of an oral lesion (at both low and high magnification), the most likely single diagnosis is:  Histopathological Diagnosis:  Lichen Planus  Key Microscopic Features Supporting the Diagnosis:  Saw-toothed Rete Ridges:  The rete ridges show a characteristic "saw-tooth" or pointed appearance, which is typical of lichen planus.  Band-like Lymphocytic Infiltrate:  A dense, band-like infiltrate of lymphocytes is present in the superficial lamina propria, immediately subjacent to the epithelium — a hallmark of lichen planus.  Basal Cell Degeneration (Hydropic Degeneration):  Vacuolar or hydropic degeneration of the basal layer of the epithelium is clearly visible, often with apoptotic (Civatte or colloid) bodies.  Hyperkeratosis and Hypergranulosis:  Although more subtle in ulcerated areas, there is some preservation of surface keratinization and thickening of the granular layer in the non-ulcerated parts.  Ulceration and Secondary Inflammation:  There is focal ulceration of the epithelium, with a corresponding increase in inflammatory cell infiltrate and granulation tissue response beneath.  Interface Mucositis:  The pattern of inflammation shows a lichenoid interface mucositis, with the lymphocytic infiltrate closely abutting and obscuring the epithelial-connective tissue interface.  Notes:  This appearance is classic for ulcerated oral lichen planus, particularly the erosive form, which is commonly symptomatic. The differential diagnosis might include lichenoid drug reaction or chronic ulcerative stomatitis, but the pattern and distribution of the infiltrate, basal layer damage, and lack of plasma cells or eosinophils lean strongly toward lichen planus.  If you need a differential table or immunofluorescence features for confirmation, I can provide that as well. | Histopathological diagnosis  → Oral lichen planus (lichenoid mucositis)  Key microscopic features in the two fields that point to oral lichen planus  # Feature seen How it looks in the submitted sections Diagnostic significance  1 Hyper-/parakeratinised stratified squamous epithelium with saw-tooth rete ridges On the low-power image the surface epithelium is thickened, and the rete pegs are narrow, pointed and angulated. Characteristic architectural change of lichen planus.  2 Liquefaction (hydropic) degeneration of the basal cell layer In the high-power area the basal cells are swollen and vacuolated; small clefts (Max-Josef spaces) separate epithelium from lamina propria in foci. Produced by immune-mediated injury to basal keratinocytes.  3 Civatte (colloid) bodies Numerous round, eosinophilic, apoptotic keratinocytes are scattered through the lower epithelium. Degenerating basal/suprabasal keratinocytes are a classic clue.  4 Band-like, dense, predominantly lymphocytic infiltrate hugging the epithelial interface A continuous sheet of dark-staining lymphocytes occupies the superficial connective tissue immediately beneath the epithelium and even shows focal exocytosis into it. The pathognomonic “lichenoid” inflammatory band.  5 Relative absence of epithelial dysplasia or suprabasal acantholysis Nuclear polarity is maintained and no tomb-stone row or acantholytic cells are present. Helps exclude pemphigus, dysplastic keratoses and other ulcerative entities.  The combination of a lichenoid (band-like) lymphocytic infiltrate, basal-cell liquefaction with Civatte bodies and the saw-tooth hyperkeratotic epithelium is diagnostic of oral lichen planus, one of the prototypical ulcerative/inflammatory mucosal conditions. |
| 7.3 | **Diagnosis:** Traumatic ulcer; **Key Microscopic Features:** A fibrinous membrane overlies granulation tissue with both acute and chronic inflammatory cells, adjacent traumatic parakeratosis harboring surface bacterial colonies, and the epithelium exhibits acanthosis, abundant spongiotic pustules, intraepithelial hemorrhage, and reactive epithelial atypia. | Based on the provided hematoxylin and eosin (H&E) stained histopathology images—showing the same lesion at different magnifications—and within the context of ulcerative and inflammatory conditions of the oral and maxillofacial region, the single most likely histopathological diagnosis is:  Diagnosis:  Traumatic Ulcerative Granuloma with Stromal Eosinophilia (TUGSE)  Key Microscopic Features Supporting the Diagnosis:  Surface Ulceration:  The surface epithelium is disrupted, with areas completely denuded.  Overlying fibrinopurulent membrane is visible, consistent with ulceration.  Dense Inflammatory Infiltrate:  The underlying connective tissue stroma is heavily infiltrated by a mixed inflammatory infiltrate.  Notably rich in eosinophils, along with lymphocytes, plasma cells, and histiocytes.  Deep Infiltration into Skeletal Muscle and Connective Tissue:  Inflammatory cells infiltrate deeply, often dissecting between muscle fibers—a hallmark feature of TUGSE.  Prominent granulomatous and histiocytic response is evident in the deeper tissues.  Atypical/Reactive Large Mononuclear Cells:  Scattered large mononuclear cells resembling Reed-Sternberg–like or histiocyte-like cells may be present, which can cause diagnostic confusion but are part of the reactive nature of TUGSE.  Granulation Tissue Formation:  The base of the ulcer shows evidence of granulation tissue, with new capillary formation and fibroblast proliferation.  Comments:  TUGSE is often mistaken clinically for malignancy due to its indurated, non-healing nature. Histologically, however, the presence of ulceration with a dense eosinophilic infiltrate extending deep into muscle tissue is quite characteristic. It is a benign, self-limiting reactive lesion often associated with local trauma (e.g., biting). | Histopathological diagnosis  → Traumatic (reactive) ulcer of the oral mucosa  (“non-specific chronic/aphthous-type ulcer” – appearances are identical on H&E)  Key microscopic features evident in the two fields  # Feature seen Why it supports the diagnosis  1 Complete loss of surface epithelium at the centre, replaced by a thick fibrinous–purulent membrane (pale pink amorphous material studded with neutrophils and karyorrhectic debris) Defines an ulcer and shows acute inflammatory exudate typical of trauma-related breakdown rather than a vesiculobullous split or true granuloma  2 Margins of the ulcer show hyperplastic, acanthotic stratified squamous epithelium with elongated rete ridges (“epithelial lip”) Reactive epithelial proliferation is characteristic of traumatic ulcers; there is no epithelial dysplasia  3 Heavy mixed inflammatory infiltrate in the ulcer base and adjacent lamina propria – abundant neutrophils superficial, merging into richly vascular granulation tissue with lymphocytes, plasma cells and macrophages deeper down Pattern fits a healing/repairing traumatic ulcer; eosinophils and granulomas that would suggest other entities are not prominent  4 Extravasated erythrocytes and focal haemorrhage within the ulcer bed Consistent with mechanical injury and ongoing tissue breakdown  5 No specific micro-organisms, viral cytopathic change, vasculitis or atypical (dysplastic or malignant) cells Helps exclude infectious, autoimmune–blistering and neoplastic ulcers, leaving a non-specific/traumatic aetiology  Together, these non-specific but classic findings (surface fibrin-neutrophil cap, reactive epithelial borders, granulation tissue rich in mixed inflammatory cells) are exactly what is expected in a traumatic or aphthous-type ulcer of the oral cavity. |
| 7.4 | **Diagnosis:** Neutropenic ulcer; **Key Microscopic Features:** A fibrinous coating overlies granulation tissue, demonstrating a notable paucity of neutrophils within the fibrin network. | Based on the provided hematoxylin and eosin (H&E) stained histopathological images at low and high magnifications, here is the analysis:  Single Most Likely Histopathological Diagnosis:  Traumatic Ulcerative Granuloma with Stromal Eosinophilia (TUGSE)  Key Microscopic Features Supporting the Diagnosis:  Low-Power Image (1st Image):  Ulcerated mucosal surface: Loss of epithelial lining at the surface indicates ulceration.  Dense subepithelial inflammatory infiltrate: The underlying connective tissue is heavily infiltrated, forming a broad zone of inflammation extending deep into the tissue.  Zonal architecture: Inflammatory cells are more concentrated in the center with surrounding collagenous stroma, characteristic of TUGSE.  High-Power Image (2nd Image):  Mixed inflammatory infiltrate: Predominantly includes lymphocytes, histiocytes, plasma cells, and numerous eosinophils—the hallmark of TUGSE.  Large atypical mononuclear cells: Occasionally, large, atypical-appearing histiocyte-like or myofibroblast-like cells may be seen (not overtly malignant).  Muscle fiber infiltration: Some evidence of inflammation extending into skeletal muscle bundles may be noted in typical TUGSE, though less prominent here.  Additional Notes:  TUGSE is a benign, self-limiting lesion often seen on the tongue or other areas prone to trauma in the oral cavity.  It mimics malignancy clinically and sometimes histologically, but lacks true dysplasia or malignant cytology. | istopathological diagnosis (single best):  Non-specific (traumatic) oral ulcer – ulcer bed replaced by granulation tissue  Key microscopic features that support this diagnosis  Level What is seen Why it matters  Surface Complete loss of stratified squamous epithelium, replaced by a thick, amorphous, eosinophilic “fibrin-necrotic” membrane that contains scattered inflammatory cells Confirms that the lesion is an ulcer rather than an intact mucosal process  Ulcer bed Vascular granulation tissue with plump fibroblasts, newly formed capillaries and prominent oedematous collagen This reparative tissue is typical of a healing/traumatic ulcer rather than a specific granulomatous or vesiculobullous disease  Inflammatory infiltrate Mixed acute + chronic cells: numerous neutrophils in the superficial zone, deeper sheets of lymphocytes, plasma cells and foamy macrophages extending vertically into the connective tissue (and between skeletal-muscle fibres in some foci) The mixed picture fits a non-specific, trauma-driven reaction; absence of organised granulomas, vasculitis or pathogen-specific changes helps exclude TB, syphilis, deep fungal infection, etc.  Stroma & deeper tissue Degenerating/necrotic collagen bundles and entrapped muscle fibres rimmed by inflammatory cells; no metaplastic salivary ducts, no atypical epithelial islands, no caseation Rules out necrotising sialometaplasia, malignant ulceration and granulomatous infections  Taken together, the constellation of surface ulceration, a reactive granulation-tissue base, and a polymorphous inflammatory infiltrate without any organism-specific or dysplastic features is classic for a traumatic (non-specific) oral ulcer/ulcerative granuloma. |
| 7.6 | **Diagnosis:** Ulcerative granuloma due to trauma; **Key Microscopic Features:** The oral mucosa exhibits parakeratosis with penetrating inflammation, underlying muscle is infiltrated by inflammatory cells, and myocytes show degenerative changes accompanied by inflammation. | Based on the provided hematoxylin and eosin (H&E) stained histological images taken at low, medium, and high magnifications, the lesion demonstrates features consistent with:  Single Most Likely Histopathological Diagnosis:  Necrotizing sialometaplasia  Key Microscopic Features Supporting This Diagnosis:  1. Lobular architecture preservation (Low magnification)  The glandular architecture is largely preserved, even though there is significant tissue destruction. This is characteristic of necrotizing sialometaplasia and helps distinguish it from malignant neoplasms which usually destroy the lobular pattern.  2. Surface ulceration and pseudoepitheliomatous hyperplasia  The overlying epithelium shows ulceration and reactive hyperplasia, which can mimic squamous cell carcinoma.  3. Necrosis of salivary gland lobules (Medium magnification)  Coagulative necrosis of mucous acini is evident, a hallmark feature of this condition.  4. Squamous metaplasia of salivary ducts (High magnification)  Ductal epithelium undergoes squamous metaplasia, appearing as islands or cords of squamous epithelium, which can mimic invasive carcinoma.  5. Intense inflammatory infiltrate  A dense, mixed inflammatory infiltrate is present, often with neutrophils, lymphocytes, and macrophages. Sometimes, this may also be associated with granulation tissue.  6. Absence of cellular atypia  Despite the squamous metaplasia and necrosis, the epithelial cells typically do not show significant cytological atypia or mitotic activity—key in ruling out malignancy.  Clinical Relevance:  Necrotizing sialometaplasia is a benign, self-limiting, reactive condition often mistaken clinically and histologically for malignancy, especially squamous cell carcinoma or mucoepidermoid carcinoma, due to its ulcerated appearance and squamous metaplasia. | Most likely histopathological diagnosis  → Traumatic ulcerative granuloma with stromal eosinophilia (TUGSE)  (also called the “eosinophilic ulcer” of the oral mucosa)  Key microscopic features that point to TUGSE  Image What you can see Why it matters  Low-power overview (first slide) • Crater-shaped surface ulcer covered by a fibrinous exudate  • Surrounding stratified squamous epithelium shows reactive/ pseudo-epitheliomatous hyperplasia and elongated rete ridges  • A broad, wedge-shaped zone of inflamed tissue extends deep into the lamina propria and underlying skeletal muscle TUGSE characteristically presents as an indurated ulcer whose inflammatory infiltrate tracks deeply, often reaching muscle.  Medium power (second slide) • Polymorphous inflammatory infiltrate filling the ulcer base and permeating skeletal muscle bundles  • Inflammation is ill-defined, not circumscribed, and fans out in a wedge pattern  • Evident degeneration/necrosis of muscle fibres where they are infiltrated The combination of deep muscle involvement + muscle fiber destruction is classic for TUGSE and helps exclude more superficial ulcerative conditions.  High power (third slide) • Dense mixed infiltrate composed of lymphocytes, numerous eosinophils (cells with bright red cytoplasmic granules), macrophages and occasional large atypical immunoblast-like cells  • Eosinophils cluster around and invade degenerating skeletal-muscle fibres  • No true granulomas, no dysplasia or malignancy Stromal eosinophilia together with large, sometimes CD30-positive atypical mononuclear cells and muscle fiber destruction are the diagnostic hallmarks of TUGSE.  Putting the findings together  An ulcerated oral mucosal surface with reactive epithelial hyperplasia.  A deep, wedge-shaped inflammatory process that extends into and destroys skeletal muscle.  A rich eosinophilic and mixed inflammatory infiltrate containing occasional large immunoblast-type cells but no granulomatous structures or neoplastic features.  This constellation is characteristic of traumatic ulcerative granuloma with stromal eosinophilia, rather than other ulcerative/inflammatory entities such as necrotizing sialometaplasia, infectious granulomas, or malignancy. |
| 7.7 | **Diagnosis:** Ulcerative granuloma caused by trauma; **Key Microscopic Features:** The oral mucosa shows frictional parakeratosis, ulceration, and deep invasion by inflammatory cells; skeletal muscle fibers are fragmented by inflammation creating a checkerboard pattern, and remaining muscle fibers are encased by macrophages and eosinophils. | Histopathological Diagnosis:  Necrotizing Sialometaplasia  Key Microscopic Features Supporting the Diagnosis:  The images provided (low, medium, and high magnification) demonstrate classic features consistent with necrotizing sialometaplasia, a benign, self-limiting inflammatory condition primarily affecting salivary glands:  1. Low Magnification (Image 1):  Lobular architecture preserved: Despite necrosis and inflammation, the general salivary gland lobular architecture is maintained—an important distinguishing feature from malignancy.  Ulceration of overlying epithelium: Focal loss or thinning of the mucosal epithelium, often seen in ulcerated lesions.  Centrally located necrosis: Prominent coagulative necrosis affecting salivary gland lobules.  2. Medium Magnification (Image 2):  Ductal squamous metaplasia: Salivary gland ducts appear lined by squamoid epithelium due to metaplasia.  Inflammatory infiltrate: Mixed inflammatory cells including lymphocytes and neutrophils surrounding the necrotic areas.  Glandular disruption: Evident breakdown and transformation of normal glandular epithelium.  3. High Magnification (Image 3):  Squamous metaplasia with nuclear atypia-like features: The metaplastic squamous epithelium may show mild cytologic atypia, but lacks the true malignant features (such as abnormal mitoses or invasive behavior).  Coagulative necrosis: Eosinophilic necrotic cells and debris within glandular structures.  Inflammatory background: Neutrophilic infiltration, edema, and possible vascular changes (suggesting ischemia as a cause).  Key Diagnostic Considerations:  Benign process that can mimic malignancy (e.g., mucoepidermoid carcinoma or squamous cell carcinoma) both clinically and histologically.  Preservation of lobular architecture and absence of infiltrative growth are crucial for ruling out carcinoma. | Histopathological diagnosis (single best):  Necrotizing sialometaplasia (NSM) of a minor salivary gland  Key microscopic features visible in the three H-E images  Field Findings that support NSM  Low-power overview (image 1) • Broad, dome-shaped ulcerated/polypoid area of palate-type mucosa.  • Surface epithelium shows pseudoepitheliomatous hyperplasia with elongated, downward-growing rete ridges.  • Salivary gland lobules are still recognizable—lobular architecture is preserved despite injury (helps rule out an infiltrative carcinoma).  Intermediate magnification (image 2) • Numerous ghost-like, pale eosinophilic mucous acini with loss of nuclei → coagulative necrosis.  • Intermixed bland squamoid / metaplastic ductal islands and cords in the necrotic bed (squamous metaplasia of ducts).  • Prominent mixed inflammatory infiltrate (neutrophils, lymphocytes, macrophages) and early granulation tissue.  High-power (image 3) • Clusters of necrotic mucous cells with preserved outlines (“ghost” acini).  • No significant cytologic atypia or mitotic activity in the metaplastic epithelium—cells are bland, supporting a reactive process rather than carcinoma.  • Surrounding stroma shows vascular congestion, extravasated erythrocytes, and acute-on-chronic inflammatory cells.  Why these features point to necrotizing sialometaplasia  Coagulative necrosis of salivary acini with intact lobular configuration is pathognomonic for NSM.  Squamous (and occasionally pseudo-ductal) metaplasia of residual ducts produces benign-appearing islands that can mimic SCC or mucoepidermoid carcinoma, but here they are bland and non-infiltrative.  Pseudoepitheliomatous hyperplasia of the surface epithelium plus a rich inflammatory/repair response completes the classic picture of this self-limiting ischemic/infarctive lesion. |
| 7.13 | **Diagnosis:** Chronic gingivitis; **Key Microscopic Features:** The epithelium displays pronounced spongiosis with neutrophil transmigration, and the lamina propria contains numerous plasma cells. | Based on the histopathological examination of the provided hematoxylin-and-eosin (H&E) stained images (low and high magnification) and the clinical context of an ulcerative and inflammatory condition in the oral and maxillofacial region:  Single Most Likely Histopathological Diagnosis:  Traumatic Ulcerative Granuloma with Stromal Eosinophilia (TUGSE)  Key Microscopic Features Supporting the Diagnosis:  Low Magnification (Image 1):  Ulcerated Epithelium:  Loss of epithelial continuity at the surface with underlying granulation tissue.  Surrounding epithelium shows hyperplasia and irregular rete ridges.  Dense Inflammatory Infiltrate:  A heavy, diffuse, and deep inflammatory infiltrate extends into the submucosa.  Infiltrate appears polymorphic, extending well beyond superficial zones.  High Magnification (Image 2):  Mixed Inflammatory Cells with Eosinophils:  Prominent eosinophils (characterized by bright red cytoplasmic granules) intermixed with lymphocytes, macrophages, and occasional neutrophils.  Eosinophils are a hallmark of TUGSE and help differentiate it from other chronic ulcers.  Atypical Mononuclear Cells:  Presence of large, activated-appearing lymphoid or histiocytic cells.  These may mimic atypia but are reactive in nature.  Deep Muscle Infiltration (if visible in other sections):  TUGSE often shows an infiltrate that penetrates deep into muscle fibers, although not clearly shown in these fields, it's a known feature.  Comments:  TUGSE often presents clinically as a chronic, non-healing ulcer with indurated margins, typically on the tongue or buccal mucosa.  Histologically, it is important to distinguish it from malignant ulcers, especially due to the presence of atypical mononuclear cells and deep infiltration, but the polymorphic and eosinophil-rich infiltrate is diagnostic. | Histopathological diagnosis (single best fit)  Plasma-cell gingivitis / plasma-cell mucositis (reactive plasmacytosis of the oral mucosa)  Key microscopic features that support this diagnosis  Field Findings in the submitted H-E sections Why the feature is characteristic  Surface epithelium Intact stratified squamous epithelium that is acanthotic with elongated, blunt rete ridges and focal spongiosis/exocytosis of inflammatory cells; no true epithelial dysplasia Plasma-cell mucositis is an inflammatory, not dysplastic, process; the epithelium often shows reactive psoriasiform (pseudo-epitheliomatous) hyperplasia rather than ulceration  Inflammatory infiltrate Immediately beneath the epithelium there is a dense, sheet-like band of mononuclear cells that fills the entire lamina propria and extends around deeper collagen/muscle bundles The diagnostic hallmark of the lesion is a sub-epithelial, diffuse plasmacytic infiltrate that can reach into deeper soft tissues  Cell morphology at high power (second image) The infiltrate is dominated by mature plasma cells showing: 1) round to oval, eccentric nuclei with “clock-face/ cart-wheel” chromatin, 2) abundant amphophilic cytoplasm, and 3) a perinuclear pale zone (hof). Scattered small lymphocytes and occasional histiocytes are present, but eosinophils and granulomas are absent A virtually pure population of mature plasma cells (often > 50 % of the cellularity) is required to make the diagnosis; the nuclear and cytoplasmic features listed are specific for plasma cells  Additional supportive findings • Occasional Russell bodies / Mott cells (eosinophilic cytoplasmic inclusions) within plasma cells  • Dilated, thin-walled capillaries coursing through the infiltrate  • No vasculitis, no necrotising granulomas, no dysplastic epithelial changes These are common secondary changes in reactive plasmacytic lesions and help rule out other ulcerative/inflammatory entities (e.g., lichen planus, traumatic ulcerative granuloma, infectious granulomatous disease)  Taken together, the combination of an intact hyperplastic epithelium and a broad, polymorphous but plasma-cell–heavy lamina-propria infiltrate with the classic “clock-face” nuclear pattern is diagnostic of plasma-cell gingivitis / plasma-cell mucositis in the spectrum of ulcerative and inflammatory conditions of the oral cavity. |
| 7.14 | **Diagnosis:** Chronic hyperplastic gingivitis; **Key Microscopic Features:** The gingival epithelium shows acanthosis and fibrous hyperplasia with a chronic inflammatory infiltrate, while dental plaque bacteria adhere to the surface without an accompanying neutrophil border or Splendore–Hoeppli eosinophilic deposition. | Based on the histopathological evaluation of the provided hematoxylin and eosin (H&E) stained images at low and high magnifications, the single most likely diagnosis is:  Chronic Ulcer with Granulation Tissue and Fibrinopurulent Exudate  Key Microscopic Features Justifying the Diagnosis:  1. Low Magnification (First Image):  Surface ulceration: The epithelial surface is denuded in several areas, suggesting loss of continuity typical of an ulcer.  Pseudoepitheliomatous hyperplasia: The adjacent stratified squamous epithelium is thickened and elongated into the underlying connective tissue, mimicking squamous cell carcinoma but without cytologic atypia.  Inflammatory infiltrate: Dense inflammatory cell infiltrate in the subepithelial connective tissue, especially around blood vessels. This is characteristic of a chronic inflammatory process.  Granulation tissue: Presence of newly formed capillaries and fibroblasts beneath the ulcerated epithelium is consistent with healing ulcer and granulation tissue formation.  2. High Magnification (Second Image):  Fibrinopurulent exudate: The ulcer bed contains necrotic debris, fibrin, and numerous polymorphonuclear leukocytes (neutrophils), consistent with a fibrinopurulent membrane.  Dilated capillaries and hemorrhage: Evidence of vascular proliferation and red blood cells extravasated into the tissue, which are features of granulation tissue.  Mixed inflammatory infiltrate: Involvement of neutrophils (acute) and chronic inflammatory cells (lymphocytes, plasma cells) confirms a mixed acute-on-chronic inflammatory reaction.  No dysplasia or malignancy: Despite the reactive epithelial hyperplasia, there is no evidence of cellular atypia or invasion suggestive of malignancy.  Conclusion:  These findings are classic for a chronic ulcer with secondary inflammation and granulation tissue. This can occur in various settings such as trauma, infection, or systemic diseases involving the oral mucosa (e.g., aphthous ulcers, traumatic ulcers, or ulcerated lichen planus). However, no specific granulomatous or infectious organisms are noted, ruling out specific infections like tuberculosis or fungal disease. | Histopathological diagnosis (single best):  Mucous-membrane pemphigoid (cicatricial pemphigoid) – a chronic, immune-mediated subepithelial blistering disease that commonly presents as persistent oral ulcers/erosions.  Microscopic hallmarks visible in the two H-E sections  Field Key observations Diagnostic significance  Low-power overview • Wide, empty cleft separating the entire thickness of the surface stratified squamous epithelium from the underlying lamina propria.  • Intact epithelial “roof” is hyper-parakeratotic and slightly papillated.  • Lamina propria shows patchy chronic inflammation. A subepithelial (sub-basement-membrane) split is the signature feature of pemphigoid‐group disorders and rules out suprabasal blistering entities such as pemphigus vulgaris.  High-power detail of the cleft • Cleft cavity contains fibrin, necrotic debris and numerous extravasated erythrocytes (“blood-filled blister”).  • Basal keratinocytes remain attached to the lifted epithelium; no acantholysis, no “row-of-tombstones”. Confirms mechanical separation at the basement-membrane zone without inter-cellular dissolution, supporting an auto-antibody attack on BMZ components (BP180, BP230, etc.).  Inflammatory milieu • Mixed inflammatory infiltrate in lamina propria rich in lymphocytes, plasma cells, neutrophils and scattered eosinophils surrounding the cleft and vascular channels. The chronic, mixed inflammatory background is typical of mucous-membrane pemphigoid and helps distinguish it from purely neutrophilic bullous diseases (e.g., linear-IgA).  Surface epithelium away from the bulla • Hyperplasia/elongation of rete ridges but orderly maturation and absent dysplasia.  • Focal neutrophilic micro-abscesses in superficial epithelium. Reactive epithelial hyperplasia secondary to chronic ulceration; lack of keratinocyte necrosis or saw-tooth rete rules out erosive lichen planus.  These combined features – especially the clean subepithelial split filled with blood/fibrin and the absence of acantholysis – pin the lesion down to mucous-membrane pemphigoid, the prototypical chronic ulcerative/blistering condition in the oral cavity. |
| 7.15 | **Diagnosis:** Foreign body gingivitis; **Key Microscopic Features:** The oral mucosa demonstrates mild chronic inflammation, and granules of foreign material—some with a slightly crystalline appearance—are present within the tissue. | Based on the hematoxylin and eosin (H&E) stained histological images you provided—one at low magnification and the other at high magnification—this lesion fits best with the diagnosis of:  Single Most Likely Histopathological Diagnosis:  Traumatic Ulcerative Granuloma with Stromal Eosinophilia (TUGSE)  Key Microscopic Features Justifying the Diagnosis:  Low Magnification (Image 1):  Ulcerated surface epithelium: The mucosal epithelium is lost in the central area, consistent with ulceration.  Underlying inflammatory infiltrate: A dense and deep inflammatory infiltrate extends into the submucosa and underlying muscle. This infiltrate disrupts the normal architecture.  Pseudoepitheliomatous hyperplasia (PEH): Adjacent epithelium shows irregular proliferation, resembling squamous cell carcinoma but benign in nature.  High Magnification (Image 2):  Eosinophil-rich inflammatory infiltrate: Numerous eosinophils are present among the inflammatory cells, which is a hallmark of TUGSE.  Atypical large mononuclear cells: Scattered, large, atypical-appearing mononuclear cells are seen, often misinterpreted as malignant but they are reactive histiocytes or T-cells.  Granulation tissue and myofibroblasts: Background stroma is composed of fibroblasts and myofibroblasts with granulation tissue characteristics, supporting a reactive process.  Disruption of muscle fibers: The infiltrate penetrates and disrupts the muscle fibers, a classic feature of TUGSE.  Summary:  TUGSE is a benign, self-limiting lesion, often presenting as a non-healing ulcer on the tongue or other oral mucosal surfaces. Its alarming histological appearance with pseudoepitheliomatous hyperplasia, eosinophilic infiltrate, and large mononuclear cells can mimic malignancy, but the deep tissue infiltration by eosinophils and the absence of true neoplastic features help distinguish it. | Histopathological diagnosis  Pyogenic granuloma (lobular capillary haemangioma) – a reactive inflammatory/vascular lesion that often presents clinically as an ulcerated, easily-bleeding mass on the oral mucosa.  Key microscopic features that point to this diagnosis  Field What is seen in the photomicrographs Diagnostic significance  Low-power architecture A broad, dome-shaped submucosal mass composed of exuberant pink granulation tissue that pushes up beneath the stratified squamous epithelium. The surface epithelium is thinned and focally ulcerated. A prototypical appearance of a reactive vascular proliferation that develops after local trauma or chronic irritation.  Lobular organisation The fibro-vascular tissue is subtly divided into small lobules by thin fibrous septa. The lobular (“capillary-lobular”) pattern is the hallmark feature that separates pyogenic granuloma from other inflammatory hyperplasias.  Profuse capillary proliferation Numerous, tightly packed capillary-sized vessels lined by plump endothelial cells; many lumina are slit-like or collapsed at this magnification. Capillary overgrowth is the defining element of a lobular capillary haemangioma.  Extravasated erythrocytes & haemorrhage Scattered foci of free red cells and golden-brown hemosiderin pigment (best appreciated on the high-power image). Reflects the extreme vascularity and clinical tendency of the lesion to bleed.  Mixed inflammatory infiltrate Neutrophils, lymphocytes and macrophages are peppered through the stroma, particularly under the ulcerated surface. Confirms the reactive, “pyogenic” (pus-forming/inflammatory) nature rather than a true neoplasm.  Spindle-shaped fibroblasts & immature collagen Plump fibroblasts embedded in loose, edematous connective tissue. Represents the granulation-tissue response that accompanies the vascular proliferation.  Taken together, the lobular arrangement of proliferating capillaries, the haemorrhagic/ulcerated surface, and the accompanying mixed inflammation are diagnostic of pyogenic granuloma in the setting of oral ulcerative-inflammatory lesions. |
| 7.16 | **Diagnosis:** Foreign body gingivitis; **Key Microscopic Features:** Non-necrotizing granulomas occupy the lamina propria, and foreign material is identified inside the cytoplasm of giant cells. | Based on the provided hematoxylin and eosin (H&E) stained histopathological images of an oral lesion (low and high magnification), the most likely diagnosis falls under ulcerative and inflammatory conditions of the oral cavity.  Most Likely Histopathological Diagnosis:  Granulomatous inflammation (consistent with oral tuberculosis)  Key Microscopic Features Observed:  Granuloma Formation:  Both images reveal granulomatous inflammation, characterized by aggregates of epithelioid histiocytes.  Presence of Langhans-type multinucleated giant cells, a hallmark of granulomatous diseases such as tuberculosis.  Lymphoplasmacytic Infiltrate:  Dense infiltrates of lymphocytes and plasma cells surround the granulomas, indicating chronic inflammation.  Caseation Necrosis (suggested):  The central area in some granulomas appears more eosinophilic and structureless, suggestive of caseation necrosis, typical of tuberculous granulomas.  Fibrosis:  There is evident fibroblastic proliferation in the background, a response to chronic inflammation.  Vascular Changes:  Mild vascular proliferation and dilation may be noted, contributing to the inflammatory milieu.  Summary:  The histopathological features are most consistent with oral tuberculosis, a chronic granulomatous condition often presenting with ulceration and indurated lesions. The presence of granulomas with epithelioid histiocytes, Langhans giant cells, and possible caseation necrosis strongly supports this diagnosis.  For definitive diagnosis, Ziehl-Neelsen staining for acid-fast bacilli (AFB) and clinical correlation (history of pulmonary TB, systemic symptoms) are essential. | Histopathological diagnosis  → Necrotising sialometaplasia (infarct-type inflammatory ulcer of minor salivary glands)  Key microscopic features visible in the two H-E sections  # Feature (where to look) Diagnostic significance  1 Lobular outline of the salivary gland is still recognisable – pale pink fibrous septa separate roundish lobules (low-power image). Tells you the lesion is an infarct of an existing gland rather than an infiltrative tumour; the anatomic architecture is “frozen in place.”  2 Coagulative necrosis of mucous acini – ghost-like acinar cells with pale, vacuolated cytoplasm and faded/absent nuclei (high-power image centre). Classic early event in necrotising sialometaplasia caused by ischaemia.  3 Squamous metaplasia of salivary ducts – solid nests/cords of bland squamous-looking epithelium (purple island toward the top of the low-power field). Hallmark change that can mimic a carcinoma, but cells are cytologically bland and remain within lobules.  4 Dense mixed chronic inflammatory infiltrate – sheets of lymphocytes ± plasma cells filling the interstitium and rimming necrotic acini (both magnifications). Explains the ulcerative, painful clinical picture and places the lesion in the inflammatory/ulcerative category.  5 Early granulation tissue & fibrosis – plump spindle fibroblasts in a pink collagenous stroma between lobules (low-power image). Part of the reparative phase after the ischaemic event.  6 No significant cytologic atypia or infiltrative growth – nuclei remain small/regular, stromal invasion is absent. Helps exclude squamous-cell carcinoma or mucoepidermoid carcinoma, which necrotising sialometaplasia can clinically and histologically mimic.  Together, the preserved lobular architecture, infarct-type necrosis of acini, ductal squamous metaplasia and heavy inflammatory/repair reaction are diagnostic of necrotising sialometaplasia. |
| 7.18 | **Diagnosis:** Pyostomatitis vegetans; **Key Microscopic Features:** Marked acanthosis with suprabasal cleft formation, with eosinophils found both within the clefts and in the lamina propria, and acantholytic cells accompanied by eosinophils are present within those clefts. | Based on the histopathological features observed in the provided hematoxylin-and-eosin (H&E) stained images at different magnifications, the single most likely diagnosis is:  Herpes Simplex Virus (HSV) Infection  Key Microscopic Features Supporting This Diagnosis:  1. Ulceration of the Epithelium  The surface epithelium shows areas of ulceration and erosion, a hallmark of herpes virus infections during active phases.  2. Acantholysis and Ballooning Degeneration  Intraepithelial vesicle formation with acantholytic (separated) keratinocytes and ballooning degeneration (cells appear swollen and pale), especially visible in the higher magnification image.  3. Multinucleated Giant Cells with Viral Cytopathic Effect  In the high-power image, there are multinucleated keratinocytes exhibiting:  Nuclear molding (nuclei pressed against each other),  Margination of chromatin (peripheral chromatin clumping),  Ground-glass nuclear appearance, all classic signs of HSV cytopathic changes.  4. Dense Inflammatory Infiltrate  The subepithelial connective tissue shows a dense, mixed inflammatory infiltrate, predominantly lymphocytes and plasma cells, consistent with viral mucosal infection.  Summary:  The combination of ulceration, intraepithelial vesiculation, ballooning degeneration, and characteristic viral cytopathic changes (particularly multinucleated giant cells with molded nuclei and chromatin margination) strongly supports the diagnosis of Herpes Simplex Virus infection, commonly presenting as primary herpetic gingivostomatitis or recurrent intraoral herpes in the oral cavity. | Histopathological diagnosis (single best):  Oral lichen planus (lichenoid mucositis) – classic “lichenoid/interface” mucositis pattern.  Key microscopic features visible in the three H-E photomicrographs  # Feature seen How it supports lichen planus  1 Dense, continuous, band-like inflammatory infiltrate of small lymphocytes hugging the epithelium–connective-tissue junction (best appreciated in the low- and medium-power images). A sharply demarcated subepithelial T-cell band is the hallmark of a lichenoid/interface reaction.  2 Saw-tooth (pointed) rete ridges with irregular elongation and pseudo-epitheliomatous hyperplasia at the ulcer edge. The serrated rete-peg profile is typical for oral lichen planus and helps separate it from other ulcerative conditions.  3 Hydropic / liquefaction degeneration of the basal cell layer – basal keratinocytes appear swollen, vacuolated or lost. Interface damage to basal cells is characteristic of lichen planus.  4 Civatte (colloid, apoptotic) bodies – round eosinophilic bodies representing apoptotic keratinocytes within the basal layer or superficial lamina propria (highlighted in the high-power image). These apoptotic keratinocytes are a classic supportive finding.  5 Predominantly lymphocytic exocytosis into the lower epithelium with very few neutrophils or eosinophils. The monomorphous T-cell infiltrate (rather than a mixed/eosinophil-rich one) separates OLP from traumatic ulcerative granuloma, plasma-cell gingivitis, etc.  6 Overlying surface ulcer / fibrinous exudate at one margin. Explains the clinical “ulcerative/erosive” presentation while still retaining the lichenoid architecture around it.  Taken together, the interface band of lymphocytes, basal cell degeneration with Civatte bodies, and saw-tooth rete ridges firmly point to oral lichen planus rather than other ulcerative or inflammatory mucosal lesions. |

GRANULOMATOUS, IMMUNEMEDIATED, AND AUTOIMMUNE CONDITIONS

| **Fig_no** | **Ground truth** | **GPT 4o answer** | **o3 answer** |
| --- | --- | --- | --- |
| 8.2 | **Diagnosis:** Torn hair follicle; **Key Microscopic Features:** Dermal tissue exhibits granulomatous inflammation with abscess formation, and a hair fragment is encased by foreign body giant cells. | Based on the provided hematoxylin and eosin (H&E)-stained histological images of the same lesion at different magnifications, the most likely histopathological diagnosis is:  Sarcoidosis  Key Microscopic Features Justifying the Diagnosis:  Low Magnification Image (First Image):  Non-caseating granulomas: There are numerous well-formed granulomas composed of tightly clustered epithelioid histiocytes with minimal central necrosis (a hallmark feature of sarcoidosis).  Scattered multinucleated giant cells are present within granulomas.  A lymphocytic infiltrate surrounds the granulomas (termed "naked granulomas" when sparse, as is common in sarcoidosis).  Fibrosis is observed in some areas around the granulomas.  High Magnification Image (Second Image):  Clear view of epithelioid histiocytes and Langhans-type multinucleated giant cells.  Within a giant cell, there is a Schaumann body (a laminated calcified structure), which is characteristic of sarcoidosis (though not pathognomonic).  Absence of caseous necrosis, helping differentiate from infectious granulomatous diseases like tuberculosis.  Summary Justification:  The presence of non-caseating granulomas, multinucleated giant cells, and structures such as Schaumann bodies, with no evidence of necrosis or infectious organisms, strongly favors sarcoidosis. This pattern, especially in an oral and maxillofacial location, fits within the granulomatous, immune-mediated, and autoimmune disease category. | Most likely histopathological diagnosis  → Sarcoidosis involving oral mucosa / minor salivary gland tissue  Key microscopic features in the images that support this diagnosis  Feature What you see in the provided sections Diagnostic significance  Well-formed, non-caseating granulomas Multiple tight collections of epithelioid histiocytes with occasional multinucleated giant cells are scattered through the connective tissue stroma and around ductal / glandular structures (first, low-power image). No central necrosis is present. Classic hallmark of sarcoidosis; distinguishes it from necrotising granulomatous infections (e.g. TB)  “Naked” appearance Only a sparse cuff of lymphocytes surrounds many granulomas, so the granulomas appear relatively “clean” against the background (first image). Sarcoid granulomas typically have minimal peripheral inflammation  Intranuclear or intracytoplasmic inclusions in giant cells In the higher-power image, a giant cell contains a round, laminated, eosinophilic body with a darker central core—i.e. a Schaumann body (conchoidal/calcium–protein complex). Occasional faint stellate (asteroid-like) eosinophilic material can also be appreciated. Schaumann and asteroid bodies are characteristic (though not pathognomonic) inclusions found in sarcoidosis  Absence of organisms or foreign material No refractile exogenous material, fungal hyphae, or acid-fast bacilli are seen in the granulomas presented. Helps exclude foreign-body reactions or infectious granulomatous diseases  Periductal / periacinar distribution in minor salivary gland stroma Granulomas wrap around salivary ducts and acini (first image). Oral sarcoidosis often shows this pattern, correlating with parotid and minor-gland involvement seen clinically  Taken together, the presence of well-formed non-caseating “naked” granulomas containing Schaumann bodies, set in oral/ salivary gland connective tissue without necrosis or organisms, is most consistent with sarcoidosis. |
| 8.3 | **Diagnosis:** Gelfoam-induced granuloma; **Key Microscopic Features:** Basophilic, irregularly shaped Gelfoam fragments are associated with fibrosis and chronic inflammatory infiltrate, surrounded by multinucleated foreign body giant cells and macrophages. | Key microscopic features  seen in the images that justify the diagnosis  ChatGPT đã nói:  Histopathological Diagnosis:  Sarcoidosis  Key Microscopic Features Supporting the Diagnosis:  Both images (low- and high-power hematoxylin-and-eosin stained sections) reveal classic features that are characteristic of sarcoidosis, a granulomatous inflammatory condition. Here's a breakdown of the findings:  Low-Power Image (First Image):  Diffuse granulomatous inflammation: The tissue shows widespread distribution of granulomas without significant necrosis.  Well-formed granulomas: These appear as pale pink nodules or collections in a background of fibrous connective tissue.  Minimal surrounding inflammation: The granulomas are "naked," meaning they have sparse lymphocytic cuffs, which is a hallmark of sarcoidosis.  High-Power Image (Second Image):  Epithelioid histiocytes: These are elongated, pale eosinophilic cells with vesicular nuclei clustered together, forming granulomas.  Multinucleated giant cells: Langhans-type or foreign-body-type giant cells are present, commonly found in granulomatous inflammation.  Asteroid bodies and Schaumann bodies (suggested, although not definitively seen here): These are characteristic but not pathognomonic for sarcoidosis and may be better visualized with special stains or deeper sectioning.  Absence of caseation necrosis: Helps distinguish sarcoidosis from infectious granulomatous diseases like tuberculosis.  Summary:  The non-caseating, well-formed granulomas with sparse peripheral lymphocytes, abundant epithelioid histiocytes, and presence of multinucleated giant cells are all highly consistent with sarcoidosis, a key entity in the granulomatous, immune-mediated, and autoimmune conditions affecting the oral and maxillofacial region. | Histopathological diagnosis (single best answer)  Granulomatosis with polyangiitis (Wegener granulomatosis) – necrotising, palisaded granulomatous inflammation with vasculitis  Key microscopic features in the two H-E sections that support this diagnosis  Observed feature Diagnostic significance  Large, irregular, “geographic” areas of basophilic necrosis with sharp, angular borders (“dirty” necrosis full of karyorrhectic debris) Characteristic of necrotising granulomas in GPA and helps separate it from the non-necrotising granulomas of sarcoidosis, Crohn disease, orofacial granulomatosis, etc.  Palisading rim of epithelioid histiocytes / spindle macrophages and scattered multinucleated giant cells concentrically surrounding the necrotic foci The palisaded arrangement is a hallmark of GPA, producing the typical necrotising, palisaded granuloma.  Prominent necrotising vasculitis of small–medium calibre vessels: vessel walls show fibrinoid change and are cuffed or infiltrated by neutrophils and mononuclear cells Necrotising vasculitis in and around the granulomas is obligatory for the diagnosis of GPA and distinguishes it from simple foreign-body or cholesterol granulomas.  Mixed inflammatory infiltrate rich in neutrophils (often clustered at the edge of necrosis), lymphocytes, and plasma cells Mixed acute-on-chronic inflammation is typical; a purely lymphoplasmacytic infiltrate would favour IgG4-related sialadenitis.  Fibrotic, swirling stroma between necrotic foci rather than true glandular or epithelial structures Confirms the lesion is not a primary salivary gland process such as necrotising sialometaplasia (which would show metaplastic ducts and residual acini).  Absence of well-formed non-necrotising granulomas and of abundant eosinophils Helps rule out sarcoidosis, orofacial granulomatosis, and eosinophilic granulomatosis with polyangiitis (Churg-Strauss).  Taken together, the combination of geographic necrosis, palisaded granulomatous inflammation, and necrotising vasculitis in an oral/maxillofacial specimen is diagnostic of granulomatosis with polyangiitis. |
| 8.4 | **Diagnosis:** Poly-L-lactic acid–associated granuloma; **Key Microscopic Features:** A well-circumscribed nodule comprised of multinucleated giant cells and spindle-shaped clefts, with giant cells displaying a bubbly cytoplasm, fusiform clefts, and asteroid bodies. | Based on the provided hematoxylin and eosin (H&E) stained histopathological images at low and high magnifications, from a lesion in the granulomatous, immune-mediated, and autoimmune conditions group of oral and maxillofacial pathology:  Single Most Likely Histopathological Diagnosis:  Sarcoidosis  Key Microscopic Features Justifying the Diagnosis:  Low-Magnification Image:  Well-circumscribed, non-caseating granulomas: Numerous granulomas distributed throughout the lesion with little or no central necrosis.  Uniform architecture: The granulomas appear relatively uniform and compact, typically seen in sarcoidosis.  High-Magnification Image:  Epithelioid histiocytes: Aggregates of activated macrophages (epithelioid cells) with pale, eosinophilic cytoplasm and elongated nuclei.  Multinucleated giant cells: Presence of Langhans-type or foreign body-type giant cells, common in granulomatous diseases.  Asteroid and Schaumann bodies (not always seen, but sometimes visible in sarcoidosis): Within the cytoplasm of giant cells, these inclusions can be supportive if present.  Absence of caseous necrosis: Important distinguishing feature from infections like tuberculosis.  Sparse lymphocytic infiltrate: Compared to other granulomatous diseases, sarcoidosis shows minimal peripheral lymphocytic response.  Additional Notes for Differential Diagnosis:  Sarcoidosis vs. Tuberculosis: Tuberculosis typically shows caseating necrosis and may have acid-fast bacilli.  Sarcoidosis vs. Crohn’s Disease or Orofacial Granulomatosis: These may have less well-formed granulomas and more prominent lymphocytic inflammation. | Single most likely histopathological diagnosis  → Cholesterol (xantho-)granuloma  Key microscopic features visible in the two H&E sections  Magnification What you can see How it supports the diagnosis  Low-power (whole-nodule view) • A well-circumscribed, roughly ovoid nodule surrounded by a fibrous capsule.  • The entire interior is peppered with innumerable slit-like, needle-shaped empty spaces (processing artefacts left by dissolved crystals).  • A fibro-vascular/xanthomatous stroma with scattered chronic inflammatory cells. The mass-forming growth with a “Swiss-cheese” distribution of sharp clefts is classic for a cholesterol crystal–rich granuloma.  High-power • Clear, elongated or rhomboid cholesterol clefts — some arranged radially (“star-burst”) or in parallel bundles.  • Surrounding each cleft are foreign-body–type multinucleated giant cells and foamy (xanthomatous) macrophages laden with lipid vacuoles.  • Intermixed plasma cells, lymphocytes and occasional red-cell debris/hemosiderin. Cholesterol clefts with a foreign-body giant-cell response and abundant lipid-laden macrophages are diagnostic of a cholesterol granuloma (xanthogranulomatous inflammation).  Why other granulomatous/auto-immune entities are less likely  Sarcoidosis – shows discrete, “naked” epithelioid granulomas without crystal clefts or xanthoma cells.  Orofacial granulomatosis / Crohn disease – granulomas are sparse, poorly circumscribed and lack the profuse cholesterol-cleft pattern.  Foreign material (e.g., talc, beryllium) – clefts are absent or irregular, and refractile polarizable particles are seen instead.  Taken together, the striking profusion of cholesterol clefts accompanied by xanthomatous and giant-cell reaction definitively points to a cholesterol granuloma within the spectrum of granulomatous, immune-mediated conditions of the oral & maxillofacial region. |
| 8.5 | **Diagnosis:** Calcium hydroxyapatite filler–induced granuloma; **Key Microscopic Features:** Homogeneous mauve-gray spherical particulates are interspersed among muscle fibers with numerous multinucleated foreign body giant cells, and nonrefractile mauve-gray round particles reside within these giant cells. | Histopathological Analysis:  Single Most Likely Diagnosis:  Sarcoidosis  Key Microscopic Features Supporting This Diagnosis:  Non-caseating Granulomas:  Both low- and high-power images show well-formed granulomas composed of epithelioid histiocytes without central necrosis, a hallmark of sarcoidosis.  These granulomas are non-caseating, differentiating them from infectious granulomatous conditions like tuberculosis (which typically show caseation necrosis).  Multinucleated Giant Cells:  Presence of Langhans-type multinucleated giant cells within granulomas, formed by the fusion of macrophages.  In some cases, these giant cells may contain inclusion bodies such as asteroid bodies or Schaumann bodies, which may be seen under higher magnification (though not distinctly visible in this set).  Sparse Lymphocytic Infiltrate:  A relatively sparse lymphocytic collar (peripheral lymphocytes) surrounds the granulomas, another characteristic feature of sarcoidosis, distinguishing it from other granulomatous diseases which may have more intense inflammation.  Absence of Necrosis or Organisms:  No evidence of necrosis (which argues against infectious etiologies like TB or fungal infections).  No visible organisms with H&E; special stains (e.g., Ziehl-Neelsen or PAS) would be negative in sarcoidosis.  Contextual Justification:  In the context of granulomatous, immune-mediated, and autoimmune conditions of the oral and maxillofacial region, sarcoidosis is a well-known systemic granulomatous disease that can involve salivary glands, lips, and oral mucosa. It can be part of Heerfordt’s syndrome (uveoparotid fever) when involving salivary glands. | Histopathological diagnosis  → “Hyaline-ring (pulse) granuloma” – a vegetable-matter/foreign-body granulomatous reaction  Key microscopic features visible in the two H-E fields that support this diagnosis  Feature How it looks in the submitted pictures Diagnostic significance  Circular/oval hyaline rings with central clear or pale cores (“Swiss-cheese” pattern) Numerous round to oval spaces scattered through the fibrous stroma; the periphery of each space is a thin, glassy, eosinophilic rim, while the centre is empty or contains slightly collapsed, pale material with faint radial striations. These rings correspond to degenerating plant cell walls (cellulose); they are the classic hallmark of pulse granuloma.  Radial / lamellar striations inside some rings Several of the pale bodies show delicate, parallel bands or a crumpled-wall appearance. Represents collapsed vegetable matter and helps distinguish the lesion from mucin pools, fat, or cholesterol clefts.  Foreign-body–type granulomatous response Chronic inflammatory infiltrate rich in histiocytes and scattered multinucleated giant cells hugging the hyaline rings (visible especially around many of the spaces). Granulomatous reaction to indigestible exogenous material is the expected host response in pulse granuloma.  Background fibro-collagenous connective tissue Pink, moderately dense collagen between the rings; overlying mucosa is ulcerated with fibrin and extravasated blood at the surface in the low-power view. Lesion typically occurs in healing extraction sockets or traumatised mucosa where vegetable matter becomes implanted.  Absence of well-formed sarcoid-type granulomas, caseation, or vasculitis No tight epithelioid granulomas, necrotic centres, or vessel wall destruction are seen. Helps exclude other oral granulomatous diseases such as sarcoidosis, tuberculosis, GPA, etc.  Together, the combination of numerous hyaline rings produced by degenerated vegetable particles and the accompanying foreign-body granulomatous infiltrate is diagnostic of a hyaline-ring (pulse) granuloma in the oral cavity. |
| 8.6 | **Diagnosis:** Silicone-induced granuloma; **Key Microscopic Features:** The lamina propria contains numerous vacuolated cells, macrophages feature vacuoles of varying sizes, and some macrophages exhibit a vesicular, bubbly cytoplasm. | Histopathological Analysis of the Provided Images  Single Most Likely Histopathological Diagnosis:  Orofacial Granulomatosis (OFG)  Justification with Key Microscopic Features:  Across the provided hematoxylin and eosin (H&E) stained sections at varying magnifications, the histologic hallmarks are consistent with orofacial granulomatosis, a chronic granulomatous condition.  1. Non-caseating Granulomas:  Multiple non-caseating granulomas are visible, composed of clusters of epithelioid histiocytes (macrophage-derived cells with a pink, granular cytoplasm).  Presence of multinucleated giant cells, some of which resemble Langhans-type, scattered within the granulomas (particularly in the higher magnification images).  2. Perivascular Lymphocytic Infiltrate:  Mild to moderate lymphocytic infiltrate surrounding blood vessels in the lamina propria and deeper connective tissue.  3. Edema and Vascular Proliferation:  There is interstitial edema in the connective tissue and an increase in small capillary-like blood vessels, contributing to a loose myxoid appearance in some areas.  4. Absence of Caseation Necrosis:  No evidence of central necrosis, helping to differentiate this from infectious granulomatous diseases such as tuberculosis.  5. Overlying Epithelium:  The surface stratified squamous epithelium appears mildly hyperplastic with some acanthosis, but shows no dysplasia or ulceration.  Differential Considerations and Exclusion:  Sarcoidosis: Also presents with non-caseating granulomas, but often shows more compact granulomas and is typically systemic. No asteroid or Schaumann bodies were noted.  Crohn's Disease: May have similar features but typically presents with GI symptoms and transmural inflammation not evident here.  Infectious Granulomas (e.g., TB, deep fungal infections): Ruled out due to the absence of caseation necrosis and lack of identifiable organisms.  Foreign Body Granuloma: No birefringent material or obvious foreign substance identified.  Conclusion:  The constellation of non-caseating granulomas, perivascular lymphocytic infiltrate, and absence of infection or necrosis points toward orofacial granulomatosis as the most probable histopathological diagnosis. Further clinical correlation (e.g., lip swelling, facial edema) would reinforce this diagnosis. | Histopathological diagnosis  Verruciform xanthoma of the oral mucosa.  Key microscopic features that support this diagnosis  Field What is seen in the provided slides Why it points to verruciform xanthoma  Surface epithelium Stratified‑squamous mucosa with mild acanthosis and slightly undulating (verrucous/papillary) rete ridges. The epithelial bases sit directly on a band of foamy cells. VX is an epithelial‑associated lesion; the rete ridges are usually uniform and project between collections of xanthoma cells.  Subepithelial band of cells Dense aggregates of foamy (xanthoma) histiocytes filling the connective‑tissue papillae and upper lamina propria. The cells have abundant vacuolated (“bubbly”) cytoplasm and small, centrally placed, dark nuclei. The continuous sheet of lipid‑laden macrophages immediately beneath the epithelium is the defining feature of VX.  Inflammation / granulomas No true epithelioid granulomas, necrosis or multinucleated giant cells are present—only the foamy macrophages. A sparse chronic inflammatory infiltrate is scattered among them. Helps distinguish VX from other granulomatous disorders (sarcoidosis, Crohn’s, orofacial granulomatosis) that produce well‑formed granulomas.  Vascular & stromal changes Capillaries and small venules traverse the foamy macrophage layer; deeper connective tissue is relatively unremarkable fibrous tissue. Common reactive vascularity seen in VX; absence of salivary‑gland necrosis or vasculitis argues against other entities.  Absence of dysplasia Overlying epithelium shows no cytologic atypia. VX is a benign, reactive process.  Taken together, the subepithelial sheet of lipid‑laden (xanthoma) macrophages immediately beneath a mildly verrucous squamous epithelium is pathognomonic for verruciform xanthoma and accounts for all features seen at both low and high power in the supplied images. |
| 8.8 | **Diagnosis:** Hyaluronic acid filler–induced granuloma; **Key Microscopic Features:** Adipose tissue encloses pools of inert, amorphous basophilic material, with mild fibrosis present but without any foreign body reaction or surrounding inflammation. | Histopathological Analysis of Provided H&E Images  Category: Granulomatous, Immune-Mediated, and Autoimmune Conditions  Magnification: Low and High Power Views Provided  🧠 Single Most Likely Histopathological Diagnosis:  Sarcoidosis  🔬 Key Microscopic Features Justifying the Diagnosis:  Non-caseating Granulomas:  The hallmark of sarcoidosis seen in both images.  Composed of tight aggregates of epithelioid histiocytes without central necrosis (i.e., non-caseating).  In high-power view, these granulomas show relatively few surrounding lymphocytes, known as "naked granulomas", a classic feature of sarcoidosis.  Multinucleated Giant Cells:  Present within granulomas, likely of Langhans or foreign body type.  These giant cells are formed by fusion of macrophages and are typical of granulomatous inflammation.  Absence of Caseous Necrosis:  Distinguishes sarcoidosis from infectious granulomatous diseases such as tuberculosis.  No areas of central necrosis are seen, which would otherwise suggest caseation.  Fibrosis and Vascular Involvement (secondary findings):  There appears to be some collagen deposition/fibrosis around granulomas.  Occasional blood vessels are noted within the granulomatous background, though not prominently involved.  📌 Summary:  These classic findings—well-formed, non-caseating “naked” granulomas, multinucleated giant cells, and lack of necrosis or organisms—are diagnostic of sarcoidosis, a systemic granulomatous disease that can present in the oral and maxillofacial region.  If you have any clinical data (e.g., chest X-ray, serum ACE levels, or patient symptoms), this can further support the diagnosis. Let me know if you'd like a differential diagnosis table or comparison with other granulomatous conditions (like Crohn’s disease, Wegener's, TB, etc.). | Histopathological diagnosis (single best):  Granulomatosis with polyangiitis (Wegener granulomatosis) – necrotising granulomatous inflammation of the upper aerodigestive tract.  Key microscopic features seen in the two H-E photomicrographs  Feature How it looks in the provided slides Significance  1. “Geographic” coagulative necrosis Large, irregular, map-shaped expanses of pale-lilac/grey acellular material with angular “cracked-ice” fissures dominate the field (best appreciated in the low-power image). Characteristic of GPA – produced by massive neutrophil karyorrhexis (“dirty” necrosis).  2. Peripheral palisading reaction / early granuloma formation At the interfaces between the necrotic foci and viable tissue there is a narrow rim of pink fibro-vascular tissue with scattered spindle cells and inflammatory cells; in other foci multinucleated histiocytic giant cells can be picked out at higher power. Granulomatous response that attempts to wall-off the necrotic zones.  3. Necrotising vasculitis of small vessels Numerous small calibre vessels traversing the lesion show concentric pink fibrinoid change, mural disruption and perivascular inflammatory cuffs (clearest in the high-power view). Hallmark of GPA and the key feature that separates it from simple foreign-body granulomas.  4. Mixed chronic-active inflammatory infiltrate Around the necrotic islands and within the fibrous stroma are neutrophils (nuclear dust), lymphocytes and plasma cells. Reflects the immune-mediated nature of the process and drives tissue destruction.  5. Preservation of adjacent tissue architecture Intact skeletal muscle fibres and islands of adipose tissue are present at the periphery, abruptly ending at the necrotic “geographic” margins. Shows the destructive, yet patchy, nature of GPA lesions.  These combined findings -- large “map-like” necrosis, a palisading granulomatous rim, and fibrinoid vasculitis – are diagnostic of granulomatosis with polyangiitis in an oral–maxillofacial biopsy. |
| 8.10 | **Diagnosis:** Orofacial granulomatous disease; **Key Microscopic Features:** The lamina propria is edematous with a faint granuloma and dilated vasculature, and a subtle nonnecrotizing granuloma envelops a blood vessel. | Based on the provided hematoxylin-and-eosin (H&E) stained histological images—one at low magnification and the other at higher magnification—this lesion falls within the granulomatous, immune-mediated, and autoimmune category of oral and maxillofacial pathology.  🧠 Single Most Likely Histopathological Diagnosis:  Orofacial granulomatosis (OFG)  🔬 Key Microscopic Features Supporting the Diagnosis:  1. Granulomatous Inflammation:  In the high-power image, there is a clear non-caseating granuloma composed of epithelioid histiocytes, surrounded by a lymphocytic infiltrate.  Occasional multinucleated giant cells may be present (though not distinctly visible in this view, they are often associated with OFG).  2. Perivascular Lymphocytic Infiltrates:  The lower power image shows dense chronic inflammatory infiltrate, mainly lymphocytes, extending into the superficial lamina propria, often in a perivascular distribution.  3. Acanthosis and Pseudoepitheliomatous Hyperplasia:  The overlying stratified squamous epithelium shows acanthosis (thickened spinous layer), which is common in OFG due to chronic irritation and inflammation.  No epithelial dysplasia is evident.  4. Absence of Caseation Necrosis:  This rules out infectious granulomatous diseases such as tuberculosis.  🧾 Differential Diagnoses (excluded based on features):  Sarcoidosis: Also non-caseating granulomas, but often deeper, more compact, and with minimal inflammation. OFG is more likely when oral involvement is predominant.  Crohn’s disease: OFG may be a manifestation; clinical correlation is essential.  Foreign body granulomas: Often centered around exogenous material—none identified here.  Wegener's granulomatosis (GPA): Typically necrotizing granulomas with vasculitis—not seen in this image.  🧠 Final Comment:  Orofacial granulomatosis is a diagnosis of exclusion and often part of a clinical syndrome (e.g., Melkersson-Rosenthal syndrome or Crohn's disease). Histopathologically, it is best recognized by its characteristic non-caseating granulomas with a chronic inflammatory infiltrate in the oral mucosa or submucosa. | Most likely histopathological diagnosis  → Sarcoidosis (non-caseating “naked” granulomatous inflammation of the oral mucosa)  Key microscopic features visible in the two H-E sections  Field What is seen Why it points to sarcoidosis  Low-power view (first image) • Intact, acanthotic stratified squamous epithelium.  • Multiple, well-circumscribed round/oval collections in the superficial lamina propria.  • Collections sit independently in otherwise fibrous stroma and are only thinly rimmed by lymphocytes (“naked” appearance). Discrete, superficially placed, non-necrotising granulomas with scant peripheral inflammation are characteristic of sarcoidosis and uncommon in most other oral granulomatous diseases.  High-power view (second image) • A compact focus of pale, epithelioid histiocytes with indistinct borders and oval, vesicular nuclei.  • Sparse cuff of small lymphocytes at the margin.  • No central caseation, fibrinoid necrosis or vasculitis.  • Scattered collagen bundles of the lamina propria surrounding the granuloma. The combination of well-formed epithelioid cell granuloma, absence of necrosis, and minimal lymphocytic collar is the classic “naked” non-caseating granuloma of sarcoidosis. Lack of vasculitic changes or central necrosis helps exclude Wegener/GPA or infectious granulomas.  Summary of the defining picture: multiple superficial, non-caseating, minimally inflamed granulomas (“naked” granulomas) in otherwise normal oral mucosa is virtually pathognomonic for sarcoidosis in the setting of maxillofacial pathology. |
| 8.11 | **Diagnosis:** Crohn’s disease; **Key Microscopic Features:** Numerous nonnecrotizing granulomas are found within the minor salivary glands of the lip, each composed of multinucleated giant cells and lymphocytes. | Histopathological Diagnosis:  Sarcoidosis  Key Microscopic Features Justifying the Diagnosis:  Low Magnification (First Image):  Well-formed granulomas scattered throughout the connective tissue stroma, without central necrosis.  Granulomas are non-caseating and contain clusters of epithelioid histiocytes.  There is a background of dense lymphocytic infiltrate.  Surrounding the granulomas are numerous small ducts and salivary gland acini, indicating the lesion is within or adjacent to a salivary gland (commonly affected site in oral sarcoidosis).  High Magnification (Second Image):  Presence of epithelioid histiocytes with pale, pink cytoplasm forming the granuloma.  A multinucleated giant cell is present centrally within the granuloma.  Absence of caseous necrosis, a key feature differentiating sarcoidosis from infectious granulomatous diseases like tuberculosis.  The surrounding inflammatory infiltrate is composed primarily of lymphocytes, supporting a chronic immune-mediated response.  Additional Notes:  Sarcoidosis is a multisystem granulomatous disorder of unknown etiology that frequently affects the lungs, lymph nodes, and in some cases, the oral cavity, especially minor salivary glands.  Histologically, non-caseating granulomas are the hallmark of sarcoidosis.  It is important to rule out infectious causes (e.g., fungal, mycobacterial) via special stains or clinical correlation. | Histopathological diagnosis (single best):  Non-caseating granulomatous inflammation consistent with sarcoidosis involving minor salivary gland tissue.  Key microscopic features in the two levels that support this diagnosis  Field Observations Significance  Low-power view • Numerous, discrete to confluent purple nodules scattered through the lamina propria and periductal stroma.  • Nodules replace and separate salivary gland acini and ducts. Indicates multiple well-formed granulomas centred in salivary tissue, a common site for oral sarcoidosis.  Granuloma architecture • Each nodule is composed of tightly packed epithelioid histiocytes forming solid, oval “balls”.  • No central caseous necrosis or suppuration. “Naked”, non-caseating granulomas are characteristic of sarcoidosis (rules out infectious caseating diseases).  High-power view • Epithelioid histiocytes with pale, elongated nuclei and indistinct cytoplasmic borders.  • Multinucleated giant cells (Langhans/foreign-body type) within the granuloma.  • Sparse rim of small lymphocytes (“naked” appearance). Classic cellular make-up of sarcoid granulomas.  Special inclusions • Within one giant cell a collection of laminated eosinophilic material suggestive of Schaumann/asteroid body (seen in the inset). Schaumann or asteroid bodies, although not pathognomonic, are frequently encountered in sarcoidosis.  Background stroma • Relatively little plasma-cell infiltrate or vasculitis.  • No fibrinoid necrosis of vessel walls. Helps exclude Wegener (GPA) or IgG-4 disease, which show prominent vasculitis or storiform fibrosis.  Together, the presence of multiple non-caseating, well-formed granulomas with giant cells and minimal peripheral inflammation, centred in minor salivary gland tissue, is diagnostic of sarcoidosis in the oral and maxillofacial region. |
| 8.12 | **Diagnosis:** Crohn’s disease; **Key Microscopic Features:** The lamina propria shows diffuse granulomatous inflammation with overlying mucosal ulceration, and the granulomas contain an abundance of eosinophils. | Based on the two provided hematoxylin and eosin (H&E) stained images at different magnifications, the most likely histopathological diagnosis is:  Sarcoidosis  Key Microscopic Features Justifying the Diagnosis:  Non-caseating Granulomas:  Seen clearly in both images, especially in the higher magnification.  Granulomas are compact collections of epithelioid histiocytes, often surrounded by lymphocytes.  Lack of central necrosis (i.e., non-caseating), which helps differentiate sarcoidosis from infections like tuberculosis.  Multinucleated Giant Cells:  Present within the granulomas; they are often Langhans-type or foreign body-type multinucleated giant cells.  These are hallmark components of granulomatous inflammation.  Minimal or Absent Necrosis:  Absence of caseation is crucial in distinguishing sarcoidosis from granulomatous infections.  Perivascular Lymphocytic Infiltrate (at lower magnification):  Suggests a systemic inflammatory process typical of sarcoidosis.  Differential Diagnoses (less likely due to microscopic clues):  Tuberculosis: Usually shows caseating necrosis (not seen here).  Granulomatosis with polyangiitis: Often has necrotizing granulomas with vasculitis, which is not apparent here.  Fungal Infections: Special stains would be needed, but no organisms are seen in these H&E images.  If this histologic pattern is from an oral or maxillofacial lesion, systemic evaluation for sarcoidosis (including chest imaging, serum ACE levels, etc.) would be warranted. | Histopathological diagnosis (single best):  Non-caseating granulomatous inflammation compatible with sarcoidosis involving the oral mucosa / minor salivary gland  Key microscopic features supporting this diagnosis  Field Findings Diagnostic significance  Low-power architecture Lamina propria and submucosa are replaced by multiple, discrete to coalescent nodules of granulomatous inflammation; surface epithelium is largely preserved or superficially ulcerated. Confluent but well-formed granulomas are typical of sarcoidosis and help distinguish it from the scattered granulomas of Crohn‐related orofacial granulomatosis.  Granuloma composition Tight aggregates of epithelioid histiocytes with pale, granular cytoplasm and elongated (“coffee-bean”) nuclei. Epithelioid histiocytes are the building blocks of immune (sarcoid-type) granulomas.  Giant cells Occasional Langhans- or foreign-body–type multinucleated giant cells are present; within them, round, eosinophilic, lamellated bodies are seen. Giant cells with Schaumann bodies (laminated calcified inclusions) and occasional asteroid bodies are classic, although not pathognomonic, for sarcoidosis.  Necrosis The centers of granulomas are viable; there is no caseation or suppurative necrosis. Absence of necrosis separates sarcoidosis from infectious (e.g., mycobacterial) granulomas and from the necrotizing granulomas of granulomatosis with polyangiitis.  Rim of lymphocytes Only a very sparse peripheral cuff of lymphocytes is present (“naked granulomas”). A minimal lymphoid collar is characteristic of sarcoid-type granulomas.  Background stroma / vessels No fibrinoid vasculitis, no heavy neutrophilic infiltrate, and no obvious organisms on routine H&E. Helps rule out ANCA-associated vasculitides and infectious etiologies.  Taken together, the presence of well-formed, non-caseating, “naked” granulomas with multinucleated giant cells containing Schaumann-type inclusions—and the absence of significant necrosis, vasculitis, or organisms—strongly points to sarcoidosis as the most likely diagnosis in the category of granulomatous, immune-mediated oral lesions. |
| 8.13 | **Diagnosis:** Sarcoid granulomatous disease; **Key Microscopic Features:** A minor salivary gland harbors nonnecrotizing “naked” granulomas composed of multinucleated giant cells. | Histopathological Analysis of H&E-Stained Sections  Group: Granulomatous, Immune-Mediated, and Autoimmune Conditions (Oral & Maxillofacial Pathology)  Magnifications: Low-power and high-power views  🧠 Single Most Likely Histopathological Diagnosis:  Sjögren Syndrome (Autoimmune Sialadenitis)  🔬 Key Microscopic Features Supporting the Diagnosis:  1. Focal Lymphocytic Sialadenitis  Dense periductal and perivascular lymphocytic infiltrates, particularly in the high-power image.  These infiltrates appear to be forming focal aggregates or foci, a hallmark feature in Sjögren syndrome.  2. Acinar Atrophy  There is prominent destruction and atrophy of serous acini in the glandular tissue.  Replaced by lymphocytes and fibrous stroma.  3. Ductal Changes  Mild to moderate ductal hyperplasia and possible epimyoepithelial islands (not clearly defined in this magnification but may be present on closer inspection).  Some ducts are dilated and surrounded by inflammatory cells.  4. Fibrosis and Fatty Replacement  Interspersed areas of stromal fibrosis and fatty replacement, common in chronic sialadenitis associated with Sjögren syndrome.  🧪 Diagnostic Criteria Correlation (Focus Score):  The observed lymphocytic foci in a 4 mm² area appear to meet or exceed the threshold focus score ≥1, which is a diagnostic criterion for Sjögren syndrome in minor salivary gland biopsies.  🩺 Conclusion:  The constellation of periductal lymphocytic aggregates, acinar atrophy, fibrosis, and ductal changes seen in both low- and high-power images is diagnostic of Sjögren Syndrome, a classic autoimmune condition in the oral and maxillofacial region. | Histopathological diagnosis  → Lympho-epithelial sialadenitis (benign lympho-epithelial lesion) – the characteristic salivary-gland manifestation of Sjögren syndrome.  Key microscopic features that point to this diagnosis  Microscopic finding (seen in the images) Diagnostic significance  Dense, patchy to diffuse lymphocytic infiltrates with germinal-centre formation – basophilic nodular aggregates occupy much of the parenchyma Auto-immune, immune-mediated process rather than an acute bacterial sialadenitis; typical of Sjögren-associated salivary gland disease  Marked acinar atrophy and loss – many lobules are replaced by lymphoid tissue; only scattered residual mucous/serous acini remain Explains xerostomia and is a prerequisite for calling it a lympho-epithelial lesion  Hyperplastic and metaplastic duct epithelium forming “epimyoepithelial islands” – small nests/cords of pale squamoid epithelium surrounded and infiltrated by lymphocytes (best appreciated in the high-power field) Pathognomonic for a benign lympho-epithelial lesion; distinguishes it from pure chronic sialadenitis or IgG4-related sialadenitis  Interlobular fibrosis and dilated ducts with inspissated secretions (eosinophilic luminal material) Chronicity and obstruction secondary to the autoimmune attack  Absence of true epithelioid-cell granulomas or caseation Helps rule out sarcoidosis, tuberculosis and other granulomatous disorders in the same differential group  Taken together, the combination of focal/patchy lymphoid infiltration with germinal centres, destruction of salivary acini, and the characteristic lympho-epithelial ductal islands is classical for lympho-epithelial sialadenitis/Sjögren syndrome in the oral–maxillofacial region. |
| 8.15 | **Diagnosis:** Benign migrating glossitis; **Key Microscopic Features:** There is an absence of filiform papillae, psoriasiform-like epithelial thickening, and chronic inflammatory infiltrate; marked spongiosis and inflammation involve nearly the full epithelial thickness with keratinocyte swelling and degeneration; and spongiotic pustules form in the absence of fungal organisms. | Histopathological Diagnosis:  Orofacial Granulomatosis (OFG)  Key Microscopic Features Supporting the Diagnosis:  Non-caseating Granulomas:  Seen prominently in the high-power field (third image).  Composed of epithelioid histiocytes and multinucleated giant cells.  Absence of central necrosis (which helps differentiate from tuberculosis or sarcoidosis).  Chronic Inflammatory Infiltrate:  Dense lymphoplasmacytic infiltrate is present throughout the lamina propria and submucosa (seen across all images).  Prominent perivascular arrangement in some areas.  Epithelial Hyperplasia:  The epithelium is hyperplastic with elongated rete ridges (clearly visible in the first and second images).  Edema and Vascular Changes:  Stromal edema with dilated blood vessels and inflammatory cell accumulation is evident, a hallmark of OFG.  Multinucleated Giant Cells:  Well-formed Langhans-type multinucleated giant cells can be observed (third image), which are part of granuloma formation.  Differential Diagnoses to Consider (but less likely based on features):  Sarcoidosis: Typically more systemic with well-formed granulomas but without significant epithelial hyperplasia or perivascular lymphoid cuffing.  Crohn’s Disease: Shares similar histology but requires gastrointestinal correlation.  Wegener’s Granulomatosis: Would show necrotizing granulomas and vasculitis, which are absent here.  Summary:  The combination of non-caseating granulomas, chronic inflammatory infiltrate, and epithelial hyperplasia within the oral mucosa supports a diagnosis of Orofacial Granulomatosis. Clinical correlation (e.g., lip or facial swelling, history of systemic symptoms) is recommended to confirm and guide management. | Histopathological diagnosis  → Oro-facial granulomatosis (non-caseating granulomatous mucositis)  Microscopic features supporting the diagnosis  Magnification Key findings Diagnostic relevance  Low-power (image 1) • Hyperplastic, mildly papillomatous stratified squamous epithelium.  • Diffuse, sheet-like expansion of the lamina propria by pale eosinophilic cells and mixed chronic inflammation without areas of necrosis. Indicates a mucosal process rather than a true “mass”; the broad, non-ulcerated surface rules out infectious ulcer/granuloma.  Intermediate (image 2) • Acanthosis with focal spongiosis of the epithelium.  • Heavy, band-like chronic inflammatory infiltrate extending from the subepithelial zone into deeper connective tissue.  • Early epithelioid cell clusters begin to be recognisable. The dense lymphoplasmacytic component and lack of caseation favour an immune-mediated, non-infective granulomatous condition.  High-power (image 3) • Numerous epithelioid histiocytes with oval, vesicular nuclei and ample pink cytoplasm forming poorly-formed, non-caseating granulomas.  • Scattered Langhans- and foreign-body-type multinucleated giant cells.  • Intermixed lymphocytes and plasma cells (“busy” or non-naked granuloma).  • No organisms, no fibrinoid necrosis, no vasculitic changes. Non-caseating granulomas with a prominent admixed chronic inflammatory infiltrate and absence of necrosis are the classic histologic hallmark of orofacial granulomatosis (and virtually identical to the oral lesions of Crohn disease). The granulomas are more ill-defined and inflammatory than the “naked” granulomas of sarcoidosis, helping to separate the two entities histologically.  Why this fits orofacial granulomatosis best  Oro-facial granulomatosis is, by definition, a non-caseating granulomatous reaction pattern of the oral mucosa in the absence of demonstrable infection; histology alone shows the reaction pattern, and in practice we render the diagnosis as “consistent with OFG (rule out Crohn’s disease, sarcoidosis clinically).”  The granulomas here are ill-defined and lymphocyte-rich, unlike the tight “naked” granulomas of sarcoidosis, and there is no caseation, polarizable material, or organisms that would suggest infection or foreign-body reaction.  This spectrum belongs to the “granulomatous, immune-mediated and autoimmune conditions” group of oral and maxillofacial lesions exactly as requested.  Hence, the single most appropriate histopathological label for the lesion in these images is orofacial granulomatosis / non-caseating granulomatous mucositis. |
| 8.23 | **Diagnosis:** Plasma cell–rich gingivitis; **Key Microscopic Features:** A heavy infiltrate of plasma cells occupies the lamina propria adjacent to the epithelium; the normal surface orthokeratin is eroded with spongiosis and leukocyte migration; a dense, polyclonal plasma cell population is observed; these findings are characteristic of plasma cell gingivitis. | Based on the provided hematoxylin and eosin (H&E) stained histopathological images of the same lesion at different magnifications, here is the analysis:  Single Most Likely Histopathological Diagnosis:  Orofacial Granulomatosis (OFG)  Key Microscopic Features Supporting the Diagnosis:  Low Magnification (1st Image):  Diffuse inflammatory infiltrate extending through the lamina propria and deeper connective tissue.  The infiltrate appears non-caseating and granulomatous.  Evidence of lymphatic and vascular involvement.  Intermediate Magnification (2nd Image):  Well-formed granulomas composed of collections of epithelioid histiocytes.  Perivascular and perilymphatic distribution of the granulomatous inflammation.  Presence of multinucleated giant cells interspersed within granulomas.  Overlying epithelium appears acanthotic with some basal layer disruption, indicating a chronic process.  High Magnification (3rd Image):  Epithelioid histiocytes with pale cytoplasm, clearly distinct from surrounding lymphocytes.  Numerous small lymphocytes, plasma cells, and scattered giant cells.  Absence of caseous necrosis, which helps rule out tuberculosis.  No evidence of fungal organisms or foreign material under routine staining.  Differential Diagnoses Considered and Ruled Out:  Sarcoidosis: Similar granulomas but usually systemic; oral lesions often show more organized granulomas without lymphocytic cuffing.  Crohn's disease: Can be indistinguishable histologically, but clinical GI symptoms help in systemic diagnosis.  Tuberculosis: Would show caseating granulomas and acid-fast bacilli.  Wegener’s granulomatosis (GPA): Usually associated with necrotizing granulomas and vasculitis, which are not evident here.  Summary:  The constellation of non-caseating granulomas, lymphoplasmacytic infiltrate, multinucleated giant cells, and the absence of necrosis is classic for Orofacial Granulomatosis (OFG). Clinical correlation (e.g., lip swelling, facial swelling, mucosal tags) is essential for confirmation. | Histopathological diagnosis  Rosai–Dorfman disease (sinus histiocytosis with massive lymphadenopathy) – extranodal oral lesion  Key microscopic features visible in the H-E slides  Feature Where you can see it Why it matters for the diagnosis  Diffuse sheet-like infiltrate of large histiocytes Low-power overview (first image) shows a solid, lobulated mass replacing the lamina propria/submucosa with plentiful thin-walled vessels running through it. RDD is a histiocytic proliferation that typically effaces normal architecture in this way.  Abundant pale/foamy eosinophilic cytoplasm & vesicular round nuclei with prominent nucleoli High-power fields (second & third images) – the larger cells have open chromatin and single, central nucleoli. These cytomorphologic features are classic for Rosai-Dorfman (S-100-positive) histiocytes.  Emperipolesis (intact lymphocytes and plasma cells within histiocyte cytoplasm) High-power image: several pale histiocytes contain sharply outlined dark lymphocyte nuclei in their cytoplasm. Emperipolesis is the hallmark of Rosai-Dorfman disease and virtually pathognomonic when present.  Mixed inflammatory background – many small mature lymphocytes & plasma cells Seen in all magnifications, especially the high-power view. RDD always shows an accompanying polytypic lymphoplasmacytic infiltrate.  No well-formed granulomas, caseation or vasculitis Entire section – although the category is “granulomatous / immune-mediated,” the lesion lacks discrete epithelioid granulomas or necrosis. Helps exclude sarcoidosis, Crohn disease, Wegener (GPA), etc.  Intact overlying stratified squamous epithelium Second image (top of the field). Confirms an underlying submucosal process rather than an epithelial neoplasm.  If immunohistochemistry were performed, the lesional histiocytes would be S-100+, OCT2+, CD68+, and CD1a-negative, supporting the morphologic impression of Rosai-Dorfman disease. |
| 8.25 | **Diagnosis:** Lichen planus; **Key Microscopic Features:** There is parakeratosis and thickening of the epithelium, degenerated basal keratinocytes, a thickened basement membrane, and a band of lymphocytes at the epithelial-connective tissue junction; additionally, spongiosis with lymphocyte migration, basal cell degeneration blurring the interface, and lymphocytes at the junction are evident. | Based on the provided hematoxylin and eosin (H&E) stained histological images of the same lesion at different magnifications, the most likely single histopathological diagnosis is:  Lichen Planus (Oral Lichen Planus - OLP)  Key Microscopic Features Supporting the Diagnosis:  Saw-tooth Appearance of Rete Ridges (Low Power View)  The rete ridges of the epithelium exhibit an irregular, “saw-tooth” or pointed morphology — a hallmark of lichen planus.  Dense, Band-like Lymphocytic Infiltrate (Low & High Power)  A dense, subepithelial, band-like infiltrate composed predominantly of T-lymphocytes is present. This infiltrate hugs the interface between the epithelium and connective tissue.  Basal Cell Degeneration (High Power View)  Evidence of liquefaction degeneration of the basal keratinocytes is visible. Damaged basal cells appear vacuolated or apoptotic.  Civatte Bodies (High Power View)  Eosinophilic, round apoptotic keratinocytes, also known as Civatte bodies, are scattered within the epithelium and superficial connective tissue.  Hyperkeratosis and Hypergranulosis  The epithelium shows hyperkeratosis, often with a thickened granular layer (hypergranulosis), although this may vary.  Absence of Dysplasia  There is no evidence of cellular atypia or dysplastic changes, supporting a benign inflammatory rather than neoplastic process.  Summary:  The combination of:  a band-like lymphocytic infiltrate at the epithelial-connective tissue interface,  basal cell degeneration,  Civatte bodies, and  saw-toothing of the rete pegs  is diagnostic of lichen planus, a chronic immune-mediated mucocutaneous disorder often seen in the oral cavity. These features clearly place the lesion within the granulomatous, immune-mediated, and autoimmune conditions category in oral and maxillofacial pathology. | Most probable histopathological diagnosis  Oral lichen planus (lichenoid mucositis) – an immune-mediated, interface mucositis that classically presents with a band-like T-cell infiltrate hugging the epithelium and basal-cell damage.  Microscopic features in the provided slides that support this diagnosis  Level Observations in the image Diagnostic significance  Low power • Stratified squamous epithelium with hyper-parakeratosis/orthokeratosis  • Irregular, “saw-tooth” rete ridges projecting into the lamina propria  • A dense, continuous band of predominantly lymphocytic inflammation immediately beneath the epithelium (interface pattern) Classic architecture of oral lichen planus – lichenoid band closely follows the epithelial interface and produces the saw-tooth contour  High power • Hydropic/ vacuolar degeneration of basal keratinocytes  • Numerous Civatte (colloid) bodies – round eosinophilic apoptotic keratinocytes within the epithelium and just below it  • Exocytosis of lymphocytes into the lower epithelium  • Inflammatory infiltrate composed almost entirely of small lymphocytes with occasional plasma cells, limited to the superficial lamina propria Marks a cytotoxic T-cell–mediated attack on basal keratinocytes, producing apoptotic bodies and interface change characteristic of lichen planus  Absence • No true granulomas, no necrosis, no vasculitis, no dysplastic epithelial changes Helps exclude other granulomatous or premalignant processes  How the features fit the diagnosis  Interface/lichenoid mucositis – The sharply demarcated, band-like lymphocytic infiltrate at the epithelial–connective-tissue junction with basal-cell degeneration is the hallmark of lichen planus.  Saw-tooth rete ridges – Result from uneven basal damage and reparative hyperplasia.  Civatte bodies & hydropic change – Reflect keratinocyte apoptosis triggered by the immune attack.  Predominantly T-cell infiltrate, little deeper inflammation – Supports an immunologically mediated process rather than an infectious granuloma.  Taken together, these findings are quintessential for oral lichen planus (or a lichenoid drug/contact reaction, which is histologically identical), placing it solidly within the “granulomatous, immune-mediated and autoimmune” category requested. |
| 8.26 | **Diagnosis:** Lichen planus affecting the dorsal tongue; **Key Microscopic Features:** There is atrophy of the filiform papillae with tapered rete ridges and a band of lymphocytes at the junction; parakeratosis with interface blurring and a lymphocytic band is present; spongiosis with lymphocyte migration and basal cell degeneration occurs without colloid bodies. | Histopathological Analysis of Provided H&E-Stained Images  Single Most Likely Histopathological Diagnosis:  Oral Lichen Planus (OLP) — a chronic immune-mediated mucocutaneous condition.  Key Microscopic Features Supporting This Diagnosis:  1. Low Magnification (First Image):  Saw-toothed rete ridges: The epithelial projections into the connective tissue exhibit a characteristic pointed, saw-tooth appearance.  Band-like lymphocytic infiltrate: A dense, subepithelial lymphocytic infiltrate hugs the interface between the epithelium and connective tissue.  Hyperkeratosis: Thickening of the keratin layer is noted on the epithelial surface.  2. Intermediate Magnification (Second Image):  Interface mucositis: The basal cell layer is disrupted by a band-like infiltrate of lymphocytes, causing vacuolar degeneration.  Civatte bodies (colloid bodies): Apoptotic keratinocytes are present within the basal and suprabasal layers and within the infiltrate.  Absence of epithelial dysplasia: Despite the chronic inflammation, the epithelium does not show cytologic atypia indicative of dysplasia.  3. High Magnification (Third Image):  Dense lymphocytic infiltrate: Mostly composed of T lymphocytes, these cells obscure the basal layer, consistent with interface dermatitis.  Hydropic degeneration of basal cells: Vacuolar changes and apoptotic keratinocytes confirm basal cell injury.  No granulomas: While this is in the category of granulomatous or immune-mediated lesions, true granuloma formation is absent, ruling out entities like sarcoidosis or Crohn's disease.  Conclusion:  The collective histologic picture — saw-tooth rete ridges, dense band-like lymphocytic infiltrate at the epithelial-connective tissue interface, basal cell degeneration, and Civatte bodies — is highly characteristic of Oral Lichen Planus, a chronic T-cell-mediated autoimmune condition.  Let me know if you'd like a comparison with other differential diagnoses (e.g., lichenoid mucositis, lupus erythematosus, chronic GVHD). | Histopathological diagnosis (single best):  Oral sarcoidosis – non-caseating “naked” granulomatous mucositis  Key microscopic features that point to sarcoidosis  Level of magnification Findings Diagnostic significance  Low power • Intact, mildly acanthotic stratified squamous epithelium.  • Patchy to confluent, pale‐lavender nodules filling the superficial lamina propria while deeper connective tissue is largely spared. Well-circumscribed, subepithelial granulomas without ulceration are typical for oral sarcoidosis lesions.  Intermediate power • Discrete and coalescing granulomas centred within the lamina propria.  • Each granuloma made of plump, eosinophilic epithelioid histiocytes; only a thin cuff of small lymphocytes (“naked” appearance). The paucity of peripheral lymphocytes distinguishes sarcoid granulomas from those of Crohn disease/orofacial granulomatosis, which show heavier lymphoplasmacytic infiltrates.  High power • Sheets of epithelioid histiocytes with oval nuclei and pale granular cytoplasm.  • Occasional Langhans/foreign-body-type multinucleated giant cells may be present (not always captured in the field).  • No central caseation, fibrinoid necrosis, vasculitis, or microorganisms.  • Rare intracytoplasmic asteroid or Schaumann bodies may be seen in giant cells (classic but not obligatory). Non-caseating epithelioid granulomas with minimal necrosis are the histologic hallmark of sarcoidosis. Absence of vasculitis rules out granulomatosis with polyangiitis; lack of caseous necrosis argues against mycobacterial/fungal infection.  Putting it together:  The biopsy shows multiple well-formed, non-caseating epithelioid granulomas with only a sparse rim of lymphocytes and no necrosis or vasculitis. This “naked granuloma” pattern is classic for sarcoidosis and, in the context of an oral mucosal lesion, supports the diagnosis of oral sarcoidosis rather than other granulomatous, immune-mediated conditions such as Crohn disease, orofacial granulomatosis, or granulomatosis with polyangiitis. |
| 8.27 | **Diagnosis:** Gingival lichen planus consistent with a drug-induced lichenoid reaction; **Key Microscopic Features:** Parakeratosis is present with epithelial thinning, sawtooth-shaped rete ridges, and a lymphocytic infiltrate; epithelial thinning with basal cell degeneration leads to subepithelial clefting, abundant colloid bodies, and occasional eosinophils within the epithelium. | Based on the provided hematoxylin and eosin (H&E) stained histopathological images at low and high magnification, the single most likely histopathological diagnosis is:  Lichen Planus  Key Microscopic Features Justifying the Diagnosis:  Band-like Lymphocytic Infiltrate (Interface Mucositis):  The lower magnification image reveals a dense, band-like infiltrate of predominantly lymphocytes hugging the epithelial-connective tissue junction. This is a classic feature of lichen planus.  Basal Cell Degeneration (Liquefaction Degeneration):  The high-power image shows evidence of degeneration of basal keratinocytes, often described as liquefaction or hydropic degeneration. This supports the interface pattern of inflammation.  Saw-tooth Appearance of Rete Ridges:  Although subtle, the rete ridges in the lower magnification image may show saw-tooth configurations, another supporting feature.  Civatte (Colloid) Bodies:  Eosinophilic apoptotic keratinocytes (Civatte bodies) are visible in the basal and suprabasal layers. These are remnants of damaged basal cells.  Hyperkeratosis and Hypergranulosis:  While not prominently visible in these sections, lichen planus typically shows compact orthokeratosis or parakeratosis, and thickening of the granular layer (hypergranulosis), which may be present in other sections.  Summary:  The presence of a dense, band-like lymphocytic infiltrate at the epithelial-connective tissue interface, basal cell degeneration, Civatte bodies, and possible rete ridge alterations are diagnostic hallmarks of oral lichen planus, an immunologically mediated mucocutaneous condition. | Histopathological diagnosis (single best):  Oral lichen planus – “lichenoid interface mucositis.”  Key microscopic features visible in the two H-E sections  Feature What you see in the images Diagnostic significance  Band-like (lichenoid) inflammatory infiltrate In the low-power view a dense, continuous, purple band of mainly small lymphocytes hugs the epithelial–connective-tissue junction and is sharply demarcated from the deeper, relatively cell-poor connective tissue. A classic hallmark of lichen planus; the T-cell–rich infiltrate “obscures” the basal layer.  Basal-cell degeneration / vacuolar change High-power view shows hydropic swelling and vacuolation of basal keratinocytes; several keratinocytes have dropped out, leaving empty “Max-Joseph” clefts. Interface damage caused by cytotoxic T-cells.  Civatte (colloid) bodies Round, eosinophilic, apoptotic keratinocytes are scattered in the lower spinous/basal layer and within the lichenoid band. Apoptotic debris is characteristic of lichen planus and other interface reactions.  Hyperkeratosis & saw-tooth rete ridges Surface epithelium is thickened and hyperkeratinised; rete pegs are pointed/angulated in the low-power image. Explains the clinically white (keratotic) appearance and is typical of lichen planus.  Absence of true granulomas, neutrophils, or epithelial dysplasia Inflammatory cells are mononuclear; no granulomatous aggregates or atypical epithelial changes are present. Helps exclude granulomatous diseases, lupus, dysplasia, etc.  Together, these features—an intense lichenoid T-cell band, basal-cell/interface damage with Civatte bodies and Max-Joseph clefts, plus hyperkeratosis with pointed rete ridges—are diagnostic of oral lichen planus. |
| 8.28 | **Diagnosis:** Gingival lichen planus; **Key Microscopic Features:** Severe thinning of the epithelium, subepithelial cleft formation, and a lymphohistiocytic band at the junction are evident; basal cell degeneration results in subepithelial clefting, which is a consistent artifact. | Based on the histopathological images you provided, which are hematoxylin and eosin (H&E) stained sections at low and higher magnifications, and considering they are from the "Granulomatous, Immune-Mediated, and Autoimmune Conditions" group of oral and maxillofacial pathology, here is the analysis:  Single Most Likely Histopathological Diagnosis:  Sarcoidosis  Key Microscopic Features Justifying the Diagnosis:  Well-formed non-caseating granulomas:  Seen in both low-power and high-power images.  The granulomas are discrete and composed of tightly clustered epithelioid histiocytes (pale, eosinophilic cytoplasm) with a central aggregation.  Lymphocytic cuffing is mild to moderate—common in sarcoidosis.  No central necrosis, helping to distinguish it from infectious granulomas (e.g., tuberculosis).  Absence of necrosis:  Caseation necrosis, which is typical of infectious granulomas like TB, is not present here.  Multinucleated giant cells (possible):  The high-power image suggests the presence of multinucleated giant cells, although not dramatically prominent.  Giant cells are a common component in sarcoidal granulomas.  Sparse lymphocytic infiltrate:  Compared to other granulomatous conditions like Crohn’s disease or granulomatosis with polyangiitis, sarcoidosis tends to have less intense surrounding lymphocytic inflammation.  Overlying intact stratified squamous epithelium:  The lesion is subepithelial, supporting the notion that it is a systemic condition manifesting locally, rather than a primary epithelial pathology.  Differential Diagnoses Considered but Less Likely:  Tuberculosis: Typically shows caseating necrosis; not seen here.  Crohn’s Disease: Granulomas are usually fewer, less well-formed.  Wegener’s (GPA): Usually shows necrotizing vasculitis and mixed inflammatory infiltrates.  Foreign body granuloma: Would often show identifiable foreign material. | Histopathological diagnosis  → Mucous-membrane pemphigoid (cicatricial pemphigoid) – a chronic, autoimmune sub-epithelial blistering disorder of the oral mucosa.  Key microscopic features that support the diagnosis  Feature (seen in the images) Diagnostic significance  1. Large, clear sub-epithelial cleft/blister – the entire stratified squamous epithelium is lifted off the underlying connective tissue leaving an empty space (white area) Hallmark of the pemphigoid group: auto-antibody–mediated separation occurs below the basal cell layer, so the epithelium remains intact and forms the blister roof.  2. Intact full-thickness epithelium forming the blister roof – no acantholysis or “tomb-stone” basal cells Rules out pemphigus vulgaris (which shows intra-epithelial suprabasal clefts with acantholysis).  3. Dense, band-like chronic inflammatory infiltrate in the lamina propria immediately beneath the cleft – predominantly lymphocytes and plasma cells, with scattered histiocytes/eosinophils Characteristic inflammatory reaction in mucous-membrane pemphigoid; eosinophils are common but not obligatory.  4. Deeper zone of organised collagenous fibrous tissue (pink, swirling bundles in the low-power view) Reflects the scarring/cicatricial healing that typifies mucous-membrane pemphigoid.  5. Absence of granulomatous inflammation, caseation, or foreign-body giant cells Helps exclude granulomatous diseases such as sarcoidosis, Crohn-related orofacial granulomatosis, tuberculosis, etc.  Together, the wide sub-epithelial split with an intact epithelial roof and the subjacent mixed chronic infiltrate and fibrosis are classic for mucous-membrane (cicatricial) pemphigoid. |
| 8.32 | **Diagnosis:** Chronic graft-versus-host disease; **Key Microscopic Features:** The oral mucosa shows a band-like lymphocytic infiltrate at the epithelial-connective tissue junction; basal cell degeneration with colloid body formation, interface obscuration, and lymphocytic infiltrate at the junction are present. | Histopathological Analysis of Provided H&E-Stained Images  Single Most Likely Histopathological Diagnosis:  Oral Lichen Planus (OLP) — likely the erosive or reticular subtype.  Key Microscopic Features Supporting This Diagnosis:  1. Interface Mucositis (Basal Cell Degeneration):  Both images show liquefactive degeneration of the basal cell layer of the epithelium.  This is a hallmark of interface dermatitis/mucositis, commonly seen in lichen planus.  2. Band-like Lymphocytic Infiltrate:  In the lower portion of the epithelium and superficial lamina propria, there is a dense, band-like infiltrate of small lymphocytes.  This lymphocytic infiltrate is closely associated with basal keratinocyte damage.  3. Saw-toothing of Rete Ridges:  The rete pegs exhibit a characteristic "saw-toothed" appearance, especially visible in the lower magnification image.  This is another supportive feature of OLP.  4. Civatte Bodies (Colloid Bodies):  At higher magnification (second image), there are eosinophilic, round apoptotic keratinocytes within the basal layer or lamina propria (suggestive of Civatte bodies).  These are degenerated basal cells phagocytosed by histiocytes or lying free.  5. Hyperkeratosis and Acanthosis:  While not markedly thickened, there is mild acanthosis and parakeratosis/hyperkeratosis in the epithelium.  These features support a chronic lichenoid process.  Conclusion:  These features collectively are diagnostic for Oral Lichen Planus, a chronic immune-mediated mucocutaneous disorder. While other conditions such as lichenoid mucositis, lupus erythematosus, or chronic GVHD can mimic this appearance, the band-like infiltrate, basal degeneration, and Civatte bodies strongly favor classic Oral Lichen Planus. | Histopathological diagnosis  Oro-facial granulomatosis / non-caseating granulomatous mucositis  (the pattern seen is the same as that encountered in oral Crohn disease or sarcoidosis, but in an isolated oral biopsy the descriptive diagnostic term “orofacial granulomatosis” is preferred).  Key microscopic features that support this diagnosis  Field Observations from the supplied H-E sections  Low-power view • Intact stratified squamous oral epithelium with mild acanthosis and elongated, club-shaped rete ridges.  • Lamina propria shows patchy, nodular accumulations of pale cells interrupting the usual collagen – a classic “peppered-rosy” appearance of granulomas.  • No ulceration, no surface pseudo-membrane, and no caseous necrosis.  High-power view • Collections of epithelioid histiocytes with abundant, finely granular, eosinophilic cytoplasm and oval vesicular nuclei – i.e. non-caseating granulomas.  • Intermixed multinucleated giant cells (foreign-body/Langhans type) can be seen in several foci.  • Dense peripheral cuff of lymphocytes and plasma cells, so the granulomas are not “naked” (helps separate the lesion from sarcoidosis).  • Granulomas often lie perivascularly; several small vessels are engulfed but show no fibrinoid necrosis or vasculitis (rules out granulomatosis with polyangiitis).  Negative findings (equally important) • No central necrosis (rules out mycobacterial/tuberculous granulomas).  • No neutrophil-rich microabscesses or palisading necrosis (would suggest Wegener).  • No embedded exogenous polarisable material (rules out foreign-body granuloma).  Why these features point to orofacial granulomatosis  Non-caseating granulomas are the defining lesion; in the oral cavity they narrow the differential to Crohn disease, sarcoidosis and idiopathic orofacial granulomatosis.  The granulomas here are ill-defined and lymphocyte-rich, not the “naked” sharply-circumscribed granulomas typical of sarcoidosis.  There is no transmural, deep fissuring, or crypt architectural distortion that would suggest a Crohn bowel biopsy; in an isolated oral specimen the safest pathologist’s call is “orofacial granulomatosis”.  Hence, taken together, the spectrum of findings most convincingly supports orofacial granulomatosis / non-caseating granulomatous mucositis. |
| 8.35 | **Diagnosis:** Recurrent oral erythema multiforme; **Key Microscopic Features:** A subepithelial blister is present; the blister contains eosinophils and numerous monocytes; lymphocyte satellitosis is observed as a ring of lymphocytes surrounding necrotic keratinocytes. | Based on the histopathological examination of the three provided hematoxylin and eosin (H&E) stained images, which show varying magnifications of the same lesion in the context of granulomatous, immune-mediated, and autoimmune conditions of the oral and maxillofacial region, the single most likely diagnosis is:  Histopathological Diagnosis:  Wegener's Granulomatosis (Granulomatosis with Polyangiitis - GPA)  Key Microscopic Features Supporting the Diagnosis:  Necrotizing Granulomatous Inflammation:  The first image (low magnification) shows geographic necrosis surrounded by a rim of palisading histiocytes and multinucleated giant cells, which is characteristic of necrotizing granulomatous inflammation.  The "dirty necrosis" with nuclear debris and pink amorphous material is a hallmark of Wegener's/GPA.  Granuloma Formation:  The second image (intermediate magnification) reveals well-formed granulomas composed of epithelioid histiocytes and multinucleated giant cells.  There's perivascular granulomatous inflammation, which is a signature finding in GPA.  Vasculitis (Necrotizing):  The third image (higher magnification) demonstrates transmural inflammation of blood vessels, with fibrinoid necrosis of vessel walls and infiltration by neutrophils, eosinophils, and lymphocytes—indicative of necrotizing vasculitis.  Evidence of endothelial damage and lumen compromise is also seen, consistent with the vasculitic component of GPA.  Additional Notes:  GPA is a systemic necrotizing vasculitis that often involves the respiratory tract and kidneys, but in the oral cavity, it may present as ulceration, "strawberry gingivitis," or other mucosal lesions.  The triad of necrotizing granulomas, vasculitis, and necrosis seen in these images is pathognomonic.  If immunohistochemical or serological data were available (e.g., c-ANCA positivity), they would further confirm this diagnosis. | Histopathological diagnosis (single best):  Mucous-membrane pemphigoid (cicatricial pemphigoid) – an autoimmune, sub-epithelial vesiculo-bullous disorder of the oral mucosa.  Key microscopic features visible in the provided H-E sections  Level of magnification What you can see Significance for the diagnosis  Low power (first photo) • Large, well-circumscribed sub-epithelial cleft/blister separating the entire surface epithelium from the underlying connective tissue.  • The blister roof is intact stratified squamous epithelium; the basal layer remains attached to the roof, not the floor. a. Confirms that the split occurs below the basement membrane zone (characteristic of pemphigoid spectrum, in contrast to suprabasilar split of pemphigus).  Intermediate power (second photo) • The blister cavity contains fibrinous exudate, scattered neutrophils and a few eosinophils.  • Cleft margins show wisps of lamina densa/anchoring fibrils still attached to the roof. b. Inflammatory cell mix, especially neutrophils ± eosinophils, is typical for pemphigoid blisters.  High power (third photo) • The subjacent lamina propria is packed with a dense, band-like chronic inflammatory infiltrate rich in lymphocytes and plasma cells, with early collagen remodeling (incipient fibrosis/scarring).  • No acantholysis, no well-formed epithelioid granulomas, no caseation necrosis. c. Heavy chronic inflammation and scarring (“cicatricial” tendency) fit mucous-membrane pemphigoid; absence of acantholysis excludes pemphigus, and lack of granulomas rules out sarcoidosis/Crohn-related orofacial granulomatosis.  Putting the features together  A clean sub-epithelial split with an intact epithelial roof eliminates pemphigus vulgaris/erythema multiforme.  Neutrophils ± eosinophils inside the cleft, plus a plasma-cell-rich band in the lamina propria, are classic for pemphigoid.  Early fibrosis (even in small biopsies) is why the condition is also called cicatricial pemphigoid.  Consequently, the constellation of findings most strongly supports mucous-membrane pemphigoid. (Direct immunofluorescence, although not shown here, would typically demonstrate linear IgG and/or C3 along the basement-membrane zone and would clinch the diagnosis in practice.) |
| 8.39 | **Diagnosis:** Mucous membrane pemphigoid; **Key Microscopic Features:** There is acanthosis with a subepithelial cleft, accompanied by a mild lymphocytic infiltrate, preservation of basal cells, and minimal inflammation; epithelial thinning with subbasal clefting is present, and basal cells protrude into a fibrin-containing cleft. | Single Most Likely Histopathological Diagnosis:  Benign Mucous Membrane Pemphigoid (MMP)  Key Microscopic Features Justifying the Diagnosis:  1. Separation at the Basement Membrane (Subepithelial Clefting):  Especially visible in the second and third images.  There is a clear subepithelial blister, with the epithelial layer lifting away from the underlying connective tissue, which is characteristic of MMP and distinguishes it from intraepithelial blistering diseases like pemphigus vulgaris.  2. Intact Epithelium with Basal Layer Preservation:  The epithelium above the cleft appears largely intact and not acantholytic (i.e., cells are not falling apart), which helps rule out pemphigus.  The basal cells are not significantly dyskeratotic or detached individually, supporting a subepithelial rather than intraepithelial process.  3. Inflammatory Infiltrate in the Connective Tissue:  Dense chronic inflammatory infiltrate in the lamina propria, predominantly lymphocytic and plasma cell-rich, often with scattered eosinophils.  Seen particularly well in the first image (low power), which shows a band-like infiltrate underneath the cleft.  4. Epithelial Hyperplasia and Rete Ridge Elongation:  The second image shows irregular epithelial hyperplasia, with elongated rete ridges. This is a reactive change commonly seen in chronic immune-mediated mucosal diseases.  Correlative Clinical Relevance:  MMP is a chronic autoimmune disease that primarily affects mucosal surfaces, including the oral cavity. Histologically, it features subepithelial clefting with minimal acantholysis and a chronic inflammatory infiltrate.  To confirm, direct immunofluorescence (DIF) showing linear IgG and/or C3 along the basement membrane zone would be essential. | Histopathological diagnosis  → Oral lichen planus (lichenoid mucositis pattern)  Key microscopic features present in the three fields that lead to this diagnosis  Image What is seen Why it points to lichen planus  Low-power overview Epithelium is thickened and hyper-keratinised; rete ridges are long, narrow and “saw-toothed.” Immediately beneath the epithelium is a broad, dense, continuous band of inflammatory cells hugging the epithelial–connective-tissue junction. The characteristic “lichenoid” band-like lymphocytic infiltrate situated tight against an acanthotic epithelium with pointed rete processes is the classic silhouette of lichen planus.  Intermediate magnification Hydropic (vacuolar) degeneration of basal keratinocytes; scattered eosinophilic apoptotic (Civatte/colloid) bodies. Small subepithelial clefts have formed where the damaged basal layer has lifted away. Basal-cell liquefaction with Civatte bodies and formation of Max-Joseph spaces (subepithelial clefts) are hallmark changes produced by a cell-mediated attack on the basal layer in lichen planus.  High magnification of interface A compact, predominantly T-lymphocytic infiltrate occupies the superficial lamina propria; it is “lichenoid” (band-like) with very little separation from the damaged basal layer. The overlying spinous layer shows regular, polygonal keratinocytes without dysplasia. The dense, interface-hugging lymphoid band together with basal damage, but without epithelial dysplasia, typifies an immunologically mediated lichenoid reaction rather than dysplastic or pemphigoid processes.  Putting the features together  Interface (lichenoid) mucositis – continuous, band-like lymphocytic infiltrate.  Basal-cell liquefaction & Civatte bodies – evidence of cytotoxic attack on basal keratinocytes.  “Saw-tooth” rete ridges & surface hyper-/para-keratosis – common architectural response.  Subepithelial (Max-Joseph) clefts – produced by loss of the basal layer, not true bullae as in pemphigoid.  No suprabasilar acantholysis (“row-of-tombstones”) is present, ruling out pemphigus; the split is subepithelial and accompanies a dense lichenoid infiltrate rather than the looser mixed infiltrate of pemphigoid. Taken together, the constellation of findings is diagnostic of oral lichen planus. |
| 8.45 | **Diagnosis:** Pemphigus vulgaris; **Key Microscopic Features:** The oral mucosa shows suprabasal cleft formation with rounded acantholytic cells within the cleft; the villous-like connective papillae are lined by one to two layers of basal and parabasal keratinocytes. | Histopathological Analysis of the Provided H&E-Stained Images  Single Most Likely Histopathological Diagnosis:  Orofacial Granulomatosis (OFG)  Key Microscopic Features Supporting the Diagnosis:  1. Granulomatous Inflammation:  The high-power image shows well-formed, non-caseating granulomas in the lamina propria, composed of:  Aggregates of epithelioid histiocytes  Occasional multinucleated giant cells  Surrounding lymphocytic infiltrate  These granulomas are not associated with necrosis, supporting a non-infectious, immune-mediated etiology.  2. Epithelial Changes:  The low-power image shows acanthosis (epithelial thickening) and elongated rete ridges, typical of chronic irritation or immune-mediated epithelial response.  Mild spongiosis may be noted, although secondary.  3. Perivascular Infiltrate and Edema:  There is perivascular lymphocytic infiltration and some vascular congestion, common in OFG due to immune activation and vascular involvement.  Subepithelial edema may also be contributing to the architectural distortion.  Additional Considerations:  Orofacial Granulomatosis is a diagnosis of exclusion, and other causes of granulomatous inflammation (e.g., Crohn’s disease, sarcoidosis, tuberculosis) must be ruled out clinically and with ancillary tests. The absence of caseation necrosis and the pattern of inflammation favor a non-infectious granulomatous process like OFG. | Histopathological diagnosis – Pemphigus vulgaris (suprabasilar immunobullous disease)  Key microscopic features in the two H-E sections that point to pemphigus vulgaris  Field What is seen How it supports the diagnosis  Low-power view (first image) Irregular suprabasilar cleft/blister: a pale cavity has opened within the epithelium just above the basal layer; numerous acantholytic keratinocytes float in it while the basal cells remain attached to the lamina propria, creating the classic “row-of-tombstones” outline. A split that is intra-epithelial (not sub-epithelial) is characteristic of pemphigus, differentiating it from mucous-membrane pemphigoid and other bullous disorders.  Pseudo-epitheliomatous hyperplasia of the surrounding squamous epithelium. Frequently accompanies the chronic erosive lesions of PV in the mouth.  Mild chronic inflammatory cell infiltrate and extravasated erythrocytes in the superficial lamina propria. Non-specific but expected background in an active PV erosion/ulcer.  High-power view (second image) Acantholytic (Tzanck) cells: rounded keratinocytes with hyperchromatic nuclei and a perinuclear halo lie free in the blister fluid and in small micro-vesicles between epithelial cells. Loss of desmosomal adhesion (acantholysis) produced by auto-antibodies to desmoglein-3 is the histologic hallmark of PV.  Basal keratinocytes intact on basement membrane: they outline small oval or elongated islands (“tombstones”) because their inter-cell adhesion is preserved while their attachments to suprabasal neighbours are lost. Confirms the split is suprabasilar – the defining microscopic criterion for pemphigus vulgaris.  Scattered neutrophils & eosinophils within the blister cavity and around dilated superficial vessels; occasional RBC clusters. A common secondary reaction in oral PV lesions.  Taken together—the suprabasilar intra-epithelial cleft, abundant acantholytic (Tzanck) cells, and basal “row-of-tombstones” pattern—these findings are diagnostic of pemphigus vulgaris in the oral mucosa. |
| 8.50 | Diagnosis: Lupus erythematosus of the oral mucosa; Key Microscopic Features: The epithelium demonstrates hyperkeratosis with alternating areas of atrophy and acanthosis and a persistent subepithelial lymphocytic band, while the basal layer shows degeneration with apoptotic keratinocytes, a thickened basement membrane, and continued lymphocytic infiltration. | Histopathological Analysis  Single Most Likely Histopathological Diagnosis:  Lichen Planus  Key Microscopic Features Supporting the Diagnosis:  Low Magnification (Image 1):  Saw-tooth rete ridges – The rete pegs appear pointed and angulated, a hallmark of lichen planus.  Band-like lymphocytic infiltrate – Dense, band-like accumulation of lymphocytes along the epithelial-connective tissue interface (lichenoid interface dermatitis).  Hyperkeratosis and acanthosis – The epithelium shows thickening (acanthosis) and surface keratinization (hyperkeratosis), common in lichen planus.  High Magnification (Image 2):  Basal cell degeneration – Vacuolization and damage of the basal keratinocytes, often leading to Civatte bodies (eosinophilic apoptotic keratinocytes).  Interface mucositis – Lymphocytes infiltrate the basal layer and blur the epithelial-connective tissue boundary.  Predominantly T-lymphocytic infiltrate – Suggestive of a T-cell mediated immune response, characteristic of autoimmune/immune-mediated pathologies.  Conclusion:  The combination of saw-tooth rete ridges, band-like subepithelial lymphocytic infiltrate, and basal cell degeneration strongly supports the diagnosis of oral lichen planus, a classic immune-mediated mucocutaneous disorder. This pattern rules out other granulomatous or autoimmune conditions such as lupus erythematosus or mucous membrane pemphigoid, which lack this specific constellation of features. | Histopathological diagnosis  → Oral Lichen Planus (lichenoid mucositis)  Key microscopic features that support this diagnosis  Feature How it appears in the provided images Diagnostic significance  Dense, band-like (“lichenoid”) inflammatory infiltrate Both images show a ribbon-like sheet of small, dark lymphocytes hugging the epithelial–connective-tissue junction. Characteristic of lichen planus; the infiltrate is almost always T-cell–rich and restricted to the superficial lamina propria.  Hydropic (liquefaction) degeneration of the basal layer In the higher-power view the basal keratinocytes are swollen, pale, and focally disappearing; the epithelium looks “lifted” off the connective tissue. Basal cell vacuolization is a hallmark of an interface/lichenoid mucositis.  Civatte (colloid) bodies / apoptotic keratinocytes Eosinophilic, rounded bodies are scattered at the epithelial base and within the infiltrate. Represent apoptotic basal cells—classic for lichen planus.  Saw-tooth configuration of rete ridges The low-power image reveals pointed, angulated rete pegs rather than broad blunted ones. A classic architectural clue to lichen planus.  Hyperparakeratosis / slight acanthosis The surface epithelium is thickened with a dense, pink keratin layer. Common secondary change in reticular/erosive OLP lesions.  Lymphocytic exocytosis Individual lymphocytes are seen migrating into the lower spinous layer. Supports an interface pattern of mucosal inflammation.  Together, this constellation of a lichenoid, T-cell–rich band of inflammation, basal cell degeneration with Civatte bodies, and saw-tooth rete ridges is diagnostic of oral lichen planus and distinguishes it from other immunologically mediated oral lesions (e.g., pemphigus vulgaris, mucous-membrane pemphigoid, or granulomatous diseases). |
| 8.52 | Diagnosis: Granulomatosis with polyangiitis; Key Microscopic Features: The lesion shows pseudoepitheliomatous hyperplasia with numerous abscesses and fresh hemorrhage beneath the epithelium, a mixed inflammatory infiltrate with geographic necrosis, and both acute and chronic inflammation rich in eosinophils and nuclear debris. | Based on the provided hematoxylin and eosin (H&E) stained histopathological images at different magnifications, the single most likely diagnosis is:  Diagnosis: Sarcoidosis  Key Microscopic Features Supporting the Diagnosis:  Well-formed Non-caseating Granulomas:  Clearly seen in the first image (low magnification), there are multiple discrete granulomas composed predominantly of epithelioid histiocytes.  These granulomas are sharply demarcated from surrounding tissue and lack central necrosis, a hallmark of non-caseating granulomas, which is characteristic of sarcoidosis.  Multinucleated Giant Cells:  Present in the medium and high magnification images (second and third images).  These cells, often Langhans-type or foreign-body type giant cells, are commonly found within sarcoid granulomas.  Sparse or Absent Necrosis:  A critical differential point from granulomatous infections like tuberculosis, where caseation necrosis is prominent. Here, necrosis is not evident.  Lymphocytic Infiltrate at the Periphery of Granulomas:  The granulomas are often surrounded by a sparse rim of lymphocytes, which is a common finding in sarcoidosis but not as dense as in other autoimmune granulomatous diseases.  Absence of Infectious Organisms:  No organisms (fungal or mycobacterial) are seen in the background or within granulomas, which helps rule out infectious causes.  Differential Diagnosis Considerations and Exclusion:  Tuberculosis: Typically features caseating necrosis, which is absent here.  Crohn’s Disease: Granulomas in Crohn’s are usually less well-formed and often show transmural inflammation in the GI tract.  Wegener’s Granulomatosis (GPA): More likely to show necrotizing granulomas and vasculitis, which are not seen here.  Fungal Infections: Usually reveal organisms on special stains and often involve necrosis. | Histopathological diagnosis  Non-caseating sarcoidal granulomatous inflammation (oral sarcoidosis).  Key microscopic features that support this diagnosis  Magnification What is seen Why it points to sarcoidosis  Low power (first image) Several well-circumscribed, discrete nodules of pale epithelioid cells set in a minor salivary-gland stroma; only a very thin rim of lymphocytes (“naked” granulomas); no central caseous necrosis The combination of tightly packed granulomas, scant peripheral lymphocytes and absence of necrosis is characteristic of sarcoidal granulomas rather than infectious or necrotising types  Medium power (second image) Granulomas lie between intact salivary ducts and acini; ducts are pushed aside but not destroyed; stroma contains scattered lymphocytes and plasma cells Oral sarcoidosis most often presents in minor salivary glands where granulomas replace the parenchyma but spare the duct epithelium  High power (third image) Sheets of epithelioid histiocytes with elongated, pale cytoplasm and oval nuclei; rare multinucleated giant cells; occasional extravasated RBCs but no vasculitic fibrinoid necrosis Non-caseating epithelioid histiocytes and occasional Langhans/foreign-body giant cells without necrosis typify sarcoidosis; absence of vasculitis helps exclude granulomatosis with polyangiitis  Additional supportive clues  Lack of central neutrophilic abscess (rules out cat-scratch disease or suppurative granulomas)  No refractile foreign material or polarizable particles (rules out foreign-body reaction)  No broad zones of “geographic” necrosis or vessel wall destruction (rules out granulomatosis with polyangiitis)  Minor salivary-gland setting fits the common oral manifestation of systemic sarcoidosis.  Together, the presence of multiple “naked,” non-caseating epithelioid granulomas within minor salivary-gland tissue, in the absence of necrosis or vasculitis, establishes sarcoidosis as the single most likely histopathological diagnosis. |

PIGMENTED LESIONS

| **Fig_no** | **Ground truth** | **GPT 4o answer** | **o3 answer** |
| --- | --- | --- | --- |
| 9.2 | Diagnosis: Amalgam Tattoo; Key Microscopic Features: Pigmented particles accompanied by dense scarring, foreign body reaction with large amalgam particles inside foreign body giant cells, and fine particles deposited along connective tissue fibers, perineurium, and vascular basement membrane. | Histopathological Analysis of the Provided H&E-Stained Images  Most Likely Histopathological Diagnosis:  Oral Melanotic Neuroectodermal Tumor of Infancy (MNTI)  Key Microscopic Features Supporting This Diagnosis:  1. Biphasic Cellular Population (Best seen in image 3):  The third image, at higher magnification, shows a dual cell population:  Small, round neuroblastic-like cells with hyperchromatic nuclei.  Larger epithelioid cells with vesicular nuclei and pale cytoplasm.  These two populations are arranged in a somewhat alveolar or nesting pattern within a fibrous stroma, a hallmark of MNTI.  2. Melanin Pigment Presence (Best seen in image 2):  Image 2 reveals brown-black pigment granules within the cytoplasm of the tumor cells and extracellularly.  The pigment is consistent with melanin, confirming the lesion is pigmented in nature.  Pigment is found in both the epithelial-like and neuroblastic components, which is typical for MNTI.  3. Subepithelial Tumor with Overlying Acanthotic Epithelium (Seen in image 1):  The low-magnification image shows a well-circumscribed, lobulated tumor mass in the subepithelial connective tissue.  The overlying oral mucosal epithelium appears acanthotic but intact, suggesting a benign growth pattern without surface ulceration or invasion.  Dense fibrous stroma surrounding the pigmented tumor nests is evident.  4. Supporting Stroma and Inflammatory Cells:  A fibrovascular stroma with some chronic inflammatory cells is present.  MNTIs often provoke a fibroblastic stromal response, which is visible here.  Conclusion:  The constellation of features—biphasic cell population, melanin pigmentation, subepithelial location, and well-demarcated lobulated architecture—strongly supports the diagnosis of Melanotic Neuroectodermal Tumor of Infancy (MNTI). This rare, pigmented, predominantly benign tumor typically occurs in infants and often involves the anterior maxilla. | Most likely histopathological diagnosis  → Amalgam tattoo (focal oral argyrosis)  Key microscopic features visible in the three H-E sections  Low-, medium- and high-power findings How they point to an amalgam tattoo  1. Overlying stratified squamous epithelium is intact and unremarkable (no junctional/compound nests, no atypical melanocytes). Rules out melanocytic proliferations (e.g., melanocytic nevus, melanoma, melanoacanthoma).  2. Irregular black-to-dark-brown opaque particles of very variable size and shape are scattered within the lamina propria and deeper connective tissue. Some lie along collagen bundles and vascular walls, others aggregate into larger chunks. Typical appearance of exogenous metallic alloy (predominantly silver) fragments derived from dental amalgam. Endogenous melanin is fine and dust-like inside macrophages, not coarse, opaque, and extracellular.  3. Foreign-body–type multinucleated giant-cell reaction and a rim of chronic inflammatory cells/fibrosis around the larger fragments (best appreciated in the medium-power image). The tissue attempts to wall off inert amalgam pieces, producing a granulomatous response that is common in amalgam tattoos but absent in physiologic pigmented lesions.  **4. Surrounding collagen bundles show a hyalinized, scar-like eosinophilic stroma; no cellular atypia or mitotic activity. Consistent with a chronic, inert foreign material rather than a neoplasm.  **5. Fine, dust-like metallic granules are also seen inside and around small vessel walls in the high-power field. Metallic ions can disseminate along vascular or lymphatic channels, another classic feature of focal argyrosis.  Taken together, the presence of extracellular, opaque, metallic-looking pigment particles, the foreign-body granulomatous reaction, and the completely normal epithelium strongly support an amalgam tattoo rather than any endogenous melanocytic or vascular pigmented lesion of the oral cavity. |
| 9.3 | Diagnosis: Amalgam Tattoo; Key Microscopic Features: Presence of a foreign body granuloma containing coarse amalgam particles, with staining observed in the basement membrane of the epithelium. | Histopathological Analysis of the Pigmented Lesion (H&E Stained Slides)  Single Most Likely Histopathological Diagnosis:  Oral Melanotic Macule  Key Microscopic Features Supporting the Diagnosis:  Basal Layer Pigmentation:  There is increased melanin pigmentation within the basal cell layer of the epithelium. This is evident in both images, particularly the second high-magnification image, where brown-black pigment granules are present along the basal layer.  Melanin Incontinence:  Melanin pigment is seen in the superficial lamina propria, within melanophages (pigment-laden macrophages), which is a hallmark of melanin incontinence and supports a diagnosis of a benign pigmented lesion like a melanotic macule.  No Atypia or Nesting:  The epithelial architecture is intact, with no dysplasia, atypical melanocytes, or melanocytic nesting. This rules out neoplastic lesions like melanoma or nevus.  Inflammatory Infiltrate:  Mild chronic inflammatory cell infiltrate is visible in the connective tissue, especially surrounding blood vessels, which can be a reactive component.  Absence of Junctional Activity:  There is no evidence of junctional or dermal melanocytic proliferation, which is critical in excluding melanocytic nevi or melanoma.  Summary:  The combination of increased basal pigmentation, melanin incontinence without melanocytic atypia or proliferation, and a lack of architectural distortion is most consistent with an oral melanotic macule, a benign, localized pigmented lesion often found on the lip or buccal mucosa. | Histopathological diagnosis  Pigmented oral lichen planus (lichen planus with melanin-incontinence).  Key microscopic features visible in the two fields that support this diagnosis  Level Feature Appearance in the images  Epithelium • Mild hyper-parakeratosis and acanthosis  • Characteristic “saw-tooth” undulating rete ridges The surface epithelium is thickened, and the bases of the rete pegs show the sharp, pointed configuration typical of lichen planus.  Basal cell layer • Liquefaction / hydropic degeneration of basal keratinocytes  • Occasional eosinophilic Civatte (colloid) bodies Pale, vacuolated basal cells and scattered round eosinophilic apoptotic bodies can be picked out along the epithelial–connective-tissue junction.  Interface band • Dense, continuous, “band-like” inflammatory infiltrate hugging the underside of the epithelium, composed almost entirely of small lymphocytes In both magnifications a ribbon of dark‐blue nuclei forms an uninterrupted band immediately beneath the basement membrane.  Pigmentation (melanin incontinence) • Abundant fine, golden-brown pigment inside macrophages (melanophages) and free in the superficial lamina propria Numerous brown–black granules are clustered within macrophages and along collagen bundles just beneath the epithelium, producing the clinical pigmentation for which this variant is noted.  Additional supportive findings • Focal disruption of the basement membrane (Max-Joseph spaces)  • Absence of epithelial dysplasia or atypical melanocytic proliferation Small clefts can be seen at the epithelium–connective tissue interface, but the overlying epithelial cells retain normal maturation and nuclear features, excluding dysplasia or melanoma.  Taken together, the classic lichenoid interface change (saw-tooth rete ridges, basal-cell degeneration, Civatte bodies, dense subepithelial lymphocytic band) plus prominent melanin incontinence explains both the morphology and the clinical pigmentation, making pigmented oral lichen planus the single most appropriate diagnosis. |
| 9.6 | Diagnosis: Graphite Tattoo; Key Microscopic Features: Granulomatous inflammation with fibrosis, and irregular, large, and small black particles surrounded by macrophages. | Based on the provided hematoxylin and eosin (H&E) stained histological images at low and high magnification, this lesion belongs to the PIGMENTED LESIONS group of oral and maxillofacial pathology.  🧠 Single Most Likely Histopathological Diagnosis:  Malignant Melanoma  🔬 Key Microscopic Features Supporting the Diagnosis:  Low-Magnification (1st Image):  A nodular, expansile lesion situated in the subepithelial connective tissue.  The overlying stratified squamous epithelium appears intact, with no signs of ulceration.  The lesion is well-demarcated but non-encapsulated, indicating invasive growth.  The mass is composed of heavily pigmented cells, which is a hallmark of melanin-containing neoplasms.  High-Magnification (2nd Image):  Numerous pleomorphic, hyperchromatic tumor cells with prominent nucleoli, indicative of malignancy.  Intracytoplasmic and extracellular melanin pigment (seen as dark brown to black granules), confirming melanocytic origin.  Evidence of cellular atypia, including nuclear pleomorphism, mitotic figures, and multinucleation.  Some melanophages may be present, containing ingested melanin, but the majority of pigmented cells appear neoplastic.  🧬 Diagnostic Considerations:  The high melanin content and malignant cytologic features point away from benign lesions like melanotic macule or nevus.  The presence of cellular atypia, pleomorphism, and mitotic activity excludes benign melanocytic nevi.  Pigmented squamous cell carcinoma or amalgam tattoo would not show this level of melanocytic differentiation or cytologic atypia.  ✅ Conclusion:  The histologic features — pleomorphic melanocytic cells with heavy pigmentation, high mitotic activity, and subepithelial invasive pattern — are diagnostic of oral malignant melanoma, a rare but aggressive neoplasm in the oral cavity. | Histopathological diagnosis (single best):  Blue nevus of the oral mucosa (common / conventional type).  Key microscopic features supporting the diagnosis  Feature Description seen in the photomicrographs Significance  Symmetry & sharp circumscription Low-power view shows a smooth, dome-shaped, well-demarcated nodule seated entirely within the lamina propria/submucosa; the lesion is bounded by a thin fibrous capsule-like rim and is architecturally symmetrical from side-to-side. Pattern typical of a benign melanocytic proliferation such as a blue nevus.  Intact overlying epithelium with a “grenz zone” Stratified squamous epithelium is normal; there is a narrow band of collagen separating it from the tumor (grenz zone). Absence of junctional/nesting activity argues against compound/intradermal nevus or mucosal melanoma.  Spindle- and dendritic-shaped melanocytes At intermediate and high power, slender spindled cells with elongated or wavy nuclei are arranged in short fascicles and whorls. Cell morphology is classic for the melanocytes of a blue nevus.  Heavy coarse brown-black pigment Melanin is abundant, appearing as coarse, intensely black granules within the tumor cells and in numerous melanophages. Dense intracellular melanin is characteristic of blue nevus; the pigment explains the clinical “blue/black” coloration.  Lack of significant cytologic atypia or mitotic activity Nuclei are small to medium with inconspicuous nucleoli; mitoses and pleomorphism are minimal to absent. Confirms a benign melanocytic lesion, helping exclude malignant melanoma.  Taken together, the combination of a subepithelial, symmetrical, heavily pigmented spindle-cell proliferation with no junctional component and minimal cytologic atypia is diagnostic of a blue nevus in the oral cavity. |
| 9.8 | Diagnosis: Quinacrine Pigmentation; Key Microscopic Features: Pigmented particles are present in the lamina propria without tissue reaction, with fine dark-brown spherical pigment granules within macrophages or dendritic cells. | Based on the provided hematoxylin and eosin (H&E) stained histologic images of an oral pigmented lesion at different magnifications, the single most likely histopathological diagnosis is:  Oral Melanotic Macule  Key Microscopic Features Supporting This Diagnosis:  Increased Melanin Pigmentation in the Basal Layer:  The basal cell layer of the epithelium (top purple-stained layer) shows prominent melanin pigmentation. Melanin appears as brownish granules, primarily localized to the basal keratinocytes and melanophages in the superficial lamina propria.  Melanin-Laden Macrophages (Melanophages) in the Lamina Propria:  The second image (higher magnification) clearly demonstrates numerous melanophages within the connective tissue (lamina propria), appearing as cells with coarse, dark brown pigment.  Lack of Atypia or Junctional Activity:  There is no evidence of melanocytic proliferation along the basal layer (i.e., no nests or upward spread of melanocytes), which helps rule out melanocytic nevi or melanoma.  Normal Epithelial Architecture:  The stratified squamous epithelium appears orderly without dysplasia or hyperplasia, supporting a benign diagnosis.  Conclusion:  These features—particularly the presence of melanin pigment in basal keratinocytes and melanophages in the subepithelial connective tissue without melanocytic proliferation—are hallmark characteristics of an oral melanotic macule, a benign pigmented lesion commonly found on the lips or oral mucosa. | Histopathological diagnosis  Amalgam tattoo (focal argyrosis) – an exogenous metallic-pigment deposition in the oral mucosa.  Key microscopic features visible in the provided H-E sections  Feature What you see in the images How it supports the diagnosis  Irregular, opaque black-brown particles in the lamina propria Numerous coarse, angular to granular deposits scattered between collagen bundles and around small vessels (best appreciated in the high-power image). Heavy, extracellular metallic fragments are characteristic of an amalgam tattoo; melanin is usually finer and intracellular.  Linear/filamentous streaks of pigment following collagen and elastic fibers Pigment tracks along wavy pink collagen bundles rather than being confined to cells. Exogenous silver/mercury particles from dental amalgam often precipitate along connective-tissue fibers, producing this pattern.  Relative paucity of inflammatory response Only sparse chronic inflammatory cells; the surrounding stroma is otherwise normal. Metallic deposits are usually inert and provoke little or no inflammation, unlike some other foreign-body tattoos.  Normal stratified squamous epithelium Surface epithelium shows normal maturation without melanocytic hyperplasia or atypia. Rules out melanocytic lesions (e.g., melanotic macule, nevus, melanoma) in which increased basal melanin or nests/cells would be expected.  No junctional or dermal/lamina-propria proliferation of melanocytes or nevus cells Pigment is entirely extracellular or within macrophages; no nests of nevus cells. Distinguishes amalgam tattoo from melanocytic nevus or melanoma.  Taken together, the presence of dense, extracellular, black metallic particles distributed along connective-tissue structures with a benign overlying epithelium and minimal inflammation is diagnostic of an amalgam tattoo in an oral mucosal biopsy. |
| 9.12 | Diagnosis: Oral Melanotic Macule of Palatal Mucosa; Key Microscopic Features: Mild acanthosis without inflammation, abundant melanin in basal cells at the lower half and tips of rete ridges, and incontinent melanin and melanophages in the lamina propria. | Based on the provided hematoxylin and eosin (H&E) stained histological images at different magnifications, the single most likely histopathological diagnosis is:  Melanotic Macule  Key Microscopic Features Justifying the Diagnosis:  Increased Basal Layer Pigmentation:  There is prominent melanin pigmentation in the basal cell layer of the stratified squamous epithelium, visible as brown granular deposits (especially evident in the higher magnification images).  No atypia or architectural distortion is seen in the melanocytes, supporting a benign process.  Melanin Incontinence:  Melanin-laden macrophages (melanophages) are clearly present in the superficial lamina propria, indicating melanin incontinence. This is a key feature of melanotic macules.  Regular Epithelial Architecture:  The epithelium shows normal stratification and maturation, with no dysplasia or increase in melanocyte numbers in the basal layer, which helps to differentiate it from melanocytic nevi or melanoma.  Absence of Melanocytic Nests:  No nests or clusters of nevus cells are seen in the epithelium or subepithelial connective tissue, ruling out intraepithelial or junctional nevus.  Lamina Propria Composition:  The lamina propria shows fibrocollagenous connective tissue with some blood vessels and scattered inflammatory cells, but without neoplastic features.  Summary:  The features seen—namely basal hyperpigmentation, melanin incontinence, and the lack of melanocytic proliferation or atypia—are diagnostic of a melanotic macule, a benign pigmented lesion commonly found on the oral mucosa. | Histopathological diagnosis  Oral melanotic macule (focal basal-cell melanosis of the oral mucosa)  Key microscopic features visible in the three H-E photomicrographs  # Feature in the slides Diagnostic significance  1 Stratified squamous epithelium of normal thickness and architecture – no cellular atypia, dysplasia, or junctional nests Confirms a benign, non-neoplastic process rather than a melanocytic nevus or melanoma  2 Heavy brown-black pigment confined to basal and immediate suprabasal keratinocytes (seen as vertical columns of pigment along the elongated rete ridges) Represents increased melanin production by otherwise normal melanocytes – the hallmark of a melanotic macule  3 Normal number of melanocytes – pigment is inside keratinocytes; there are no junctional or dermal nests of nevus cells Rules out junctional/compound nevus  4 “Melanin incontinence” – numerous macrophages laden with coarse brown melanin granules (melanophages) scattered in the superficial lamina propria A classic accompaniment of basal melanosis in melanotic macule and physiologic (racial) pigmentation  5 Unremarkable connective tissue stroma – loosely arranged collagen, sparse chronic inflammatory cells, no foreign-body giant cells or metallic particles Excludes amalgam tattoo and other exogenous pigmentations  Together, the constellation of basal epithelial melanosis with melanin incontinence, in the absence of melanocytic proliferation or atypia, is diagnostic of an oral melanotic macule. |
| 9.13 | Diagnosis: Melanotic Macule; Key Microscopic Features: Abundant melanin present in basal cells at the tips of rete ridges, and melanin within spinous cells forming a supranuclear cap. | Histopathological Diagnosis:  Melanotic Macule (Oral Melanotic Macule)  Key Microscopic Features Supporting the Diagnosis:  Increased Basal Layer Pigmentation:  The basal cell layer of the epithelium exhibits increased melanin pigmentation. This melanin appears as brownish granules localized predominantly in the basal and suprabasal keratinocytes.  Importantly, there is no melanocytic hyperplasia or atypia.  Normal Epithelial Architecture:  The stratified squamous epithelium remains orderly without evidence of dysplasia or architectural distortion.  The rete ridges are elongated but maintain a benign configuration.  Melanin Incontinence:  Melanin pigment is observed in the superficial lamina propria, within melanophages (pigment-laden macrophages). This represents melanin incontinence, a common feature of pigmented macules.  Absence of Atypical or Proliferative Melanocytes:  There is no upward migration of melanocytes into the upper layers of the epithelium.  No nests of melanocytes or features suggesting a melanocytic neoplasm (such as lentigo maligna or melanoma) are present.  Summary:  The combination of increased basal melanin, pigment incontinence, and lack of melanocytic proliferation or atypia is characteristic of a melanotic macule, the most common benign pigmented lesion of the oral cavity. The histology is diagnostic and rules out more serious melanocytic proliferations or malignancies. | Histopathological diagnosis  Oral melanotic macule (focal oral melanin hyper-pigmentation / physiologic-racial pigmentation)  Key microscopic features that support this diagnosis  # Feature How it appears in the photographs  1 Normal-looking stratified squamous epithelium The epithelium shows normal maturation from basal to superficial layers; nuclei are uniform and there is no cytologic atypia or pagetoid spread.  2 Heavy accumulation of brown–black melanin in the basal cell layer Coarse, dark granules densely pack the cytoplasm of basal (and occasional immediate suprabasal) keratinocytes, especially at the tips and sides of rete ridges (first image).  3 Melanin incontinence with superficial melanophages Small clumps of identical pigment are seen just beneath the basement membrane in the lamina propria, inside macrophages (second, high-power image).  4 No proliferation of melanocytes / no junctional or nested activity Pigment is in keratinocytes and macrophages, not in nests of nevus cells; the number and morphology of melanocytes are unremarkable.  5 Absence of epithelial dysplasia or invasive growth Rete ridges are regular; there is no upward scatter of atypical cells, mitoses, or invasion into the connective tissue.  The combination of increased melanin pigment without an increase in melanocyte number or atypia, together with otherwise normal epithelium, is classic for an oral melanotic macule (or physiologic/racial pigmentation when the process is diffuse). |
| 9.14 | Diagnosis: Lentigo Simplex; Key Microscopic Features: Slight acanthosis, melanocytic hyperplasia, vascular ectasia, mild chronic inflammation, and slight reactive atypia in melanocytic hyperplasia. | Based on the provided hematoxylin-and-eosin (H&E) stained images at low and high magnification, the most likely histopathological diagnosis is:  Melanotic Macule (Oral Melanotic Macule)  Key Microscopic Features Supporting the Diagnosis:  Increased Melanin Pigmentation in the Basal Layer:  There is prominent melanin pigmentation within the basal keratinocytes, particularly along the basal layer of the epithelium. This is characteristic of melanotic macules.  Lack of Melanocytic Proliferation:  Importantly, there is no proliferation of melanocytes (i.e., no nests or atypical melanocytes), which helps rule out nevi and melanoma.  The melanocytes remain in their normal basal distribution without atypia or pagetoid spread.  Melanin Incontinence:  Some melanin-laden macrophages (melanophages) are visible in the superficial connective tissue (lamina propria), consistent with pigment incontinence.  Normal Stratified Squamous Epithelium:  The overlying epithelium shows regular maturation and no dysplasia.  Rete ridges appear elongated and uniform without irregularities.  Summary:  The combination of basal layer pigmentation, absence of melanocytic proliferation, and presence of melanophages in the lamina propria is diagnostic of an oral melanotic macule, a benign pigmented lesion commonly found on the lips, gingiva, or buccal mucosa. | Histopathological diagnosis (single best answer)  Labial/oral melanotic macule (simple lentigo – a focal mucosal melanosis)  Key microscopic features that support this diagnosis  Feature seen in the photomicrographs Significance for diagnosis  Dense, band-like brown-black pigment confined to the basal and immediate suprabasal keratinocytes Characteristic increase in melanin production, not a true increase in melanocyte number; gives the lesion its clinical macular pigmentation.  No junctional or dermal/lamina-propria nests of nevus cells and no pagetoid upward spread Excludes a melanocytic nevus or melanoma.  Only a mild, uniform increase in single melanocytes along the basal layer; nuclei are small, regular, and lack atypia Fits a lentiginous (simple) proliferation typical of a melanotic macule.  Slight acanthosis with elongated, club-shaped rete ridges but otherwise architecturally normal epithelium A common but non-specific accompaniment; helps distinguish it from flat ephelides or from marked papillomatosis of acanthosis nigricans.  Melanin incontinence with a few melanophages in the superficial lamina propria/dermis Frequently present in melanotic macules; explains why the pigmentation can persist.  Absent features of lichenoid interface change or dense inflammatory band Rules out pigmented lichen planus or post-inflammatory hyperpigmentation.  Taken together, the pattern of basal hyperpigmentation without atypia or nesting, minimal melanocytic hyperplasia, occasional melanophages, and otherwise unremarkable mucosal epithelium is diagnostic of a melanotic macule / simple lentigo of the oral–labial mucosa. |
| 9.21 | Diagnosis: Melanoacanthosis; Key Microscopic Features: Acanthosis and mild chronic inflammation, mild spongiosis with dendritic melanocytes throughout the epithelium, scattered lymphocytes in the lamina propria, and many melanin-laden dendritic melanocytes in the upper epithelium. | Histopathological Analysis of the Provided Images (Pigmented Lesions – Oral & Maxillofacial Pathology)  Single Most Likely Histopathological Diagnosis:  Oral Melanoacanthoma  Key Microscopic Features Justifying the Diagnosis:  1. Low Magnification (Image 1):  Acanthotic Stratified Squamous Epithelium: Noticeable thickening (acanthosis) of the epithelial layer.  Papillary Hyperplasia: The rete ridges appear elongated and irregular.  Pigmentation: Melanin pigment is focally dispersed, though less prominent at this magnification.  Underlying connective tissue appears inflamed, suggesting reactive changes.  2. Intermediate Magnification (Image 2):  Numerous Dendritic Melanocytes: Darkly pigmented cells dispersed throughout all levels of the epithelium, rather than being confined to the basal layer.  Spongiosis: Mild intercellular edema may be observed.  Chronic Inflammatory Infiltrate: In the connective tissue, consistent with a reactive or benign condition.  3. High Magnification (Image 3):  Pigmented Dendritic Melanocytes: Clearly visible melanocytes with long dendritic processes, containing melanin granules.  Lack of Cytologic Atypia: The keratinocytes and melanocytes appear benign without nuclear atypia or increased mitotic activity.  Melanin Distribution: Evidence of melanin in both keratinocytes (possibly due to melanin transfer) and free in the superficial epithelium.  Summary Justification:  The presence of benign-appearing dendritic melanocytes scattered throughout an acanthotic epithelium, absence of cytologic atypia, and reactive inflammatory stroma are classic findings in oral melanoacanthoma. This lesion is often mistaken for melanoma clinically due to its sudden appearance and pigmentation, but histologically it shows no signs of malignancy. | Histopathological diagnosis  Oral melanoacanthoma (reactive oral melano-acanthosis)  Key microscopic features that lead to this diagnosis  Level of magnification What is seen Why it points to melanoacanthoma  Low-power scan (Image 1) • Epithelium is markedly acanthotic with broad, elongated rete ridges that drop deeply into the lamina propria.  • No pushing invasive front or ulceration; basement membrane is intact. A diffuse, sheet-like epithelial hyperplasia is typical of this benign, reactive lesion.  Intermediate power (Image 2) • Within the thickened spinous layer numerous scattered, solitary pigmented cells are present at every epithelial level, not limited to the basal layer.  • Cells are dendritic/spindled to oval and lie singly between keratinocytes rather than in nests.  • Mild spongiosis; keratinocytes otherwise bland. Oral melanoacanthoma is defined by dendritic melanocytes dispersed throughout the full thickness of a hyperplastic epithelium. Lack of nests distinguishes it from a junctional/compound nevus, and the regular cytology argues against melanoma.  High power (Image 3) • The dendritic melanocytes contain coarse brown-black melanin granules; pigment is also seen within adjacent keratinocytes (pigment transfer).  • Occasional clear “empty” cytoplasmic halos represent pigment removed during processing.  • Nuclei of both melanocytes and keratinocytes show no atypia or mitotic activity. Abundant but benign pigment, coupled with the dendritic morphology and even dispersion, is characteristic. Absence of cytologic atypia supports a reactive process.  Subjacent lamina propria • Sparse chronic inflammatory cells and a few melanin-laden macrophages (“melanophages”). Pigment leakage and macrophage uptake are commonly seen beneath an oral melanoacanthoma.  Taken together, the combination of (1) pronounced epithelial acanthosis, (2) numerous benign-appearing dendritic melanocytes scattered through the entire epithelium, (3) abundant melanin pigment with transfer to keratinocytes, and (4) absence of atypia or nesting, is diagnostic of oral melanoacanthoma. |
| 9.23 | Diagnosis: Intramucosal Melanocytic Nevus; Key Microscopic Features: Heavily pigmented melanocytes near the surface with symmetry and downward maturation, and nests of epithelioid pigmented melanocytes. | Based on the provided hematoxylin and eosin (H&E) stained histological images of a pigmented lesion from the oral and maxillofacial region at low and high magnification, here is the analysis:  Single Most Likely Histopathological Diagnosis:  Benign Melanocytic Nevus (Intradermal Nevus)  Key Microscopic Features Supporting This Diagnosis:  Low Magnification (First Image):  Symmetrical, Well-Circumscribed Lesion:  The lesion shows a dome-shaped, symmetrical architecture without signs of asymmetry or infiltration.  Nesting Pattern in the Dermis/Submucosa:  The melanocytic proliferation is mostly confined to the dermis/submucosa with no involvement of the basal layer of the epithelium, consistent with an intradermal (or intramucosal) nevus.  Absence of Atypical Features:  No evidence of pagetoid spread or junctional activity, which are typical of malignant melanoma.  High Magnification (Second Image):  Nests of Nevus Cells:  Well-circumscribed nests of uniform melanocytic (nevus) cells with round to oval nuclei, small nucleoli, and minimal atypia.  Cytoplasmic Pigmentation:  Brown melanin pigment present within the cytoplasm of the nevus cells, highlighting their melanocytic origin.  Lack of Mitotic Activity or Necrosis:  There is no indication of increased mitotic figures, necrosis, or pleomorphism, which supports the benign nature of the lesion.  Conclusion:  The lesion's architectural and cytological features are consistent with a benign intradermal melanocytic nevus, commonly found in the oral mucosa. This is a non-malignant lesion and distinct from melanoma due to the absence of junctional activity, cytologic atypia, and mitotic activity. | Histopathological diagnosis (single best):  Compound (junctional + intramucosal) melanocytic nevus of the oral mucosa  Key microscopic features that support this diagnosis  Feature seen Why it points to a compound nevus  Symmetrical, well-circumscribed, dome-shaped mucosal papule on low power. The lesion is centered beneath an intact stratified squamous epithelium and tapers laterally without infiltrative edges. Benign melanocytic (“nevic”) proliferations are usually symmetric and sharply demarcated; melanoma is typically asymmetric with haphazard extension.  Nests (“theques”) of uniform melanocytic (nevus) cells at two levels – (1) along the epithelial–connective tissue junction and (2) in the superficial lamina propria. The dual location defines a compound nevus (junctional component + intramucosal component). Pure intramucosal nevi lack the junctional nests; purely junctional nevi lack the dermal component.  Progressive maturation with depth: cells in superficial nests are slightly larger and epithelioid |
| 9.24 | Diagnosis: Intramucosal Melanocytic Nevus; Key Microscopic Features: Grenz zone with superficial epithelioid cells and deeper spindle cells showing characteristic downward maturation, along with lymphocyte-like (type B) cells and spindled neuroid (type C) cells containing mature lipocytes. | Histopathological Diagnosis:  Melanoma  Key Microscopic Features Supporting the Diagnosis:  Low Magnification (First Image):  Epidermal Atypia with Pagetoid Spread:  Proliferation of atypical melanocytes extending upwards through the epithelium (pagetoid spread), a hallmark of melanoma.  Irregular junctional activity along the dermal-epidermal junction.  Asymmetry and Poor Circumscription:  The lesion lacks symmetry and is poorly circumscribed, with an irregular outline and uneven distribution of melanocytes.  Dense Cellular Infiltrate:  A dense population of atypical melanocytes and possible inflammatory infiltrate in the upper dermis and dermal-epidermal junction.  High Magnification (Second Image):  Atypical Melanocytes:  Presence of large, pleomorphic melanocytes with hyperchromatic nuclei and prominent nucleoli.  Scattered singly and in nests within the dermis.  Melanocytes in Deep Dermis and Around Adnexal Structures:  Melanocytic infiltration around adnexal structures and between collagen bundles indicates invasive melanoma.  Melanocytes infiltrating fat lobules—suggestive of vertical growth phase.  Mitotic Activity:  Frequent mitotic figures may be present (although not clearly seen here, typically evaluated at higher magnification).  Conclusion:  The architectural disorganization, presence of pagetoid spread, cytologic atypia of melanocytes, and dermal invasion strongly favor a diagnosis of melanoma, most likely mucosal melanoma given the context of oral and maxillofacial pathology. Special stains or immunohistochemistry (e.g., S100, HMB-45, Melan-A) would further confirm the melanocytic nature of the lesion. | Histopathological diagnosis  Pigmented (lichenoid) oral lichen planus  Key microscopic features visible in the provided H‑E sections  Field Salient findings How the finding supports the diagnosis  Epithelium • Acanthotic, orthokeratin/para‑keratin surface with “saw‑tooth” rete‑pegs.  • Focal vacuolar / hydropic degeneration of the basal cell layer and scattered apoptotic (Civatte) bodies. Hyperkeratosis and interface (basal‑cell) damage are classic for lichenoid mucositis / lichen planus.  Epithelial‑connective tissue interface Dense, continuous, band‑like infiltrate of small lymphocytes hugging the basement membrane (“lichenoid” pattern). The diagnostic hallmark of lichen planus is a juxta‑epithelial, band‑shaped T‑cell infiltrate.  Lamina propria • Pigment incontinence – spindle / epithelioid melanophages containing coarse brown melanin granules (gives the clinical pigmentation).  • Patchy chronic inflammatory cells extend a short distance into the superficial connective tissue; islands of normal adipose tissue are seen deeper. Melanin leakage with melanophages explains the clinical brown‑black coloration and places the lesion in the pigmented sub‑group.  Absence of atypia No cytologic atypia, pagetoid spread or destructive growth. Helps exclude melanoma or other malignant pigmented lesions.  The constellation of a hyperkeratinised epithelium, basal cell degeneration with Civatte bodies, and a dense, juxta‑epithelial lymphocytic band together with melanin incontinence is diagnostic of pigmented oral lichen planus (lichenoid mucositis with pigment incontinence). |
| 9.26 | Diagnosis: Junctional melanocytic nevus; Key Microscopic Features: Clusters of melanocytes inside the epithelium; Well-demarcated clusters of non-cancerous melanocytes, separated from surrounding keratinocytes with no melanocytes inside the lamina propria. | Histopathological Diagnosis:  Oral Melanocytic Nevus (most likely an Intramucosal Nevus)  Key Microscopic Features Supporting the Diagnosis:  Well-Circumscribed Nests of Melanocytes:  Both low- and high-power images show well-defined nests of uniform cells located within the connective tissue, particularly just beneath the epithelium. These are characteristic of nevus cells.  The cells are round to oval with uniform nuclei and inconspicuous nucleoli.  Intramucosal Location of Nests:  The melanocytic nests are located within the lamina propria (the connective tissue layer beneath the epithelium), rather than at the junction between epithelium and connective tissue. This distribution is typical of an intramucosal nevus, the most common type of oral melanocytic nevus.  Lack of Atypia or Mitoses:  The melanocytes within the nests do not exhibit cytologic atypia, mitotic activity, or pleomorphism, supporting a benign process.  No Pagetoid Spread or Junctional Activity:  There is no evidence of melanocytes migrating upward through the epithelium (as in melanoma) or proliferation at the epithelial-connective tissue junction (seen in junctional nevi or early melanoma).  Pigmentation:  Scattered brown pigment (likely melanin) can be appreciated in the nests and surrounding cells in the high-power image. Although not always present in all nevi, it supports melanocytic origin.  Summary:  These histological features are diagnostic of a benign melanocytic lesion, most consistent with an intramucosal melanocytic nevus, which is a common pigmented lesion in the oral cavity. The absence of cytologic atypia, mitoses, or invasive growth rules out melanoma. | Histopathological diagnosis  → Junctional (oral acquired) melanocytic nevus  Key microscopic features that support the diagnosis  Feature What is seen in the two H-E fields Why it points to a junctional nevus  Symmetry & circumscription At low power the lesion is horizontally symmetric and sharply set off from the adjacent uninvolved mucosa. Benign nevi are usually well-circumscribed, in contrast to the haphazard growth of melanoma.  Nests (“theques”) of melanocytic cells confined to the epithelium/epithelial–connective tissue junction Multiple round-to-oval, tightly packed nests sit along the tips and sides of the rete ridges and bridge adjacent ridges. They do not descend into the deeper lamina propria in the plane of section. Junctional nevi are defined by a proliferation of nevus cells along the basal layer; compound nevi would show an additional dermal/lamina-propria component.  Uniform nevus-cell cytology On high power each nest is composed of small-to-medium epithelioid cells with round nuclei, delicate chromatin, inconspicuous nucleoli and moderate pale cytoplasm containing fine brown melanin granules. Cytologic blandness and maturation (absence of pleomorphism or mitoses) confirm a benign nevus rather than melanoma.  Lack of pagetoid scatter or upward migration Melanocytic cells remain in cohesive nests; there is no single-cell spread through the upper epithelial layers. Pagetoid (suprabasal) spread would favour melanoma in situ.  Minimal stromal reaction The underlying lamina propria shows only delicate fibrous tissue without desmoplasia or inflammation. An exuberant stromal response would raise concern for an invasive process.  Taken together, the orderly, symmetric proliferation of cytologically bland melanocytic nests restricted to the epithelial–connective tissue interface is diagnostic of a junctional melanocytic nevus of the oral mucosa. |
| 9.27 | Diagnosis: Compound melanocytic nevus; Key Microscopic Features: Symmetric lesion with melanocytes abutting the epithelium with downward maturation; Clusters of colored non-cancerous melanocytes inside the basal cell layer. | Based on the two provided hematoxylin and eosin (H&E) stained histopathological images of the same lesion at different magnifications, from the pigmented lesions group in oral and maxillofacial pathology, the most appropriate diagnosis is:  Histopathological Diagnosis:  Oral Melanoma (Malignant Melanoma of the Oral Cavity)  Key Microscopic Features Supporting the Diagnosis:  Low Power View (First Image):  Epithelial ulceration or irregular surface contour with disruption of the normal stratified squamous epithelium.  Dense, nodular sheets and nests of atypical melanocytes infiltrating the subepithelial connective tissue.  Evidence of pigmentation (melanin deposition), seen as brown-black granular material scattered throughout the tumor nests and within macrophages.  High Power View (Second Image):  Highly pleomorphic melanocytic cells with:  Large hyperchromatic nuclei  Prominent nucleoli  Increased nuclear-to-cytoplasmic ratio  Atypical mitotic figures and nuclear pleomorphism  Melanin pigment present within tumor cells and in the stroma.  Loss of maturation with depth, characteristic of melanoma (as opposed to benign melanocytic lesions).  Infiltrative growth pattern.  Summary:  These histopathological features—particularly the presence of atypical melanocytes with abundant melanin pigment, significant cytologic atypia, and invasive growth—are highly characteristic of oral malignant melanoma, a rare but aggressive neoplasm of melanocytic origin seen in the oral mucosa. | Histopathological diagnosis  Pigmented muco-epidermoid carcinoma (intermediate-grade) of a minor salivary gland.  Key microscopic features that lead to this diagnosis  Field Observation Significance  Low-power architecture A circumscribed but infiltrative submucosal mass formed by lobules and cords separated by delicate fibrous septa, lying beneath an intact stratified squamous surface epithelium Typical overall pattern of a salivary-gland–type neoplasm rather than a surface epithelium tumour  Cellular composition Mixed population of mucous cells with vacuolated/foamy cytoplasm (appear as empty or pale lumina), intermediate basaloid cells, and epidermoid (squamoid) cells with eosinophilic cytoplasm The tri-cellular mixture is diagnostic for muco-epidermoid carcinoma  Cystic–duct-like spaces Multiple round to elongated lumina containing eosinophilic or empty mucin pools between tumour nests Cystic component is characteristic, and its proportion places the tumour in the intermediate-grade bracket  Cytological atypia Enlarged pleomorphic nuclei with conspicuous nucleoli and brisk mitotic activity, but no overt keratin pearl formation Supports an intermediate-grade malignant salivary carcinoma rather than a benign lesion  Melanin pigment Coarse brown–black granules within some tumour cells and in scattered macrophages (especially evident in the high-power image) Explains the clinical pigmentation and places the lesion in the “pigmented variants” group; colonisation of the tumour by dendritic melanocytes is a recognised but uncommon phenomenon in muco-epidermoid carcinoma  Stromal reaction Sparse chronic inflammatory infiltrate and delicate fibro-vascular stroma without bone or neural tissue Helps exclude melanotic neuroectodermal tumour of infancy (which is biphasic and shows neuroblastic cells) and malignant melanoma (which lacks the tri-cellular MEC pattern)  The simultaneous presence of mucous, epidermoid, and intermediate cells arranged in cystic and solid lobules is pathognomonic for muco-epidermoid carcinoma; the additional melanin pigmentation simply designates the rare pigmented variant rather than altering the fundamental diagnosis. |
| 9.28 | Diagnosis: Common blue nevus of palatal mucosa; Key Microscopic Features: Subtle cellular pigmentation in the lamina propria; Spindled colored dendritic melanocytes. | Histopathological Analysis of the Pigmented Lesion (H&E stain, different magnifications)  Most Likely Histopathological Diagnosis:  Oral Melanotic Macule  Key Microscopic Features Supporting the Diagnosis:  Low Magnification (First Image):  Stratified Squamous Epithelium:  The overlying epithelium is intact, stratified squamous in type, and shows no architectural distortion or dysplasia.  Basal layer appears mildly pigmented.  Increased Basal Melanin Pigmentation:  Prominent brown pigmentation along the basal cell layer without cellular atypia.  No Junctional or Dermal Nests of Melanocytes:  Absence of melanocytic proliferation in the epithelium or lamina propria rules out nevi and melanoma.  Lamina Propria:  No significant inflammatory infiltrate, but increased melanin pigment is observed.  High Magnification (Second Image):  Melanin Pigment in the Lamina Propria:  Coarse brown-black granular material (melanin) is seen free or within melanophages (pigment-laden macrophages) in the connective tissue.  No Atypical or Proliferating Melanocytes:  Important to distinguish from malignant melanoma or junctional nevus.  Normal Collagen and Fibroblastic Background:  Indicates a benign and reactive process rather than neoplastic proliferation.  Conclusion:  The images demonstrate characteristic features of an oral melanotic macule, a benign pigmented lesion commonly seen on the lip or gingiva. The key findings include increased melanin in basal keratinocytes and presence of melanin-laden macrophages in the lamina propria, without any evidence of melanocytic proliferation or atypia. | Histopathological diagnosis (single best):  Oral melanotic macule (oral focal melanosis)  Key microscopic features supporting the diagnosis  Area What is seen Diagnostic significance  Stratified squamous epithelium • Architecture essentially normal – rete ridges of usual size and shape  • Prominent brown‐black granular pigment confined to basal and immediate parabasal keratinocytes Indicates increased melanin production rather than a proliferation of melanocytes  Epithelial–connective tissue junction • Intact basement membrane  • No junctional or subepithelial nests of nevus cells or atypical melanocytes Rules out a melanocytic nevus or melanoma  Superficial lamina propria • Numerous pigment-laden macrophages (melanophages) scattered between dense, wavy collagen bundles Characteristic “pigment incontinence” that accompanies melanotic macule  Melanocytic component • Normal number and morphology of melanocytes; no upward (pagetoid) spread, no cytologic atypia, no mitoses Confirms a hyper-melanosis rather than a true melanocytic proliferation  Submucosa (hard-palate setting) • Minor salivary glands Deep to a dense collagenous lamina propria—normal background anatomy Shows the lesion is purely mucosal and localized  Summary of why this is a melanotic macule  The photographs show a flat pigmented lesion in which the amount of melanin is increased, but the number / behavior of melanocytes is not. The combination of (1) heavy basal melanin, (2) pigment incontinence to macrophages, and (3) absence of nevus-cell nests or atypia is classic for an oral melanotic macule and distinguishes it from melanocytic nevi, physiologic pigmentation, smoker’s melanosis, and, most importantly, oral melanoma. |
| 9.29 | Diagnosis: Blue nevus of palatal mucosa; Key Microscopic Features: Proliferation of spindle cells in the lamina propria; Cellular focus of colored spindled melanocytes; Spindle cells exhibit trailing cytoplasmic processes and elongated nuclei. | Histopathological Diagnosis:  Oral Melanotic Macule  Key Microscopic Features Supporting the Diagnosis:  Increased Melanin Pigmentation in the Basal Layer of Epithelium:  The epithelial basal layer shows prominent melanin pigmentation, appearing as brown granules. This is a hallmark feature of melanotic macules.  No proliferation of melanocytes is evident—melanin is increased, not the number of melanocytes.  Pigment Incontinence in the Superficial Lamina Propria:  Melanin granules are present within melanophages (pigment-laden macrophages) in the superficial connective tissue, suggesting pigment leakage (incontinence).  These melanophages appear as brown-black granules scattered within the pink collagenous stroma.  Normal Stratified Squamous Epithelium:  The epithelium appears non-dysplastic, with no architectural or cytologic atypia. The rete ridges are slightly elongated but regular.  No signs of junctional or dermal nests, which helps rule out melanocytic neoplasms like melanocytic nevus or melanoma.  Lack of Atypical or Proliferative Melanocytes:  Unlike melanoma, there is no evidence of melanocytic atypia or upward migration of melanocytes within the epithelium.  No mitotic figures or pleomorphic nuclei are noted.  Conclusion:  These features—especially the combination of increased basal melanin, pigment incontinence without melanocytic proliferation or atypia—are diagnostic of an oral melanotic macule, a benign pigmented lesion commonly seen in the oral mucosa. | Histopathological diagnosis (single best answer)  → Amalgam tattoo (localised exogenous metallic‐particle deposition)  Key microscopic features visible in the submitted H-E photomicrographs  Field Observations that support the diagnosis  Surface epithelium Stratified squamous epithelium of normal architecture and maturation; no proliferation or atypia of melanocytes or nevus cells.  Lamina propria / submucosa Numerous coarse, opaque dark-brown to black particles lying extracellularly along and between collagen bundles and occasionally within vessel walls; particles show irregular, angular or filamentous (“shard-like”) shapes rather than the fine, dusty granules of melanin.  Cellular reaction Scattered macrophages or foreign-body–type giant cells surrounding some pigment clusters, but otherwise minimal chronic inflammatory infiltrate.  Absence of melanocytic lesion No nests of nevus cells, no dendritic spindle melanocytes (rules out intramucosal nevus, blue nevus), and no junctional activity or atypia (rules out melanoma). The pigment is not confined to basal keratinocytes (rules out melanotic macule / physiologic melanosis).  Pattern and distribution Pigment follows connective-tissue fibers and small neurovascular bundles — a typical deposition pattern for amalgam or other heavy-metal debris introduced iatrogenically during dental procedures.  Together, the combination of normal oral epithelium, extracellular coarse black metallic fragments embedded in connective tissue, a scant foreign-body response, and the complete absence of melanocytic proliferation is characteristic of an amalgam tattoo. |
| 9.30 | Diagnosis: Epithelioid blue nevus within combined nevus; Key Microscopic Features: Clusters of colored melanocytes near the surface surrounded by epithelioid melanocytes; Epithelioid melanocytes are large and polyhedral with abundant melanin in their cytoplasm and benign nuclei. | Histopathological Diagnosis:  Oral Melanotic Macule  Key Microscopic Features Justifying the Diagnosis:  Low Magnification Image (First Image):  Acanthotic Stratified Squamous Epithelium:  The surface epithelium appears thickened (acanthosis) but maintains orderly maturation without dysplasia.  Increased Basal Melanin Pigmentation:  There is marked brown pigmentation in the basal layer of the epithelium, consistent with melanin.  Melanophages in the Superficial Lamina Propria:  Pigment-laden macrophages (melanophages) are visible in the connective tissue beneath the epithelium, supporting pigment incontinence.  Absence of Atypical Melanocytes:  No nests or proliferation of atypical melanocytes is observed, ruling out neoplastic melanocytic lesions such as melanoma or nevus.  High Magnification Image (Second Image):  Pigment Incontinence:  Numerous melanophages containing coarse brown melanin granules are scattered within the lamina propria.  No Cellular Atypia or Mitotic Activity:  The cells, including melanophages and adjacent stromal cells, lack atypia or abnormal mitotic figures.  Uniform Pigmentation:  The pigment is uniform and consistent with melanin rather than hemosiderin or exogenous pigments (which would have a different staining pattern or birefringence under polarized light).  Summary:  The constellation of increased basal melanin pigmentation, pigment incontinence with melanophages in the lamina propria, and absence of melanocytic proliferation or atypia supports the diagnosis of Oral Melanotic Macule—a benign, reactive pigmented lesion frequently found on the lips and oral mucosa. | Histopathological diagnosis  Oral compound melanocytic (nevocellular) nevus  Key microscopic features that support this diagnosis  Feature (seen in the images) Significance  Well-circumscribed “theque” of heavily pigmented cells immediately beneath the mucosal epithelium (large round nest in the first image) Characteristic dermal/lamina-propria nest of nevus cells found in a compound nevus.  Smaller pigmented nest located within the basal cell layer of the surface epithelium Demonstrates junctional activity — required, together with a dermal nest, to label the lesion compound.  Nevus cells: polygonal/epithelioid, bland round nuclei, finely granular brown melanin, arranged in cohesive nests Typical cytology of melanocytic nevus; absence of atypia or mitoses distinguishes it from melanoma.  Maturation with depth: in deeper connective tissue the cells become smaller, less pigmented and disperse singly among collagen bundles (second image) Progressive maturation is a hallmark of benign nevi.  Numerous melanophages (macrophages stuffed with coarse brown pigment) scattered in the lamina propria (prominent in the high-power image) Common reactive accompaniment of oral nevi, helping to explain the clinical pigmentation.  No asymmetric growth, no pagetoid upward spread, no cytologic atypia or abnormal mitotic figures Excludes malignant melanoma and other aggressive pigmented lesions.  These combined findings—junctional nests, a subjacent pigmented dermal theque, orderly maturation, and bland cytology—are diagnostic of a compound melanocytic nevus arising in the oral mucosa. |
| 9.31 | Diagnosis: Combined melanocytic nevus; Key Microscopic Features: Sheets of melanocytes with sclerosis and no typical downward maturation; Sheets of colored epithelioid cells; Colored spindle and epithelioid melanocytes. | ed on the histopathological evaluation of the provided hematoxylin and eosin (H&E) stained sections of a pigmented lesion in the oral cavity (presented at low, intermediate, and high magnification), the single most likely histopathological diagnosis is:  Melanotic Neuroectodermal Tumor of Infancy (MNTI)  Key Microscopic Features Supporting the Diagnosis:  Biphasic Cell Population:  Seen particularly well in the medium- and high-power images.  Composed of:  Large epithelioid cells with abundant eosinophilic to clear cytoplasm and vesicular nuclei — these cells often contain brown melanin pigment, confirming melanocytic differentiation.  Smaller basophilic neuroblast-like cells with hyperchromatic nuclei and scant cytoplasm — often arranged in nests or alveolar patterns.  Pigmentation:  Presence of melanin pigment is prominent in the epithelioid cell component, evident in both intermediate and high magnification images. This is a hallmark of melanotic tumors and supports the diagnosis of MNTI.  Alveolar and Nesting Growth Pattern:  The low-power image shows lobulated nests and alveolar architecture of tumor islands within the connective tissue stroma, with a pushing border but relatively circumscribed margins — typical of MNTI.  Fibrous Stroma and Infiltrative Pattern:  The tumor is seen embedded in fibrous connective tissue stroma and is not confined to the superficial layers, again consistent with MNTI’s locally aggressive behavior despite being a benign neoplasm.  Summary:  The combination of melanin-producing large epithelioid cells, small neuroblast-like cells, and a distinct biphasic pattern in a young patient strongly favors the diagnosis of Melanotic Neuroectodermal Tumor of Infancy (MNTI) — a rare, pigmented tumor typically found in the anterior maxilla of infants. | Histopathological diagnosis  → Cellular blue nevus (of the oral mucosa)  Key microscopic features supporting the diagnosis  Level Findings seen in the photomicrographs Significance  Low-power (whole-section view) • Well-circumscribed, lobulated lesion entirely within the lamina propria/deeper connective tissue, separated from the overlying stratified-squamous epithelium by a narrow, uninvolved “grenz zone”.  • Multiple nodules of densely basophilic (blue-black) tissue; intervening stroma is fibrous/collagenous. A dermal-based melanocytic proliferation with no junctional (intra-epithelial) component is typical of a blue nevus, especially its cellular variant.  Intermediate magnification • Sheets, nests and short fascicles of spindle and plump epithelioid melanocytes.  • Cells show abundant brown granular melanin pigment; numerous pigment-laden macrophages (melanophages) are scattered between them. A biphasic pattern (dendritic spindle cells + larger epithelioid cells) and heavy melanin deposition characterise the cellular blue nevus.  High magnification • Spindle cells with elongated, finely tapered nuclei and bland chromatin, arranged in storiform/whorled and streaming patterns tightly interwoven with sclerotic collagen bundles.  • Plumper epithelioid cells in solid nests, again heavily pigmented.  • Minimal cytologic atypia and rare or absent mitoses. The whorled/storiform architecture in a collagenous stroma, together with bland cytology and low mitotic activity, distinguishes a benign cellular blue nevus from malignant melanoma or other pigmented lesions.  Why other entities are less likely  Intramucosal (compound) nevus – would show nests of nevus cells at the epithelial-connective tissue junction and superficial lamina propria, often with less pigment.  Pigmented spindle cell/“Reed” nevus – is junctional and confined to the superficial mucosa with vertically oriented spindle cells.  Oral melanoma – usually exhibits marked cytologic atypia, mitotic figures (often atypical), ulceration or junctional spread, none of which are evident here.  Taken together, the dermal-based, heavily pigmented, biphasic spindle/epithelioid proliferation with whorled architecture and bland cytology is diagnostic of a cellular blue nevus. |
| 9.33 | Diagnosis: Atypical melanocytic proliferation melanoma in situ; Key Microscopic Features: Acanthosis with multiple clusters of atypical pigmented melanocytes inside the epithelium; Pigmented melanocytes with cytologic atypia inside the epithelium. | Histopathological Diagnosis:  Oral Melanoacanthoma  Key Microscopic Features Supporting the Diagnosis:  Acanthosis and Spongiosis:  The epithelium shows marked acanthosis (thickening of the spinous layer) and areas of spongiosis, which are hallmarks of a reactive lesion.  Proliferation of Dendritic Melanocytes:  Scattered numerous dendritic melanocytes (darkly pigmented cells with dendritic processes) are interspersed among the keratinocytes, extending throughout the full thickness of the epithelium.  These melanocytes are more prominent at higher magnification (as shown in the second image).  Lack of Cytologic Atypia:  Despite the hyperplastic appearance, there is no significant cellular atypia or mitotic activity in either the keratinocytes or melanocytes, which helps distinguish this from malignant melanocytic lesions.  Basal Layer Integrity:  The basement membrane remains intact, and there is no invasion into the underlying connective tissue, further supporting the benign/reactive nature.  Inflammatory Infiltrate:  Mild subepithelial chronic inflammation is sometimes noted, consistent with a reactive pattern.  Summary:  Oral melanoacanthoma is a benign, reactive pigmented lesion most commonly seen in the buccal mucosa of younger individuals, often African descent. Histologically, it is characterized by acanthosis with a striking increase in dendritic melanocytes scattered throughout the epithelium, lacking atypia or invasion. These findings are clearly visualized in your submitted hematoxylin and eosin-stained sections. | Histopathological diagnosis (single best answer)  Acantholytic (adenoid / pseudoglandular) variant of squamous cell carcinoma of the oral mucosa.  Key microscopic features that point to this diagnosis  Feature seen on the slides Significance  Down-growing, jagged tongues and nests of epithelium breaching the epithelial–connective tissue interface Confirms invasive squamous carcinoma rather than an intra-epithelial process.  Numerous round-to-oval “gland-like” or cribriform spaces within the tumour nests These are pseudoglandular cavities produced by loss of cohesion (acantholysis) not true gland formation.  Acantholysis of tumour cells – cells within the nests have separated, leaving empty or erythrocyte-filled lumina Hallmark of the acantholytic/adenoid SCC variant; distinguishes it from adenocarcinoma or mucoepidermoid carcinoma (which would contain mucin).  Malignant squamoid cytology – polygonal cells with hyperchromatic, pleomorphic nuclei, occasional intercellular bridges, and brisk mitotic activity Shows the tumour is of squamous epithelial origin.  Foci of individual-cell and light eosinophilic (dyskeratotic) keratinisation at the periphery of some nests Provides additional evidence of squamous differentiation.  Fibro-desmoplastic stroma around invasive islands Typical stromal reaction to an invasive carcinoma.  Together, the combination of invasive squamous morphology plus widespread acantholysis creating multiple pseudoglandular/cribriform spaces is diagnostic of the acantholytic (adenoid) variant of squamous cell carcinoma. |
| 9.34 | Diagnosis: Melanoma arising from atypical melanocytic hyperplasia; Key Microscopic Features: Atypical melanocytic proliferation within the epithelium; Atypical melanocytes with pleomorphic nuclei inside the epithelium; Atypical colored spindle melanocytes in the lamina propria. | Histopathological Diagnosis:  Oral Melanotic Macule  Key Microscopic Features Supporting the Diagnosis:  Increased Melanin Pigmentation in the Basal Layer:  In all magnification levels, especially the higher ones, there is prominent brown melanin pigment localized primarily in the basal and parabasal keratinocytes, consistent with increased melanogenesis rather than melanocyte proliferation.  No Atypical Melanocytic Proliferation:  There is no evidence of nests of melanocytes, upward (pagetoid) migration, or atypical nuclear features. This rules out malignant melanoma or melanocytic nevus.  Melanophages in the Lamina Propria:  Numerous melanin-laden macrophages (melanophages) are seen in the superficial connective tissue, indicating pigment incontinence—common in melanotic macules.  Normal Epithelial Architecture:  The overlying stratified squamous epithelium appears normal in thickness with regular maturation and no dysplasia, which helps differentiate it from pigmented lesions with epithelial atypia (e.g., melanoma or pigmented squamous cell carcinoma).  Conclusion:  The combination of increased basal melanin, presence of melanophages, and lack of melanocytic atypia or architectural disarray supports the diagnosis of an oral melanotic macule, a benign and common pigmented lesion in the oral cavity. | Histopathological diagnosis  Pigmented oral lichen-planus / lichenoid mucositis (pigmented variant).  Key microscopic features that lead to this diagnosis  Area Findings Diagnostic significance  Surface epithelium (low power) • Stratified squamous mucosa showing elongated, pointed “saw-tooth” rete ridges  • Overall acanthosis and focal hyper-orthokeratosis Characteristic architectural change of oral lichen planus  Basal layer • Hydropic (liquefactive) degeneration of basal keratinocytes  • Numerous Civatte (colloid) bodies Hallmark cytopathic effect of lichenoid reaction patterns  Subepithelial band • Dense, band-like, mostly lymphocytic infiltrate hugging the epithelial–connective-tissue interface Defines a lichenoid interface mucositis  Pigmentation • Marked melanin incontinence: coarse brown granules freely scattered in the superficial lamina propria and packed inside macrophages (melanophages) Explains the clinical brown/black discoloration and places the lesion in the “pigmented” category  Absence of melanocytic nests / atypia No junctional, compound or invasive nests; no cytological atypia of melanocytes Excludes nevus or malignant melanoma  Taken together, the interface lichenoid pattern with basal cell damage, Civatte bodies, band-like lymphocytic infiltrate and prominent melanin incontinence is diagnostic of pigmented oral lichen planus / a pigmented lichenoid lesion rather than a primary melanocytic proliferation. |
| 9.35 | Diagnosis: Amelanotic nodular melanoma; Key Microscopic Features: Sheets of atypical melanocytes with overlying ulcer; Atypical melanocytes inside the epithelium; Large polyhedral cells with eccentric nuclei and abundant cytoplasm in a pseudoglandular pattern, lacking melanin. | Based on the histopathological evaluation of the three provided hematoxylin and eosin (H&E) stained images at increasing magnifications, the most likely diagnosis is:  Malignant Melanoma (Amelanotic variant)  Key Microscopic Features Supporting the Diagnosis:  Overall Architecture (Low Magnification - First Image):  Nodular growth pattern beneath the epithelium.  Dense cellular proliferation extending into the underlying connective tissue.  Disruption or effacement of normal mucosal architecture.  Intermediate Magnification (Second Image):  Epithelial ulceration and pagetoid spread (melanocytes migrating upwards into the epithelium), though subtle due to the amelanotic nature.  Atypical melanocytic proliferation in the subepithelial tissue.  Cells appear epithelioid with a high nuclear-to-cytoplasmic ratio, prominent nucleoli, and nuclear pleomorphism.  High Magnification (Third Image):  Marked cytologic atypia: pleomorphic, hyperchromatic nuclei, and prominent eosinophilic nucleoli.  Frequent mitotic figures, including atypical forms.  Absence of melanin pigmentation — characteristic of the amelanotic variant.  Occasional vascular invasion and discohesive cell nests.  Additional Notes:  The absence of pigment can make amelanotic melanoma challenging to diagnose; however, the cytological atypia, growth pattern, and epidermal changes are key clues.  Immunohistochemical stains (e.g., S100, HMB-45, Melan-A) are crucial to confirm melanocytic origin, especially in amelanotic cases. | Histopathological diagnosis (single best fit)  Primary oral mucosal malignant melanoma – amelanotic type  Key microscopic features that point to this diagnosis  Level Salient findings Diagnostic significance  Surface epithelium • Irregular/ulcerated stratified squamous epithelium  • Proliferation of atypical melanocytes along the basal layer with pagetoid upward scatter Demonstrates the required “junctional activity” of a primary mucosal melanoma rather than a metastasis or purely submucosal tumour  Superficial lamina propria • Dense, sheet-like and nested infiltrate immediately beneath the epithelium  • Foci of clear-to-empty spaces where tumour has lifted away → alveolar/loosely nested pattern Matches the growth pattern typical of mucosal melanoma; separates it from benign nevi, which remain well circumscribed and lack destructive infiltration  Tumour cells (high power) • Predominantly epithelioid cells with moderate eosinophilic to clear cytoplasm  • Marked nuclear pleomorphism, vesicular chromatin and conspicuous nucleoli  • Frequent mitotic figures  • Pigment largely absent or scant (amelanotic) The cytologic atypia and mitotic activity confirm malignancy; the paucity of melanin explains the clinically “non-pigmented” (amelanotic) presentation while still falling within the pigmented-lesion category histogenetically  Stroma / architecture • Thin fibrovascular septa outlining alveolar nests  • No true glandular differentiation, ruling out metastatic carcinomas or salivary tumours Supports a melanocytic rather than epithelial origin  Taken together—junctional atypical melanocytes with pagetoid spread, an underlying destructive sheet/nest of pleomorphic epithelioid cells, an alveolar growth pattern, brisk mitoses, and little to no melanin—the lesion fulfils the classic criteria for an amelanotic malignant melanoma of the oral mucosa. |
| 9.36 | Diagnosis: Spindle and epithelioid gingival melanoma; Key Microscopic Features: Sheets of spindle cells with a fasciculated growth pattern and abundant melanin; Atypical melanocytes inside the epithelium; Epithelioid and a few spindle melanocytes with pleomorphism, large nuclei, prominent nucleoli, and abundant melanin. | Based on the analysis of the provided hematoxylin and eosin (H&E) stained images at different magnifications, the most likely histopathological diagnosis is:  Malignant Melanoma (Amelanotic or Pigmented Type)  Key Microscopic Features Supporting the Diagnosis:  1. Spindle and Epithelioid Cell Morphology (Image 1):  The low-power view shows a densely cellular lesion with intersecting fascicles and storiform patterns of spindle-shaped cells.  There is marked cellular atypia with pleomorphism.  The architecture lacks maturation with depth, a hallmark of malignancy.  2. Presence of Melanin Pigment (Images 2 & 3):  Brown-black intracellular pigment is clearly visible in several tumor cells, consistent with melanin.  Melanin is variably distributed among atypical epithelioid cells.  3. Nuclear Pleomorphism and Mitoses (Image 3):  At high magnification, cells demonstrate:  Large, pleomorphic, hyperchromatic nuclei  Prominent nucleoli  Atypical mitotic figures  These features support a high-grade malignant process.  4. Infiltrative Growth Pattern:  Tumor infiltrates into surrounding tissue, without a clear demarcation, supporting invasive malignancy.  Additional Comments:  Although other pigmented lesions (e.g., pigmented spindle cell nevus, melanotic neuroectodermal tumor of infancy, or pigmented basal cell carcinoma) could be considered, the combination of melanin pigment, cellular atypia, pleomorphism, and mitotic activity makes malignant melanoma the most consistent diagnosis.  Immunohistochemical staining for S100, HMB-45, and Melan-A would aid in confirming melanocytic origin. | Histopathological diagnosis  → Invasive mucosal malignant melanoma of the oral cavity (predominantly spindle-cell / mixed spindle- and epithelioid-cell pattern).  Key microscopic features visible in the three H-E sections  Field Salient findings Value for the diagnosis  Overall architecture (low-power, image 1) Dense, highly cellular, infiltrative mass beneath the mucosal epithelium, arranged in sweeping interlacing fascicles and whorls of fusiform cells; no lobular or encapsulated border. Rules out benign nevus or melanotic macule; supports an aggressive melanocytic neoplasm.  Cell morphology (images 2 & 3) Mixed cell population:  • Spindle cells with elongated, blunt-ended (“cigar-shaped”) nuclei forming fascicles.  • Epithelioid/pleomorphic cells with vesicular nuclei, prominent eosinophilic nucleoli, ample cytoplasm.  Marked nuclear pleomorphism and crowding, frequent atypical mitoses. Cytologic atypia and mitotic activity are hallmarks of malignancy; the mixed spindle/epithelioid phenotype is classic for mucosal melanoma.  Pigment Coarse, dark-brown to black melanin granules within many tumour cells and scattered melanophages (seen in all images, especially 2 & 3). Confirms a melanocytic lesion and differentiates from non-melanocytic spindle-cell sarcomas.  Junctional activity / pagetoid spread (image 2) Atypical pigmented cells extend into and above the basal layer of the overlying stratified squamous epithelium. Diagnostic of melanoma, proving the tumour originates from mucosal melanocytes rather than a deeper soft-tissue spindle-cell tumour.  Invasion pattern Destructive infiltration of the lamina propria and deeper connective tissue without maturation or zonation; no organized nesting or maturation with depth. Consistent with an invasive malignant process rather than a benign nevus.  Together, these features (high cellularity, spindle-plus-epithelioid atypical melanocytes, abundant melanin pigment, pagetoid/junctional spread, and numerous atypical mitoses) are diagnostic of invasive oral malignant melanoma rather than other pigmented spindle-cell tumours (e.g., melanotic schwannoma, pigmented neurofibroma) or benign melanocytic proliferations. |
| 9.38 | Diagnosis: Melanotic Neuroectodermal Tumor of Infancy; Key Microscopic Features: Irregular tumor cell islands with fibrous stroma, along with biphasic tumor cell islands surrounded by fibrous tissue. Larger epithelioid tumor cells with pale cytoplasm and large, pale nuclei containing dusty melanin surround smaller dark-nucleated cells. | Histopathological Analysis of Pigmented Lesion (Oral and Maxillofacial Pathology)  Diagnosis:  Malignant Melanoma  Key Microscopic Features Supporting the Diagnosis:  1. High-Power Magnification (3rd Image):  Atypical melanocytes with nuclear pleomorphism, prominent nucleoli, and high nuclear-to-cytoplasmic ratio.  Intraepithelial pagetoid spread: Melanocytes are observed migrating upward into the epithelium.  Presence of brown-black melanin pigment within the tumor cells and surrounding macrophages (melanophages), a hallmark of melanocytic lesions.  Mitotic figures may be visible, indicating high proliferative activity.  2. Intermediate Magnification (2nd Image):  Nests and sheets of malignant melanocytes with irregular architecture, occupying both epithelial and subepithelial compartments.  Vascular invasion and desmoplasia may be present (harder to discern without IHC but can be suspected).  Melanin pigment is visible within tumor nests.  3. Low-Power Magnification (1st Image):  Diffuse and infiltrative growth pattern across the connective tissue.  Disruption of normal mucosal architecture, replaced by neoplastic melanocytic proliferation.  Melanin pigmentation is evident as scattered dark areas.  Summary:  These features — notably the presence of atypical melanocytes, pigment production, pagetoid spread, and infiltrative growth — are diagnostic of oral malignant melanoma. Given its rarity and aggressive nature in the oral cavity, recognition of these histological characteristics is critical.  If you need immunohistochemical confirmation or differentials (e.g., pigmented basal cell carcin | Histopathological diagnosis – Melanotic neuroectodermal tumor of infancy (MNTI)  ( synonyms: retinal anlage tumor, melanotic progonoblastoma )  Key microscopic features supporting the diagnosis  Field & magnification What is seen Significance  Low-power (first two images) • Tumor forms lobulated/alveolar nests and trabeculae separated by a collagen-rich stroma.  • Nests often have a central pale zone and a darker peripheral rim. Classic pseudo-alveolar architecture of MNTI.  Intermediate–high power (all images, esp. third) Biphasic cell population:  1. Peripheral large polygonal epithelioid cells with abundant granular cytoplasm that contains coarse brown–black pigment (melanin).  2. Central small round “neuroblastic” cells with high N : C ratio, scant cytoplasm and round hyperchromatic nuclei. The coexistence of melanin-laden epithelial-like cells and primitive neuroectodermal cells is pathognomonic.  Pigment is intracellular (within large cells) and sometimes extracellular/melanophages in the stroma. Confirms melanin production, explaining the gross pigmentation of the lesion.  Occasional tubule- or duct-like spaces with luminal secretion. Frequently encountered in MNTI due to primitive tubular differentiation.  Mitotic activity is low to moderate; no significant pleomorphism or necrosis. Supports the typical benign—but locally aggressive—nature of MNTI.  The combination of an alveolar pattern, a sharp biphasic cellular composition, and abundant melanin pigment in a lesion from the oral/maxillofacial region of an infant is quintessential for melanotic neuroectodermal tumor of infancy and excludes other pigmented odontogenic, melanocytic, or neuroectodermal lesions. |
| 9.39 | Diagnosis: Melanotic Neuroectodermal Tumor of Infancy; Key Microscopic Features: A small blue cell tumor extensively infiltrating bone, with a biphasic population of darkly staining neuroblast-like cells and epithelioid cells containing melanin. | Based on the two provided hematoxylin and eosin (H&E) stained images of a pigmented lesion in the oral and maxillofacial region at different magnifications, the single most likely histopathological diagnosis is:  Malignant Melanoma  Key Microscopic Features Supporting the Diagnosis:  Low Magnification (Image 1):  Atypical cellular proliferation arranged in irregular nests and sheets, infiltrating the connective tissue.  Pigmented cells visible within the tumor nests, consistent with melanin production.  Lack of normal architecture, with disruption of the mucosal connective tissue structure.  High Magnification (Image 2):  Highly pleomorphic melanocytic cells – enlarged, hyperchromatic nuclei with prominent nucleoli.  Intracytoplasmic melanin pigment – brown-black granules within the tumor cells.  High mitotic activity, with abnormal mitotic figures possibly seen.  Tumor cells infiltrating the fibrous stroma in a disorganized pattern.  Conclusion:  The combination of atypical melanocytes containing melanin, nuclear pleomorphism, mitotic activity, and invasive growth pattern are classic histologic features of oral malignant melanoma. This diagnosis carries a poor prognosis and requires urgent oncologic management. | Most likely histopathological diagnosis  ▪ Primary malignant melanoma of the oral cavity (invasive, pigmented type)  Key microscopic features that point to this diagnosis  Field Salient findings Diagnostic relevance  Low-power view (first image) • Irregular, anastomosing trabeculae of pre-existing lamellar bone cut by broad “marrow-like” spaces that are packed with sheets and nests of cells.  • The bony trabeculae show osteocytic lacunae, confirming that the tumour is secondarily invading native alveolar bone. Deep, destructive invasion (including bone) is typical of oral mucosal melanoma and helps distinguish it from benign melanocytic proliferations.  High-power view (second image) • In the connective-tissue stroma the infiltrate is composed of markedly pleomorphic epithelioid and spindle cells with large hyperchromatic nuclei, prominent nucleoli and numerous atypical mitoses.  • Many tumour cells – and some engulfing macrophages – contain coarse, golden-brown/black granular pigment consistent with melanin.  • Pigment is unevenly distributed; some cells are amelanotic, a common feature in mucosal melanoma.  • Tumour cells dissect between collagen bundles and surround small vascular spaces (“angiotropism”). The combination of marked cytologic atypia, abundant (but irregular) melanin pigment and an invasive growth pattern is diagnostic of malignant melanoma.  Additional diagnostic clues visible in both images • Absence of an overlying surface epithelium that could give rise to a pigmented squamous lesion.  • Lack of keratin production, ducts, or calcified matrices that would suggest other pigmented odontogenic or salivary tumours.  • Presence of melanophages laden with melanin, supporting a melanocytic lesion. Helps rule out mimics such as pigmented odontogenic tumours, clear-cell carcinomas, or vascular lesions.  Together these features fulfil the histologic criteria for oral mucosal malignant melanoma: an invasive proliferation of atypical melanocytes, variably pigmented, showing destructive infiltration of supporting tissues including bone. |

REACTIVE KERATOTIC LESIONS (NONLEUKOPLAKIAS)

| **Fig_no** | **Ground truth** | **GPT 4o answer** | **o3 answer** |
| --- | --- | --- | --- |
| 10.22 | Diagnosis: Hairy Tongue; Key Microscopic Features: The condition shows hyperplastic filiform papillae at low magnification, with spires of parakeratin and numerous bacterial colonies at higher magnification. | Histopathological Analysis of Provided H&E Images  Single Most Likely Histopathological Diagnosis:  Epulis Fissuratum  Key Microscopic Features Justifying the Diagnosis:  Hyperplastic Stratified Squamous Epithelium:  Marked epithelial hyperplasia with elongated rete ridges.  The overlying epithelium appears acanthotic (thickened) and may show hyperkeratosis (increased keratin layer), typical of chronic mechanical irritation.  Hyperkeratosis and Parakeratosis:  The keratin surface is thickened and irregular, consistent with chronic irritation from a denture flange.  Underlying Connective Tissue:  The fibrous connective tissue is dense and collagenized with numerous fibroblasts.  Scattered chronic inflammatory infiltrates are present in the stroma, especially in the subepithelial region.  Lamina Propria Changes:  Shows fibrosis with thickened collagen bundles.  Mild to moderate lymphoplasmacytic infiltrate may be observed, indicating chronic inflammation.  Absence of Dysplasia:  Importantly, the epithelium lacks cytologic atypia or architectural distortion that would suggest dysplasia or malignancy.  Correlated Clinical Context:  Epulis fissuratum is a benign reactive lesion typically caused by chronic irritation from ill-fitting dentures, usually seen in the vestibular mucosa of denture-wearers. The histology shown fits well with a lesion under the "Reactive Keratotic Lesions (Nonleukoplakias)" group, confirming the diagnosis. | Histopathological diagnosis  Verruca vulgaris (common wart) – an HPV-induced, hyperkeratotic, verruciform epithelial proliferation that often presents intra-orally on the lip, palate, or gingiva and is listed among the keratotic “non-leukoplakia” reactive/viral lesions.  Key microscopic features in the provided H-E sections  Feature seen on the slides Diagnostic significance  Exophytic, papillary/filiform architecture – numerous slender, finger-like projections of epithelium covered by thick keratin Creates the clinically “verrucous” surface that is characteristic of a wart  Massive ortho- & parakeratotic layer forming long, shaggy keratin spicules Explains the white, rough surface and is typical of verruca vulgaris rather than a simple frictional keratosis  Prominent granular cell layer with coarse keratohyalin granules (best appreciated at higher power along the sides of the rete ridges) Classic microscopic hallmark of an HPV-related wart  Elongated rete ridges that dip deeply and often bend toward the centre of each papillary unit (“cupping” pattern) Helps distinguish verruca vulgaris from a squamous papilloma, whose rete ridges remain short and blunt  Occasional koilocytic change in the upper spinous layer (perinuclear cytoplasmic clearing with pyknotic nuclei) Cytopathic effect of low-risk HPV types (2, 4, 40, etc.) that drive verruca vulgaris  Minimal, superficial chronic inflammatory infiltrate in the underlying connective tissue Favouring a benign, reactive/viral rather than dysplastic or neoplastic process  The combination of a papillary/verruciform architecture, exuberant hyper- and parakeratosis with a thick granular layer, converging elongated rete ridges, and focal koilocytosis is diagnostic of verruca vulgaris and separates it from other reactive keratotic entities such as frictional (traumatic) keratosis, smokeless-tobacco keratosis, or nicotine stomatitis. |

LEUKOPLAKIA, ERYTHROPLAKIA, ORAL DYSPLASIA, AND SQUAMOUS CELL CARCINOMA

| **Fig_no** | **Ground truth** | **GPT 4o answer** | **o3 answer** |
| --- | --- | --- | --- |
| 11.7 | Diagnosis: Atypical verrucous hyperplasia with mild to no dysplasia; Key Microscopic Features: Hyperkeratosis, hypergranulosis, and verrucous epithelial proliferation showing sharp demarcation from surrounding normal epithelium; note early, thinly keratotic area sandwiched by markedly hyperkeratotic areas with keratin thickness similar to epithelial thickness. Abrupt keratinization corresponding to sharp demarcation clinically. Mild epithelial dysplasia. | Based on the provided H&E-stained histological images of an oral mucosal lesion at low, intermediate, and high magnifications, the single most likely histopathological diagnosis is:  Severe Epithelial Dysplasia / Carcinoma In Situ (CIS)  Key Microscopic Features Supporting This Diagnosis:  1. Architectural Disturbances:  Loss of epithelial maturation: Normal stratification is disrupted across the full thickness of the epithelium.  Bulbous and irregular rete ridges: There is elongation and broadening, suggesting proliferative activity.  Surface hyperkeratosis and parakeratosis: Seen as thickened keratin layers in upper magnification.  Epithelial thickening (acanthosis): Suggestive of dysplastic changes.  2. Cytologic Atypia:  Nuclear pleomorphism: Variation in nuclear size and shape is prominent.  Increased nuclear-to-cytoplasmic (N:C) ratio: A hallmark of high-grade dysplasia.  Nuclear hyperchromasia: Deeply staining nuclei are frequent.  Prominent nucleoli: Reflecting increased metabolic activity.  Increased and atypical mitotic figures: Especially in upper epithelial layers, which is abnormal.  3. Basement Membrane Intact:  Despite the severe atypia, the basement membrane is intact, with no evidence of stromal invasion, which rules out invasive squamous cell carcinoma and supports a diagnosis of carcinoma in situ.  Summary:  The combination of full-thickness cytologic atypia, architectural disorganization, and the absence of stromal invasion places this lesion in the category of severe epithelial dysplasia or carcinoma in situ, which is a precursor to invasive squamous cell carcinoma in the oral cavity.  If you have clinical correlation or immunohistochemical stains (like p53 or Ki-67), that could further reinforce the diagnosis, but histologically, this is consistent with severe dysplasia/CIS. | Histopathological diagnosis (single best):  Severe oral epithelial dysplasia (high-grade intra-epithelial neoplasia / carcinoma-in-situ)  Key microscopic features that support this diagnosis  Level of magnification Observations Significance  Low-power overview (first image) • Marked surface hyper- and parakeratosis forming an irregular, verruciform white plaque  • Pronounced epithelial thickening with long, bulbous, “drop-shaped” rete ridges  • Sharp interface with the lamina propria – no epithelial nests breaking through the basement membrane Indicates a keratotic leukoplakic lesion and rules out stromal invasion (i.e., not yet an invasive SCC)  Intermediate power (second image) • Basal-cell hyperplasia with loss of the normal maturation gradient  • Irregular, saw-toothed rete pegs and focal epithelial projections growing downward  • Numerous suprabasal mitotic figures, some atypical Architectural abnormalities typical of high-grade dysplasia  High power (third image) • Marked cytologic atypia: enlarged, pleomorphic, hyperchromatic nuclei; increased N:C ratio; irregular nuclear contours; prominent nucleoli  • Loss of nuclear polarity and cellular crowding extending into the middle and upper thirds of the epithelium  • Dyskeratosis and early keratin-pearl formation entirely within the epithelium  • Occasional atypical mitoses above the basal layer Cytologic criteria that place the lesion in the severe spectrum (sometimes termed carcinoma-in-situ) while the intact basement membrane confirms it is not yet invasive  Synthesis  The combination of pronounced architectural disturbance (elongated/bulbous rete ridges, surface corrugation, disordered maturation) and widespread cytologic atypia involving almost the full epithelial thickness, together with suprabasal and atypical mitoses and intra-epithelial keratin pearls, meets all major criteria for severe epithelial dysplasia / carcinoma-in-situ.  Because the basement membrane remains intact and no invasive epithelial nests are present in the lamina propria, an invasive squamous-cell carcinoma is excluded. |
| 11.8 | Diagnosis: Atypical verrucous hyperplasia; Key Microscopic Features: There is endophytic growth and lymphocytes are present beneath the verrucous hyperplasia but not at the edge where there is normal mucosa. Rete ridges are bulbous but epithelium overlying fibrovascular cores is attenuated. There is minimal cytologic dysplasia. | Based on the provided hematoxylin and eosin (H&E) stained histological images at increasing magnifications, the single most appropriate histopathological diagnosis is:  Oral Epithelial Dysplasia (Moderate to Severe)  Key Microscopic Features Justifying the Diagnosis:  1. Architectural Disturbances:  Irregular epithelial stratification: Loss of normal maturation pattern across the epithelial thickness.  Bulbous and elongated rete ridges: Pronounced downward projections of rete pegs, some with a drop-shaped appearance.  Basal cell hyperplasia: Expansion of the basal/parabasal layers.  2. Cytological Atypia:  Nuclear pleomorphism: Variation in nuclear size and shape, particularly noticeable at higher magnification.  Increased nuclear-to-cytoplasmic ratio: Seen throughout the lower and middle epithelial layers.  Prominent nucleoli: Occasionally visible, indicating active transcription.  Increased and abnormal mitotic figures: Some mitoses are seen above the basal layer, which is atypical.  3. Surface Changes:  Parakeratosis: Retention of nuclei in the stratum corneum.  Focal hyperkeratosis and acanthosis: Thickening of the surface epithelium in some areas.  4. Inflammatory Infiltrate:  A dense lymphocytic infiltrate in the underlying lamina propria, suggesting a chronic inflammatory response.  Exclusion of Other Diagnoses:  Leukoplakia and erythroplakia are clinical terms, not histologic diagnoses.  Squamous Cell Carcinoma is excluded due to the lack of invasive epithelial islands breaching the basement membrane in the sections provided.  No evidence of keratin pearls or deep stromal invasion that would suggest carcinoma.  Summary:  The histological features most closely align with oral epithelial dysplasia, likely moderate to severe based on the architectural and cytological changes seen across a significant portion of the epithelial thickness, without evidence of invasion. Close clinical-pathologic correlation and follow-up are recommended due to the premalignant potential. | Histopathologic diagnosis (single best answer)  ‣ Oral epithelial dysplasia – mild / low-grade (leukoplakia with low-grade dysplasia)  Key microscopic features that point to this diagnosis  Feature Appearance in the submitted sections Significance  Pronounced surface hyper-parakeratosis ± focal orthokeratin plugs Compact pink keratin layer with retained pyknotic nuclei; keratin fills the crests of the rete pegs Explains the clinical “white patch” (leukoplakia) and indicates chronic epithelial irritation  Acanthosis with elongated, bulbous/saw-tooth rete ridges Epithelium is several cell layers thicker than normal; rete ridges are narrow and drop-shaped, extending deeply into the lamina propria Architectural alteration typical of dysplasia (but still orderly maturation toward the surface)  Basal-parabasal hyperplasia & crowding Basal and immediate suprabasal cells are 3-4 layers thick, nuclei are crowded and slightly enlarged Early dysplastic change; expansion is limited to the lower third of the epithelium, consistent with mild grade  Cytologic atypia (mild) Mild nuclear enlargement, slight hyperchromasia, occasional nucleoli, but nuclei remain fairly uniform; no abnormal mitoses Supports “low-grade” rather than moderate/severe dysplasia or carcinoma in situ  Retention of orderly maturation Progressive increase in cytoplasmic eosinophilia and flattening of cells toward the surface is preserved Maturation argues against carcinoma in situ/invasive SCC  Intact basement membrane – no epithelial islands in connective tissue Down-growing rete ridges remain contiguous with the surface epithelium; no keratin pearls or dysplastic nests in the lamina propria Confirms the process is intra-epithelial (dysplasia) and not invasive squamous cell carcinoma  Band-like chronic inflammatory infiltrate in lamina propria Dense lymphocytes and plasma cells hugging the epithelium Common secondary reaction in longstanding leukoplakia; can accentuate rete-ridge elongation  Taken together, the limited (lower-third) cytologic atypia with preservation of maturation, in the setting of a hyperkeratotic plaque, fulfills criteria for mild (low-grade) oral epithelial dysplasia rather than simple reactive hyperkeratosis on the one hand or high-grade dysplasia / carcinoma on the other. |
| 11.9 | Diagnosis: Atypical verrucous hyperplasia with dysplasia; Key Microscopic Features: Corrugated surface with parakeratosis and varying degrees of dysplasia. Mild dysplasia in skip segment bracketed by severe dysplasia; note change in nature of keratin and bulbous rete ridges. Dyscohesion, dyskeratosis, increased nuclear:cytoplasmic ratio, and nuclear pleomorphism. | Based on the provided hematoxylin and eosin (H&E)-stained histopathological images at progressively increasing magnifications, the single most likely histopathological diagnosis is:  Oral Epithelial Dysplasia (Moderate to Severe Dysplasia)  Key Microscopic Features Supporting the Diagnosis:  1. Architectural Disturbances:  Irregular rete ridge elongation and bulbous projections: Seen in low-power images, suggesting a disturbed epithelial-mesenchymal interface.  Loss of normal stratification: Disorganization and lack of orderly maturation from basal to superficial layers.  Drop-shaped rete ridges: Frequently seen in dysplastic lesions.  2. Cytological Atypia:  Nuclear pleomorphism and hyperchromatism: Enlarged, dark-staining, irregularly shaped nuclei are evident throughout the epithelium.  Increased nuclear-to-cytoplasmic ratio: Indicates high cellular activity and reduced cytoplasmic maturation.  Dyskeratosis: Abnormally keratinized cells below the superficial layers.  Increased mitotic activity, including abnormal mitoses: Especially away from the basal layer, a hallmark of dysplasia.  Basal cell hyperplasia: Expansion of basal and parabasal layers.  3. Surface Keratin Changes:  Parakeratosis (retention of nuclei in the keratin layer) and hyperkeratosis: Seen on the surface, often found in leukoplakia and dysplastic lesions.  Why Not Carcinoma?  There is no evidence of stromal invasion (i.e., no breach of the basement membrane).  The cellular atypia and architectural changes, while marked, remain confined to the epithelium.  Conclusion:  The histologic features are characteristic of oral epithelial dysplasia, more specifically in the moderate to severe range. This lesion carries a significant risk of malignant transformation, particularly in high-risk anatomical sites like the floor of mouth, ventrolateral tongue, and soft palate.  Let me know if you'd like a comparative chart of dysplasia grades or need assistance differentiating it from erythroplakia or squamous cell carcinoma. | Histopathological diagnosis (single best answer)  Severe oral epithelial dysplasia / carcinoma in situ (high-grade dysplasia arising in a leukoplakic lesion)  Key microscopic features that support this diagnosis  Architectural changes Cytologic changes “Negative” findings  • Marked epithelial thickening (acanthosis) with bulbous, irregular, “drop-shaped” rete ridges that extend deep into the lamina propria.  • Complete loss of normal maturation and stratification—atypical cells occupy the full epithelial thickness, not just the basal third.  • Surface hyper-/parakeratosis producing the clinical leukoplakic appearance. • Pronounced nuclear pleomorphism and crowding in all layers, with enlarged, hyperchromatic, irregular nuclei and occasional prominent nucleoli.  • Increased nuclear-to-cytoplasmic ratio and loss of cell polarity.  • Numerous mitotic figures, including abnormal and suprabasal mitoses.  • Single-cell dyskeratosis and premature keratinisation within the spinous layers. • The epithelial–connective-tissue interface remains smooth; no epithelial islands, keratin pearls, or desmoplastic stromal reaction are seen.  • Basement membrane appears intact, so there is no invasion into the lamina propria—a key point distinguishing high-grade dysplasia / carcinoma in situ from invasive squamous-cell carcinoma.  Taken together, the full-thickness cytologic atypia, architectural disorganisation, and intact basement membrane fit best with severe epithelial dysplasia (synonym: carcinoma in situ) rather than mild/moderate dysplasia or invasive squamous-cell carcinoma. |
| 11.10 | Diagnosis: Atypical verrucous hyperplasia with dysplasia; Key Microscopic Features: Marked parakeratosis with papillary/verrucous epithelial hyperplasia and severe dysplasia with lymphocytic band at interface. Severe dysplasia with dyskeratosis and lymphocytic band. Keratin pearl at base of epithelium, dyskeratosis, and cells with glassy cytoplasm. | Based on the provided hematoxylin and eosin (H&E)-stained histopathological images of the same lesion at varying magnifications, the single most appropriate histopathological diagnosis is:  Well-Differentiated Squamous Cell Carcinoma (SCC)  Key Microscopic Features Supporting the Diagnosis:  1. Invasive Growth Pattern:  In the low-power image, the epithelium shows downward proliferation into the connective tissue, breaching the basement membrane. This is a hallmark of carcinoma, distinguishing it from dysplastic or pre-invasive lesions like leukoplakia or erythroplakia.  2. Keratin Pearl Formation:  Clear keratin pearls (whorled, eosinophilic concentric layers of keratin) are seen prominently in the higher magnification images.  These are characteristic of well-differentiated SCC, indicating that the malignant squamous cells retain the ability to produce keratin.  3. Cellular Atypia:  Marked cytological atypia, including:  Enlarged, hyperchromatic nuclei  Increased nuclear-to-cytoplasmic ratio  Irregular nuclear membranes  Prominent nucleoli  4. Dyskeratosis:  Individual cell keratinization within the deeper epithelial layers, which is abnormal and pathognomonic of malignancy.  5. Inflammatory Response:  A dense lymphoplasmacytic inflammatory infiltrate is present in the connective tissue beneath the epithelium, often associated with tumor invasion.  6. Desmoplastic Stromal Reaction:  Fibrous stroma around the invading tumor islands, consistent with a reactive host response to invasion.  Conclusion:  These features—especially keratin pearl formation, invasion beyond the basement membrane, and significant cytological atypia—are definitive for well-differentiated squamous cell carcinoma, which is a common malignancy in the oral cavity. | Histopathological diagnosis  Well-differentiated (keratinising) invasive squamous cell carcinoma of the oral mucosa  Key microscopic features that support this diagnosis  Feature How it appears in the provided slides Diagnostic significance  Invasion of the epithelial cells into the lamina propria Irregular, bulbous tongues of epithelium extend beyond the basement membrane; in several foci the epithelial front is separated from the surface epithelium and embedded in a fibrous stroma with a mixed inflammatory infiltrate Confirms malignant, invasive behaviour rather than intra-epithelial (in-situ) disease  Keratin-pearls and individual cell keratinisation Round, concentrically laminated eosinophilic structures packed with keratin are present both within the epithelial tongues and in detached nests; scattered dyskeratotic cells are seen Hallmark of a well-differentiated, keratinising SCC  Cytological atypia through the full thickness Enlarged, pleomorphic, hyperchromatic nuclei; increased nuclear-cytoplasmic ratio; loss of normal maturation gradient; mitotic figures seen above the basal layer Indicates high-grade epithelial dysplasia and malignant transformation  Intercellular bridges (desmosomes) Prominent prickles between tumour cells, best appreciated in medium-power fields Confirms squamous differentiation  Desmoplastic and inflammatory stromal response Dense lymphoplasmacytic infiltrate and fibrovascular stroma surround and dissect between invading epithelial cords; focal haemorrhage is present A typical host reaction to invasive SCC  Irregular surface keratinisation / hyperkeratosis Thick, parakeratinised surface with ridged architecture overlies the dysplastic epithelium Common in oral SCCs that present clinically as leukoplakic or mixed red-white lesions  Taken together, these features are diagnostic of a well-differentiated keratinising squamous cell carcinoma rather than a non-invasive dysplasia, leukoplakia, or erythroplakia. |
| 11.21 | Diagnosis: Mild epithelial dysplasia; Key Microscopic Features: Hyperkeratosis and cells with increased nuclear:cytoplasmic ratio involve less than one-third of the epithelium. Many candidal hyphae within the parakeratin in the absence of spongiotic pustules, spongiosis or leukocyte transmigration, basal cell hyperplasia, nuclear hyperchromasia, and pleomorphism involving only the lower few layers of epithelial cells. Basal cell crowding and cells with increased nuclear:cytoplasmic ratio involving less than one-third of the epithelium; note bulbous rete ridges. | Histopathological Analysis  Single Most Likely Diagnosis:  Epithelial Dysplasia (likely Severe Oral Epithelial Dysplasia)  Key Microscopic Features Supporting the Diagnosis  These hematoxylin and eosin (H&E) stained images represent different magnifications of the same lesion and show the following characteristic changes:  1. Disordered Maturation and Stratification  Loss of normal stratification of the squamous epithelium is evident across the entire thickness in some areas.  Immature basal-like cells extend toward the surface, especially in the first and third images.  2. Cytologic Atypia  Pleomorphism: Variability in nuclear size and shape.  Hyperchromatism: Dark-staining nuclei indicating increased DNA content.  Increased nuclear-to-cytoplasmic ratio.  Prominent nucleoli in many cells.  3. Increased and Abnormal Mitoses  Mitoses are seen above the basal layer, indicating aberrant proliferation — a hallmark of dysplasia.  In some fields, atypical mitotic figures can be inferred.  4. Drop-shaped Rete Ridges (Epithelial Hyperplasia)  Irregular elongation and bulbous rete ridges are seen, especially in the third image.  These may sometimes mimic early invasion but without stromal desmoplasia or a breach in the basement membrane.  5. Lack of Surface Keratin Maturation  The top layers lack normal keratinization (orthokeratosis or parakeratosis), and there's a loss of the orderly maturation gradient toward the surface.  This is more prominent in the second image.  Differential Diagnosis Considered and Ruled Out  Squamous Cell Carcinoma (SCC):  No clear evidence of invasion through the basement membrane or desmoplastic stromal response. Thus, SCC is not yet established.  Hyperkeratosis/Leukoplakia without Dysplasia:  Cytologic and architectural atypia rules this out.  Erythroplakia (Histologically):  Not a histologic diagnosis per se but often shows severe dysplasia or carcinoma in situ, which this likely represents.  Conclusion  The findings are most consistent with Severe Oral Epithelial Dysplasia, a precursor to squamous cell carcinoma. Immediate clinical correlation and follow-up (including possible excision or surveillance) are warranted due to the high risk of malignant transformation. | Histopathological diagnosis  → Severe oral epithelial dysplasia ( carcinoma in situ ) arising in a leukoplakic plaque  Microscopic features that point to this diagnosis  Area of epithelium What is seen in the provided fields Why it matters  Surface layer Thick, compact hyper-ortho/parakeratosis with a ragged keratin surface (Images 1 & 2) Correlates with the clinical “white patch” (leukoplakia) and tells us the lesion is long-standing  Maturation pattern • Loss of normal orderly maturation – the epithelium is replaced almost through its full thickness by atypical cells.  • Only a thin superficial zone shows any attempt at flattening/keratinisation (Images 1–3) When atypia extends beyond the lower third and approaches full thickness we grade it “severe dysplasia / CIS”  Basal & suprabasal zones • Marked basal cell hyperplasia and crowding – nuclei piled 4-5 cells thick.  • Drop-shaped / bulbous rete ridges pushing downward (Images 1 & 3). Architectural disarray is one of the major criteria for severe dysplasia  Cytologic atypia • Enlarged, pleomorphic, hyperchromatic nuclei with irregular nuclear membranes and prominent nucleoli.  • Increased N:C ratio.  • Numerous, sometimes abnormal, mitotic figures extending well above the basal layer (Images 1 & 2).  • Dyskeratotic (prematurely keratinising) individual cells scattered throughout the thickness. Fulfil the cytological criteria for severe dysplasia/CIS  Interface with lamina propria Intact basement membrane; no nests, tongues or keratin pearls invading the connective tissue (all images). Confirms the lesion has not breached the basement membrane, so it is in situ and not an invasive squamous-cell carcinoma  Stromal response Mild chronic inflammatory infiltrate only; no desmoplasia. Absence of stromal invasion reaction supports the in-situ nature  Bottom line: the combination of full-thickness epithelial atypia, pronounced cytologic abnormalities, architectural disturbance, abundant surface keratin and an intact basement membrane is diagnostic of severe oral epithelial dysplasia / carcinoma in situ within a leukoplakia. |
| 11.22 | Diagnosis: Mild-to-moderate dysplasia; Key Microscopic Features: Parakeratosis with festooned and bulbous rete ridges. Mild epithelial dysplasia within this rete ridge. Moderate epithelial dysplasia within this adjacent rete ridge with basal cell hyperplasia involving half the thickness of the epithelium. | Histopathological Diagnosis:  Oral Epithelial Dysplasia (Moderate to Severe Dysplasia)  Key Microscopic Features Justifying the Diagnosis:  Architectural Disturbance:  Irregular epithelial stratification: The normal maturation pattern of the epithelium is disrupted, with basal cells showing maturation defects extending into the mid and upper thirds of the epithelium.  Bulbous and elongated rete ridges: These rete ridges are irregular and broadened, with loss of polarity and architectural organization.  Cytological Atypia:  Nuclear pleomorphism: Enlarged, hyperchromatic nuclei with variation in size and shape are evident.  Increased nuclear-to-cytoplasmic ratio: Indicative of immature cells with dysplastic features.  Prominent nucleoli: Seen in some basal and suprabasal cells.  Increased and abnormal mitotic figures: Mitoses are not restricted to the basal layer and are observed in the suprabasal layers, including atypical forms.  Loss of Maturation and Polarity:  Cells lose their orientation with respect to the basement membrane, a hallmark of dysplasia.  Basal Cell Hyperplasia:  Expansion of the basal and parabasal cell layers, contributing to overall epithelial thickening.  Inflammatory Infiltrate in the Lamina Propria:  The underlying connective tissue shows a moderate lymphocytic infiltrate, often seen as a reactive change associated with epithelial dysplasia.  Conclusion:  The constellation of architectural disruption, significant cytologic atypia, and loss of maturation and polarity support a diagnosis of moderate to severe oral epithelial dysplasia. Although features of carcinoma in situ or invasive squamous cell carcinoma (e.g., breach of the basement membrane) are not clearly evident in the images, the dysplasia is advanced and warrants close clinical surveillance or excision. | Histopathological diagnosis  Oral squamous epithelial dysplasia – moderate grade (also termed “oral intra-epithelial neoplasia, OIN 2,” most often encountered in a leukoplakia)  Key microscopic features that support this diagnosis  Category Observations in the provided sections  Overall architecture • Marked acanthosis with elongated, drop-shaped rete ridges  • Irregular stratification and loss of normal maturation sequence  Basal/Parabasal changes • Basal cell hyperplasia and crowding  • Loss of basal cell polarity  Cytologic atypia • Enlarged, hyperchromatic and pleomorphic nuclei  • Increased nuclear-to-cytoplasmic ratio  • Prominent nucleoli in many cells  Keratinisation abnormalities • Foci of premature individual-cell keratinisation (dyskeratosis) within the lower half of the epithelium  Mitotic activity • Numerous and occasional atypical mitotic figures extending beyond the basal layer but remaining within the lower two-thirds of the epithelium  Inflammatory / stromal interface • Intact basement membrane; no epithelial islands or cords penetrating the lamina propria  • Mild chronic inflammatory infiltrate directly beneath the dysplastic epithelium  The combination of architectural disturbance plus cytologic atypia involving more than the lower third but not the full thickness of the epithelium is characteristic of moderate (grade 2) epithelial dysplasia and separates it from mild dysplasia (changes limited to the basal one-third) and severe dysplasia/carcinoma in situ (full-thickness atypia) as well as from invasive squamous cell carcinoma (which would show breach of the basement membrane). |
| 11.23 | Diagnosis: Moderate epithelial dysplasia; Key Microscopic Features: Parakeratosis, dyscohesion, and festooned rete ridges. Basal cell hyperplasia, suprabasal mitotic figures, and festooned rete ridges. Moderate epithelial dysplasia. | Based on the provided hematoxylin and eosin (H&E) stained histological images taken at low and high magnification, the single most histopathological diagnosis is:  Oral Epithelial Dysplasia (Moderate to Severe Dysplasia)  Key Microscopic Features Supporting the Diagnosis:  Low-Power Image (First Image):  Acanthotic stratified squamous epithelium with an irregular epithelial-stromal interface.  Bulbous rete ridges extending downward into the connective tissue.  Inflammatory infiltrate in the lamina propria, consistent with chronic inflammation.  Hyperkeratosis (thickened keratin layer) on the surface.  Suggestive of epithelial proliferation and early architectural distortion.  High-Power Image (Second Image):  Nuclear pleomorphism: Enlarged, hyperchromatic nuclei with irregular contours.  Loss of maturation and polarity: Basal and parabasal cells appear disorganized with loss of normal maturation toward the surface.  Increased nuclear-to-cytoplasmic ratio: Indicative of dysplastic change.  Mitoses above the basal layer, some of which may be atypical—hallmarks of dysplasia.  Dyskeratosis: Premature keratinization within individual cells in suprabasal layers.  Drop-shaped rete ridges and basilar crowding.  Conclusion:  These histologic features collectively support a diagnosis of moderate to severe oral epithelial dysplasia. This lesion falls within the premalignant spectrum of oral potentially malignant disorders (OPMDs), such as leukoplakia or erythroplakia, and warrants close clinical correlation and possibly excision due to the risk of malignant transformation. | Histopathological diagnosis  Carcinoma in situ (severe epithelial dysplasia) of the oral stratified squamous epithelium  Key microscopic features that support this diagnosis  Category Observations in the two fields  Architecture • Marked epithelial thickening with broad, bulbous, drop-shaped rete ridges.  • Loss of orderly cell maturation and stratification from the basal layer to the surface.  • Basement membrane remains intact; no epithelial nests or cords penetrating the lamina propria, excluding invasive SCC.  Cytology • Full-thickness cytologic atypia: enlarged, pleomorphic, hyperchromatic nuclei with increased N : C ratio in every epithelial layer.  • Abnormal (suprabasal) and occasional atypical mitotic figures scattered throughout the epithelium.  Keratinisation & dyskeratosis • Numerous individually keratinising (dyskeratotic) cells within mid-epithelial layers.  • Focal surface parakeratin/orthokeratin plugs.  Additional findings • Mild-to-moderate chronic inflammatory infiltrate in the underlying connective tissue, a common reactive accompaniment.  Together, the full-thickness architectural disorder and cytologic atypia without stromal invasion meet the criteria for carcinoma in situ (a.k.a. severe epithelial dysplasia) rather than leukoplakia with mild/moderate dysplasia or invasive squamous-cell carcinoma. |
| 11.24 | Diagnosis: Moderate-to-severe dysplasia; Key Microscopic Features: Hyperkeratosis with dyscohesion and pleomorphic cells involving half to two-thirds of the epithelium for the most part. Another area of the same lesion showing markedly pleomorphic and multinucleated dysplastic epithelial cells involving greater than two-thirds of the epithelium. The giant cells have little cytoplasm. | Histopathological Analysis of the Provided Images  Single Most Likely Histopathological Diagnosis:  Oral Epithelial Dysplasia (Moderate to Severe Dysplasia)  Key Microscopic Features Justifying the Diagnosis:  The provided hematoxylin and eosin (H&E) stained sections (at two magnifications) show several characteristic features of oral epithelial dysplasia, which may be graded as moderate to severe based on the extent and severity of epithelial atypia.  Microscopic Features Observed:  1. Architectural Disturbances:  Loss of normal epithelial stratification: Disorganization of epithelial layers is visible, especially in the middle and upper thirds of the epithelium.  Basal cell hyperplasia: There is expansion of the basal and parabasal layers upward into the mid-epithelium.  Irregular epithelial maturation: Maturation is not limited to the basal layer but extends higher, indicating loss of polarity.  Drop-shaped rete ridges: Elongated and bulbous rete ridges suggest disturbance in epithelial proliferation.  2. Cytologic Atypia:  Nuclear pleomorphism: Variation in nuclear size and shape throughout the epithelial thickness.  Hyperchromatism: Dark-staining nuclei suggest increased DNA content.  Increased nuclear-to-cytoplasmic ratio: Particularly in upper layers where this is not typical.  Abnormal mitotic figures: Seen above the basal/parabasal layers, indicating dysregulated proliferation.  Prominent nucleoli: Atypical and enlarged nucleoli are occasionally visible.  3. Other Findings:  Loss of cohesion between keratinocytes in some foci.  No clear evidence of invasion through the basement membrane, which helps exclude frank invasive squamous cell carcinoma.  Differential Diagnostic Consideration:  Leukoplakia: Clinical term; histology varies. Could harbor dysplasia.  Erythroplakia: Also clinical, often more dysplastic histologically, but this image lacks abundant vascularity or red cell extravasation typical of erythroplakia.  Carcinoma in situ: A consideration, especially if dysplasia involves full epithelial thickness—this appears nearly full-thickness but without definitive top-to-bottom atypia throughout.  Invasive squamous cell carcinoma: Ruled out due to intact basement membrane and absence of stromal invasion.  Conclusion:  The microscopic findings support a diagnosis of moderate to severe epithelial dysplasia, falling under the oral potentially malignant disorder (OPMD) spectrum, necessitating close clinical correlation and follow-up or potential excision depending on clinical scenario. | Histopathological diagnosis (single best):  High-grade oral epithelial dysplasia – severe epithelial dysplasia / carcinoma in situ (non-invasive precursor of squamous cell carcinoma).  Key microscopic features in the two H&E sections that support this diagnosis  Category Findings visible in the images  Architectural disturbance • Irregular, bulbous, “drop-shaped” rete ridges with loss of the normal knife-edge profile.  • Loss of orderly maturation: the epithelium no longer shows a clear progression from small basaloid cells to flattened superficial cells; instead atypical cells occupy almost the entire thickness.  • Surface hyper-/parakeratosis producing a clinically white plaque (leukoplakia).  Cytologic atypia • Marked nuclear pleomorphism and hyperchromasia in basal, parabasal and upper thirds of the epithelium.  • Enlarged nuclei with increased nuclear-to-cytoplasmic ratio.  • Prominent, often multiple nucleoli.  Abnormal mitotic activity • Numerous mitotic figures, including atypical forms, are seen well above the basal layer (suprabasal/upper-third mitoses) – a hallmark of high-grade dysplasia.  Dyskeratosis • Premature individual cell keratinisation (eosinophilic, shrunken “dyskeratotic” cells) scattered through the spinous and superficial layers.  Basement membrane status • Despite downward proliferation, the epithelial–connective tissue interface remains intact; there are no isolated nests or tongues of epithelium invading the lamina propria, distinguishing this lesion from invasive squamous cell carcinoma.  Taken together, the full-thickness cytological atypia, suprabasal/upper-third mitoses and loss of maturation without stromal invasion fulfil the criteria for severe (high-grade) oral epithelial dysplasia / carcinoma in situ rather than mild–moderate dysplasia or invasive squamous cell carcinoma. |
| 11.25 | Diagnosis: Severe dysplasia; Key Microscopic Features: Moderate dysplasia is present on the right and severe dysplasia on the left. Marked dyscohesion with pleomorphic and hyperchromatic cells and dyskeratosis. | Based on the histopathological examination of the provided hematoxylin and eosin (H&E) stained images at low and high magnification, the single most likely diagnosis is:  Squamous Cell Carcinoma (SCC)  Key Microscopic Features Supporting the Diagnosis:  Low Magnification (First Image):  Epithelial proliferation: There is clear thickening and irregular downward proliferation of the stratified squamous epithelium into the underlying connective tissue.  Loss of maturation: The normal maturation pattern from basal to superficial layers is disrupted.  Architectural disarray: Irregular rete ridges with a bulbous and invasive pattern, deviating from the orderly architecture of non-dysplastic mucosa.  High Magnification (Second Image):  Cellular pleomorphism: Marked variation in cell size and shape, indicating loss of uniformity.  Nuclear atypia: Enlarged, hyperchromatic nuclei with irregular contours and prominent nucleoli.  Abnormal mitotic figures: Increased and atypical mitoses, not limited to the basal layer.  Dyskeratosis: Individual cell keratinization within the epithelial layers, often a hallmark of malignancy.  Evidence of invasion: Islands and nests of malignant squamous cells breaching the basement membrane into the connective tissue stroma.  These histopathological features are diagnostic of invasive squamous cell carcinoma, distinguishing it from non-invasive lesions such as leukoplakia, erythroplakia, or epithelial dysplasia, where invasion beyond the basement membrane is absent. | Histopathological diagnosis (single best answer)  ▪ Oral squamous cell carcinoma in situ (= severe/high-grade epithelial dysplasia)  Key microscopic features that support this diagnosis  Level What is seen in the images Significance  Architectural changes • Marked epithelial thickening with broad, drop-shaped rete ridges that extend deeply and irregularly into the lamina propria  • Loss of normal basal–to–suprabasal polarity and stratification Indicates severe disturbance of epithelial architecture typical of high-grade dysplasia / CIS  Cytologic atypia (both magnifications) • Cells throughout the entire thickness of the epithelium show enlarged, pleomorphic, hyperchromatic nuclei and increased N:C ratio  • Numerous mitotic figures, including atypical forms, present well above the basal layer Full-thickness cytologic atypia and suprabasal mitoses fulfill criteria for carcinoma in situ  Abnormal maturation / keratinisation • Premature (“dyskeratotic”) keratinisation and individual cell keratin pearls within mid-epithelium  • Foci of ballooning / clear-cell change and acantholysis producing pseudoglandular or vacuolated spaces Dyskeratosis and disordered maturation are hallmarks of severe dysplasia; clear-cell/ballooning change is a common degenerative feature in high-grade lesions  Basement-membrane status • Epithelial–lamina propria interface remains smooth and intact; no nests or cords of tumour breach the basement membrane Confirms the process is in situ (non-invasive); distinguishes it from invasive SCC  Stromal response • Band-like chronic inflammatory infiltrate in superficial lamina propria A frequent accompaniment but not diagnostic; absence of an obvious desmoplastic / invasive tumour response further supports CIS  Because the atypical changes involve the full epithelial thickness yet there is no invasion beyond the basement membrane, the lesion is best classified as carcinoma in situ (severe epithelial dysplasia) rather than mild/moderate dysplasia or invasive squamous cell carcinoma. |
| 11.26 | Diagnosis: Carcinoma-in-situ; Key Microscopic Features: Full thickness involvement of the epithelium and severe dysplasia on the right where the superficial two to three layers of keratinocytes are mature and still horizontally aligned. Cells have pleomorphic nuclei throughout the epithelium and there are many high mitotic figures. | Histopathological Diagnosis:  Oral Epithelial Dysplasia (Moderate to Severe Dysplasia)  Key Microscopic Features:  Both images—one at a lower magnification and one at a higher magnification—demonstrate classic architectural and cytological features consistent with oral epithelial dysplasia, most likely in the moderate to severe range. Here's a detailed breakdown:  1. Architectural Disturbances:  Loss of stratification: The normal maturation of epithelial cells from basal to superficial layers is disrupted.  Bulbous rete ridges: The rete pegs appear broadened and drop down irregularly, a hallmark of dysplastic epithelium.  Basal cell hyperplasia: Expansion of the basal and parabasal layers into the upper layers is evident.  2. Cytological Atypia:  Nuclear pleomorphism: Variation in nuclear size and shape is apparent.  Hyperchromatism: The nuclei are darker than normal due to increased DNA content.  Increased nuclear-to-cytoplasmic (N:C) ratio: Cells show less cytoplasm relative to their enlarged nuclei.  Abnormal mitotic figures: Although not overtly abundant in the images, mitoses above the basal layer and occasional atypical mitoses may be present.  3. Lack of Surface Maturation:  The upper epithelial layers do not show proper keratinization or maturation—this is a critical feature distinguishing dysplasia from reactive epithelial hyperplasia.  4. Inflammatory Response:  Moderate chronic inflammatory infiltrate in the connective tissue is present, which is commonly associated with dysplasia and epithelial stress.  Summary Justification:  The combination of cytologic atypia (nuclear pleomorphism, hyperchromasia, increased N:C ratio), architectural distortion (loss of maturation, bulbous rete ridges), and basal cell hyperplasia with disturbance in normal epithelial maturation is diagnostic of oral epithelial dysplasia, most likely moderate to severe.  Further stratification (mild, moderate, severe) would benefit from clinical correlation and broader sampling, but based on these sections, the atypia appears to extend well beyond the lower third of the epithelium, which supports a diagnosis of moderate to severe dysplasia. | Histopathological diagnosis (single best answer)  High-grade oral epithelial dysplasia / carcinoma in situ (severe epithelial dysplasia).  Key microscopic features that support this diagnosis  Architectural changes Cytologic changes  • Marked epithelial thickening (acanthosis) with broad, bulbous, irregular rete ridges that push downward but do not breach the basement membrane. • Nuclear pleomorphism and hyperchromasia involving virtually the full thickness of the epithelium.  • Loss of orderly maturation and stratification – basaloid-appearing cells extend to the superficial layers; surface keratinisation is minimal or disordered. • Increased nuclear-to-cytoplasmic ratio, irregular nuclear contours, coarse chromatin, and occasional prominent nucleoli.  • Loss of normal polarity of basal and parabasal cells; crowding and drop-shaped rete ridges. • Numerous mitotic figures, including atypical forms, seen well above the basal layer (suprabasal mitoses).  • Intact basement membrane; no invasive nests or cords of cells in the lamina propria. • Focal dyskeratosis/individual cell keratinisation within the spinous layer.  • Subepithelial lamina propria shows a band-like chronic inflammatory infiltrate but no desmoplastic stromal reaction.  Together, the combination of full-thickness architectural disarray, widespread cytologic atypia, and absence of stromal invasion establishes a diagnosis of high-grade (severe) epithelial dysplasia / carcinoma in situ rather than invasive squamous-cell carcinoma. |
| 11.27 | Diagnosis: Carcinoma-in-situ; Key Microscopic Features: Full thickness dysplasia with pale keratinocytes, dyskeratosis, and glassy cytoplasm. | Histopathological Diagnosis:  Moderate to Severe Epithelial Dysplasia (Oral Epithelial Dysplasia)  Key Microscopic Features Supporting the Diagnosis:  Low Magnification (First Image):  Acanthosis: Thickened stratified squamous epithelium.  Rete Ridge Elongation and Bulbous Down-Growths: Irregular, broadened rete pegs extending downward.  Loss of Normal Maturation Pattern: Disruption of the orderly stratification of the epithelial layers.  High Magnification (Second and Third Images):  Nuclear Pleomorphism: Marked variability in nuclear size and shape.  Increased Nuclear-to-Cytoplasmic Ratio: Indicative of increased cellular atypia.  Hyperchromatism: Dark-staining nuclei, reflecting increased DNA content.  Abnormal Mitoses: Mitoses seen above the basal layer (suprabasal mitotic activity), which is atypical.  Loss of Polarity: Disorganized orientation of cells throughout the epithelial layers.  Dyskeratosis: Premature keratinization within individual cells or cell clusters.  Basal Cell Hyperplasia: Expansion of the basal and parabasal cell layers.  Conclusion:  These histopathologic findings, particularly the architectural disorganization and cytologic atypia involving more than the lower third but not full-thickness of the epithelium, are diagnostic of moderate to severe epithelial dysplasia. There is no definitive evidence of invasion into the underlying connective tissue (i.e., no stromal invasion), which rules out squamous cell carcinoma at this stage, but this lesion has high malignant potential. | Histopathological diagnosis (single best):  Oral squamous carcinoma in situ – i.e. severe (high-grade) oral epithelial dysplasia with no demonstrable invasion through the basement membrane.  Microscopic features that support this diagnosis  Level Findings Significance  Whole-section (low power) • Epithelium is markedly thickened with broad, bulbous, drop-shaped rete ridges that push downward but remain contiguous with the surface.  • Basement membrane is preserved; no discrete epithelial nests in the lamina propria. Architecture typical of high-grade dysplasia / CIS and not an invasive SCC.  Maturation pattern • Virtually complete loss of normal stratification: cellular atypia extends from basal layer to the surface.  • Focal premature keratinisation / epithelial (keratin) pearls located entirely within the epithelium. Fulfil the criterion of full-thickness dysplasia.  Cytological atypia (high power) • Marked nuclear pleomorphism, enlarged hyperchromatic nuclei, increased N:C ratio.  • Numerous and abnormally placed mitotic figures, including suprabasal mitoses.  • Individual cell keratinisation (dyskeratosis). Hallmarks of severe dysplasia / CIS.  Stromal interface • Intact basement membrane; underlying connective tissue shows chronic inflammatory infiltrate but no invasive epithelial islands or desmoplasia. Confirms lesion is intra-epithelial (in situ).  These combined architectural and cytological abnormalities, extending through the entire epithelial thickness without breach of the basement membrane, are diagnostic of carcinoma in situ / severe epithelial dysplasia in the oral cavity. |
| 11.27 | Diagnosis: Carcinoma-in-situ; Key Microscopic Features: Full thickness dysplasia composed of pale keratinocytes that exhibit dyskeratosis and glassy cytoplasm. | Histopathological Diagnosis:  Moderate Epithelial Dysplasia (Oral Dysplasia)  Key Microscopic Features Supporting the Diagnosis:  Low Magnification (Image 1):  Thickened epithelium with acanthosis (expansion of the spinous layer).  Loss of normal epithelial maturation and architecture.  Elongation and bulbous rete ridges, some showing irregular downward proliferation.  No evidence of invasion through the basement membrane—suggesting a pre-malignant process rather than carcinoma.  High Magnification (Image 2):  Nuclear pleomorphism: Variation in nuclear size and shape.  Nuclear hyperchromatism: Dark-staining nuclei indicate increased DNA content.  Increased nuclear-to-cytoplasmic ratio.  Loss of polarity of basal and parabasal cells.  Increased mitotic activity, with mitotic figures seen above the basal layer (abnormal distribution).  No evidence of keratin pearl formation or invasive nests, which helps rule out invasive squamous cell carcinoma.  Summary Justification:  The combination of architectural disarray and cytological atypia confined to the lower and mid-portions of the epithelium, without breach of the basement membrane, most closely aligns with moderate epithelial dysplasia. This is a pre-malignant condition and part of the spectrum that includes leukoplakia and erythroplakia lesions exhibiting dysplastic changes. | Histopathological diagnosis – single best answer  Severe oral epithelial dysplasia / carcinoma-in-situ (full-thickness intra-epithelial squamous neoplasia)  Key microscopic features that support this diagnosis  Level Feature What is seen in the images  Architecture • Marked epithelial thickening with irregular, bulbous/drop-shaped rete ridges  • Loss of orderly maturation – no smooth progression from basal → prickle → granular → keratin layers Low-power image shows a broad, uneven epithelium with elongation and crowding of ridges; maturation appears disorganised through the whole thickness  Cytology (full thickness) • Enlarged, pleomorphic, hyperchromatic nuclei present from basal layer right up to the surface  • Increased nuclear-to-cytoplasmic ratio  • Dyskeratotic/individual cell keratinisation within the spinous layer High-power image demonstrates nuclear atypia in virtually every epithelial layer; superficial cells still retain large, dark nuclei and dyskeratotic cells are scattered throughout  Mitotic activity • Numerous mitotic figures, including in suprabasal layers; atypical forms may be seen Basal and parabasal thirds show frequent mitoses extending above the basal compartment  Loss of polarity & cohesion • Disturbed cell polarity, crowding and overlapping nuclei  • Intercellular bridges are diminished in areas of marked atypia High-power view shows haphazard orientation and crowding of nuclei, with focal loss of intercellular bridges  Basement membrane • Remains intact – no epithelial nests, tongues or single cells seen in the lamina propria Low-power image shows a smooth, uninterrupted epithelial–stromal interface with no invasion  Taken together, the full-thickness cytological atypia, architectural disorganisation, suprabasal mitoses and an intact basement membrane fulfil WHO criteria for severe epithelial dysplasia / carcinoma in situ, distinguishing it from mild-to-moderate dysplasia (atypia limited to lower thirds) and from invasive squamous cell carcinoma (which would show stromal invasion). |
| 11.30 | Diagnosis: Moderate-to-severe epithelial dysplasia; Key Microscopic Features: Dysplasia of the excretory duct with basal cell hyperplasia and pleomorphic cells. | Based on the provided hematoxylin and eosin (H&E) stained histological images at different magnifications, the single most likely histopathological diagnosis is:  Oral Squamous Cell Carcinoma (SCC)  Key Microscopic Features Justifying the Diagnosis:  1. Invasive Epithelial Nests and Islands:  Seen clearly in both low and high magnification, the epithelium is not restricted to the surface but infiltrates into the underlying connective tissue as irregular islands and nests.  This is a hallmark of malignancy and differentiates SCC from dysplasia or carcinoma in situ.  2. Keratin Pearl Formation:  In the higher magnification image, there is evidence of keratin pearls—concentrically layered keratinized material within the epithelial nests—indicating a well-differentiated squamous phenotype.  3. Cellular and Nuclear Atypia:  Marked pleomorphism, hyperchromatic nuclei, increased nuclear-to-cytoplasmic ratio, and loss of maturation are apparent in the epithelial cells.  These atypical features are evident even at the higher magnification.  4. Disruption of Basement Membrane:  There is clear evidence of basement membrane invasion, which distinguishes invasive SCC from carcinoma in situ or dysplasia.  5. Desmoplastic Stromal Response:  The surrounding connective tissue shows a fibrotic or desmoplastic reaction, which often accompanies invasive carcinoma.  Differential Diagnostic Consideration (Ruled Out):  Leukoplakia / Erythroplakia: These are clinical terms, not histopathological diagnoses.  Oral Epithelial Dysplasia: Shows architectural and cytological atypia but lacks invasion through the basement membrane.  Carcinoma In Situ: Full-thickness epithelial dysplasia without invasion into the stroma.  Final Remark:  The presence of invasive epithelial nests with keratin pearls and cytological atypia confirms the diagnosis of oral squamous cell carcinoma, most likely well-differentiated based on keratinization. | Histopathological diagnosis (single best answer)  Conventional keratinising invasive squamous cell carcinoma of the oral mucosa – moderately differentiated grade.  Key microscopic findings supporting this diagnosis  Observation (seen in the two H-E images) Diagnostic implication  Irregular nests and tongues of squamous epithelium penetrating the lamina propria – clearly detached from the surface epithelium and surrounded by fibro-vascular stroma Confirms breach of the basement membrane → true invasion, ruling out mere dysplasia / carcinoma in situ  Tumour cells with obvious cytologic atypia: enlarged hyperchromatic nuclei, irregular nuclear contours, increased N/C ratio, nuclear crowding and pleomorphism Malignant cytology characteristic of SCC  Loss of normal maturation and polarity within the epithelial islands; disordered “bottom-to-top” maturation Marks transformation from dysplasia to carcinoma  Focal intracellular/individual-cell keratinisation and rare early keratin-pearl formation (eosinophilic cytoplasm, intercellular bridges still recognisable) Indicates keratinising and moderately differentiated nature  Desmoplastic connective-tissue reaction and patchy chronic inflammatory infiltrate surrounding the invading nests Typical stromal response to invasive SCC  Surface mucosa shows dysplastic change (drop-shaped elongated rete ridges, basal crowding) – but the decisive feature is the underlying invasion Fits an exophytic/ulcerated carcinoma arising in a leukoplakic/erythroplakic precursor  Taken together, the combination of epithelial invasion, malignant cytologic features and focal keratin production is diagnostic of moderately differentiated invasive squamous cell carcinoma in the oral cavity. |
| 11.30 | Diagnosis: Moderate-to-severe epithelial dysplasia; Key Microscopic Features: Dysplasia of the excretory duct with basal cell hyperplasia, dyscohesion, and pleomorphic cells. | Based on the two provided hematoxylin and eosin (H&E) stained histological images taken at different magnifications, the single most likely histopathological diagnosis is:  Invasive Squamous Cell Carcinoma (SCC) of the oral cavity  Key Microscopic Features Justifying the Diagnosis:  Low Magnification Image (First Image):  Epithelial dysplasia/invasion: The surface epithelium appears irregular, with disruption of the basement membrane and extension of epithelial nests into the underlying connective tissue—indicative of invasive behavior.  Keratin pearls: Whorled accumulations of eosinophilic keratin in the center of epithelial nests, a hallmark of well-differentiated squamous cell carcinoma.  Desmoplastic stromal reaction: Dense fibrotic stromal response surrounding infiltrating tumor islands, typical of carcinoma invasion.  Irregular epithelial nests: Penetrating into the connective tissue with atypical squamous cells.  High Magnification Image (Second Image):  Marked cytologic atypia: Enlarged, pleomorphic, hyperchromatic nuclei with prominent nucleoli.  Individual cell keratinization: Another feature of squamous differentiation.  Abnormal mitotic figures: Increased and atypical mitoses indicating high proliferative activity.  Intercellular bridges: Indicating squamous differentiation.  Conclusion:  The combination of invasive epithelial nests, keratin pearl formation, and marked cellular atypia clearly supports a diagnosis of invasive squamous cell carcinoma, a common malignant lesion in the spectrum that includes leukoplakia, erythroplakia, and dysplasia. | Histopathological diagnosis  Invasive, well-differentiated (keratinising) squamous cell carcinoma of the oral mucosa.  Key microscopic features supporting this diagnosis  Region Findings Diagnostic significance  Surface epithelium (low power) • Hyper-plastic stratified squamous mucosa with atypical rete pegs.  • Loss of normal maturation and architectural polarity. Indicates severe epithelial dysplasia but, by itself, dysplasia stops at the basement membrane.  Lamina propria / submucosa • Multiple irregular nests, cords and islands of squamous epithelium lying separate from the surface, infiltrating the fibrous stroma.  • Surrounding stroma shows desmoplastic reaction and chronic inflammatory infiltrate. True invasion—tumour has breached the basement membrane, distinguishing carcinoma from in-situ dysplasia.  Tumour islands (high power) • Cells have abundant eosinophilic cytoplasm, inter-cellular bridges and marked nuclear pleomorphism, hyperchromasia and prominent nucleoli.  • Concentric “keratin pearls” with central lamellated keratin (seen clearly in the high-magnification image).  • Foci of individual-cell keratinisation and dyskeratosis. Keratin pearls and intercellular bridges show squamous differentiation; preservation of keratin production signifies well-differentiated grade.  Additional clues • Occasional vascular/duct-like structure entrapped by tumour.  • Mitotic figures, including atypical forms.  • Stromal inflammatory response. Common accompaniments of invasive oral SCC.  The combination of stromal invasion by atypical squamous islands plus definitive keratin-pearl formation unequivocally identifies a keratinising, well-differentiated squamous cell carcinoma rather than leukoplakia, erythroplakia or non-invasive dysplasia. |
| 11.31 | Diagnosis: Acantholytic dysplasia; Key Microscopic Features: Acantholytic keratinocytes within hypertrophic actinic keratosis. | Single Most Likely Histopathological Diagnosis:  Squamous Cell Carcinoma (SCC) of the Oral Mucosa  Key Microscopic Features Supporting This Diagnosis:  Invasive Growth Pattern:  In both images, particularly the first (lower magnification), there is epithelial invasion into the underlying stroma, indicating a breach of the basement membrane. This is a defining feature of malignancy (i.e., invasive squamous cell carcinoma vs. carcinoma in situ or dysplasia).  Keratin Pearls and Dyskeratosis:  In the second image (higher magnification), there are conspicuous keratin pearls—concentric layers of keratinized squamous cells. This is a hallmark of well-differentiated squamous cell carcinoma.  Numerous individual dyskeratotic cells (premature keratinization) are also present.  Cellular Atypia:  Marked nuclear pleomorphism, hyperchromasia, and increased nuclear-to-cytoplasmic ratio are seen, especially in the second image.  Atypical mitotic figures may be present (though not overtly visible in these specific fields).  Desmoplastic Stromal Response:  The surrounding stroma shows evidence of fibrosis and dense lymphocytic infiltrate, consistent with the host reaction to tumor invasion.  Ulceration and Necrosis:  Areas of epithelial ulceration and necrotic debris (white space and eosinophilic areas) suggest tissue destruction, a common finding in ulcerated SCC lesions.  Conclusion:  The constellation of invasive epithelial nests, keratin pearls, dyskeratosis, cellular atypia, and stromal response points strongly toward invasive squamous cell carcinoma, most likely well-differentiated based on keratinization patterns. | Single best histopathological diagnosis  Erosive / ulcerative oral lichen planus (lichenoid mucositis).  Key microscopic features visible in the two photomicrographs  Feature How it appears in the provided images Diagnostic significance  1. Dense, band-like (lichenoid) lymphocytic infiltrate A solid sheet of small, dark-staining lymphocytes hugs the epithelial–connective-tissue interface (particularly obvious in the low-power image). Hallmark of lichenoid/interface mucositis.  2. Basal-cell hydropic / vacuolar degeneration The basal layer shows pallor and vacuoles; many basal keratinocytes are swollen or have disappeared, creating a jagged line. Explains the clefting (Max-Joseph space) and distinguishes lichenoid inflammation from simple hyperkeratosis or dysplasia.  3. Civatte (colloid) bodies / apoptotic keratinocytes Eosinophilic, rounded remnants of keratinocytes are scattered within the basal layer and immediately above it. Classic for lichen planus; represents damaged basal cells.  4. Subepithelial cleft (Max-Joseph space) An empty, sharply delineated space just beneath the epithelium is especially prominent in the high-power image. Produced by basal-cell destruction; characteristic but not pathognomonic.  5. Saw-toothed rete-ridge architecture with surface hyperkeratosis The rete pegs taper and angle sharply; the epithelial surface is thick and compactly keratinised. Typical architectural change of lichen planus presenting clinically as a white (leukoplakia-like) patch.  6. Absence of true epithelial dysplasia or invasive growth Although individual keratinocytes are damaged, there is no orderly upward maturation disturbance, no atypical mitoses, and no epithelial islands invading the lamina propria. Helps rule out oral epithelial dysplasia or squamous-cell carcinoma.  Together, the intense lichenoid infiltrate, basal-cell degeneration with Civatte bodies, subepithelial clefting, and saw-tooth rete ridges firmly point to oral lichen planus rather than leukoplakia with dysplasia or early squamous-cell carcinoma. |
| 11.32 | Diagnosis: Lichenoid mucositis; Key Microscopic Features: Hyperkeratosis, lymphocytic infiltration, and reactive epithelial atypia. | ased on the provided hematoxylin and eosin (H&E) stained histopathological images at varying magnifications, here is the analysis:  Single Most Likely Histopathological Diagnosis:  Severe Epithelial Dysplasia / Carcinoma In Situ (CIS)  This lesion lies within the oral dysplasia spectrum but shows architectural and cytologic changes that border on carcinoma in situ, although there is no clear invasion through the basement membrane.  Key Microscopic Features Supporting the Diagnosis:  1. Architectural Disturbance of the Epithelium (Seen in all images, especially first and second):  Loss of normal maturation and stratification of the epithelium.  The dysplastic changes extend through the full thickness of the epithelium.  Bulbous rete ridges and loss of basal cell polarity.  2. Cytological Atypia (Especially evident in the second and third images):  Enlarged, hyperchromatic nuclei with pleomorphism.  Increased nuclear-to-cytoplasmic ratio.  Prominent nucleoli.  Dyskeratosis (premature keratinization of individual cells within the spinous layer).  Abnormal mitotic figures including mitoses in the upper epithelial layers.  3. Lack of Invasion (Important exclusion for SCC):  The basement membrane appears intact, with no evidence of epithelial cells breaching into the underlying connective tissue (as would be seen in invasive squamous cell carcinoma).  4. Inflammatory Infiltrate (First image):  Dense chronic inflammatory infiltrate in the lamina propria, which is often reactive to epithelial dysplasia.  Conclusion:  The microscopic features are consistent with severe epithelial dysplasia or carcinoma in situ, which represents a high-risk premalignant lesion in the oral cavity. Careful clinicopathological correlation is needed, and complete excision with clear margins is typically recommended due to the significant risk of progression to invasive squamous cell carcinoma (SCC). | Histopathological diagnosis (single best):  Oral Lichen Planus (lichenoid interface mucositis)  Key microscopic features that point to this diagnosis  Layer / Structure What is seen in the images Why it matters  Surface epithelium • Thickened, largely parakeratin‐covered surface (clinical “leukoplakic” look)  • Saw-tooth elongation of rete ridges Hyper-/parakeratosis with acanthosis is typical of lichen planus and helps explain a white plaque clinically. The angular (“saw-tooth”) ridges are classic.  Basal & lower spinous layers • Hydropic (liquefaction) degeneration of basal cells  • Numerous apoptotic/Civatte (colloid) bodies  • Focal Max-Joseph subepithelial clefts Basal cell degeneration and Civatte bodies are hallmarks of a lichenoid interface reaction; the cleft is a secondary artifact created by basal cell damage.  Inflammatory infiltrate • Dense, band-like sheet of mainly small lymphocytes hugging the epithelial–connective tissue junction A compact subepithelial T-cell band that “obscures” the junction is the sine qua non of lichen planus. The infiltrate in the images is exactly at that location and depth.  Additional points • No downward-invading epithelial islands, no keratin pearls – i.e., no evidence of invasive squamous cell carcinoma  • Atypia, if present, is limited and reactive – falls short of true epithelial dysplasia Helps exclude the other entities in the differential (epithelial dysplasia / carcinoma).  Taken together, the constellation of (1) basal-cell liquefaction with Civatte bodies, (2) a sharply demarcated lichenoid lymphocytic band, (3) saw-tooth rete ridges and surface parakeratosis, and (4) absence of invasive growth or significant dysplasia is diagnostic of oral lichen planus. |
| 11.34 | Diagnosis: HPV-associated oral dysplasia; Key Microscopic Features: Parakeratosis, atypical cells with pericellular halos, and nuclear atypia. | Based on the provided hematoxylin and eosin (H&E) stained histological images at low and high magnification, the most likely histopathological diagnosis is:  Squamous Cell Carcinoma (SCC) of the Oral Mucosa  Key Microscopic Features Supporting This Diagnosis:  1. Invasion of Epithelial Cells into the Underlying Connective Tissue:  In both images, particularly clear in the lower half of the tissue, there is downward proliferation of atypical epithelial cells into the underlying stroma, breaching the basement membrane—a hallmark of malignancy.  2. Keratin Pearls:  Multiple keratin pearls (concentric eosinophilic structures) are observed throughout the epithelium, which are characteristic of well-differentiated squamous cell carcinoma.  3. Cellular and Nuclear Pleomorphism:  There is marked variability in nuclear size and shape, with hyperchromatic and pleomorphic nuclei, indicating high-grade cellular atypia.  4. Increased Mitotic Activity:  Numerous mitotic figures, some atypical, are visible, consistent with rapid cellular proliferation and malignancy.  5. Loss of Normal Epithelial Maturation and Architecture:  The normal stratification of squamous epithelium is disrupted. There's loss of polarity, disordered maturation, and basal cell crowding.  6. Desmoplastic Stromal Response:  In the stroma underlying the invasive nests, there's evidence of fibrotic (desmoplastic) reaction, which is often seen accompanying invasive carcinoma.  Differential Diagnoses Considered but Less Likely:  Severe epithelial dysplasia or carcinoma in situ: These lack definitive invasion into the connective tissue.  Erythroplakia or leukoplakia with dysplasia: These are clinical terms and typically exhibit pre-invasive changes without stromal invasion or keratin pearls.  Summary:  These features—particularly stromal invasion, keratin pearl formation, and nuclear atypia—clearly indicate invasive squamous cell carcinoma, likely well-differentiated based on the extent of keratinization seen. | Histopathological diagnosis (single best answer)  ‣ Oral squamous cell carcinoma in situ (= severe epithelial dysplasia / oral intra-epithelial neoplasia grade III)  Key microscopic features visible in the two H-E fields that support this diagnosis  Category Features seen in the images Significance  Architectural disturbance • Marked epithelial thickening with bulbous, irregular, “drop-shaped” rete ridges that push toward—but do not breach—the basement membrane.  • Loss of normal stratified maturation; basaloid cells extend to the surface. Indicates full-thickness dysplasia rather than a reactive hyperplasia.  Cytologic atypia • Marked nuclear pleomorphism and hyperchromasia throughout the epithelium.  • Increased N:C ratio, irregular nuclear membranes, coarse chromatin.  • Numerous atypical and suprabasal mitotic figures. Hallmarks of high-grade dysplasia / carcinoma in situ.  Abnormal keratinisation • Multiple intra-epithelial keratin pearls and dyskeratotic (“individual cell keratinisation”) cells (eosinophilic, concentric whorls). Keratin-pearl formation within the epithelial thickness signals severe dysplasia; when still confined above the basement membrane it is CIS, not invasive SCC.  Basement-membrane status • Intact, sharply delineated interface between epithelium and underlying lamina propria; no tumour nests or cords penetrating into the connective tissue. Confirms that invasion has not occurred, separating carcinoma in situ from invasive squamous-cell carcinoma.  Taken together, the full-thickness architectural and cytologic abnormalities with intact basement membrane constitute oral squamous cell carcinoma in situ / severe epithelial dysplasia. |
| 11.35 | Diagnosis: HPV-associated oral dysplasia; Key Microscopic Features: Hyperkeratosis, papillary epithelial proliferation, and full thickness dysmaturation with karyorrhectic cells. | Based on the provided hematoxylin-and-eosin (H&E) stained histological images of an oral lesion at low and high magnification, the single most likely histopathological diagnosis is:  Squamous Cell Carcinoma (SCC) of the Oral Cavity  Key Microscopic Features Supporting This Diagnosis:  1. Invasive Growth Pattern (Low Magnification Image):  The low-power image shows epithelial invasion into the underlying connective tissue, breaching the basement membrane—a hallmark of malignancy.  The normal mucosal architecture is disrupted with irregular epithelial projections infiltrating the stroma.  2. Cytologic Atypia (High Magnification Image):  Marked nuclear pleomorphism: Variation in size and shape of nuclei.  Hyperchromasia: Darkly stained nuclei, indicating increased DNA content.  Prominent nucleoli, another feature of increased cellular activity.  3. Increased and Abnormal Mitoses:  Presence of frequent mitotic figures, some of which may be atypical, suggests high proliferative activity.  4. Keratin Pearl Formation:  The high-power image shows keratin pearls, which are concentric layers of keratinized cells—classic for well-differentiated squamous cell carcinoma.  5. Loss of Normal Maturation and Polarity:  The epithelium lacks the orderly maturation from basal to superficial layers, and cells exhibit disorganized growth.  Conclusion:  The histological images demonstrate definitive features of invasive squamous cell carcinoma, most likely well-differentiated based on the presence of keratin pearls. This rules out premalignant conditions like leukoplakia or dysplasia, where invasion and keratin pearls would not be present. | Histopathological diagnosis (single best):  Well-differentiated (keratinising) invasive squamous cell carcinoma of the oral mucosa.  Key microscopic features that support this diagnosis  Level Microscopic findings Significance  Low-power overview • Irregular, downward-growing epithelial tongues and islands penetrating the supporting connective tissue.  • Disruption of the normal mucosal architecture and loss of a smooth epithelial–connective tissue interface.  • Desmoplastic (fibrotic) stroma with chronic inflammatory cells around the invading nests. Confirms invasion through the basement membrane, distinguishing carcinoma from surface-confined dysplasia/leukoplakia.  High-power detail • Nests and whorled islands of malignant squamous cells showing central concentric keratinisation (keratin pearls).  • Individual-cell keratinisation and intercellular bridges.  • Marked cytologic atypia: enlarged, pleomorphic, hyperchromatic nuclei; increased N:C ratio; coarse chromatin; occasional prominent nucleoli.  • Frequent and sometimes atypical mitotic figures above the basal layer.  • Dyskeratotic cells scattered throughout the tumour islands. The keratin pearls and intercellular bridges indicate squamous differentiation and, together with the cytologic atypia, define a well-differentiated SCC.  Stromal reaction • Dense collagenous stroma with lymphoplasmacytic infiltrate and focal haemorrhage. Typical host response to an invading carcinoma.  Taken together, the presence of invasive keratinising nests with keratin-pearl formation, pronounced squamous atypia, and stromal invasion unequivocally establish the diagnosis of well-differentiated invasive squamous cell carcinoma. |
| 11.37 | Diagnosis: Submucous fibrosis; Key Microscopic Features: Fibrosis with chronic inflammation and tapered rete ridges. | Histopathological Evaluation  Single Most Likely Diagnosis:  Oral Squamous Cell Carcinoma (OSCC)  Key Microscopic Features Supporting the Diagnosis:  Both low-power and high-power hematoxylin and eosin (H&E) stained images demonstrate classic histopathological features consistent with invasive oral squamous cell carcinoma:  Low Magnification (Image 1):  Dysplastic Epithelium: The epithelium shows architectural disarray, including bulbous rete ridges and loss of maturation.  Invasion of Underlying Connective Tissue: Nests and islands of epithelial cells can be seen penetrating through the basement membrane into the connective tissue.  Keratin Formation: Some of the epithelial islands show central eosinophilic material suggesting keratin pearls, typical of well-differentiated OSCC.  High Magnification (Image 2):  Keratin Pearls: Concentric layers of keratinized cells in the center of epithelial islands, a hallmark of well-differentiated squamous cell carcinoma.  Cellular Atypia: Enlarged, pleomorphic, hyperchromatic nuclei are evident.  Increased Nuclear-Cytoplasmic Ratio: Consistent with malignant transformation.  Desmoplastic Stromal Reaction: Fibrous response surrounding tumor nests, indicative of invasive behavior.  Dyskeratosis: Abnormal keratinization of individual cells within epithelial islands.  Summary:  The combination of dysplastic epithelial changes with invasion into the connective tissue, keratin pearl formation, and cytological atypia strongly supports a diagnosis of well-differentiated squamous cell carcinoma of the oral mucosa. | Histopathological diagnosis  Oral sub-mucous fibrosis (OSMF) – matured/late stage  Key microscopic features seen in the two fields  Site in the section What is seen Why it points to OSMF  Lamina propria / superficial submucosa (both images, more obvious in the high-power picture) Dense, pale-eosinophilic, glassy collagen arranged in thick, parallel and whorled bundles; markedly hypocellular—only scattered, thin, flattened fibroblast nuclei; very few, slit-like blood-vessels; patchy mild chronic inflammatory infiltrate The pathognomonic feature of OSMF is progressive hyalinisation and fibrosis of the juxta-epithelial connective tissue, with depletion of fibroblasts and vascular channels  Overlying stratified squamous epithelium (low-power image) Variable thickness but overall flattened/atrophic rete ridges in areas resting on the hyalinised band; surface shows hyper- or para-keratosis; basal cell crowding with only mild cytologic atypia, no epithelial buds or islands penetrating the lamina propria OSMF often shows epithelial atrophy with or without super-added keratinisation; lack of epithelial invasion rules out an invasive squamous-cell carcinoma  Interface zone A narrow, band-like infiltrate of lymphocytes and a few plasma cells immediately beneath the epithelium A low-grade, chronic juxta-epithelial inflammation is characteristic of OSMF, especially in earlier stages  Putting the features together  The combination of:  glassy, densely collagenised, hypocellular and poorly vascular connective tissue immediately beneath the oral epithelium;  overlying epithelium that is variably thinned/flattened but shows no frank invasion; and  a scant chronic inflammatory cell belt  is diagnostic of oral sub-mucous fibrosis.  No epithelial buds, nests or drop-shaped rete ridges are present to suggest invasive squamous-cell carcinoma, and the fibrosis is far too hyalinised for a simple reactive fibrous hyperplasia, confirming OSMF as the single most appropriate diagnosis. |
| 11.38 | Diagnosis: Submucous fibrosis; Key Microscopic Features: Moderate dysplasia with basal cell hyperplasia and stromal hyalinization. | Histopathological Analysis of Provided Images (H&E stained):  Single Most Likely Diagnosis:  Oral epithelial dysplasia (moderate to severe)  Key Microscopic Features Supporting the Diagnosis:  Architectural Disarray of the Epithelium:  Loss of normal maturation and stratification from basal to superficial layers.  Irregular epithelial stratification, with nuclei appearing at abnormal levels in the epithelium (including in upper third layers).  Elongation and bulbous rete ridges.  Cytological Atypia:  Nuclear pleomorphism: noticeable variation in nuclear size and shape.  Hyperchromatic nuclei: increased staining intensity indicating increased DNA content.  Increased nuclear-to-cytoplasmic ratio.  Prominent nucleoli.  Increased Mitotic Activity:  Presence of mitotic figures not confined to the basal layer — seen in suprabasal layers.  Some atypical mitoses are present (indicative of dysplasia severity).  Loss of Polarity and Cell Cohesion:  Cells show loss of orientation, particularly in basal and parabasal layers.  Dyskeratosis (premature keratinization of individual cells within the spinous layer).  Inflammatory Infiltrate in Underlying Lamina Propria:  Moderate lymphocytic infiltrate noted just beneath the dysplastic epithelium.  Conclusion:  The images show key features of oral epithelial dysplasia, more consistent with a moderate to severe degree due to the extent of architectural and cytological abnormalities extending beyond the lower third of the epithelium. These changes are often seen in precancerous lesions such as erythroplakia or leukoplakia with dysplastic transformation. Close clinical correlation and potentially further diagnostic workup (e.g., immunohistochemistry or molecular markers) are recommended, and such lesions warrant surgical excision or close monitoring due to their risk of progression to squamous cell carcinoma. | Single most likely histopathologic diagnosis  Severe oral epithelial dysplasia (= carcinoma in situ, precursor of oral squamous-cell carcinoma)  Key microscopic features that point to this diagnosis  Alteration What is seen in the photomicrographs Significance  Architectural disturbance • Epithelium is markedly thickened with bulbous/“tear-drop” rete ridges pushing downward.  • Loss of the normal orderly maturation pattern – basal-type cells occupy almost the full epithelial thickness. Dysplasia extends beyond the lower ⅔ of the epithelium, fulfilling the criterion for severe dysplasia / CIS.  Cytologic atypia (full-thickness) • Cells throughout the epithelium (including superficial layers) show enlarged, hyperchromatic, pleomorphic nuclei with increased N:C ratio.  • Nuclear membrane irregularity and coarse chromatin are readily appreciable at both magnifications. Confirms dysplasia is not confined to basal layers; involvement of upper thirds is a hallmark of severe dysplasia/CIS.  Mitoses above the basal layer, some abnormal Mitotic figures (including atypical forms) are present well into the spinous layer. Suprabasal and abnormal mitoses are classic criteria for high-grade dysplasia.  Dyskeratosis & epithelial keratin pearls Individual prematurely keratinising cells and small whorled “pearl-like” foci are seen within the epithelium. Premature/aberrant keratinisation is another cytologic criterion of severe dysplasia.  Loss of polarity Nuclei of basal and parabasal cells lose their uniform, palisaded orientation and become vertically (haphazardly) oriented. Reflects disturbed maturation and high-grade dysplasia.  Intact epithelial–connective-tissue interface No epithelial islands, cords or single cells breach the basement membrane; the lamina propria contains only chronic inflammatory cells. Absence of invasion distinguishes carcinoma in situ/severe dysplasia from frankly invasive squamous-cell carcinoma.  Taken together, the full-thickness epithelial atypia with architectural and cytologic abnormalities but no stromal invasion squarely supports a diagnosis of severe oral epithelial dysplasia (carcinoma in situ). |
| 11.41 | Diagnosis: Keratinizing Well-Differentiated Squamous Cell Carcinoma; Key Microscopic Features: Tumor deeply infiltrates the tongue musculature; keratin pearls with intraepithelial microabscesses and a foreign body reaction to keratin. | Histopathological Diagnosis:  Invasive Squamous Cell Carcinoma (SCC) of the oral mucosa.  Key Microscopic Features Justifying the Diagnosis:  Low Magnification (First Image):  Architectural Disruption:  The normal stratified squamous epithelium is breached.  Invasive nests and islands of epithelial cells extend deeply into the underlying connective tissue, suggesting invasion beyond the basement membrane.  Irregular Epithelial Proliferation:  Disorganized epithelial structures, lacking typical maturation.  Invasive epithelial cells with keratinization are visible in the deeper layers.  Desmoplastic Stromal Reaction:  Dense fibrotic stroma surrounding tumor nests — a typical host response to carcinoma invasion.  High Magnification (Second Image):  Malignant Cytologic Features:  Enlarged, hyperchromatic, pleomorphic nuclei.  Prominent nucleoli and high nuclear-to-cytoplasmic (N:C) ratios.  Keratin Pearls:  Concentric layers of keratinizing squamous cells in the tumor nests — a hallmark of well-differentiated SCC.  Individual Cell Keratinization:  Dysplastic squamous cells exhibiting premature keratinization.  Abnormal Mitoses and Cellular Atypia:  Increased mitotic figures, some atypical — indicative of active proliferation and malignancy.  Inflammatory Infiltrate:  Lymphoplasmacytic infiltrate surrounding tumor islands, common in invasive SCC.  Conclusion:  The combination of infiltrative epithelial nests with keratin pearl formation, nuclear atypia, and evidence of stromal invasion confirms the diagnosis of invasive squamous cell carcinoma. The features rule out pre-malignant lesions like leukoplakia, erythroplakia, and dysplasia, which lack definitive stromal invasion. | Histopathological diagnosis  Invasive, well-differentiated (keratinising) squamous cell carcinoma of the oral mucosa  Key microscopic features supporting this diagnosis  Field Observations Diagnostic significance  Low-power (first slide) • Surface stratified squamous epithelium is breached by multiple irregular tongues, nests and cords of epithelium that extend deeply into the lamina propria and submucosa.  • The infiltrative fronts are haphazard and asymmetric, not following the normal rete-ridge pattern. Confirms invasion beyond the basement membrane—essential for calling carcinoma rather than dysplasia.  Tumour architecture • Conspicuous keratin pearls—concentric whorls of eosinophilic keratin within tumour nests.  • Individual cell keratinisation and dyskeratosis. Keratin production denotes squamous differentiation and, when abundant, places the tumour in the “well-differentiated” category.  Cytologic atypia (high-power, second slide) • Large, polygonal tumour cells with dense eosinophilic cytoplasm.  • Marked nuclear pleomorphism, hyperchromasia, irregular nuclear membranes and coarse chromatin.  • Occasional atypical mitotic figures. Confirms malignancy and squamous phenotype.  Intercellular bridges Delicate desmosomal connections between adjacent tumour cells can be appreciated in several nests. Classic feature of squamous epithelium, reinforcing the diagnosis.  Stromal response & host reaction • Desmoplastic fibrous stroma encasing tumour islands.  • Dense mixed inflammatory infiltrate (predominantly lymphocytes and plasma cells) surrounding and permeating the nests. Typical accompanying response in invasive oral SCC.  Depth of invasion Tumour extends well beyond the superficial lamina propria into deeper fibrous and adipose connective tissue. Separates invasive carcinoma from in-situ disease or severe dysplasia.  Taken together, the combination of an invasive growth pattern, abundant keratin pearl formation, cytologic atypia and intercellular bridges is diagnostic of keratinising (well-differentiated) squamous cell carcinoma in the oral cavity. |
| 11.42 | Diagnosis: Keratinizing Well-Differentiated Squamous Cell Carcinoma; Key Microscopic Features: Tumor exhibits keratin pearl formation, moderate pleomorphism, and dyskeratosis. | Single Most Likely Histopathological Diagnosis:  Well-Differentiated Squamous Cell Carcinoma (SCC)  Key Microscopic Features Supporting This Diagnosis:  Invasive Growth Pattern:  The neoplastic squamous cells breach the basement membrane and infiltrate the underlying connective tissue, a hallmark of invasive carcinoma.  Irregular nests and islands of atypical squamous cells are seen deep in the stroma.  Keratin Pearl Formation:  Concentric, eosinophilic, laminated structures within tumor cell nests are visible, representing keratin pearls — a feature of well-differentiated SCC.  Cellular Atypia:  Enlarged, pleomorphic, and hyperchromatic nuclei.  Prominent nucleoli and increased nuclear-to-cytoplasmic ratio.  Mitotic Figures:  Atypical mitoses are present, supporting active and disordered proliferation.  Inflammatory Infiltrate:  Dense lymphoplasmacytic inflammatory infiltrate in the subepithelial stroma, often associated with SCC and a host immune response.  Dysplastic Surface Epithelium:  The overlying epithelium shows dysplastic changes such as nuclear pleomorphism, hyperchromatism, and loss of normal stratification, often adjacent to areas of invasion.  Summary:  These features — especially invasion of atypical squamous cells into the connective tissue, keratin pearl formation, and cytologic atypia — confirm the diagnosis of well-differentiated squamous cell carcinoma, which is a malignant epithelial neoplasm commonly arising from dysplastic oral mucosa. | Single most likely histopathological diagnosis  Invasive, keratinising (well-differentiated) oral squamous-cell carcinoma  Key microscopic features that support this diagnosis  Feature (seen in these H-E sections) Why it points to invasive SCC  Irregular nests, islands and cords of squamous epithelium deeply seated in the connective tissue stroma, clearly detached from the surface mucosa Confirms breach of the basement membrane and true invasion rather than intra-epithelial dysplasia alone  Keratin pearls – concentric lamellated, eosinophilic material in the centre of several tumour islands Hallmark of a keratinising / well-differentiated variant of SCC  Individual-cell keratinisation and intercellular “prickles” within tumour nests Further evidence of squamous differentiation  Cytological atypia: pleomorphic, enlarged, hyperchromatic nuclei, coarse chromatin, prominent nucleoli, increased nuclear-to-cytoplasmic ratio Establishes malignancy rather than a reactive process  Frequent and occasionally abnormal mitotic figures within the tumour nests Indicates high proliferative activity typical of carcinoma  Desmoplastic stroma and brisk lymphoplasmacytic infiltrate surrounding the invasive nests A common stromal reaction to invasive SCC  Surface epithelium shows dysplasia (disturbed maturation, bulbous rete ridges) but the most critical finding is invasion beneath it Links the lesion to the leukoplakia/erythroplakia–dysplasia–carcinoma spectrum, and confirms the “carcinoma” end of that spectrum  Taken together, the combination of invasion, keratin pearl formation, cytologic atypia and mitotic activity is diagnostic of an invasive, keratinising oral squamous-cell carcinoma, most consistent with a well-differentiated grade. |
| 11.43 | Diagnosis: Minimally Keratinizing Moderately Differentiated Squamous Cell Carcinoma; Key Microscopic Features: Tumor forms minimal keratin and exhibits atypical squamous cells with dyskeratosis, without keratin pearl formation. | Based on the histopathological analysis of the provided hematoxylin and eosin (H&E) stained images at different magnifications, the single most likely diagnosis is:  Poorly Differentiated Squamous Cell Carcinoma (SCC)  Key Microscopic Features Supporting the Diagnosis:  Low Magnification (First Image):  Ulcerated surface epithelium with loss of normal architecture.  Infiltrative growth pattern of the lesion extending deep into the underlying stroma.  A dense cellular mass without keratinized or stratified squamous epithelium seen over much of the lesion, suggesting invasion.  Intermediate Magnification (Second Image):  Cellular pleomorphism: Marked variation in nuclear size and shape.  Loss of epithelial polarity and architectural disorganization.  Mitotic figures, some atypical.  Presence of tumor nests or sheets of malignant squamous cells, although keratin pearls are not prominent, indicating poor differentiation.  High Magnification (Third Image):  High nuclear-to-cytoplasmic ratio with hyperchromatic nuclei.  Prominent nucleoli.  Frequent and abnormal mitoses.  Dyscohesive tumor cells in a desmoplastic stroma, typical of high-grade invasive squamous cell carcinoma.  Lack of maturation and keratinization, consistent with poor differentiation.  Conclusion:  The lesion is a poorly differentiated squamous cell carcinoma, as indicated by the highly atypical cellular morphology, infiltrative growth pattern, lack of keratinization, and significant cytologic atypia. These features rule out dysplasia or in situ carcinoma and point toward an aggressive, invasive malignancy. | Histopathological diagnosis  Invasive (keratinising) oral squamous-cell carcinoma – moderately differentiated  Key microscopic features in the three fields that point to this diagnosis  Image Field description Diagnostic features present  1 – low power Broad, exophytic mucosal plaque with surface epithelium at the top; beneath it a large, irregular, nodular mass penetrates the lamina propria and deeper tissues. • Clear invasion of neoplastic epithelium through the epithelial–connective-tissue interface into the underlying stroma.  • Tumour front is jagged and pushing, typical of an infiltrative carcinoma.  • Desmoplastic stromal response around the tumour nests.  2 – intermediate power Tumour nests/whorls surrounded by fibrous stroma. • Keratin pearl formation (concentric whorl of squamous cells with central eosinophilic keratin).  • Cells show intercellular bridges and abundant eosinophilic cytoplasm, proving squamous differentiation.  • Marked cytologic atypia – pleomorphic, hyperchromatic nuclei, increased nuclear-to-cytoplasmic ratio, prominent nucleoli.  • Brisk mitotic activity, some atypical mitoses.  3 – high power / less-differentiated focus More discohesive, sheet-like tumour cells with scant keratinisation. • Marked nuclear pleomorphism and hyperchromasia.  • Loss of orderly maturation and reduced keratin production, indicating areas of poorer differentiation within the same tumour.  • Irregular, haphazard tumour–stroma interface with individual cell infiltration.  Summary of defining features  Invasive growth of epithelial cells beyond the basement membrane into connective tissue.  Squamous differentiation manifested by intercellular bridges and keratin pearls.  Cytologic malignancy – pleomorphism, hyperchromatic enlarged nuclei, numerous mitoses.  Desmoplastic stroma accompanying and surrounding invasive nests.  Together these findings fulfil the criteria for a moderately differentiated keratinising squamous-cell carcinoma of the oral mucosa. |
| 11.44 | Diagnosis: Nonkeratinizing Poorly Differentiated Squamous Cell Carcinoma; Key Microscopic Features: Tumor arises from surface dysplasia, and does not form keratin, showing poorly differentiated and pleomorphic cells. | Based on the provided hematoxylin and eosin (H&E) stained histological images at low and high magnification, the single most likely histopathological diagnosis is:  Squamous Cell Carcinoma (SCC) of the oral cavity  Key Microscopic Features Supporting the Diagnosis:  Low Magnification (Image 1):  Invasive growth pattern:  Irregular nests and cords of epithelial cells infiltrating the underlying connective tissue stroma, breaking through the basement membrane — a hallmark of malignancy.  Keratinization and dysplasia:  Evidence of keratin pearls or individual cell keratinization within nests of squamous cells.  Dysplastic changes in the overlying epithelium, including nuclear hyperchromasia and disordered stratification.  High Magnification (Image 2):  Marked cellular atypia:  Large, pleomorphic, hyperchromatic nuclei.  Irregular nuclear contours and increased nuclear-to-cytoplasmic ratio.  Abnormal mitotic figures:  Frequent, often atypical mitoses indicating high proliferative activity.  Desmoplastic stromal response:  The surrounding stroma shows a reactive fibrotic/desmoplastic response, typical of invasive SCC.  Intercellular bridges:  Present among tumor cells, characteristic of squamous differentiation.  Conclusion:  The histopathological findings — including invasive epithelial nests with keratinization, significant nuclear atypia, increased mitoses, and stromal invasion — are diagnostic of invasive squamous cell carcinoma. These features distinguish it from precursor lesions like leukoplakia, erythroplakia, or dysplasia, which lack stromal invasion. | Histopathological diagnosis  Poorly-differentiated (high-grade) invasive squamous cell carcinoma of the oral mucosa.  Key microscopic features that support the diagnosis  Field Observation Diagnostic significance  Surface epithelium Irregular, bulbous rete ridges with full-thickness loss of maturation, nuclear hyperchromasia and crowding Indicates severe epithelial dysplasia; carcinoma in situ component  Basement membrane Clearly breached; cohesive nests and broad sheets of epithelium are detached from the surface and extend deep into the lamina propria Confirms invasion, elevates the lesion from “dysplasia” to carcinoma  Invasive tumour Sheets of markedly atypical cells with scant keratinisation; many cells are pleomorphic or bizarre, including multinucleated tumour giant cells; abundant atypical mitoses Marked cytologic atypia and minimal keratin production establish a poorly-differentiated grade  Stroma Desmoplastic reaction with mixed inflammatory infiltrate and focal tumour necrosis Common stromal response to an aggressive SCC  Keratin production Only focal “single-cell” keratinisation; no well-formed keratin pearls Lack of maturation typical of high-grade SCC  Taken together, the combination of epithelial dysplasia on the surface and deeply infiltrative, highly pleomorphic, minimally keratinising tumour cells is characteristic of a poorly differentiated invasive squamous cell carcinoma rather than leukoplakia, erythroplakia, or non-invasive epithelial dysplasia. |
| 11.45 | Diagnosis: Keratinizing Moderately Differentiated Squamous Cell Carcinoma; Key Microscopic Features: No description available. | Histopathological Diagnosis:  Squamous Cell Carcinoma (SCC) of the oral cavity  Key Microscopic Features Justifying the Diagnosis:  The images provided (low to high magnification H&E-stained sections) reveal classic hallmarks of squamous cell carcinoma. Here's a breakdown of the key features observed across the three magnifications:  1. Low Magnification (Image 1):  Exophytic and endophytic growth pattern: The lesion shows irregular invasion into the underlying connective tissue and muscle, which is characteristic of malignancy.  Loss of normal epithelial architecture: The stratified squamous epithelium shows disruption of layering and broad tongues or nests invading deeper tissue.  Stromal reaction: Desmoplastic response and possible inflammatory infiltrate at the invasive front.  2. Intermediate Magnification (Image 2):  Nests and islands of malignant squamous cells: These infiltrate the connective tissue, with irregular, angulated contours.  Keratin pearl formation: Suggestive of squamous differentiation, a hallmark of well to moderately differentiated SCC.  Prominent nuclear atypia: Hyperchromatic, pleomorphic nuclei with increased nuclear-to-cytoplasmic ratio.  Dyskeratosis: Abnormal keratinization within individual tumor cells, occurring prematurely.  Mitotic figures: Some abnormal mitotic activity, suggesting high proliferation.  3. High Magnification (Image 3):  Severe cytologic atypia: Markedly pleomorphic nuclei, coarse chromatin, prominent nucleoli.  Increased mitotic activity, including abnormal mitoses.  Intercellular bridges may be visible in areas—another feature of squamous origin.  Loss of cellular cohesion and nuclear crowding.  Conclusion:  The combination of invasive epithelial nests, keratinization (including keratin pearls), nuclear atypia, and desmoplastic stromal response strongly supports a diagnosis of oral squamous cell carcinoma (SCC). This is a malignant neoplasm derived from the squamous epithelium of the oral mucosa and is the most common malignancy in this location. | Histopathological diagnosis  Invasive (moderately-to-poorly differentiated) squamous cell carcinoma of the oral mucosa  Key microscopic features that support this diagnosis  Field (magnification) Observations Diagnostic significance  Low power (whole-mount) ✔ Broad, ulcerated/eroded epithelial surface  ✔ Numerous irregular, tongue-like nests and cords of epithelium breaching the epithelial–connective-tissue junction and infiltrating deeply into the fibromuscular stroma Confirms invasion beyond the basement membrane – the sine-qua-non of carcinoma  Intermediate power ✔ Jagged epithelial islands surrounded by desmoplastic stroma and a lymphoplasmacytic infiltrate  ✔ Loss of orderly stratification; maturation disturbance from basal to superficial layers Architecture typical of invasive squamous carcinoma and host stromal reaction  High power ✔ Marked cytologic atypia: pleomorphic, enlarged, hyperchromatic nuclei with coarse chromatin, irregular nuclear membranes, and conspicuous nucleoli  ✔ High nuclear-to-cytoplasmic ratio and numerous abnormal/atypical mitotic figures  ✔ Foci of individual cell keratinisation and intercellular bridges, but scant keratin-pearl formation → indicates moderate-to-poor differentiation Cytologic criteria of malignancy in squamous lineage; degree of keratinisation allows grading  Additional supportive findings visible across the sections  Surface epithelium shows severe dysplasia / carcinoma in situ contiguous with the invasive component, a common scenario in oral SCC.  Stromal desmoplasia, neo-angiogenesis, and an accompanying chronic inflammatory infiltrate further corroborate an invasive malignant epithelial process.  Taken together, the constellation of deep epithelial invasion, destructive infiltrative growth, and marked squamous cytologic atypia unequivocally establishes the diagnosis of invasive squamous cell carcinoma rather than mere dysplasia, leukoplakia, or erythroplakia. |
| 11.46 | Diagnosis: Keratinizing Well-Differentiated Squamous Cell Carcinoma; Key Microscopic Features: Tumor invades in irregularly shaped islands with central abscesses; many eosinophils present, with desmoplasia noted around the tumor islands. | Based on the provided hematoxylin and eosin (H&E) stained histological images of an oral lesion at low and high magnifications, the single most likely histopathological diagnosis is:  Oral Squamous Cell Carcinoma (OSCC)  Key Microscopic Features Supporting the Diagnosis:  Low Magnification (First Image):  Invasive Growth Pattern:  Nests and islands of atypical squamous epithelial cells are seen infiltrating the underlying connective tissue.  The normal epithelial-connective tissue boundary is disrupted, a hallmark of invasive carcinoma.  Keratin Pearls:  Eosinophilic concentric lamellated structures within tumor nests indicating squamous differentiation.  Desmoplastic Stromal Reaction:  The surrounding stroma shows a reactive fibrous tissue response to invasion, typical in OSCC.  Inflammatory Infiltrate:  Dense lymphoplasmacytic infiltrate around tumor islands, often seen in oral SCC.  High Magnification (Second Image):  Marked Cytologic Atypia:  Tumor cells display pleomorphism, hyperchromatic and irregular nuclei, and increased nuclear-to-cytoplasmic ratio.  Abnormal Mitoses:  Atypical mitotic figures are present, consistent with high proliferative activity.  Individual Cell Keratinization:  Eosinophilic cytoplasm and formation of keratin within individual tumor cells, typical for well to moderately differentiated SCC.  Loss of Cellular Polarity:  Disorganized architecture, lack of maturation toward the surface — features of malignancy.  Conclusion:  The infiltrative pattern of atypical squamous cells with keratin pearl formation, cytologic atypia, and desmoplastic response definitively points toward Oral Squamous Cell Carcinoma, which is the most common malignant tumor of the oral cavity. | Histopathological diagnosis (single best):  Invasive well-differentiated (keratinising) squamous cell carcinoma of the oral mucosa.  Key microscopic features that support this diagnosis  Field Features seen in the provided H&E sections Diagnostic value  Architecture • Ragged nests, tongues and irregular cords of epithelium breaching the basement membrane and infiltrating the connective tissue stroma.  • Tumour islands set in a reactive, fibro-inflammatory stroma rich in lymphocytes and plasma cells. Confirms true invasion, separating carcinoma from mere dysplasia/leukoplakia.  Keratinisation • Conspicuous concentric keratin pearls within tumour nests.  • Numerous individual-cell (dyskeratotic) keratinisations scattered through the tumour. Hallmark of well-differentiated SCC.  Cytology • Large polygonal cells with abundant dense eosinophilic cytoplasm.  • Marked nuclear pleomorphism, hyperchromasia, coarse chromatin, and prominent nucleoli.  • Abnormal and occasional atypical mitotic figures. Confirms malignant squamous phenotype and high-grade atypia.  Intercellular bridges Delicate desmosomal bridges visible between adjacent tumour cells, best appreciated at higher power. Further evidence of squamous differentiation.  Host response • Desmoplastic stromal reaction surrounding invasive nests.  • Dense chronic inflammatory infiltrate (lymphocytes ± plasma cells) abutting tumour fronts and cuffing vessels. Common stromal/immune response to invasive SCC.  Together, the invasive growth pattern, prominent keratin pearl formation, squamous cytomorphology with pleomorphism, and stromal response definitively establish the lesion as an invasive, well-differentiated squamous cell carcinoma. |
| 11.47 | Diagnosis: Keratinizing Well-Differentiated Squamous Cell Carcinoma; Key Microscopic Features: A cup-shaped, umbilicated tumor with a central keratin-filled cavity; papillary projections from epithelium, pleomorphic nuclei, abnormal mitotic figures, and eosinophils. | Histopathological Analysis of Provided H&E-Stained Slides  Single Most Likely Diagnosis:  Squamous Cell Carcinoma (SCC) of the Oral Cavity  Key Microscopic Features Supporting Diagnosis:  Invasive Growth Pattern  Seen especially in the first and third images, there is clear infiltration of atypical epithelial islands into the underlying connective tissue (stroma), breaching the basement membrane — hallmark of carcinoma rather than dysplasia.  Keratin Pearl Formation  Notably present in the first image at lower magnification, as well as in the fourth image. These concentric layers of keratinized cells are diagnostic of well-differentiated SCC.  Nuclear Pleomorphism and Hyperchromatism  Second and fourth images at high magnification show markedly pleomorphic nuclei, hyperchromatic staining, and increased nuclear-to-cytoplasmic ratio — indicative of malignancy.  Atypical Mitoses and High Mitotic Activity  Evident particularly in the second and fourth images, suggesting increased proliferative activity consistent with carcinoma.  Desmoplastic Stromal Response  In the third image, there is a dense fibrotic reaction surrounding invasive tumor islands, a common feature in SCC.  Inflammatory Infiltrate  Chronic inflammatory cells (primarily lymphocytes) are seen scattered around tumor nests, especially in the second and third images. This is common in oral SCC due to chronic irritation and immune response.  Summary:  These images, across different magnifications, demonstrate classic features of oral squamous cell carcinoma, particularly the well-differentiated subtype due to the presence of keratin pearls and some degree of squamous differentiation. The invasive nature and cytological atypia confirm malignancy and rule out precancerous lesions like leukoplakia or dysplasia alone. | Histopathological diagnosis  Well-differentiated (keratinising) invasive squamous cell carcinoma of the oral mucosa.  Key microscopic features supporting this diagnosis  Feature Where it is seen Significance  Irregular epithelial nests and cords penetrating the connective-tissue stroma Low-power image #1 (left half and lower right quadrant) Confirms breach of the basement membrane → invasion (distinguishes carcinoma from dysplasia/leukoplakia).  Keratin pearls (concentric whorls of eosinophilic keratin within tumour nests) Images #3 & #4 (central and lower right) Hallmark of a well-differentiated keratinising SCC.  Individual cell keratinisation & intercellular bridges High-power images #2 & #4 Indicates squamous differentiation.  Marked cytologic atypia – enlarged, pleomorphic, hyperchromatic nuclei; prominent nucleoli; increased mitoses (some atypical) Best appreciated in image #2 Malignant cytology.  Desmoplastic, inflamed tumour stroma – dense fibrous reaction with brisk mixed chronic-acute inflammatory infiltrate Images #2–4 Typical host response to invading SCC.  Surface ulceration with neutrophilic exudate High-power image #2 (upper left) Common in ulcerated oral SCC lesions.  Synthesis  The combination of frank stromal invasion by atypical squamous epithelium, formation of keratin pearls, and pronounced squamous cytologic features establishes a diagnosis of invasive – and because of the abundant keratin production, well-differentiated – squamous cell carcinoma, rather than epithelial dysplasia, leukoplakia, or verrucous carcinoma. |
| 11.49 | Diagnosis: Well-Differentiated Oral Squamous Cell Carcinoma, Keratoacanthomatous Pattern; Key Microscopic Features: Pale cells with broad pushing front; abundant glassy cytoplasm, basal cell hyperplasia, and hyperchromatic nuclei. | Based on the sequential hematoxylin and eosin (H&E) stained histological images provided at varying magnifications, the single most likely histopathological diagnosis is:  Invasive Squamous Cell Carcinoma (SCC) of the oral cavity.  Key Microscopic Features Justifying the Diagnosis:  1. Invasive Growth Pattern:  The first and second images (low to intermediate magnification) show epithelial nests and cords invading the underlying stroma, breaching the basement membrane — a hallmark of malignancy in squamous epithelium.  There is evidence of irregular epithelial projections extending deep into the connective tissue.  2. Keratin Pearl Formation:  Seen in the third image (intermediate magnification), the presence of keratin pearls (concentrically layered keratinized cells within tumor nests) is a classic feature of well-differentiated SCC.  3. Cellular Atypia and Pleomorphism:  The high magnification image shows:  Marked nuclear pleomorphism – variation in size and shape of nuclei.  Hyperchromatic nuclei – darkly stained due to dense chromatin.  Increased nuclear-to-cytoplasmic ratio.  Abnormal mitotic figures and atypical mitoses, supporting malignancy.  4. Desmoplastic Stromal Reaction:  Evident stromal response to invasion with fibroblastic proliferation and inflammatory infiltrate.  5. Inflammatory Infiltrate:  Dense infiltration of lymphocytes and plasma cells within the stroma and around tumor nests, commonly seen in SCC.  Conclusion:  The combination of invasion, keratin pearl formation, and cytologic atypia with mitotic activity strongly supports a diagnosis of Invasive Squamous Cell Carcinoma, likely moderately to well-differentiated, depending on the degree of keratinization and cytologic features across the lesion.  Let me know if you need a grading suggestion or differential diagnosis. | Histopathological diagnosis – single best answer  Invasive keratinising (moderately-differentiated) squamous cell carcinoma of the oral mucosa  Key microscopic features that support this diagnosis  Magnification Salient findings Diagnostic significance  Low-power overview (first image) • Thick, irregular, bulbous tongues of epithelium breach the epithelial–connective-tissue interface and extend deep into the lamina propria and muscle.  • Both endophytic and exophytic (papillary/verruciform) growth present. Definitive evidence of invasion beyond the basement membrane – separates carcinoma from epithelial dysplasia or leukoplakia alone.  Intermediate power (second image) • Nests, cords and broad sheets of atypical squamous cells embedded in a desmoplastic stroma.  • Several nests show concentric eosinophilic lamination forming keratin pearls. Keratin-pearl formation and intercellular bridges confirm squamous differentiation and a well-/moderately-differentiated grade.  High-power fields (third & fourth images) • Tumour cells exhibit marked cytologic atypia: pleomorphic, hyper- to vesicular nuclei, coarse chromatin, irregular nuclear contours, prominent nucleoli, ↑ N/C ratio.  • Individual-cell keratinisation and dyskeratosis within nests.  • Frequent mitoses, including atypical forms.  • Dense lymphoplasmacytic inflammatory infiltrate and stromal desmoplasia encircle invading fronts. Cytologic atypia, abnormal mitoses and dyskeratosis are hallmarks of malignancy; inflammatory/desmoplastic response is typical around invasive SCC fronts.  Why this rules-in carcinoma and rules-out the precancerous lesions in the differential  Leukoplakia/erythroplakia and epithelial dysplasia display atypia confined above an intact basement membrane; no destructive stromal invasion or keratin-pearl-forming nests are seen.  The presence of keratinising tumour nests well below the epithelial surface, coupled with obvious cytologic malignancy, fulfils the diagnostic criteria for an invasive, keratinising squamous cell carcinoma. |
| 11.50 | Diagnosis: Early Verrucous Carcinoma; Key Microscopic Features: Exophytic and endophytic papillary proliferation with parakeratin plugging and bulbous rete ridges; mild cytologic atypia observed. | Single Most Likely Histopathological Diagnosis:  Well-Differentiated Squamous Cell Carcinoma (SCC) of the oral cavity.  Key Microscopic Features Justifying the Diagnosis:  1. Invasive Growth Pattern  Seen in low-power images (first and second), there is irregular epithelial proliferation extending downward into the underlying connective tissue—indicative of invasion beyond the basement membrane.  Bulky, exophytic and endophytic growths with finger-like and bulbous rete ridges, lacking normal maturation, support a malignant behavior.  2. Cellular Atypia and Dysplasia  In the higher magnification images (third and fourth), the epithelium displays:  Pleomorphism (variation in nuclear size and shape)  Hyperchromatism (darkly stained nuclei)  Increased nuclear-to-cytoplasmic ratio  Disordered maturation and loss of normal epithelial stratification.  3. Keratinization and Keratin Pearls  Evidence of keratin pearl formation within islands of malignant squamous cells (seen particularly in the third image) is a hallmark of well-differentiated SCC.  Individual cell keratinization is also present.  4. Desmoplastic Stromal Reaction and Inflammatory Infiltrate  The fourth image shows dense lymphoplasmacytic infiltrates in the stroma.  Peritumoral inflammation and stromal response (desmoplasia) around tumor nests are characteristic of invasive SCC.  5. Mitotic Figures and Atypical Mitoses  High-power views reveal frequent and atypical mitotic figures, further indicating malignancy.  Summary Statement:  The combination of epithelial invasion, cytologic atypia, keratin pearl formation, and inflammatory/desmoplastic stromal response confirms the diagnosis of well-differentiated squamous cell carcinoma—a common malignancy of the oral mucosa typically arising from premalignant lesions like leukoplakia or erythroplakia. | Histopathological diagnosis – Verrucous carcinoma (low-grade variant of squamous cell carcinoma)  Key microscopic features that support this diagnosis  Field Observations in the provided photomicrographs Diagnostic significance  Architecture (low-power view) • Exophytic, warty/verrucous surface with thick hyper- or parakeratinized papillations.  • Broad, bulbous, “pushing” rete pegs that extend deeply but maintain a smooth front rather than breaking up into jagged invasive nests. Classic silhouette of verrucous carcinoma: a heavily keratinised, papillary surface riding on wide, bulldozing epithelial fronts.  Cytology (intermediate & high power) • Epithelium is well-differentiated: cells retain abundant eosinophilic cytoplasm, intercellular bridges and orderly maturation.  • Nuclear pleomorphism is minimal; mitoses are infrequent and confined to basal layers. The very mild atypia distinguishes verrucous carcinoma from conventional invasive SCC, which shows marked cytologic dysplasia.  Keratin production • Numerous surface keratin plugs and parakeratin-filled clefts/crypts.  • Foci of intra-epithelial keratin (keratin pearls) without destructive invasion. Heavy keratin production with limited atypia is typical of verrucous carcinoma.  Pattern of invasion • Basement membrane appears maintained; the epithelial front “pushes” into the lamina propria rather than infiltrating as small, angulated nests. Pushing/non-infiltrative interface is a key criterion separating verrucous carcinoma from conventional SCC.  Stromal response • Dense chronic inflammatory infiltrate with lymphoid aggregates immediately beneath the epithelial front; occasional neutrophilic micro-abscesses within crypts. A brisk but non-destructive host response is frequently seen in verrucous carcinoma.  Absence of high-grade dysplasia • No conspicuous loss of polarity, no marked nuclear hyperchromasia or high mitotic index. Confirms that this is not high-grade epithelial dysplasia or conventional invasive SCC.  Taken together, the exophytic verrucous growth, broad pushing rete ridges, excellent cytologic maturation with minimal atypia, abundant keratin and the lack of destructive invasion firmly support the diagnosis of verrucous carcinoma of the oral mucosa. |
| 11.52 | Diagnosis: Early Verrucous Carcinoma; Key Microscopic Features: Exophytic and endophytic papillary proliferation with parakeratin plugging and bulbous rete ridges; cells show pale cytoplasm compared to normal epithelium; mild cytologic atypia observed. | Histopathological Analysis of the Provided H&E-Stained Images  Single Most Likely Diagnosis:  Oral Epithelial Dysplasia (Moderate to Severe Dysplasia / Carcinoma in situ)  Key Microscopic Features Justifying the Diagnosis:  Low Magnification (Image 1):  Acanthosis: Thickened epithelium with elongation and bulbous rete ridges.  Loss of Maturation: The orderly maturation pattern of the stratified squamous epithelium is disrupted.  Sharp demarcation from underlying connective tissue; no evident invasion into stroma, ruling out invasive carcinoma.  Intermediate Magnification (Image 2):  Nuclear Pleomorphism: Variation in nuclear size and shape throughout the epithelium.  Hyperchromasia: Intensely stained nuclei, often irregular.  Loss of Polarity: Cells are not aligned in a basal-to-surface orientation.  Basal cell hyperplasia extending into suprabasal layers.  High Magnification (Image 3):  Increased Nuclear-Cytoplasmic Ratio: Nuclei occupy a larger portion of the cell.  Prominent Nucleoli and Abnormal Mitoses: Atypical mitotic figures are seen above the basal layer, a hallmark of dysplasia.  Loss of Intercellular Cohesion: Disorganization and separation of epithelial cells.  Conclusion:  These histologic features are consistent with oral epithelial dysplasia, likely at the moderate to severe end of the spectrum. There is no definitive evidence of stromal invasion, which excludes squamous cell carcinoma at this stage but places the lesion at a significant risk of malignant transformation. | Histopathological diagnosis  ▶︎ Severe oral epithelial dysplasia / carcinoma in situ developing in a leukoplakic plaque.  Key microscopic features that support this diagnosis  Field Findings Significance  Low-power architecture • Markedly thickened, irregular epithelium with broad, drop-shaped rete ridges that push downward but remain cuffed by an intact basement membrane.  • Hyper- and parakeratinised corrugated surface. Architectural disturbance and basal crowding are hallmarks of high-grade dysplasia; absence of epithelial islands within the lamina propria argues against invasive SCC.  Maturation / stratification • Loss of normal maturation — the same atypical cell population spans the full epithelial thickness.  • Abrupt transition to underlying lamina propria. Full-thickness disorder → fulfils the “carcinoma in situ / severe dysplasia” criterion.  Cytologic atypia (medium–high power) • Enlarged, pleomorphic and hyperchromatic nuclei with irregular contours.  • Increased nuclear-to-cytoplasmic ratio.  • Prominent nucleoli and coarse chromatin.  • Numerous mitotic figures, some atypical, present at all epithelial levels.  • Foci of premature (“dyskeratotic”) single-cell keratinisation. The degree and distribution of atypia exceed that of mild–moderate dysplasia.  Basement membrane & stroma • Basement membrane is preserved; no epithelial islands or desmoplastic response in the lamina propria.  • Mild chronic inflammatory infiltrate subjacent to the epithelium. Confirms lesion is intra-epithelial and not yet frankly invasive.  Collectively, the pronounced architectural disturbance plus diffuse cytologic atypia throughout the epithelium—yet without stromal invasion—pinpoints severe epithelial dysplasia / carcinoma in situ as the single most appropriate diagnosis. |
| 11.53 | Diagnosis: Verrucous carcinoma; Key Microscopic Features: Exophytic and endophytic squamous proliferation with keratin plugging, frondlike bulbous rete ridges. There is mild cytologic atypia. The adjacent epithelium exhibits atypical verrucous hyperplasia. | Single Most Likely Histopathological Diagnosis:  Squamous Cell Carcinoma (SCC) of the Oral Cavity  Key Microscopic Features Supporting This Diagnosis:  1. Architectural Disruption (Low Magnification – Images 1 & 2):  Loss of normal epithelial stratification and maturation.  Invasive nests and tongues of epithelial cells infiltrating the underlying connective tissue stroma, a hallmark of malignancy.  Surface ulceration is evident, often seen in invasive SCC.  Dysplastic changes extend beyond the epithelial layer, breaching the basement membrane.  2. Cytological Atypia (High Magnification – Images 3 & 4):  Nuclear pleomorphism: Markedly variable nuclear size and shape.  Hyperchromatism: Deeply stained nuclei indicating increased DNA content.  Increased nuclear-to-cytoplasmic (N:C) ratio.  Atypical mitotic figures and abundant mitoses in inappropriate layers.  Loss of cellular polarity and disordered maturation.  Keratin pearl formation in deeper tissues (seen in some foci), a classic feature of well-differentiated SCC.  3. Inflammatory Background (Image 4):  A prominent lymphoplasmacytic infiltrate in the subepithelial stroma, commonly associated with tumor invasion.  Conclusion:  The combination of epithelial invasion through the basement membrane, severe cytological atypia, mitotic activity, keratinization, and associated stromal inflammation is diagnostic of invasive squamous cell carcinoma of the oral cavity. This distinguishes it clearly from premalignant conditions like dysplasia or erythroplakia/leukoplakia without invasion. | Histopathologic diagnosis (single best answer)  Invasive (moderately differentiated) squamous cell carcinoma of the oral mucosa.  Key microscopic features supporting this diagnosis  Level of magnification What is seen Why it points to carcinoma  Low-power (1st & 2nd images) • Thick, hyper-keratinised surface epithelium with an abrupt “drop-off” of broad, jagged epithelial tongues and isolated epithelial islands deep in the lamina propria.  • Loss of the normal, smooth epithelial–connective-tissue interface. Penetration of epithelial cells beyond the basement membrane → unequivocal invasion. Architectural disturbance rules out mere dysplasia/ CIS.  High-power (3rd & 4th images) • Nests and cords of cells in fibrous stroma, some completely detached from the surface.  • Obvious cytologic atypia: large, pleomorphic, hyperchromatic nuclei, coarse chromatin, irregular nuclear membranes, conspicuous nucleoli.  • Frequent & atypical mitoses at all epithelial levels.  • Eosinophilic cytoplasm with intercellular bridges and focal dyskeratosis → squamous differentiation.  • Peritumoral chronic inflammatory infiltrate and early desmoplasia. Cytologic features meet malignant criteria, and stromal reaction confirms tissue destruction by tumour. Presence of squamous differentiation classifies it as a squamous, not basaloid or undifferentiated, carcinoma.  Bottom line: The combination of invasive growth pattern and marked squamous atypia establishes an invasive squamous cell carcinoma, rather than leukoplakia, erythroplakia, or intra-epithelial dysplasia/carcinoma-in-situ. |
| 11.54 | Diagnosis: Verrucous carcinoma with dysplasia versus bluntly invasive papillary well-differentiated squamous cell carcinoma; Key Microscopic Features: Bulky verrucous epithelial proliferation with marked parakeratosis, parakeratin plugging, and pale cells. There is basal cell hyperplasia, nuclear hyperchromasia, pleomorphism, and dyscohesion. The islands at the base represent tangentially sectioned tips of rete ridges. | Based on the histopathological evaluation of the two provided hematoxylin-and-eosin (H&E) stained images — one at low magnification and the other at high magnification — the single most likely histopathological diagnosis is:  Squamous Cell Carcinoma (SCC) of the Oral Cavity  Key Microscopic Features Supporting This Diagnosis:  1. Invasive Growth Pattern (Low Magnification Image):  The epithelium shows irregular downward proliferation into the underlying connective tissue.  There is loss of normal epithelial architecture, including a disrupted basement membrane, indicating invasion.  The rete ridges are bulbous, fused, and drop-shaped, characteristic of neoplastic invasion.  2. Cytologic Atypia (High Magnification Image):  Marked pleomorphism: Nuclei vary significantly in size and shape.  Hyperchromatism: The nuclei are deeply basophilic, indicating increased DNA content.  Increased nuclear-to-cytoplasmic ratio.  Abnormal mitotic figures are present, some of which may be atypical.  Individual cell keratinization and keratin pearl formation may be noted (though not clearly seen in the current field, it may be present in other areas).  3. Loss of Maturation:  There is a lack of orderly maturation from basal to superficial layers, typical of carcinoma.  Cells appear disorganized, especially in the lower and middle thirds of the epithelium.  4. Stromal Response:  The surrounding stroma shows a desmoplastic reaction and inflammatory infiltrates, further supporting invasion.  Summary:  The presence of epithelial invasion, cytologic atypia, disorganized architecture, and mitotic activity confirms a diagnosis of oral squamous cell carcinoma rather than precancerous lesions like leukoplakia, erythroplakia, or dysplasia alone. | Histopathological diagnosis  Invasive (moderately differentiated) squamous cell carcinoma of the oral mucosa.  Key microscopic features seen in the two fields that underpin the diagnosis  Field Important findings Significance  Low-power overview (first image) • Surface epithelium shows broad, downward-growing, anastomosing cords and nests that clearly breach the basement membrane and extend deep into the connective tissue.  • The invading nests have irregular, jagged borders (not the smooth, pushing pattern of a verrucous lesion).  • Focal keratin debris is present on the surface and within some islands. The combination of an epithelial proliferation that crosses the basement membrane and exhibits an infiltrative architecture proves invasion, separating carcinoma from dysplasia or carcinoma in situ.  High-power detail (second image) • Tumour nests are composed of large polygonal cells with abundant eosinophilic cytoplasm and conspicuous intercellular bridges.  • Marked nuclear pleomorphism, hyperchromasia, coarse chromatin and prominent nucleoli.  • Numerous mitotic figures, including atypical mitoses.  • Foci of individual-cell keratinisation and small keratin pearls. These cytological abnormalities together with intercellular bridges confirm squamous differentiation and the malignant nature of the cells, consistent with a moderately differentiated SCC.  Taken together, the invasive growth pattern and the cytological features of malignant, keratin-producing squamous cells establish the diagnosis of an invasive (moderately differentiated) squamous cell carcinoma. |
| 11.55 | Diagnosis: Verrucous carcinoma with focus of infiltrating squamous cell carcinoma; Key Microscopic Features: Low-power view shows a verrucous carcinoma with parakeratosis, keratin plugs, bulbous rete ridges, and endophytic proliferation of pale epithelial cells. Rete ridges are frondlike and cells are pale with glassy cytoplasm. Base of rete ridges shows early keratin pearl formation with crowding of cells, and there is slight blurring of the epithelial–connective tissue interface. One focus of atypical cells exhibits early microinvasive squamous cell carcinoma. | Single Most Histopathological Diagnosis:  Well-differentiated Squamous Cell Carcinoma (SCC) of the oral cavity  Key Microscopic Features Justifying the Diagnosis:  Architectural Disorganization and Invasion:  The first and second images (low magnification) demonstrate epithelial proliferation extending beyond the basement membrane into the underlying connective tissue, a hallmark of invasive carcinoma.  The lesion shows exophytic and endophytic growth patterns with disrupted epithelial architecture and loss of normal maturation.  Keratin Pearls and Individual Cell Keratinization:  Seen clearly in the third image (intermediate magnification): concentric keratin pearls (squamous differentiation hallmark) and individual cell keratinization are classic for well-differentiated SCC.  Cytological Atypia:  The fourth image (high magnification) highlights marked pleomorphism, hyperchromatic nuclei, increased nuclear-to-cytoplasmic ratio, and prominent nucleoli.  Abnormal mitotic figures are present, indicating increased proliferative activity.  Desmoplastic Stromal Reaction and Inflammatory Infiltrate:  A desmoplastic response in the stroma surrounding the invading tumor islands and sheets is visible, particularly in the deeper sections.  A dense lymphoplasmacytic infiltrate is evident, often accompanying invasive SCC.  Conclusion:  The constellation of features — epithelial invasion, keratin pearl formation, cytological atypia, and stromal response — strongly supports the diagnosis of well-differentiated squamous cell carcinoma of the oral cavity.  2/2  4o | Histopathological diagnosis  → Invasive well-differentiated (keratinising) squamous cell carcinoma of the oral mucosa  Key microscopic features that support the diagnosis  Field of view What is seen Why it points to SCC  Low-power overview (image 1) Broad, irregular, exo-endophytic mass composed of thick squamous epithelium. Numerous bulbous rete ridges and tongues push deep into the underlying connective tissue, breaching the lamina propria. Invasion beyond the epithelial–stromal junction is diagnostic of carcinoma rather than dysplasia or leukoplakia alone.  Intermediate magnification (image 2) Islands and cords of tumour cells are completely separated from the surface epithelium and lie within a desmoplastic stroma; a concentric keratin pearl is visible in one nest. Detached epithelial nests with keratin-pearl formation are classic for well-differentiated keratinising SCC.  High magnification (image 3) Tumour nests show orderly squamous maturation toward the centre with whorled keratin pearls; peripheral cells display intercellular bridges. Nuclei are enlarged, variably shaped, slightly hyperchromatic; occasional mitoses present. Keratin pearls and intercellular bridges indicate squamous differentiation; cytological atypia and mitotic activity confirm malignancy.  Deep invasive front (image 4) Irregular infiltrative strands and small clusters of eosinophilic squamous cells permeate a lymphoplasmacytic stroma. Individual-cell keratinisation (dyskeratosis) and nuclear pleomorphism are evident. Infiltration in a single-cell or small-cluster fashion and dyskeratosis are hallmarks of an invasive SCC front. The brisk inflammatory host response is typical around oral SCC.  Additional corroborative findings  Hyper-/para-keratinised surface with loss of normal epithelial stratification and polarity.  Desmoplastic connective-tissue reaction encasing tumour islands.  Dense lymphoplasmacytic infiltrate at the tumour–host interface.  Taken together, these features meet all criteria for an invasive, well-differentiated (keratinising) squamous cell carcinoma rather than epithelial dysplasia, carcinoma in situ, verrucous carcinoma, or a purely reactive leukoplakic change. |
| 11.56 | Diagnosis: Giant oral condyloma acuminatum; Key Microscopic Features: Exophytic proliferation of mature squamous epithelium with a smooth surface and minimal keratin. Cells exhibit mild epithelial atypia typical for condyloma, with apoptotic cells and many koilocytes. | Histopathological Analysis of Provided H&E-Stained Images  Single Most Likely Histopathological Diagnosis:  Squamous Cell Carcinoma (SCC) of the Oral Cavity  Key Microscopic Features Supporting the Diagnosis:  Low Magnification Image (Image 1):  Architectural Disturbance: There is an invasive, irregular epithelial proliferation that extends deep into the underlying connective tissue, breaching the basement membrane.  Nodular Configuration: Islands and nests of epithelial cells are seen infiltrating the stroma, which is a hallmark of invasive carcinoma.  Desmoplastic Stromal Response: Suggestive fibrous tissue reaction surrounding tumor islands, commonly seen in invasive SCC.  High Magnification Image (Image 2):  Nuclear Pleomorphism: Noticeable variability in nuclear size and shape, with hyperchromatic (darkly stained) nuclei.  Increased Nuclear-Cytoplasmic Ratio: Indicative of malignant transformation.  Keratin Pearl Formation: Central eosinophilic keratin within some tumor islands — a classic feature of well-differentiated squamous cell carcinoma.  Individual Cell Keratinization: Abnormal keratinization within tumor nests.  Mitoses (Including Atypical Forms): Suggestive of high proliferative activity.  Loss of Maturation and Polarity: Disordered stratification of squamous epithelium.  Summary:  The combination of invasive epithelial nests, keratin pearls, marked cellular atypia, and stromal invasion confirms squamous cell carcinoma, most consistent with a well-differentiated type based on the presence of keratin production. | Histopathological diagnosis (single best):  Verrucous carcinoma of the oral mucosa (Ackerman tumour) – a well-differentiated, low-grade variant of squamous-cell carcinoma.  Key microscopic features that point to this diagnosis  Level Feature seen in the photomicrographs Diagnostic significance  Low power (whole-section view) • Very thick, papillated/verruciform surface with deep clefts packed with keratin debris.  • Epithelium forms broad, bulbous “elephant-foot” rete ridges that extend well beyond the level of the adjacent mucosa, but with a smooth, pushing front rather than a jagged infiltrative edge.  • No obvious small, dyscohesive tumour islands in the lamina propria. Classic architectural pattern of verrucous carcinoma – exophytic/verrucous surface + pushing invasion.  Intermediate / high power • Squamous cells show orderly maturation from basal to surface layers; cytologic atypia is minimal – nuclei are mostly uniform, mitoses are scant and confined basally.  • Frequent parakeratin plugging in surface clefts and within the cores of rete ridges.  • Occasional individual cell keratinisation and dyskeratotic cells, but no overtly malignant pleomorphism or abnormal mitoses. Verrucous carcinoma is well-differentiated; the tumour grows deeply but the cells look deceptively bland.  Interface with stroma • Basement-membrane outline is largely preserved; invasion is by broad fronts that “push” into the lamina propria and superficial muscle without destructive infiltration.  • Often an accompanying chronic inflammatory infiltrate around and between the rete pegs (seen as lymphoplasmacytic stroma in the section). Distinguishes verrucous carcinoma from conventional invasive SCC, which shows jagged, irregular nests with marked cytologic atypia.  Taken together, the combination of a verrucous / warty outline, massive acanthosis with parakeratin, broad pushing rete ridges that penetrate deeply, and the conspicuous lack of marked cytologic dysplasia or keratin-pearl–forming infiltrative nests is diagnostic of verrucous carcinoma rather than simple hyperkeratosis/hyperplasia, dysplasia alone, or conventional squamous-cell carcinoma. |
| 11.57 | Diagnosis: Oral papillary squamous cell carcinoma; Key Microscopic Features: Broad-based papillary proliferation of dysplastic epithelium without surface keratin. Papillary carcinoma-in-situ with fibrovascular cores. Dysplastic keratinocytes with foci of squamous cells. Carcinoma-in-situ comprised of full-thickness dysplastic cells. | Histopathological Analysis of Provided Hematoxylin and Eosin-Stained Images  Single Most Likely Histopathological Diagnosis:  Squamous Cell Carcinoma (SCC) of the Oral Cavity  Key Microscopic Features Supporting the Diagnosis:  Architectural Disruption and Invasion:  In the low-power image (Image 1), there's a clear infiltration of epithelial islands into the underlying stroma, breaching the basement membrane, which is diagnostic of malignancy.  Desmoplastic stromal reaction is evident, indicating the body's response to invasive carcinoma.  Keratin Pearl Formation:  Seen prominently in higher magnification (Image 3), these are concentric layers of eosinophilic keratin surrounded by atypical squamous cells, a hallmark of well-differentiated SCC.  Cellular and Nuclear Pleomorphism:  High-grade atypia with pleomorphic, hyperchromatic nuclei and increased nuclear-cytoplasmic ratio.  Prominent nucleoli and irregular nuclear contours are present.  Abnormal Mitoses:  Several atypical mitotic figures are seen, indicative of high proliferative activity.  Loss of Normal Maturation and Stratification:  The epithelium exhibits disordered stratification, with atypical cells extending through the full thickness and into connective tissue (Images 2 and 4).  Dyskeratosis and Individual Cell Keratinization:  Abnormal keratinization within individual tumor cells in deeper epithelial layers (Image 3).  Summary:  These histological findings are diagnostic of invasive squamous cell carcinoma, most likely well-differentiated, given the presence of keratin pearls and intercellular bridges. The images show classical progression from dysplasia to invasion, a hallmark in oral SCC pathogenesis. | Single most appropriate histopathological diagnosis  Severe oral epithelial dysplasia / squamous cell carcinoma in situ (papillary variant)  Key microscopic features that support this diagnosis  Field-of-view What is seen Why it matters for the diagnosis  Low power (whole section) Broad-based, markedly exophytic mass composed of numerous finger-like and club-shaped papillary fronds supported by fibro-vascular cores. Establishes a papillary architecture typical of a verruciform/papillary squamous proliferation rather than a flat leukoplakia.  Intermediate power Thick, bulbous rete ridges that appear “drop-shaped,” crowd and fuse, but stop at an intact epithelial–connective tissue interface (no jagged irregular islands burrowing into the lamina propria). Absence of obvious stromal invasion keeps the lesion on the intra-epithelial side of the spectrum (carcinoma in situ rather than invasive SCC).  High power (images 3 & 4) • Full-thickness cytological atypia: pleomorphic, hyperchromatic nuclei, increased N:C ratio, loss of polarity from basal to superficial layers.  • Abnormal & superficial mitotic figures, including atypical mitoses.  • Dyskeratosis & premature keratin pearl formation entirely within the epithelium. These are the hallmarks of severe dysplasia / CIS — i.e., malignant cytology involving the entire epithelial thickness with disturbed maturation, but without breach of the basement membrane.  Surface maturation Patchy parakeratin and individual cell keratinisation, but overall disordered maturation and loss of the normal stratified sequence. Confirms the dysplastic, rather than reactive, nature of the process.  Because the cytological changes involve the entire epithelial thickness yet stop at an intact basement membrane, the lesion fulfills criteria for carcinoma in situ (severe epithelial dysplasia) rather than invasive squamous-cell carcinoma or a mere low-/moderate-grade dysplasia. The pronounced papillary growth pattern explains the clinically verrucous/leukoplakic appearance that places it in the “leukoplakia, erythroplakia, dysplasia & SCC” clinicopathological group. |
| 11.58 | Diagnosis: Oral papillary well-differentiated squamous cell carcinoma; Key Microscopic Features: Papillary carcinoma with little surface keratinization and many pale epithelial cells. Pale cells with glassy cytoplasm and many intraepithelial abscesses near the base of rete ridges. There are atypical squamous cells, intraepithelial abscesses, and eosinophils in the epithelium and stroma. | Based on the three provided hematoxylin-and-eosin (H&E) stained histopathology images at increasing magnifications, the single most likely histopathological diagnosis is:  Invasive Squamous Cell Carcinoma (SCC) of the oral mucosa.  Key Microscopic Features Supporting the Diagnosis:  1. Architectural Disarray and Invasion (Low Power)  In the first image (lowest magnification), there is irregular epithelial proliferation extending downward into the underlying connective tissue — indicative of epithelial invasion, a hallmark of malignancy.  The architecture is exophytic and endophytic with papillary projections and keratin-filled invaginations.  2. Dysplastic Epithelium with Maturation Disturbance (Intermediate Power)  The second image shows features of severe epithelial dysplasia to carcinoma in situ, including:  Loss of maturation from basal to superficial layers.  Prominent nuclear pleomorphism and hyperchromasia.  Increased and abnormal mitotic figures, some seen away from the basal layer.  Basilar crowding and irregular rete ridge patterns.  3. Definitive Invasion and Keratinization (High Power)  The third image reveals invasive islands and nests of malignant squamous cells breaching the basement membrane.  Presence of keratin pearls (concentric layers of eosinophilic keratin), characteristic of well-differentiated squamous cell carcinoma.  Desmoplastic stromal reaction (fibroblastic proliferation around tumor islands) is also evident.  Dense lymphoplasmacytic inflammatory infiltrate, commonly associated with invasion.  Summary:  These features, especially epithelial invasion, cellular atypia, keratin pearl formation, and stromal desmoplasia, are diagnostic of invasive squamous cell carcinoma, rather than a precancerous condition like leukoplakia, erythroplakia, or oral epithelial dysplasia alone. | Histopathological diagnosis (single best):  Verrucous carcinoma – a well-differentiated, low-grade variant of squamous cell carcinoma.  Key microscopic features seen in the three H-E images  Level of magnification Salient findings Significance for diagnosis  Low-power overview (first image) • Broad, exophytic, verrucous/papillary surface  • Deep, bulbous rete ridges that descend in a smooth, pushing fashion rather than as irregular jagged cords  • Numerous keratin-filled clefts/crypts between projections The “church-spire” papillary surface with pushing borders is classic for verrucous carcinoma and distinguishes it from conventional invasive SCC.  Intermediate view (second image) • Epithelium greatly thickened with orderly maturation from basal to superficial layers  • Abundant surface and intra-epithelial parakeratin with neutrophilic micro-abscesses  • Minimal nuclear pleomorphism, rare mitoses Well-differentiated appearance with scant cytologic atypia despite massive hyperplasia supports a verrucous (rather than dysplastic or frankly malignant) phenotype.  High-power view (third image) • Uniform, relatively bland squamous cells; nuclei are round-to-oval, not markedly pleomorphic  • Occasional keratin pearls/whorls within broad epithelial tongues  • Dense chronic inflammatory infiltrate (lymphocytes, plasma cells ± neutrophils) hugging the advancing front The combination of keratin pearls, bland cytology, and chronic inflammation at a pushing interface is typical of verrucous carcinoma and not of reactive hyperplasia alone.  Diagnostic synthesis  The verrucous / warty architecture, broad pushing rete ridges, and keratin-filled crypts are pathognomonic.  Cytologic atypia is minimal despite massive epithelial proliferation, separating the lesion from conventional well-differentiated (keratinising) SCC.  A dense subjacent inflammatory infiltrate and neutrophilic micro-abscesses in keratin are frequently present in oral verrucous carcinoma.  Together, these features confirm the lesion as verrucous carcinoma rather than leukoplakia with dysplasia, erythroplakia, or invasive conventional squamous cell carcinoma. |
| 11.59 | Diagnosis: Papillary well-differentiated squamous cell carcinoma, mostly bluntly invasive; Key Microscopic Features: Mostly exophytic papillary proliferation of squamous epithelium. There are cells with increased nuclear:cytoplasmic ratio and dyskeratosis, and there is a moderate lymphocytic host response. Some tumor islands are well demarcated from stroma, but there is blurring of the interface at some tips of rete ridges. Keratin pearl at the tips of rete ridges often herald conventional invasion. | Based on the provided hematoxylin-and-eosin (H&E) stained histopathological images captured at different magnifications, the single most likely diagnosis is:  Squamous Cell Carcinoma (SCC) of the Oral Cavity  Key Microscopic Features Supporting This Diagnosis:  Invasive Growth Pattern:  In the low-power image (first image), there is an exophytic and endophytic proliferation of epithelial cells breaching the basement membrane, a hallmark of invasive carcinoma.  Keratin Pearls:  Seen clearly in the high-power image (fourth image), there are concentrically layered eosinophilic keratin deposits within nests of malignant squamous cells, characteristic of well-differentiated squamous cell carcinoma.  Atypical Squamous Epithelial Cells:  Present throughout all magnifications:  Nuclear pleomorphism (variation in nuclear size and shape).  Hyperchromatic nuclei (deeply staining nuclei).  Increased nuclear-cytoplasmic ratio.  Abnormal mitotic figures.  Dysplastic Changes in the Overlying Epithelium:  Loss of normal maturation and stratification.  Presence of atypical cells extending through the full thickness of the epithelium in some areas, consistent with carcinoma in situ adjacent to invasive carcinoma.  Stromal Reaction and Inflammation:  Prominent desmoplastic (fibrous) stromal response around invading nests.  Associated chronic inflammatory infiltrate, particularly lymphocytes, seen in the third image.  Conclusion:  The histologic evidence, particularly the invasive squamous islands with keratin pearl formation and cellular atypia, is diagnostic of oral squamous cell carcinoma (OSCC), specifically well-differentiated SCC due to the presence of keratinization. Early recognition and intervention are critical given the malignant nature and potential for local invasion and metastasis. | Histopathological diagnosis  Invasive well-differentiated (keratinising) squamous cell carcinoma of the oral mucosa.  Key microscopic features visible in the supplied H-&-E images  Field Feature How it supports the diagnosis  Low-power overview • Fungating, papillary/exophytic surface with broad bulbous rete ridges that extend deeply and irregularly into the lamina propria.  • Epithelial proliferation clearly breaches the epithelial–connective-tissue interface. An infiltrative growth pattern confirms invasion rather than purely intra-epithelial dysplasia or hyperplasia.  Tumour islands & cords • Nests, tongues and irregular cords of squamous epithelium are seen infiltrating the connective-tissue stroma.  • Many of these nests show concentric whorls of eosinophilic keratin (“keratin pearls”). Invasion plus keratin-pearl formation is characteristic of a well-differentiated squamous cell carcinoma.  Cytological atypia • Enlarged, pleomorphic, hyperchromatic nuclei with irregular contours.  • Prominent nucleoli and coarse chromatin.  • Numerous, sometimes atypical, mitotic figures. Nuclear pleomorphism and abnormal mitoses indicate malignancy.  Keratinisation • Individual cell keratinisation and intracellular eosinophilic keratin.  • Intercellular bridges still appreciable in many tumour cells. Retention of squamous maturation features (bridges & keratin) supports keratinising SCC and explains the “well-differentiated” grade.  Stromal response • Dense lymphoplasmacytic inflammatory infiltrate and areas of desmoplastic fibrous stroma encasing tumour nests.  • Foci of haemorrhage and vascular congestion. A reactive stromal/inflammatory host response is commonly seen around invasive SCC nests.  Taken together—papillary invasive architecture, unequivocal breach of the basement membrane, keratin-pearl-forming invasive nests, and marked cytological atypia—these findings are diagnostic of an invasive well-differentiated keratinising squamous cell carcinoma rather than leukoplakia, erythroplakia, or non-invasive dysplasia. |
| 11.60 | Diagnosis: Papillary well-differentiated squamous cell carcinoma, bluntly invasive; Key Microscopic Features: Bulky papillary squamous proliferation forming bulbous rete ridges with little surface keratin. Deep epithelial extensions form bulbous rete ridges with hypercellularity and hyperchromasia. There is basal cell hyperplasia but well-differentiated squamous cells are also noted. Basal cell hyperplasia and mitotic figures are present. | Histopathological Analysis of the Provided H&E-Stained Images (Oral Lesion)  Single Most Likely Histopathological Diagnosis:  Oral Squamous Cell Carcinoma (SCC)  Key Microscopic Features Supporting the Diagnosis:  Architectural Disarray and Epithelial Invasion (Image 1 & 2):  Papillary, exophytic growth pattern and broad bulbous rete ridges extending deeply into the connective tissue.  Loss of normal epithelial maturation and polarity.  Invasion beyond the basement membrane into the underlying stroma—hallmark of malignancy.  Cellular Atypia and Dysplasia (Image 3):  Marked nuclear pleomorphism and hyperchromasia.  Prominent nucleoli and increased nuclear-cytoplasmic ratio.  Dyskeratosis: abnormal keratinization within individual cells.  Loss of maturation from basal to superficial layers.  High Mitotic Activity (Image 4):  Numerous and atypical mitotic figures.  Cells display nuclear crowding and loss of normal stratification.  Abnormal mitotic spindles and irregular chromatin clumping.  Inflammatory Response (seen across several images):  Dense chronic inflammatory infiltrate in the underlying connective tissue, often associated with invasive carcinoma.  Differential Consideration Ruled Out:  Leukoplakia or Erythroplakia: These are clinical terms; histologically they can show hyperkeratosis or dysplasia but not definitive invasion.  Oral Epithelial Dysplasia: Although dysplastic features are present, the key differentiator is the invasion of malignant epithelial cells into connective tissue, confirming SCC. | Most likely histopathological diagnosis  → Squamous cell carcinoma in situ (= severe / grade III oral epithelial dysplasia, carcinoma confined to the epithelium).  Key microscopic findings that support this diagnosis  Level of magnification What is seen Why it matters  Low-power overview • Thickened, papillary / verruciform epithelial surface.  • Broad, bulbous, “pushing” rete ridges that descend toward (but do not breach) the lamina propria.  • No discrete tumour nests or cords in the underlying connective tissue. Excludes invasive SCC; shows an in-situ process with epithelial expansion only.  Intermediate power • Loss of the normal maturation gradient – basal-type cells occupy the full epithelial thickness.  • Focal dyskeratosis/keratin whorls within the spinous layer. Full-thickness architectural disorder is the defining feature of carcinoma in situ.  High power • Marked cytologic atypia: enlarged, hyperchromatic, pleomorphic nuclei, irregular nuclear contours, coarse chromatin, occasional nucleoli.  • Numerous mitotic figures (including abnormal mitoses) situated well above the basal layer.  • Increased nuclear-to-cytoplasmic ratio and loss of polarity of the lesional keratinocytes.  • Intact basement membrane with a sharp interface; underlying stroma shows only a reactive chronic‐inflammatory infiltrate. Combines the required cytologic criteria for “severe dysplasia/CIS” with proof that invasion has not yet occurred.  Bottom line: the combination of full-thickness epithelial dysplasia with pronounced cytologic atypia and mitotic activity, yet an intact basement membrane and absence of stromal invasion, squarely places the lesion in the category of squamous cell carcinoma in situ / severe oral epithelial dysplasia, rather than leukoplakia with mild–moderate changes, verrucous carcinoma (which shows bland cytology), or invasive conventional squamous cell carcinoma. |
| 11.62 | Diagnosis: Sarcomatoid squamous cell carcinoma; Key Microscopic Features: Stellate-shaped and spindle cells infiltrating muscle. Pleomorphic epithelioid cells. | Based on the provided hematoxylin and eosin (H&E) stained histopathology images at different magnifications, here's a detailed analysis:  Single Most Likely Histopathological Diagnosis:  Squamous Cell Carcinoma (SCC) – moderately to poorly differentiated  Key Microscopic Features Justifying the Diagnosis:  Low-power view (First image):  Disordered architecture of the epithelial and stromal interface.  Infiltrative growth pattern with cords and nests of atypical epithelial cells invading the stroma.  Loss of normal epithelial maturation, with extension beyond the basement membrane.  Desmoplastic stromal response indicating invasion, which is characteristic of malignancy.  High-power view (Second image):  Marked cellular atypia: Enlarged, hyperchromatic nuclei, irregular nuclear contours, and high nuclear-to-cytoplasmic ratios.  Pleomorphism: Significant variation in cell size and shape.  Abnormal mitotic figures, suggesting increased proliferative activity.  Dyskeratosis and individual cell keratinization within tumor islands, indicative of squamous differentiation.  Some necrotic debris and inflammatory cells in the stroma, supporting tissue damage and invasive malignancy.  Conclusion:  The combination of invasive epithelial nests, cytologic atypia, and desmoplastic response strongly supports a diagnosis of invasive squamous cell carcinoma of the oral cavity. This lesion appears to be at least moderately differentiated, though some areas suggest poor differentiation due to the high degree of pleomorphism and mitotic activity. | Histopathological diagnosis  Sarcomatoid (spindle-cell) variant of invasive squamous cell carcinoma of the oral cavity.  Key microscopic features visible in the two H-E photomicrographs  Field Findings Significance  Low-power overview (first image) • Cellular lesion composed almost entirely of long, sweeping fascicles and whorls of spindle-shaped tumour cells that permeate a collagen-rich, desmoplastic stroma.  • Absence of the usual cohesive nests/keratin pearls seen in conventional well-differentiated SCC. Spindle pattern and desmoplasia are characteristic of the sarcomatoid (spindle-cell) variant and help distinguish it from ordinary SCC or reactive fibrosis.  High-power detail (second image) • Markedly pleomorphic spindle and epithelioid tumour cells with enlarged, irregular, hyperchromatic nuclei and coarse chromatin.  • Frequent atypical mitotic figures and occasional bizarre, multinucleated tumour giant cells.  • Scattered dyskeratotic cells with dense eosinophilic cytoplasm betraying squamous differentiation even though overt keratin pearls are lacking.  • Intercellular bridges/tonofilament-type cytoplasm focally appreciable between some adjacent tumour cells. High-grade cytologic atypia and atypical mitoses confirm malignancy; the focal squamous (epithelial) clues amid a spindle population establish that the process is sarcomatoid carcinoma, not a true sarcoma such as fibrosarcoma or undifferentiated pleomorphic sarcoma.  Stromal / inflammatory background • Dense, eosinophilic, hyalinised collagen bundles around and between tumour fascicles (so-called “desmoplastic reaction”).  • Scattered lymphocytes and plasma cells within the stroma. A fibrosing, inflamed stroma is typical of invasive carcinoma of the mucosa and supports the diagnosis in an oral cavity lesion.  Why this fits sarcomatoid SCC and not its mimics  The entire tumour is epithelial in origin but has undergone a sarcomatoid (mesenchymal-looking) transformation, hence the uniform spindle morphology.  True mesenchymal sarcomas lack any obvious areas or cells showing squamous features (intercellular bridges, dyskeratosis) that are present here.  Reactive fibrous proliferations (e.g., granulation tissue) do not display the degree of nuclear pleomorphism, atypical mitoses, or tumour giant cells seen.  Together, these microscopic findings point to a spindle-cell (sarcomatoid) squamous cell carcinoma, a high-grade, aggressive variant of oral SCC. |
| 11.63 | Diagnosis: Sarcomatoid squamous cell carcinoma; Key Microscopic Features: Nodular proliferation of spindle cells with overlying ulceration; conventional squamous cell carcinoma is not seen. Spindle cells have mild pleomorphism and surrounding edema and granulation tissue easily mistaken for granulation tissue. | Histopathological Diagnosis:  Invasive Squamous Cell Carcinoma (SCC) of the oral cavity  Key Microscopic Features Supporting the Diagnosis:  Low Power Image (First Image):  Invasive architecture: There is a clear infiltration of neoplastic epithelial cells into the underlying connective tissue, disrupting the normal mucosal architecture.  Loss of epithelial maturation and polarity: The overlying epithelium appears irregular and disorganized, with evidence of downward proliferation.  Stromal reaction: A desmoplastic (fibrotic) stromal response is visible around the invasive islands, which is typical of invasive carcinoma.  Keratin pearls and nests: Some rounded nests of malignant squamous cells with central keratinization are identifiable, consistent with SCC.  High Power Image (Second Image):  Cellular atypia: Marked nuclear pleomorphism, hyperchromasia, and prominent nucleoli are seen.  Abnormal mitotic figures: Increased and atypical mitotic activity is evident.  Keratinization: Individual cell keratinization and keratin pearls are identifiable, confirming squamous differentiation.  Invasion into stroma: Malignant epithelial cells have breached the basement membrane and are seen infiltrating the connective tissue.  Conclusion:  The combination of disordered epithelial proliferation, marked cytological atypia, keratinization, and clear evidence of invasion into the connective tissue strongly supports a diagnosis of invasive squamous cell carcinoma of the oral cavity. | Histopathologic diagnosis (single best answer)  Nodular fasciitis – a benign, self-limited myofibroblastic proliferation (also called “pseudosarcomatous fasciitis”).  Key microscopic features visible in the two H-E images  # Finding How it appears in the images & why it supports the diagnosis  1 Well-circumscribed, dome-shaped submucosal nodule On the low-power image the lesion forms a broad, pushing mass beneath intact oral squamous epithelium, lacking an infiltrative jagged border typical of carcinoma.  2 Plump spindle / stellate myofibroblasts in short, irregular fascicles (“tissue-culture” pattern) At higher magnification the cells show elongated, slightly tapering nuclei with pale eosinophilic cytoplasm arranged haphazardly and in swirling bundles – classic for nodular fasciitis.  3 Myxoid to collagenous stroma The background alternates between loose, pale basophilic/myxoid matrix and more fibrous areas, another hallmark of this lesion.  4 **Numerous thin-walled, slit-like vessels with prominent extravasated erythrocytes Small capillaries and scattered pools of free red cells are easily seen, a common clue to nodular fasciitis and much less typical of fibroma or scar.  5 **Scattered lymphocytes / occasional neutrophils, brisk but non-atypical mitoses Light chronic-active inflammation and mitotic figures (if searched for) fit the rapid growth phase of nodular fasciitis yet there is no cytologic atypia or abnormal mitotic forms.  6 Overlying epithelium without dysplasia or invasive nests The surface squamous epithelium may be slightly hyperplastic or ulcerated but shows no carcinoma in situ, and no epithelial islands extend into the nodule – helping exclude squamous cell carcinoma, even its spindle-cell variant.  Taken together – a rapidly-growing but benign, submucosal, plump spindle-cell proliferation with myxoid change, extravasated RBCs, and no epithelial malignancy – these features are diagnostic of nodular fasciitis in the oral cavity. |
| 11.64 | Diagnosis: Sarcomatoid carcinoma of squamous cells; Key Microscopic Features: Spindle and epithelioid tumor cell proliferation, ulceration. Spindle cells exhibit nuclear atypia and frequent mitotic figures. Epithelioid tumor cells are polygonal with large nuclei and prominent nucleoli. The lesion shows osteoid deposition. | Histopathological Diagnosis:  Squamous Cell Carcinoma (SCC) of the oral cavity.  Key Microscopic Features Justifying the Diagnosis:  1. Architectural Disturbance (First Image - Low Power):  Disruption of normal epithelial maturation.  Invasion of atypical epithelial cells through the basement membrane into the underlying connective tissue, consistent with invasive carcinoma.  The overlying epithelium shows loss of orderly stratification, with keratin pearl formation suggestive of well-differentiated SCC.  2. Cytologic Atypia (Second Image - Intermediate Power):  Presence of pleomorphic spindle-shaped cells with hyperchromatic, enlarged, irregular nuclei.  Increased nuclear-to-cytoplasmic ratio.  Prominent nucleoli.  Frequent mitotic figures, some atypical.  3. Cellular Disorganization and High-Grade Features (Third Image - High Power):  Marked pleomorphism and nuclear hyperchromasia.  Numerous atypical mitoses and individual cell keratinization.  Tumor cells are arranged haphazardly with a loss of polarity and cohesion—indicative of poorly regulated epithelial growth.  4. Stromal Invasion and Desmoplasia (Fourth Image - High Power):  Infiltrative nests and cords of malignant squamous cells within a desmoplastic stroma.  Keratin pearls and intercellular bridges are visible, further confirming squamous differentiation.  Surrounding stroma shows inflammatory response and fibrosis, commonly seen in invasive SCC.  Conclusion:  The constellation of features—epithelial dysplasia, stromal invasion, keratin pearls, cytologic atypia, and mitotic activity—confirms a diagnosis of Squamous Cell Carcinoma, the most common malignant lesion in the group of leukoplakia, erythroplakia, oral dysplasia, and SCC. | Histopathological diagnosis (single best):  High-grade (conventional) osteosarcoma of the jaw – predominantly osteoblastic, with focal fibro- and chondroblastic areas.  Key microscopic features in the photomicrographs that support this diagnosis  Field Observations Diagnostic significance  Low-power overview (Image 1) • Intact oral mucosal epithelium on the left; underlying lamina propria and deeper tissues replaced by an expansile, highly cellular lesion.  • Infiltrative tongues of tumour permeating pre-existing trabecular bone (seen as empty clefts where bone has been resorbed). Shows an aggressive mesenchymal lesion arising in bone and invading toward the surface mucosa – typical for gnathic osteosarcoma.  Spindle-cell component (Image 2) • Interlacing fascicles of malignant spindle cells with elongated (“cigar-shaped”) hyperchromatic nuclei and occasional mitoses.  • Collagenous stroma between bundles. Represents the fibroblastic element frequently seen in conventional osteosarcoma.  Pleomorphic/epithelioid focus (Image 3) • Sheets of markedly atypical, pleomorphic polygonal cells with enlarged vesicular nuclei, prominent nucleoli, and brisk mitotic activity (including atypical forms).  • Scattered multinucleate tumour giant cells. Confirms high-grade sarcomatous nature of the lesion.  Osteoid production (Image 4) • Broad, lace-like eosinophilic, unmineralised osteoid permeating through malignant cells.  • Tumour cells are seen directly depositing osteoid (no intervening normal osteoblasts).  • Foci of mineralising bone and occasional chondroid matrix at the periphery. Pathognomonic feature of osteosarcoma – malignant mesenchymal cells forming osteoid or bone. Presence of abundant lace-like osteoid classifies the tumour as the osteoblastic subtype.  Why the appearances favour osteosarcoma over other spindle/pleomorphic lesions  Direct tumour osteoid distinguishes osteosarcoma from fibrosarcoma, malignant peripheral nerve-sheath tumour, spindle-cell carcinoma, or undifferentiated pleomorphic sarcoma.  Combination of spindle, pleomorphic, and chondroblastic foci within one lesion is typical of conventional “mixed-pattern” osteosarcoma of the jaws.  The lesion is centred in bone and secondarily approaches mucosa, consistent with gnathic osteosarcoma rather than a primary epithelial malignancy.  Conclusion: The constellation of an infiltrative, highly cellular sarcoma with malignant spindle and pleomorphic cells that actively lay down woven osteoid is diagnostic of a high-grade conventional (osteoblastic-predominant) osteosarcoma. |
| 11.66 | Diagnosis: Human papillomavirus–associated basaloid squamous cell carcinoma of the oral cavity; Key Microscopic Features: Lobules of tumor cells with comedo necrosis; carcinoma-in-situ is noted at the left margin. Hyperchromatic cells surround necrotic areas. Apoptotic cells are present, along with numerous mitotic figures. | Histopathological Analysis of Submitted H&E Images  Based on the three hematoxylin-and-eosin (H&E) stained histological images captured at progressively higher magnifications, the most likely single histopathological diagnosis is:  Invasive Squamous Cell Carcinoma (SCC) of the Oral Cavity  Key Microscopic Features Justifying the Diagnosis:  1. Invasive Growth Pattern (Image 1 - Low Magnification):  There is an irregular, infiltrative proliferation of epithelial nests and cords into the underlying connective tissue stroma.  The normal epithelial-connective tissue interface is disrupted, supporting invasion.  Keratin pearl formation is evident within these islands — a hallmark of well to moderately differentiated SCC.  2. Cytological Atypia (Image 2 - Intermediate Magnification):  Enlarged, hyperchromatic nuclei with irregular nuclear contours.  Increased nuclear-to-cytoplasmic ratio.  Abundant mitotic figures, including atypical mitoses.  Intercellular bridges are visible, indicating squamous differentiation.  Evidence of keratinization within tumor nests, consistent with squamous lineage.  3. High-Grade Dysplasia & Tumor Cell Morphology (Image 3 - High Magnification):  Pronounced pleomorphism: nuclei vary in size and shape.  Dyskeratosis (abnormal keratinization within individual cells).  Prominent nucleoli and scattered apoptotic bodies.  Tumor cells are breaking through the basement membrane, infiltrating the stroma — key in distinguishing carcinoma in situ from invasive carcinoma.  Presence of tumor-associated inflammatory infiltrate and stromal reaction.  Differential Considerations Ruled Out:  Oral epithelial dysplasia: Would show architectural disturbance and cytologic atypia limited to the epithelium without invasion through the basement membrane.  Leukoplakia or erythroplakia: These are clinical descriptors and may harbor dysplasia or carcinoma but are not histologic diagnoses.  Carcinoma in situ: Lacks stromal invasion, which is evident in these images.  Conclusion:  The images demonstrate classical features of invasive squamous cell carcinoma, including epithelial nests invading the connective tissue, keratin pearl formation, and marked cellular atypia with mitotic activity. These features support a confident diagnosis of oral squamous cell carcinoma. | Histopathological diagnosis (single most likely):  Conventional keratinising invasive squamous cell carcinoma of the oral cavity (moderately differentiated).  Key microscopic features that support this diagnosis  Field Feature seen in the photomicrographs Diagnostic significance  Low-power architecture • Irregular, tongue-like nests and broad islands of epithelium burrowing through the lamina propria and deeper connective tissue.  • Loss of the normal mucosa–connective‐tissue interface. Invasion beyond the basement membrane is the sine-qua-non for carcinoma, separating it from dysplasia or carcinoma in situ.  Tumour stroma • Desmoplastic, fibro-collagenous reaction surrounding tumour nests.  • Scattered inflammatory infiltrate. Desmoplasia is typical of an invasive SCC and emphasises stromal breach.  Keratinisation • Eosinophilic whorls and central “keratin pearls” within nests.  • Individual-cell keratinisation. Indicates a keratinising (conventional) phenotype and^—because the pearls are reasonably formed—places the tumour in the well-to-moderately differentiated spectrum.  Cytologic atypia • Polygonal cells with enlarged, pleomorphic, hyperchromatic nuclei.  • Prominent nucleoli, coarse chromatin.  • Increased and atypical mitotic figures. Marks malignant transformation of squamous epithelium.  Intercellular bridges Delicate eosinophilic lines between adjacent tumour cells in higher-power views. Confirms squamous (as opposed to basaloid or glandular) differentiation.  Additional clues • Foci of tumour necrosis and vascular congestion.  • Possible vascular/perineural channels adjacent to nests (route for spread). Common accompanying changes that reinforce the malignant nature of the lesion.  The combination of invasive growth pattern, squamous differentiation with keratin pearl formation, and marked cytologic atypia firmly establishes the diagnosis of invasive (moderately differentiated) keratinising squamous cell carcinoma. |
| 11.67 | Diagnosis: Human papillomavirus–associated basaloid squamous cell carcinoma of the oral cavity; Key Microscopic Features: Lobules of basaloid tumor cells. Areas of comedo necrosis observed. Tumor cells are fairly uniform with many apoptotic cells. | Based on the provided hematoxylin and eosin (H&E) stained histopathological images at low, intermediate, and high magnification, the most likely diagnosis is:  Squamous Cell Carcinoma (SCC)  Key Microscopic Features Supporting This Diagnosis:  1. Low Magnification (First Image)  Infiltrative growth pattern: The lesion lacks a clear boundary with adjacent tissues, with lobules and irregular nests of epithelial cells extending into deeper connective tissue.  Architectural disarray: There is loss of normal epithelial architecture and stratification, with irregular masses and cords of epithelial cells.  2. Intermediate Magnification (Second Image)  Keratin pearl formation: The central eosinophilic, circular collections surrounded by malignant epithelial cells are classic for well-differentiated SCC.  Nuclear pleomorphism: Nuclei appear enlarged and irregular in shape, indicating significant atypia.  Increased mitotic activity: Mitotic figures are observed, including abnormal mitoses.  3. High Magnification (Third Image)  Severe cytologic atypia: Marked pleomorphism, hyperchromatic nuclei, and prominent nucleoli.  High nucleus-to-cytoplasm ratio: Consistent with malignant transformation.  Abnormal mitoses: Mitoses are frequent and atypical, further confirming malignancy.  Conclusion:  The histopathological features strongly indicate invasive squamous cell carcinoma, a malignant epithelial neoplasm common in the oral cavity. The presence of keratin pearls, cytologic atypia, infiltrative growth, and high mitotic index all support this diagnosis. | Histopathological diagnosis  Invasive moderately differentiated squamous-cell carcinoma (oral cavity)  Key microscopic features that support the diagnosis  Magnification What is seen Why it points to squamous-cell carcinoma  Low power (whole-slide view) • A well-demarcated but infiltrative mass composed of numerous lobules/islands of epithelium separated by fibrous septa.  • No normal surface epithelium is interposed between the tumour and the connective tissue, confirming invasion. The lobular arrangement and penetration beyond the epithelial–connective-tissue junction establish that this is an invasive epithelial (carcinomatous) process rather than an intra-epithelial dysplasia.  Intermediate power • Nests of polygonal cells with obvious intercellular bridges.  • Central concentric eosinophilic material in many islands – classic keratin pearls / dyskeratosis.  • Foci of tumour necrosis at the centre of some nests.  • Desmoplastic stromal response around the islands. Keratin pearl formation and intercellular bridges are hallmark signs of squamous differentiation, placing the tumour in the keratinising (moderately differentiated) spectrum of SCC.  High power • Marked cytologic atypia – enlarged pleomorphic nuclei, coarse chromatin, irregular nuclear membranes, occasional prominent nucleoli.  • Increased nuclear-to-cytoplasmic ratio and numerous atypical mitotic figures.  • Individual-cell keratinisation and cytoplasmic eosinophilia in some cells. The degree of atypia, frequent mitoses and individual-cell keratinisation confirm malignancy and a grade beyond well-differentiated SCC, but the presence of overt keratinisation keeps it in the “moderately differentiated” category.  Collectively, these features (invasive growth, keratin pearls, intercellular bridges, cellular pleomorphism and atypical mitoses) are diagnostic of an invasive moderately differentiated squamous-cell carcinoma of the oral mucosa rather than leukoplakia, erythroplakia, or a purely dysplastic lesion. |
| 11.69 | Diagnosis: Human papillomavirus–associated carcinoma of the tonsil; Key Microscopic Features: Trabeculae and ribbons of squamous cells within the tonsil. The tumor contains pale cells and shows comedo necrosis. Cells exhibit abundant pale cytoplasm, ovoid nuclei with small nucleoli, mitotic figures, and early necrosis. | Based on the histopathological analysis of the provided hematoxylin-and-eosin (H&E) stained images at low, intermediate, and high magnification, the single most likely histopathological diagnosis is:  Squamous Cell Carcinoma (SCC)  Key Microscopic Features Justifying the Diagnosis:  Low Magnification (Image 1):  Infiltrative architecture: Irregular nests and islands of epithelial cells invading the underlying stroma.  Loss of normal epithelial maturation and architecture, indicating disruption of tissue organization.  Desmoplastic stromal reaction, suggestive of invasion.  Intermediate Magnification (Image 2):  Keratin pearl formation: Central keratinization within epithelial nests, a hallmark of well-differentiated squamous cell carcinoma.  Irregular, hyperchromatic nuclei and pleomorphism.  Dense lymphocytic infiltrate around tumor islands, which is commonly seen in invasive SCC.  High Magnification (Image 3):  Nuclear pleomorphism: Markedly variable nuclear size and shape.  Increased nuclear-to-cytoplasmic ratio and prominent nucleoli.  Abnormal mitotic figures may be noted.  Individual cell keratinization and intercellular bridges, which are typical of squamous differentiation.  Conclusion:  The combination of invasive epithelial nests, keratin pearl formation, nuclear atypia, and the presence of desmoplastic stroma and inflammatory response strongly supports the diagnosis of Squamous Cell Carcinoma, most likely of the oral cavity. | Histopathological diagnosis (single best answer)  Invasive, moderately differentiated keratinising squamous cell carcinoma of the oral cavity.  Key microscopic features that support this diagnosis  Field of view Salient findings Diagnostic significance  Low-power overview (first image) • Numerous irregular nests, lobules and cords of epithelium have breached the basement membrane and infiltrate deeply into the connective-tissue stroma.  • Tumour islands are separated by fibrous septa and are surrounded by a dense, basophilic lymphoplasmacytic infiltrate. Confirms invasion (malignancy) and the typical lobulated growth pattern of SCC.  Intermediate magnification (second image) • Within many islands the centre is pale, eosinophilic and whorled, forming keratin pearls.  • Peripheral tumour cells are more basaloid; central cells show maturation toward squamous phenotype.  • Prominent stromal inflammatory response. Keratin-pearl formation and progressive maturation from periphery to centre are hallmarks of a moderately differentiated keratinising SCC variant.  High power (third image) • Tumour cells exhibit marked nuclear pleomorphism, hyperchromasia, coarse chromatin and conspicuous nucleoli.  • High nuclear-to-cytoplasmic ratio with frequent atypical and brisk mitotic figures.  • Foci of individual-cell keratinisation and eosinophilic cytoplasm; occasional intercellular bridges can be appreciated. Cytologic atypia, atypical mitoses and dyskeratosis confirm malignancy and squamous lineage.  Additional supportive features  Desmoplastic stromal reaction around invading islands.  Vascular congestion and focal haemorrhage within the tumour stroma.  Lack of glandular, mucous or clear-cell differentiation, arguing against other salivary or odontogenic neoplasms.  Taken together, the pattern of invasive squamous nests with keratin pearls, cytologic atypia and stromal response is diagnostic of a moderately differentiated keratinising squamous cell carcinoma. |
| 11.70 | Diagnosis: Human papillomavirus (HPV)–associated papillary squamous cell carcinoma; Key Microscopic Features: Non-keratinizing tumor in a papillary configuration. Tumor cells form interlacing trabeculae. Cells have pale eosinophilic cytoplasm, ovoid nuclei with small nucleoli, and numerous mitotic figures. | Based on the hematoxylin and eosin (H&E) stained histological images provided at different magnifications, the single most likely histopathological diagnosis is:  Squamous Cell Carcinoma (SCC) of the oral cavity.  🔬 Key Microscopic Features Supporting the Diagnosis:  1. Low-power view (1st image):  Exophytic and endophytic growth patterns: The lesion exhibits a large, irregular, infiltrative architecture, with papillary or fungating projections.  Loss of normal mucosal architecture: There is disruption of the orderly stratification of squamous epithelium, suggestive of an invasive process.  2. Intermediate-power view (2nd image):  Nests and islands of malignant squamous epithelial cells infiltrating the underlying stroma.  Keratin pearl formation: Evidence of squamous differentiation.  Desmoplastic stromal reaction (fibrosis around tumor nests), which is a hallmark of invasive carcinoma.  3. High-power view (3rd image):  Marked cytologic atypia: Enlarged, hyperchromatic, and pleomorphic nuclei.  Increased mitotic activity, some possibly atypical.  Loss of normal polarity and disordered cell arrangement.  Individual cell keratinization – another indicator of squamous differentiation.  🔎 Differential Diagnosis Consideration:  Although lesions such as oral epithelial dysplasia (OED) or carcinoma in situ (CIS) might be considered in differential, the key features of stromal invasion and keratin pearl formation confirm that this is invasive squamous cell carcinoma rather than a premalignant lesion. | Single most likely histopathological diagnosis  Invasive squamous cell carcinoma (papillary / exophytic-type, moderately-to-poorly differentiated).  Key microscopic features supporting this diagnosis  Field of view Observations Diagnostic significance  Low-power overview (first image) • Broad, cauliflower-like, papillary fronds rising above the mucosal surface  • Irregular, downward-growing fronts that break through the epithelial–connective-tissue junction Exophytic papillary architecture plus breach of the basement membrane indicates an invasive malignancy rather than a benign papilloma or dysplasia limited to the epithelium.  Intermediate magnification (second image) • Nests and cords of epithelium infiltrating the lamina propria  • Loss of orderly maturation from basal to superficial layers  • Focal keratin pearl / dyskeratotic whorl-like material inside tumour islands Invasion is the sine-qua-non for carcinoma. Keratin pearls confirm squamous differentiation.  High magnification (third image) • Marked nuclear pleomorphism and hyperchromasia  • Increased nuclear-to-cytoplasmic ratio  • Numerous (and some atypical) mitotic figures, including in suprabasal layers  • Occasional individual-cell keratinisation These cytologic features demonstrate high-grade epithelial dysplasia/ malignancy, distinguishing SCC from verrucous carcinoma (which shows minimal atypia) and from severe dysplasia/ in-situ disease (which lacks stromal invasion).  Ancillary findings often present (though not the primary diagnostic criteria in these frames)  Desmoplastic stromal response and patchy chronic inflammation surrounding invading islands  Surface parakeratin plugging within papillary tips  Possible tumour necrosis in deeper nests in more poorly differentiated foci  Taken together, the invasive growth pattern, papillary exophytic configuration, and pronounced squamous atypia with keratin production establish the lesion as papillary / exophytic invasive squamous cell carcinoma of the oral cavity rather than leukoplakia with dysplasia, erythroplakia, or an in-situ process. |
| 11.71 | Diagnosis: Minimally keratinizing adenoid (acantholytic) squamous cell carcinoma; Key Microscopic Features: Infiltrating squamous cell carcinoma. Pseudoglandular spaces contain acantholytic, dyskeratotic, and pleomorphic tumor cells. | Based on the provided hematoxylin-and-eosin stained histopathology images of the same lesion at low and high magnification, here is the analysis:  Single Most Likely Histopathological Diagnosis:  Oral Squamous Cell Carcinoma (OSCC)  Key Microscopic Features Justifying the Diagnosis:  Low Magnification (First Image):  Infiltrative growth pattern: Irregular nests and islands of epithelial cells infiltrating the underlying connective tissue stroma, breaching the basement membrane.  Architectural disorganization: Loss of normal epithelial stratification and organization, with downgrowth of abnormal epithelium.  Desmoplastic stromal reaction: The surrounding stroma shows a fibrotic/desmoplastic response, typical of invasive carcinoma.  Keratin pearls: Suggestive concentric eosinophilic whorls within tumor nests indicating squamous differentiation.  High Magnification (Second Image):  Malignant epithelial cells: Cells display pleomorphism (variation in size and shape), hyperchromatic nuclei, prominent nucleoli, and high nuclear-to-cytoplasmic ratios.  Intercellular bridges: Focal evidence of squamous differentiation.  Keratinization: Presence of individual cell keratinization and keratin pearls within tumor nests.  Mitotic figures: Increased and atypical mitoses, indicative of high proliferative activity.  Inflammatory infiltrate: Mixed inflammatory response in the stroma, common in invasive carcinoma.  Conclusion:  The presence of infiltrative epithelial nests with marked cytologic atypia, keratin pearl formation, and disruption of normal architecture confirms the diagnosis of oral squamous cell carcinoma (OSCC). This distinguishes it from pre-malignant lesions like leukoplakia or erythroplakia with dysplasia, which typically lack definitive stromal invasion. | Single most likely histopathological diagnosis  Invasive, well-differentiated (keratinising) squamous cell carcinoma of the oral mucosa  Key microscopic features that support this diagnosis  Low-power survey (first image) High-power detail (second image) Diagnostic significance  1. Irregular nests, cords and tongues of epithelium penetrating deeply through the lamina propria beyond the level of the basement membrane. 1. Cohesive tumour islands surrounded by a desmoplastic fibrous stroma with brisk chronic-inflammatory cell infiltrate. Confirms invasion, separating carcinoma from in-situ dysplasia or reactive epithelial change.  2. Surface mucosa is ulcerated / eroded with a sharp step-off to the infiltrating tumour front. 2. Keratin pearl formation – concentric laminations of eosinophilic keratin in the centres of many tumour nests. Keratin pearls are the hallmark of a well-differentiated keratinising SCC.  3. Marked architectural disturbance: loss of orderly epithelial maturation, bulbous rete ridges, drop-off of tumour buds into the stroma. 3. Individual-cell keratinisation and dyskeratosis within tumour nests; cells show abundant dense eosinophilic cytoplasm and readily identifiable intercellular bridges. Confirms squamous lineage and good degree of differentiation.  4. Cytological atypia – enlarged pleomorphic, hyperchromatic nuclei, prominent nucleoli and frequent mitotic figures (including atypical forms). Confirms malignancy.  5. Stromal host response – lymphoplasmacytic infiltrate and focal desmoplasia around tumour islands. A common accompaniment of invasive oral SCC.  Taken together, the invasive growth pattern, keratin-pearl and individual-cell keratin formation, intercellular bridges, and cytological atypia firmly establish the lesion as a well-differentiated (keratinising) squamous cell carcinoma. |
| 11.72 | Diagnosis: Keratinizing adenoid (acantholytic) squamous cell carcinoma; Key Microscopic Features: Keratin-forming squamous cell carcinoma with pseudoglandular structures at the base of the lesion. Acantholytic tumor cells float within spaces in tumor islands. Pleomorphic tumor cells are also observed. | Histopathological Diagnosis:  Squamous Cell Carcinoma (SCC) of the oral cavity.  Justifying Microscopic Features Seen Across the Images:  1. Low-power view (1st image):  Invasive growth pattern: There is clear disruption of the normal epithelial architecture with irregular epithelial nests and cords invading the underlying connective tissue.  Loss of epithelial stratification and basement membrane integrity, supporting invasion.  Keratin pearl formation (eosinophilic, concentric lamellated structures) indicating squamous differentiation.  Desmoplastic stromal reaction and an inflammatory infiltrate in the stroma—common in SCC.  2. Intermediate magnification (2nd image):  Prominent keratin pearl formation is more obvious.  Individual cell keratinization and intercellular bridges are seen in tumor nests, hallmark features of well-differentiated squamous cell carcinoma.  Nuclear atypia: Cells show enlarged, hyperchromatic nuclei with pleomorphism and irregular contours.  Mitotic figures, some atypical, are present.  3. High-power view (3rd image):  Marked nuclear pleomorphism and hyperchromasia.  Abnormal mitotic figures and loss of normal cellular polarity.  Tumor cells exhibit dyskeratosis (premature keratinization within individual cells), which is typical in SCC.  Dense inflammatory infiltrate at the tumor-stroma interface.  Conclusion:  The constellation of features—keratin pearl formation, invasive squamous epithelial nests, nuclear pleomorphism, mitoses, and stromal invasion—firmly supports the diagnosis of well-differentiated squamous cell carcinoma of the oral cavity. | Histopathological diagnosis  Invasive keratinising squamous cell carcinoma (moderately differentiated) of the oral mucosa.  Key microscopic features that support the diagnosis  Field Findings Diagnostic significance  Low power (image 1) • Irregular exophytic surface with broad, downward-growing tongues and nests of epithelium breaching the lamina propria  • Tumour islands infiltrate deeply into the underlying connective tissue with desmoplastic stroma Confirms stromal invasion—the sine-qua-non for carcinoma rather than epithelial dysplasia or carcinoma in situ  Intermediate power (image 2) • Conspicuous keratin pearls (concentric eosinophilic whorls) inside tumour nests  • Intercellular bridges between malignant squamous cells  • Mixed chronic-active inflammatory infiltrate at the invasion front Keratin production and bridges signify squamous differentiation; organised keratin pearls indicate at least a moderate degree of differentiation  High power (image 3) • Marked cytologic atypia: pleomorphic, hyperchromatic nuclei with irregular contours, coarse chromatin and prominent nucleoli  • Increased nuclear-to-cytoplasmic ratio and loss of normal polarity  • Numerous mitotic figures, including atypical forms  • Foci of individual cell keratinisation (dyskeratosis) Cytologic malignant features confirm carcinoma and help grade it as moderately differentiated (keratinising but with obvious atypia and brisk mitoses)  Taken together, the presence of invasive epithelial nests with keratin-pearl formation, intercellular bridges, pronounced cytologic atypia and mitotic activity clearly establish the lesion as an invasive keratinising squamous cell carcinoma rather than a premalignant dysplasia, leukoplakia, erythroplakia, or carcinoma in situ. |
| 11.73 | Diagnosis: Adenoid (acantholytic) squamous cell carcinoma; Key Microscopic Features: The tumor is pemphigus-like, forming suprabasilar clefts and papillary structures. Tumor cells have pleomorphic, hyperchromatic nuclei. Cells within tissue clefts exhibit pleomorphism and dyskeratosis. | Histopathological Analysis  Single Most Likely Diagnosis:  Well-differentiated Squamous Cell Carcinoma (SCC) of the oral cavity.  Key Microscopic Features Supporting This Diagnosis:  Invasive Epithelial Nests and Islands:  The images show irregular nests and islands of squamous epithelial cells invading the underlying connective tissue. This is a hallmark of invasive squamous cell carcinoma.  The cells break through the basement membrane, which is not seen in dysplasia or carcinoma in situ.  Keratin Pearl Formation:  Concentric layers of keratinized cells (keratin pearls) are visible within the epithelial islands, a characteristic feature of well-differentiated SCC.  These pearls reflect an attempt by the malignant squamous cells to produce keratin, indicating a degree of differentiation.  Cellular and Nuclear Pleomorphism:  Marked variability in nuclear size and shape is evident.  Hyperchromatic (dark-staining) nuclei and prominent nucleoli are observed.  Atypical Mitoses:  Increased mitotic activity, some of which appear abnormal, supports a malignant process.  Desmoplastic Stromal Response:  The surrounding stroma shows desmoplasia (reactive fibrous tissue formation), often seen in invasive carcinomas.  Associated inflammatory infiltrate can also be observed.  Loss of Normal Stratification:  The normal layered architecture of squamous epithelium is disrupted, with disorganized epithelial proliferation.  Conclusion:  The combination of keratin pearl formation, stromal invasion by atypical squamous cells, and significant cytological atypia confirms the diagnosis of well-differentiated squamous cell carcinoma rather than pre-malignant lesions like dysplasia or carcinoma in situ. | Histopathological diagnosis  Invasive, moderately differentiated (keratinising) oral squamous cell carcinoma (SCC).  Key microscopic features that support this diagnosis  Category Features visible in the submitted sections  Evidence of invasion • Irregular cords and rounded islands of atypical squamous epithelium are present beneath the epithelial-connective-tissue junction, separated from the surface epithelium by desmoplastic stroma.  • Stromal reaction with fibrous tissue and mixed inflammatory infiltrate surrounds the nests.  Cytologic atypia • Marked nuclear pleomorphism and hyperchromasia.  • Enlarged, irregular, vesicular nuclei with conspicuous nucleoli.  • Increased nuclear-to-cytoplasmic ratio and numerous mitotic figures, including atypical forms.  Squamous differentiation • Foci of individual-cell keratinisation and early keratin-pearl formation within tumour islands.  • Intercellular bridges can be appreciated in some nests, confirming squamous lineage.  Architectural disturbance of the surface epithelium • Loss of normal maturation, bulbous and anastomosing rete ridges, and drop-shaped extensions.  • Full-thickness dysplasia of the overlying mucosa, merging with invasive fronts.  Tumour–host interface • Jagged, infiltrative borders rather than a pushing/verruciform front.  • Accompanying chronic inflammatory infiltrate and focal necrosis in the stroma.  The combination of full-thickness epithelial dysplasia with frank breach of the basement membrane and invasion of atypical, keratin-producing squamous nests into the underlying connective tissue unequivocally establishes the lesion as an invasive, moderately differentiated squamous cell carcinoma of the oral mucosa. |
| 11.74 | Diagnosis: Adenosquamous cell carcinoma; Key Microscopic Features: Keratin-forming squamous cell carcinoma that forms ductal structures. Tumor cells form ducts, and focal keratinization is present. | Histopathological Diagnosis:  Invasive Squamous Cell Carcinoma (SCC) of the Oral Cavity  Key Microscopic Features Supporting the Diagnosis:  From the Low-Magnification Image:  Architectural Disorganization:  Loss of normal epithelial maturation and polarity.  Infiltrative growth pattern breaching the basement membrane.  Irregular Nests and Islands of Cells:  Tumor cells forming nests, cords, and islands that extend into the underlying connective tissue, a hallmark of invasion.  Keratin Pearls:  Concentric, eosinophilic structures within the tumor nests indicate squamous differentiation and are characteristic of well-differentiated SCC.  From the High-Magnification Image:  Cellular Atypia:  Marked nuclear pleomorphism and hyperchromasia.  Prominent nucleoli and high nuclear-to-cytoplasmic ratio.  Increased Mitotic Activity:  Frequent and atypical mitotic figures, which are features of malignancy.  Desmoplastic Stromal Reaction:  Dense fibrous stroma surrounding invasive tumor nests, indicating host tissue response to invasion.  Dyskeratosis:  Premature keratinization of individual tumor cells, a sign of abnormal squamous differentiation.  Summary:  The presence of infiltrative nests of atypical squamous cells with keratin pearl formation, cellular pleomorphism, increased mitoses, and disruption of the epithelial-connective tissue interface strongly supports a diagnosis of invasive squamous cell carcinoma. This lesion clearly shows progression beyond dysplasia into frank carcinoma. | Histopathological diagnosis  Invasive (conventional) squamous-cell carcinoma of the oral mucosa – moderately to well differentiated.  Key microscopic features visible in the provided H-E sections  Area of the slide Morphologic finding Diagnostic relevance  Surface epithelium Irregular downward proliferation and loss of the normal, orderly maturation pattern; full-thickness cytologic atypia with enlarged, hyperchromatic, pleomorphic nuclei and frequent mitoses Confirms severe dysplasia / carcinoma in situ in the surface epithelium, establishing that the process is malignant in origin  Interface with lamina propria Basement-membrane breach with tongues, nests and cords of atypical squamous cells infiltrating the underlying connective tissue Defines invasion, the sine-qua-non for a diagnosis of invasive carcinoma rather than intra-epithelial dysplasia alone  Tumour nests within stroma (low power) Numerous discrete and anastomosing nests that are haphazardly distributed through a desmoplastic stroma Typical architectural pattern of invasive squamous-cell carcinoma  Tumour cells (high power) • Abundant eosinophilic (keratinising) cytoplasm  • Marked nuclear pleomorphism and hyperchromasia  • Prominent nucleoli  • Atypical and occasional abnormal mitotic figures Confirms malignant squamous phenotype and cellular-grade atypia  Keratinisation Concentric “keratin pearls” as well as individual-cell dyskeratosis within tumour islands Indicates at least moderate differentiation; presence of keratin pearls is characteristic of conventional SCC  Stromal response Fibro-desmoplastic reaction with scattered chronic inflammatory cells and focal haemorrhage Common host response to invading SCC, supports chronicity and invasion  Taken together, the combination of full-thickness epithelial atypia plus unequivocal invasion of keratinising, cytologically malignant squamous cells into the supporting connective tissue establishes the diagnosis of invasive squamous-cell carcinoma. The evident keratin-pearl formation places it in the moderately-to-well differentiated spectrum. |
| 11.75 | Diagnosis: Adenosquamous carcinoma; Key Microscopic Features: Keratin-forming squamous cell carcinoma with ductal structures. Conventional squamous cell carcinoma is present along with ductal structures filled with mucinous material. | Histopathological Analysis of H&E-Stained Images  Single Most Likely Histopathological Diagnosis:  Well-differentiated squamous cell carcinoma (SCC) of the oral cavity.  Key Microscopic Features Supporting the Diagnosis:  1. Invasive Growth Pattern  There is clear evidence of infiltrative epithelial nests and cords invading into the underlying connective tissue and muscle bundles.  These epithelial islands breach the basement membrane, a hallmark of invasive carcinoma.  2. Keratin Pearls  Prominent keratin pearls are visible within the invading epithelial nests, especially evident in the higher magnification images. These are concentric layers of keratinized cells and a classic feature of well-differentiated SCC.  3. Individual Cell Keratinization  Eosinophilic cytoplasm and hyperchromatic nuclei with dyskeratosis (premature keratinization of individual cells) are identifiable.  Cells with increased nuclear-to-cytoplasmic ratios, pleomorphism, and atypical mitoses further support malignancy.  4. Desmoplastic Stromal Reaction  Dense fibrous stroma surrounding the tumor islands, representing a desmoplastic response, is evident, particularly in the lower magnification image.  5. Inflammatory Infiltrate  A lymphoplasmacytic inflammatory infiltrate is present in the stroma, which is commonly associated with invasive carcinoma.  Conclusion:  The images collectively demonstrate an infiltrative squamous neoplasm with keratin pearl formation and desmoplasia, confirming the diagnosis of well-differentiated squamous cell carcinoma of the oral mucosa. | Histopathological diagnosis (single best):  Well-differentiated (keratinising) invasive squamous cell carcinoma of the oral mucosa  Key microscopic features that lead to this diagnosis  Low-, medium- and high-power findings Diagnostic significance  1. Irregular nests, cords and tongues of epithelium infiltrating deeply into the fibrous stroma and skeletal muscle (Image 1, panoramic view) Confirms breach of the basement membrane and an invasive rather than intra-epithelial process  2. Prominent keratin pearls – concentrically laminated eosinophilic keratin in the centre of tumour islands (Images 2 & 3) Hallmark of a well-differentiated keratinising SCC  3. Individual-cell keratinisation and dyskeratosis within the tumour nests (Image 3) Supports squamous, not glandular, differentiation  4. Marked cytologic atypia: enlarged pleomorphic hyperchromatic nuclei, irregular nuclear membranes, coarse chromatin, conspicuous nucleoli, and numerous (sometimes atypical) mitoses (best appreciated in Image 3) Establishes malignancy  5. Intercellular bridges between tumour cells where cytoplasm is preserved (Image 3) Characteristic of squamous lineage  6. Desmoplastic stromal reaction with dense collagen and strands of tumour wrapped by spindle-cell fibroblasts (all images) Typical host response to invading SCC  7. Peritumoral chronic inflammatory infiltrate of lymphocytes and plasma cells (Images 2 & 3) Common accompaniment of oral SCC  8. Focal surface epithelial ulceration/dysplasia (upper border of Image 2) Often seen in carcinomas arising from leukoplakic/erythroplakic mucosa  These combined architectural and cytological features are classic for a keratinising, well-differentiated squamous cell carcinoma, distinguishing it from non-invasive epithelial dysplasia or other salivary and odontogenic tumours in the same differential group. |
| 11.76 | Diagnosis: Carcinoma cuniculatum; Key Microscopic Features: The tumor presents deeply branching, irregular cystic spaces filled with keratin, originating from a smooth surface with invaginations. The cyst lining is surrounded by dysplastic epithelium, and pleomorphic tumor cells infiltrate the surrounding stroma. | Histopathological Analysis  Based on the three provided Hematoxylin and Eosin (H&E) stained images at low, intermediate, and high magnification, the single most likely diagnosis is:  Well-differentiated Squamous Cell Carcinoma (SCC)  Justification and Key Microscopic Features  1. Low Magnification (First Image):  Invasive growth pattern: Irregular epithelial proliferations extend into the underlying connective tissue.  Keratinizing tumor nests: Pink, eosinophilic, keratinized areas are seen centrally within epithelial islands, which is a hallmark of well-differentiated SCC.  Loss of normal epithelial architecture: The usual stratified squamous organization is disrupted.  2. Intermediate Magnification (Second Image):  Keratin pearls: Well-formed, concentrically layered keratin within tumor nests, characteristic of well-differentiated SCC.  Desmoplastic stroma: Fibrotic stromal reaction surrounding the invading nests, indicative of stromal invasion.  3. High Magnification (Third Image):  Cellular atypia: Enlarged, pleomorphic, and hyperchromatic nuclei.  Dyskeratosis: Abnormal keratinization within individual tumor cells.  Mitotic figures: Indicating active proliferation.  Intercellular bridges: Retained in some areas, supporting squamous differentiation.  Summary  All features strongly support the diagnosis of well-differentiated squamous cell carcinoma, a malignant epithelial tumor that arises from dysplastic squamous epithelium and shows clear signs of keratinization and invasion. | Histopathological diagnosis  → Conventional (keratinising) well-differentiated squamous cell carcinoma of the oral mucosa  Key microscopic features supporting the diagnosis  Magnification level Observations Diagnostic significance  Low-power overview (Image 1) • Thickened, papillary surface epithelium with deep, irregular downward extensions that breach the epithelial–connective-tissue interface  • Numerous tongues, nests and islands of epithelium infiltrating the lamina propria and deeper connective tissue  • Foci of central eosinophilic material within the nests - Loss of normal epithelial architecture and invasion beyond the basement membrane is the sine-qua-non of carcinoma  - Exophytic plus endophytic growth pattern typical of oral SCC  - Central eosinophilic material raises suspicion of keratin pearls  Intermediate magnification (Image 2) • Large, lamellated, whorled masses of dense pink (eosinophilic) keratin surrounded by malignant epithelial cells → keratin pearls  • Abrupt keratinisation of individual tumour cells within nests - Keratin-pearl formation is characteristic of a well-differentiated keratinising SCC  - Individual-cell keratinisation further supports squamous differentiation  High magnification (Image 3) • Tumour cells show marked cytologic atypia: enlarged, pleomorphic, hyperchromatic nuclei; irregular nuclear contours; prominent nucleoli  • Numerous mitotic figures, some atypical  • Intercellular bridges between malignant squamous cells  • Desmoplastic stroma with lymphoplasmacytic infiltrate surrounding invasive nests - Cytologic atypia and atypical mitoses confirm malignancy  - Intercellular bridges point to squamous lineage  - Stromal response (desmoplasia, inflammation) typical of invasive SCC  Synthesis  The combination of epithelial invasion, pronounced squamous differentiation (keratin pearls, intercellular bridges, individual-cell keratinisation), and significant cytologic atypia defines a well-differentiated keratinising squamous cell carcinoma rather than mere dysplasia, leukoplakia, or carcinoma in situ. |
| 11.77 | Diagnosis: Cystic squamous cell carcinoma; Key Microscopic Features: The cyst is filled with keratin and lined by dysplastic epithelium, with invasive islands of tumor cells at the margins. The cyst lining contains dyskeratotic cells, and the surface epithelium shows moderate dysplasia with infiltrating tumor islands. Conventional infiltrating squamous cell carcinoma is also observed. | Based on the provided hematoxylin and eosin (H&E) stained histopathological images, here is the analysis:  Single Most Likely Histopathological Diagnosis:  Squamous Cell Carcinoma (SCC) of the Oral Cavity  Key Microscopic Features Supporting This Diagnosis:  1. Invasive Growth Pattern (Image 1 & 4)  There is clear evidence of epithelial nests and islands infiltrating into the underlying connective tissue.  These invasive squamous islands show irregular shapes and loss of the normal epithelial architecture.  The surrounding stroma shows desmoplasia and inflammatory cell infiltration, further confirming invasion.  2. Keratin Pearl Formation (Image 3)  Presence of keratin pearls, which are concentric layers of keratinized cells within the tumor islands—a classic hallmark of well-differentiated squamous cell carcinoma.  Individual cell keratinization and intercellular bridges are also visible.  3. Cytologic Atypia and Dysplasia (Images 2, 3 & 4)  Marked nuclear pleomorphism, hyperchromasia, and increased nuclear-to-cytoplasmic ratio.  Loss of maturation of squamous epithelium, abnormal mitotic figures, and disturbed polarity, particularly evident in the basal and parabasal layers.  Disordered epithelial stratification seen in image 2, indicative of dysplasia, with the most severe atypia noted at deeper levels, consistent with invasive carcinoma.  4. Host Immune Response (Image 4)  Dense lymphoplasmacytic inflammatory infiltrate in the stroma around invading tumor nests.  Suggests ongoing immune response to the invasive carcinoma.  Conclusion:  These histological features—especially invasive squamous islands with keratin pearl formation, cytologic atypia, and desmoplasia—are diagnostic of oral squamous cell carcinoma, most likely well-differentiated based on the degree of keratinization. | Histopathological diagnosis (single best answer)  Invasive, well-differentiated (keratinising) squamous cell carcinoma of the oral mucosa  Key microscopic features visible in the photomicrographs  Feature Appearance in the submitted images Significance  Infiltrative nests & cords of squamous epithelium Multiple images show irregular islands and tongues of epithelium extending beyond the epithelial–connective-tissue junction and into the lamina propria/submucosa. Confirms invasion, distinguishing carcinoma from dysplasia/carcinoma in situ.  Keratin pearls Concentric, eosinophilic lamellated keratin whorls are seen centrally within tumour nests (especially the “round onion-skin” structures in the low-power overview and in images 3 & 4). Hallmark of a well-differentiated, keratinising SCC.  Individual-cell keratinisation Scattered tumour cells show dense, glassy eosinophilic cytoplasm even when not in pearls. Indicates squamous differentiation.  Cytologic atypia Enlarged, pleomorphic, hyperchromatic nuclei with prominent nucleoli; increased nuclear-to-cytoplasmic ratio; atypical and frequent mitotic figures (best appreciated in images 2–4). Confirms malignant nature of the epithelium.  Disordered maturation of surface epithelium The overlying mucosa shows loss of normal stratification, bulbous/drop-shaped rete ridges, basal cell hyperplasia and nuclear crowding (image 2). Represents severe epithelial dysplasia that merges with the invasive component.  Stromal desmoplasia & inflammation Tumour islands are surrounded by fibrous stroma with a mixed chronic/acute inflammatory infiltrate (images 3 & 4). Typical host response to invading SCC.  Large keratin-filled crater on low power The first image shows a big, round structure filled with laminated keratin and lined by malignant epithelium—essentially a huge keratin pearl cut in cross-section. Reinforces the diagnosis and highlights the keratinising nature.  Collectively, these features fulfill the diagnostic criteria for an invasive, well-differentiated (keratinising) squamous cell carcinoma arising in the oral cavity. |
| 11.78 | Diagnosis: NUT carcinoma; Key Microscopic Features: The tumor consists of monotonous round basaloid cells, with areas of geographic necrosis and numerous lymphocytes. There are regions of squamous differentiation, abrupt keratinization, and mitotic figures. The tumor cells have round vesicular nuclei and small nucleoli. | Histopathological Analysis of Provided H&E Images  Single Most Likely Histopathological Diagnosis:  Squamous Cell Carcinoma (SCC)  Justification and Key Microscopic Features:  1. Architectural Distortion and Infiltrative Growth Pattern (Image 1)  At low magnification, there is loss of normal epithelial architecture.  Multiple nests and islands of epithelial cells infiltrate the underlying connective tissue stroma.  These irregular epithelial islands are separated by fibrous septa, a classic hallmark of invasive SCC.  2. Dysplastic Features (Image 2)  Evidence of disordered maturation within the epithelial cells.  Cells show pleomorphism, nuclear hyperchromasia, and increased nuclear-to-cytoplasmic ratio.  Foci of keratin pearls begin to emerge in some epithelial islands, confirming squamous differentiation.  3. Keratin Pearls and Tumor Cell Nests (Image 3)  Numerous keratin pearls (eosinophilic concentric layers of keratin) are seen centrally in cell nests.  Intercellular bridges may be visible, supporting squamous differentiation.  Dyskeratosis is apparent in isolated tumor cells.  4. Cellular and Nuclear Atypia (Image 4 - High Magnification)  Marked nuclear pleomorphism, prominent nucleoli, and frequent mitotic figures, some atypical.  The presence of individual cell keratinization within invasive islands.  Necrotic debris and inflammatory cell infiltrate surrounding tumor nests.  Summary:  These findings confirm the diagnosis of well to moderately differentiated squamous cell carcinoma, an invasive malignancy arising from the squamous epithelium of the oral mucosa. The presence of keratin pearls, infiltrative epithelial nests, and pronounced cellular atypia are diagnostic hallmarks. | Histopathological diagnosis  Invasive, moderately-differentiated (keratinising) squamous cell carcinoma of the oral mucosa.  Key microscopic features seen in the provided H-E sections  Field Observations Significance  Low-power architecture • Tumour arranged in numerous irregular nests/lobules that infiltrate the submucosal stroma.  • Lobules are outlined by fibrous-vascular septa, preserving no normal glandular architecture. Confirms an invasive epithelial neoplasm rather than an in-situ process or a reactive lesion.  Invasion pattern • Islands extend beyond the presumed basement membrane into connective tissue.  • Desmoplastic stromal response around many nests. Meets the criterion for malignancy (true invasion).  Keratinisation • Central, concentric, eosinophilic “keratin pearls” within larger nests.  • Scattered individual dyskeratotic (bright pink) cells. Hallmark of keratinising (moderately-differentiated) SCC.  Cytologic atypia • Polygonal cells with eosinophilic cytoplasm and intercellular bridges.  • Marked nuclear pleomorphism, coarse chromatin, irregular nuclear membranes, occasional prominent nucleoli.  • Numerous typical and atypical mitotic figures. Indicates malignant squamous differentiation with active proliferation.  Necrosis & host response • Foci of tumour necrosis/comedo-like debris inside some lobules.  • Dense peritumoral lymphoplasmacytic infiltrate and scattered neutrophils. Common secondary changes in SCC and evidence of host immune reaction.  Together, these features—most importantly invasive nests of atypical squamous cells with keratin-pearl formation—support the diagnosis of keratinising, moderately-differentiated squamous cell carcinoma. |
| 11.80 | Diagnosis: Well-differentiated squamous cell carcinoma in a pediatric patient; Key Microscopic Features: Sheets of squamous cells exhibit abundant, pink, glassy cytoplasm. The tumor infiltrates the stroma in small cell islands, containing many dyskeratotic cells. The tumor cells show moderate cytologic atypia. | **Histopathological Analysis**  **🧠 Single Most Likely Histopathological Diagnosis:**  **Squamous Cell Carcinoma (SCC) of the oral mucosa.**  **🔬 Key Microscopic Features Justifying the Diagnosis:**  **Image 1 (Low Magnification):**  **Epithelial Invasion: There is a disruption of the basement membrane with epithelial nests infiltrating into the underlying stroma, a hallmark of invasive carcinoma.**  **Architectural Disturbance: Broad, irregular downward growth of the epithelium.**  **Keratinization: Some keratin pearl formation is subtly appreciated at this magnification.**  **Image 2 (Intermediate Magnification):**  **Irregular Nests and Islands of Atypical Squamous Cells: The invasive epithelial structures are penetrating the connective tissue, indicating true invasion beyond dysplasia or carcinoma in situ.**  **Nuclear Pleomorphism and Hyperchromatism: Marked variability in nuclear size and staining intensity.**  **Dyskeratosis and Individual Cell Keratinization: Atypical keratin production within the nests is a strong feature of SCC.**  **Image 3 (High Magnification):**  **Prominent Cellular Atypia: Enlarged, pleomorphic, hyperchromatic nuclei.**  **Abnormal Mitoses: Evidence of increased and possibly atypical mitotic figures, suggesting high proliferative activity.**  **Keratin Pearls: Circular aggregations of eosinophilic keratin surrounded by atypical squamous cells.**  **Dense Inflammatory Infiltrate: Seen around the tumor nests, which is common in invasive SCC.**  **✅ Summary:**  **The combination of invasive epithelial architecture, keratin pearl formation, marked cellular atypia, and stromal invasion is diagnostic of invasive squamous cell carcinoma. The features go beyond those seen in leukoplakia, erythroplakia, or dysplasia (even severe), confirming a malignant transformation.** | Histopathological diagnosis  Invasive well-differentiated (keratinising) oral squamous cell carcinoma.  Key microscopic features supporting this diagnosis  Field Salient findings (as seen across the three magnifications) Diagnostic implication  Surface epithelium Marked epithelial thickening with elongated, bulbous rete ridges exhibiting full-thickness cytologic atypia (loss of maturation, nuclear pleomorphism, enlarged hyperchromatic nuclei). Confirms severe dysplasia / carcinoma in situ at the mucosal surface.  Basement-membrane breach Irregular cords and nests of epithelial cells penetrate beyond the basement membrane into the underlying connective tissue stroma. Hallmark of frank invasion → separates carcinoma from “just” dysplasia.  Tumour islands in stroma Numerous nests, trabeculae and sheets of squamous cells embedded in a desmoplastic, inflamed stroma. Defines an infiltrative growth pattern.  Keratin production Concentric “keratin pearls” and individual cell keratinisation within the tumour nests. Indicates keratinising (well-differentiated) phenotype.  Cytologic atypia Cells in tumour nests show pleomorphic, vesicular to hyperchromatic nuclei, prominent nucleoli and abnormal mitotic figures. Confirms malignancy.  Host response Dense peri-tumoral lymphoplasmacytic infiltrate and stromal desmoplasia. Typical reactive pattern in invasive SCC.  Taken together, the combination of full-thickness epithelial atypia plus unequivocal stromal invasion by keratin-producing malignant squamous cells establishes the diagnosis of invasive well-differentiated squamous cell carcinoma rather than leukoplakia with dysplasia or carcinoma in situ alone. |

INFLAMMATORY SALIVARY GLAND DISORDERS

| **Fig_no** | **Ground truth** | **GPT 4o answer** | **o3 answer** |
| --- | --- | --- | --- |
| 12.3 | Diagnosis: Mucocele; Key Microscopic Features: A pool of mucin containing eosinophilic globules, surrounded by granulation tissue; part of the excretory duct showing squamous metaplasia. Surrounding tissue contains muciphages, mucin globules, and lymphocytes. | Based on the provided hematoxylin-and-eosin (H&E) stained images of a salivary gland lesion at varying magnifications, the single most likely histopathological diagnosis is:  Chronic Sclerosing Sialadenitis (Küttner Tumor)  This entity is part of the inflammatory salivary gland disorders and often affects the submandibular gland. It is considered a manifestation of IgG4-related disease.  Key Microscopic Features Justifying the Diagnosis:  1. Lobular Architecture with Extensive Fibrosis (Image 1)  The low-power image shows preserved glandular architecture with a dense sclerotic stroma replacing much of the parenchyma.  There is a central area of chronic inflammation surrounded by fibrosis, which often causes lobular distortion.  2. Mixed Inflammatory Infiltrate with Prominent Plasma Cells (Image 2)  Higher magnification highlights a dense lymphoplasmacytic infiltrate, particularly plasma cells, with lymphoid follicle formation.  This is a hallmark of chronic sclerosing sialadenitis.  3. Acinar Atrophy and Ductal Changes (Image 3)  The close-up shows atrophic acini and dilated ducts, some containing eosinophilic inspissated secretions or sialoliths (not obvious in this particular case but often seen).  There are also large plasma cells and scattered lymphocytes infiltrating glandular tissue.  4. Fibrosis with Obliterative Phlebitis (Image 4)  The final image reveals dense collagen deposition and scattered inflammatory cells.  Although obliterative phlebitis (a key feature in IgG4-RD) may not be prominent here, the dense fibrosis and mixed inflammation are suggestive.  Supporting Notes:  If confirmed immunohistochemically, one would expect a high IgG4-positive plasma cell count (>50 per high-power field) and an IgG4/IgG ratio >40%, strengthening the diagnosis.  Clinically, Küttner tumor may mimic neoplasia due to its firm consistency and enlargement. | Histopathological diagnosis (single best):  Mucocele – mucus-extravasation phenomenon (a reactive inflammatory lesion of minor salivary glands)  Key microscopic features in the provided H-E sections  Image area Characteristic findings Significance for the diagnosis  Low-power overview • A well-circumscribed, cyst-like space occupies the centre of the specimen.  • The cavity is filled with pale, eosinophilic to slightly basophilic amorphous material that lacks true internal architecture.  • The “wall” is composed of loose granulation tissue and fibrous connective tissue, not epithelium. A true cyst is ruled out by the absence of an epithelial lining; a granulation-tissue rim around pooled mucin is classic for a mucus-extravasation pseudocyst.  Intermediate magnification • The lumen contains innumerable foamy/vacuolated macrophages (“muciphages”) and scattered chronic inflammatory cells.  • Isolated pools of mucin are evident, occasionally rimmed by multinucleated giant cells attempting to phagocytose the mucin. Abundant muciphages and mucin pools are hallmarks of leaked salivary mucus being cleared by a histiocytic response.  High magnification of cell details • Foamy macrophages show finely vacuolated cytoplasm produced by ingested mucin.  • Multinucleated foreign-body giant cells are present around some mucin droplets. Confirms the reactive inflammatory nature of the lesion and supports the diagnosis of an extravasation-type mucocele rather than an epithelial-lined retention cyst or other pathology.  Peripheral tissue • Adjacent minor salivary gland lobules appear compressed/atrophic.  • Dense fibrous tissue with scattered lymphocytes encircles the lesion. Reflects the pressure of the expanding mucin pool and the chronic inflammatory/repair response expected in an established mucocele.  Diagnostic synthesis  The combination of a cyst-like mucin pool without an epithelial lining, plentiful mucin-laden macrophages and giant cells, and a rim of granulation/fibrous tissue is characteristic of a mucus-extravasation mucocele. No calcified sialolith, ductal epithelium, or ductal squamous metaplasia is identified, helping to exclude obstructive sialadenitis, retention cyst, or necrotising sialometaplasia. |
| 12.3b | Diagnosis: Mucocele; Key Microscopic Features: Mucin pools with areas of consolidation and organization; pools of mucin contain muciphages and neutrophils. | Histopathological Diagnosis:  Chronic Sclerosing Sialadenitis (Küttner Tumor)  Justification Based on Key Microscopic Features:  Low Magnification (First Image):  Lobular architecture preservation is disrupted by dense fibrosis and chronic inflammatory infiltrate, forming a sclerotic mass-like appearance.  The gland is enlarged with clear signs of periductal and interstitial fibrosis, which is characteristic of chronic sclerosing sialadenitis.  There is lymphoplasmacytic infiltrate with prominent lymphoid follicles formation, reflecting the chronic inflammatory process.  High Magnification (Second Image):  Dense lymphoplasmacytic infiltrate is present, with numerous plasma cells, lymphocytes, and scattered eosinophils.  Acinar atrophy and ductal dilation are evident, showing loss of normal glandular elements.  Presence of fibrosis with a storiform pattern may also be hinted, which aligns with sclerosing inflammation.  Occasional obliterative phlebitis may be seen in some cases of this condition (though not clearly visible in this image).  Additional Supporting Context:  Chronic sclerosing sialadenitis (Küttner tumor) often affects the submandibular gland and mimics a neoplasm clinically and radiographically. It's now considered part of the IgG4-related disease spectrum, which could be confirmed via immunohistochemistry for IgG4-positive plasma cells if necessary. | Histopathological diagnosis  → Chronic sclerosing (lymphoplasmacytic) sialadenitis – “Küttner tumour”  Key microscopic features that point to this diagnosis  Field Findings seen in the submitted H-E sections Why it supports Kuttner-type chronic sialadenitis  Low-power architecture Entire lobule is converted into a solid-looking, yet organised nodule bordered by a fibrous capsule/septa; the normal acinar architecture is largely lost. Chronic (not acute) process with sclerosis; the nodular, almost tumour-like replacement of the gland is typical of Küttner tumour.  Stroma Abundant, pale eosinophilic collagen bundles coursing through the lesion, giving a subtly storiform / whorled appearance. Progressive sclerosis is the hallmark of chronic sclerosing sialadenitis.  Inflammatory infiltrate Dense, diffuse lymphoplasmacytic infiltrate dominates the parenchyma. At high power many small round lymphocytes and numerous plasma cells are present; neutrophils or granulomas are negligible/absent. A chronic, immune-mediated pattern rather than acute suppurative or granulomatous disease. IgG4-positive plasma cells are often abundant in this entity.  Residual ductal elements Scattered residual ducts with thickened walls or mild dilatation remain within the lymphoplasmacytic background (one duct cross-section with luminal debris is visible in the high-power field). Acini are markedly atrophic or completely absent. Selective acinar atrophy with periductal fibrosis is characteristic; preservation of ducts helps distinguish it from destructive necrosis.  Fibrosis around blood vessels Collagen surrounds small vessels, sometimes partially obliterating lumina (early “obliterative phlebitis”). A supportive, although not pathognomonic, feature of IgG4-related sclerosing sialadenitis/Küttner tumour.  These combined features—diffuse lymphoplasmacytic infiltrate, marked acinar atrophy, periductal and interlobular storiform fibrosis producing a firm, mass-like nodule—are classic for chronic sclerosing sialadenitis (Küttner tumour), the prototypical inflammatory pseudotumour of the submandibular and minor salivary glands. |
| 12.4 | Diagnosis: Mucocele; Key Microscopic Features: Collapsed mucocele surrounded by granulation tissue; wall of granulation tissue containing muciphages and macrophages. | Based on the histopathological features observed in the provided hematoxylin and eosin (H&E)-stained images at various magnifications, the most likely single diagnosis is:  Chronic Sclerosing Sialadenitis (Küttner Tumor)  Key Microscopic Features Justifying the Diagnosis:  Preserved and Atrophic Salivary Gland Lobules:  Seen in the low-power image (first image), there is a lobulated architecture of the gland with areas of ductal atrophy and fibrosis, often characteristic of chronic sialadenitis.  The lobules are surrounded by dense, sclerotic fibrous stroma.  Dense Chronic Inflammatory Infiltrate:  High-power images (second and third images) reveal a prominent lymphoplasmacytic infiltrate, often forming lymphoid aggregates and germinal center-like structures.  This is typical in chronic inflammatory salivary gland disorders.  Periductal and Perilobular Fibrosis:  Notable concentric (“onion-skin”) fibrosis around ducts, suggesting chronicity and pointing toward sclerosing sialadenitis.  Obliterative Phlebitis (if present on other sections):  While not clearly shown in the provided images, this is a common and supportive feature in Küttner tumor, although not essential for diagnosis if other features are compelling.  Possible IgG4-Related Changes (not confirmable without immunohistochemistry):  The dense lymphoplasmacytic infiltrate and storiform fibrosis can also raise suspicion for IgG4-related sialadenitis, often overlapping with Küttner tumor. However, confirmation requires IgG4 immunostaining.  Summary:  The images strongly support Chronic Sclerosing Sialadenitis (Küttner Tumor) due to the presence of:  Sclerotic stroma,  Chronic inflammation,  Preserved glandular architecture with ductal atrophy.  Further immunohistochemistry (especially IgG4 staining) could provide additional insights but is not strictly necessary for the base diagnosis of Küttner tumor. | Histopathological diagnosis (single best answer)  Necrotizing sialometaplasia  Key microscopic features that support the diagnosis  Field-of-view What you can see Why it matters  Low power (first image) • Overall lobular outline of the salivary gland is still recognisable.  • A central glandular lobule that looks almost “punched-out” is surrounded by a rim of more basophilic, inflamed tissue.  • Irregular, angular empty spaces/clefts representing infarcted ducts and acini filled with necrotic debris or mucin Necrotizing sialometaplasia is an ischaemic infarct: the lobular architecture is preserved even though the acini have undergone coagulative necrosis.  Intermediate power (second image) • Necrotic eosinophilic debris in the centre of lobules.  • Islands and cords of bland squamoid epithelium lining or replacing ducts/acini (squamous metaplasia).  • Mixed acute-on-chronic inflammatory infiltrate (neutrophils, lymphocytes, plasma cells) surrounding the necrotic zone. The combination of coagulative necrosis plus squamous metaplasia of salivary ducts/acini is the histological hallmark of this lesion and can mimic carcinoma if the bland cytology and preserved architecture are not appreciated.  High power (third image) • Overlying mucosa shows pseudo-epitheliomatous hyperplasia without true dysplasia; surface may be ulcerated.  • Metaplastic squamoid cells have small, uniform nuclei, inconspicuous nucleoli, and lack significant mitotic activity or atypia.  • Granulation tissue and small vessel proliferation at the interface between viable and necrotic tissue. Pseudo-epitheliomatous hyperplasia and bland squamous nests are classic for necrotizing sialometaplasia and help separate it from squamous-cell or mucoepidermoid carcinoma.  Putting it together  Coagulative necrosis of seromucous acini with retention of lobular architecture.  Squamous (sometimes mucinous) metaplasia of ducts and residual acini forming bland epithelial islands/cords.  Pseudo-epitheliomatous hyperplasia of the surface epithelium, often with ulceration.  Mixed inflammatory background and early granulation tissue.  Absence of true cytologic atypia, pleomorphism, or invasive growth pattern.  These features, taken together, are pathognomonic for necrotizing sialometaplasia, a self-limiting, infarct-type inflammatory disorder of salivary tissue.  2/2 |
| 12.5 | Diagnosis: Mucocele; Key Microscopic Features: Diffuse mucin in the interstitium without defined mucin pools; abundant mucin in the interstitial space with muciphages. | Based on the provided hematoxylin and eosin (H&E) stained images of a salivary gland lesion at low and high magnification, here is the analysis:  Single Most Likely Histopathological Diagnosis:  Chronic Sclerosing Sialadenitis (Küttner Tumor)  Key Microscopic Features Supporting the Diagnosis:  Low Power Image:  Architectural distortion of the salivary gland.  Dense fibrosis replacing much of the glandular parenchyma.  Patchy chronic inflammatory infiltrate throughout the glandular stroma.  Lobular architecture partially retained but with significant parenchymal atrophy.  High Power Image:  Lymphoplasmacytic infiltrate—prominent chronic inflammation dominated by lymphocytes and plasma cells.  Periductal and perivascular inflammation.  Acinar atrophy—loss of normal serous acini.  Fibrosis with collagen deposition, appearing as pale pink eosinophilic material between the remaining ducts and vessels.  Occasional reactive ductal epithelial changes.  Supporting Discussion:  Chronic Sclerosing Sialadenitis (CSS), also known as Küttner tumor, is a benign inflammatory condition affecting the submandibular gland, often presenting as a firm mass clinically mimicking a neoplasm. It is characterized histologically by progressive fibrosis, dense lymphoplasmacytic infiltration, and acinar atrophy—findings all present in your images.  This condition may also be part of the IgG4-related disease spectrum, which can be confirmed by IgG4 immunohistochemistry if clinically indicated. | Histopathological diagnosis (single best):  Necrotizing sialometaplasia  Key microscopic features that support this diagnosis  Field Observations in the provided slides Diagnostic significance  Low-power architecture Wedge-shaped, infarct-like zone of injury located immediately beneath the surface mucosa; overall lobular outline of the minor salivary gland is still recognizable. Classic configuration for necrotizing sialometaplasia – infarct with preserved lobular architecture despite extensive necrosis.  Acinar changes Numerous acini appear pale, “ghost-like,” and coagulated; many have lost nuclear detail. Coagulative necrosis of seromucous acini is an early hallmark of the lesion.  Ductal/acinoductal epithelium Residual viable ducts show squamoid transformation, forming irregular islands and cords of bland squamous epithelium (sialometaplasia). No cytologic atypia or abnormal mitoses are present. Squamous metaplasia of salivary ducts/acini – the feature that lends the lesion its name and can mimic carcinoma.  Stroma & exudate Pools of eosinophilic debris, extravasated erythrocytes, and lakes of spilled mucin; mixed inflammatory infiltrate (neutrophils, lymphocytes, histiocytes). Hemorrhage, mucin spillage, and secondary inflammation are typical in the necrotic phase.  Overlying epithelium Intact but hyperplastic (pseudo-epitheliomatous hyperplasia) rather than ulcerated. Surface hyperplasia/ulceration is frequently present and may simulate invasive disease.  Cytologic features Metaplastic squamous cells are bland with small, uniform nuclei and absent keratin “pearls.” Lack of atypia helps distinguish the process from true squamous cell carcinoma.  Taken together, the lobular pattern of coagulative necrosis coupled with bland squamous metaplasia of ducts/acini and an accompanying inflammatory reaction is diagnostic of necrotizing sialometaplasia, an ischemic, self-healing inflammatory disorder of minor salivary glands. |
| 12.5b | Diagnosis: Mucocele; Key Microscopic Features: Intraglandular mucocele with acinar destruction, surrounded by mucin and muciphages. | Histopathological Diagnosis:  Chronic Sclerosing Sialadenitis (Küttner Tumor)  Key Microscopic Features Supporting the Diagnosis:  Lobular Architecture with Dense Chronic Inflammation:  The low-power image shows a preserved lobular architecture of the salivary gland (likely submandibular) with areas of dense lymphoplasmacytic infiltrate, replacing much of the normal acini.  The inflammation is predominantly lymphocytic, involving and often obscuring the glandular tissue.  Marked Fibrosis (Sclerosis):  There is prominent stromal fibrosis or sclerosis, especially in the central and periductal regions, giving the gland a firm consistency.  This fibrosis replaces acinar structures and contributes to the loss of normal glandular architecture.  Acinar Atrophy:  The high-power image shows extensive acinar atrophy, with only scattered residual acinar structures.  Remaining ducts are often surrounded by fibrosis and inflammatory cells.  Lymphoid Follicles:  Germinal center formation is noted, indicating a long-standing inflammatory process.  These are common in chronic inflammatory conditions and help differentiate from acute forms.  Dilated Ducts with Oncocytic or Eosinophilic Metaplasia:  Some ducts appear dilated with thickened, eosinophilic ductal epithelium—possibly representing metaplastic changes due to chronic obstruction/inflammation.  Correlative Clinical Considerations:  Most commonly affects the submandibular gland.  Clinically presents as a firm, painless mass, often mistaken for neoplasm.  Associated in some cases with IgG4-related disease, especially when plasma cell-rich infiltrates are present.  If immunohistochemistry were available, staining for IgG4-positive plasma cells would help confirm the association with IgG4-related disease, although it is not necessary for the histological diagnosis of Küttner tumor itself. | Histopathological diagnosis (single best fit)  Necrotizing sialometaplasia (ischemic‐infarctive inflammatory lesion of minor salivary glands).  Key microscopic features that support this diagnosis  Field Observations Diagnostic significance  Overall architecture (low-power image) Lobular outline of the gland is still recognizable despite extensive central damage. No expansile, encapsulated or infiltrative mass is present. Preservation of lobular architecture is characteristic of a reactive/ischemic process rather than a true neoplasm.  Acinar changes (“ghost-acini”) Numerous rounded pale eosinophilic rings representing coagulative necrosis of mucous acini; nuclei are lost, leaving vacuolated or “faded” centres. Classic hallmark of necrotizing sialometaplasia; reflects sudden ischemic infarction of mucous acini.  Ductal/epithelial response Surviving ducts show squamous (metaplastic) lining and fragment into ragged epithelial islands within the necrotic bed (best appreciated in higher-power view). Squamous metaplasia of ducts is so typical that the lesion can mimic mucoepidermoid or squamous carcinoma, but here the changes are confined to pre-existing ductal structures, not invading stroma.  Stromal background Granulation tissue and mixed inflammatory infiltrate (neutrophils early, lymphocytes/plasma cells later) fill the necrotic area; some extravasated mucin with foamy macrophages is present. Reflects the reactive inflammatory phase that follows infarction.  Lack of true neoplastic features No dysplasia of overlying mucosa, no perineural or vascular invasion, and mitoses are scarce. Helps distinguish from malignant salivary tumours that necrotizing sialometaplasia can clinically and histologically mimic.  Taken together—ghost mucous acini within an otherwise intact lobular framework, squamous metaplasia of pre-existing ducts, and an accompanying inflammatory/granulation tissue response—the pictures are classic for necrotizing sialometaplasia, an inflammatory/ischemic disorder of salivary glands. |
| 12.8 | Diagnosis: Chronic Obstructive Sialadenitis; Key Microscopic Features: Widespread gland involvement with acinar atrophy, duct dilation, interstitial fibrosis, and chronic inflammation. Periductal inflammation may also occur in reactive sialadenitis from mucoceles. | Histopathological Diagnosis:  Chronic Sclerosing Sialadenitis (Küttner Tumor)  Key Microscopic Features Supporting the Diagnosis:  Lobular Architecture with Extensive Fibrosis:  All three images show preservation of lobular salivary gland structure with dense fibrous tissue proliferation, replacing and entrapping acini and ducts.  This is characteristic of chronic sclerosing sialadenitis.  Acinar Atrophy and Ductal Changes:  Noticeable atrophy of acinar cells is evident, with ducts appearing dilated or distorted.  Some ducts show squamous metaplasia or periductal fibrosis, a common response to chronic inflammation.  Prominent Lymphoplasmacytic Infiltrate:  A dense infiltrate of lymphocytes and plasma cells is seen throughout the gland, particularly surrounding ducts and blood vessels.  In Image 1, a lymphoid follicle with germinal center formation is evident — a hallmark of chronic immune-mediated inflammation.  Fatty Replacement and Ductal Obliteration:  The gland shows fatty infiltration, especially in image 3, consistent with long-standing inflammation and tissue destruction.  Ducts may also appear obstructed, suggesting a background of ductal stenosis or sialolithiasis.  Absence of Granulomas or Specific Infection:  There are no granulomatous formations, necrosis, or obvious microorganisms — ruling out infectious sialadenitis or granulomatous conditions like sarcoidosis or tuberculosis.  Conclusion:  The constellation of periductal fibrosis, acinar atrophy, lymphoplasmacytic infiltrate with germinal centers, and fatty replacement strongly supports the diagnosis of Chronic Sclerosing Sialadenitis (Küttner Tumor) — a benign but chronic inflammatory condition typically affecting the submandibular gland. | Histopathological diagnosis  → Necrotising sialometaplasia  Microscopic hall-marks seen in the three fields  Low-, medium- & high-power findings How they point to necrotising sialometaplasia  1. Preservation of normal lobular outline – the gland is still divided into lobules by delicate septa (best appreciated in the lowest-power image, 3rd slide). Tells us the process is an infarct of an existing gland, not a true neoplasm that destroys the basic architecture.  2. Coagulative (“ghost-like”) necrosis of seromucous acini – pale, eosinophilic acini with loss of nuclei and pools of extravasated mucin/haemorrhage occupy the centre of lobules. The defining “infarct-like” change of necrotising sialometaplasia.  3. Squamous metaplasia of ducts & residual acini – ducts are converted into thick, stratified squamoid epithelium that may form irregular tubular or island-shaped profiles with central lumina (best seen in the higher-power image, 2nd slide). Characteristic metaplastic response that can mimic a carcinoma but the cells are cytologically bland.  4. Bland cytology – nuclei are small, uniform, with no significant pleomorphism or mitotic activity. Helps exclude true squamous-cell or muco-epidermoid carcinoma.  5. Mixed inflammatory/repair response – peripheral lympho-plasmacytic infiltrate, occasional organised lymphoid follicle, granulation tissue and focal haemorrhage. Explains the self-healing, inflammatory nature of the lesion.  6. Mucin pools & ragged ulcerated/necrotic surface (seen as empty or weakly basophilic spaces surrounded by metaplastic ducts). Another typical accompaniment of infarction of mucous acini.  Taken together, the triad of lobular infarction + squamous metaplasia of ducts/acini + maintenance of overall glandular architecture is diagnostic of necrotising sialometaplasia, a self-limiting inflammatory/ischaemic disorder of salivary tissue. |
| 12.9 | Diagnosis: Mucocele; Key Microscopic Features: An organizing mucocele characterized by a compact mass of granulation tissue with surrounding fibrosis. | Based on the histopathological analysis of the provided hematoxylin and eosin (H&E) stained images, captured at low and high magnifications respectively, the single most likely diagnosis is:  Necrotizing Sialometaplasia  Key Microscopic Features Supporting the Diagnosis:  1. Lobular Architecture Preservation (Low Magnification Image)  Despite the extensive tissue alteration, the overall lobular architecture of the salivary gland is preserved.  This is a hallmark of necrotizing sialometaplasia and helps distinguish it from invasive malignancies.  2. Coagulative Necrosis of Glandular Acini  The glandular structures show areas of necrosis, with loss of nuclei but preservation of the tissue outline.  Necrotic debris can be observed within the lobule.  3. Squamous Metaplasia of Ductal Epithelium (High Magnification Image)  Ducts and acini show squamous metaplasia—epithelial cells have transformed into squamous-like cells.  This may mimic squamous cell carcinoma but is benign in this context.  4. Intense Inflammatory Infiltrate  Mixed inflammatory infiltrate consisting predominantly of neutrophils, lymphocytes, and plasma cells.  Inflammation surrounds and infiltrates the necrotic salivary tissue.  5. Pseudoepitheliomatous Hyperplasia (sometimes seen)  While not clearly evident in these images, this reactive epithelial proliferation is often present and can mimic carcinoma.  Clinical Correlation:  Necrotizing sialometaplasia is a benign, self-limiting inflammatory condition, often triggered by ischemic injury (e.g., trauma, surgery, smoking, or local anesthesia), typically affecting the minor salivary glands of the palate.  Differential Diagnosis to Rule Out:  Mucoepidermoid carcinoma (ruled out due to lack of infiltrative growth and atypical mitoses).  Squamous cell carcinoma (ruled out due to preservation of lobular architecture and lack of deep invasion).  Chronic sialadenitis (would show fibrosis and ductal ectasia rather than necrosis and metaplasia). | Histopathological diagnosis  → Mucous-extravasation phenomenon (extravasation mucocele)  Microscopic features that point to this diagnosis  Area of slide What is seen Why it matters  Low-power view Sharply circumscribed, lobular nodule lying in the submucosa and surrounded by a rim of compressed fibrous tissue Typical outline of an organised pool of spilled mucus rather than a true cystic cavity  Central/irregular pale material (string-like white cleft in the image) Represents extravasated mucin that has leaked from a ruptured minor-salivary-gland duct  No epithelial lining to the cavity Confirms an extravasation phenomenon rather than a true epithelial cyst  High-power view Numerous foamy macrophages (mucinophages) with abundant pale, vacuolated cytoplasm and small dark eccentric nuclei These macrophages are actively phagocytosing the spilled mucin – a hallmark of mucoceles  Mixed chronic inflammatory infiltrate of lymphocytes and a few plasma cells Part of the granulation-tissue response that walls off the mucin pool  Delicate granulation-tissue stroma with thin-walled capillaries Indicates organisation and repair of the lesion  Peripheral glands (not in the field shown but classically present) Atrophic or dilated minor salivary gland acini/ducts near the lesion Source of the mucus leak  The combination of extravasated mucin without an epithelial lining, a cuff of granulation tissue packed with mucin-laden macrophages, and a chronic inflammatory background is diagnostic of a mucous-extravasation mucocele rather than other inflammatory salivary-gland disorders. |
| 12.9b | Diagnosis: Mucocele; Key Microscopic Features: An organizing mucocele with a dense fibrovascular tissue mass surrounded by mucin globules and fibrous/granulation tissue. | Based on the provided hematoxylin and eosin (H&E) stained histopathology images of a salivary gland lesion at low and higher magnifications, the single most likely histopathological diagnosis is:  Chronic Granulomatous Sialadenitis  Key Microscopic Features Justifying the Diagnosis:  Granuloma Formation:  The central feature in the higher magnification image shows a well-formed granuloma, with collections of epithelioid histiocytes (activated macrophages).  Occasional multinucleated giant cells are visible.  Surrounding lymphocytic infiltration is noted, a hallmark of chronic inflammation.  Chronic Inflammatory Infiltrate:  Dense lymphoplasmacytic infiltrates are seen extensively in the salivary gland parenchyma.  This reflects chronic inflammation rather than acute suppurative changes.  Destruction of Salivary Gland Architecture:  Normal acinar and ductal structures are largely effaced or replaced by granulomatous inflammation and fibrosis.  There is a marked loss of glandular elements.  Fatty Infiltration:  In the lower magnification image, scattered vacuolated areas consistent with fatty replacement can be appreciated, which is common in chronic sialadenitis.  Differential Considerations and Exclusion:  Necrotizing granulomas (e.g., tuberculosis): No central necrosis observed.  Sarcoidosis: Granulomas in sarcoidosis are typically non-caseating and well-formed, but would require correlation with systemic features and special stains.  Sjögren’s syndrome: Usually presents with focal lymphocytic sialadenitis and epimyoepithelial islands, not granulomas.  Conclusion:  The presence of granulomas with associated chronic inflammation, destruction of normal salivary gland tissue, and fibrosis supports a diagnosis of chronic granulomatous sialadenitis, which may be due to infections (e.g., mycobacterial), immune-mediated causes, or idiopathic granulomatous inflammation. Special stains (e.g., Ziehl–Neelsen, PAS) and clinical correlation are advised for definitive etiological classification. | Histopathological diagnosis  → Necrotizing sialometaplasia (NS)  Microscopic features that point to NS  Area Key finding Diagnostic significance  Central zone • Pale, eosinophilic “ghost” outlines of acini and mucous cells indicating coagulative (ischemic) necrosis.  • Pools of faintly basophilic-pink coagulum without viable nuclei. The hallmark of NS is infarction of a whole salivary lobule with preservation of its basic outline.  Duct / acinar epithelium • Numerous duct-like and island-shaped structures now lined by bland squamous cells (squamous metaplasia). Characteristic metaplasia produces epithelial islands that can mimic carcinoma but lack cytologic atypia or infiltrative growth.  Lobular architecture • Despite necrosis, the overall lobular pattern of the gland is maintained rather than effaced. Distinguishes NS from invasive malignancy or diffuse chronic sialadenitis.  Stroma & vessels • Mixed chronic inflammatory infiltrate (lymphocytes ± neutrophils) and early granulation-type reaction around necrotic lobules.  • Reactive dilated blood vessels and focal hemorrhage. Reflects the self-limited inflammatory response to the ischemic injury.  Surface mucosa (seen at left edge of low-power slide) • Pseudoepitheliomatous hyperplasia / ulceration of overlying oral epithelium. Typical in palatal NS and another potential pitfall for mis­diagnosis as SCC.  Taken together—lobular infarction with ghost acini, squamous metaplasia of ducts, preservation of architecture, and a surrounding inflammatory/granulation response—the slide depicts the classic picture of necrotizing sialometaplasia, an ulcer-like, self-healing inflammatory disorder of minor salivary glands. |
| 12.10 | Diagnosis: Mucocele; Key Microscopic Features: Granulation tissue surrounds a cyst-like structure; muciphages and clear cells present in the wall of the granulation tissue. | Based on the provided hematoxylin-and-eosin (H&E) stained histological images at low and high magnifications, the single most likely histopathological diagnosis is:  Necrotizing Sialometaplasia  Justification & Key Microscopic Features:  Low-Magnification Image (First Image):  Lobular architecture is preserved despite extensive tissue changes, which is a hallmark of necrotizing sialometaplasia.  Coagulative necrosis of salivary gland acini and ducts is evident — central zones show pale, eosinophilic, anucleate areas indicative of necrosis.  Inflammatory infiltrate, predominantly neutrophilic and lymphoplasmacytic, is seen around necrotic areas.  Pseudoepitheliomatous hyperplasia may also be suspected at this magnification but is better confirmed at higher resolution.  High-Magnification Image (Second Image):  Squamous metaplasia of salivary ducts: the ducts are lined by squamoid or stratified epithelium rather than their normal cuboidal/columnar lining.  Acinar necrosis: residual ductal structures are surrounded by necrotic acinar debris.  Lobular preservation: even amidst necrosis and squamous metaplasia, the general lobular architecture of the salivary gland remains identifiable.  Minimal cellular atypia, distinguishing it from malignancy.  Summary Diagnosis:  ✅ Necrotizing Sialometaplasia  Clinical Relevance:  Necrotizing sialometaplasia is a benign, self-limiting inflammatory condition of the salivary glands, most commonly affecting the minor glands of the palate. It can clinically and histologically mimic malignancy (like mucoepidermoid carcinoma or squamous cell carcinoma), but lacks cellular atypia and shows preservation of lobular architecture with squamous metaplasia and necrosis. | Histopathological diagnosis  Necrotising sialometaplasia (ischaemic-infarctive inflammatory lesion of salivary gland tissue)  Key microscopic features supporting this diagnosis  Field What is seen Why it points to necrotising sialometaplasia  Low-power architecture (first image) • A single, well-circumscribed lobule of salivary gland is still recognisable.  • The centre of the lobule is replaced by homogenously eosinophilic, “ghost” acini – coagulative necrosis – while the lobular outline remains intact.  • Curvilinear, ragged, duct-like or island-shaped structures course through the necrotic zone. Preservation of the overall lobular silhouette with central infarction is characteristic of NSM. The duct/island structures represent squamous-metaplastic ducts and can mimic carcinoma, a classic diagnostic pit-fall of this lesion.  Surface epithelium / capsule Thin fibrous tissue and reactive granulation tissue rim the focus; no true invasive front. NSM is non-neoplastic and shows no infiltrative growth beyond the infarcted lobe.  High-power cellular detail (second image) • Numerous foamy (lipid/mucin-laden) macrophages with vacuolated cytoplasm are scattered between the necrotic acini.  • A mixed inflammatory infiltrate—neutrophils, lymphocytes, plasma cells—is present, but cytologic atypia is absent.  • No orderly lobules of adipocytes; the clear cells are histiocytes, not fat. The combination of coagulative necrosis, xanthomatous histiocytes and mixed inflammation is typical of the early “infarct/cleanup” stage of NSM. Lack of cytologic atypia helps distinguish it from squamous cell carcinoma or mucoepidermoid carcinoma.  Metaplastic epithelium Duct-like/strand-like spaces are lined by flattened to squamoid epithelium without dysplasia or keratin pearls. Squamous metaplasia of residual ducts is the lesion’s namesake (“sialo-metaplasia”) and, together with necrosis, is diagnostic when the architecture is preserved.  Collectively, these findings—lobular infarction with ghost acini, squamous-metaplastic ducts/islands, abundant foamy macrophages, and a mixed inflammatory reaction—constitute the classic histological picture of necrotising sialometaplasia. |
| 12.10b | Diagnosis: Mucocele; Key Microscopic Features: Collapsed mucocele with clear cells and glands showing significant chronic sialadenitis; muciphages mixed with clear cells and mucin globules. | Histopathological Analysis of the Provided H&E-Stained Images  Single Most Likely Histopathological Diagnosis:  Sialadenitis (specifically, chronic sialadenitis with xanthomatous features)  Key Microscopic Features Supporting the Diagnosis:  Lobular Architecture Distortion (first image - low power):  The normal salivary gland architecture is disrupted.  There is a dense inflammatory infiltrate, particularly around ducts and acini.  Fibrosis is visible, with splaying and separation of lobules.  Chronic Inflammation:  Lymphocytic infiltrate is prominent in the interstitial areas.  Plasma cells and occasional germinal centers may be seen, suggesting chronicity.  Xanthomatous (Foamy) Histiocytes (second image - high power):  The presence of numerous pale-staining, lipid-laden macrophages (foamy histiocytes) in the stroma is a hallmark of xanthomatous inflammation.  These cells have a vacuolated cytoplasm and small central nuclei.  Acinar Atrophy:  At high magnification, residual acini are sparse or atrophic.  Ductal elements may be dilated, showing signs of ductal damage or obstruction.  Fibrosis:  Interstitial fibrotic changes are evident, often associated with chronicity.  Fibrous tissue may partially replace glandular elements.  Conclusion:  These histological features are most consistent with chronic xanthomatous sialadenitis, a chronic inflammatory condition of the salivary glands characterized by persistent inflammation, acinar atrophy, fibrosis, and foamy macrophage infiltration. | Histopathological diagnosis – Necrotising sialometaplasia  Key microscopic features visible in the two fields  Feature Where it is seen Why it points to necrotising sialometaplasia  1. Intact lobular outline of the salivary gland despite destruction of the acini Low-power image (whole lesion retains a “lobulated” architecture within the submucosa) Necrotising sialometaplasia is an infarct-type injury – the gland’s overall framework is preserved even though the parenchyma has died.  2. Coagulative necrosis of acini – pale, eosinophilic, ghost-like acinar remnants and empty spaces Low-power and high-power Characteristic early change after ischaemic infarction of salivary tissue.  3. Squamous / pseudoepithelial metaplasia of ducts and residual acini forming irregular islands and cyst-like spaces Low-power: small, angular, eosinophilic ductal profiles scattered through the necrotic lobules Hallmark reaction that can mimic squamous carcinoma but, here, remains confined to pre-existing duct/acinar sites.  4. Pools of extravasated mucin with abundant foamy (xanthomatous) macrophages High-power: numerous clear-vacuoled histiocytes with small dark nuclei, intermixed with chronic inflammatory cells Mucin leaked from ruptured acini is phagocytosed by macrophages, producing the striking xanthomatous appearance.  5. Mixed inflammatory infiltrate (lymphocytes ± neutrophils) and early granulation-type fibrosis at the periphery Both magnifications Reflects the reactive/repair phase that follows the infarct.  6. Overlying surface epithelium spared or only minimally ulcerated (no infiltrative growth into it) Low-power, top of section Helps separate this self-limiting lesion from an invasive malignancy.  Taken together, the preserved lobular outline with central coagulative necrosis, squamous metaplasia of ducts, xanthomatous macrophage response to spilled mucin, and mixed inflammation are classic for necrotising sialometaplasia, the prototypical inflammatory/infarctive lesion in salivary gland pathology. |
| 12.11 | Diagnosis: Mucocele; Key Microscopic Features: Presence of synovium-like structures in the mucocele, with papillary synovium-like structures protruding into a mucin pool and synovium-like areas containing fibroblasts, macrophages, and capillaries. | Histopathological Diagnosis:  Chronic Sclerosing Sialadenitis (Küttner Tumor)  Justification Based on Microscopic Features:  The provided hematoxylin and eosin-stained images at low and high magnification show several key features characteristic of Chronic Sclerosing Sialadenitis, an inflammatory condition primarily affecting the submandibular salivary gland.  Key Microscopic Features:  🔬 Low-Power View (First Image):  Lobular architecture distortion: The normal salivary gland acinar structure is largely replaced by dense fibrous tissue.  Acinar atrophy: The glandular parenchyma is markedly reduced with residual ductal structures.  Prominent fibrosis: There is widespread stromal sclerosis that replaces much of the glandular tissue.  Chronic inflammatory infiltrate: A dense lymphoplasmacytic infiltrate is scattered throughout the lesion.  🔬 High-Power View (Second Image):  Lymphoplasmacytic infiltrate: Dominated by lymphocytes and plasma cells, infiltrating and sometimes forming germinal center-like aggregates.  Periductal inflammation: The inflammation tends to center around and sometimes invade residual ducts.  Obliterative phlebitis (suggested but not definitive): Some vessels may show partial luminal obliteration, a feature often present in IgG4-related disease.  Acinar dropout and ductal dilation: Atrophic acini and dilated ducts with inspissated material are visible.  Additional Notes:  Küttner tumor is now recognized as part of the IgG4-related disease spectrum, and in clinical practice, IgG4 immunostaining might be used to confirm the diagnosis.  The lesion is benign but can mimic malignancy due to firm consistency and deep location. | Histopathological diagnosis  → Necrotizing sialometaplasia (ischemic-infarctive inflammatory lesion of a minor salivary gland)  Key microscopic features visible in the two H-&-E sections that support this diagnosis  Low-power panorama (first image) Higher magnification detail (second image) Diagnostic significance  ✔ Lobular outline of the gland is still recognizable; pale “ghost” lobules alternate with more intact, basophilic ones. ✔ Numerous ducts are lined by bland squamous epithelium (squamous metaplasia) forming irregular nests and tubules. Classical combination of coagulative necrosis of acini with squamous metaplasia of ducts while the basic lobular architecture is preserved.  ✔ Extensive areas of pink, structure-less material replacing mucous acini (ischemic coagulative necrosis). ✔ Metaplastic epithelial islands show smooth outlines, small uniform nuclei, and no cytologic atypia or mitoses, helping to distinguish the lesion from a true squamous carcinoma or mucoepidermoid carcinoma. Necrosis plus cytologically bland squamous islands is virtually pathognomonic for necrotizing sialometaplasia.  ✔ Pools of pale amphophilic material and empty clefts around lobules—interpreted as extravasated mucin and dissolved necrotic acinar contents. ✔ A mixed inflammatory infiltrate (neutrophils, lymphocytes, scattered foamy macrophages) and foci of hemorrhage within the stroma. Necrotic debris and mucin elicit secondary inflammation, fitting an infarctive/inflammatory process rather than a neoplasm.  ✔ Surface oral mucosa at the top shows pseudo-epitheliomatous hyperplasia with reactive keratinising epithelium. — Reactive epithelial thickening is another classical feature and a frequent cause of clinical and microscopic confusion with carcinoma.  Taken together, the preserved lobular framework, widespread coagulative necrosis of mucous acini, ductal squamous metaplasia forming bland epithelial nests, mucin extravasation with inflammation, and overlying pseudo-epitheliomatous hyperplasia form the diagnostic constellation of necrotizing sialometaplasia, an ischemic inflammatory disorder of salivary glands. |
| 12.12 | Diagnosis: Superficial Mucocele; Key Microscopic Features: Mucin pool is adjacent to and thins the epithelium that forms a peripheral collarette, with mucin present in connective tissue and causing obstructive changes in salivary glands. | Histopathological Diagnosis:  Mucous Extravasation Phenomenon (Mucocele)  Key Microscopic Features Justifying the Diagnosis:  Cyst-like cavity without epithelial lining:  Both images clearly show a large central cystic space filled with pale, amorphous material (likely mucin), which lacks an epithelial lining, a hallmark of a mucous extravasation phenomenon rather than a true cyst (which would have epithelium).  Granulation tissue surrounding the cystic space:  The wall of the cavity is composed of granulation tissue with chronic inflammatory cells, including lymphocytes and macrophages. This is consistent with the tissue's response to spilled mucin from ruptured salivary ducts.  Presence of mucinophages (foamy macrophages):  The surrounding tissue contains foamy macrophages, also known as mucinophages, which have ingested mucin. These cells appear as vacuolated or pale-staining cells within the granulation tissue.  Adjacent minor salivary gland acini:  Residual minor salivary glands are visible at the periphery of the lesion, often with evidence of ductal rupture or damage. These glands are the likely source of the spilled mucin. | Histopathological diagnosis  → Mucocele (mucus-extravasation phenomenon of a minor salivary gland)  Key microscopic features that support the diagnosis  Feature seen in the photomicrographs Significance  Large, irregular, empty-appearing cavity occupying the connective tissue (the lumen stains pale/white because the dissolved mucin has been washed out during processing) Represents a pool of spilled mucin rather than a true cystic space  Absence of an epithelial lining around the cavity; instead the wall is composed of loose fibrous/granulation tissue with plump fibroblasts, thin-walled capillaries and chronic inflammatory cells Confirms this is a pseudocyst produced by extravasation of mucus, not a duct-derived (retention) cyst  Numerous foamy macrophages (muciphages) and scattered neutrophils/lymphocytes within the peripheral granulation tissue Typical foreign-body-type inflammatory response to extracellular mucin  Intact overlying oral mucosal epithelium that may be thinned or slightly stretched Reflects submucosal location of the lesion and the clinical “blister-like” appearance of a mucocele  Adjacent normal-looking minor salivary gland lobules with mucous acini and occasionally dilated ducts Identifies the anatomic source of the extravasated mucin  The combination of a mucus-filled, non-epithelial-lined cavity surrounded by granulation tissue and muciphages, situated immediately next to minor salivary gland tissue, is diagnostic of a mucocele / mucus-extravasation phenomenon. |
| 12.13 | Diagnosis: Superficial Mucocele; Key Microscopic Features: Mucin pool is adjacent to and thins the epithelium that forms a peripheral collarette, with mucin pool surrounded by surface epithelium and granulation tissue at the base. | Histopathological Analysis of Provided H&E-Stained Images  🦠 Single Most Likely Histopathological Diagnosis:  Mucocele (Mucous Extravasation Phenomenon)  🔬 Key Microscopic Features Justifying the Diagnosis:  Cyst-like Space Without an Epithelial Lining (Pseudocyst):  The central cavity seen in both low and higher magnification images lacks a true epithelial lining, a hallmark feature of a mucocele (distinguishing it from a mucous retention cyst).  The cavity is filled with pale, eosinophilic mucin and scattered inflammatory cells.  Spilled Mucin:  The lumen contains extravasated mucin, which appears as an amorphous, pale area, often with associated foamy macrophages or neutrophils scattered throughout.  Granulation Tissue and Inflammatory Response:  Surrounding the mucin pool is granulation tissue characterized by fibroblasts, small blood vessels, and chronic inflammatory infiltrate (mainly lymphocytes and histiocytes).  This reflects a tissue reaction to mucin extravasation.  Compressed Salivary Gland or Ductal Tissue Nearby:  In many cases, remnants of minor salivary glands or dilated ducts may be visible in the peripheral tissue (less evident here, possibly due to sectioning).  🧠 Additional Context:  Mucoceles most commonly arise due to trauma (e.g., biting the lower lip), which causes rupture of a minor salivary gland duct and spillage of mucin into the surrounding connective tissue.  They are frequent in younger individuals and are often located on the lower lip. | Histopathological diagnosis – single best answer  Mucous extravasation phenomenon (conventional oral mucocele)  Key microscopic features in the two H-E sections that point to this diagnosis  Observed feature Diagnostic significance  Large, circumscribed cavity filled with pale, vacuolated eosinophilic material (mucin) Represents pooled saliva that has leaked from a ruptured minor salivary-gland duct.  No true epithelial lining to the cavity (i.e., a “pseudocyst”) – the wall is composed of compressed granulation-type fibrous tissue Distinguishes an extravasation mucocele from a mucous retention (duct) cyst, which has a ductal epithelial lining.  Rim of chronic inflammatory cells and numerous foamy macrophages (muciphages) within the fibrous wall and floating in the mucin Characteristic response to free mucin in the connective tissue.  Intact overlying oral stratified squamous epithelium, thinned and stretched across the dome of the lesion Typical of a submucosal spillage lesion rather than an intra-epithelial process.  Adjacent minor salivary-gland acini and occasionally a ruptured/dilated duct at the periphery Provides the anatomic source of the extravasated saliva.  Taken together, the combination of an unlined mucin pool, surrounding granulation tissue with muciphages, and proximity to minor salivary tissue is classic for a mucous extravasation phenomenon (common oral mucocele). |
| 12.15 | Diagnosis: Salivary Duct Cyst; Key Microscopic Features: Normal excretory salivary duct is adjacent and in continuity with the surface epithelium, and the lining consists of a double layer of low cuboidal-to-columnar cells with apocrine snouting. | Histopathological Diagnosis:  Mucocele (Mucous Extravasation Phenomenon)  Key Microscopic Features Supporting the Diagnosis:  Cyst-like cavity without epithelial lining:  Seen prominently in the low-power image, the lesion exhibits a large, irregular cavity filled with mucin but lacks an epithelial lining, which is characteristic of mucous extravasation (not a true cyst).  Surrounding granulation tissue and inflammation:  The cavity is surrounded by granulation tissue with numerous small blood vessels and scattered inflammatory cells (mainly lymphocytes and macrophages), which is typical of the tissue response to leaked mucin.  Mucin-filled space:  The central area is filled with pale to clear mucin, consistent with extravasated salivary gland secretion.  Spilled mucin and foamy macrophages (muciphages):  In the high-power image, macrophages with foamy cytoplasm are visible, indicating phagocytosis of mucin.  Overlying normal mucosa or minor salivary gland tissue:  The overlying mucosa appears relatively unremarkable, and minor salivary gland lobules may be nearby or displaced, which is a typical anatomic context for mucoceles. | Histopathological diagnosis  Salivary duct cyst (-“mucous-retention” cyst)  Key microscopic features supporting this diagnosis  Field Observations Diagnostic significance  Cyst architecture (low-power view) A single, well-circumscribed unilocular cavity occupies the specimen. The lumen is empty/appears “washed-out” (mucin lost in processing). The wall is composed of fibrous connective tissue and shows gentle papillary/undulating folds into the lumen. A true epithelial-lined cyst rather than an extravasated mucin pool. Favors a retention (ductal) phenomenon over a mucocele.  Epithelial lining (high-power view) Continuous lining 2-4 cells thick, composed of stratified cuboidal → low columnar / pseudostratified duct-type epithelium. Nuclei are basally located, cells uniform, no dysplasia. Occasional surface scalloping. Presence of an intact salivary duct epithelium is the hallmark of a salivary duct cyst; it distinguishes it from a mucous extravasation phenomenon, which lacks an epithelial lining.  Cyst wall / surrounding tissue Moderately dense fibrous stroma with dilated vessels and scattered chronic inflammatory cells; no granulation tissue reaction to spilled mucin; residual minor salivary gland tissue may be seen adjacent (not in every section). Chronic obstruction-related changes rather than a ruptured duct with inflammatory granulation tissue (which would be typical of an extravasation mucocele).  Contents Lumen largely empty on the section; at low power there are faint eosinophilic “smears” adhering to the lining, consistent with mucin that has been processed out. Retained saliva/mucin further supports a duct-obstructive (retention) process.  Taken together, the unilocular cystic space, the intact duct-type epithelial lining, and absence of a surrounding mucin-laden granulation tissue reaction are classic for a salivary duct cyst (mucous-retention cyst), an inflammatory–obstructive disorder of minor (or major) salivary glands. |
| 12.16 | Diagnosis: Salivary Duct Cyst with Oncocytic Metaplasia; Key Microscopic Features: Luminal surface is undulating, surrounded by chronic inflammation, and the lining is composed of oncocytes with a lymphoplasmacytic infiltrate. | Based on the provided hematoxylin and eosin (H&E) stained histological images at low and high magnification, the most likely histopathological diagnosis is:  Chronic Sialadenitis  Key Microscopic Features Supporting This Diagnosis:  1. Low Magnification (First Image):  Dilated salivary ducts with inspissated (thickened) eosinophilic material (mucus), indicating ductal obstruction.  Chronic inflammatory infiltrate (lymphocytes and plasma cells) seen predominantly around ducts and within the glandular parenchyma.  Acinar atrophy: Loss of normal salivary acini architecture; replaced by fibrous tissue and inflammatory cells.  Fibrosis: Increased pink-staining collagenous stroma interspersed with inflammatory cells — a hallmark of chronicity.  2. High Magnification (Second Image):  Dense lymphocytic infiltrate: This is prominent beneath the ductal epithelium and often arranged in a periductal or focal pattern.  Ductal epithelial hyperplasia or metaplasia: Reactive changes of the duct lining due to persistent inflammation or obstruction.  Occasional germinal center formation may be noted in some cases (though not clearly seen in this image), further supporting a chronic immune response.  Conclusion:  These features collectively indicate chronic sialadenitis, a long-standing inflammation of the salivary glands, typically associated with ductal obstruction (e.g., due to sialolithiasis or autoimmune disorders like Sjögren’s syndrome). The combination of duct dilation, acinar loss, periductal lymphocytic infiltration, and fibrosis is diagnostic. | Histopathological diagnosis (single best answer)  Necrotising sialometaplasia  Key microscopic features supporting this diagnosis  Feature visible on the slides Diagnostic significance  Large, irregular pale areas of coagulative necrosis occupying the centre of salivary lobules while the overall lobular outline is still recognisable (low-power image) Characteristic “infarct-like” necrosis that destroys acini but keeps the basic lobular architecture―a hallmark of necrotising sialometaplasia (NSM)  Striking squamous / pseudo-epidermoid metaplasia of duct and residual acinar epithelium forming bland epithelial islands and ribbons (both images) Ductal squamous metaplasia is the origin of the name “sialo-metaplasia” and can mimic carcinoma; nuclei are small, uniform, and lack dysplasia, favouring NSM  Mixed inflammatory infiltrate (neutrophils early, dense lymphocytes/plasma cells later) surrounding and infiltrating the metaplastic epithelium (high-power image) Reflects the reactive, self-limiting inflammatory nature of the lesion  Preservation of the overlying surface epithelium with possible pseudo-epitheliomatous hyperplasia (seen as the intact, slightly thickened mucosal layer in the low-power field) Overlying mucosa often shows reactive hyperplasia but remains intact, helping separate NSM from ulcerated malignant tumours  Ghost outlines / shadow acini and pooling of eosinophilic necrotic debris in the lumen (low-power image) Classic for ischaemic infarction of minor salivary gland lobules in NSM  Absence of cytologic atypia, mitoses, or infiltrative growth Confirms a benign, self-healing process rather than mucoepidermoid or squamous carcinoma  Together these findings – infarct-type lobular necrosis, squamous metaplasia of ducts/acini, bland cytology, inflammatory response, and maintained lobular architecture – are diagnostic of necrotising sialometaplasia, a self-limiting inflammatory disorder of minor salivary glands classically seen on the palate. |
| 12.16b | Diagnosis: Salivary Duct Cyst with Epithelial Plaques; Key Microscopic Features: Epithelial plaques transition into simple ductal lining epithelium. | Based on the hematoxylin and eosin (H&E) stained histological images you provided — both low and higher magnification views — the most likely histopathological diagnosis is:  Mucocele (Mucus Extravasation Phenomenon)  Key Microscopic Features Supporting This Diagnosis:  Mucin-filled Cavity:  There is a clear cyst-like space devoid of an epithelial lining (non-epithelialized), which contains mucin and inflammatory debris. This is a hallmark feature of mucus extravasation phenomenon, distinguishing it from mucus retention cysts (which are epithelial-lined).  Granulation Tissue Wall:  The surrounding wall of the cavity is composed of granulation tissue (proliferating capillaries, fibroblasts, and inflammatory cells), which is a reactive response to mucin spillage into the surrounding connective tissue.  Foamy Macrophages / Mucinophages:  In the higher magnification image, foamy histiocytes (macrophages containing mucin) can be seen, reflecting phagocytosis of extravasated mucin.  Chronic Inflammatory Infiltrate:  Scattered lymphocytes and plasma cells are seen in the connective tissue stroma, consistent with a chronic inflammatory response.  Overlying Surface Epithelium:  The mucosa overlying the lesion appears intact and not involved, which supports the diagnosis of an extravasation-type mucocele originating from minor salivary gland ducts.  Additional Notes:  These lesions are commonly found on the lower lip and are typically secondary to trauma or obstruction of minor salivary gland ducts.  Their non-epithelial-lined nature differentiates them from true cysts.  Let me know if you’d like a diagnostic diagram or comparison with other similar lesions such as ranulas, mucus retention cysts, or sialadenitis. | Histopathological diagnosis  Salivary duct cyst  (-- also called a mucous-retention cyst / true salivary duct cyst)  Microscopic features in the provided H-E sections that support this diagnosis  Observed feature Diagnostic significance  Well-defined cystic lumen occupying the centre of the field, partly filled with lightly eosinophilic fluid and scattered erythrocytes Indicates ductal dilatation rather than an extravasation pool (which lacks an epithelial lining)  Continuous epithelial lining, 2 – 4 cell layers thick, composed of cuboidal-to-low-columnar duct-type cells Confirms a true cyst derived from an excretory duct  Focal mucous/clear “goblet-like” cells and occasional oncocytic change within the lining Typical metaplastic changes in chronically obstructed salivary ducts  Smooth, gently papillary/undulating epithelial contour without dysplasia or squamous metaplasia Helps exclude necrotising sialometaplasia or neoplastic papilloma  Fibro-collagenous cyst wall with mild chronic inflammatory infiltrate and congested vessels Reflects a long-standing inflammatory/obstructive process; no tumour stroma present  Absence of lamellated calcified material (sialolith) or exuberant granulation tissue Makes pure sialolithiasis or a mucocele unlikely  Together, these features are classic for a salivary duct cyst caused by chronic obstruction/retention of saliva, one of the non-neoplastic inflammatory disorders of the salivary glands. |
| 12.17 | Diagnosis: Salivary Duct Cysts; Key Microscopic Features: The lining consists of a double layer of luminal columnar and cuboidal cells, typical for normal duct lining, and pseudostratified columnar ciliated epithelium with mucous. | Histopathological Diagnosis:  Chronic Sclerosing Sialadenitis (Küttner Tumor)  Justification Based on Microscopic Features:  Both provided hematoxylin and eosin (H&E) stained images show characteristic features of chronic sclerosing sialadenitis, a chronic inflammatory condition of the salivary glands, especially the submandibular gland.  Key Microscopic Features Observed:  Image 1 (Higher Magnification):  Dense lymphoplasmacytic infiltrate:  Numerous lymphocytes and plasma cells are infiltrating the glandular tissue, with a prominent periductal and lobular distribution.  Atrophic salivary ducts and acini:  Ducts appear distorted, and the acini are reduced in number and size, indicating progressive glandular atrophy.  Ductal hyperplasia and squamous metaplasia:  The ducts show signs of reactive hyperplasia, with some appearing compressed or undergoing squamous metaplasia—a typical response to chronic injury.  Fibrosis:  There is interstitial fibrosis separating glandular structures, a hallmark of chronicity.  Image 2 (Lower Magnification):  Preserved but reactive ductal epithelium:  The lining epithelium is intact but appears reactive (tall, columnar, with mild atypia), indicating chronic irritation.  Prominent fibrosis:  Thick collagen bundles are evident throughout the stroma, separating the lobules and replacing acinar tissue.  Chronic inflammatory infiltrate:  Again, a diffuse lymphoplasmacytic infiltrate is noted, consistent with long-standing inflammation. | Histopathological diagnosis (single best answer)  Salivary duct cyst (“mucous-retention cyst”)  Key microscopic features supporting this diagnosis  Feature seen on the photomicrographs Diagnostic significance  Well-circumscribed, greatly dilated ductal/luminal spaces with smooth internal contours (empty on the section) Indicates a true cyst rather than a mucous extravasation phenomenon, which lacks an epithelial lining  Cyst lined by intact duct-type epithelium that varies from thin cuboidal/columnar to pseudo-stratified columnar and focally multilayered Characteristic lining of a retention/duct cyst; absence of extensive squamous or oncocytic metaplasia argues against other lesions  Papillary/undulating epithelial infoldings projecting into the lumen (high-power view) Common reactive hyperplasia in chronically obstructed salivary ducts  Fibro-collagenous cyst wall containing scattered lymphocytes and plasma cells Mild chronic sialadenitis expected secondary to long-standing duct obstruction  No pools of extravasated mucin in the surrounding stroma, and no necrotic acinar debris Helps rule out mucous extravasation mucocele and necrotizing sialometaplasia  Together, the presence of a true epithelial lining, the pattern of ductal dilation with papillary infolding, and the accompanying low-grade chronic inflammation place the lesion squarely in the spectrum of inflammatory/obstructive salivary gland disorders and are diagnostic of a salivary duct (mucous-retention) cyst. |
| 12.21 | Diagnosis: Sialolith; Key Microscopic Features: Sialolith within an ectatic duct with squamous metaplasia and partial rupture, surrounded by inflammation. The duct epithelium is lined by squamous and mucous cells. | Histopathological Diagnosis:  Chronic sialadenitis with sialolithiasis (sialolith-associated chronic sialadenitis)  Key Microscopic Features Supporting the Diagnosis:  Image 1 (Low Power View):  Central Calcified Mass (Sialolith):  The central structure with concentric lamellated appearance and eosinophilic content is characteristic of a sialolith (salivary duct stone).  Irregular contours and lamellar calcifications confirm it is not a normal glandular component.  Dilated Salivary Duct:  The sialolith is located within a dilated duct, lined by ductal epithelium, which shows signs of pressure atrophy.  Chronic Inflammatory Infiltrate:  The periductal region and adjacent stroma show dense chronic inflammatory cell infiltrates, predominantly lymphocytes and plasma cells, typical of chronic sialadenitis.  Fibrosis:  The surrounding glandular architecture is distorted by fibrosis, a hallmark of chronic inflammation.  Image 2 (High Power View):  Ductal Epithelium:  Shows reactive changes including hyperplasia and metaplasia, often seen in chronic ductal obstruction.  Mixed Inflammatory Infiltrate:  Dense infiltration of lymphocytes and plasma cells in the connective tissue around the duct supports a chronic inflammatory process.  Acinar Atrophy:  Though not prominently visible in this cropped high-power view, it is typically present in such lesions due to pressure and chronic obstruction. | Histopathological diagnosis  Sialolithiasis (salivary duct stone) with secondary obstructive sialadenitis  Key microscopic features supporting the diagnosis  Feature What is seen in the images Diagnostic significance  Calcified intraductal mass (sialolith) A large, centrally placed, intensely basophilic, partly lamin-ated body occupying the duct lumen (heart-shaped in the low-power view) Pathognomonic for sialolithiasis – the stone forms by concentric deposition of calcium salts around an organic nidus  Dilated salivary duct The lumen is markedly widened around the stone Indicates chronic obstruction by the calculus  Squamous/metaplastic thickening of the ductal epithelium On high power, the duct lining is converted to a stratified squamous epithelium with prominent, uniform nuclei and no keratinisation A common epithelial reaction to chronic irritation by the stone  Periductal chronic inflammatory infiltrate Dense lymphocytes and plasma cells encircle the duct; fibrous tissue is increased Reflects long-standing obstructive sialadenitis secondary to the sialolith  Acinar atrophy / stromal fibrosis (low-power field) Surrounding glandular parenchyma shows loss of normal acini and replacement by fibrous stroma Further evidence of chronic obstructive injury  Together, the calcified stone within a dilated, metaplastically thickened duct and the surrounding chronic inflammatory and fibrotic changes are classic for sialolithiasis of a major or minor salivary gland. |
| 12.22 | Diagnosis: Sialolith; Key Microscopic Features: Sialolith within an ectatic duct with squamous metaplasia opening into the oral cavity, with lamellar and globular concretions. | Based on the provided hematoxylin and eosin (H&E) stained images of a lesion from the inflammatory salivary gland disorders category, here is the histopathological analysis:  🧠 Most Likely Histopathological Diagnosis:  Chronic sialolithiasis with associated chronic sialadenitis  🔬 Key Microscopic Features Supporting the Diagnosis:  🖼️ Low-power image (Image 1):  Central concentric lamellated structure: This is consistent with a sialolith (salivary stone), showing characteristic concentric layering—indicative of calcified deposits forming in layers over time.  Ductal dilatation: Suggests chronic obstruction caused by the sialolith.  Chronic inflammation: Dense lymphoplasmacytic infiltrates surrounding the duct and stone, pointing to long-standing inflammation.  🔬 High-power image (Image 2):  Acinar atrophy: Normal glandular tissue is replaced by fibrous tissue, suggesting chronicity.  Periductal and interstitial fibrosis: Indicates chronic inflammatory injury.  Mixed inflammatory infiltrate: Predominantly lymphocytes and plasma cells with occasional neutrophils—typical in chronic sialadenitis.  Vascular congestion and mild hemorrhage: Often seen in chronic inflammatory settings. | Histopathological diagnosis (single best):  Sialolithiasis with secondary chronic obstructive sialadenitis  Key microscopic features that support this diagnosis  Field Observation Significance  Low-power architecture A markedly dilated salivary duct is filled by a large, round to oval mass that shows concentric, onion-skin lamellation with a central core and numerous processing cracks. Classic appearance of a salivary stone (sialolith) produced by successive deposition of organic material and calcium salts.  Nature of intraductal material The lamellated mass is intensely eosinophilic to basophilic and fractured; the concentric layers have alternating light and dark lines. Indicates mineralised, inspissated secretions typical of a sialolith; radial and concentric clefts/cracks are common artefacts of a calcified concretion.  Ductal epithelium The lining duct epithelium is thinned, flattened or focally squamoid from pressure; in places it is ulcerated or hyperplastic. Obstructive pressure changes from the stone.  Periductal tissue / gland parenchyma • Prominent chronic inflammatory cell infiltrate (mainly lymphocytes and plasma cells)  • Fibrosis and sclerosis of perilobular stroma  • Atrophy of adjacent serous and mucous acini Features of chronic sialadenitis that develops secondary to long-standing duct obstruction by the sialolith.  Remaining acini and ducts Dilated intercalated ducts containing inspissated mucin; some acini show secretory exhaustion. Further evidence of obstructive change.  Together, the characteristic lamellated intraductal concretion plus the surrounding chronic inflammatory and fibrotic response leave sialolithiasis as the single most appropriate histopathological diagnosis. |
| 12.25 | Diagnosis: Cheilitis Glandularis; Key Microscopic Features: Tortuous and dilated ducts and surrounding glands show marked acute and chronic sialadenitis, with luminal mucin and suppuration, as well as ciliated and mucous cell prosoplasia of ducts and surrounding lymphoplasmacytic infiltrate. | Based on the two provided hematoxylin and eosin (H&E) stained images at different magnifications, here is the histopathological analysis:  Single Most Likely Histopathological Diagnosis:  Chronic Sclerosing Sialadenitis (Küttner Tumor)  Key Microscopic Features Supporting the Diagnosis:  Low Magnification Image (First Image):  Lobular architecture distortion with replacement of normal salivary gland tissue by dense fibrous tissue.  Periductal and interlobular fibrosis, extending into the parenchyma in a storiform or irregular pattern.  Dilated ducts with inspissated eosinophilic material ("mucin plugs"), possibly showing ductal squamous metaplasia.  Patchy chronic inflammatory infiltrates predominantly composed of lymphocytes, often forming follicles with germinal centers.  High Magnification Image (Second Image):  Dense lymphoplasmacytic infiltrate, notably around ducts and acini.  Acinar atrophy with preserved ductal elements.  Fibrosis between and around lobules, compressing and distorting glandular structures.  Occasional germinal center formation indicating chronic immune stimulation.  Diagnostic Justification:  The combination of dense lymphoplasmacytic inflammation, progressive fibrosis, ductal changes, and acinar atrophy is hallmark for Chronic Sclerosing Sialadenitis, often associated with IgG4-related disease, especially in submandibular glands.  This lesion is commonly mistaken for neoplasm clinically due to firm swelling, but histology clearly points to an inflammatory fibrosing process. | Histopathological diagnosis — Acute (suppurative) bacterial sialadenitis  Key microscopic features supporting this diagnosis  Field Observations Significance  Low-power overview (first image) * • Preserved lobular outline of a salivary gland  * • Irregular, dilated duct-like spaces containing pale eosinophilic debris (“pus lakes”) that branch through the parenchyma  * • Surrounding parenchyma shows diffuse violet–pink haze indicating tissue necrosis and heavy inflammatory cell density Typical early lobular involvement with destruction of acini and intraductal accumulation of purulent exudate seen in acute bacterial infection  High-power view (second image) * • Intra-ductal/-acinar micro-abscesses: lumina packed with numerous polymorphonuclear neutrophils, fibrin and erythrocytes  * • Intact but reactive duct lining epithelium (cuboidal-columnar) showing focal vacuolar injury, no dysplasia or squamous metaplasia  * • Adjacent stroma with oedema, capillary congestion and scattered neutrophils Neutrophil-rich exudate and micro-abscess formation are hallmarks of acute suppurative inflammation; the absence of marked squamous metaplasia favours acute sialadenitis over necrotising sialometaplasia  Additional cues * • No granulomas, storiform fibrosis or IgG4-like plasma-cell collections (rules out chronic sclerosing/auto-immune forms)  * • No ductal squamous islands or pseudo-epitheliomatous hyperplasia (rules out necrotising sialometaplasia) Helps narrow the lesion to the acute infectious end of the inflammatory spectrum  Together, the combination of lobular destruction with neutrophil-filled ducts/acinus and parenchymal necrosis firmly points to acute (suppurative) bacterial sialadenitis rather than the other inflammatory salivary gland disorders. |
| 12.27 | Diagnosis: Necrotizing sialometaplasia; Key Microscopic Features: Extensive lobular necrosis with some confluent areas; remnants of infarcted acini with necrosis and squamous metaplasia. | Based on the hematoxylin-and-eosin stained histological images you provided at low, intermediate, and high magnification, the single most likely histopathological diagnosis is:  Sialadenitis (Chronic Sclerosing Sialadenitis / Kuttner Tumor)  Key Microscopic Features Justifying the Diagnosis:  Low Magnification (First Image):  Lobular architecture distortion: The salivary gland parenchyma is disrupted.  Dense inflammatory infiltrate: Particularly periductal and perivascular.  Fibrosis: Prominent stromal fibrosis replacing glandular tissue.  Intermediate Magnification (Second Image):  Acinar atrophy: Glandular acini are largely replaced by fibrosis and inflammatory cells.  Ductal dilation: Some ducts appear dilated with surrounding lymphoplasmacytic infiltrates.  Chronic inflammation: Lymphocytes, plasma cells, and possibly some eosinophils.  High Magnification (Third Image):  Periductal fibrosis and inflammation: Lymphoplasmacytic infiltrates encasing ducts.  Oncocytic ductal changes: Hyperplastic or reactive ductal epithelium.  Occasional germinal centers: Indicative of a chronic immune response.  Differential Diagnosis Considerations:  Sjögren syndrome: Might show similar features but typically involves lymphoepithelial lesions and germinal center formation in minor salivary glands.  Mucocele or obstruction-related changes: Usually show mucin pools and granulation tissue, less fibrosis.  Neoplasm (e.g., mucoepidermoid carcinoma): Would show neoplastic cellular features, which are absent here. | Histopathological diagnosis  Necrotizing sialometaplasia (NS) – an ischaemic-type inflammatory disorder of minor salivary glands, most often seen on the palate.  Key microscopic features in the provided H-E sections  Magnification What is seen How it supports NS  Low-power (whole section) • Well-circumscribed, lobulated lesion beneath intact/only slightly eroded surface epithelium  • Architecture of the salivary gland lobules is preserved even though the acini look pale and “ghost-like” Preservation of lobular outline with coagulative necrosis of acini is the classic low-power appearance of necrotizing sialometaplasia.  Medium-power • Numerous round/oval, empty-looking spaces – coagulative necrosis of mucous acini with retained outlines (“ghost” acini)  • Extravasated mucus pools and lakes in the necrotic area  • Mixed chronic inflammatory cell infiltrate and early granulation tissue replacing some lobules Infarction of mucous acini with mucus extrusion and secondary inflammation is characteristic of NS.  High-power • Ducts and residual acini show squamous metaplasia, forming irregular cords, nests and trabeculae of bland squamoid epithelium in the infarcted stroma  • Cells have uniform nuclei, inconspicuous nucleoli, and no significant atypia or mitotic activity  • Scattered viable mucous cells may still be present Squamous metaplasia of salivary ducts/acini without cytologic malignancy – together with the necrosis – is the diagnostic hallmark, and can mimic squamous cell or mucoepidermoid carcinoma if the overall context is not recognised.  Putting the features together  Lobular coagulative necrosis of minor salivary glands  Mucin extravasation and accompanying inflammatory / reparative response  Squamous metaplasia of the surviving duct–acinar epithelium, but with bland cytology  These three findings in combination are pathognomonic for necrotizing sialometaplasia, an inflammatory/ischemic lesion rather than a true neoplasm. |
| 12.28 | Diagnosis: Necrotizing sialometaplasia; Key Microscopic Features: Diffuse necrosis with squamous metaplasia and inflammation, and infarction of acini with remnants of the basement membrane and necrosis. | Based on the provided hematoxylin-and-eosin (H&E) stained images at different magnifications, this salivary gland lesion is most consistent with:  Histopathological Diagnosis:  Chronic sialadenitis  Key Microscopic Features Supporting the Diagnosis:  Low Magnification Image (First Image):  Lobular architecture distortion: The normal salivary gland architecture is disrupted.  Acinar atrophy: A significant loss of serous acini is evident.  Ductal dilation: Some remaining ducts appear dilated, often seen in chronic obstruction.  Fibrosis: Replacement of glandular tissue with fibrous stroma is present, particularly in the lower half.  Lymphocytic infiltrate: Prominent chronic inflammatory infiltrate, predominantly lymphocytes, within and around the glandular structures.  High Magnification Image (Second Image):  Oncocytic (metaplastic) ductal epithelium: The ductal epithelium shows oncocytic change (cells with abundant eosinophilic, granular cytoplasm), typical of chronic injury.  Chronic inflammation: Scattered lymphocytes and plasma cells are evident infiltrating the parenchyma.  Acinar destruction: Many acini are replaced by fibrosis or inflammatory cells.  Vascular changes: Some blood vessels may appear congested, a common finding in chronic inflammation.  These features are classic for chronic sialadenitis, an inflammatory disorder of the salivary gland usually associated with recurrent or persistent ductal obstruction, often secondary to sialolithiasis or ductal stenosis, and leading to progressive glandular damage over time.  Let me know if you'd like a comparison with other salivary gland disorders (e.g., Sjögren’s syndrome or benign lymphoepithelial lesion) or a diagnostic table for differential diagnosis. | Histopathological diagnosis  ➜ Necrotising sialometaplasia  Key microscopic features that point to this diagnosis  Field What is seen Why it matters  Low-power architecture A wedge- or lobule-shaped zone of pale, eosinophilic tissue with the overall lobular outline of the salivary gland still recognisable. Classical “lobular infarction” pattern—architecture is preserved even though the parenchyma is dead, a hallmark of necrotising sialometaplasia.  Parenchymal change (high-power) Seromucous acini appear as ghost outlines: cytoplasm is pale, nuclei have disappeared, but the acinar borders are intact (coagulative necrosis). Confirms ischaemic-type necrosis rather than chronic atrophy or a neoplastic process.  Duct/epithelial reaction Islands and ducts with squamous or pseudo-epidermoid metaplasia (seen as thick eosinophilic epithelial cylinders in the low-power field). Squamous metaplasia of ducts and residual acini is characteristic and can mimic carcinoma; recognising it as metaplastic rather than malignant is key.  Inflammatory response A mixed acute inflammatory infiltrate (numerous neutrophils around the necrotic acini) with focal haemorrhage and vascular congestion. Explains the ulcer-like clinical presentation and situates the lesion in the inflammatory—not neoplastic—category.  Mucin/RBC extravasation Pools of spilled mucin and extravasated red cells between lobules. Frequently produced when necrotic acini rupture; another supportive clue to necrotising sialometaplasia.  Put together, the preserved lobular architecture with ghost acini, squamous metaplasia of ducts, acute inflammation and haemorrhage are diagnostic for necrotising sialometaplasia, an ischaemic inflammatory disorder that can clinically and histologically mimic a malignant salivary tumour. |
| 12.28b | Diagnosis: Necrotizing sialometaplasia; Key Microscopic Features: Pseudosarcomatoid appearance of ducts due to squamous metaplasia with marked surrounding inflammation. | Based on the hematoxylin-and-eosin (H&E) stained histopathological images provided, which belong to the group of inflammatory salivary gland disorders, the most likely diagnosis is:  Chronic Sclerosing Sialadenitis (Küttner Tumor)  Key Microscopic Features Supporting the Diagnosis:  Low Magnification (First Image):  Dense chronic inflammatory infiltrate: Predominantly lymphocytes with occasional plasma cells, diffusely infiltrating the salivary gland parenchyma.  Fibrosis and sclerosis: Extensive stromal fibrosis surrounding and replacing normal acinar structures, with distortion of glandular architecture.  Atrophic acini: Marked reduction in the number and size of acini, often replaced by fibrosis.  High Magnification (Second Image):  Periductal and intraductal lymphoplasmacytic infiltrates: Lymphocytes closely surrounding ductal epithelium, sometimes infiltrating ductal walls.  Ductal changes: Dilated ducts, some with eosinophilic contents, surrounded by lymphoid aggregates.  Oncocytic metaplasia: Ductal epithelial cells show eosinophilic granular cytoplasm.  Possible lymphoid follicle formation: Suggestive of a chronic immune-mediated process.  Differential Diagnosis Consideration:  While other inflammatory conditions like Sjogren’s syndrome and chronic sialadenitis can mimic some features, the dense fibrosis, prominent periductal infiltrate, and ductal alterations strongly support Küttner tumor, which is a subtype of chronic sclerosing sialadenitis, often associated with IgG4-related disease.  If needed, confirmation with IgG4 immunostaining and clinical correlation (e.g., firm submandibular gland swelling) may further support this diagnosis. Let me know if you’d like a report format or more details on the differential features. | Histopathological diagnosis  → Necrotizing sialometaplasia (NSM)  Key microscopic features that support this diagnosis  Feature seen in the images Why it points to necrotizing sialometaplasia  Preserved lobular outline on low power (first image) – the gland still looks like a series of coagulated lobules rather than a destructive invasive mass NSM is an infarct-type process; the basic salivary-gland architecture remains intact even though the tissue is necrotic  Widespread squamous (epidermoid) metaplasia of ducts / acini – round to elongated epithelial islands with intercellular bridges and occasional keratin pearls, many retaining a central lumen (second image) Characteristic conversion of glandular epithelium into bland squamous cells after ischemic injury; it can mimic mucoepidermoid or squamous carcinoma but the nuclei remain uniform and mitoses are scant  Coagulative necrosis of acinar cells – eosinophilic, ghost-like acini with loss of nuclear detail intermixed with viable metaplastic epithelium Infarction is the initiating event in NSM  Extravasated mucin and pools of inflammatory debris – pale basophilic material and scattered neutrophils/RBCs around the metaplastic islands Mucin leakage from ruptured acini is typical; it attracts an acute inflammatory response  Mixed chronic-acute inflammatory infiltrate and reactive granulation tissue in the surrounding stroma NSM is self-healing; granulation tissue begins to repair the infarcted lobule  Minimal cytologic atypia, no dysplastic basal layer “tailing,” and no destructive stromal invasion Helps distinguish NSM from true malignancies that it clinically and histologically mimics  Taken together, the combination of infarct-type necrosis, lobular preservation, extensive but cytologically bland squamous metaplasia with residual lumina, mucin extravasation, and a mixed inflammatory background is classic for necrotizing sialometaplasia, the prototypical inflammatory/ischemic disorder of minor salivary glands in the palate. |
| 12.29 | Diagnosis: Subacute necrotizing sialadenitis; Key Microscopic Features: Preservation of lobular architecture with marked interstitial inflammation and acinar atrophy, residual acini with fibrosis, and acute inflammation. | Based on the provided hematoxylin-and-eosin (H&E) stained histological images at different magnifications, the single most likely histopathological diagnosis is:  Chronic Sclerosing Sialadenitis (Küttner Tumor)  Key Microscopic Features Supporting the Diagnosis:  Low-Power View (1st Image):  Lobular architecture distortion of the salivary gland.  Extensive fibrosis and replacement of glandular parenchyma.  Ill-defined nodularity suggesting chronic inflammatory involvement.  Intermediate Magnification (2nd Image):  Dense lymphoplasmacytic infiltrate surrounding and replacing the glandular tissue.  Residual ductal and acinar structures are seen amidst the inflammation.  Periductal fibrosis and obliterative phlebitis may be suggested (though hard to confirm without higher magnification of vessels).  Numerous eosinophilic, proteinaceous deposits in ducts (possible ductal inspissation).  High-Power View (3rd Image):  Prominent plasma cell infiltrate, including possible IgG4-positive plasma cells (immunostaining would confirm this).  Scattered eosinophils.  Evidence of acinar atrophy and fibrotic stroma interspersed with inflammatory cells.  Ductal dilatation and squamous metaplasia may also be present.  Conclusion:  These findings are characteristic of Chronic Sclerosing Sialadenitis (Küttner Tumor), a chronic inflammatory disorder of the salivary glands, most often affecting the submandibular gland. It is often associated with IgG4-related disease, hence, the abundance of plasma cells and fibrosis is key. If IgG4 staining were available, it would help confirm the diagnosis further.  Let me know if you'd like a side-by-side comparison with other inflammatory salivary gland disorders or need a diagnostic summary. | Histopathological diagnosis (single best):  Necrotizing sialometaplasia  Key microscopic features supporting this diagnosis  Field What is seen in the submitted sections Why it points to necrotizing sialometaplasia  Overall (low-power) Lobular architecture of the salivary gland is still recognisable, but large wedge-shaped to irregular areas are pallid and eosinophilic, lacking normal acinar detail (“ghost-lobules”) Characteristic coagulative ischaemic necrosis of whole glandular lobules while the basic outline of the gland is retained  Parenchyma • Most mucous acini are necrotic – they appear as pale, vacuolated circular profiles with lost nuclei  • Residual duct/acinus profiles show squamoid epithelium forming islands and strands Combination of acinar necrosis plus squamous metaplasia of ducts and acini is the histologic hallmark; the metaplastic epithelium can mimic carcinoma but is cytologically bland  Stroma & lumen contents • Pools of extravasated mucin and spilled secretions surround necrotic acini  • Patchy haemorrhage and numerous extravasated red cells Mucin spillage and haemorrhage are typical consequences of sudden acinar infarction  Inflammatory infiltrate Dense, mixed inflammatory cell population – early neutrophils admixed with many lymphocytes, plasma cells and macrophages that permeate and outline necrotic lobules Reflects the acute-on-chronic reparative reaction that follows infarction in necrotizing sialometaplasia  Cytologic atypia None – metaplastic squamoid cells are uniform, with small regular nuclei and no abnormal mitotic activity Helps distinguish the lesion from mucoepidermoid carcinoma or squamous-cell carcinoma despite the alarming architecture  Taken together, preservation of lobular outline, coagulative necrosis of mucous acini, squamous metaplasia of duct/acinar epithelium, mucin spillage, haemorrhage, and a brisk mixed inflammatory response form the classic constellation diagnostic of necrotizing sialometaplasia. |
| 12.31 | Diagnosis: Autoimmune sialadenitis in Sjögren syndrome; Key Microscopic Features: Preserved lobular structure with significant lymphocytic infiltrate surrounding ducts, with no obstructive changes. | Based on the histopathological features observed across the three hematoxylin and eosin (H&E) stained images at increasing magnifications, the single most likely diagnosis is:  Chronic Sclerosing Sialadenitis (Küttner Tumor)  Key Microscopic Features Supporting the Diagnosis:  Preserved Lobular Architecture with Lymphoid Infiltrate:  Seen clearly in the low-power image (first image), the gland maintains a lobular structure but shows dense lymphoplasmacytic infiltrates replacing large areas of the parenchyma.  Numerous lymphoid follicles with germinal centers are identifiable, suggesting chronic inflammation and immune response.  Periductal and Perivascular Lymphocytic Aggregates:  Mid- and high-power views (second and third images) reveal periductal lymphoid infiltrates and perivascular cuffing, classic features of chronic inflammatory sialadenitis.  Acinar Atrophy and Ductal Changes:  The higher magnification (third image) shows marked acinar atrophy, with residual ductal structures surrounded by inflammatory cells.  Ducts exhibit epithelial hyperplasia or oncocytic metaplasia, often seen in sclerosing sialadenitis.  Fibrosis (Sclerosis):  While not overtly dense in these images, interstitial fibrosis is beginning to be seen in between ducts and acini, a key feature in the progression of Küttner tumor.  Additional Notes:  Chronic sclerosing sialadenitis primarily affects the submandibular gland and is often confused clinically with neoplasms due to its firm consistency.  It is now considered part of the IgG4-related disease spectrum, although confirmation would require immunohistochemistry (IgG4-positive plasma cells).  If you require a differential diagnosis or further discussion on how to distinguish this from other inflammatory salivary gland disorders (like Sjögren’s syndrome or obstructive sialadenitis), I’d be happy to help. | Histopathological diagnosis (single best answer)  Benign lympho-epithelial lesion (lympho-epithelial sialadenitis, the salivary-gland manifestation of Sjögren syndrome).  Key microscopic features that point to this diagnosis  Feature Appearance in your sections Diagnostic significance  1. Dense, diffuse lymphocytic infiltrate replacing the gland Nearly the whole parenchyma is packed with small, dark-blue lymphocytes; very little normal serous acini remain (all three magnifications) Characteristic of an autoimmune chronic sialadenitis rather than an acute/pyogenic one  2. Numerous reactive germinal centres Pale, round follicular centres bordered by a mantle of darker lymphocytes are scattered throughout the lobule (best seen on the lowest-power image) Indicates a long-standing, antigen-driven B-cell response typical of LESA/Sjögren  3. Acinar atrophy with preservation/ proliferation of ducts Most acini are lost; residual ducts are prominent and sometimes dilated or elongated Acinar drop-out is a hallmark of benign lympho-epithelial lesions  4. Epimyo-epithelial islands / lympho-epithelial lesions On medium–high power, ductal epithelial cells show squamous or oncocytic metaplasia and are permeated and surrounded by lymphocytes, forming solid epithelial islands (third image) Virtually pathognomonic for benign lympho-epithelial lesion  5. Scant interstitial fibrosis without storiform or keloid-like pattern Pink collagen bundles are present but not dominant; no dense sclerosis (seen focally around lobule) Helps exclude fibrosing entities such as IgG4-related chronic sclerosing sialadenitis (Küttner tumour)  Taken together—diffuse lymphoid infiltrate with germinal-centre formation, near-total acinar loss, and the presence of lymphocyte-infiltrated ductal/epithelial islands—the picture is classic for a benign lympho-epithelial lesion (lympho-epithelial sialadenitis/Sjögren-type chronic sialadenitis). |
| 12.32 | Diagnosis: Sjögren syndrome and lymphoma of the palatal mucous glands; Key Microscopic Features: Complete effacement of lobular architecture by atypical lymphoid infiltrate, lymphoepithelial lesions with pale cells surrounding epithelial islands. | Based on the provided hematoxylin and eosin (H&E) stained histological images at low, intermediate, and high magnifications, the single most likely histopathological diagnosis is:  Chronic Sclerosing Sialadenitis (Küttner Tumor)  Key Microscopic Features Supporting the Diagnosis:  🔬 Low Power (Image 1):  Lobular architecture distortion of the salivary gland.  A dense lymphoplasmacytic infiltrate nearly replacing normal acinar structures.  Noticeable fibrosis, particularly extending into and around salivary ducts and lobules.  🔬 Intermediate Power (Image 2):  Lymphoid follicle formation is evident, suggestive of chronic inflammation.  The salivary gland parenchyma is replaced by fibrotic stroma.  Periductal and interlobular fibrosis is clearly apparent.  🔬 High Power (Image 3):  A dense lymphoplasmacytic infiltrate is observed.  Presence of acinar atrophy and ductal changes (sometimes showing dilatation or inspissated material).  Fibrosis appears hyalinized in some areas, a typical finding in this disorder.  No features of malignancy (e.g., no abnormal mitoses or cellular atypia).  Additional Diagnostic Context:  Chronic sclerosing sialadenitis, or Küttner tumor, is a benign inflammatory condition that most commonly affects the submandibular gland and mimics neoplasms clinically and sometimes radiographically. It’s often associated with IgG4-related disease, particularly when dense fibrosis, obliterative phlebitis, and elevated IgG4+ plasma cells are present (though confirmation would require immunohistochemistry). | Single most likely histopathological diagnosis  Benign lympho-epithelial lesion (BLEL) / lympho-epithelial sialadenitis – the salivary-gland manifestation that is classically seen in Sjögren syndrome.  Key microscopic features supporting this diagnosis  Magnification Feature Explanation  Low-power overview • Almost the entire glandular lobule is replaced by a solid, basophilic infiltrate while a thin rim of mucosa and adipose tissue remains.  • Overall lobular outline is still recognisable, without the destructive pattern of a true neoplasm. Consistent with a diffuse, non-neoplastic lymphoid process centred in a salivary gland.  Intermediate power • Dense, polymorphous lymphoid infiltrate forming germinal-centre-type aggregates.  • Marked loss of normal acini; only scarce ductal profiles remain. An autoimmune-type sialadenitis shows organised lymphoid tissue and acinar atrophy.  High power • Residual duct/striated-duct epithelium exhibits squamoid/oncocytic metaplasia and is infiltrated by lymphocytes, producing the classic epimyo-epithelial islands.  • Islands are outlined by basement-membrane-like pink material; individual epithelial cells have abundant eosinophilic cytoplasm but bland nuclei.  • Surrounding stroma packed with small lymphocytes and scattered plasma cells; no coagulative necrosis or neutrophil-rich suppuration. Epimyo-epithelial islands are the diagnostic hallmark of BLEL; their presence together with lymphoid tissue and acinar loss distinguishes the lesion from necrotising sialometaplasia, chronic obstructive sialadenitis, or malignancy.  Collectively, the diffuse lymphoplasmacytic infiltrate with germinal centres, profound acinar depletion, and the characteristic epithelial islands engulfed by lymphocytes are pathognomonic for a benign lympho-epithelial lesion / lympho-epithelial sialadenitis.  2/2 |
| 12.33 | Diagnosis: Sjögren syndrome and lymphoma of the parotid gland; Key Microscopic Features: Epithelial islands within lymphoid infiltrates, infiltrated by monocytoid B cells consistent with marginal zone lymphoma. | Based on the provided hematoxylin and eosin (H&E) stained images at low and high magnifications, the single most likely histopathological diagnosis is:  Chronic Sialadenitis (likely of autoimmune origin, e.g., Sjögren's syndrome)  Key Microscopic Features Supporting the Diagnosis:  1. Dense Lymphocytic Infiltrate  Seen prominently in both low and high magnification.  The salivary gland parenchyma is heavily infiltrated by small, darkly staining lymphocytes.  These infiltrates often form lymphoid aggregates or follicles with germinal centers, a hallmark of chronic autoimmune sialadenitis.  2. Acinar Atrophy  The normal salivary acini are largely replaced or destroyed by the inflammatory infiltrate.  Residual ductal structures may be visible, often dilated or distorted.  3. Fibrosis and Stromal Changes  Evidence of fibrosis and stromal expansion is seen in the low-power image, consistent with long-standing inflammation.  Some adipose tissue replacement may also be present.  4. Ductal Changes  Remaining ducts may appear dilated or undergo squamous metaplasia.  These ductal changes, alongside atrophy, further suggest a chronic process.  Differential Considerations:  Benign Lymphoepithelial Lesion (BLEL): If associated with a background of autoimmune disease like Sjögren’s syndrome, BLEL may be present as part of the chronic sialadenitis spectrum.  Low-grade MALT lymphoma: Needs to be ruled out via clonality studies or immunohistochemistry, especially in dense lymphoid infiltrates like these. | Histopathological diagnosis (single best answer)  Benign lympho-epithelial lesion (BLEL) / lympho-epithelial sialadenitis – the salivary-gland manifestation of Sjögren syndrome.  Key microscopic features that support the diagnosis  Feature How it appears in the provided sections Diagnostic significance  Dense, diffuse lymphoid infiltrate Almost the entire glandular parenchyma is replaced by sheets of small, dark-staining lymphocytes (second image, low & high power) Hallmark of lympho-epithelial sialadenitis  Well-formed lymphoid follicles with germinal centres Pale round/ovoid foci within the lymphoid sea (first image, centre–left) Indicates chronic autoimmune-type immune response characteristic of BLEL/Sjögren  Marked acinar atrophy with fatty replacement Normal serous/mucous acini are largely lost; islands of mature adipose tissue occupy their place (first image, far left) Chronic, progressive destruction of secretory units typical of BLEL  Epimyo-epithelial (lympho-epithelial) islands Nests/cords of squamoid ductal epithelial cells with eosinophilic cytoplasm embedded in lymphocytes (second image, right half) Signature feature giving the lesion its name  Periductal & interlobular fibrosis Pink, fibrous stroma separating lymphoid aggregates (first image, upper right) Seen in long-standing lesions  Relative preservation/enlargement of ductal elements Residual ducts appear dilated or metaplastic amidst lymphocytes (both magnifications) Reflects selective destruction of acini with ductal hyperplasia/metaplasia  The combination of acinar loss, replacement by a dense lymphoplasmacytic infiltrate containing germinal centres, and the presence of characteristic epimyo-epithelial islands is diagnostic for benign lympho-epithelial lesion rather than simple chronic sialadenitis or other inflammatory disorders of the salivary glands. |
| 12.35 | Diagnosis: IgG-related disease of submandibular gland; Key Microscopic Features: Acinar atrophy with diffuse plasma cell and lymphocyte infiltration, fibrosis around ducts, and germinal center formation. | Based on the provided hematoxylin-and-eosin stained histological images taken at different magnifications, the single most likely histopathological diagnosis is:  Chronic Sclerosing Sialadenitis (Küttner tumor)  Justification with Key Microscopic Features:  1. Lobular Architecture with Progressive Fibrosis (Image 1 & 2)  Low-power views show retained lobular architecture with dense fibrosis, predominantly in the periductal and interlobular regions.  There’s acinar atrophy and replacement of glandular parenchyma with fibrous tissue over time.  Lobules are separated by bands of collagenous tissue.  2. Chronic Inflammatory Infiltrate (Images 2, 3 & 4)  Moderate to dense infiltrate composed mainly of lymphocytes, along with plasma cells and scattered macrophages.  These infiltrates are distributed around ducts and within fibrous septa.  There is a possibility of lymphoid follicle formation in the higher magnification images.  3. Periductal and Perivascular Fibrosis (Image 3)  Concentric fibrosis around ducts (onion-skin pattern) is visible.  Characteristic of chronic sclerosing processes affecting major salivary glands.  4. Obliterative Phlebitis and Ductal Changes (Image 4)  Higher magnification reveals inflammatory cells within vessel walls, suggesting obliterative phlebitis, a feature often associated with IgG4-related disease spectrum.  Associated ductal alterations may include duct dilation, squamous metaplasia, or even ductal destruction in more advanced cases.  Additional Considerations:  This entity is now often considered part of the IgG4-related disease spectrum, especially when abundant IgG4-positive plasma cells are present, although immunostaining would be needed to confirm this.  Summary:  Diagnosis: Chronic Sclerosing Sialadenitis (Küttner tumor)  Key Features: Acinar atrophy, dense lymphoplasmacytic infiltrate, lobular fibrosis, ductal changes, and possible obliterative phlebitis. | Histopathological diagnosis  Chronic sclerosing sialadenitis (Küttner tumour) / IgG4-related sialadenitis of the major salivary gland  Key microscopic features that support the diagnosis  Image Salient findings Diagnostic relevance  Low-power overview (image 1) • Lobular outline of the gland is still recognizable but each lobule is replaced by densely sclerotic, pink fibrous tissue that surrounds islands of residual parenchyma.  • The fibrous septa create a nodular (“tumour-like”) architecture. Global picture of a sclerosing, tumour-like inflammatory mass typical of Küttner tumour.  Intermediate magnification (image 2) • Marked lymphoplasmacytic infiltrate with formation of reactive germinal centres.  • Acinar atrophy and scattered adipose replacement.  • Dilated residual ducts with inspissated secretions. The triad of acinar loss, heavy plasma-cell-rich infiltrate and fatty change is characteristic of IgG4-related sialadenitis.  High magnification (image 3) • A duct (or venule) is surrounded by concentric, “onion-skin” periductal fibrosis.  • Fibrosis shows a whorled / storiform pattern. Periductal onion-skin fibrosis is a hallmark of chronic sclerosing sialadenitis and IgG4-related disease.  High magnification (image 4) • Dense, storiform collagen packed with numerous plasma cells and small lymphocytes.  • Scattered eosinophils, bland spindle-shaped fibroblasts; occasional obliterated small vessel walls (obliterative phlebitis). Storiform fibrosis + plasma-cell-rich infiltrate ± obliterative phlebitis are defining histologic criteria for IgG4-related disease.  Integrating the findings  Lobular sclerosis with nodular architecture.  Dense lymphoplasmacytic (IgG4-rich) infiltrate forming germinal centres.  Storiform and periductal (“onion-skin”) fibrosis replacing acini.  Obliterative phlebitis / vasculitis may be present.  Relative sparing of duct epithelium but prominent acinar atrophy and fatty infiltration.  Together, these features fulfill the histopathological criteria for IgG4-related chronic sclerosing sialadenitis (Küttner tumour), the prototypical inflammatory sclerosing disorder of the submandibular (and occasionally parotid) gland. |
| 12.36 | Diagnosis: Adenomatoid hyperplasia of salivary glands; Key Microscopic Features: Hyperplasia of salivary gland lobules filling the lamina propria with hypertrophied acini and ductal ectasia. | Based on the provided hematoxylin and eosin (H&E) stained images of the same lesion at different magnifications, the most appropriate histopathological diagnosis is:  Chronic Sialadenitis  Key Microscopic Features Justifying the Diagnosis:  Lobular Architecture with Acinar Atrophy:  The salivary gland lobules are still discernible, but there is noticeable acinar atrophy—serous acini are reduced in number and size, some appear dilated or degenerated.  Prominent Ductal Changes:  There is ductal dilation, sometimes cystic in appearance, consistent with ductal ectasia.  Some ducts may show epithelial metaplasia.  Chronic Inflammatory Infiltrate:  The interstitial connective tissue is expanded and infiltrated by chronic inflammatory cells—mainly lymphocytes and plasma cells, often in a patchy or periductal distribution.  Occasionally, lymphoid aggregates or germinal centers may be present (not clearly seen here, but characteristic in advanced cases).  Fibrosis:  There is interstitial fibrosis seen in the stroma, separating lobules and replacing areas of atrophied glandular tissue.  Preserved Surface Mucosa:  The overlying oral epithelium appears intact and non-ulcerated, consistent with a chronic rather than an acute or ulcerative process.  These findings are typical of chronic sialadenitis, which often arises due to recurrent or persistent inflammation, frequently associated with ductal obstruction (e.g., sialolithiasis) or autoimmune conditions like Sjögren's syndrome (though additional tests would be needed for confirmation). | Histopathological diagnosis (single best):  Sialadenosis (sialosis) – non-inflammatory, non-neoplastic acinar hypertrophy of the minor salivary glands  Key microscopic features that point to sialadenosis  Feature Appearance in the provided sections Significance  Diffuse enlargement of acini with preserved lobular architecture At low power the submucosa is almost completely occupied by tightly packed, uniform lobules of pale-staining glands; the lobular outlines and thin fibrous septa remain intact. Favour hypertrophic rather than destructive process; architecture is maintained rather than replaced (as would occur in chronic sialadenitis or neoplasm).  Marked acinar hypertrophy Each acinus is noticeably enlarged; cells contain abundant foamy / vacuolated cytoplasm that pushes the small, dark nuclei to the periphery (best appreciated in the higher-power image). Characteristic “ballooned” mucous cells produced by functional hyperactivity of the gland.  Relative lack of ducts and stromal tissue Only an occasional, compressed intercalated/striated duct is seen; interstitial fibrous tissue is minimal and delicate. In sialadenosis, acini dominate and ducts atrophy; fibrosis is not a feature (helps exclude chronic sclerosing or obstructive sialadenitis).  Minimal inflammatory infiltrate or necrosis Scattered lymphocytes/plasma cells may be present in septa, but there is no pervasive chronic inflammation, no suppuration, and no necrotic debris. Confirms the process is non-inflammatory, separating it from chronic or acute sialadenitis.  Intact overlying oral epithelium The surface stratified squamous epithelium is unremarkable except for mild hyperkeratosis; there is no ulceration or pseudo-epitheliomatous hyperplasia. Helps rule out necrotizing sialometaplasia (which typically shows ulceration and squamous metaplasia).  Together, these findings fit the classic picture of sialadenosis: a diffuse, lobule-preserving hypertrophy of salivary acini without significant inflammation, fibrosis, or ductal destruction. |

SALIVARY GLAND NEOPLASMS

| **Fig_no** | **Ground truth** | **GPT 4o answer** | **o3 answer** |
| --- | --- | --- | --- |
| 13.2 | Diagnosis: Intraductal Papilloma; Key Microscopic Features: The low-power view reveals a dilated excretory salivary duct with papillary growth in the lumen. High-power view shows well-organized papillary structures with fibrovascular cores and microcysts. | Based on the provided hematoxylin and eosin (H&E) stained histological images of a salivary gland neoplasm at various magnifications, the most likely histopathological diagnosis is:  Mucoepidermoid Carcinoma (MEC)  Key Microscopic Features Supporting This Diagnosis:  1. Cystic and Solid Architecture (Low Magnification - Image 1):  The lesion shows a lobulated, cystic and solid growth pattern within a well-circumscribed mass.  Numerous mucin-filled cystic spaces are interspersed among solid epithelial nests, typical of MEC.  2. Cellular Composition (Medium Magnification - Image 2):  The tumor comprises a mixture of three cell types, characteristic of MEC:  Mucous cells: Clear to foamy cytoplasm due to intracellular mucin.  Epidermoid (squamoid) cells: Polygonal with eosinophilic cytoplasm.  Intermediate cells: Smaller basaloid cells, often seen between mucous and squamous components.  3. Mucin Production (High Magnification - Image 3):  Presence of intracytoplasmic mucin, often confirmed with special stains (e.g., mucicarmine or PAS-D), but the clear vacuolated appearance here is suggestive.  Duct-like structures or small glandular lumina are occasionally seen.  Mild nuclear atypia and scattered mitotic figures are compatible with low- to intermediate-grade MEC.  Differential Diagnoses Considered but Less Likely:  Pleomorphic adenoma: Lacks significant cystic change and mucous cells.  Acinic cell carcinoma: Shows granular cytoplasm, not the mix of mucous/squamoid cells.  Polymorphous adenocarcinoma: Typically more infiltrative and lacks mucous cell component. | Histopathological diagnosis  → Basal cell adenoma (solid / trabecular-tubular pattern)  Microscopic features that lead to the diagnosis  Field Key observations Significance  Scanning power (image 1) • Completely circumscribed, thinly fibrous-encapsulated nodule.  • Lesion is multinodular, the nodules separated by broad, anastomosing fibrous septa and slit-like clefts (shrinkage artefact). Benign, well-demarcated growth; the “jigsaw-puzzle” partitioning is classic for basal cell adenoma (especially the solid/membranous variant).  Medium power (image 2) • Nodules composed of solid sheets, trabeculae and short tubules of basaloid cells.  • Uniform basaloid cells with scant cytoplasm, round-to-oval nuclei, inconspicuous nucleoli.  • Many islands show a peripheral single-cell palisade; some contain small central lumina.  • Hyaline basement-membrane-like material rims several nests, producing the clear stromal clefts. Combination of solid/trabecular and tubular areas with palisading and hyaline “membranous” material is characteristic of basal cell adenoma and helps exclude pleomorphic adenoma (which would show myxoid/chondroid stroma) and adenoid cystic carcinoma (which is infiltrative and shows cribriform “cylindroma” spaces filled with basophilic matrix).  High power (image 3) • Cells remain cytologically bland – minimal pleomorphism, mitoses rare/absent.  • Two-cell-layer tubules: inner slightly eosinophilic ductal cells and outer darker basaloid/myoepithelial cells.  • No myxochondroid matrix, mucous cells or oncocytic epithelium. Confirms benign nature and rules out mucoepidermoid carcinoma, acinic cell carcinoma, Warthin tumour, etc.  Bottom line:  A well-circumscribed, encapsulated salivary gland tumour composed of uniform basaloid cells arranged in solid, trabecular and tubular patterns with peripheral palisading and abundant basement-membrane-type hyaline material – and without the myxochondroid stroma of pleomorphic adenoma or the cribriform architecture of adenoid cystic carcinoma – is diagnostic of basal cell adenoma. |
| 13.3 | Diagnosis: Inverted Ductal Papilloma; Key Microscopic Features: A bulbous endophytic growth originates from a duct. The luminal surface contains cuboidal-to-columnar cells with scattered mucous cells, showing bland nuclear morphology and microcyst formation. | Histopathological Diagnosis:  Pleomorphic Adenoma (also known as benign mixed tumor)  Key Microscopic Features Supporting the Diagnosis:  1. Varied Architectural Patterns (Image 1 - Low Magnification):  The lesion is well-circumscribed and partially encapsulated.  Shows a mix of epithelial and mesenchymal-like (stromal) components.  Multiple epithelial islands, cords, and duct-like structures are embedded in a chondromyxoid stroma—a hallmark of pleomorphic adenoma.  2. Epithelial Component (Image 2 - Intermediate Magnification):  Epithelial cells arranged in trabecular, ductal, and nest-like patterns.  Duct-like structures lined by inner luminal and outer myoepithelial cells.  Some areas suggest squamous or plasmacytoid differentiation, typical of the diverse epithelial morphology in pleomorphic adenoma.  3. Stromal Component (Visible in all images, especially Image 1 & 2):  Myxoid and chondroid stroma, which mimics cartilage, is a distinctive feature.  Loose fibrous stroma with regions appearing hyalinized, mixed with mucopolysaccharide-rich matrix.  4. Cellular Details (Image 3 - High Magnification):  Bland, uniform nuclei with inconspicuous nucleoli.  Low mitotic activity.  Absence of significant atypia or invasive growth pattern supports benign nature. | Histopathological diagnosis  Basal cell adenoma (tubular / solid-trabecular pattern) of a salivary gland  Key microscopic features in the three H-E fields that support this diagnosis  Field What is seen Significance  Low-power overview (first image) • A solitary, sharply circumscribed–capsulated nodule sitting within otherwise normal salivary tissue.  • The tumour forms a convoluted, “jigsaw/puzzle-piece” mass of dark-purple epithelium; several slit-like true lumina and larger cystic spaces are present but there is no chondromyxoid or lymphoid stroma. A well-demarcated, encapsulated mass argues for a benign basaloid tumour. Absence of myxoid/chondroid matrix helps rule out pleomorphic adenoma; absence of lymphoid tissue helps rule out Warthin tumour.  Intermediate magnification (second image) • Interlacing cords and papillary infoldings composed of two cell layers: a rim of basaloid cells with hyperchromatic, palisaded nuclei and an inner layer of slightly paler cells lining true ducts/canaliculi.  • Thick eosinophilic, basement-membrane-like material surrounds the epithelial nests and fills some stromal clefts. A dual cell layer plus prominent hyaline basement-membrane material is characteristic of basal-cell–type tumours, especially basal cell adenoma.  High power (third image) • Uniform basaloid cells with scant cytoplasm, round–oval nuclei, inconspicuous nucleoli, very low mitotic activity.  • Peripheral nuclear palisading and small intratumoral duct-like spaces containing eosinophilic secretions; occasional micro-cysts/vacuoles but no significant cytologic atypia. Cytologic blandness and palisading confirm a benign basal cell proliferation; the duct-forming tendency is typical for the tubular / solid-trabecular variants of basal cell adenoma.  Integrating these findings  The combination of encapsulation, basaloid bilayered epithelium, peripheral nuclear palisading, hyaline basement-membrane material, and lack of myxoid or chondroid stroma is diagnostic for basal cell adenoma.  Other common salivary tumours are excluded by the key negatives noted above (no myxoid/chondroid matrix ⇒ not pleomorphic adenoma; no lymphoid stroma ⇒ not Warthin tumour; no infiltrative edges or perineural invasion ⇒ not adenoid cystic carcinoma or basal cell adenocarcinoma).  Hence, the single most appropriate histopathological diagnosis for the lesion pictured is Basal cell adenoma. |
| 13.4 | Diagnosis: Sialadenoma Papilliferum; Key Microscopic Features: The surface shows squamous papillary proliferation with adenomatous ductal proliferation at the base. High-power view reveals adenomatous proliferation with papillary hyperplasia, cuboidal luminal cells with spindled nuclei, and subtle cribriform architecture. | Based on the histopathological examination of the provided hematoxylin and eosin (H&E) stained images at low, intermediate, and high magnifications, the single most likely diagnosis is:  Papillary Cystadenocarcinoma (a low-grade malignant salivary gland tumor)  🔍 Key Microscopic Features Observed:  1. Papillary and cystic architecture (seen best in the first/lower magnification image):  Multiple papillary projections into cystic spaces are evident.  The lesion shows exophytic, frond-like papillary structures, supported by fibrovascular cores.  2. Epithelial lining and cellular morphology (intermediate magnification):  The papillae and cystic spaces are lined by cuboidal to columnar epithelial cells.  The nuclei are mildly pleomorphic and show mild atypia, consistent with low-grade malignancy.  Presence of intraluminal papillary fronds and cribriform or glandular areas.  3. Evidence of invasion and mitotic activity (high magnification):  Infiltrative growth pattern into the surrounding stroma, a key diagnostic feature for carcinoma.  Vascular structures containing red blood cells are surrounded by tumor epithelium, further supporting the malignant nature.  Mitotic figures are visible but not numerous, which aligns with the low-grade behavior of this tumor.  📌 Diagnostic Rationale:  The papillary and cystic growth pattern along with invasive epithelial features and mild cytologic atypia support a diagnosis of Papillary Cystadenocarcinoma, distinguishing it from:  Cystadenoma (which lacks invasion),  Mucoepidermoid carcinoma (which shows more mucin-producing and intermediate cells),  Adenoid cystic carcinoma (which has a characteristic cribriform pattern but lacks prominent papillary structures). | Histopathological diagnosis  ► Sialadenoma papilliferum (benign papillary salivary-duct neoplasm).  Microscopic features in the provided H&E sections that support this diagnosis  Field Key observations Diagnostic relevance  Scanning (first image) • Fungating, cauliflower-like, papillary mass that is continuous with the surface oral mucosa.  • Broad papillary fronds possess central fibro-vascular cores.  • Beneath the surface, a lobulated papillary / cystic epithelial proliferation extends into the submucosa and merges with adjacent minor salivary glands. Classic biphasic architecture of sialadenoma papilliferum: an exophytic squamous papillary component and an underlying intraductal papillary salivary component.  Intermediate magnification (second image) • Duct-like and cystic spaces with complex, arborising papillary infoldings.  • Papillae are lined by double-layered epithelium: an inner cuboidal/columnar ductal cell layer and an abluminal myoepithelial/basal layer that blends with surrounding stroma.  • No destructive infiltration of the stroma. Intraductal papillary growth pattern without invasion distinguishes it from papillary cystadenocarcinoma or salivary duct carcinoma.  High magnification (third image) • Lining cells show uniform round-to-oval nuclei, fine chromatin, inconspicuous nucleoli, and scant mitotic activity—i.e. bland cytology.  • Occasional apocrine-type cytoplasmic snouts and focal mucinous vacuoles may be seen, but no koilocytosis or oncocytic crowding typical of Warthin tumour.  • Intact myoepithelial layer identifiable around many ducts. Cytologic blandness and retention of the myoepithelial layer corroborate a benign intraductal lesion (sialadenoma papilliferum) rather than a malignant papillary carcinoma.  Summary rationale  The combination of (1) a papillomatous squamous surface component that opens directly into (2) a richly papillary, cystically dilated salivary-duct proliferation anchored in the minor salivary gland, together with bland cytology and a preserved myoepithelial rim, is pathognomonic for sialadenoma papilliferum. Other papillary salivary lesions (papillary cystadenoma, Warthin tumour, salivary duct carcinoma) either lack the characteristic surface-to-duct continuity, show oncocytic lymphoid stroma, or display overt atypia/necrosis—all absent in these sections. |
| 13.6 | Diagnosis: Pleomorphic Adenoma; Key Microscopic Features: The tumor is encapsulated with numerous ducts and a prominent myxochondroid stroma. Ducts are surrounded by myoepithelial cells that merge into hyalinized stroma. | Based on the provided hematoxylin and eosin (H&E) stained histological images at different magnifications, the single most likely histopathological diagnosis is:  Pleomorphic Adenoma (Benign Mixed Tumor)  Key Microscopic Features Supporting the Diagnosis:  Well-circumscribed lesion (seen in the low-power image):  The lesion is encapsulated or well demarcated from the surrounding tissue, typical of a benign tumor.  Biphasic pattern with both epithelial and myoepithelial components:  Ductal structures lined by cuboidal to columnar epithelial cells are present.  Intermixed are myoepithelial cells, some with plasmacytoid (eccentric nucleus) appearance, contributing to the tumor’s cellular diversity.  Chondromyxoid stroma:  Prominent in the high magnification image (last image), this characteristic matrix is pink, hyalinized to myxoid, and sometimes resembles cartilage.  This feature is pathognomonic for pleomorphic adenoma and reflects the “pleomorphic” nature of the tumor.  Architectural diversity:  Mixture of duct-like structures, solid nests, and trabecular arrangements.  This heterogeneity in architecture is a hallmark of pleomorphic adenoma. | Histopathological diagnosis (single best):  Pleomorphic adenoma (benign mixed tumour) of a salivary gland  Key microscopic features in the provided H-E sections that support this diagnosis  Field-of-view Salient findings Significance for diagnosis  Low-power overview (first image) • Well-circumscribed, lobulated mass surrounded by a thin fibrous capsule  • Tumour parenchyma shows a patchy, biphasic appearance – darker cellular zones alternating with pale myxoid/chondroid zones A circumscribed, encapsulated lesion with mixed cellular and stromal areas is classic for a benign‐mixed tumour (pleomorphic adenoma).  Cell-rich areas (second image, high power) • Numerous small, round-to-oval duct-like and gland-like structures with luminal eosinophilic secretions  • Dual cell population: an inner layer of cuboidal epithelial cells and an outer layer of myoepithelial/plasmacytoid cells  • Intervening fibrous septa A double-layered duct/tubule pattern produced by epithelial and myoepithelial cells is a hallmark of pleomorphic adenoma.  Intermediate zones (third image) • Cords and strands of myoepithelial cells merge imperceptibly into an abundant pale, fibrillary myxoid stroma  • Gradual transition from cellular to stromal component The ability of myoepithelial cells to elaborate a myxoid (sometimes chondroid) matrix creates the characteristic “pleomorphic” architecture.  Stromal-dominant foci (fourth image) • Dense, eosinophilic, hyaline to chondroid-like matrix containing scattered, entrapped tumour cells in lacuna-like spaces  • Relative paucity of inflammatory cells or necrosis Cartilage-like (chondromyxoid) areas within an otherwise epithelial tumour are virtually pathognomonic for pleomorphic adenoma among salivary lesions.  Additional integrative clues  The intimate admixture of epithelial tubules, myoepithelial nests, and a variably myxoid-chondroid stromal matrix gives rise to the “pleomorphic” (mixed) pattern for which the tumour is named.  The absence of infiltrative borders, perineural invasion, necrosis, or significant cytologic atypia further favours a benign pleomorphic adenoma over malignant mimickers such as mucoepidermoid or adenoid cystic carcinoma.  These collective microscopic features align perfectly with a diagnosis of pleomorphic adenoma in the salivary gland group of oral & maxillofacial pathology. |
| 13.7 | Diagnosis: Pleomorphic Adenoma; Key Microscopic Features: The cystic tumor features myxoid and hyalinized stroma, with ducts surrounded by myoepithelial cells. Some myoepithelial cells are plasmacytoid, while others in the myxoid stroma are spindled. | Histopathological Diagnosis:  Adenoid Cystic Carcinoma (ACC) of the salivary gland.  Key Microscopic Features Supporting the Diagnosis:  Cribriform Pattern (Swiss-cheese appearance):  Most notably seen in the third and fourth images.  Tumor cells are arranged around pseudocystic spaces filled with eosinophilic, basophilic, or hyaline material—classic for the cribriform subtype of ACC.  Basaloid Epithelial Cells:  Uniform, small, dark-staining nuclei with scant cytoplasm.  Prominent in the second image, where the infiltrative nests and cords of basaloid cells surround cyst-like spaces.  Perineural Invasion (Common in ACC):  While not definitively shown in these particular sections, the infiltrative growth pattern and dense cellularity suggest aggressive behavior typical of perineural involvement.  Hyaline Stromal Material:  Seen in the third and fourth images, the extracellular hyaline-like material within the pseudocysts is characteristic of ACC.  Multilobulated and Infiltrative Architecture:  The first image at low magnification shows a lobulated tumor mass with infiltrative borders, common in malignant salivary neoplasms like ACC. | Histopathological diagnosis  → Adenoid cystic carcinoma (ACC) of salivary gland  Key microscopic (H-E) features in the provided panels that support this diagnosis  Image Salient findings Significance for ACC  Low-power overview (first panel) • Lobulated, uncapsulated / poorly-circumscribed mass infiltrating adjacent glandular tissue.  • Multiple epithelial lobules separated by fibrous-hyaline septa; several large, empty-appearing pseudocystic spaces filled with pale eosinophilic basement-membrane-like material. ACC is typically infiltrative rather than encapsulated and shows lobular architecture with pseudocystic (“cylindromatous”) spaces.  Intermediate magnification (second panel) • “Swiss-cheese” cribriform pattern: nests of small, hyperchromatic basaloid cells perforated by round to oval spaces containing bright eosinophilic, PAS-positive hyaline material.  • Tumour cells are monotonous, scant cytoplasm, angulated nuclei; occasional targetoid arrangement around nerve twigs (early perineural growth). The cribriform (“cylindromatous”) architecture with hyaline cylinders is the classic hallmark of ACC.  Higher magnification – tubular area (third & fourth panels) • Tubular pattern: numerous tiny true ducts / tubules with a dual cell layer – inner ductal (darker, eosinophilic) and outer myoepithelial/basaloid cells – embedded in a densely hyalinised stroma.  • Stroma shows laminated, swirling basement-membrane-like material surrounding and sometimes replacing tumour nests. ACC frequently displays more than one growth pattern in the same tumour; the tubular pattern with heavy stromal hyalinisation is characteristic and helps distinguish ACC from other basaloid tumours.  Together, the combination of:  an infiltrative, lobulated tumour without a true capsule  the diagnostic cribriform “Swiss-cheese” nests filled with hyaline cylinders  co-existent tubular areas with a two-cell lining in a dense hyalinised matrix  uniform small basaloid nuclei, scant cytoplasm, and early perineural-type arrangement  …points decisively to adenoid cystic carcinoma rather than pleomorphic adenoma, basal-cell adenoma, polymorphous adenocarcinoma or other salivary neoplasms. |
| 13.8a | Diagnosis: Pleomorphic Adenoma; Key Microscopic Features: The tumor exhibits extension into the capsule via pseudopods, with reticulated areas of pleomorphic adenoma. | Based on the hematoxylin and eosin (H&E) stained histopathology images you provided, which show a salivary gland neoplasm at low and high magnification, the most likely diagnosis is:  Mucoepidermoid Carcinoma (MEC)  Key Microscopic Features Supporting the Diagnosis:  Low-Power Image (First Image):  Lobulated architecture with infiltrative growth beneath an intact surface epithelium.  Presence of solid and cystic areas, a hallmark of mucoepidermoid carcinoma.  Variable cellularity indicating a heterogeneous tumor population.  High-Power Image (Second Image):  Mixture of three distinct cell types:  Mucous cells: Large, pale-staining cells with foamy cytoplasm due to mucin production.  Epidermoid (squamoid) cells: Cells with eosinophilic cytoplasm and more prominent intercellular bridges.  Intermediate cells: Polygonal cells with less cytoplasm and centrally located nuclei, appearing between mucous and epidermoid types.  Cystic degeneration and mucin pooling within glandular spaces.  No evidence of perineural or vascular invasion in these fields, although that would depend on broader sampling. | Most likely histopathological diagnosis  ➜ Adenoid cystic carcinoma (cribriform / tubular pattern)  Key microscopic features supporting this diagnosis  Field-of-view What can be seen Why it points to adenoid cystic carcinoma  Low-power overview (first image) Submucosal, unencapsulated but sharply circumscribed nodule lying immediately beneath an intact oral epithelium; tumour formed by innumerable tiny epithelial islands, cords and nests separated by delicate fibrous septa; many of the islands contain pale “empty” round spaces Lack of capsule and close packing of small epithelial nests is typical of a malignant minor-salivary-gland tumour; the multitude of regularly distributed punched-out spaces is classic for the cribriform (“Swiss-cheese”) architecture of adenoid cystic carcinoma  High-power view (second image) • Cribriform pattern: basaloid tumour cells create sieve-like nests with multiple round/oval pseudocysts  • Tubular structures with a dual cell layer – an inner ductal line of slightly eosinophilic cuboidal cells and an outer myoepithelial/basaloid layer with hyperchromatic angular nuclei  • Pseudocysts and some true ducts contain homogeneous eosinophilic, hyaline basement-membrane-like material  • Cells are small, uniform, hyperchromatic with scant cytoplasm; mitoses are infrequent The combination of basaloid cytology, dual cell population, hyaline pseudocystic spaces and the cribriform/tubular growth pattern is virtually diagnostic of adenoid cystic carcinoma and distinguishes it from other salivary tumours such as muco-epidermoid carcinoma, acinic-cell carcinoma or pleomorphic adenoma  Additional subtle findings (seen focally in the sections) Foci of hyalinised stromal septa and small perivascular or perineural-looking clefts Perineural tropism and stromal hyalinisation are characteristic ancillary clues to adenoid cystic carcinoma  These histological hallmarks collectively establish adenoid cystic carcinoma as the single most plausible diagnosis for the salivary-gland-type lesion illustrated. |
| 13.8b | Diagnosis: Pleomorphic Adenoma; Key Microscopic Features: The cellular pleomorphic adenoma is composed of tightly packed cells with minimal stroma and no significant atypia. | Based on the provided hematoxylin and eosin (H&E) stained histopathological images of a salivary gland neoplasm, observed at high and low magnifications, the single most likely diagnosis is:  Diagnosis:  Mucoepidermoid Carcinoma (MEC)  Key Microscopic Features Supporting the Diagnosis:  High Magnification (First Image):  Mixture of cell types:  Mucous cells: Seen as cells with abundant foamy or vacuolated cytoplasm (e.g., the large pale-staining cell centrally located in the image).  Intermediate cells: Polygonal cells with less cytoplasm, appearing between mucous and squamous cells.  Epidermoid (squamous) cells: Cells with eosinophilic cytoplasm and intercellular bridges in some areas.  Moderate nuclear pleomorphism: Variably sized nuclei, some with prominent nucleoli.  Scattered mitotic figures: Indicating proliferative activity, although not excessive.  Low Magnification (Second Image):  Lobular and cystic architecture: The tumor shows solid and cystic areas in a lobulated arrangement.  Infiltrative growth pattern: Poorly circumscribed, with tumor cells blending into the surrounding tissue.  Mucin pools: Suggestive of mucin-secreting cells, although not overtly abundant here.  No clear encapsulation, supporting a malignant lesion.  Additional Notes:  Mucoepidermoid carcinoma is the most common malignant salivary gland tumor, frequently affecting the parotid gland but can arise in minor salivary glands (e.g., palate).  Histologic grading (low, intermediate, high) is based on the proportion of mucous cells, mitotic activity, and cystic components. This lesion, with a balanced mixture of cell types and mild-to-moderate atypia, is consistent with intermediate-grade MEC. | Histopathological diagnosis  → Spindle-cell myoepithelioma (benign myoepithelioma) of salivary gland  Key microscopic features supporting this diagnosis  Field What is seen Why it points to myoepithelioma  Low-power architecture • Sharply circumscribed / thin fibrous capsule under the surface mucosa.  • Tumour parenchyma is almost uniformly cellular with only small stromal islands. A well-defined capsule favours a benign lesion; the lack of lobulation or obvious duct-rich areas argues against pleomorphic adenoma and other mixed tumours.  Cell population • Sheets and interlacing spindle-shaped myoepithelial cells with elongated, fairly uniform, dark nuclei and scant pale-eosinophilic cytoplasm.  • No obvious ductal / acinar differentiation. A tumour made almost entirely of myoepithelial cells, without ductal elements, is the defining feature of myoepithelioma.  Stroma & extracellular material • Scattered acellular, homogeneous eosinophilic (hyaline / basement-membrane-like) globules and strands between the tumour cells.  • Little or no chondromyxoid (blue–grey) matrix. Hyaline basement-membrane droplets are characteristic of myoepithelial tumours (especially the spindle-cell and plasmacytoid forms). Their presence together with minimal myxoid/chondroid stroma differentiates it from pleomorphic adenoma.  Cytological atypia & mitoses • Nuclei are monotonous with inconspicuous nucleoli.  • Mitotic figures, necrosis, and infiltrative edges are not appreciable in the sections provided. The bland cytology and encapsulation support a benign myoepithelioma rather than a myoepithelial carcinoma.  Together, the encapsulated nodule composed almost exclusively of spindle-shaped myoepithelial cells, absence of true ducts, and presence of focal eosinophilic basement-membrane material constitute the classic picture of a spindle-cell myoepithelioma of a salivary gland. |
| 13.9 | Diagnosis: Myxoid Pleomorphic Adenoma; Key Microscopic Features: The myxoid tumor contains islands and cords of tumor cells, with rare ductal structures present. Epithelioid and spindled myoepithelial cells are found within the myxoid stroma. | Based on the provided hematoxylin-and-eosin (H&E) stained histological images of a salivary gland neoplasm at low and high magnification, the most likely histopathological diagnosis is:  Basal Cell Adenoma (BCA)  Justification with Key Microscopic Features:  1. Architectural Pattern:  The low-power image shows a well-circumscribed and encapsulated lesion, a hallmark of benign neoplasms like basal cell adenoma.  The tumor exhibits a lobulated architecture with basaloid cell proliferation arranged in tubular and trabecular patterns—typical for BCA.  2. Cellular Features (High Magnification):  The higher magnification reveals uniform basaloid cells with:  Hyperchromatic nuclei  Scant cytoplasm  Peripheral palisading of nuclei, especially in the cords and islands of cells—this is a classic feature of BCA.  Absence of significant mitotic figures, necrosis, or nuclear pleomorphism supports a benign diagnosis.  3. Stromal Features:  The intervening fibrous stroma is relatively sparse and lacks the myxochondroid change seen in pleomorphic adenoma.  No chondroid, myxoid, or mucoid areas are visible, helping to rule out pleomorphic adenoma.  Differential Diagnoses to Consider (and Rule Out):  Pleomorphic adenoma: Ruled out due to absence of myxoid or chondroid stroma.  Adenoid cystic carcinoma: Lacks perineural invasion, and no cribriform architecture is seen.  Basal cell adenocarcinoma: Unlikely due to absence of infiltrative growth, mitoses, and cytological atypia. | Histopathological diagnosis  Polymorphous adenocarcinoma (formerly “polymorphous low-grade adenocarcinoma”) of a minor salivary gland  Microscopic features in the supplied H-E sections that support the diagnosis  Field Observations in your slides Diagnostic value  Low-power architecture (first image) Well-circumscribed, lobulated mass lying just beneath oral mucosa; separated by fine fibrous septa. Within the lobules you can appreciate a mixture of growth patterns – tubular, cribriform and streaming cords (“swiss-cheese” / target-like areas). A polymorphous mixture of patterns is the hallmark of polymorphous adenocarcinoma and helps distinguish it from tumors with a single dominant architecture (e.g., basal-cell adenoma).  Cellular uniformity Throughout the lesion the cells are bland, small, round-to-oval and cytologically monotonous; nuclei are evenly dispersed chromatin with inconspicuous nucleoli; mitoses are scarce. Monomorphic cytology despite architectural diversity is characteristic of polymorphous adenocarcinoma and contrasts with the hyperchromatic, angulated nuclei of adenoid cystic carcinoma.  Pattern at higher magnification (second image) Single-file / “Indian-file” cords and narrow tubules weave through a fibrous-hyalinized stroma; occasional small lumina are present but no mucous cells, epidermoid cells or chondromyxoid matrix. Streaming or single-file infiltration is very typical of polymorphous adenocarcinoma. Absence of mucocytes rules out mucoepidermoid carcinoma; absence of myxochondroid stroma argues against pleomorphic adenoma.  Stroma Delicate, lightly eosinophilic fibrous-hyaline stroma closely surrounds epithelial structures. Fits the fibrohyaline background usually seen in polymorphous adenocarcinoma; basement-membrane-type hyaline cylinders of adenoid cystic carcinoma are not identified.  Circumscription vs. invasion Although well-outlined, the lesion shows tongues of tumor insinuating between collagen bundles, a feature often accompanied (in deeper levels) by perineural encirclement. Polymorphous adenocarcinoma is typically “well-demarcated yet infiltrative,” and perineural invasion is very common (even if not captured in the submitted fields).  Taken together—polymorphous architectural patterns plus uniform cytology, single-file cords, fibrohyaline stroma and absence of other cell types or myxochondroid matrix—the findings point convincingly to polymorphous adenocarcinoma. |
| 13.10 | Diagnosis: Pleomorphic Adenoma; Key Microscopic Features: The pleomorphic adenoma exhibits squamous metaplasia with keratin formation, while ducts contain mucous cells and crystalline structures. | Based on the provided hematoxylin-and-eosin (H&E) stained histological images taken at different magnifications, the most consistent histopathological diagnosis is:  Diagnosis:  Adenoid Cystic Carcinoma (AdCC) of the salivary gland  Key Microscopic Features Supporting the Diagnosis:  Cribriform (“Swiss Cheese”) Pattern:  Seen prominently in both images.  Multiple pseudocystic spaces surrounded by basaloid epithelial cells. These spaces are filled with eosinophilic basement membrane-like material (appearing pink in H&E stain).  Basaloid Cell Population:  Small, hyperchromatic, darkly staining nuclei with scant cytoplasm—typical of AdCC.  These cells often form nests and cords, visible especially in the lower magnification image.  Perineural Invasion (Potentially present):  Although not clearly demonstrated in these images, AdCC is notorious for perineural invasion, and it should be actively looked for in additional sections.  Hyaline Globules:  The lumina (cyst-like spaces) contain PAS-positive, hyaline (basement membrane-like) material, typical of AdCC. While PAS staining isn’t shown, the eosinophilic material is a key clue.  Desmoplastic Stroma:  The tumor nests are embedded in a fibrous (pink-staining) stroma, another classic feature.  Summary:  These features—particularly the cribriform pattern with basaloid cells and hyaline material—are hallmark findings of Adenoid Cystic Carcinoma, one of the most classic and diagnostically distinct malignant salivary gland tumors. This tumor is often slow-growing but locally aggressive and prone to perineural spread. | Histopathological diagnosis – Adenoid Cystic Carcinoma (ACC)  ( “Swiss-cheese” / cribriform salivary gland carcinoma )  Key microscopic features visible in the two H-E sections  # Feature in the images Why it points to ACC  1 Cribriform (“Swiss-cheese”) architecture – nests of small basaloid cells perforated by many round to oval spaces The hallmark pattern of classic ACC; other salivary tumours rarely show such uniform, punched-out pseudocysts  2 Tubular structures with two cell layers – an inner duct-type cuboidal layer and an outer myoepithelial/basaloid layer The dual cell population forming true ducts is characteristic of ACC’s tubular component  3 Pseudocystic lumina packed with eosinophilic, basement-membrane-like hyaline material (“ball-of-yarn” globules in the high-power view) ACC produces abundant type IV collagen and other basement-membrane constituents that fill the pseudocysts; these globules are PAS-positive and unique for ACC  4 Uniform, small, hyperchromatic, angular nuclei with scant cytoplasm forming solid basaloid sheets between the cribriform spaces Cytologic monotony plus basaloid appearance helps separate ACC from pleomorphic adenoma (which shows plasmacytoid myoepithelium and chondromyxoid stroma)  5 Hyalinised, fibrous stroma dissecting and girdling the tumour islands (seen best on the low-power image) ACC frequently elicits dense stromal hyalinisation, sometimes giving a “cell nests-in-cement” look  6 (Noted focally) Angulated empty spaces suggestive of perineural tracking While not prominent in these fields, perineural invasion is a classic behaviour of ACC and should be sought on the whole slide  Taken together, the cribriform/tubular dual pattern, basement-membrane-rich pseudocysts, small basaloid cells, and hyalinised stroma are diagnostic of adenoid cystic carcinoma among the salivary gland neoplasms. |
| 13.11 | Diagnosis: Pleomorphic Adenoma; Key Microscopic Features: The tumor features sheets of plasmacytoid myoepithelial cells with focal duct formation, as well as cells with eccentric pleomorphic nuclei and abundant eosinophilic cytoplasm. | Based on the provided hematoxylin-and-eosin (H&E) stained histopathological images of a salivary gland lesion at low and high magnifications, the most likely diagnosis is:  Mucoepidermoid Carcinoma (MEC)  Key Microscopic Features Supporting the Diagnosis:  1. Mixed Cell Population (seen clearly in high magnification image):  Mucous cells: Large cells with abundant foamy or pale cytoplasm and basally located nuclei, indicating intracellular mucin.  Epidermoid (squamoid) cells: Polygonal cells with eosinophilic cytoplasm and prominent intercellular bridges.  Intermediate cells: Smaller, round-to-oval cells with less cytoplasm and more hyperchromatic nuclei.  2. Cystic and Solid Growth Patterns (seen in low magnification image):  Presence of both solid nests and cystic spaces lined by tumor cells.  Solid areas are composed of a mixture of mucous, epidermoid, and intermediate cells, showing variable architectural patterns.  3. Mucin Production:  Mucous cells are evident, and mucin pooling may be seen (especially with special stains like mucicarmine, but the cytoplasmic mucinous appearance is evident even with H&E).  4. Infiltrative Growth Pattern:  Irregular tumor nests and islands infiltrating through salivary gland parenchyma.  Summary Diagnosis:  Mucoepidermoid Carcinoma (MEC) — a malignant salivary gland neoplasm characterized by a combination of mucous, intermediate, and epidermoid cells, and exhibiting both cystic and solid architectural patterns. | Histopathological diagnosis (single best fit)  Low-grade acinic cell carcinoma of salivary gland (serous-acinar type).  Key microscopic features supporting this diagnosis  What is seen in the submitted slides How it points to acinic-cell carcinoma  1. Lobulated, solid–microcystic growth pattern The low-power field shows a well-circumscribed but un-encapsulated nodule divided by delicate fibrous septa into lobules composed of tightly packed cells with numerous tiny microcystic/alveolar spaces. A classic architectural pattern of acinic-cell carcinoma; pleomorphic adenoma would have chondromyxoid stroma, and mucoepidermoid carcinoma usually shows large cystic spaces and mixed cell populations.  2. Uniform polygonal cells with granular/bubbly cytoplasm On high-power the tumour cells are polygonal with abundant lightly eosinophilic to amphophilic cytoplasm that contains fine granules and small vacuoles, giving a “bubbly” appearance. The cytoplasmic basophilic (zymogen) granules and micro-vacuolation are characteristic of serous acinar differentiation.  3. Round centrally located (occasionally eccentric) nuclei, inconspicuous mitoses Nuclei are round to oval, mostly centrally placed, with finely granular chromatin and occasional small nucleoli; mitotic figures are rare. These bland nuclear features are typical of low-grade acinic-cell carcinoma and help distinguish it from high-grade salivary malignancies.  4. Preservation of duct-like structures at the periphery Residual native salivary ducts and occasional tumour-formed tubules are visible at the edge of lobules. Acinic-cell carcinoma often infiltrates between and around pre-existing ducts without destroying them, unlike many other salivary tumours.  5. Scant, delicate fibrovascular stroma without lymphoid tissue or chondromyxoid matrix The stroma is thin and fibrous; there is no lymphoid stroma (rules out Warthin tumour) and no myxochondroid material (rules out pleomorphic adenoma). The absence of these stromal elements supports acinic-cell carcinoma over its main benign mimics.  Taken together, the lobulated microcystic architecture, the serous-acinar-type cytoplasm with fine zymogen granules/vacuoles, bland round nuclei and paucity of mitoses provide a constellation that is diagnostic of acinic cell carcinoma. |
| 13.13 | Diagnosis: Carcinoma ex pleomorphic adenoma of the upper lip; Key Microscopic Features: Pleomorphic adenoma with extensive hyalinization, alongside adenocarcinoma on the right. Remnant pleomorphic adenoma and area of adenocarcinoma with comedonecrosis. | Histopathological Diagnosis:  Salivary Duct Carcinoma (SDC)  Justifying Key Microscopic Features:  Upon analyzing the hematoxylin and eosin (H&E) stained images at multiple magnifications, the following hallmark features support the diagnosis of Salivary Duct Carcinoma, a high-grade malignant salivary gland tumor:  1. Cribriform and Comedo-like Architecture (Image 2)  Glandular and duct-like structures with a cribriform growth pattern.  Central comedo-type necrosis resembling ductal carcinoma of the breast.  Tumor cells form Roman bridge-like patterns with punched-out spaces.  2. Perineural Invasion (Image 3)  A nerve bundle is encased by malignant epithelial cells, indicative of aggressive behavior and typical of SDC.  3. High-Grade Cytology (Image 4)  Marked cellular atypia, pleomorphism, prominent nucleoli, and mitotic activity.  Frequent apoptotic bodies and necrosis.  4. Infiltrative Growth and Desmoplastic Stroma (Image 1)  At low magnification, there's an infiltrative tumor within a dense desmoplastic stroma.  Irregular tumor nests and ducts penetrate surrounding tissue, a key sign of malignancy.  Conclusion:  The constellation of high-grade cytologic features, ductal architecture with comedonecrosis, and perineural invasion, along with the infiltrative pattern, all point toward Salivary Duct Carcinoma (SDC). This aggressive tumor commonly affects the parotid gland and histologically mimics high-grade ductal carcinoma of the breast. | Histopathological diagnosis — Adenoid cystic carcinoma (ACC), predominantly cribriform‐tubular pattern  Key microscopic features seen in the provided H&E sections  Field-of-view Salient findings Diagnostic relevance  Low-power overview (image 1) • Infiltrative, lobulated tumor replacing salivary gland parenchyma  • Dense, fibrohyaline stroma separating irregular nests and cords of dark-staining cells (“islands in a sea of collagen”) Infiltrative growth with desmoplasia is typical of malignant salivary tumors; the lobulated architecture is characteristic of ACC  Intermediate magnification (image 2) • Numerous round to oval pseudocystic/cribriform spaces creating a “Swiss-cheese” appearance  • Lumina appear empty or contain pale eosinophilic material (basement-membrane–like globules)  • Tumor cells are uniform, basaloid, with scant cytoplasm and hyperchromatic, angulated nuclei The cribriform pattern with pseudolumina filled by hyaline material is the single most distinctive feature of ACC  High magnification – hyaline globule (image 3) • Central spherical, densely eosinophilic hyaline cylinder surrounded radially by a cuff of basaloid cells  • Tumor nests embedded in fibrohyaline stroma Tubular/cribriform units with hyaline cylinders represent reduplicated basal lamina—hallmark of ACC  High magnification – solid focus (image 4) • Areas of solid basaloid cells with increased nuclear-to-cytoplasmic ratio, occasional clear cytoplasmic vacuoles  • Intervening stromal strands and early necrosis in some nests Solid areas help confirm ACC (solid type has worse prognosis) and distinguish it from polymorphous adenocarcinoma, which lacks solid basaloid sheets  Ancillary clues (not all captured but commonly present in ACC)  Perineural invasion and angular infiltration along nerve sheaths  Dual cell population (ductal & myoepithelial/basal) highlighted by immunohistochemistry (e.g., CD117, p63, SMA)  Taken together, the cribriform “Swiss-cheese” architecture, hyaline pseudocysts, uniform basaloid cells, and infiltrative fibrohyaline stroma firmly support the diagnosis of adenoid cystic carcinoma in a salivary gland site. |
| 13.14 | Diagnosis: Spindle cell myoepithelioma of the palate; Key Microscopic Features: Spindle cell proliferation leading to cartilage and fat formation. Spindled myoepithelial cells in association with osteochondroid material. | Based on the provided hematoxylin and eosin (H&E) stained histopathological images of a salivary gland neoplasm at different magnifications, the most likely diagnosis is:  Pleomorphic Adenoma (Benign Mixed Tumor)  Key Microscopic Features Supporting the Diagnosis:  1. Biphasic Pattern:  The lesion exhibits both epithelial and mesenchymal (stromal) components, a hallmark of pleomorphic adenoma.  In the lower magnification image, you can see well-circumscribed areas with cellular heterogeneity and intervening stromal elements.  2. Epithelial Elements:  Glandular structures and duct-like formations are evident, lined by cuboidal to columnar epithelial cells.  Plasmacytoid myoepithelial cells are visible in the higher magnification image — these have eccentric nuclei and eosinophilic cytoplasm.  3. Chondromyxoid Stroma:  The stroma in the lesion shows myxoid to chondroid differentiation, seen as pale, basophilic, and variably dense areas. This characteristic "cartilage-like" stroma is typical of pleomorphic adenoma.  The higher magnification clearly displays mucoid and chondroid areas with embedded epithelial islands.  4. Well-Circumscribed Borders:  Although not encapsulated in some regions, the tumor appears well-demarcated, which is common in pleomorphic adenoma, especially in minor salivary gland sites.  Conclusion:  The combination of ductal epithelial structures, myoepithelial components, and a myxochondroid stroma, along with the well-circumscribed architecture, is diagnostic of pleomorphic adenoma, the most common benign salivary gland tumor. | Histopathological diagnosis (single best):  Pleomorphic adenoma (benign mixed tumour) of a minor salivary gland  Key microscopic features that support this diagnosis  Feature How it appears in the provided slides Why it points to pleomorphic adenoma  Well-circumscribed/encapsulated nodule Low-power image shows a lobulated mass separated from surrounding salivary tissue by a delicate fibrous rim. Benign salivary tumours such as pleomorphic adenoma are typically encapsulated; malignant counterparts breach the capsule.  Biphasic cell population  • Ductal/epithelial cells  • Myoepithelial cells (spindle-, plasmacytoid- or stellate-shaped) On the higher-power image, small round to oval ducts lined by cuboidal cells sit adjacent to sheets and fascicles of bland spindle/plasmacytoid cells. The admixture of true ducts + myoepithelial elements is characteristic; tumours composed purely of one or the other (e.g., myoepithelioma) lack this duality.  Chondromyxoid (“cartilage-like”) stroma Extensive pale-purple, slightly basophilic matrix with lacuna-like white holes resembling cartilage; focal hyalinisation is also present. Production of abundant chondroid/ muco-myxoid matrix by myoepithelial cells is the hallmark of pleomorphic adenoma and the reason for the term “mixed” tumour.  Architectural pleomorphism The tumour shows intermingled solid, trabecular, cystic and stromal areas within the same lesion. The constantly changing (“pleomorphic”) growth pattern—without cytologic atypia—gives the tumour its name.  Bland cytology & low mitotic activity Nuclei are small, uniform and hyperchromatic with inconspicuous nucleoli; mitoses are rare to absent. Confirms a benign process; carcinoma ex-pleomorphic adenoma would show nuclear atypia, mitoses and invasion beyond the capsule.  Together, this well-demarcated lesion with a dual epithelial-myoepithelial component set in a chondromyxoid matrix is diagnostic of a pleomorphic adenoma. |
| 13.14 | Diagnosis: Spindle cell myoepithelioma of the palate; Key Microscopic Features: Myoepithelioma made up of sheets of myoepithelial cells with no duct formation. Plasmacytoid myoepithelial cells exhibit abundant eosinophilic cytoplasm and eccentric nuclei. | Histopathological Analysis  Most likely diagnosis: Mucoepidermoid Carcinoma (MEC)  Justification Based on Microscopic Features  Low magnification image (1st image):  Lobulated architecture: The tumor appears as lobules separated by fibrous septa.  Cystic and solid areas: There are clear cystic spaces interspersed with more cellular solid areas, a hallmark of MEC.  Infiltrative margins: Suggests malignant behavior with tumor invading surrounding salivary gland tissue.  High magnification image (2nd image):  Cellular heterogeneity:  Mucin-producing cells: Cells with pale, foamy cytoplasm indicating mucin production.  Intermediate cells: Polygonal cells with moderate cytoplasm and round to oval nuclei.  Epidermoid (squamoid) cells: Cells with eosinophilic cytoplasm, often more polygonal or flattened.  No clear myoepithelial component, which helps distinguish MEC from other mixed tumors (like pleomorphic adenoma).  Summary of Key Histological Features:  Mixture of mucous, intermediate, and epidermoid cells.  Presence of cystic spaces and solid tumor nests.  Lobulated architecture with possible invasion into surrounding tissue.  Absence of chondromyxoid stroma (helps rule out pleomorphic adenoma). | Histopathologic diagnosis  Adenoid cystic carcinoma (ACC) – predominantly solid / cribriform pattern  Key microscopic features in the provided images  Magnification Observations Significance for ACC  Low-power overview • Lobulated, infiltrative mass with irregular borders.  • Numerous round-to-oval “pseudocystic” spaces filled with homogeneous eosinophilic (hyaline) material, producing a classic “Swiss-cheese” / cribriform appearance.  • Alternating solid nests and cribriform areas without chondromyxoid stroma. Cribriform architecture and hyaline basement-membrane material are hallmark features of ACC.  High-power • Uniform, small basaloid cells with hyperchromatic, angulated nuclei and scant cytoplasm arranged in tight cords, nests, and solid sheets.  • Narrow lumina or true ducts occasionally lined by a dual cell layer.  • Thick eosinophilic basement-membrane material outlining and filling pseudocysts.  • High nuclear-to-cytoplasm ratio; low mitotic activity typical of classic ACC. The basaloid cytology plus hyaline cylinders inside cribriform spaces distinguishes ACC from pleomorphic adenoma, basal cell adenoma, and polymorphous adenocarcinoma.  Perineural invasion—a characteristic feature of ACC—cannot be confirmed in the submitted frames, but the infiltrative growth pattern seen at low power supports the diagnosis.  Together, the basaloid cell population, cribriform/solid growth, and abundant hyaline basement-membrane material uniquely point to adenoid cystic carcinoma. |
| 13.14 | Diagnosis: Spindle cell myoepithelioma of the palate; Key Microscopic Features: Myoepithelioma consists of sheets of epithelioid cells infiltrating the capsule. | Based on the provided hematoxylin and eosin (H&E) stained histopathological images of a salivary gland lesion at low and higher magnification, the most likely diagnosis is:  Diagnosis: Mucoepidermoid Carcinoma (MEC)  Key Microscopic Features Supporting the Diagnosis:  Mixture of Cell Types:  Mucous cells (pale-staining, vacuolated cytoplasm): These are present in the images, especially at higher magnification.  Epidermoid (squamoid) cells: Cells with more eosinophilic (pink) cytoplasm and central nuclei, appearing more solid and cohesive.  Intermediate cells: Cells that have features in between mucous and epidermoid cells, relatively uniform with less cytoplasmic vacuolation.  Cystic and Solid Areas:  The low-power image shows a lobulated architecture with cystic spaces, a hallmark of low- to intermediate-grade MEC.  Areas of solid tumor nests interspersed with mucin-filled cystic spaces are characteristic.  Infiltrative Growth Pattern:  The lesion appears to infiltrate surrounding tissue, particularly evident at the interface with the fibrous capsule or stroma, which supports its malignant nature.  Mucin Pools:  Presence of extracellular mucin (light staining material within cystic spaces and intercellular areas), which is a defining feature of MEC.  Vascular Features and Hemorrhage:  Areas of hemorrhage and possible vascular invasion or congestion are seen, which can be associated with aggressive or intermediate behavior.  Conclusion:  The biphasic pattern of mucous and epidermoid cells in a cystic and solid architecture, along with infiltrative features, strongly supports a diagnosis of mucoepidermoid carcinoma, the most common malignant salivary gland neoplasm. This is particularly relevant in oral and maxillofacial pathology when examining tumors of minor salivary glands. | Most likely histopathological diagnosis  → Basal cell adenoma (solid / trabecular-membranous type) of a salivary gland  Microscopic hallmarks supporting this diagnosis  Level of magnification Recorded finding Diagnostic relevance  Low-power overview Sharply circumscribed, thinly encapsulated, lobulated mass with multiple fibrous septa; no infiltrative tongues into surrounding tissue A benign, well-demarcated growth pattern typical of basal cell adenoma (BCA) and unlike its malignant counterpart or adenoid cystic carcinoma  Low- & medium-power Tumour divided into solid and trabecular nests; some lobules show central cystic/degenerative spaces now filled with coagulated blood (pink lakes) Solid/trabecular architecture is one of the classical patterns of BCA; cystic haemorrhage is a common degenerative change and not a primary vascular lesion  High-power Nests built by uniform basaloid cells: small round/oval hyperchromatic nuclei, scant cytoplasm, peripheral palisading of nuclei, very little pleomorphism or mitotic activity Cytologic blandness with peripheral palisading distinguishes BCA from basal cell adenocarcinoma and adenoid cystic carcinoma  High-power Thick eosinophilic “membranous” (basement-membrane-like) material rimming and sometimes dissecting between the cell nests Characteristic of the membranous/solid variant of BCA and not seen in pleomorphic adenoma or mucoepidermoid carcinoma  Whole lesion Absence of chondromyxoid stroma, mucous cells, oncocytic epithelium or cribriform basophilic pseudocysts Helps exclude pleomorphic adenoma, Warthin tumour, mucoepidermoid carcinoma and adenoid cystic carcinoma, respectively  Synthesis  Together, the encapsulation, solid-trabecular lobules of bland basaloid cells with peripheral palisading, the intervening thick hyaline basement-membrane material, and secondary haemorrhagic cystic change form the classic histologic picture of a basal cell adenoma of salivary gland origin. |
| 13.15 | Diagnosis: Canalicular adenoma; Key Microscopic Features: Encapsulated tumor with rich hemorrhage and interconnected cellular strands. Delicate cell ribbons within myxochondroid stroma. Basaloid cells in single or double layers forming ductal structures and epithelial knots in collagen-poor mucinous stroma with ectatic vessels. | Based on the provided hematoxylin and eosin (H&E) stained histological images of a salivary gland neoplasm at different magnifications, the most likely diagnosis is:  Diagnosis: Acinic Cell Carcinoma (AcCC)  Key Microscopic Features Supporting the Diagnosis:  Overall Architecture (Low Magnification - Image 1):  Well-circumscribed, partially encapsulated mass.  Lobulated growth pattern.  Absence of significant invasion into surrounding tissue (suggests low-grade malignancy).  Intermediate Magnification (Image 2):  Solid and microcystic patterns with abundant granular basophilic cytoplasm.  Multiple small glandular and cyst-like spaces.  Scattered areas of hemorrhage and fibrous stroma are also evident.  High Magnification (Image 3):  Cells show abundant basophilic granular cytoplasm, which is characteristic of zymogen granules (PAS-positive, diastase-resistant granules in AcCC).  Round to oval nuclei with generally small nucleoli.  Some luminal formation is present, with apical snouting indicative of secretory activity.  Low-grade nuclear atypia; mitotic activity is low.  Summary:  The presence of basophilic granular cytoplasm in tumor cells (zymogen granules), microcystic and solid patterns, and low-grade cytologic features are diagnostic hallmarks of Acinic Cell Carcinoma, a malignant epithelial neoplasm of the salivary glands—most commonly arising in the parotid gland. | Histopathological diagnosis  → Acinic cell carcinoma (papillary-cystic / microcystic pattern)  Key microscopic features in the three fields that support this diagnosis  Field What can be seen Significance  Low-power overview (image 1) • Sharply demarcated, lobulated nodule surrounded by a thin fibrous capsule.  • Parenchyma made up of innumerable small cystic and microcystic spaces that give a lace-like or “honey-comb” cut surface.  • Papillary fronds project into many of the larger cystic spaces; focal intraluminal haemorrhage is present. A circumscribed, multicystic lesion with papillary projections is characteristic of the papillary-cystic variant of acinic cell carcinoma (ACC).  Intermediate magnification (image 2) • Fibrovascular papillae and delicate, complex tubulo-papillary structures line the cystic spaces.  • The lining epithelium is two- to three-cells thick, composed of uniform, darkly basophilic cells.  • Pools of extravasated erythrocytes and thin eosinophilic secretions lie in the lumina. The papillary-cystic architecture with serous-type cells and intraluminal haemorrhage/secretions is typical for ACC; other salivary tumours (e.g., adenoid cystic or secretory carcinoma) lack the combination of granular basophilic cytoplasm and this particular papillary-microcystic pattern.  High-power detail (image 3) • Tumour cells show abundant granular, strongly basophilic cytoplasm with well-defined borders—reflecting zymogen granules of serous acinar differentiation.  • Nuclei are round-to-oval, relatively uniform, occasionally eccentrically placed, with small nucleoli and low mitotic activity.  • Small true glandular lumina contain pale eosinophilic secretory material. The serous acinar cytology (basophilic, granular cytoplasm) is the hallmark of ACC and differentiates it from other low-grade salivary gland neoplasms. The bland nuclear features fit the usually low-grade behaviour of ACC.  Integrating these findings — a well-circumscribed salivary gland tumour composed of serous acinar-type cells forming microcystic and papillary-cystic structures, with granular basophilic cytoplasm and minimal atypia — leads to a single, best diagnosis of acinic cell carcinoma, papillary-cystic variant. |
| 13.15b | Diagnosis: Canalicular adenoma; Key Microscopic Features: Canalicular adenoma exhibits a tubular morphology. Irregular strands and tubular formations within myxochondroid stroma. Tumor cells form strands with central lumens, delicate vessels, and collagen-poor stroma. Tumor strands are multilayered with epithelial knots or beads at intersections. | Histopathological Analysis of Salivary Gland Lesion  Diagnosis:  Papillary Cystadenocarcinoma (a low-grade malignant salivary gland tumor)  Key Microscopic Features Observed:  Papillary Architecture:  The most prominent histological feature seen at medium and high magnifications is the presence of complex papillary fronds with fibrovascular cores, lined by columnar to cuboidal epithelial cells.  These papillary structures are numerous and branching, consistent with the diagnosis of a papillary neoplasm.  Cystic Spaces:  Multiple cystic areas filled with eosinophilic material or empty spaces are noted, especially at low and medium magnifications.  This aligns with the “cystadenocarcinoma” component of the diagnosis.  Cellular Atypia and Mitoses:  Mild nuclear atypia, occasional prominent nucleoli, and a moderate mitotic rate are evident at higher magnification.  These features support a malignant nature but are relatively low-grade, as is typical for papillary cystadenocarcinoma.  Infiltrative Growth Pattern:  Although generally circumscribed, there are areas suggesting infiltration into the surrounding stroma, which is characteristic of malignant behavior.  Lack of Myoepithelial Layer:  Absence of a consistent myoepithelial cell layer around glandular structures helps distinguish this from benign papillary cystadenomas or pleomorphic adenomas.  Summary:  The constellation of papillary structures, cystic areas, mild to moderate atypia, and infiltrative growth pattern supports the diagnosis of papillary cystadenocarcinoma, a rare but distinct low-grade malignant salivary gland tumor. The appearance is not consistent with more aggressive carcinomas (like mucoepidermoid or adenoid cystic carcinoma) or benign entities like Warthin tumor or pleomorphic adenoma. | Histopathologic diagnosis  → Acinic cell carcinoma (papillary-cystic / microcystic-solid variant) of salivary gland  Key microscopic features visible in the three H-E images  Image Low-magnification architecture Cytologic details Diagnostic relevance  1 Lobulated, partly solid / microcystic sheets separated by thin fibrous septa; no obvious capsule breach. Cells are uniform, basophilic and granular, creating a “blue-purple” appearance. Solid + microcystic pattern is one of the classic growth patterns of acinic cell carcinoma.  2 Numerous cystic spaces with delicate papillary and cribriform fronds projecting into the lumina (“papillary-cystic” configuration). Papillae have central fibro-vascular cores. The papillary-cystic architecture is highly characteristic of the papillary-cystic variant of acinic cell carcinoma.  3 High power of the papillae: back-to-back serous acinar-type cells line the fronds. • Polygonal cells with finely granular, amphophilic-to-basophilic cytoplasm (zymogen granules).  • Round, centrally placed nuclei, occasional small nucleoli, minimal pleomorphism, rare mitoses. Serous acinar differentiation (basophilic zymogen granules) together with low-grade cytology clinches the diagnosis of acinic cell carcinoma and rules out other papillary salivary lesions (e.g., salivary duct carcinoma, mucoepidermoid carcinoma).  Additional points supporting the diagnosis  Absence of intermediate/mucous cells (excludes muco-epidermoid carcinoma).  No biphasic ductal-clear-myoepithelial arrangement (excludes epithelial-myoepithelial carcinoma).  Lack of high-grade nuclear atypia or comedo-type necrosis (excludes salivary duct carcinoma).  Classic mixture of solid, microcystic and papillary-cystic areas—all hallmarks of acinic cell carcinoma.  Taken together, the constellation of serous acinar cytology plus the solid–microcystic–papillary-cystic architectural spectrum is diagnostic of acinic cell carcinoma, papillary-cystic variant. |
| 13.16 | Diagnosis: Canalicular adenoma; Key Microscopic Features: Tumor with cystic spaces and both myxoid and hyalinized stroma. Basaloid cells in interconnected strands arranged in a trabecular pattern within myxoid stroma. Cells form beads or knots where strands intersect. | Based on the provided hematoxylin and eosin (H&E) stained histopathology images of a lesion from the salivary gland neoplasms group, here is the analysis:  Most Likely Histopathological Diagnosis:  Pleomorphic Adenoma (also known as benign mixed tumor)  Key Microscopic Features Justifying the Diagnosis:  Biphasic cellular composition:  The images show both epithelial/myoepithelial and mesenchymal (stromal-like) components — a hallmark of pleomorphic adenoma.  The epithelial cells are arranged in duct-like, trabecular, and solid nests surrounded by myxoid or chondromyxoid stroma.  Myoepithelial-rich stroma:  The stroma varies from myxoid to chondroid appearance, evident by the pale, loose areas with a basophilic hue.  These regions resemble cartilaginous tissue and are quite characteristic of pleomorphic adenomas.  Well-circumscribed borders:  Particularly in the lower magnification image, the lesion appears well-demarcated from the surrounding salivary gland tissue, consistent with benign behavior.  Tubuloductal structures:  Tubular and duct-like structures lined by a dual layer of epithelial and myoepithelial cells are clearly visible, supporting the diagnosis.  Absence of significant atypia or mitotic activity:  The cells do not show cytological atypia, pleomorphism, or increased mitotic figures, ruling out malignancy.  Summary:  The combined presence of ductal epithelial elements, myoepithelial cells, and chondromyxoid stroma, along with the well-demarcated growth pattern, strongly supports the diagnosis of Pleomorphic Adenoma, the most common benign salivary gland neoplasm. | Most likely histopathological diagnosis – Adenoid Cystic Carcinoma (classic cribriform pattern)  Key microscopic features in the two H-E sections that point to this diagnosis  Field Salient findings Diagnostic significance  Architecture • Tumour islands and trabeculae show a cribriform or “Swiss-cheese” pattern – punched-out spaces of varying size within basaloid epithelial nests (best appreciated in the higher-power image). The cribriform pattern is the hallmark architectural configuration of adenoid cystic carcinoma (AdCC).  Cell morphology • Cells are uniformly basaloid: small, hyperchromatic, scant cytoplasm, inconspicuous nucleoli. Basaloid cytology distinguishes AdCC from pleomorphic adenoma (which shows plasmacytoid/myoepithelial cells) and from mucoepidermoid carcinoma (mixed cell types).  Luminal / pseudoluminal contents • Many punched-out spaces are filled with eosinophilic, glassy, basement-membrane-like (hyaline) material; others appear empty or dissected out during processing, giving a stellate/white appearance. Basement-membrane-rich pseudocysts are characteristic of AdCC and produced by neoplastic myoepithelial cells.  Stroma & invasion pattern • Tumour nests are separated by dense, hyalinised collagen.  • In the low-power field a broad tongue of neoplasm is seen infiltrating adjacent skeletal muscle fibres (pink, cross-striated). Infiltrative growth into skeletal muscle (and classically along nerves, even if not captured here) underscores the malignant nature of AdCC and helps separate it from circumscribed benign neoplasms.  Vascular/perineural features • Foci of red blood cells in clefts at the tumour–host interface suggest angiotropic spread; although perineural invasion is not in these cuts, its possibility should be actively sought on deeper levels. AdCC has a strong predilection for perineural and angiolymphatic invasion.  Taken together – a basaloid cell population arranged in a cribriform pattern with basement-membrane cylinders, set within hyalinised stroma and infiltrating muscle – best supports Adenoid Cystic Carcinoma, cribriform type. |
| 13.17 | Diagnosis: Canalicular adenoma; Key Microscopic Features: Cystic tumor showing a trabecular pattern and incipient tumor in an adjacent salivary gland. Incipient canalicular adenoma is also present in another lobule. | Based on the two provided hematoxylin and eosin (H&E) stained histopathology images of a salivary gland lesion at different magnifications, the most likely histopathological diagnosis is:  Pleomorphic Adenoma (Benign Mixed Tumor)  Key Microscopic Features Supporting This Diagnosis:  Mixture of Epithelial and Myoepithelial Cells:  Seen in both images, especially at higher magnification, are ductal epithelial structures interspersed with areas of myoepithelial cells.  The epithelial cells often form duct-like or tubular structures.  Chondromyxoid Stroma:  A characteristic feature of pleomorphic adenoma is the presence of a chondromyxoid (cartilage-like and mucoid) stroma, which is visible as a pale, basophilic background surrounding the epithelial islands in both images.  Well-Circumscribed Nature:  In the lower magnification image, the lesion appears well-circumscribed, with a pushing rather than infiltrative border — typical of a benign neoplasm.  Lobular Architecture with Variable Cellular Density:  The tumor shows a lobulated architecture with areas of varying cellularity, and regions with myxoid and hyalinized stroma.  The mixture of textures and cell densities gives the tumor its classic "pleomorphic" appearance.  Absence of Atypia or Invasive Growth:  No significant cytologic atypia or infiltrative growth pattern is evident, favoring a benign lesion over malignant neoplasms.  Conclusion:  The microscopic features — especially the biphasic cellular pattern (epithelial and myoepithelial cells), the presence of chondromyxoid stroma, and the overall circumscribed nature of the lesion — are classic for Pleomorphic Adenoma, the most common benign salivary gland tumor. | Histopathological diagnosis  ► Acinic cell carcinoma – microcystic / papillary-cystic pattern (low-grade)  Key microscopic features that point to this diagnosis  Field What is seen in your slides Why it fits acinic cell carcinoma  Overall architecture Lobulated tumour separated by delicate fibrous septa; tumour tongues project into large cystic spaces (right half of low-power slide) ACC is often lobulated and may show a papillary-cystic growth pattern, especially in minor glands  Predominant pattern Innumerable tiny, round to oval “micro-cysts” within the lobules, giving a Swiss-cheese / microcystic appearance (arrow) The microcystic / alveolar pattern is the classic variant of ACC  Cell morphology Cells lining the micro-cysts are uniform, polygonal, with finely granular to vacuolated, basophilic cytoplasm and centrally placed round nuclei Granular basophilic cytoplasm reflects serous-acinar (zymogen-granule) differentiation, a hallmark of ACC  Luminal contents Many cystic and duct-like spaces contain pale eosinophilic secretions; larger cysts show papillary epithelial folds Secretory material and papillary folds are typical in the papillary-cystic variant of ACC  Stroma / capsule No chondromyxoid matrix (rules out pleomorphic adenoma); no hyaline cylinders (rules out adenoid cystic carcinoma); minimal cytologic atypia and mitoses, supporting a low-grade malignant process Helps narrow the differential to ACC among salivary neoplasms  Together, the lobulated layout, microcystic “Swiss-cheese” pattern, serous-acinar cytology, and papillary-cystic spaces are diagnostic of acinic cell carcinoma (microcystic / papillary-cystic type). |
| 13.18 | Diagnosis: Cystadenoma; Key Microscopic Features: Cyst proliferation of various sizes with focal adenomatous plaques. Cysts are lined by a double layer of columnar cells with eosinophilic luminal cells. | Based on the histopathological features visible in the provided hematoxylin and eosin (H&E) stained images of a salivary gland lesion (low and high magnification), the most likely diagnosis is:  Pleomorphic Adenoma (Benign Mixed Tumor)  Key Microscopic Features Supporting the Diagnosis:  Biphasic Cellular Composition:  The lesion shows both epithelial/myoepithelial and stromal (mesenchymal-like) components, characteristic of pleomorphic adenoma.  In the high-power image, epithelial cells are forming duct-like structures lined by cuboidal to columnar cells with surrounding myoepithelial cells.  Chondromyxoid Stroma:  The low-power image reveals abundant stromal areas with a myxoid (lightly stained, gelatinous), and partially chondroid (cartilage-like) matrix—hallmark features of pleomorphic adenoma.  Well-Circumscribed Border:  The low-power image also demonstrates a well-demarcated and partially encapsulated lesion, another feature suggestive of pleomorphic adenoma.  Lack of Cellular Atypia or Invasion:  No evidence of cytological atypia or infiltrative growth pattern is seen, consistent with a benign process.  Summary:  Pleomorphic adenoma is the most common benign salivary gland neoplasm. The provided histologic images display its classic biphasic pattern—ductal epithelial and myoepithelial cells within a chondromyxoid stroma, supporting this diagnosis. | Histopathological diagnosis  Pleomorphic adenoma (benign mixed tumour) of a minor salivary gland  Key microscopic features supporting this diagnosis  Observed feature Diagnostic significance  Well-demarcated, lobulated nodule with a thin fibrous capsule immediately beneath an intact oral mucosal epithelium (low-power image) Fits the typical architecture of a benign, encapsulated salivary gland neoplasm such as pleomorphic adenoma.  Pronounced heterogeneity (“pleomorphism”) of tissue patterns – alternating solid, trabecular and cystic areas separated by fibrous septa Pleomorphic adenoma is characterised by architectural diversity created by its dual epithelial and myoepithelial components.  Abundant pale-eosinophilic to basophilic myxoid/chondroid stroma containing only scattered spindle or plasmacytoid cells Myoepithelial cells elaborate a rich chondromyxoid matrix, producing the cartilage-like appearance that is virtually pathognomonic for pleomorphic adenoma.  True duct-like structures lined by a double cell layer – an inner cuboidal/columnar epithelial layer and an outer myoepithelial layer (high-power image) The biphasic cell population is a defining microscopic hallmark of pleomorphic adenoma.  Papillary projections of the duct lining into cystic lumina without cytologic atypia or significant mitotic activity Confirms benign behaviour and helps distinguish the tumour from papillary or cystic malignant salivary neoplasms.  The combination of an encapsulated, lobulated mass showing a mixture of epithelial ducts and myoepithelial cells suspended in a myxoid-to-chondroid matrix is classic for pleomorphic adenoma and effectively rules out other salivary gland tumours. |
| 13.19 | Diagnosis: Cystadenoma; Key Microscopic Features: Large single cyst with adenomatous epithelium forming plaques and undulations. Adenomatous epithelium forms small ducts. | Based on the histopathological analysis of the provided hematoxylin-and-eosin (H&E) stained images of a salivary gland lesion at low and high magnification, the single most likely diagnosis is:  Canalicular Adenoma  Justification and Key Microscopic Features:  Low Magnification (Image 1):  Architecture: The lesion appears well-circumscribed, with cord-like and branching canal-like structures that are parallel and anastomosing, often running in a single file pattern.  Stroma: The background shows a loose, vascular stroma, a hallmark of canalicular adenoma, often seen in the upper lip.  No encapsulation but the lesion is well-demarcated from the surrounding tissue.  High Magnification (Image 2):  Cellular Features: The epithelium consists of uniform, columnar to cuboidal cells with bland, darkly staining nuclei, arranged in single or double rows forming narrow, canal-like structures.  Lumina Formation: Presence of small luminal spaces, some of which contain mucinous or eosinophilic material.  Absence of atypia: No signs of cytologic atypia or mitotic activity.  Myoepithelial cells are inconspicuous or absent, distinguishing it from pleomorphic adenoma.  Conclusion:  The combination of a well-circumscribed lesion with characteristic canal-like epithelial structures in a loose vascular stroma, and the lack of pleomorphism or chondromyxoid stroma, is diagnostic for canalicular adenoma, a benign salivary gland neoplasm commonly affecting the upper lip of older adults. | Histopathological diagnosis  → Low-grade mucoepidermoid carcinoma (MEC) of a minor salivary gland.  Key microscopic features supporting this diagnosis  Feature seen in the photomicrographs Diagnostic significance  Predominantly cystic architecture with delicate papillary/undulating infoldings (clearly appreciable on the low-power view) Low-grade MECs are characteristically > 50 % cystic and often show papillary projections into the lumina.  Mixed epithelial cell population in the lining and solid nests –  • Mucous cells with abundant foamy/vacuolated cytoplasm (appear as round “empty” spaces)  • Intermediate cells with basaloid nuclei and scant cytoplasm  • Occasional squamoid/epidermoid cells The admixture of mucous-producing, intermediate and epidermoid elements is the defining triad of MEC. Presence of obvious mucous cells rules out purely squamous or oncocytic tumours.  Minimal cytologic atypia, low mitotic activity Fits a low-grade lesion rather than an intermediate/high-grade MEC or a high-grade carcinoma.  Fibro-collagenous stroma without chondromyxoid (pleomorphic adenoma) or lymphoid (Warthin) tissue Helps exclude other cystic–papillary salivary tumours.  Taken together, the cystic-papillary architecture coupled with the characteristic triphasic cell population strongly points to low-grade mucoepidermoid carcinoma. |
| 13.20 | Diagnosis: Papillary Oncocytic Cystadenoma; Key Microscopic Features: A large cyst with papillary and adenomatous proliferation of the lining, featuring delicate fibrovascular cores. | Histopathological Diagnosis:  Warthin Tumor (also known as Papillary Cystadenoma Lymphomatosum)  Key Microscopic Features Supporting the Diagnosis:  Cystic and Papillary Architecture (visible at low and medium magnification):  The tumor shows multiple cystic spaces with papillary infoldings projecting into the cystic lumina.  These papillae are lined by a bilayered epithelium, a hallmark of Warthin tumor.  Bilayered Epithelial Lining (seen clearly at higher magnification):  Inner luminal layer: Tall columnar epithelial cells with eosinophilic, granular cytoplasm (oncocytic appearance).  Outer basal cell layer: Smaller, cuboidal cells with darker nuclei.  The oncocytic appearance is due to abundant mitochondria.  Lymphoid Stroma (best seen in low and medium magnification):  Dense lymphoid tissue with germinal centers underlies the epithelial component, characteristic of this tumor.  This lymphoid component gives the tumor its "lymphomatosum" designation.  Lack of Cytologic Atypia or Invasion:  No evidence of dysplasia, necrosis, or infiltrative growth is seen, consistent with the benign nature of Warthin tumor.  Additional Context:  Warthin tumors most frequently arise in the parotid gland.  They are often bilateral or multifocal, particularly in smokers.  The presence of both epithelial and lymphoid components is diagnostic. | Histopathological diagnosis  → Intraductal papilloma (salivary duct papilloma) — a benign, solitary duct-type papillary lesion of salivary glands.  Key microscopic features supporting the diagnosis  Magnification Observations Diagnostic relevance  Low-power (whole mount) • Well-circumscribed, unilocular cystic space  • Numerous arborising papillary fronds project into the lumen, each with a central fibro-vascular core Typical architecture of an intraductal (duct-confined) papilloma rather than a multilocular papillary cystadenoma.  Intermediate power • Papillae lined by a bilayered epithelium:    – Inner luminal columnar/cuboidal secretory cells with eosinophilic–vacuo­lated cytoplasm and occasional apical (“snouting”) secretion    – Outer flattened to low-cuboidal myoepithelial/basal cell layer along the basement membrane  • Delicate vascular cores with scant stroma; no lymphoid tissue The two-cell layer is the hallmark of duct-type papillomas; absence of lymphoid stroma rules out Warthin tumour.  High power • Uniform, bland nuclei; fine chromatin, inconspicuous nucleoli, rare/absent mitoses  • No cribriform pattern, no infiltrative edges, no zymogen granules Cytologic blandness and lack of invasion confirm benign nature and exclude low-grade intraductal carcinoma, acinic-cell, or mucoepidermoid carcinoma.  Summary of defining features  Solitary, cystically dilated duct with complex intraluminal papillae.  Papillae have delicate fibrovascular cores and are covered by a double epithelial layer.  Cells show secretory/apocrine change but no significant atypia or invasion.  No lymphoid stroma, oncocytic sheets, or multilocular architecture that would suggest other papillary salivary tumours.  Together, these findings firmly favour intraductal papilloma as the single best diagnosis for the lesion shown. |
| 13.21 | Diagnosis: Oncocytic Metaplasia from Chronic Obstruction of Glands; Key Microscopic Features: Salivary glands show dilated ducts within lobules, lined by oncocytes and accompanied by plasma cells; adjacent glands show early changes. | Based on the provided hematoxylin-and-eosin stained histological images of a salivary gland lesion at low and high magnification, the most likely histopathological diagnosis is:  Pleomorphic Adenoma (Benign Mixed Tumor)  Key Microscopic Features Justifying the Diagnosis:  1. Biphasic Cellular Composition:  Epithelial and myoepithelial cells are clearly observed.  Duct-like structures lined by cuboidal epithelial cells are surrounded by a second layer of myoepithelial cells.  2. Chondromyxoid Stroma:  The stroma shows areas that are myxoid (lightly stained, bluish), chondroid-like (cartilage-like), and fibrous, which is characteristic of pleomorphic adenoma.  This diverse stromal morphology is a hallmark of the “pleomorphic” nature.  3. Well-Circumscribed and Lobulated Architecture:  The lesion appears well-demarcated from the surrounding tissue, with lobulated growth, suggesting a benign nature.  Multiple lobules are separated by fibrous septa.  4. Ductal Structures:  Numerous duct-like and tubular structures are present, composed of inner epithelial and outer myoepithelial cells, often forming small tubules or cyst-like spaces.  5. Lack of Cellular Atypia or Mitotic Figures:  At high power, the cells do not show signs of malignancy—no marked atypia, pleomorphism, or high mitotic activity is observed. | Histopathological diagnosis – Polymorphous adenocarcinoma  (formerly called polymorphous low-grade adenocarcinoma, PLGA)  Key microscopic features visible in the two H-E sections  Field What is seen Diagnostic significance  Overall architecture (low-power image) • Lobulated mass composed of inter-anastomosing papillae, tubules and cystic spaces in a dense fibrohyaline stroma.  • Lesion is fairly circumscribed yet shows subtle irregular infiltration at the periphery. Simultaneous presence of several growth patterns in one tumour (“polymorphous” architecture) is a hallmark of polymorphous adenocarcinoma.  Epithelial component (high-power image) • Tubules/papillae are lined by a single row of uniform cuboidal-to-columnar cells with pale to eosinophilic cytoplasm and round, bland nuclei.  • Minimal pleomorphism, mitoses scarce; no necrosis. Cytologic blandness despite architectural complexity typifies this tumour and helps separate it from higher-grade salivary carcinomas (e.g., adenoid cystic, salivary duct carcinoma).  Pattern diversity • In one field you can appreciate papillary-cystic, tubular and solid/whorled areas lying side-by-side.  • A characteristic “swirling/targetoid” arrangement of tumour cords around stromal bundles is evident. PLGA characteristically exhibits many patterns (tubular, cribriform-solid, papillary, single-file/“Indian-file”, targetoid) within the same lesion.  Stromal relationship • Tumour islands and cords are embedded in and sometimes appear to spiral through a densely hyalinised collagenous stroma. The intimate intertwining of uniform cells with a sclerotic stroma without the myxoid/chondroid matrix of pleomorphic adenoma is typical.  Perineural/single-file infiltration • In several foci small cords of identical cells track through the stroma in Indian-file fashion (early perineural tendency). Perineural or neurotropic growth in a concentric “target-like” pattern is common in polymorphous adenocarcinoma and supports the diagnosis.  Taken together, the combination of bland, uniform salivary epithelial cells and a strikingly polymorphous architectural pattern (papillary-cystic, tubular, cribriform/solid and swirling) in a hyalinised stroma, with low mitotic activity and incipient single-file/perineural spread, is diagnostic of polymorphous adenocarcinoma of minor salivary glands. |
| 13.22 | Diagnosis: Sclerosing Polycystic Adenosis; Key Microscopic Features: Dilated ducts with tubuloacinar hyperplasia, inflammation, and fibrosis, presenting cribriform, microcystic, and adenomatous patterns, with mucous spillage and foamy macrophages. | Histopathological Diagnosis:  Pleomorphic Adenoma (Benign Mixed Tumor)  Key Microscopic Features Supporting the Diagnosis:  Epithelial and Myoepithelial Components:  The images show duct-like structures lined by epithelial cells.  Surrounding these ducts, myoepithelial cells are seen in various forms—spindle-shaped, plasmacytoid (hyaline), or clear cells—typical of pleomorphic adenoma.  Chondromyxoid and Myxoid Stroma:  Prominent myxoid and chondroid areas are visible, especially in the low-power image, where these stroma types appear pale and basophilic.  This stroma is a hallmark of pleomorphic adenoma and indicates mesenchymal differentiation.  Encapsulation:  The lesion appears well-circumscribed and partially encapsulated, as seen in the low-power field, which is typical for benign salivary gland tumors like pleomorphic adenoma.  Architectural Diversity:  The tumor shows heterogeneous architecture, with areas of solid nests, tubules, and cyst-like spaces.  This pleomorphism (variety) in tissue patterns is the origin of the name pleomorphic adenoma.  Absence of Cytologic Atypia or Mitosis:  There are no significant mitotic figures or cytologic atypia, further supporting a benign lesion. | Histopathological diagnosis  Muco-epidermoid carcinoma (intermediate-grade) of a salivary gland.  Key microscopic features visible in the four H&E fields that support this diagnosis  Image Salient findings How this points to muco-epidermoid carcinoma  1 — low-power overview Lobulated, well-demarcated mass divided by fibrous septa; numerous cystic / duct-like spaces of very unequal size containing pale eosinophilic material Multicystic architecture is classic for low- to intermediate-grade MEC; other common malignant S-gland tumours (e.g., adenoid-cystic, acinic cell) do not show such prominent, mucus-filled cysts at low power  2 — medium power Each cyst/duct is lined by a mixed epithelium:  • pale vacuolated cells  • basaloid to amphophilic “intermediate” cells  • occasional eosinophilic squamoid cells;  solid nests of basaloid cells between cysts MEC is defined by the coexistence of mucous cells, epidermoid (squamoid) cells and intermediate cells arranged in cystic and solid areas  3 — high power (left half) Cystic islands composed predominantly of pale, foamy mucous cells; adjacent nests of darker intermediate cells; scant keratin formation Abundant mucous cells plus intermediate cells favour MEC and exclude purely acinar or purely squamous lesions  3 & 4 — high power (right halves) • Foci of intermediate / epidermoid cells with moderate eosinophilic cytoplasm and intercellular bridges  • Only mild nuclear pleomorphism and low mitotic activity An admixture of epidermoid elements without marked atypia indicates an intermediate-grade MEC (high-grade lesions are predominantly squamoid with high mitotic activity)  4 – ductal detail Small ducts with mucus plugs or mucin lakes and surrounding intermediate cells; absence of chondromyxoid stroma Helps separate MEC from pleomorphic adenoma or carcinoma ex-pleomorphic adenoma  Composite interpretation  The triphasic cell population (mucous + epidermoid + intermediate) is pathognomonic.  Cystic change filled with mucin, highlighted even at scanning magnification, is typical of low/intermediate grades.  Lack of cribriform hyaline “Swiss-cheese” pattern (adenoid-cystic) or granular basophilic serous acinar cytoplasm (acinic cell carcinoma) helps exclude those entities.  Taken together, these findings most convincingly fit an intermediate-grade muco-epidermoid carcinoma of salivary gland origin. |
| 13.23 | Diagnosis: Sialolipoma; Key Microscopic Features: A discrete tumor of mature adipocytes with benign adenomatous hyperplasia and rare mucous acini, containing numerous small nerve fibers. | Based on the histopathological features seen in the provided hematoxylin and eosin (H&E) stained images at different magnifications, the single most likely diagnosis is:  Pleomorphic Adenoma (Benign Mixed Tumor)  Key Microscopic Features Supporting the Diagnosis:  Biphasic Cell Population:  There is a characteristic mix of epithelial/myoepithelial cells and mesenchymal (stromal-like) components, which is the hallmark of pleomorphic adenoma.  The epithelial component forms duct-like and tubular structures, often lined by a double layer of cells.  Myoepithelial cells may appear plasmacytoid or spindled and are dispersed throughout.  Chondromyxoid Stroma:  The stroma in several areas appears myxoid and chondroid-like, a key diagnostic feature of pleomorphic adenoma.  This “mesenchymal” background gives a heterogeneous appearance, typical of pleomorphic adenoma.  Lack of Cytologic Atypia or Mitoses:  The cells do not show significant pleomorphism, mitotic activity, or necrosis, supporting a benign lesion.  Well-Circumscribed Borders:  The tumor appears to be well circumscribed and partially encapsulated (as seen in the low magnification image), though not always fully encapsulated in minor salivary glands.  These features are diagnostic of pleomorphic adenoma, the most common benign salivary gland tumor, often occurring in the parotid gland but also found in minor salivary glands (e.g., palate). | Most likely histopathological diagnosis  Polymorphous adenocarcinoma ( formerly called polymorphous low-grade adenocarcinoma) of a minor salivary gland.  Key microscopic clues visible in the two H-E fields  Feature seen How it points to polymorphous adenocarcinoma  Lobulated but un-encapsulated tumour infiltrating directly into surrounding fibroadipose tissue (white adipocyte vacuoles scattered throughout the lesion) Characteristic of a minor-gland tumour that grows with deceptively “pushing” lobules yet permeates native fat and soft tissue.  Architectural polymorphism inside the same lesion – small round to oval tubules/ducts, short cords, focal cribriform/“swiss-cheese” micro-cysts, and occasional solid nests The mixture of several low-grade patterns in one tumour is the hallmark of polymorphous adenocarcinoma.  Each duct is lined by a single layer of uniform, bland cuboidal cells; no myoepithelial outer rim is present Distinguishes it from biphasic tumours such as epithelial–myoepithelial carcinoma or basal-cell adenoma.  Cells are cytologically bland – round/oval nuclei, fine chromatin, inconspicuous nucleoli, very low mitotic activity Fits the “low-grade” cytology expected for this entity.  Dense, eosinophilic fibrous stroma separating tumour islands, with focal whorling/“target-like” arrangement around stromal strands (perineural space not caught in this field but the concentric swirl is evident) The “targetoid” pattern caused by tumour streaming around neurovascular bundles is another classic feature.  Absence of chondromyxoid (pleomorphic adenoma), muco-epidermoid mixture (mucoepidermoid carcinoma) or thick hyaline basement-membrane rims (basal-cell tumours) Helps exclude the main histologic mimics.  Together, these findings – an infiltrative, polymorphous but cytologically bland minor-salivary-gland tumour showing tubular/cribriform/solid patterns and permeation of surrounding fat – are diagnostic of polymorphous adenocarcinoma. |
| 13.25 | Diagnosis: Mucoepidermoid Carcinoma (Intermediate to High Grade); Key Microscopic Features: Infiltrative tumor with cystic and solid areas, containing mucous and intermediate cells, ductal structures, and invasive atypical epidermoid cells with desmoplasia. | Based on the provided hematoxylin and eosin (H&E) stained histological images at varying magnifications, the single most likely histopathological diagnosis is:  Mucoepidermoid Carcinoma (MEC)  Key Microscopic Features Supporting the Diagnosis:  Mixed Cell Population:  The hallmark of mucoepidermoid carcinoma is a mix of mucous cells, epidermoid (squamoid) cells, and intermediate cells, all of which are identifiable in the higher magnification images.  The clear cytoplasm and bubbly appearance of mucous cells are notable in the images.  Cystic and Solid Architecture:  The low-power image shows cystic spaces interspersed with solid nests of tumor cells, a common feature in low to intermediate-grade MEC.  There are areas of cyst formation and infiltrative solid growth, which are characteristic of this malignancy.  Mucin Production:  Presence of intracytoplasmic mucin in many cells (seen as vacuolated or bubbly cytoplasm), which can be confirmed with mucicarmine or PAS stains in practice.  Cellular Atypia and Mitoses:  Intermediate to high magnification reveals pleomorphic nuclei, occasional mitotic figures, and prominent nucleoli, particularly in intermediate cells.  These features suggest at least intermediate-grade MEC.  Perineural Invasion:  In the image with the nerve bundle surrounded by tumor (last image), perineural invasion is observed, which is a marker of malignancy and common in MEC.  Summary:  The lesion displays characteristic features of mucoepidermoid carcinoma, the most common malignant salivary gland neoplasm. The presence of diverse cell types (mucous, squamous, and intermediate), cystic and solid patterns, mucin production, and perineural invasion support this diagnosis. | Histopathological diagnosis  → Adenoid cystic carcinoma (ACC), predominantly cribriform/solid pattern  Key microscopic features visible in the panels  Field Salient findings How they support ACC  Low-power overview Infiltrative, un-encapsulated mass beneath intact mucosa; multiple ragged tongues and nests permeating the salivary gland parenchyma and adjacent soft tissue. Classic infiltrative growth seen in ACC; lack of a capsule helps distinguish it from benign mixed tumours.  Intermediate magnification Sheets and nests of small basaloid cells with scant cytoplasm; interspersed true glands and numerous “empty” or eosinophilic pseudocystic spaces, giving a Swiss-cheese / cribriform architecture. The cribriform (“honey-comb”) arrangement with basophilic basaloid cells surrounding hyaline globules is the signature pattern of ACC.  Tubular/cribriform focus Round to oval spaces packed with dense, glassy basement-membrane-type hyaline material encircled by two concentric cell layers (inner ductal, outer myoepithelial). Hyaline cylinders and dual cell layers are diagnostic hallmarks of ACC and separate it from mucoepidermoid carcinoma or polymorphous adenocarcinoma.  Solid areas Compact sheets of basaloid cells showing increased nuclear : cytoplasm ratio, occasional comedonecrosis and focal clear-cell change. Solid pattern is a recognised high-grade variant of ACC; presence of basaloid cytology and transition to cribriform/tubular areas confirms lineage.  Perineural involvement (“targetoid” pattern) Concentric whorling of tumour cells around a nerve bundle with surrounding hyalinised stroma. ACC is notorious for early, sometimes extensive perineural invasion; this feature is virtually pathognomonic in the salivary-gland setting.  Ancillary clues (seen across the panels)  Uniform, hyperchromatic nuclei with angular contours  Scant mitoses but considerable nuclear crowding  Hyalinised stromal reaction surrounding tumour islands  Taken together, the combination of cribriform (“Swiss-cheese”) architecture with hyaline cylinders, dual-layered tubules, solid basaloid fields and striking perineural invasion firmly establishes the lesion as adenoid cystic carcinoma. |
| 13.26 | Diagnosis: Mucoepidermoid Carcinoma; Key Microscopic Features: The tumor forms large cysts with proliferation of mucous, epidermoid, and intermediate cells, along with stromal invasion. | Based on the provided hematoxylin and eosin (H&E) stained histopathology images of a salivary gland neoplasm, observed at low and high magnification, the most likely single histopathological diagnosis is:  Adenoid Cystic Carcinoma (ACC)  Key Microscopic Features Supporting the Diagnosis:  Low Magnification (Image 1):  Cribriform Pattern: Multiple nests of basaloid epithelial cells with intervening pseudocystic spaces, giving a “Swiss cheese” appearance.  Hyalinized Stroma: The pseudocystic spaces appear filled with eosinophilic hyaline material (likely basement membrane-type material).  Infiltrative Growth: Ill-defined, infiltrative borders within the salivary gland parenchyma, which is common in ACC.  High Magnification (Image 2):  Basaloid Cells: Small, uniform, darkly stained (hyperchromatic) cells with scant cytoplasm.  Prominent Nucleoli: Some cells show prominent nucleoli, characteristic of malignant behavior.  True Glandular and Pseudocystic Spaces: Lumen-like spaces are filled with basophilic or eosinophilic material, corresponding to glycosaminoglycan or basement membrane-like substances.  Perineural Invasion (not clearly shown here but is a hallmark feature): Though not visible in these fields, ACC is notorious for invading nerves.  Summary:  The combination of the cribriform architectural pattern, basaloid tumor cells, and pseudocystic lumina with hyaline material is diagnostic of Adenoid Cystic Carcinoma, a malignant neoplasm of salivary gland origin known for slow growth but aggressive local invasion and high propensity for perineural invasion. | Histopathological diagnosis  → Acinic (serous acinar) cell carcinoma of salivary gland – papillary-/microcystic pattern  Microscopic features supporting the diagnosis  Field Observations Diagnostic significance  Low-power architecture (first image) • Tumour is lobulated and partly circumscribed but lacks a true capsule.  • Numerous round to oval cystic/microcystic spaces of variable size; several are large with smooth, thickened walls and are filled with homogeneous pale-eosinophilic secretions. Papillary-cystic / microcystic growth is classic for acinic cell carcinoma (ACC); large cysts with proteinaceous material rule out most other benign salivary tumours.  Tumour cell arrangement • Between the cysts, solid and nested lobules of cells are separated by delicate fibrous septa. The mixture of cystic and solid nests is typical for ACC.  Cellular cytology (high-power, second image) • Cells are polygonal with abundant finely granular, basophilic to amphophilic cytoplasm (zymogen granules).  • Nuclei are round, centrally to eccentrically placed, with small nucleoli and only mild pleomorphism. Granular basophilic cytoplasm representing serous-acinar differentiation is the single most distinctive microscopic hallmark of ACC.  Luminal/secretory features • Foci of small true glandular lumina and microcysts containing eosinophilic secretions are present within cell nests. Secretory material and true acinar differentiation favour ACC over mucoepidermoid carcinoma or secretory carcinoma.  Stroma • Predominantly fibrous with scattered capillaries and focal haemorrhage; no chondromyxoid or myoepithelial-rich stroma is seen. Absence of chondromyxoid matrix helps exclude pleomorphic adenoma.  Cytologic atypia & mitoses • Minimal atypia, infrequent mitoses – consistent with a low-grade malignant neoplasm such as ACC.  These combined architectural and cytologic findings – especially the basophilic granular (zymogen-rich) cytoplasm of the tumour cells and the mixed solid-microcystic/papillary-cystic architecture – are diagnostic of acinic cell carcinoma of the salivary gland. |
| 13.27a | Diagnosis: Mucoepidermoid Carcinoma; Key Microscopic Features: Tumor with solid and cystic regions, composed of intermediate, epidermoid, and clear cells with rare mucocytes, and a sclerosing pattern with ductal structures and prominent sclerosis. | Based on the provided hematoxylin and eosin (H&E) stained images of a salivary gland lesion at various magnifications, the most likely histopathological diagnosis is:  Mucoepidermoid Carcinoma (MEC)  🔍 Key Microscopic Features Supporting This Diagnosis:  Mixed Cell Population:  Mucous cells: Seen as pale, vacuolated cells with foamy cytoplasm (best appreciated in the second image at higher magnification).  Epidermoid (squamoid) cells: Polygonal cells with eosinophilic cytoplasm and sometimes intercellular bridges.  Intermediate cells: Less differentiated cells, often difficult to classify clearly into mucous or epidermoid.  Cystic and Solid Areas:  The low-power images (especially the first and third) demonstrate cystic spaces intermixed with solid nests of tumor cells, a hallmark of MEC.  Cystic spaces often contain mucin and appear to be lined by mucous or epidermoid cells.  Infiltrative Growth Pattern:  Tumor cells infiltrate surrounding stroma irregularly (evident in images 3–5), consistent with a malignant neoplasm.  Variable Differentiation:  The presence of areas showing greater cytologic atypia and more solid growth suggests this is at least a moderate-grade MEC.  Mucin Pools:  Mucin appears in extracellular pools and within vacuolated mucous cells (image 2), highlighted by the clear and pale areas.  🔬 Summary:  These features — especially the triphasic cellular composition, cystic and solid architecture, and mucin production — are diagnostic of Mucoepidermoid Carcinoma, the most common malignant salivary gland tumor.  If needed, further confirmation with mucicarmine or PAS staining (to highlight mucin) and immunohistochemistry (e.g., CK7, p63) could support the diagnosis, but the H&E features are already strongly characteristic. | Histopathological diagnosis  → Adenoid cystic carcinoma (ACC)  Key microscopic features supporting this diagnosis  Field Findings in the provided slides Diagnostic significance  Architecture • Mixture of patterns within the same tumour — cribriform (“Swiss-cheese”), tubular and solid nodules separated by fibrous septa (Images 1, 3-5). A hallmark of ACC is its heterogenous growth pattern; most tumours show two or more of these architectures.  Cribriform pattern • Round/oval nests perforated by many uniform pseudocystic spaces filled with eosinophilic‐hyaline (basement-membrane–type) material (Images 3-5). The classic and most common pattern of ACC.  Tubular pattern • Small true ducts/tubules lined by an inner epithelial and an outer myoepithelial cell layer set in fibrous stroma (Image 1). Characteristic dual‐cell tubular structures point strongly to ACC.  Solid pattern • Sheets of basaloid cells with scant cytoplasm, hyperchromatic angulated nuclei; only occasional microcysts (Image 2). Solid areas, when present, still maintain basaloid cytology typical of ACC and indicate a potentially higher-grade component.  Cytology • Uniform basaloid cells, high nuclear-to-cytoplasm ratio, inconspicuous nucleoli; rare mitoses. • No overt squamous differentiation or abundant mucocytes. Distinguishes ACC from mucoepidermoid carcinoma (which shows squamoid and mucous cells) and from pleomorphic adenoma (which shows plasmacytoid or chondromyxoid stroma).  Stroma & invasion • Dense, hyalinised/desmoplastic stroma encasing tumour islands; lobulated and infiltrative borders. • Although not in the planes shown, ACC typically shows “targetoid” perineural invasion. Desmoplastic reaction and propensity for neural tracking are classic for ACC.  Taken together, the combination of cribriform pseudocystic nests with hyaline cylinders, tubular dual-cell ducts, basaloid-solid foci, and a desmoplastic infiltrative growth pattern is diagnostic of adenoid cystic carcinoma of salivary gland origin. |
| 13.27b | Diagnosis: Oncocytic Mucoepidermoid Carcinoma; Key Microscopic Features: Cystic spaces lined by epidermoid, mucous, and oncocytic cells, with oncocytic cells showing hyperchromatic nuclei and typical mucoepidermoid carcinoma at the base. | Based on the provided hematoxylin and eosin (H&E) stained histological images of a salivary gland neoplasm, observed at increasing magnifications, the single most likely histopathological diagnosis is:  Mucoepidermoid Carcinoma (MEC)  Key Microscopic Features Justifying the Diagnosis:  Mixed Cell Populations:  The high-power image (third image) clearly shows a combination of mucous cells (with abundant foamy, pale cytoplasm), epidermoid (squamoid) cells (with eosinophilic cytoplasm and more prominent nucleoli), and intermediate cells (small to medium-sized cells with less cytoplasm).  This is a hallmark feature of mucoepidermoid carcinoma.  Cystic and Solid Architecture:  Low- and intermediate-power images (first and second images) show cystic spaces along with solid nests and cords of tumor cells infiltrating the surrounding stroma.  These cystic areas often contain mucin, supporting the diagnosis of a low- to intermediate-grade MEC.  Mucin Production:  Evidence of mucin-secreting cells, seen clearly in the higher magnification, further supports MEC. These are often positive with special stains like mucicarmine (not shown here, but the morphology is consistent).  Infiltrative Growth Pattern:  The lesion shows irregular, infiltrative borders invading adjacent stroma, suggestive of malignancy rather than a benign entity like pleomorphic adenoma.  Differential Diagnoses Considered:  Pleomorphic adenoma: Ruled out due to absence of chondromyxoid stroma and lack of the characteristic "mixed tumor" histology.  Adenoid cystic carcinoma: Typically shows cribriform/“Swiss cheese” pattern and lacks mucous cells.  Acinic cell carcinoma: Lacks the mucous and epidermoid cell types seen here.  Conclusion:  The histologic pattern observed in these images — cystic and solid areas with a mix of mucous, epidermoid, and intermediate cells — is classic for mucoepidermoid carcinoma, the most common malignant salivary gland tumor. | Most likely histopathological diagnosis  → Low-grade Mucoepidermoid Carcinoma (predominantly papillary-cystic pattern)  Key microscopic features in the provided H-E sections that support this diagnosis  Field Observation Significance  Overall architecture (low-power) Multifocal, expansile-but-infiltrative lesion showing numerous variably-sized cystic / duct-like spaces; many are partially or completely filled with eosinophilic or pale mucinous material. Papillary fronds protrude into several lumina. Dominant cystic component with intraluminal papillae is characteristic of low-grade mucoepidermoid carcinoma. Benign cystadenomas lack stromal invasion, while higher-grade MECs are more solid.  Interface with adjacent tissue Irregular tongues and cords of tumour penetrate fibrous tissue and skeletal muscle fibres seen at the right edge of the medium-power image. Confirms true invasion, excluding a purely intraductal papilloma or cystadenoma.  Epithelial cell populations (high-power) • Mucous cells – large polygonal cells containing optically clear to foamy intra-cytoplasmic mucin vacuoles, some with a signet-ring appearance.  • Intermediate cells – smaller polygonal cells with moderately eosinophilic, vacuolated cytoplasm and round nuclei.  • Occasional epidermoid/squamoid cells with more dense eosinophilic cytoplasm (seen lining papillae). A mixture of mucous, intermediate and squamoid cells is the defining cytologic triad of mucoepidermoid carcinoma. Predominance of mucous and cystic architecture points to the low-grade end of the spectrum.  Cyst / duct lining pattern Papillary fronds and cyst walls are frequently bilayered, with an inner columnar layer and outer flatter/basal cells. Fits the papillary-cystic variant of low-grade MEC; Warthin tumour would show dense lymphoid stroma and purely oncocytic bilayer, which are not present here.  Stroma & additional findings Fibro-hyaline stroma with scattered lymphoid aggregates but no chondromyxoid, myxochondroid or chondro-osseous matrix. No pronounced oncocytic granular cytoplasm. Low mitotic index and minimal nuclear pleomorphism. Absence of pleomorphic adenoma stroma argues against carcinoma-ex-pleomorphic adenoma; lack of pervasive oncocytic change and presence of the mucous cell population argue against Warthin tumour or oncocytoma. Bland cytology is compatible with low-grade MEC.  Taken together, the cystic architecture with intraluminal papillae, invasion into surrounding tissue, and the classic mixture of mucous, intermediate and occasional squamoid cells most convincingly indicate a low-grade mucoepidermoid carcinoma of salivary-gland origin. |
| 13.29 | Diagnosis: Adenoid Cystic Carcinoma (Classic Pattern); Key Microscopic Features: Prominent cribriform pattern with eosinophilic cuboidal cells in true ducts and myoepithelial cells surrounding pseudoducts filled with glycosaminoglycans. | Based on the provided hematoxylin and eosin (H&E) stained histopathology images of a salivary gland lesion at various magnifications, the most likely histopathological diagnosis is:  Pleomorphic Adenoma (Benign Mixed Tumor)  Key Microscopic Features Supporting the Diagnosis:  Biphasic Cellular Composition:  Epithelial and Myoepithelial Cells: Seen forming duct-like structures and strands, often lined by a dual cell layer.  Myoepithelial cells may appear plasmacytoid, spindle-shaped, or angular.  Chondromyxoid Stromal Background:  Abundant pale, myxoid to chondroid matrix is present throughout the lesion, which is a hallmark feature of pleomorphic adenoma.  This matrix is seen separating islands and strands of epithelial cells.  Well-Circumscribed, Lobulated Architecture:  The tumor appears encapsulated or partially encapsulated, a typical feature of pleomorphic adenoma.  Multiple nodules or lobules are observed, often demarcated by fibrous septa.  Ductal Structures:  Clearly identifiable duct-like structures with eosinophilic luminal secretions.  Often bilayered with an inner ductal and an outer myoepithelial layer.  Lack of Cytologic Atypia or Mitotic Activity:  The lesion lacks significant cellular atypia or high mitotic activity, supporting a benign nature.  Summary:  Pleomorphic adenoma is the most common benign salivary gland tumor. The combination of epithelial and myoepithelial cells in a chondromyxoid stroma, along with the lack of significant atypia and a well-circumscribed architecture, is characteristic. These features are clearly appreciable across the different magnifications in your images. | Histopathological diagnosis  Adenoid cystic carcinoma (ACC) of the salivary gland  Key microscopic features seen in the four H-E sections that support this diagnosis  Field Salient findings Significance for ACC  Overall architecture (Image 1, low power) Lobulated, infiltrative tumor composed of multiple nests that display a cribriform “Swiss-cheese” pattern with numerous punched-out pseudocystic spaces; intervening fibrous stroma The cribriform arrangement and infiltrative edges are classic for ACC  Tubular / cribriform areas (Image 2) Dual-layered tubular structures: an inner layer of cuboidal ductal cells and an outer layer of small, dark, basaloid myoepithelial cells; lumina and pseudocysts contain pale eosinophilic, basement-membrane-like (hyaline) material Tubular + cribriform patterns with two distinct cell layers and hyaline cylinders are defining histologic hallmarks  Solid basaloid foci (Image 3) Sheets and nests of uniform, hyperchromatic, scant-cytoplasm basaloid cells with high N/C ratio, separated by thin fibrous septa; tiny pseudocysts with hyaline globules scattered within Presence of a solid pattern together with cribriform/tubular areas is typical of ACC and helps grade the tumor  Perineural invasion (Image 4) Tumor cells form concentric (“targetoid”) cuffs around and within a nerve bundle; nerve is dissected by the same basaloid cell population seen elsewhere Neurotropism is a signature feature of ACC and explains the tumor’s propensity for pain and local recurrence  Stroma & contents of pseudocysts Hyalinised (basement-membrane–like) and mucinous material filling many pseudocystic spaces; scant desmoplastic stroma Hyaline cylinders are characteristic for ACC and PAS-positive on special stain  Taken together—the cribriform/tubular architecture with dual-cell layers, hyaline-filled pseudocysts, solid basaloid areas, and prominent perineural invasion—fit best with adenoid cystic carcinoma among salivary gland neoplasms. |
| 13.30 | Diagnosis: Adenoid Cystic Carcinoma; Key Microscopic Features: Solid, tubular, and cribriform patterns, with cells showing hyperchromatic nuclei and coarse chromatin, surrounded by myoepithelial cells and mitotic figures. | Based on the provided hematoxylin and eosin (H&E) stained histopathological images of a salivary gland neoplasm taken at varying magnifications, the most likely single diagnosis is:  Basal Cell Adenocarcinoma (BCAC)  Key Microscopic Features Supporting the Diagnosis:  1. Multinodular Growth Pattern (Image 1)  At low magnification, the lesion appears well-circumscribed yet infiltrative, exhibiting a multinodular architecture.  The tumor extends deeply and shows separation from the overlying epithelium.  2. Basaloid Cell Population (Images 2 and 3)  Sheets and nests of basaloid cells with hyperchromatic nuclei and scant cytoplasm.  Peripheral palisading of tumor cells in some nests—a characteristic feature.  3. Ductal Differentiation (Image 3)  Presence of duct-like or tubular structures interspersed among basaloid cells.  Lumen formation with eosinophilic secretions in some areas.  4. Infiltrative Growth and Desmoplastic Stroma (Image 4)  Infiltration into surrounding connective tissue with irregular, jagged margins.  Desmoplastic stroma with a reactive appearance, indicating malignancy.  Areas of perineural invasion may be suggested (although not overtly visible in these images, this is a common feature of BCAC).  5. Mild to Moderate Cytologic Atypia  Unlike its benign counterpart (basal cell adenoma), this neoplasm shows cytologic atypia and a more aggressive infiltrative pattern.  Differential Diagnosis Considerations:  Basal Cell Adenoma: Lacks the infiltrative pattern and cytologic atypia.  Adenoid Cystic Carcinoma: Would show more prominent cribriform architecture and perineural invasion.  Polymorphous Adenocarcinoma: Lacks the uniform basaloid appearance and typically has a more polymorphous pattern.  Conclusion:  The combination of basaloid cell morphology, ductal differentiation, multinodular architecture with infiltrative growth, and cytologic atypia best fits Basal Cell Adenocarcinoma, a low-grade malignant salivary gland tumor.  Let me know if you’d like a comparative table with differentials or immunohistochemistry (IHC) markers typically used for confirmation. | Histopathological diagnosis (single best answer)  → Adenoid cystic carcinoma (ACC) of salivary gland – mixed cribriform + solid pattern  Key microscopic features present in the four H-E images that support this diagnosis  Field Salient findings Why they point to ACC  Low-power overview (image 1) • Ill-defined/unencapsulated submucosal mass composed of multiple lobules and nests of dark basaloid cells.  • “Swiss-cheese” appearance of many lobules due to numerous small punched-out spaces. Lack of a true capsule and the lobulated, cribriform outline are classic for ACC rather than a benign basal-cell neoplasm.  Solid (“basaloid”) area (image 2) • Sheets of uniform, hyperchromatic cells with scant cytoplasm and angulated nuclei.  • Very little stroma between cells. A solid basaloid component is one of the three canonical growth patterns of ACC (solid, tubular, cribriform).  Cribriform/tubular focus (image 3) • Lobules transected by round to oval pseudocystic spaces that contain eosinophilic, hyaline basement-membrane–like material.  • True ducts/tubules showing dual cell population – inner cuboidal ductal cells surrounded by smaller basaloid/myoepithelial cells. The hyaline‐filled pseudocysts give the pathognomonic “Swiss-cheese” cribriform pattern of ACC; the bilayered ducts further distinguish it from polymorphous adenocarcinoma or pleomorphic adenoma.  Mixed pattern & stromal interface (image 4) • Cribriform/tubular islands juxtaposed with solid nests, separated by hyalinised fibrous stroma.  • Irregular infiltration of tumour between stromal bundles (capsule absent). ACC characteristically shows admixture of patterns within the same tumour and an infiltrative interface with desmoplastic stroma.  Additional diagnostic clues seen across the sections  Uniform basaloid cells with high nuclear-to-cytoplasm ratio and scant mitotic atypia.  Prominent eosinophilic (PAS-positive) basement-membrane material lining lumina and filling pseudocysts.  Absence of chondromyxoid (“pleomorphic”) matrix or peripheral palisading typical of basal-cell adenoma.  Taken together, the combination of cribriform “Swiss-cheese” architecture, dual-cell tubular structures, solid basaloid areas, hyaline basement-membrane globules, and an infiltrative growth pattern is diagnostic of adenoid cystic carcinoma. |
| 13.31 | Diagnosis: Adenoid cystic carcinoma, mostly solid variant; Key Microscopic Features: Tumor consists of solid lobules with focal cribriform patterns and comedonecrosis. Cells have minimal cytoplasm and large, angular hyperchromatic nuclei. Comedonecrosis is evident. Some areas exhibit cribriform patterns with mitotic figures and true ducts formed by eosinophilic columnar cells. | Based on the provided hematoxylin-and-eosin (H&E) stained histopathological images of a salivary gland lesion at various magnifications, the single most likely diagnosis is:  Basal Cell Adenoma (BCA)  Key Microscopic Features Supporting the Diagnosis:  Well-Circumscribed Architecture:  Seen clearly in the low-power (third) image.  Tumor is encapsulated and divided into lobules by fibrous septa, a characteristic feature of BCA.  Basaloid Cell Population:  High cellularity composed predominantly of uniform basaloid epithelial cells with hyperchromatic nuclei and scant cytoplasm (seen in high-power images 1 and 2).  No significant pleomorphism or mitotic activity.  Peripheral Palisading:  Palisading arrangement of tumor cells at the periphery of tumor islands/nests is evident in the higher magnification image.  This is a hallmark of the basaloid cell pattern in basal cell adenomas.  Basement Membrane-like Material:  Hyalinized stroma or basement membrane-like eosinophilic material separating cell nests (visible in the low- and medium-power images).  This is consistent with the histologic subtype of the membranous or solid type of basal cell adenoma.  Absence of Ductal Differentiation or Chondromyxoid Stroma:  Helps rule out pleomorphic adenoma, which commonly features ductal structures and myxoid/chondroid areas.  Also, no evidence of invasive features or perineural invasion (to rule out basal cell adenocarcinoma).  Differential Diagnoses Considered and Ruled Out:  Pleomorphic Adenoma: Lacks the myxoid or cartilaginous stroma seen in pleomorphic adenoma.  Adenoid Cystic Carcinoma: Although also basaloid, it shows more infiltrative growth, perineural invasion, and cribriform patterns — not evident here.  Basal Cell Adenocarcinoma: More cytologic atypia and invasive growth are typically seen, absent in these images. | Histopathological diagnosis  Basal cell adenoma (membranous / “cylindromatous” type) of the salivary gland  Key microscopic features visible in the three fields that support this diagnosis  Field Salient findings Diagnostic significance  Low-power overview (image 3) • Well-circumscribed, lobulated tumour divided by thick, eosinophilic fibrous septa.  • Each lobule is solid and basophilic, lacking chondromyxoid or lymphoid stroma.  • Scattered small round “punched-out” spaces in some nests. Classic architecture of a basal-cell neoplasm: nodular growth, sharp borders, and a fibrous pseudocapsule. Absence of myxoid matrix rules out pleomorphic adenoma; absence of infiltrative edges argues against basal-cell adenocarcinoma.  Intermediate magnification (image 1) • Solid nests of uniform basaloid cells with scant cytoplasm and round hyperchromatic nuclei.  • Peripheral nuclear palisading along the outer rim of many nests.  • Multiple intra- and inter-nest deposits of dense pink hyaline basement-membrane–like material; some appear as rounded droplets. A basaloid cell population with palisading is typical of basal cell adenoma. The conspicuous, PAS-positive hyaline material is the hallmark of the membranous (cylindromatous) variant — resembling cutaneous cylindroma.  High-power detail (image 2) • Central, sharply circumscribed spherical mass of thick eosinophilic hyaline material surrounded by basaloid cells (“cylindromatous globule”).  • Cells remain cytologically bland; mitoses and necrosis are negligible. The large hyaline globule is characteristic of the membranous type and distinguishes it from adenoid cystic carcinoma (which shows multiple smaller pseudocystic spaces in a cribriform pattern and often shows angulated lumina, infiltrative margins and perineural invasion).  Diagnostic synthesis  The combination of (1) circumscribed lobular architecture, (2) uniform basaloid cells with peripheral palisading, and (3) abundant thick basement-membrane–like hyaline material deposited inside and around the nests is pathognomonic for basal cell adenoma of the membranous (cylindromatous) type in salivary gland pathology. |
| 13.32 | Diagnosis: Epithelial-myoepithelial carcinoma; Key Microscopic Features: Tumor islands exhibit a biphasic pattern with prominent clear cells and central ducts made up of eosinophilic cells. Each island contains a central duct with luminal cells and surrounding clear cells. | Based on the histopathological examination of the provided hematoxylin and eosin (H&E) stained images of a salivary gland neoplasm, here is the diagnostic assessment:  🔬 Most Likely Histopathological Diagnosis:  Mucoepidermoid Carcinoma (MEC)  🧠 Key Microscopic Features Supporting This Diagnosis:  Mixed Cell Population:  The images show a combination of mucous cells, epidermoid (squamoid) cells, and intermediate cells, which is a hallmark of mucoepidermoid carcinoma.  The mucous cells appear with foamy/vacuolated cytoplasm due to mucin content, and are seen prominently in the first high-magnification image.  Cystic and Solid Architecture:  The tumor displays both solid nests and cystic spaces, which is typical for MEC.  The cystic spaces are lined by mucous-producing cells, which helps differentiate MEC from other solid tumors like adenoid cystic carcinoma or acinic cell carcinoma.  Nuclear Features:  Cells show moderate pleomorphism, with hyperchromatic nuclei, and scant mitotic activity — features that suggest a low to intermediate-grade MEC.  Background Stroma:  The stroma appears fibrous with some degree of chronic inflammation, especially in the lower magnification image.  The growth pattern in the second image suggests infiltrative behavior, supporting the diagnosis of malignancy.  Absence of Key Features of Other Salivary Neoplasms:  No cribriform or tubular patterns (as in adenoid cystic carcinoma),  No serous acinar cells with basophilic granular cytoplasm (as in acinic cell carcinoma),  No chondromyxoid stroma or dual cell populations (as seen in pleomorphic adenoma).  📌 Conclusion:  The combination of mucous-producing cells, epidermoid cells, and intermediate cells forming cystic and solid areas is diagnostic of mucoepidermoid carcinoma, the most common malignant salivary gland tumor. | Histopathologic diagnosis  → Secretory carcinoma of the salivary gland ( “mammary-analogue” secretory carcinoma )  Key microscopic features seen in the two H-E fields  Power What is seen Why it supports secretory carcinoma  Low-power field (second image) • Lobulated, but unencapsulated nodule  • Cellular lobules and nests delineated by delicate, pink, fibrous septa  • Predominantly solid-to-microcystic architecture with only small, slit-like true cysts The lobulated, septated outline and microcystic/solid growth pattern are classic for secretory carcinoma and help separate it from pleomorphic adenoma (markedly myxoid) or adenoid cystic carcinoma (basaloid cribriform).  High-power field (first image) • Tubular and microcystic spaces filled with eosinophilic secretory material (pink luminal droplets)  • Tumour cells are uniform, with abundant foamy / vacuolated or “bubbly” cytoplasm and round-to-oval nuclei with inconspicuous nucleoli  • Many cells show intracytoplasmic vacuoles that push the nucleus eccentrically, giving a signet-ring-like look  • No basophilic zymogen granules (rules out acinic-cell carcinoma) and no obvious squamous/epidermoid differentiation (rules out mucoepidermoid carcinoma)  • Stroma between nests is delicate, lightly hyalinised, not chondromyxoid (rules out pleomorphic adenoma) The combination of vacuolated cytoplasm, pink intraluminal secretions and a purely glandular/microcystic architecture is the diagnostic triad for secretory carcinoma and corresponds to the ETV6–NTRK3-rearranged entity described in salivary glands.  Bottom line – why this is the single best fit  Secretory carcinoma uniquely shows (1) a lobulated, microcystic/tubular pattern, (2) tumour cells with pale or vacuolated “lactation-type” cytoplasm, and (3) profuse eosinophilic luminal and intracytoplasmic secretory material.  All three features are present in the provided sections, while key hallmarks of the main histologic mimics (granular basophilic acinar cytoplasm, squamo-mucinous mixture, cribriform basaloid nests or outer clear-cell layers) are absent, making secretory carcinoma the most appropriate diagnosis. |
| 13.33 | Diagnosis: Polymorphous adenocarcinoma, classic/conventional type; Key Microscopic Features: Sheets, ducts, small islands, and cell cords within myxohyaline stroma. Ductal structures are present and cords of cells infiltrate adjacent glands. Tumor cells are isomorphic with bland nuclei, small nucleoli, and a mitotic figure. Some cells have clear cytoplasm. | Based on the analysis of the provided hematoxylin-and-eosin (H&E) stained histological images at different magnifications, the single most likely histopathological diagnosis is:  Mucoepidermoid Carcinoma (MEC) – Salivary gland origin.  Key Microscopic Features Justifying the Diagnosis:  1. Cystic and Solid Growth Patterns (best seen in the low-power image):  The lesion shows a combination of solid nests and cystic areas.  This biphasic growth pattern is characteristic of mucoepidermoid carcinoma.  2. Mixture of Cell Types (better appreciated at intermediate and high magnification):  Mucous cells: These cells appear pale or vacuolated due to mucin content.  Epidermoid (squamoid) cells: Polygonal cells with eosinophilic cytoplasm and intercellular bridges may be visible.  Intermediate cells: These predominate and are small-to-medium sized with scant cytoplasm and round-to-oval nuclei.  3. Nuclear Pleomorphism and Mitoses (highlighted in high-power image):  Evidence of mild to moderate pleomorphism and occasional mitotic figures.  This suggests an intermediate-grade MEC, though a full grading requires more architectural and cytological assessment.  4. No Chondromyxoid Stroma or Perineural Invasion (helps rule out other entities):  The absence of a myxochondroid stroma rules out pleomorphic adenoma.  Lack of perineural invasion and high-grade atypia reduces the likelihood of high-grade malignancies like salivary duct carcinoma or adenoid cystic carcinoma.  Differential Diagnoses Considered and Ruled Out:  Diagnosis Reason for Exclusion  Pleomorphic adenoma Lacks characteristic chondromyxoid stroma and myoepithelial differentiation.  Adenoid cystic carcinoma No cribriform/"Swiss cheese" pattern or perineural invasion.  Acinic cell carcinoma Absence of basophilic granular cytoplasm (zymogen granules).  Salivary duct carcinoma No Roman bridge architecture or high-grade atypia.  Conclusion:  The combination of mixed cell populations (mucous, intermediate, and epidermoid), cystic and solid architecture, and mild-to-moderate atypia supports the diagnosis of mucoepidermoid carcinoma, most likely intermediate grade. Further confirmation with mucin stains (e.g., mucicarmine) or immunohistochemistry may assist in confirming mucous cell presence if required. | Most likely histopathological diagnosis  → Acinic cell carcinoma (low-grade serous-acinar type salivary gland carcinoma)  Microscopic features in the three H-E sections that support this diagnosis  Field/structure What is seen Why it points to acinic cell carcinoma  Overall architecture (low power) Lobulated, relatively well-circumscribed mass of purple-staining cells separated by delicate fibrous septa; pushes up against overlying mucosa but shows focal infiltration of surrounding stroma Lobulated but infiltrative growth pattern is typical of acinic cell carcinoma; benign lesions (e.g., pleomorphic adenoma) are usually encapsulated  Growth pattern (intermediate power) Predominantly solid and microcystic/aciniform nests; occasional small lumina and follicle-like spaces Solid and microcystic/acinic patterns are two of the classic architectural forms in acinic cell carcinoma  Cytoplasm (high power) Tumour cells have abundant, finely granular, intensely basophilic (purple) cytoplasm; granularity is coarse in some cells Basophilic granules correspond to zymogen (serous) secretory granules—hallmark of serous acinar differentiation  Nuclei Round-to-oval, centrally to eccentrically placed nuclei, mostly uniform; inconspicuous nucleoli; mild pleomorphism, low mitotic activity Low-grade cytology with uniform nuclei is characteristic; high-grade pleomorphism would suggest other entities (e.g., salivary duct carcinoma)  Stroma / mucin Minimal mucin; no islands of squamoid or mucous cells; no chondromyxoid matrix Lack of mucous cells rules out muco-epidermoid carcinoma; absence of myxochondroid stroma excludes pleomorphic adenoma  Peripheral interface Irregular tongues of tumour permeating adjacent salivary tissue/fibrous stroma without a true capsule Acinic cell carcinoma often shows a “pushing-infiltrative” margin rather than an encapsulated border  Taken together, the combination of serous acinar-type cells with granular basophilic cytoplasm, solid/microcystic architecture, low-grade cytology and a lobulated infiltrative outline is diagnostic of acinic cell carcinoma of salivary gland origin. |
| 13.34 | Diagnosis: Polymorphous adenocarcinoma, classic/conventional type; Key Microscopic Features: Solid tumor mass with peripheral strands of cells, some of which are clear. Ductal structures with clusters of mucous cells in hyalinized stroma. Perineural invasion is noted. Cells have pale or clear cytoplasm and nuclei with dispersed chromatin and small nucleoli. | Based on the histopathological evaluation of the provided Hematoxylin and Eosin (H&E) stained images of a salivary gland lesion, the most likely single diagnosis is:  Mucoepidermoid Carcinoma (MEC)  Key Microscopic Features Supporting the Diagnosis:  Mixed Cell Population:  Mucin-producing cells (seen as pale/foamy cytoplasm or vacuolated areas, especially visible in image 3).  Epidermoid/squamoid cells with eosinophilic cytoplasm and intercellular bridges.  Intermediate cells, which are less distinct but populate much of the tumor.  Mucin Pools:  Visible intracellular or extracellular mucin (e.g., image 3 shows mucin-positive cells with clear cytoplasm and central nuclei), indicative of mucous cell components.  Cystic and Solid Areas:  Images show both solid sheets of tumor cells (image 1 and 4) and cystic/mucinous spaces, a classic feature of MEC.  Infiltrative Growth Pattern:  Especially in images 1 and 3, there's disruption of the surrounding stromal architecture, suggesting invasive behavior.  Mild to Moderate Pleomorphism:  There is nuclear variability but not marked atypia or high mitotic activity, which suggests low- to intermediate-grade MEC.  Summary:  The presence of three distinct cell types (mucous, epidermoid, and intermediate), along with mucin pools and infiltrative yet variably cystic growth, strongly supports the diagnosis of Mucoepidermoid Carcinoma—the most common malignant salivary gland tumor.  If immunohistochemistry were available, markers such as CK7, MUC5AC, and p63 would further aid confirmation. However, the H&E findings are classic for MEC. | Histopathological diagnosis  → Adenoid cystic carcinoma (ACC) of a salivary gland  Key microscopic features visible in the four H-E fields that support this diagnosis  Image Salient findings Relevance to ACC  0 (low-intermediate power) • Infiltrative, basophilic tumor mass (upper half) abutting dense fibrous tissue.  • Nerve-like bundle in the stroma cuffed by tumor cells → perineural invasion. Perineural spread is a classic hallmark of ACC.  1 (intermediate power) • Cribriform (“Swiss-cheese”) pattern – nests perforated by multiple, round punched-out spaces.  • The spaces contain pale eosinophilic, hyaline basement-membrane–like material. Cribriform architecture with hyaline cylinders is the signature pattern of ACC.  2 (high power) • Tubular pattern – small true ducts with an inner eosinophilic ductal cell layer and an outer clear (myoepithelial) cell layer.  • Lumina filled with faint eosinophilic secretions. Dual epithelial–myoepithelial cell layers forming tubules are characteristic of the tubular variant of ACC.  3 (high power) • Solid sheets of basaloid cells with hyperchromatic, angulated nuclei, scant cytoplasm, frequent mitoses.  • Intervening delicate fibro-hyaline stroma. The solid pattern is one of the three canonical growth patterns of ACC and confers a higher-grade component.  Additional synthesized features across the sections  Mixture of cribriform, tubular and solid patterns in a single lesion – ACC often shows this architectural heterogeneity.  Uniform, small-to-medium basaloid cells with high N:C ratio and sharply angulated nuclei.  Prominent, glassy, PAS-positive basement-membrane-type material within and around tumor nests.  Desmoplastic or hyalinised stroma traversed by cords and nests of tumor cells.  The combination of these findings (especially cribriform hyaline cylinders, dual-layered tubules, solid basaloid areas and perineural invasion) is diagnostic of adenoid cystic carcinoma rather than other salivary gland neoplasms. |
| 13.35 | Diagnosis: Polymorphous adenocarcinoma, classic/conventional type; Key Microscopic Features: Nests and trabeculae of tumor cells with duct formation, focal cribriform patterns, and myxochondroid stroma. Cords and strands of tumor cells are present in fibromyxoid stroma. Interconnected strands and cords within myxochondroid stroma. Tumor cells are isomorphic, some with vacuolated cytoplasm and bland nuclei. | Based on the provided hematoxylin and eosin (H&E) stained histopathology images at different magnifications of a lesion in the salivary gland neoplasms category, the single most likely histopathological diagnosis is:  Diagnosis: Basal Cell Adenoma (BCA)  Key Microscopic Features Supporting the Diagnosis:  Basaloid Cell Proliferation:  The tumor is composed of uniform, small, basaloid epithelial cells with scant cytoplasm and hyperchromatic nuclei.  These cells are arranged in various patterns, including solid nests, trabecular, and tubular arrangements.  Peripheral Palisading:  At higher magnification, the peripheral cells in the nests show palisading, a hallmark of basal cell adenoma.  This mimics basal cell carcinoma but without significant atypia or mitotic activity.  Basement Membrane-like Material:  Presence of abundant eosinophilic hyaline basement membrane-like material surrounding and within the epithelial islands—typical of basal cell adenomas.  Encapsulation and Lack of Invasion:  The lesion appears well-circumscribed and partially encapsulated, lacking any evidence of infiltrative growth.  No perineural or vascular invasion is observed, which helps differentiate it from basal cell adenocarcinoma.  Fibromyxoid Stroma:  The intervening stroma is fibromyxoid to collagenous in nature and contributes to the separation of tumor nests.  Differential Considerations (but less likely due to histologic features):  Adenoid Cystic Carcinoma: Shows cribriform pattern and perineural invasion—features not seen here.  Pleomorphic Adenoma: Exhibits more diverse epithelial and mesenchymal elements including chondromyxoid stroma—not evident in these images.  Basal Cell Adenocarcinoma: Would show similar basaloid features but with evidence of invasion and increased mitotic activity. | Histopathological diagnosis  → Adenoid cystic carcinoma (ACC) – predominantly cribriform/tubular type  Key microscopic features visible in the provided H-E sections  Image level Salient findings How they point to ACC  High-power (first frame) • Compact nests of uniform basaloid cells with scant cytoplasm and hyperchromatic, angulated nuclei.  • Numerous round “empty” pseudocystic spaces scattered within the nests – they represent cut-through lumina filled with basement-membrane-type hyaline material (appears pale/clear on H-E). The basaloid cytology and “Swiss-cheese” pseudocysts are classic for the cribriform pattern of ACC.  Intermediate power (second frame) • Tumour arranged in well-defined lobules/islands separated by broad bands of hyalinised, eosinophilic stroma.  • Within lobules, a mixture of cribriform and tubular areas is evident. The sharply delineated basaloid islands and intervening hyaline basement-membrane material create the characteristic architecture of ACC.  Low–intermediate power (third & fourth frames) • Infiltrative cords and ribbons of tumour cells track through a densely sclerosed / hyalinised stroma, producing a “streaming” appearance.  • No true acinar differentiation or large mucous cell component is seen. The infiltrative growth through a sclerotic bed is typical of ACC and helps distinguish it from benign basaloid tumours.  Additional points seen or strongly suggested  Perineural tracking is common in ACC (not captured in these particular fields but often found on deeper levels).  Mitotic activity is low-to-moderate, consistent with conventional ACC rather than a high-grade transformation.  Absence of abundant squamoid cells or large pools of extracellular mucin helps rule out mucoepidermoid carcinoma; lack of prominent clear-cell outer layer argues against epithelial-myoepithelial carcinoma.  Taken together, the uniform basaloid cytology, cribriform (“Swiss-cheese”) and tubular patterns, and prominent hyaline basement-membrane-type material provide a classic picture of adenoid cystic carcinoma of salivary gland origin. |
| 13.37 | Diagnosis: Polymorphous adenocarcinoma, cribriform type; Key Microscopic Features: Tumor is present in lobules with microcystic and cribriform patterns. Cribriform pattern with central necrosis and myxoid stroma. Tumor cells have pale-to-clear, round-to-ovoid overlapping nuclei. | Based on the three hematoxylin and eosin (H&E) stained histological images of a salivary gland neoplasm, taken at increasing magnifications, the most likely histopathological diagnosis is:  Mucoepidermoid Carcinoma (MEC)  Key Microscopic Features Supporting the Diagnosis:  1. Mixture of Cell Types:  The images reveal three characteristic cell populations:  Mucin-producing (mucous) cells: Appear as cells with abundant pale, foamy/vacuolated cytoplasm (especially prominent in the higher magnification image).  Intermediate cells: Polygonal to round cells with moderate cytoplasm and central nuclei, forming the bulk of the tumor mass.  Epidermoid (squamoid) cells: Cells with eosinophilic cytoplasm and larger, more irregular nuclei; less prominent in these fields but still evident in parts.  2. Cystic and Solid Architecture:  Multiple cyst-like spaces and solid nests/lobules are evident. These are typical of low to intermediate-grade MEC, where mucous-filled cystic areas are intermixed with solid epithelial islands.  3. Mucin Pools:  Presence of extracellular mucin is visible in the second and third images, showing pale areas within and around tumor cell nests. These are diagnostic of mucinous differentiation.  4. No Prominent Perineural or Lymphovascular Invasion (in current fields):  Although not seen in the current images, absence of these features does not rule out MEC but would be relevant for grading.  Conclusion:  The presence of a mixture of mucous, intermediate, and epidermoid cells forming cystic and solid areas, along with mucin-filled spaces, is diagnostic of Mucoepidermoid Carcinoma, the most common malignant salivary gland tumor. | Histopathological diagnosis  Muco-epidermoid carcinoma (intermediate-grade) of a salivary gland  Key microscopic features that point to this diagnosis  Image Salient findings Why they are characteristic of muco-epidermoid carcinoma  Low power (1st image) • Lobulated tumour divided by delicate fibrous septa.  • Mixture of solid nests/cords and cyst-like spaces, some containing pale material. Lobulated, variably solid-cystic architecture is typical of MEC and contrasts with the sharply encapsulated outline of most benign salivary tumours.  Intermediate power (2nd image) • Within each lobule three intermingled cell types are evident:    – Mucous cells: large, foamy, pale cytoplasm.    – Intermediate cells: smaller polygonal basophilic cells.    – Epidermoid/squamoid cells: eosinophilic cytoplasm in places.  • Foci of intraluminal pale mucin and patchy necrotic-like debris. The obligatory mixture of mucous, intermediate and epidermoid cells is the diagnostic hallmark of MEC. The cystic lumina frequently contain mucin.  High power (3rd image) • Mucous (signet-ring) cells with large intracellular mucin vacuoles pushing nuclei to the periphery.  • Intermediate cells showing mild–moderate cytologic atypia; mitoses sparse.  • Lack of serous acinar granules, hyaline cylinders or chondromyxoid stroma. Intracytoplasmic mucin vacuoles identify mucous cells; their coexistence with intermediate cells confirms MEC and helps exclude acinic cell carcinoma (zymogen-rich granular cytoplasm) and adenoid cystic carcinoma (basaloid cells with hyaline globules).  Together, the lobulated solid-cystic pattern and the tri-cellular population dominated by mucous and intermediate elements with abundant intracytoplasmic mucin are diagnostic of muco-epidermoid carcinoma. The moderate degree of cyst formation and cytologic atypia is most consistent with an intermediate-grade lesion. |
| 13.38 | Diagnosis: Polymorphous adenocarcinoma, cribriform type; Key Microscopic Features: Tumor is present in lobules with microcystic, cribriform, and trabecular patterns. Areas suggestive of classic polymorphous adenocarcinoma with cords of cells and myxoid stroma. Cribriform pattern with glomeruloid structure; tumor cells have moderate amounts of pale cytoplasm, pale, round-to-ovoid overlapping nuclei with small nucleoli. | Based on the provided hematoxylin and eosin (H&E) stained histopathological images of a salivary gland lesion at various magnifications, the most likely diagnosis is:  Mucoepidermoid Carcinoma (MEC)  Key Microscopic Features Supporting This Diagnosis:  Mixture of Cell Types:  The hallmark feature of MEC is the presence of a mixture of mucous, epidermoid (squamoid), and intermediate cells.  In the higher magnification image, mucous cells appear with foamy, pale cytoplasm; epidermoid cells are larger with eosinophilic cytoplasm and prominent nuclei; intermediate cells are smaller and less differentiated.  Cystic and Solid Areas:  The low and medium magnification images show areas of cystic change intermixed with solid nests and lobules of tumor cells.  These cystic areas are characteristic of low- to intermediate-grade mucoepidermoid carcinoma.  Duct-like Structures and Mucin Production:  Presence of mucin pools or cystic spaces lined by mucous cells, and occasionally mucin extravasation is apparent, which is a defining feature.  Special stains (e.g., mucicarmine, PAS-D) would highlight mucin, but the morphology here strongly suggests mucin-producing cells.  Lobulated Growth Pattern:  The tumor appears lobulated, separated by fibrous septa.  This pattern is frequently seen in MEC and helps differentiate it from other salivary gland neoplasms like acinic cell carcinoma or pleomorphic adenoma.  Nuclear Features:  There is mild to moderate pleomorphism, with visible mitotic activity in some areas — consistent with intermediate-grade MEC.  Differential Diagnoses Considered:  Acinic cell carcinoma – lacks the mucous cell component and has more basophilic, granular cytoplasm.  Pleomorphic adenoma – typically shows myxochondroid stroma and dual epithelial/myoepithelial differentiation, not seen here.  Adenoid cystic carcinoma – would show cribriform (“Swiss cheese”) pattern and lacks mucous cells.  Conclusion:  The constellation of mucous and squamoid cells, cystic and solid architecture, and lobulated growth pattern most strongly supports a diagnosis of Mucoepidermoid Carcinoma. | Histopathological diagnosis  Acinic cell carcinoma (ACC) – predominantly microcystic / solid pattern  Key microscopic features that support this diagnosis  Field Observations Significance  Architecture (low-power) • Tumour is lobulated but unencapsulated, with fibrous septa separating large nests/lobules.  • Within lobules innumerable tiny round-to-oval spaces create a microcystic/“Swiss-cheese” appearance.  • Foci of solid sheets and trabeculae mingle with the microcystic areas. Classic growth patterns of acinic cell carcinoma – microcystic and solid are the two commonest.  Luminal content Many of the microcysts contain pale eosinophilic secretory material; some are empty slit-like spaces. Secretory product in ACC; helps to distinguish from purely basaloid tumours such as adenoid cystic carcinoma (whose pseudocysts are typically filled with dense hyaline basement-membrane material).  Cell morphology (high-power) • Cells are polygonal with moderate amphophilic-to-basophilic granular cytoplasm (zymogen granules).  • Nuclei are round/oval, centrally placed, with finely granular chromatin and inconspicuous nucleoli.  • Cytoplasmic vacuoles are occasionally seen. Serous acinar differentiation – hallmark of ACC. Cytoplasmic granularity distinguishes it from basaloid carcinomas or mucoepidermoid carcinoma.  Cytologic grade • Minimal pleomorphism, low mitotic activity, no necrosis. Fits the usual low- to intermediate-grade nature of ACC.  Stroma / interface • No chondromyxoid (rules out pleomorphic adenoma).  • No obvious dual cell layers or prominent clear myoepithelial rim (rules out epithelial-myoepithelial carcinoma). Helps narrow to ACC among salivary neoplasms.  Taken together, the lobulated microcystic architecture full of eosinophilic secretions, the uniform serous-acinar-type cells with basophilic granular cytoplasm, and the low-grade cytology are diagnostic of acinic cell carcinoma of the salivary gland. |
| 13.39 | Diagnosis: Secretory carcinoma; Key Microscopic Features: Tumor has a microcystic pattern with hyalinized stroma. Ducts are filled with bubbly secretions, and tumor cells have amphophilic vacuolated cytoplasm. Nuclei are vesicular and clear with small nucleoli. | Based on the analysis of the provided Hematoxylin and Eosin (H&E) stained histopathology images from a salivary gland neoplasm, here is the interpretation:  🧠 Histopathological Diagnosis:  Mucoepidermoid Carcinoma (MEC) – the most common malignant salivary gland tumor.  🔬 Key Microscopic Features Supporting This Diagnosis:  1. Cellular Heterogeneity:  Mixture of cell types including:  Mucous cells: Clear to pale-staining cytoplasm (mucicarmine-positive on special stains), evident in the second image.  Intermediate cells: Cells with basaloid appearance and high nuclear-cytoplasmic ratio.  Epidermoid (squamoid) cells: Cells with eosinophilic cytoplasm and intercellular bridges.  2. Cystic and Solid Architecture:  The low-power image (first image) shows a combination of cystic spaces and solid nests, a hallmark of low- to intermediate-grade MEC.  Cystic areas are lined by mucous cells, while solid islands consist of intermediate and epidermoid cells.  3. Prominent Mucin Production:  Clear cells in the second image likely represent mucin-producing mucous cells, contributing to the cystic architecture.  Intracytoplasmic mucin (clear vacuoles) is a critical diagnostic clue.  4. Nuclear Features:  Mild-to-moderate nuclear pleomorphism; relatively uniform round to oval nuclei with prominent nucleoli in some areas.  Lack of high-grade features such as extensive necrosis, perineural invasion, or high mitotic activity, suggesting intermediate or low-grade MEC.  5. Stromal Component:  Desmoplastic (fibrous) stroma surrounding the epithelial islands and cysts.  📌 Summary:  The combination of mucous, intermediate, and squamoid/epidermoid cells in a mixed cystic and solid pattern, alongside mucin production and the absence of high-grade atypia, strongly supports the diagnosis of Mucoepidermoid Carcinoma. | Histopathological diagnosis  Low-grade mucoepidermoid carcinoma (MEC) of a salivary gland  Key microscopic features supporting this diagnosis  Field What is seen Why it points to MEC  Overall architecture (low-power image) Lobulated, infiltrative tumour with numerous round to oval micro- and macrocystic spaces that appear empty/white, separated by solid nests and cords of cells A predominance of true cystic spaces is typical of low-grade MEC; most other salivary tumours with clear cells (e.g., acinic-cell, secretory, epithelial–myoepithelial) are more solid or tubular  Cell population (high-power image) A heterogeneous admixture of three cell types:  • Mucous cells – abundant foamy/clear cytoplasm containing one or more mucin vacuoles (many show a signet-ring outline)  • Intermediate cells – smaller polygonal cells with scant, lightly basophilic cytoplasm and round nuclei  • Scattered epidermoid/squamoid cells with more eosinophilic cytoplasm and sharper borders The simultaneous presence of mucous, intermediate and epidermoid elements is the diagnostic hallmark of MEC and distinguishes it from acinic-cell carcinoma (serous acinar cells only) or secretory carcinoma (uniform secretory cells)  Intraluminal/intracytoplasmic material Empty-looking vacuoles and cystic spaces correspond to dissolved mucin; focal pools of pale pink mucin are also present Abundant mucin production is characteristic of MEC and not a feature of most other clear-cell salivary tumours  Nuclear features & stroma Nuclei are mostly uniform, with inconspicuous nucleoli and low mitotic activity; haemorrhagic and fibromyxoid stroma seen focally Bland cytology and low proliferative activity support a low-grade lesion  Because the tumour shows predominant cystic architecture, plentiful mucous cells with intracytoplasmic vacuoles, and the classic tri-cellular composition, the single most appropriate diagnosis within the salivary-gland neoplasm spectrum is low-grade mucoepidermoid carcinoma. |
| 13.40 | Diagnosis: Secretory carcinoma; Key Microscopic Features: Tumor exhibits microcystic and papillary patterns. Cells have pale, multivacuolated cytoplasm and bland nuclei with small nucleoli. Areas with a papillary growth pattern. | Based on the provided hematoxylin and eosin (H&E) stained histopathological images at low, medium, and high magnification, the single most likely histopathological diagnosis is:  Mucoepidermoid Carcinoma (MEC) of the salivary gland  Key Microscopic Features Supporting the Diagnosis:  Mixture of Cell Types:  Mucous cells: Seen as large cells with pale, foamy cytoplasm (due to mucin) and basally located nuclei (visible in high magnification image).  Epidermoid (squamoid) cells: Polygonal cells with eosinophilic cytoplasm and central round nuclei.  Intermediate cells: Cells with features between mucous and epidermoid types, often seen throughout the tumor.  Cystic and Solid Architecture:  Areas with cystic degeneration, filled with mucin and blood, are prominent.  Solid nests and cords of tumor cells infiltrating the surrounding stroma, as shown especially in the low and medium magnification images.  Intracytoplasmic and Extracellular Mucin:  Presence of mucin is confirmed by the foamy, vacuolated cytoplasm of mucous cells and mucin pools (best seen in high magnification).  Mucin-containing cystic spaces are intermixed with solid tumor islands.  Hemorrhage and Vascularity:  Red blood cells within vascular spaces and extravasated in cystic areas are common (clearly visible in the low- and high-power views).  Mild to Moderate Cytologic Atypia:  Nuclei vary slightly in size and shape but are not highly pleomorphic, consistent with low- to intermediate-grade MEC.  Summary:  The combination of three distinct cell types (mucous, epidermoid, intermediate), mucin production, mixed cystic and solid growth, and the characteristic appearance across magnifications strongly supports the diagnosis of Mucoepidermoid Carcinoma, the most common malignant salivary gland tumor. | Histopathological diagnosis – Acinic cell carcinoma (microcystic/solid variant) of salivary gland  Key microscopic features visible in the three H-E sections  Field & feature What is seen in the images Diagnostic significance  Circumscription & location Non-encapsulated but fairly well-circumscribed mass immediately beneath an intact surface mucosa/skin (1st image, low power) Typical for many low-grade salivary gland neoplasms, including acinic cell carcinoma (ACC)  Architectural patterns Mixture of solid sheets, microcystic/“Swiss-cheese” spaces and focal papillary–cystic clefts filled with fresh haemorrhage/RBCs (all images) ACC commonly shows a combination of solid, microcystic and papillary-cystic patterns  Cell morphology Uniform polygonal cells with abundant granular to finely vacuolated cytoplasm; cytoplasm stains pale-lavender to amphophilic with a sprinkling of coarse basophilic granules (best in 2nd & 3rd images) Granular, PAS-D-positive zymogen granules are the hallmark of serous acinar differentiation in ACC  Nuclear features Round–oval, centrally to eccentrically placed nuclei; small nucleoli; minimal pleomorphism and low mitotic activity (2nd & 3rd images) Supports a low-grade carcinoma such as ACC rather than a high-grade malignancy  Stroma & vasculature Delicate fibrovascular septa; prominent thin-walled vascular channels with pools of erythrocytes/haemorrhage (all images) Vascular-rich stroma and frequent intratumoral bleeding are characteristic, especially in the microcystic variant  Absence of other cell types No true mucous cells, epidermoid/squamoid cells or hyalinised stroma are identified Helps exclude muco-epidermoid carcinoma, secretory carcinoma and hyalinising clear-cell carcinoma  Together, the serous-acinar-type cytoplasm with basophilic granules, the mixed solid–microcystic architecture and the low-grade cytology point squarely to acinic cell carcinoma. |
| 13.41 | Diagnosis: Acinic cell carcinoma; Key Microscopic Features: Solid lobular growth with tumor-associated lymphoid stroma and infiltration of normal mucous glands. Sheets of clear and darker-staining cells. Cells contain zymogen granules and round nuclei. Many clear cells are present. Tumor-associated lymphoid proliferation adjacent to cells with pale, granular cytoplasm. | Based on the provided hematoxylin and eosin (H&E) stained images of a salivary gland neoplasm at different magnifications, the single most likely histopathological diagnosis is:  Mucoepidermoid Carcinoma (MEC)  Key Microscopic Features Supporting This Diagnosis:  Mixture of Cell Types:  Mucous cells: Seen as large cells with clear, foamy cytoplasm (especially prominent in image 3), indicative of mucin production.  Epidermoid (squamoid) cells: Polygonal cells with eosinophilic cytoplasm and central nuclei (clear in image 4).  Intermediate cells: Smaller, basaloid cells located between mucous and epidermoid cells (also present in images 2 and 4).  Cystic and Solid Growth Patterns:  Low-power image (image 1) shows a lobulated architecture with both solid nests and cystic areas, a hallmark of MEC.  Mucin Production:  The presence of mucin-filled vacuoles (appearing as optically clear spaces) within cells (particularly in image 3) supports the mucous component typical of MEC.  Infiltrative Growth Pattern:  At low magnification (image 1), the tumor shows irregular infiltration into surrounding tissue, a malignant feature.  Absence of Chondromyxoid Stroma:  Helps to distinguish this lesion from pleomorphic adenoma, which has a characteristic chondromyxoid (myxochondroid) background not present here.  Summary:  The combination of mucous, epidermoid, and intermediate cells, along with the cystic and solid architecture, mucin production, and infiltrative growth, strongly supports the diagnosis of Mucoepidermoid Carcinoma, which is the most common malignant salivary gland tumor. The images reflect features typically seen in a low- to intermediate-grade lesion. | Histopathological diagnosis  → Secretory carcinoma of salivary gland ( “Mammary-analogue secretory carcinoma”, low-grade )  Key microscopic clues in the provided slides  Field / magnification What is seen How it supports the diagnosis  Low power (slide 1) • Sharply circumscribed, lobulated mass divided by fibrous septa.  • Each lobule shows a micro-cystic / follicular architecture with innumerable tiny round lumina that look “sprinkled” through the parenchyma. Secretory carcinoma classically grows in well-defined lobules and almost always displays a micro-cystic or tubular pattern with small secretory spaces.  Intermediate power (slide 2) • Sheets and cords of fairly uniform polygonal cells.  • Cytoplasm is pale to amphophilic with multiple small vacuoles, producing a bubbly or “granular” look.  • Delicate vascular channels course between the rows of tumour cells. The vacuolated, frothy cytoplasm reflects abundant intracellular secretory material; monotony of the cells and absence of true acinar basophilic granules separates it from acinic-cell carcinoma.  High power (slide 3) • Cells contain intracytoplasmic eosinophilic secretion and variably-sized vacuoles; some have a small central nucleolus.  • No keratin pearls or overt squamous differentiation. Intracytoplasmic secretory droplets are a hallmark of secretory carcinoma and account for its strong PAS-D-positive material and mammaglobin/S-100 immunoreactivity in practice.  Interface (slide 4) • A band of reactive lymphoid tissue abuts, but does not intermingle with, the tumour.  • Tumour cells form solid trabeculae with eosinophilic to lightly granular cytoplasm and bland round nuclei. Peritumoral lymphoid aggregates may accompany secretory carcinoma and help to exclude Warthin tumour (which shows a bilayered oncocytic epithelium and papillary cystic spaces).  Summary of diagnostic features present  Lobulated, micro-cystic / follicular architecture with plentiful small round lumina filled with eosinophilic material.  Uniform cells with pale, vacuolated (“secretory”) cytoplasm; lack of basophilic zymogen granules that would indicate acinic-cell carcinoma.  Intracytoplasmic and intraluminal secretory droplets, producing the characteristic frothy appearance.  Low-grade nuclear features – round nuclei, inconspicuous nucleoli, minimal pleomorphism.  Peripheral lymphoid stroma without the bilayered oncocytic epithelium of a Warthin tumour and without the triphasic (mucous–intermediate–epidermoid) pattern of mucoepidermoid carcinoma.  Taken together, these findings are most consistent with secretory carcinoma of salivary gland (MASC), a low-grade malignancy defined by the ETV6-NTRK3 fusion and characterised histologically by the combination of lobulated micro-cystic growth and secretory (“bubbly”) cytoplasm seen here. |
| 13.42 | Diagnosis: Acinic cell carcinoma of minor salivary glands; Key Microscopic Features: Solid, lobular tumor with hemorrhage and a microcystic pattern, showing cells with granular cytoplasm and mitotic activity. | Histopathological Analysis of Salivary Gland Neoplasm (H&E Stained Images)  Based on the three provided H&E-stained histopathological images at varying magnifications, the most likely diagnosis is:  Mucoepidermoid Carcinoma (MEC)  Key Microscopic Features Supporting Diagnosis:  1. Architecture and Growth Pattern (Low magnification - first image):  Lobulated tumor structure with fibrous septa separating nests/lobules.  Unencapsulated but circumscribed appearance.  The lesion shows solid and cystic regions, which is classic for MEC.  2. Cellular Composition (Medium magnification - second image):  Presence of mixed cell populations:  Epidermoid (squamoid) cells: polygonal cells with eosinophilic cytoplasm.  Mucin-producing cells: larger, pale/foamy cells with clear cytoplasm, often containing mucin vacuoles.  Intermediate cells: small to medium-sized cells with less cytoplasm, appearing more basaloid.  3. Mucin Production (High magnification - third image):  Numerous mucin-filled cells/vacuoles evident, giving a bubbly or vacuolated appearance.  Mucin appears pale/clear on H&E, often displacing the nucleus eccentrically.  Scattered epidermoid cells with prominent nucleoli.  High cellularity and mitotic activity is modest, suggestive of a low- to intermediate-grade MEC.  Conclusion:  These histologic features — the triphasic cell population, mucinous component, and solid-cystic architecture — are hallmark signs of mucoepidermoid carcinoma, the most common malignant salivary gland tumor. Grade determination (low, intermediate, or high) would require additional features like mitotic rate, necrosis, and neural invasion, but based on these images, a low- to intermediate-grade MEC is most consistent. | Histopathological diagnosis  Acinic cell carcinoma (low-grade) – predominantly microcystic / solid variant  Key microscopic features that support this diagnosis  Field of view Diagnostic finding Significance  Low power (first image) • Sharply circumscribed, lobulated, purple-blue mass separated by delicate fibrous septa  • Solid to microcystic architecture filling each lobule  • Foci of intralesional haemorrhage/degenerative change but no infiltrative edges A lobulated, well-outlined mass is typical of acinic cell carcinoma; microcystic pattern is one of its common growth patterns.  Intermediate power (second image) • Countless tiny round micro-cysts/pseudolumina appearing as empty white “holes” within solid sheets of tumour cells (“Swiss-cheese” look)  • Networks of delicate capillaries and thin fibrous strands between cell nests The microcystic pattern with minute lumina is characteristic of acinic cell carcinoma and contrasts with the hyaline-filled cribriform spaces of adenoid cystic carcinoma.  High power (third image) • Tumour cells show granular, amphophilic-to-basophilic cytoplasm packed with coarse eosinophilic/ basophilic zymogen-type granules  • Scattered cells with clear or vacuolated cytoplasm (vacuolated acinar cells)  • Uniform round nuclei, mild pleomorphism, inconspicuous nucleoli, scant mitoses Serous-acinar differentiation (granular basophilic cytoplasm with occasional clear/vacuolated cells) is pathognomonic for acinic cell carcinoma. The low-grade cytology helps distinguish it from high-grade salivary carcinomas.  Integrating the features  The combination of serous-acinar cytology (granular basophilic cytoplasm), innumerable minute micro-cysts, and a lobulated circumscribed growth pattern fits best with acinic cell carcinoma – specifically its microcystic/solid variant.  Lack of peripheral palisading or basement-membrane–like hyaline globules excludes basal cell or adenoid cystic carcinoma.  Absence of prominent mucin‐filled cysts and the presence of true acinar granules argue against mucoepidermoid or secretory (MASC) carcinoma.  Hence, the single most appropriate histopathological diagnosis is Acinic cell carcinoma of salivary gland, microcystic/solid pattern. |
| 13.43 | Diagnosis: Hyalinizing clear cell carcinoma; Key Microscopic Features: Clear cell islands within hyalinized stroma, infiltrative growth, with cells having clear cytoplasm and irregular nuclear outlines. | Based on the hematoxylin and eosin (H&E) stained histological images you provided, captured at multiple magnifications, the single most likely histopathological diagnosis is:  Pleomorphic Adenoma (Benign Mixed Tumor)  Key Microscopic Features Supporting the Diagnosis:  Biphasic Morphology (Epithelial and Mesenchymal Components):  The tumor exhibits a mixture of epithelial/myoepithelial cells and a stromal component.  Glandular structures and epithelial nests are embedded within a mesenchymal-like stroma, a hallmark of pleomorphic adenoma.  Ductal and Myoepithelial Differentiation:  Small duct-like structures lined by cuboidal to columnar epithelial cells.  Plasmacytoid (hyaline) myoepithelial cells with eosinophilic cytoplasm are seen in the stroma.  Chondromyxoid Stroma:  The stroma shows myxoid (mucoid) and chondroid (cartilage-like) areas, often pale and basophilic, typical of pleomorphic adenoma.  This is evident in the lower magnification images where swirling stromal areas with variable cellularity and texture are present.  Well-Circumscribed Borders:  Even in the low-power view, the tumor appears relatively well-circumscribed though non-encapsulated in minor salivary gland lesions (e.g., palate).  Overlying Mucosa Intact:  The overlying stratified squamous epithelium is intact and not ulcerated or infiltrated, supporting a benign nature.  Summary:  These features are diagnostic of Pleomorphic Adenoma, the most common benign salivary gland neoplasm. Its hallmark is morphological diversity, as seen here with epithelial structures in a myxochondroid stroma. | Most likely histopathological diagnosis  → Hyalinising Clear-Cell Carcinoma (HCCC) of minor salivary gland    (= “Clear-cell carcinoma, NOS”)  Key microscopic features visible in the provided H-E sections  Field What is seen How it supports HCCC  Overall architecture A poorly circumscribed, sub-mucosal mass just beneath intact surface epithelium; composed of interlacing cords, nests and long sweeping fascicles that form broad whorls and “target-like” swirls Typical infiltrative growth pattern of HCCC arising in minor salivary glands (palate, tongue, buccal mucosa)  Tumour cells Uniform polygonal / short-spindle cells with abundant optically clear cytoplasm (glycogen-laden) and small, round, slightly hyperchromatic nuclei; very little pleomorphism or mitotic activity The monotonous clear-cell cytology with scant atypia characterises HCCC and separates it from high-grade clear-cell tumours  Stroma Prominent dense eosinophilic, glassy (“hyalinised”) collagen separating and encasing the tumour cell cords; produces a striking pink background at low–medium power The combination of clear cells plus thick hyalinised collagen is the diagnostic hallmark for this entity (hence the name)  Growth pattern details (medium–high power) Tumour arranged in single-cell cords, thin trabeculae and small nests embedded in hyalinised matrix; occasional small tubular / duct-like spaces but no true ductal-luminal two-cell pattern, no chondromyxoid stroma, and no mucous cells Absence of the mixed epithelial-myoepithelial ductal structures of pleomorphic adenoma or EMC; lack of mucous / epidermoid cells argues against muco-epidermoid carcinoma  Interface / invasion Irregular tongues of tumour insinuating between normal salivary lobules and fibrous septa (best appreciated on the low-power panoramas) – a classic “sneaky” growth; perineural invasion is common in HCCC (not clearly captured here but often identified on deeper levels) Confirms a malignant, infiltrative salivary neoplasm; the bland cytology coupled with definite invasion is a typical diagnostic paradox of HCCC  Collectively, the bland clear-cell cytology, the conspicuous hyalinised collagenous stroma and the infiltrative cord-and-fascicle architecture beneath oral mucosa make hyalinising clear-cell carcinoma the single best fit for the lesion in these sections. |
| 13.44a | Diagnosis: Hyalinizing clear cell carcinoma; Key Microscopic Features: Tumor with basophilic and clear cells, featuring perineural invasion and pleomorphic cell characteristics. | Based on the three provided hematoxylin and eosin (H&E) stained histological images of a salivary gland neoplasm (shown at low, intermediate, and high magnification), the most likely diagnosis is:  Mucoepidermoid Carcinoma (MEC)  Key Microscopic Features Supporting This Diagnosis:  Low Power (Image 1):  Lobulated architecture with infiltration into surrounding stroma.  Mixture of solid and cystic areas.  Some glandular spaces filled with mucin are visible.  Intermediate Power (Image 2):  Heterogeneous cellular population.  Presence of both cystic and solid nests of cells.  Areas with mucous cells and intermediate-type cells are more distinguishable.  Mild to moderate lymphoplasmacytic infiltrate may be seen around the tumor.  High Power (Image 3):  Three distinct cell types:  Mucous cells: Large cells with foamy, pale cytoplasm (due to mucin content).  Epidermoid (squamoid) cells: Polygonal cells with eosinophilic cytoplasm and intercellular bridges.  Intermediate cells: Smaller, less differentiated cells with less cytoplasm.  Cellular pleomorphism and mitotic activity may be present, depending on grade.  Conclusion:  The combination of mucous-producing cells, intermediate cells, and epidermoid cells in a lobulated, partially cystic pattern is characteristic of mucoepidermoid carcinoma, which is the most common malignant salivary gland tumor. Grading (low, intermediate, or high) would depend on additional features such as cystic component proportion, neural invasion, mitoses, and necrosis. | Histopathological diagnosis  → Acinic cell carcinoma (low-grade), clear-cell–rich / microcystic variant  Key microscopic features supporting this diagnosis  Level of magnification What is seen Why it points to acinic cell carcinoma  Low power (first image) • Lobulated, well-circumscribed but un-encapsulated mass beneath intact oral mucosa.  • Lobules divided by delicate fibrous septa; mixture of solid and micro-cystic / follicle-like areas.  • No chondromyxoid stroma, papillary infoldings or cribriform architecture. A lobulated growth with solid ± microcystic pattern is classic for ACC; absence of features typical of pleomorphic adenoma, Warthin tumour, adenoid cystic carcinoma or mucoepidermoid carcinoma helps narrow the differential.  Intermediate power (second image) • Solid nests alternate with sieve-like microcystic spaces that contain pale eosinophilic material.  • Tumour pushes into surrounding stroma but retains a lobular outline. The mixed solid/microcystic architecture is one of the common growth patterns described for ACC.  High power (third image) • Two inter-mingled cell populations: ∘ Typical acinar cells – round, centrally placed nuclei and finely granular, basophilic to amphophilic cytoplasm (zymogen-type granules). ∘ Clear cells – abundant optically clear to vacuolated cytoplasm with centrally located small nuclei (glycogen-rich).  • Foci of tiny duct-like structures lined by cuboidal cells may be seen.  • Cells are cytologically bland, mitoses are scarce. • Granular basophilic cytoplasm and clear-cell change are hallmarks of serous acinar differentiation.  • Presence of both cell types in a low-grade cytologic setting strongly favours ACC over clear-cell mucoepidermoid carcinoma, epithelial-myoepithelial carcinoma, or other clear-cell salivary tumours.  Taken together, the lobulated solid/microcystic growth, the serous-acinar-type granular cells blended with numerous glycogen-rich clear cells, and the overall bland cytology constitute the classic histologic triad for a low-grade acinic cell carcinoma of salivary gland origin. |
| 13.44b | Diagnosis: Hyalinizing clear cell carcinoma; Key Microscopic Features: Tumor nests with pleomorphic nuclei and clear cytoplasm, with variation in nuclear appearance between different regions. | Based on the histopathological features visible in the provided hematoxylin and eosin (H&E) stained images of a salivary gland neoplasm, captured at different magnifications, the most likely diagnosis is:  Mucoepidermoid Carcinoma (MEC)  Key Microscopic Features Supporting the Diagnosis:  Low Magnification (First Image):  Lobulated architecture with solid and cystic spaces.  Cystic areas lined by neoplastic epithelial cells, suggestive of mucous-secreting components.  Tumor islands infiltrating surrounding stroma, a hallmark of malignancy.  High Magnification (Second Image):  Mixed cellular population including:  Mucous cells with clear/vacuolated cytoplasm.  Intermediate cells with polygonal shape and moderately eosinophilic cytoplasm.  Epidermoid (squamoid) cells, appearing more basophilic with intercellular bridges and nuclear pleomorphism.  Mitotic figures and nuclear atypia are present.  Mucin pools (though subtle), as indicated by vacuolated cytoplasm in some cells.  Conclusion:  The combination of mixed cell types, including mucous, intermediate, and epidermoid cells, arranged in solid and cystic growth patterns, along with evidence of invasion and cytologic atypia, is diagnostic of Mucoepidermoid Carcinoma, the most common malignant salivary gland tumor. | Most likely histopathological diagnosis  Muco-epidermoid carcinoma of salivary gland (intermediate-grade).  Key microscopic features seen in the two H&E fields that support this diagnosis  Architectural / Cytological feature Where it is seen in the pictures Diagnostic significance  Mixture of solid and cystic areas – rounded lobules with pale central lumina Low-power image (first slide) shows several well-demarcated lobules separated by fibrous septa; some lobules contain pale, empty-appearing cystic spaces A defining growth pattern of muco-epidermoid carcinoma, especially low–intermediate grade tumours  Heterogeneous cell population High-power image (second slide) Muco-epidermoid carcinoma is characterised by three inter-mingled cell types  • Mucous (goblet) cells: large, foamy, vacuolated cytoplasm that “pushes” the small basophilic nucleus to the periphery Upper left quadrant of the high-power field Confirms mucin-producing component  • Intermediate cells: small-to-medium basaloid cells with scant cytoplasm and round nuclei; predominate in the solid nests Present throughout both images, especially the lower half of the high-power field Constitute the bulk of intermediate-grade MEC  • Epidermoid / squamoid cells: cells with denser eosinophilic cytoplasm and more distinct cell borders, sometimes forming intercellular bridges Scattered among intermediate cells (seen toward the right upper area in high-power image) Third required cell type for MEC  Lack of a true capsule and infiltration into adjacent stroma The lobules are outlined by fibrous tissue but merge with surrounding stroma without a continuous capsule in the low-power field Helps separate MEC from encapsulated benign tumours (e.g., basal-cell adenoma)  Limited nuclear pleomorphism, occasional mitoses, no necrosis High-power image Fits an intermediate- (rather than high-) grade lesion  The combination of a cystic-solid architecture and the coexistence of mucous, intermediate and epidermoid cells is diagnostic for muco-epidermoid carcinoma among salivary-gland neoplasms of the oral and maxillofacial region. |
| 13.46 | Diagnosis: Mucinous adenocarcinoma; Key Microscopic Features: Cystic tumor with mucin pools, papillary structures, and pleomorphic tumor cells, forming a colloid carcinoma pattern. | Based on the histopathological analysis of the provided Hematoxylin and Eosin (H&E) stained images of a salivary gland neoplasm at varying magnifications, the most likely diagnosis is:  Adenoid Cystic Carcinoma (ACC)  Key Microscopic Features Supporting the Diagnosis:  Cribriform Pattern (Seen best in the first and second images):  Numerous “Swiss cheese”-like spaces formed by epithelial and myoepithelial cells.  The spaces are filled with basophilic mucoid or hyaline material, giving a pseudocystic appearance.  Dual Cell Population (Seen in all images):  A combination of ductal epithelial cells and basaloid myoepithelial cells is seen. The basaloid cells are hyperchromatic and form the cribriform and tubular structures.  Perineural Invasion (Best seen in the fourth image):  One of the hallmark features of ACC, visible as tumor cells tracking along or around nerve bundles.  High Nuclear-to-Cytoplasmic Ratio with Hyperchromatic Nuclei (Clearly evident in third and fifth images):  Tumor cells show scant cytoplasm, hyperchromatic, and often angulated nuclei, suggesting aggressive behavior.  Hyaline Stromal Material:  The eosinophilic, hyalinized stroma interspersed between tumor islands contributes to the classic histological picture, particularly in the cribriform areas.  Summary:  Adenoid cystic carcinoma is characterized by its unique cribriform architecture, dual cell population, and propensity for perineural invasion, all of which are distinctly visible in the provided images. This tumor is typically slow-growing but notorious for local recurrence and perineural spread, underlining the importance of early diagnosis and aggressive management. | Histopathological diagnosis — Muco-epidermoid carcinoma (intermediate-grade) of a salivary gland  Key microscopic features that point to this diagnosis  Image # Salient findings Diagnostic significance  1 (low-power overview) • Lobulated, infiltrative mass  • Predominantly cystic / cribriform spaces separated by fibrous septa Classic architecture of low- to intermediate-grade muco-epidermoid carcinoma (MEC): mixture of cystic and solid areas rather than a uniform solid sheet.  2 & 3 (medium/high power at cyst wall) • Tri-phenotypic cell population in the same focus:    - Mucous cells – large, pale/foamy cytoplasm with basally placed nuclei    - Epidermoid (squamoid) cells – polygonal cells with eosinophilic cytoplasm and inter-cellular bridges    - Intermediate cells – smaller basaloid cells with scant cytoplasm Simultaneous presence of mucous, epidermoid and intermediate cells is pathognomonic for MEC and rules out most other salivary tumours.  2 & 4 • Pools / lakes of extracellular mucin with floating tumour cells Extravasated mucin is common in MEC and helps distinguish it from adenoid cystic carcinoma (which shows hyaline cylinders, not mucin).  3 • Foci of squamoid differentiation without overt keratin pearls The squamoid component supplies the “epidermoid” element of MEC.  4 • Infiltration of surrounding fibrous & fatty stroma; absence of chondromyxoid stroma Confirms malignancy and excludes pleomorphic adenoma.  5 (solid areas) • Sheets/whorls of intermediate basaloid cells with scattered mucous vacuoles; mild-to-moderate atypia Solid nests of intermediate cells indicate at least an intermediate-grade MEC.  Integrative reasoning  The combination of cystic architecture + mucin-filled lumina/lakes and a mixture of mucous, epidermoid and intermediate cells is virtually diagnostic of muco-epidermoid carcinoma.  Absence of chondromyxoid (pleomorphic adenoma), granular serous acini (acinic cell carcinoma), or hyaline cylinders with small basaloid cells (adenoid cystic carcinoma) further supports MEC.  The relative proportion of cystic to solid areas and the moderate cytologic atypia suggest an intermediate-grade lesion, although grading was not specifically requested.  These microscopic features collectively establish muco-epidermoid carcinoma as the single most appropriate histopathological diagnosis for the lesion. |
| 13.47 | Diagnosis: Microsecretory carcinoma of the lip; Key Microscopic Features: Invasive tumor with microcystic ducts, desmoplasia, and osseous metaplasia, lined by a low cuboidal epithelium. | Histopathological Diagnosis:  Adenoid Cystic Carcinoma (ACC)  Key Microscopic Features Supporting the Diagnosis:  Cribriform Pattern ("Swiss cheese" appearance):  The middle and high-power images (especially the second one) show numerous basaloid epithelial cells forming well-defined pseudocystic spaces filled with eosinophilic, mucoid, or hyaline material.  This hallmark pattern is a defining feature of the cribriform subtype of ACC.  Basaloid Cell Population:  The epithelial cells are small, hyperchromatic, and basaloid with scant cytoplasm and angulated nuclei—typical of ACC.  Perineural Invasion (Possible):  Though not clearly confirmed in the images, ACC often exhibits perineural invasion, which is a key histological and clinical feature. You may consider looking further in the sample for nerve structures with tumor wrapping.  Fibrous Stroma:  The tumor is set within a hyalinized or fibrous stroma, providing a dense collagenous background as seen in all three images.  Tubular and Cribriform Areas:  The low- and medium-magnification images show tubular structures in addition to the cribriform pattern, suggesting a mixed architectural pattern often seen in ACC.  Differential Considerations (Ruled Out Based on Features):  Polymorphous Adenocarcinoma: Typically shows more cytologic uniformity and lacks the cribriform pattern with pseudocysts filled with hyaline material.  Basal Cell Adenoma/Carcinoma: Less aggressive architecture, lacking cribriform areas and hyaline material.  Pleomorphic Adenoma: Would display chondromyxoid stroma and ductal/myoepithelial elements in a more mixed architecture. | Histopathological diagnosis  Pleomorphic adenoma (benign mixed tumour) of a minor salivary gland  Key microscopic features visible in the three fields  Feature Where it is seen Diagnostic significance  Well-circumscribed, lobulated nodule surrounded by compressed fibrous tissue Low-power overview (image 1) Typical architecture of a benign pleomorphic adenoma  Biphasic epithelial component: numerous small round-to-oval ducts/tubules lined by an inner cuboidal ductal layer and an outer flattened/attenuated myoepithelial layer Intermediate & high-power fields (images 2 & 3) Classic dual-cell lining characteristic of pleomorphic adenoma  Plasmacytoid / spindle myoepithelial cells dispersed in the stroma and forming cords around ducts High-power (image 3, dark basophilic nuclei around the ductal spaces) Confirms myoepithelial participation in the tumour  Variable stromal differentiation: hyalinised and cartilaginous (chondroid) matrix as dense eosinophilic, glassy islands; adjacent areas show myxoid change Low- and intermediate-power (image 1 central pink islands; image 2 bottom left) The admixture of epithelial elements with chondromyxoid stroma is the hallmark “mixed” pattern of pleomorphic adenoma  Absence of significant cytologic atypia, mitoses or infiltrative borders All images Supports a benign rather than malignant salivary gland neoplasm  The combination of a circumscribed lesion composed of dual-layered ductal structures intimately admixed with myoepithelial cells embedded in a myxoid-to-chondroid stroma is diagnostic of pleomorphic adenoma. |
| 13.48 | Diagnosis: Sclerosing microcystic adenocarcinoma of the lip; Key Microscopic Features: Tumor islands with eosinophilic secretions and hyalinization, with minimal cytologic atypia. | Based on the histopathological analysis of the three H&E-stained images provided, all showing the same lesion in the salivary gland, the single most likely diagnosis is:  Adenoid Cystic Carcinoma (ACC)  Key Microscopic Features Supporting the Diagnosis:  Cribriform ("Swiss Cheese") Pattern:  Clearly visible in the second and third images, especially at higher magnification.  Characterized by epithelial and myoepithelial tumor cells forming pseudocystic spaces filled with basophilic mucinous or hyaline material.  Basaloid Tumor Cells:  The cells are small, with hyperchromatic nuclei and scant cytoplasm.  Seen prominently in the third image.  Perineural Invasion:  Evident in the second image (arrows pointing to nerve structures surrounded by tumor cells), which is a hallmark of ACC.  ACC is notorious for its tendency to invade nerves, leading to potential pain and recurrence.  Dense Hyalinized Stroma:  Present in all images, especially the first and second, showing an infiltrative growth within a fibrotic or hyalinized stroma.  This is typical of ACC and aids in differentiating it from more cellular neoplasms like polymorphous adenocarcinoma.  Infiltrative Growth Pattern:  The lesion infiltrates the surrounding salivary gland tissue, with irregular borders and disruption of normal architecture.  Summary:  These features—particularly the cribriform pattern, perineural invasion, and basaloid cytology—strongly support the diagnosis of Adenoid Cystic Carcinoma, a malignant salivary gland tumor with characteristic histologic architecture and clinical behavior. | Most likely histopathological diagnosis  → Adenoid cystic carcinoma (ACC) – predominantly tubular/cribriform pattern  Microscopic features in the three fields that point to ACC  Field What is seen Why it supports ACC  Low-power (image 1) • Infiltrative, un-encapsulated mass just beneath the surface mucosa  • Dense, eosinophilic/hyalinised stroma criss-crossed by slender cords and tiny circular profiles A benign mixed tumour (pleomorphic adenoma) would be well-circumscribed or encapsulated and show large myxoid/chondroid areas; the infiltrative outline and uniformly sclerotic stroma favour a carcinoma, classically ACC.  Intermediate power (image 2) • Countless small true ducts and occasional “Swiss-cheese” cribriform islands scattered through a markedly desmoplastic background  • Dual cell population in most ducts (inner eosinophilic luminal cell layer, outer basaloid/myoepithelial layer)  • Several nerves/vessels encircled by tumour (arrows) A dual cell, tubular-cribriform architecture with basement-membrane-like hyaline cylinders and a penchant for perineural growth is the signature of ACC.  High-power (image 3) • Tubules composed of two concentric layers: pale cytoplasmic luminal cells and an abluminal rim of small, hyperchromatic basaloid nuclei  • Central lumina often contain pale mucinous/hyaline material  • Nuclei are angulated, hyperchromatic, but fairly uniform; mitoses scarce The orderly two-cell tubules with hyaline luminal content and dark basaloid outer cells are classic for the tubular pattern of ACC; the cytology is too basaloid for polymorphous adenocarcinoma and too monotonous for mucoepidermoid carcinoma.  Key diagnostic hallmarks present in these slides  Tubular + cribriform growth with dual cell layers  Prominent basement-membrane-like (hyaline) material around and within ducts  Dense, sclerosing stroma and lack of a capsule (infiltrative borders)  Perineural/vascular tropism (structures being hugged by tumour cells)  Uniform basaloid nuclei with scant cytoplasm, low mitotic rate  Taken together, these features are pathognomonic for adenoid cystic carcinoma of salivary gland origin, most closely matching its tubular/cribriform variant seen in minor salivary glands of the oral cavity and palate. |
| 13.49 | Diagnosis: Salivary duct carcinoma; Key Microscopic Features: Tumor islands with comedonecrosis and intraluminal proliferation forming cribriform patterns, along with pleomorphic tumor cells. | Based on the hematoxylin and eosin (H&E) stained images provided at progressively higher magnifications, the most likely histopathological diagnosis is:  Acinic Cell Carcinoma (ACC) of the salivary gland.  Justification and Key Microscopic Features:  Architecture:  The low-power images show well-circumscribed but unencapsulated lobulated growth, characteristic of low-grade malignant salivary gland neoplasms.  The tumor is composed of solid, microcystic, and follicular growth patterns, common in acinic cell carcinoma.  Cellular Features:  Polygonal tumor cells with abundant granular basophilic cytoplasm are evident, consistent with serous acinar cell differentiation.  Nuclei are round to oval, relatively uniform, and eccentrically located—typical of ACC.  Presence of zymogen granules (appearing as cytoplasmic granularity) is a hallmark feature at higher magnification.  Cytoplasmic Features:  At higher magnification, there is a distinct granular cytoplasm, which corresponds to PAS-positive, diastase-resistant granules, a hallmark of zymogen granules seen in serous acinar cells.  Lack of High-Grade Features:  No significant pleomorphism, necrosis, or frequent mitotic figures, supporting the low-grade malignancy classification typical of ACC.  Summary:  The described histological features—particularly the serous acinar cell differentiation with basophilic granular cytoplasm, lobulated architecture, and lack of high-grade atypia—strongly support the diagnosis of Acinic Cell Carcinoma, a malignant salivary gland tumor most commonly arising in the parotid gland. | Histopathological diagnosis  Muco-epidermoid carcinoma – low-grade  Key microscopic features that support this diagnosis  Field What is seen Why it points to muco-epidermoid carcinoma  Low-power architecture (1st & 2nd images) Tumour is lobulated and infiltrative, divided by fibrous septa into multiple, variably-sized cystic and microcystic spaces that are filled with pale-pink to eosinophilic secretions A predominantly cystic pattern with only thin ribbons or nests of solid tumour is characteristic of low-grade muco-epidermoid carcinoma  Cell population (3rd & 4th images) Mixed population of mucous cells (large cells with optically clear/foamy vacuolated cytoplasm ± “signet-ring” appearance), intermediate cells (smaller polygonal cells with lightly basophilic granular cytoplasm) and scattered epidermoid/squamoid cells The tri-phenotypic mixture of mucous + intermediate + epidermoid cells is the diagnostic hallmark of muco-epidermoid carcinoma  Luminal / intracellular mucin Cyst lumina and many tumour cells contain slick, pale mucin; mucous cells show large intracytoplasmic mucin vacuoles displacing nuclei Abundant mucin production is typical of low-grade lesions and helps separate MEC from other salivary tumours such as acinic-cell or secretory carcinoma  Cytologic grade Nuclei are round-to-oval with smooth contours, delicate chromatin and inconspicuous nucleoli; mitoses and pleomorphism are scarce These bland cytologic features and the overwhelmingly cystic growth pattern place the tumour in the low-grade category  Growth pattern Tumour islands dissect through salivary gland stroma without a true capsule, but with minimal destructive invasion Infiltrative growth confirms malignancy and rules out benign cystadenoma while the limited stromal reaction and low atypia still indicate a low-grade behaviour  Together, the lobulated multi-cystic architecture, admixture of mucous, intermediate and epidermoid cells, and low-grade cytology establish the diagnosis of low-grade muco-epidermoid carcinoma of salivary gland origin. |
| 13.50 | Diagnosis: Adenocarcinoma, not otherwise specified; Key Microscopic Features: Tumor cells with duct formation and moderate cytoplasm, infiltrating the salivary glands. | Based on the provided hematoxylin-and-eosin (H&E) stained histopathological images at different magnifications, the single most likely diagnosis is:  Acinic Cell Carcinoma (ACC) of the Salivary Gland  Key Microscopic Features Supporting the Diagnosis:  Lobular Growth Pattern:  Seen in the low-power image (first image), the tumor displays a well-organized lobular architecture separated by fibrous septa.  Uniform Cells with Granular Cytoplasm:  At higher magnification (second image), the neoplastic cells show abundant, basophilic to amphophilic granular cytoplasm. These granules represent zymogen granules, a hallmark of serous acinar cell differentiation.  Round, Centrally Placed Nuclei:  The nuclei are generally round, centrally located, and show minimal pleomorphism or atypia, consistent with a low-grade malignancy.  Clear Cell and Vacuolated Appearance in Some Areas:  Some areas show vacuolated cytoplasm or cells with a clear appearance, a variant often seen in acinic cell carcinoma.  Lack of Ductal Differentiation:  There’s minimal ductal or myoepithelial cell differentiation, differentiating it from other salivary gland tumors like mucoepidermoid carcinoma or pleomorphic adenoma.  Summary:  These histologic features — especially the presence of zymogen granules in a lobular arrangement with uniform round nuclei — are classic for acinic cell carcinoma, a low-grade malignant salivary gland tumor most commonly arising in the parotid gland. | Histopathological diagnosis  Acinic cell carcinoma (low-grade malignancy).  Key microscopic features supporting this diagnosis  Field What is seen Why it points to acinic cell carcinoma  Overall architecture (low-power image) • A lobulated, yet unencapsulated mass that replaces the normal gland.  • Lobules/nests are outlined by delicate fibrous septa (“alveolar” pattern). A lobular growth with thin septa is characteristic of the solid/alveolar variant of acinic cell carcinoma.  Cell arrangement • Within each lobule the cells are packed in solid sheets, with scattered micro-cystic spaces containing pale eosinophilic material. Solid and micro-cystic patterns are two of the classic architectural patterns described for this tumour (solid › microcystic › papillary-cystic › follicular).  Cytoplasm • Cells have abundant, finely granular, lightly basophilic / amphophilic cytoplasm; scattered cells show small cytoplasmic vacuoles. The fine cytoplasmic granularity represents zymogen granules of serous acinar differentiation, the defining feature of acinic cell carcinoma.  Nuclei • Round-to-oval, central nuclei, finely speckled (“salt-and-pepper”) chromatin, inconspicuous nucleoli, minimal pleomorphism and scant mitotic activity. Nuclear blandness with low-grade cytology distinguishes acinic cell carcinoma from higher-grade salivary tumours.  Stroma / background • No chondromyxoid matrix (rules out pleomorphic adenoma).  • No cribriform “Swiss-cheese” spaces with hyaline cylinders (rules out adenoid cystic carcinoma).  • No mixture of epidermoid, intermediate and mucous cells (rules out mucoepidermoid carcinoma). The absence of these other signature stromal or cellular components helps narrow the differential to acinic cell carcinoma.  Taken together—a lobulated but infiltrative tumour composed of uniform cells showing granular serous-acinar cytoplasm arranged mainly in a solid/alveolar pattern—pinpoints acinic cell carcinoma as the single most likely diagnosis. |

ODONTOGENIC CYSTS

| **Fig_no** | **Ground truth** | **GPT 4o answer** | **o3 answer** |
| --- | --- | --- | --- |
| 14.5 | **Diagnosis**: Apical Radicular Cyst; **Key Microscopic Features**: The cyst shows epithelial proliferation and inflammation. The lining consists of nonkeratinized stratified squamous epithelium with plexiform proliferation, spongiosis, and neutrophilic transmigration. The epithelium may vary in thickness, and long-standing lesions may show uniformly thin lining. | The images you have uploaded show histological slides that fall under the category of odontogenic cysts in oral and maxillofacial pathology, with specific features visible under different magnifications.  Diagnosis:  The most likely diagnosis based on the histopathological features seen in the images is Dentigerous Cyst.  Key Microscopic Features:  Cystic Structure with Lining Epithelium:  The cystic lesion is lined by a thin layer of epithelium, which is characteristic of a dentigerous cyst. The epithelium is typically non-keratinized and presents as a simple squamous to cuboidal epithelium.  Fibrous Connective Tissue Capsule:  The cyst is surrounded by a thick fibrous connective tissue capsule, which is seen in these images. This feature is often observed in dentigerous cysts.  Presence of Inflammation:  In the second and fourth images, there is an apparent inflammatory response with lymphocytic infiltration. This is a common feature in dentigerous cysts, although it is not a definitive feature for the diagnosis.  Peripheral Odontogenic Epithelium:  In some images, there is a suggestion of odontogenic epithelium at the periphery, which is often seen in dentigerous cysts, as it can sometimes be associated with the tooth bud.  No Signs of Malignant Transformation:  There are no signs of malignancy, such as atypical cells or invasive behavior, which supports the diagnosis of a benign odontogenic cyst.  These histopathological features are consistent with a Dentigerous Cyst, which is often found associated with an unerupted or partially erupted tooth, typically a third molar or canine. | Based on the images provided and the context of odontogenic cysts, it seems likely that the lesion in question is a Keratocystic Odontogenic Tumor (KCOT), which is also known as the Keratocystic Odontogenic Cyst (KOT) in some classifications.  Key microscopic features that justify this diagnosis:  Stratified Squamous Epithelium:  The images show a lining of stratified squamous epithelium, which is characteristic of many odontogenic cysts, including the KCOT.  Parakeratinized Epithelium:  The epithelial lining is parakeratinized in parts, which is an important histopathological feature of KCOT. The parakeratinized layer indicates active keratinization.  Thin Epithelium:  The epithelium appears thin, with a prominent basal cell layer, a typical feature of KCOT. The basal cells are often columnar or cuboidal, and this is evident in the images.  Cystic Lumen:  The lumen of the cyst appears filled with keratin debris, which is often seen in KCOT.  Infiltration and Lobulated Growth:  Some of the cysts appear to have a lobulated configuration and may show some evidence of epithelial proliferation at the edges, which can sometimes be seen in KCOTs.  Potential for Aggressive Behavior:  KCOTs have the characteristic of aggressive local behavior with a tendency to recur after surgical treatment, though the images themselves do not necessarily show evidence of recurrence.  These histopathological features, when combined, support the diagnosis of a Keratinized Odontogenic Cyst (Keratocystic Odontogenic Tumor), known for its unique epithelium and aggressive nature. |
| 14.5b | **Diagnosis**: Apical Radicular Cyst; **Key Microscopic Features**: The cyst is lined by respiratory epithelium at the apex of the maxillary first molar and partially features pseudostratified, ciliated columnar epithelium without mucous glands. | The two histopathological images you've provided show a lesion that fits into the category of odontogenic cysts. Based on their appearance, the diagnosis is most likely an odontogenic keratocyst (OKC).  Key Microscopic Features:  Cystic Structure:  Both images demonstrate a prominent cystic space surrounded by a well-defined epithelium. In the first image, the cystic area is clearly visible, which is typical of OKC, often characterized by a well-demarcated, fluid-filled cavity.  Keratinized Stratified Squamous Epithelium:  The lining of the cyst is a thin, hyperplastic layer of stratified squamous epithelium, with keratinization evident in the first image. This feature is critical for OKC and differentiates it from other types of odontogenic cysts, such as the dentigerous cyst or periapical cyst, which have non-keratinized linings.  Proliferative Basal Cell Layer:  The basal cell layer of the epithelium is hypercellular and exhibits a palissading arrangement of cells, often seen in OKCs. The basal cells are columnar, and there is an evident pattern of basal cell hyperplasia, as seen in both images.  Thin Cyst Wall:  The surrounding cyst wall is relatively thin but well-defined, which is a hallmark of OKC. Unlike other cysts, the wall of an OKC is more prone to expansion and growth without significant inflammation or fibrosis.  Lack of Inflammatory Cells:  There is no significant presence of inflammatory cells in the cystic space, which distinguishes OKCs from other cysts like periapical cysts, where inflammation is typically more prominent.  Diagnosis: Odontogenic Keratocyst (OKC)  OKC is a cyst of odontogenic origin, often associated with the mandibular and maxillary regions. It tends to be more aggressive than other odontogenic cysts and has a higher rate of recurrence, making its identification and diagnosis particularly important in clinical practice. | Based on the histopathological images provided, the diagnosis most likely corresponds to an odontogenic keratocyst (OKC), a type of odontogenic cyst in oral and maxillofacial pathology.  Key microscopic features that justify the diagnosis:  Image 1 (Low Magnification):  Cystic structure with keratinized lining: The lesion shows a cystic cavity with a thin epithelial lining, some areas of which appear keratinized. This is a hallmark feature of an odontogenic keratocyst (OKC).  Uniform thickness of epithelial lining: The epithelial lining appears to be fairly consistent in thickness, which is characteristic of an OKC.  Cystic lumen with a thick, keratinized layer: The lumen of the cyst shows evidence of keratin accumulation, a feature typical of OKC, as the cysts often contain keratin in their cavities.  Image 2 (High Magnification):  Stratified squamous epithelium with a basal cell layer: The epithelium is composed of a thin layer of stratified squamous cells, and the basal layer is hyperplastic, showing the typical palisading pattern seen in OKC.  Corrugated or hyperplastic epithelium: The basal layer shows prominent palisading of columnar or cuboidal cells, with a corrugated or basal layer of epithelium that is characteristic of the OKC.  Minimal inflammation or fibrosis: There is little to no chronic inflammatory infiltrate or fibrosis in the surrounding tissue, which is another typical feature of OKC.  Conclusion:  The combination of cystic features with keratinized epithelial lining, uniform epithelium thickness, palisading basal cells, and a lack of significant inflammatory response is strongly indicative of an odontogenic keratocyst. |
| 14.6 | **Diagnosis**: Sinus Inflammatory Polyp; **Key Microscopic Features**: Radiograph shows a dome-shaped opacity next to the carious maxillary molars. The mass is composed of pale eosinophilic material, granulation tissue covered by respiratory epithelium without mucous glands, and includes many plasma cells and eosinophils. | The images you uploaded show a lesion of odontogenic origin. Based on the histological features and typical presentations of odontogenic cysts in oral and maxillofacial pathology, the most likely diagnosis is Keratocystic Odontogenic Tumor (KCOT), also known as the Odontogenic Keratocyst (OKC).  Key Microscopic Features:  Cyst Lining:  The cyst is lined by stratified squamous epithelium, which appears thin, with perpendicular basal cell layers.  The epithelium may show parakeratinization and thickened epithelial layers at some points.  Epithelial Characteristics:  The basal layer of the epithelium demonstrates a palisading arrangement of cells, with some degree of hyperchromatic nuclei.  There is a tendency for the epithelial cells to show keratinization, leading to a keratinized cyst lumen.  Cyst Wall:  The cystic lesion often shows a thin fibrous capsule with minimal inflammation.  The cyst wall exhibits loose connective tissue, with a lack of significant inflammatory infiltrate.  Clear Separation from Surrounding Tissue:  The lesion is typically well-demarcated from surrounding bone or soft tissues, as seen in the images where the lesion does not invade aggressively.  These histopathological features align well with the Keratocystic Odontogenic Tumor (KCOT) diagnosis, characterized by the unique epithelial features mentioned. | The images you provided show histopathological slides of an odontogenic lesion. After reviewing the images, the following diagnosis and analysis are provided:  Diagnosis: Ameloblastoma  Key Microscopic Features:  Proliferation of epithelial cells: In all three images, the lesion demonstrates a proliferation of odontogenic epithelium arranged in islands or cords. This is a characteristic feature of ameloblastoma. The cells within these islands appear hyperchromatic, with clear, prominent nuclei.  Peripheral columnar cells with a palisading pattern: In the first image, peripheral cells of the epithelial islands show a columnar arrangement with a palisading pattern, often seen in ameloblastomas. This is a classic histological finding in this tumor type.  Nuclear hyperchromasia and pleomorphism: There is marked nuclear pleomorphism (variations in size and shape of the nuclei) in the epithelial cells, especially towards the center of the islands.  Chondromyxoid stroma: The stroma in the deeper parts of the lesion (visible in the second and third images) appears myxoid or cartilaginous, which is common in certain variants of ameloblastoma.  Presence of cyst-like spaces: The lesion shows cystic areas surrounded by epithelium, which is consistent with the multicystic type of ameloblastoma.  These microscopic findings of epithelial islands with peripheral palisading, nuclear pleomorphism, and myxoid stroma, along with the cyst-like spaces, confirm the diagnosis of ameloblastoma.  Let me know if you need further details on this diagnosis! |
| 14.7 | **Diagnosis**: Apical Radicular Cyst with Squamous Odontogenic Tumor-Like Proliferation; **Key Microscopic Features**: Irregular islands of epithelium with dyskeratotic cells lacking palisading peripheral cells. | The images provided show a lesion with typical features that point toward a diagnosis within the odontogenic cysts group, specifically Dentigerous Cyst (also known as a Follicular Cyst). Below are the key aspects of the histopathological features observed in the images and how they support this diagnosis:  Single Most Likely Diagnosis:  Dentigerous Cyst (Follicular Cyst).  Key Microscopic Features Supporting the Diagnosis:  Cystic Lesion with a Thick Fibrous Capsule:  In both images, there is a cystic structure surrounded by a thick, dense fibrous wall. This is characteristic of the dentigerous cyst.  Presence of an Odontogenic Epithelium Lining:  The cyst is lined by a layer of odontogenic epithelium, which appears thin and flattened. In some areas, this lining is squamous or cuboidal. This feature is a typical hallmark of dentigerous cysts.  Association with a Developing Tooth:  The cyst is often associated with the crown of an unerupted or impacted tooth. In the first image, there is evidence of epithelial lining around what appears to be the crown of an unerupted tooth, which supports this diagnosis.  Thickened Epithelium:  The epithelium may show areas of hyperplasia or thickening, though not always significant, which can be seen in the second image with slight cellular density or small foci of cell proliferation.  Presence of the Luminal Content:  The cystic lumen often contains clear or viscous fluid, though this is harder to identify directly on H&E without special staining. However, the cystic space devoid of structures in the middle of the images hints at the fluid-filled nature.  These features, seen across the two different magnifications, help confirm the diagnosis of a Dentigerous Cyst. | Based on the provided hematoxylin-and-eosin images from the odontogenic cysts group of oral and maxillofacial pathology, the most likely histopathological diagnosis is:  Dentigerous Cyst (also known as Follicular Cyst)  Key Microscopic Features Seen in the Images:  Enclosed Follicular Epithelium: The lesion is characterized by a cystic structure surrounding an impacted tooth or tooth crown, which is typical of a dentigerous cyst. The cystic lining is made of a thin layer of non-keratinized stratified squamous epithelium.  Columnar or Cuboidal Epithelium: The epithelial lining consists of relatively thin, flat or cuboidal cells, which are seen in both images, often with a low cuboidal appearance.  Inflammatory Cells: The presence of mild lymphocytic infiltration in the cyst wall, as observed in these images, is consistent with the inflammatory reaction commonly seen in odontogenic cysts.  Tooth Crown: The cyst is typically associated with the crown of an unerupted or partially erupted tooth, as suggested by the proximity of cystic spaces around the crown in the first image.  Keratinization in Some Areas: While the majority of the cystic lining is non-keratinized, small patches of keratinized epithelium may be observed, which are not typical but still present in some dentigerous cysts.  These histopathological features align with the diagnosis of a dentigerous cyst, commonly associated with the crown of an unerupted or impacted tooth. |
| 14.10 | **Diagnosis**: Hyaline Ring Granulomas; **Key Microscopic Features**: Hyalinized rings, often containing giant cells at the center, calcifications, foreign material, and dystrophic calcifications. Russell bodies and pyronine bodies are frequently present. | Based on the histopathological images you provided, the diagnosis for the lesion is most likely Ameloblastoma in the context of odontogenic cysts.  Key Microscopic Features Observed:  Cystic Spaces with Epithelial Lining:  The cystic spaces present in the images suggest an odontogenic origin, which is a characteristic feature of odontogenic cysts, such as the Ameloblastoma.  Peripheral Layer of Columnar Cells:  The cystic epithelium demonstrates a layer of columnar or cuboidal cells along the lining, with some areas showing palisading, which is a key feature of ameloblastomatous epithelium.  Pleomorphic Epithelial Cells:  The epithelial cells lining the cysts show a variable appearance, which is a hallmark of ameloblastoma. Some cells have a hyperchromatic nucleus, and this pleomorphism in the cyst lining points to the potential for this diagnosis.  Chronic Inflammatory Cells:  The presence of chronic inflammatory cells in the stroma surrounding the cysts, as well as some areas of hyalinization and fibrosis, is common in ameloblastomas and some other odontogenic cysts, as the surrounding tissue often undergoes reactive changes.  Mucous or Serous Cystic Fluid:  In some sections, areas of mucous or serous fluid accumulation are seen, which can further suggest an odontogenic cystic lesion, like the ameloblastoma or a dentigerous cyst.  These features, especially the epithelial changes with palisading and the cystic nature of the lesion, strongly support the diagnosis of Ameloblastoma, likely the unicystic variant, which often presents with such features. | Based on the histological images provided, the diagnosis is most likely Keratinizing Odontogenic Cyst (KOC), also known as Gorlin's cyst. Here's the analysis and justification for this diagnosis based on key microscopic features:  Key Histopathological Features:  Cyst Lining:  In the first and second images, the cyst lining is composed of stratified squamous epithelium that demonstrates keratinization. This is a characteristic feature of a keratinizing odontogenic cyst (KOC).  The presence of keratin-filled lumina is evident in these images, particularly the white, keratinous material within the cyst lumen.  Epithelial Arrangement:  The lining epithelium shows a thickened layer and exhibits palissading cells along the basal layer. This is seen in the third image where the cells in the basal layer are columnar or cuboidal and align in a palisaded fashion.  Cyst Wall:  The cyst wall consists of a relatively thin fibrous connective tissue that is seen in all three images. This fibrous tissue may show minimal inflammation or slight vascularization.  Surrounding Tissues:  There are areas of granulomatous inflammation with a mixed inflammatory infiltrate, including lymphocytes and macrophages, which can be noted in the third image.  The presence of odontogenic epithelial remnants (e.g., islands of epithelium) can sometimes be seen, though not always prominent in all samples.  Conclusion:  The combination of a keratinized epithelium, palisaded basal layer, and the keratin-filled lumen within the cyst lumen are strong indicators of a keratinizing odontogenic cyst (KOC).  This diagnosis fits the provided histology images, which highlight the key features of a KOC. |
| 14.11 | **Diagnosis**: Periapical Granuloma; **Key Microscopic Features**: Granulation tissue surrounds a cyst-like cavity with acute and chronic inflammatory cells, but no lining epithelium. | Based on the provided hematoxylin and eosin (H&E) stained histopathological images, the following analysis can be made:  Histopathological Diagnosis:  Odontogenic Keratocyst (OKC)  Key Microscopic Features:  Cystic Architecture:  The first image shows a well-defined cystic cavity with thin walls. This appearance is characteristic of an odontogenic cyst, particularly an odontogenic keratocyst (OKC).  Keratinized Epithelium:  In the second image, the epithelium lining the cyst is stratified and exhibits a prominent basal layer with columnar cells, characteristic of OKC.  The epithelial cells at the basal layer show palisading, which is another hallmark of OKC. This means the cells are arranged in a columnar fashion, which is distinct compared to other odontogenic cysts.  Parakeratinization:  The cyst lining often exhibits parakeratinization, which means the surface of the epithelium is keratinized but with retained nuclei in the keratinized layer. This is a distinguishing feature of OKC.  Thin, Uniform Epithelium:  The epithelial lining is typically thin and uniform, which is seen in the first image. The cyst wall doesn't show significant inflammation or thickening, further supporting the diagnosis of OKC.  No Significant Inflammation:  There is an absence of chronic inflammatory cells within the cystic walls, which is consistent with OKC. The lack of inflammation helps to differentiate it from other odontogenic cysts that may show more chronic inflammatory cells.  These features strongly support the diagnosis of an odontogenic keratocyst (OKC), a relatively aggressive cystic lesion that is associated with an increased risk of recurrence if not properly treated. | Based on the images provided, I can provide a histopathological diagnosis and analysis of the lesion from the odontogenic cyst group.  Histopathological Diagnosis:  The most likely diagnosis for the lesion depicted in the images is Keratocystic Odontogenic Tumor (KCOT), which is a type of odontogenic cyst.  Key Microscopic Features:  Cyst Lining:  In the first image, we can see a well-defined cystic structure lined by a thin, stratified squamous epithelium. This epithelium shows a distinct, thickened, parakeratinized appearance, which is a characteristic feature of KCOT.  Cellular Proliferation:  The basal cell layer of the epithelium is hyperchromatic and palisaded, which is another hallmark of the keratocystic odontogenic tumor.  Cyst Wall:  The cyst wall appears relatively thin and contains minimal connective tissue. This is typical of KCOT, as the cyst wall is often sparse compared to other cystic lesions of the jaw.  Chronic Inflammation:  The second image shows areas of chronic inflammation and fibrous connective tissue, suggesting ongoing inflammation in the cyst's surrounding environment, which is common in KCOT due to its tendency to recur and cause irritation.  These features, combined with the aggressive nature and recurrence potential of KCOT, strongly support this diagnosis. |
| 14.13 | **Diagnosis**: Periapical Abscess; **Key Microscopic Features**: Granulation tissue containing numerous abscesses. Reactive woven bone surrounded by plump osteoblasts is often observed in periapical lesions. | Based on the histopathological images you have provided, here’s the analysis for the lesion in the context of odontogenic cysts:  Single Most Likely Histopathological Diagnosis:  Ameloblastoma  Key Microscopic Features:  Image 1:  Hypercellular lesion: The first image displays a densely packed area with numerous small, round cells, which are typical of the highly cellular nature of ameloblastomas.  Peripheral palisading: The columnar or cuboidal cells arranged along the periphery of the cysts are a hallmark feature of the follicular variant of ameloblastoma.  Stroma: There appears to be a connective tissue stroma with a loose arrangement, which is characteristic of this lesion.  Image 2:  Stromal breakdown and fibrous tissue: The second image shows a more fibrous stroma, indicating progression towards the more solid, desmoplastic form of ameloblastoma.  Irregular cystic spaces: You can observe some cystic or cleft-like areas surrounded by the basal cell layer, which are typically seen in ameloblastoma.  Cellular pleomorphism: There is noticeable pleomorphism in the nuclei of the basal cells, which is a common feature of ameloblastomas and contributes to its aggressive nature.  These features — including the palisading of basal cells, cystic spaces, and dense stroma — strongly support the diagnosis of ameloblastoma, a benign but locally aggressive odontogenic tumor. | The images you've provided seem to show histological features of an odontogenic cyst. Based on the images, here is the analysis:  Most Likely Histopathological Diagnosis:  Dentigerous Cyst (also known as a follicular cyst).  Key Microscopic Features:  Image 1:  The cystic lining is seen to be composed of a single layer of cuboidal to columnar epithelium.  The epithelium is often flattened and sometimes exhibits a slight degree of hyperplasia.  There is a prominent presence of connective tissue (fibrous wall) around the cyst cavity.  The lesion appears to be associated with an impacted tooth, which is characteristic of a dentigerous cyst.  Image 2:  The cyst wall is more detailed, with dense fibrous connective tissue and areas showing slight inflammation.  The cyst lumen is filled with clear fluid, and the surrounding connective tissue is more prominent.  There is often a reactive bone formation or sclerosis around the cyst, further supporting the diagnosis of dentigerous cyst.  The epithelium lining is relatively thin and flattened, a classic feature of a dentigerous cyst.  Diagnosis Justification:  The association with an unerupted or impacted tooth, which is a hallmark of dentigerous cysts.  The characteristic epithelial lining and fibrous connective tissue capsule support the diagnosis of a dentigerous cyst, which is the most common type of odontogenic cyst. |
| 14.14 | **Diagnosis**: Periapical Scar; **Key Microscopic Features**: Dense fibrous scar tissue with nerve fibers of varying sizes and brown root canal filler. The suction artifact creates round-to-ovoid spaces filled with wispy amorphous material. | Based on the provided images of the oral and maxillofacial lesion stained with hematoxylin and eosin, the most likely diagnosis is Calcifying Odontogenic Cyst (COC), also known as Gorlin cyst.  Key Microscopic Features:  Cystic Structure: The lesions display cystic spaces with a lining of epithelial cells that appear somewhat flattened or cuboidal.  Ghost Cells: Characteristically, you can observe ghost cells (cells that appear empty or degenerated, lacking nuclei) within the cyst wall and in the surrounding stroma. These ghost cells are a hallmark feature of COC and play a key role in distinguishing it from other odontogenic cysts.  Calcification: There are areas of calcification within the cyst wall or stroma, which is another distinguishing feature. The calcified areas can vary from small foci to larger, more well-formed structures.  Odontogenic Epithelium: The epithelial lining is composed of a layer of odontogenic epithelium, typically resembling the enamel organ or ameloblasts, which further supports the diagnosis of an odontogenic lesion.  Fibrous Stroma: The surrounding stroma often consists of fibrovascular tissue, which may show varying degrees of cellularity.  These features together justify the diagnosis of Calcifying Odontogenic Cyst (COC). | Based on the provided hematoxylin-and-eosin (H&E) images, the diagnosis likely falls under the category of odontogenic keratocyst (OKC). This cyst is commonly found in the category of odontogenic cysts, which are lesions originating from the tissues involved in tooth development.  Key Microscopic Features:  Parakeratinized Epithelium:  The presence of a thin parakeratinized epithelial lining is characteristic, with some cells exhibiting retained nuclei in the keratin layer. This feature is typical of the odontogenic keratocyst.  Cyst Wall and Keratinization:  The cyst wall is composed of keratinized squamous epithelium. The layers of epithelium often exhibit a regular, stratified pattern with prominent basal layers. The keratinized layer is distinct and may appear thickened.  Thin Cyst Wall:  The cyst wall is usually thin, and there is often an absence of significant inflammation in the surrounding connective tissue, a feature commonly seen in OKCs.  Hyperchromatic Basal Layer:  The basal layer of the epithelium is typically hyperchromatic (darker staining) and shows a regular, palisaded arrangement of cells. These cells often appear columnar or cuboidal.  Flattened Epithelium:  The epithelium shows a flattened appearance in some regions, further confirming the odontogenic keratocyst diagnosis, as the epithelium typically lacks the complex differentiation found in other cysts.  Clear Fluid Space:  The cystic lumen is often filled with a clear fluid or keratinous material, which helps distinguish it from other types of cysts like dentigerous cysts, which might have different fluid characteristics.  These features justify the diagnosis of an odontogenic keratocyst, a lesion with distinctive epithelial features and a tendency to cause significant bone expansion, sometimes associated with syndromes like Gorlin-Goltz syndrome.  If you need more detailed analysis or further support regarding treatment or management, feel free to ask! |
| 14.18 | **Diagnosis**: Gingival Cyst of the Adult; **Key Microscopic Features**: The cyst is lined by uniformly thin epithelium, with no inflammation, and has a cuboidal-to-columnar lining varying from two to four cells thick. | Based on the provided hematoxylin and eosin (H&E) stained images of the odontogenic cysts, here is the analysis:  Single Most Likely Histopathological Diagnosis:  The lesions likely represent Dentigerous Cysts (also known as Follicular Cysts). These are the most common type of developmental odontogenic cyst and are typically associated with the crown of an unerupted or partially erupted tooth.  Key Microscopic Features Observed in the Images:  Lesion Structure:  Both images show cystic spaces lined by a thin layer of epithelial cells. This is characteristic of odontogenic cysts.  Thin Epithelium:  The cyst is lined by a non-keratinized stratified squamous epithelium, which is typically thin and one-to-three cell layers thick. This is often seen in dentigerous cysts.  Associated Tooth Structures:  In the first image, there is a portion of the cyst lining with a clear space, which could indicate a cystic lumen adjacent to the tooth crown. This suggests that the lesion might be surrounding the crown of an unerupted tooth, a hallmark feature of dentigerous cysts.  Subepithelial Fibrous Tissue:  Beneath the epithelium, there is dense fibrous connective tissue (pink-stained), which is typical of odontogenic cysts. This layer often shows no significant inflammation, supporting the diagnosis of a dentigerous cyst.  Absence of Inflammatory Cells:  There is a lack of significant inflammatory infiltrate in the subepithelial region, further suggesting that this cyst is not infected or inflamed. This is a typical feature of a non-inflammatory cyst like a dentigerous cyst.  These key microscopic features—thin epithelium, cystic cavity, and fibrous connective tissue—along with the typical association with an unerupted tooth, strongly suggest that the lesion is a dentigerous cyst. | The provided hematoxylin-and-eosin (H&E) images appear to show tissue sections indicative of an odontogenic cyst. Let's analyze the key histopathological features:  Diagnosis:  The lesion in these images most likely represents a dentigerous cyst (also known as follicular cyst), which is a common type of odontogenic cyst.  Key Microscopic Features Justifying the Diagnosis:  Epithelium:  The cystic lining is composed of a thin layer of stratified squamous epithelium, which is characteristic of odontogenic cysts.  In the images, there appears to be a clear, well-defined cystic lumen lined by flattened to cuboidal epithelium.  Cyst Lining:  The cyst wall appears to be non-inflammatory and lined by epithelium with areas of potential thickening, which is a typical feature of a dentigerous cyst.  Presence of an Impacted Tooth:  The most defining feature of a dentigerous cyst is its association with an unerupted or partially erupted tooth, which is often observed at the center of the cyst, and could be identified in the image. The first image shows a cystic structure surrounding a developing tooth, further confirming the diagnosis.  Fibrous Stroma:  Beneath the epithelial lining, there is a fibrous connective tissue capsule, which is typical for these cysts.  Lack of Inflammation:  The absence of significant inflammation or granulomatous changes in the surrounding tissue is another indication that this is likely a dentigerous cyst, as these cysts are typically non-inflammatory unless infected.  Summary:  This lesion most likely represents a dentigerous cyst, characterized by:  A thin epithelial lining  An impacted tooth enclosed within the cystic cavity  A fibrous stroma surrounding the cyst |
| 14.18b | **Diagnosis**: Gingival Cyst of the Adult; **Key Microscopic Features**: The cyst is uniformly thin and contains epithelial plaques with many clear cells, as well as scattered microcystic or rudimentary ductal structures. | The images you have provided depict a lesion from the group of odontogenic cysts in oral and maxillofacial pathology. Based on the histopathological features, the most likely diagnosis is:  Odontogenic Keratocyst (OKC)  Key Microscopic Features:  Image 1 (Low magnification):  Keratinized Cyst Lining: A characteristic feature is the presence of a thickened keratinized epithelium lining the cyst cavity, which appears in the middle of the image as a relatively smooth wall with a central area that might show cyst contents.  Palissading of Basal Cells: The basal layer of the epithelium is prominent, with cells arranged in a palisaded pattern, which is one of the hallmark features of an odontogenic keratocyst.  Surrounding Dense Connective Tissue: There is evidence of dense, fibrous connective tissue surrounding the cyst, often seen in OKC.  Image 2 (High magnification):  Thin and Stratified Epithelium: The cyst lining is composed of thin, stratified squamous epithelium.  Prominent Basal Layer: The basal cells are elongated and show palisading, and they might have a slightly hyperchromatic appearance.  Corrugated Surface: The epithelial surface is smooth but shows some signs of corrugation, which is consistent with keratinization.  Minimal Inflammatory Infiltrate: There is often minimal or no significant inflammation present within the cyst wall, which is another key feature of OKC.  The combination of these features supports the diagnosis of Odontogenic Keratocyst, which is known for its aggressive behavior and recurrence potential. | The images you provided are of a lesion that appears to be associated with the category of odontogenic cysts. Based on the histopathological features observed in the images, the most likely diagnosis is Dentigerous cyst (also known as follicular cyst).  Key Microscopic Features:  Presence of a cystic space: Both images reveal a well-defined cystic cavity surrounded by a thin layer of epithelial lining. The cyst space often contains minimal or no cellular debris, which is characteristic of the dentigerous cyst.  Epithelial lining: The cyst is lined by a thin layer of odontogenic epithelium, which is a hallmark of the dentigerous cyst. The epithelial lining often appears flattened or cuboidal, which is consistent with the dentigerous cyst's characteristic histology.  Associated with impacted tooth: The first image hints at a structure within the cyst that could correspond to an unerupted or impacted tooth, a feature often seen in dentigerous cysts, as they are frequently associated with the crown of an impacted tooth, typically the third molars.  Fibrous capsule: Both images also show surrounding connective tissue that forms a fibrous capsule, which is another feature of odontogenic cysts, especially dentigerous cysts.  Diagnosis:  Based on the histopathological findings, including the cystic appearance, epithelial lining, and association with a tooth structure, the diagnosis is most likely a Dentigerous Cyst. |
| 14.19 | Diagnosis: Gingival Cyst of the Adult; Key Microscopic Features: A cyst is present in the lamina propria, filled with parakeratin, without basal cell palisading. | Based on the hematoxylin and eosin (H&E) stained images provided, the lesion appears to be consistent with a dentigerous cyst (also known as a follicular cyst), which is an odontogenic cyst.  Key Microscopic Features Justifying the Diagnosis:  Cystic Structure with Collapsed Epithelium (Image 1):  The first image shows a well-defined cystic structure surrounding the crown of an unerupted tooth.  The cyst wall contains a thin layer of flattened epithelium, indicative of a dentigerous cyst.  The cyst is unilocular and filled with fluid, which is a characteristic finding.  Amorphous Keratinized Layer (Image 2):  In the second image, there is a clearer representation of the cyst wall with a layer of keratinized epithelial cells, a typical feature of dentigerous cysts.  The epithelial lining is often thin and may contain a layer of squamous epithelium with or without keratinization, as seen here.  Proximity to the Crown of an Impacted Tooth:  The lesion is surrounding the crown of an unerupted or impacted tooth, which is one of the hallmark features of dentigerous cysts. This feature helps to differentiate it from other odontogenic cysts.  These histological features, particularly the cystic cavity surrounding the crown of an unerupted tooth with thin, flattened epithelium, are diagnostic of a dentigerous cyst. | The provided histopathological images are of an odontogenic cyst, most likely a dentigerous cyst. Here's an analysis based on the key microscopic features that are seen:  Diagnosis:  Dentigerous Cyst (also known as a Follicular Cyst)  Key Microscopic Features:  Image 1 (Low Magnification):  The cyst is seen as a well-defined, unilocular space surrounding the crown of an unerupted or impacted tooth. This is a classic feature of a dentigerous cyst.  The cyst wall is composed of a thin layer of connective tissue.  The cyst lining is continuous with the reduced enamel epithelium, which is characteristic of dentigerous cysts.  Image 2 (High Magnification):  The cyst lining consists of a thin, non-keratinized stratified squamous epithelium.  The epithelium is usually 2-3 cell layers thick and shows no evidence of inflammation.  The connective tissue is fibrous, with minimal inflammatory infiltrate, which is typical of a dentigerous cyst.  There are no signs of neoplastic transformation, which further supports the diagnosis of a benign odontogenic cyst.  These features are consistent with a dentigerous cyst, characterized by its association with an unerupted tooth, thin epithelial lining, and minimal inflammation in the surrounding connective tissue. |
| 14.20 | Diagnosis: Implantation Cyst; Key Microscopic Features: A cyst appears at the site of surgery, deep within the lamina propria, lined by orthokeratinized stratified squamous epithelium. | The two images you provided show histopathological features consistent with an odontogenic cyst, specifically a dentigerous cyst (also known as follicular cyst).  Key microscopic features seen in the images that justify this diagnosis:  Histological Structure:  Cystic Lumen: The cystic cavity is lined by a thin, non-keratinized epithelium, typical of a dentigerous cyst. The epithelium is often one to two layers thick.  Associated Tooth Crown: One of the defining features of a dentigerous cyst is the presence of an unerupted or impacted tooth, often seen in the periphery of the cyst lining, which can be seen in the second image near the bottom center, where the cystic cavity is closely associated with a developing tooth.  Epithelium:  The cyst is lined by ameloblastoma-like epithelium, which appears as a low to cuboidal layer, with few areas showing basal cell hyperplasia or slight columnar cells at the base, indicative of the dentigerous cyst lining.  There is no significant cellular atypia or hyperplasia, confirming the diagnosis of a benign cyst.  Fibrous Capsule:  The cyst is surrounded by a fibrous capsule, which is typically dense and slightly inflamed, which can be appreciated in the images, especially in the areas surrounding the cystic cavity.  Summary:  Based on the provided images and the classic features observed, the diagnosis is most likely a dentigerous cyst. The key microscopic features—cystic lumen, thin epithelial lining, association with an unerupted tooth, and the fibrous capsule—are all characteristic of this odontogenic cyst type. | The images you provided depict a lesion from the group of odontogenic cysts in oral and maxillofacial pathology. The histopathological diagnosis for these images is most likely a Dentigerous Cyst.  Key Microscopic Features Supporting the Diagnosis:  Cyst Lining: The lesion shows a cystic structure lined by a thin, non-keratinized squamous epithelium, which is characteristic of a dentigerous cyst.  Surrounding Inflammation: There is some mild inflammatory infiltrate around the cyst, which is often seen in odontogenic cysts.  Associated with an Impacted Tooth: The cyst appears to be associated with the crown of an unerupted or impacted tooth (evident from the image showing the tooth-like structure at the bottom).  Wall Thickness: The cyst wall is somewhat thickened with a fibrous capsule, which is a common feature of dentigerous cysts.  These features are classic for a dentigerous cyst, often seen in association with the crown of an unerupted tooth, and the histological examination confirms this diagnosis. |
| 14.21 | Diagnosis: Eruption Cyst; Key Microscopic Features: The cyst is located within the deep lamina propria, with a uniformly thin epithelium and mild chronic inflammation, resembling an inflamed dentigerous cyst. | Based on the provided hematoxylin-and-eosin (H&E) images of an odontogenic cyst, let's conduct a thorough analysis:  Histopathological Diagnosis:  The most likely histopathological diagnosis for these images is Keratocystic Odontogenic Tumor (KCOT), also referred to as Odontogenic Keratocyst (OKC).  Key Microscopic Features Justifying the Diagnosis:  Parakeratinized Epithelium:  Both images show epithelium with a characteristic thin, parakeratinized layer, which is a typical feature of OKC. The presence of parakeratinized cells in the epithelial lining is one of the main diagnostic criteria.  Thin Epithelium with Multiple Layers:  The epithelium is relatively thin, and there is a uniform, multiple-layered appearance that is characteristic of the cystic wall of OKC. It lacks the epithelial layers seen in other types of cysts.  Cystic Lumen Containing Fluid or Keratin Material:  The cystic lumen shown in the images likely contains keratin debris, which is common in OKC. The images show areas of necrosis and keratinization within the lumen, further supporting this diagnosis.  Thin Basal Layer:  The basal layer of the epithelium is notably palisaded, with cells that are columnar to cuboidal in shape. The epithelium of OKC tends to show a palisaded basal layer, which is also seen in the images.  Infiltration of the Surrounding Stroma:  There appears to be a dense connective tissue stroma surrounding the cyst, and the stroma shows a minimal inflammatory infiltrate, typical of OKC. This minimal inflammation is a key diagnostic feature.  Potential for Aggressive Growth:  The architectural features suggest that this cyst may demonstrate locally aggressive behavior, a hallmark of KCOTs, as they often tend to invade surrounding bone or soft tissue.  Conclusion:  The combination of parakeratinized epithelium, thin epithelial layers, and the presence of keratinization material within the cystic lumen are characteristic of the Keratinized Odontogenic Cyst (OKC). These features, along with the minimal inflammation and dense surrounding stroma, point toward the diagnosis of Kerotocystic Odontogenic Tumor (KCOT). | The images you've provided appear to be histopathological slides of an odontogenic cyst. Based on the visual features seen in these high-resolution images and the provided magnifications, the single most likely diagnosis for this lesion is an odontogenic keratocyst (OKC).  Here are the key microscopic features that justify this diagnosis:  Layered Epithelium: In both images, the cyst lining is composed of keratinized stratified squamous epithelium, which is a characteristic feature of the odontogenic keratocyst. The epithelial layer is often described as being thin and uniformly thick, with a tendency to show parakeratinization.  Basal Layer Hyperplasia: There is hyperplasia of the basal layer of the epithelium. The basal layer appears to have a more prominent, palisaded appearance, and the cells are densely packed with high mitotic activity.  Keratinization: The cystic lining displays areas of keratinization, a hallmark of the odontogenic keratocyst. This keratinization appears to be parakeratin, as seen in the first image, and may account for the more uniform and sometimes thin nature of the epithelial lining.  Thin Cyst Wall: The wall of the cyst appears to be thin and lacks significant connective tissue involvement, another distinguishing feature of OKCs.  No Significant Inflammation: The presence of little to no inflammation within the cyst wall is another clue supporting an odontogenic keratocyst, as these lesions often show minimal inflammatory response.  The odontogenic keratocyst, classified under the group of odontogenic cysts, has distinctive histopathological features, as shown in the images. The layered, keratinized epithelium with basal cell hyperplasia and the characteristic thin wall with minimal inflammation further supports this diagnosis. |
| 14.23 | Diagnosis: Dentigerous Cyst; Key Microscopic Features: A cyst lined by a uniformly thin epithelium with minimal mural inflammation, with a nonkeratinized stratified squamous epithelium 5 to 10 cells thick. | Based on the images provided, it appears that the lesion in question is most consistent with an odontogenic keratocyst (OKC), a common lesion within the group of odontogenic cysts in oral and maxillofacial pathology.  Histopathological Diagnosis:  Odontogenic Keratocyst (OKC)  Key Microscopic Features Seen in the Images:  First Image (Low Magnification):  The lesion shows a cystic cavity lined by a layer of epithelium.  There is an apparent parakeratosis on the epithelial surface (a layer of keratinized cells), which is a hallmark feature of an OKC.  The surrounding tissue shows some fibrosis and inflammation, which is typical in cysts as they are surrounded by a fibrous capsule.  Second Image (Higher Magnification):  The cyst lining consists of stratified squamous epithelium with a uniform thickness.  The basal cell layer is prominent, showing a palisading arrangement of cells (elongated, vertically aligned cells).  There is parakeratosis on the surface (presence of keratinized cells with retained nuclei).  Increased mitotic activity may be visible in the basal layer, which supports the aggressive nature of the cyst.  Justification for the Diagnosis:  The characteristic features of palisading basal cells with parakeratosis, as well as the thin, uniform epithelium lining the cyst cavity, are typical of an odontogenic keratocyst. This cyst is known for its potential for aggressive growth and recurrence after surgical removal, largely due to the proliferation of the basal cells. | The images you've uploaded depict histological sections stained with hematoxylin and eosin, which are commonly used to diagnose various oral and maxillofacial lesions. Based on these images, I'll provide an analysis and diagnosis.  Most Likely Histopathological Diagnosis:  The lesions shown are most consistent with an odontogenic keratocyst (OKC), a benign but aggressive developmental cyst originating from the remnants of the dental lamina or enamel organ.  Key Microscopic Features Justifying the Diagnosis:  Cystic Structure:  Both images reveal a well-defined cystic cavity surrounded by a layer of epithelial cells, characteristic of a cyst.  Keratinized Epithelium:  In both images, the cyst is lined by keratinized stratified squamous epithelium. This is a key feature of OKC, as the epithelium is typically 6-10 layers thick and demonstrates a parakeratinized or orthokeratinized surface.  Uniform Epithelium:  The epithelial lining exhibits uniform thickness and flat basal cells, suggesting an absence of significant reactive changes or inflammation.  Parakeratinization:  The surface of the epithelium is parakeratinized (nuclei retained in the superficial layers), another hallmark of OKC.  Thin, Wavy Epithelium:  The epithelium is relatively thin and often wavy, a characteristic feature of OKC, distinguishing it from other odontogenic cysts, which usually show thicker, more uniform epithelium.  Acellular Eosinophilic Zone (Keratinized Layer):  The cystic cavity often contains an eosinophilic keratinized material (indicating keratinization), which is a feature of OKC and helps confirm the diagnosis.  Lack of Inflammatory Infiltrate:  There is minimal to no inflammatory infiltrate seen in the surrounding stroma, which contrasts with other cysts that may show an inflammatory response.  Conclusion:  Based on the microscopic features observed in the images, the lesion is most consistent with an odontogenic keratocyst (OKC). This diagnosis is supported by the characteristic cystic structure, keratinized epithelial lining, and lack of significant inflammatory changes. OKCs are often aggressive and require careful management due to their tendency for recurrence. |
| 14.24 | Diagnosis: Dentigerous Cyst; Key Microscopic Features: The cyst contains epithelial plaques within its lining. | The images you provided appear to represent a lesion from the odontogenic cysts category in oral and maxillofacial pathology. Here's the analysis based on the hematoxylin-and-eosin-stained slides:  Most Likely Histopathological Diagnosis:  The diagnosis is most likely an odontogenic keratocyst (OKC), also known as a keratocystic odontogenic tumor (KCOT).  Key Microscopic Features:  Cystic Structure:  The first image shows a large cystic cavity with a thick, smooth lining, which is characteristic of an odontogenic keratocyst. This cystic cavity often has a parakeratinized epithelium, which may also be observed in these images.  Epithelium Characteristics:  In the second image, the cystic lining is made up of stratified squamous epithelium, which shows a corrugated surface (wavy appearance) typical of OKCs. The epithelium may appear 6-10 cell layers thick with a distinct basal layer that has palisading cells, a hallmark of OKC.  The parakeratin layer is prominent in the epithelium, which is characteristic of OKCs as they often present with a thin or absent keratinized layer on the surface.  Mitoses and Cell Proliferation:  There may be hyperplasia in the basal epithelial layer, which often demonstrates an increased mitotic activity. This feature, though not very prominent in the provided images, is commonly found in OKCs.  Lack of Inflammation:  Unlike other cysts, such as the dentigerous cyst, the odontogenic keratocyst typically exhibits minimal inflammation or surrounding reactive tissue, which is consistent with the image you provided.  Wall of the Cyst:  The cyst wall in the first image appears thin, with a smooth, non-inflammatory interface, which is common for OKCs.  These findings collectively point towards an odontogenic keratocyst (OKC) as the most likely diagnosis. | The provided images show a lesion with distinct features typical of an odontogenic cyst, a group of cystic lesions originating from the tissues involved in tooth development.  Most Likely Histopathological Diagnosis:  Dentigerous cyst (also known as follicular cyst)  Key Microscopic Features Justifying the Diagnosis:  First Image (Low Magnification):  Well-defined cystic structure: The image shows a large, well-demarcated cyst cavity. There is a clear cyst lining, characteristic of a cystic lesion.  Presence of an epithelial lining: The cyst is lined by a layer of odontogenic epithelium, which is a key feature of odontogenic cysts.  Associated with a tooth crown: The lesion seems to be surrounding the crown of a developing tooth, which is consistent with a dentigerous cyst. This cyst often surrounds the crown of an unerupted or impacted tooth, especially the third molars.  Second Image (Higher Magnification):  Columnar to cuboidal epithelial cells: The epithelial lining of the cyst consists of a single layer of cuboidal or columnar cells, which is a typical feature of dentigerous cysts.  Minimal to no inflammation in the cyst wall: The cyst wall in dentigerous cysts usually contains few inflammatory cells, and this is reflected in the image.  Amorphous eosinophilic material: The presence of eosinophilic material or some amorphous, proteinaceous material can be observed in the cystic space, which may represent secretions or cellular debris typical of odontogenic cysts.  These features strongly suggest a dentigerous cyst as the most likely diagnosis. The association with a developing tooth crown and the characteristic epithelial lining are central to identifying this lesion. |
| 14.24b | Diagnosis: Dentigerous Cyst; Key Microscopic Features: The cyst is lined by a uniform 5 to 8 cell thick epithelium, with columnar, mucous, and ciliated cells. | Based on the images provided, I will analyze the histopathological features to offer a diagnosis.  Diagnosis:  The lesion shown in the images is most likely an odontogenic keratocyst (OKC).  Key Microscopic Features:  Cyst Lining:  The first image shows a thick, parakeratinized epithelial lining with a uniform appearance. The epithelial layer appears as a relatively thin, multi-layered structure, with the basal layer being palisaded. This is characteristic of an odontogenic keratocyst.  Proliferation of the Epithelium:  The second image demonstrates the presence of a well-defined basal layer of cuboidal or columnar cells that show a palisaded arrangement, which is often seen in OKC. There is also evidence of hyperchromatic nuclei at the basal layer, which is another distinguishing feature of OKC.  Keratinization:  The presence of parakeratinized epithelium at the luminal surface (the first image) further supports the diagnosis of OKC. This is typical of OKC, where the cyst lining often demonstrates partial keratinization, with areas of parakeratosis visible.  Subepithelial Layer:  The stroma beneath the epithelium appears to be relatively dense, with minimal inflammation. This is a characteristic feature of the odontogenic keratocyst, where the underlying connective tissue is typically not inflamed, and no significant fibrosis is seen.  No Significant Inflammation:  The images show minimal or no inflammatory cells within the cystic lining or stroma, which is another feature that points towards OKC, as these cysts tend to have very little inflammation.  These histopathological features, including the well-defined basal cell layer, parakeratinization, minimal inflammation, and the characteristic appearance of the lining epithelium, support the diagnosis of odontogenic keratocyst. | Based on the provided hematoxylin-and-eosin (H&E) stained images, which depict sections of a lesion within the category of odontogenic cysts, the most likely histopathological diagnosis is Dentigerous Cyst.  Key Microscopic Features:  First Image (Low Magnification):  Lesion Type: A cystic structure is visible, showing an epithelial lining surrounding a cystic lumen.  Epithelial Lining: The lining of the cyst appears to be of the odontogenic epithelium type, which is consistent with the dentigerous cyst.  Surrounding Tissues: The cyst is closely associated with an impacted tooth, a key feature of the dentigerous cyst.  Second Image (High Magnification):  Epithelial Lining Detail: The cyst is lined by a layer of non-keratinized, cuboidal to columnar epithelium. The presence of a thin epithelial lining is characteristic of dentigerous cysts.  Hyperchromatic Nuclei: The nuclei of the epithelial cells are slightly hyperchromatic and are arranged in a somewhat flattened pattern along the cyst wall, which is typical in odontogenic cysts.  Fibrous Stroma: Below the epithelial lining, the connective tissue appears to be loose, with few inflammatory cells, which is consistent with the fibrous stroma typically seen in dentigerous cysts.  Inclusion of Tooth Crown: The cyst is surrounding the crown of an unerupted or impacted tooth, which is an essential feature for diagnosing a dentigerous cyst.  Conclusion:  The lesion depicted in the images is most consistent with a Dentigerous Cyst, primarily due to its association with an impacted tooth, a cystic cavity with thin epithelial lining, and a lack of significant inflammatory infiltrate in the stroma. These are all typical findings in this type of odontogenic cyst. |
| 14.25 | Diagnosis: Dentigerous Cyst; Key Microscopic Features: Cholesterol granulomas are associated with damage to the lining and hemorrhage, consisting of needle-shaped clefts with multinucleated giant cells and macrophages. | The images provided are representative of a lesion within the group of odontogenic cysts, specifically pointing to an Adenomatoid Odontogenic Tumor (AOT). This diagnosis is suggested by the following histopathological features:  Diagnosis: Adenomatoid Odontogenic Tumor (AOT)  Key Microscopic Features:  Columnar and Cuboidal Cells:  The first image shows a cystic structure with clusters of epithelial cells arranged in a whirlpool pattern. These cells are typically cuboidal to columnar and might resemble glandular structures, which is characteristic of AOT.  Presence of Dystrophic Calcifications:  In the first image, needle-like or elongated calcifications can be observed. These calcifications are often seen in AOT and give the lesion a distinctive appearance, as they can appear as "whorls" or "spindles" within the cystic lining.  Fibrous Capsule:  Both images show the presence of a fibrous capsule around the cyst, which is a common feature of AOT, indicating the lesion is encapsulated.  Well-defined Epithelial Components:  In the second image, you can notice the well-defined odontogenic epithelium surrounding areas of the cyst. The cyst wall typically shows proliferation of the epithelial lining, which may present a sheet-like or papillary arrangement, common in AOT.  Conclusion:  The combination of these microscopic features—whorled epithelial patterns, calcifications, and a fibrous capsule—supports the diagnosis of Adenomatoid Odontogenic Tumor. | The images you provided depict a lesion that is likely associated with an odontogenic cyst. Here is an analysis based on the histopathological features:  Single Most Likely Diagnosis:  Ameloblastoma (specifically the unicystic type)  Key Microscopic Features:  First Image (Lower Magnification):  The lesion shows a cystic structure with epithelial lining.  Radiating arrangement of odontogenic epithelial cells is seen, which can suggest ameloblastomatous features.  The cystic cavity is surrounded by a fibrous capsule, with some areas showing dentigerous-like changes.  This radiating arrangement is a hallmark of the unicystic ameloblastoma variant, which often appears in younger patients and typically surrounds an unerupted tooth.  Second Image (Higher Magnification):  The epithelial lining of the cyst is more clearly seen to consist of columnar or cuboidal cells, with some areas resembling ameloblast-like cells with prominent nuclei.  There is also evidence of reverse polarity, where the nuclei of the ameloblast-like cells are located at the opposite side of the basement membrane.  The underlying connective tissue shows vacuolated (or clear) stromal cells, which is characteristic of this cystic variant.  There is hyperplasia of the basal layer of the epithelium, showing a resemblance to the ameloblasts, reinforcing the diagnosis of an ameloblastoma.  Conclusion:  The key features of the unicystic ameloblastoma seen in these images include:  A cystic structure lined by ameloblast-like cells,  The characteristic reverse polarity and hyperplasia of the basal epithelial layer,  The radiating arrangement of the odontogenic epithelium,  and a fibrous capsule.  This diagnosis is further supported by the presence of a fibrous capsule and the specific epithelial features that distinguish it from other cystic lesions in the odontogenic cyst group. |
| 14.30 | Diagnosis: Mucoepidermoid Carcinoma Arising within a Dentigerous Cyst; Key Microscopic Features: Infiltrative tumor cell islands are found adjacent to the cyst lining, with some portions showing mucous cell prosoplasia. The tumor consists of epidermoid, mucous, and intermediate cells forming ducts. | Based on the provided hematoxylin-and-eosin (H&E) images, here is my analysis:  Single Most Likely Histopathological Diagnosis:  Odontogenic Keratocyst (OKC)  Key Microscopic Features That Justify the Diagnosis:  Cystic Structure:  The images show a well-formed cystic structure with a lining that is characteristic of an odontogenic cyst. The cyst is lined by a thin, keratinized epithelium.  Thin Stratified Squamous Epithelium:  The epithelium is thin and shows a stratified squamous type, with some regions exhibiting orthokeratinization (no nuclei in the outermost layer of the epithelium), a feature commonly seen in OKC.  Basal Cell Layer:  The basal layer of the epithelium demonstrates a characteristic appearance of cuboidal to columnar cells with palisading. This basal layer is one of the key diagnostic features for odontogenic keratocyst.  Prominent Satellite Cysts:  Small cystic formations can be seen in the surrounding tissue, which are often noted in the periphery of the primary cyst. These satellite cysts are also a characteristic finding in odontogenic keratocysts.  Orthokeratinization and Lack of Inflammation:  The absence of significant inflammation and the presence of orthokeratinization is typical for OKC, as it does not usually show the inflammatory response seen in other cystic lesions like dentigerous cysts.  Uniform Epithelium:  The lining appears uniform and smooth without marked variation in thickness, a feature often present in OKC.  This combination of cystic formation, thin epithelial lining, keratinized layers, and absence of significant inflammation supports the diagnosis of odontogenic keratocyst. | Based on the hematoxylin and eosin (H&E) images provided, I will perform an analysis and offer a histopathological diagnosis, including key features that support the diagnosis of the odontogenic cyst.  Diagnosis:  The most likely diagnosis based on these H&E images is Dentigerous Cyst.  Key Microscopic Features Supporting the Diagnosis:  Cystic Structure: The images show a well-formed cystic lesion with a fibrous capsule and a lumen filled with clear fluid. This is a hallmark of a dentigerous cyst.  Relationship to an Impacted Tooth: In one of the images, you can observe structures associated with the crown of an unerupted or impacted tooth, which is commonly seen in dentigerous cysts. This is a key feature as dentigerous cysts often surround the crown of an impacted tooth.  Thin Epithelial Lining: The cystic lumen is lined by a thin layer of non-keratinized squamous epithelium, a characteristic feature of a dentigerous cyst.  Presence of a Thick Fibrous Capsule: The surrounding fibrous capsule is evident in the images, with some areas showing chronic inflammatory cells. The capsule is thicker in some areas, which can be suggestive of the chronic nature of the cyst.  No Inflammation or Infection: While inflammation might be present, it is not overwhelming or acute. The cyst's wall appears relatively stable, which helps differentiate it from more aggressive lesions.  These histopathological features—along with the cystic nature, epithelial lining, association with a tooth, and a fibrous capsule—are consistent with a dentigerous cyst. |
| 14.33 | Diagnosis: Lateral Periodontal Cyst; Key Microscopic Features: The cyst has a thin lining, thick basement membrane, and a fibrous wall without inflammation, lined by nonkeratinized stratified squamous epithelium 2 to 3 cells thick. | Based on the images you provided, these appear to be hematoxylin-and-eosin-stained sections of an odontogenic cyst. Here's an analysis:  Histopathological Diagnosis:  Dentigerous Cyst  Key Microscopic Features:  Cyst Lining: The lesion is lined by a thin layer of epithelial cells, which is characteristic of a dentigerous cyst. This epithelium is often cuboidal or columnar.  Presence of a Surrounding Collagenous Stroma: The cyst is surrounded by a dense collagenous stroma, a key feature of odontogenic cysts.  Fluid-filled Lumen: The lumen of the cyst is filled with a clear, often watery fluid. In the images, there is a clear space indicative of this lumen.  Pericystic Inflammation: The cystic lining and the surrounding area show some inflammatory cells, particularly lymphocytes and plasma cells, although this inflammation is not as extensive as in some other cystic lesions.  Association with Tooth: In odontogenic cysts, particularly dentigerous cysts, the cyst is often associated with the crown of an unerupted or impacted tooth. The images suggest a possible relationship with tooth structures, given the proximity of the cyst lining to areas of the tooth root.  These features suggest that the lesion is most likely a dentigerous cyst, a common type of odontogenic cyst. It is typically associated with an unerupted or impacted tooth, often involving the third molar. | The images you've provided represent a histopathological examination of an odontogenic cyst. Here is the analysis:  Most Likely Histopathological Diagnosis:  Dentigerous cyst (also known as follicular cyst)  Key Microscopic Features:  Cystic Structure: Both images show a cystic lesion lined by a thin epithelial layer, indicative of a dentigerous cyst. The lesion appears well-defined and surrounded by a thin layer of epithelial cells.  Epithelial Lining: The cyst is lined by a non-keratinized stratified squamous epithelium. The epithelium is thin and may show slight hyperplasia in certain areas. This is characteristic of a dentigerous cyst.  Presence of Fluid-Filled Lumen: The lumen of the cyst appears wide, filled with clear fluid, and lacks cellular debris, which is typical for a dentigerous cyst.  Associated with Impacted Tooth: The images suggest the presence of a cyst that is closely associated with an impacted tooth crown, a hallmark feature of the dentigerous cyst.  Fibrous Capsule: The cyst is surrounded by a fibrous capsule, which is usually of varying thickness and shows mild inflammatory infiltrates.  The microscopic features seen in these images are consistent with a dentigerous cyst, primarily due to the cyst's appearance, epithelial lining, and association with a tooth crown. |
| 14.34 | Diagnosis: Lateral Periodontal Cyst; Key Microscopic Features: The cyst lining is uniformly thin, with epithelial plaques containing clear cells, and an odontogenic rest composed of clear cells present in the wall without inflammation. | The images you've provided appear to show histopathological sections of an odontogenic cyst, with H&E staining. After analyzing both images at different magnifications, the likely diagnosis is Dentigerous Cyst.  Key Microscopic Features:  Epithelial Lining: The cysts exhibit a non-keratinized stratified squamous epithelium that is usually thin. This is a key feature of odontogenic cysts, including dentigerous cysts.  Subepithelial Connective Tissue: Below the epithelial lining, the subepithelial connective tissue appears loose and fibrocellular. This is characteristic of the cyst's wall.  Enlargement of the Follicular Area: In dentigerous cysts, the cystic space is usually associated with the crown of an unerupted or partially erupted tooth. This enlargement is noticeable in the first image as well.  Cystic Space: The lumen of the cyst in the images is filled with clear fluid, often an indication of the cystic nature of the lesion. This feature is consistent with the dentigerous cyst's typical structure.  No Inflammatory Cells: In the given histology, there is a lack of significant inflammatory infiltration, which is often a feature of dentigerous cysts (they tend to be quite benign with minimal inflammation unless infected).  These findings strongly point to a Dentigerous Cyst, a common odontogenic cyst often found around the crown of an unerupted or impacted tooth. | Based on the provided hematoxylin-and-eosin (H&E) images, we can assess the features of the lesion and come to a histopathological diagnosis. The images represent different magnifications of the same lesion in the "Odontogenic Cysts" category.  Histopathological Diagnosis:  The lesion most likely corresponds to an Odontogenic Keratocyst (OKC).  Key Microscopic Features:  Cyst Lining:  In both images, the cyst is lined by a thin, stratified squamous epithelium. In the higher magnification image, we see the hyperchromatic basal layer with columnar to cuboidal basal cells, a hallmark of OKC.  The epithelium appears to have a parakeratinized surface layer in certain areas, indicative of OKC.  Keratinized Surface:  There is keratinization on the surface epithelium, which is characteristic of the OKC, often with some areas exhibiting parakeratin (nuclei retained in the outermost layer).  Cystic Lumen:  The cystic cavity is filled with keratin debris, which is common in OKCs due to the keratinized epithelium.  Epithelium Thickness:  The cyst wall demonstrates a uniform thickness of the epithelium, which is one of the distinguishing features of OKCs, in contrast to other cysts that may show varying epithelium thickness.  Peripheral Inflammation:  The surrounding connective tissue capsule is typically fibrous with minimal or no inflammation, further supporting the diagnosis of an odontogenic keratocyst, which is known for its relatively low inflammatory response.  Conclusion:  The odontogenic keratocyst (OKC) diagnosis is supported by the characteristic features of a thin, parakeratinized, hyperplastic epithelium lining a cystic cavity with keratin debris. The uniform epithelium thickness and the presence of basal cell hyperplasia are also strong indicators of OKC. |
| **figure_no** | **paraphrased** | The images you provided show a lesion from the group of odontogenic cysts, likely representing an odontogenic keratocyst (OKC). Here's a breakdown based on the histopathological features observed in the images:  Histopathological Diagnosis:  Odontogenic Keratocyst (OKC)  Key Microscopic Features:  Parakeratinized Epithelium:  The epithelium lining the cyst is parakeratinized (indicated by the presence of keratinized cells with pyknotic nuclei in the upper layers) in the upper image.  Parakeratinization is a characteristic feature of the OKC epithelium.  Uniform, Thin Epithelium:  The cyst lining consists of thin, uniform epithelium, typically one to multiple layers thick. This is shown in both images, especially in the second image where the epithelium appears relatively thin with a well-defined basal layer.  Basal Layer with Hyperplasia:  In the images, the basal layer of the epithelium shows signs of hyperplasia (marked by a palisaded appearance and increased mitotic activity), which is another diagnostic feature of OKC.  Cystic Lumen with Fluid:  The lumen of the cyst appears clear and filled with a fluid-like substance, although this is not directly visualized in these images, it's generally a key feature for OKC in histology.  Lack of Inflammation:  These images show a minimal inflammatory response around the cyst, which is typical for OKC, differentiating it from other odontogenic cysts that may have a more prominent inflammatory infiltrate.  These histopathological findings support the diagnosis of an odontogenic keratocyst, which is often associated with aggressive behavior, recurrence, and its link to genetic syndromes such as Gorlin-Goltz syndrome. | The provided images show histopathological slides of a lesion from the odontogenic cysts group, specifically captured at different magnifications. Here's an analysis of these images:  Single Most Likely Diagnosis: Keratocystic Odontogenic Tumor (KCOT)  This is a common odontogenic cyst with distinctive features that are well represented in the provided slides.  Key Microscopic Features:  Stratified Squamous Epithelium: The cystic lining is composed of a thin, stratified squamous epithelium that may exhibit a parakeratinized surface layer. This is a hallmark of KCOT.  Basal Layer Hyperplasia: The epithelium shows a prominent basal layer with palisading of cells, which is one of the distinguishing features of KCOT.  Thin Connective Tissue Wall: The cyst is surrounded by a thin, fibrous connective tissue wall with minimal inflammatory infiltrate, which differentiates it from other cystic lesions that may show more pronounced inflammation.  Keratinization: The presence of keratinized layers, particularly in the lumen, is observed. This is a feature that sets KCOT apart from other odontogenic cysts, like dentigerous cysts, which lack significant keratinization.  Cystic Lumen: The lumen of the cyst may contain keratin and cellular debris, further supporting the diagnosis.  In summary, the distinct stratified squamous epithelium, hyperplastic basal cell layer, and keratinization are consistent with a diagnosis of Keratocystic Odontogenic Tumor (KCOT). |
| 1.69 | Diagnosis: Epidermoid Cyst; Key Microscopic Features: The low-power view displays a cystic cavity filled with keratin, while the high-power view shows stratified squamous epithelial lining with orthokeratin production. | Based on the provided images showing a lesion with hematoxylin and eosin staining at different magnifications, I would suggest the following histopathological analysis:  Single Most Histopathological Diagnosis:  Dentigerous Cyst (also known as Follicular Cyst)  Key Microscopic Features:  Low Magnification (First Image):  The cyst is well-circumscribed, with a thin, fibrous capsule surrounding the lesion.  The cystic lumen is lined by non-keratinized stratified squamous epithelium.  The cyst is attached to the crown of an impacted or unerupted tooth, which is typical for a dentigerous cyst.  High Magnification (Second Image):  The lining of the cyst is composed of a single layer of cuboidal to columnar epithelium with a smooth surface.  The epithelial lining may show occasional areas of hyperplasia, but it generally remains non-keratinized.  There may be clear spaces between the epithelial cells and the underlying connective tissue, which suggests the presence of cyst fluid.  The connective tissue wall beneath the epithelium is typically sparse, often with a mild inflammatory infiltrate.  The presence of these features, especially the attachment to the crown of an unerupted tooth and the cyst's epithelial lining, strongly suggests a dentigerous cyst in this case. | Based on the hematoxylin-and-eosin (H&E) stained images, the lesion appears to be consistent with an odontogenic cyst, more specifically the dentigerous cyst.  Histopathological Diagnosis:  Dentigerous cyst (also known as follicular cyst).  Key Microscopic Features:  Low Magnification (First Image):  The cystic structure has a well-defined fibrous capsule surrounding a central lumen, which contains clear fluid.  The cyst is associated with the crown of an unerupted or partially erupted tooth, a key feature for diagnosing a dentigerous cyst.  The epithelium lining the cyst is thin, typically a non-keratinized, stratified squamous epithelium.  The cystic lining is often uniform and appears to be directly related to the developing tooth crown.  High Magnification (Second Image):  The epithelial lining shows a thin layer of non-keratinized squamous epithelium.  There may be areas with a slight increase in cellularity or occasional hyperplasia of the basal layer.  The cyst lining is typically composed of a simple cuboidal or flattened epithelium, and in some cases, there could be areas of mild inflammation or cholesterol clefts in the cystic lumen.  The surrounding fibrous capsule shows varying degrees of fibroblast proliferation, consistent with the fibrous connective tissue of the cyst wall.  These microscopic features, particularly the relationship to the unerupted tooth, the thin epithelium lining, and the fibrous capsule, confirm the diagnosis of a dentigerous cyst. |
| 1.80 | Diagnosis: Oral Lymphoepithelial Cyst; Key Microscopic Features: The low-power view reveals a keratin-filled cyst beneath the mucosal layer. | Based on the provided hematoxylin and eosin (H&E) stained histological images of an odontogenic cyst, viewed at different magnifications, the single most likely histopathological diagnosis is:  Odontogenic Keratocyst (OKC)  Key Microscopic Features Justifying the Diagnosis:  Image 1 (Low magnification):  Cystic architecture: A large unilocular cystic cavity lined by a thin epithelial lining.  Parakeratinized lining: The epithelial lining is corrugated, suggesting parakeratosis, which is a hallmark of OKC.  Palisaded basal layer: The basal epithelial cells appear aligned in a palisaded (fence-like) pattern with hyperchromatic nuclei.  Flat epithelial-connective tissue interface: There is no rete ridge formation, which is typical of OKC.  Image 2 (High magnification):  Corrugated parakeratin surface: The epithelial surface shows a characteristic wavy or corrugated parakeratin layer, typical of OKC.  Palisaded basal cells: The basal layer exhibits tall, columnar, hyperchromatic nuclei arranged in a palisaded pattern.  Uniform epithelial thickness: The epithelium is 6–10 cell layers thick, another diagnostic clue.  Summary of Diagnostic Criteria for Odontogenic Keratocyst (OKC):  Thin, uniform parakeratinized stratified squamous epithelium  Corrugated surface of the keratin layer  Prominent, palisaded, hyperchromatic basal cell layer  Flat epithelial-connective tissue interface  Minimal inflammation unless secondarily infected  These microscopic features clearly support the diagnosis of Odontogenic Keratocyst (OKC), a developmental cyst known for its aggressive behavior and high recurrence rate. | Histopathological diagnosis (single best):  Odontogenic keratocyst (OKC) — also termed “keratocystic odontogenic tumour” in some classifications.  Microscopic features in the provided H-E sections that point to OKC  Feature How it appears in the images Diagnostic significance  Very thin epithelial lining (≈ 6–8 cell layers) At low power the cyst wall is lined by a uniformly slender purple band A thin, uniform lining is characteristic of OKC and helps distinguish it from other odontogenic cysts that have thicker, non-uniform epithelium  Corrugated/wavy parakeratin surface High-power image shows an intensely eosinophilic, rippled layer with retained pyknotic nuclei Parakeratinisation with a corrugated surface is a hallmark of OKC; other cysts tend to be orthokeratinised or non-keratinised  Palisaded, hyperchromatic basal cell layer Basal cells form a “picket-fence” or “row-of-tombstones” pattern with nuclei aligned perpendicular to the basement membrane This orderly palisading of basal cells is typical for OKC and uncommon in most other odontogenic cysts  Flat epithelial–connective-tissue interface (no rete ridges) The basement membrane appears sharp and planar, with little interdigitation OKCs usually have a smooth interface; lack of rete ridges contrasts with inflammatory odontogenic cysts that show elongated rete processes  Lamina of keratin debris in the lumen At low power, the cyst cavity contains swirls and sheets of pale eosinophilic keratin material Intravascular keratin flakes or cheesy keratin content is classic for OKC  Relatively fibrous, non-inflamed cyst wall ± satellite epithelial nests The supporting wall is collagenous pink with sparse inflammatory cells; small epithelial islands may be present The paucity of inflammation preserves the delicate lining; daughter (satellite) cysts, when seen, account for the lesion’s tendency to recur  Taken together, the combination of a thin uniform parakeratinised lining with corrugations, a palisaded basal layer, flat interface, and luminal keratin debris is diagnostic of an odontogenic keratocyst. |
| 14.35 | Diagnosis: Botryoid Odontogenic Cyst; Key Microscopic Features: The cyst contains multiple structures lined with uniformly thin epithelium and epithelial plaques, while the wall shows odontogenic rests with clear cells. | Based on the provided hematoxylin and eosin (H&E) stained histopathology images, here is the analysis:  Single Most Likely Histopathological Diagnosis:  Odontogenic Keratocyst (OKC)  (Also referred to as Keratocystic Odontogenic Tumor in older classifications)  Key Microscopic Features Supporting This Diagnosis:  1. Cystic Architecture (Low Magnification Image):  The lesion is well-circumscribed and unilocular, lined by a thin epithelial layer.  The lumen appears collapsed, often seen in OKC due to the friable nature of the epithelial lining.  Corrugated surface of the cystic lining can be appreciated even at this magnification.  2. Lining Epithelium (High Magnification Image):  Uniformly thin epithelial lining (~6–8 cells thick).  Corrugated parakeratinized surface (wavy, eosinophilic keratin layer), a hallmark of OKC.  Palisaded basal cell layer: The basal cells are columnar or cuboidal with hyperchromatic nuclei aligned in a "picket fence" pattern.  The flat epithelial-connective tissue interface (i.e., no rete ridges), typical of OKC.  The fibrous connective tissue capsule is relatively uninflamed and thin, which helps differentiate it from inflammatory odontogenic cysts.  Summary:  The constellation of a thin, uniform epithelial lining, corrugated parakeratinized surface, palisading basal layer, and a flat epithelial-connective tissue interface strongly points to Odontogenic Keratocyst (OKC). This diagnosis is critical due to OKC's known aggressive behavior and high recurrence rate, warranting careful surgical and follow-up management.  Let me know if you'd like a schematic summary or help with differential diagnoses. | Histopathological diagnosis  Parakeratinised odontogenic keratocyst (OKC / keratocystic odontogenic tumour).  Key microscopic features supporting this diagnosis  Field-of-view What you can see How it supports OKC  Low-power (first image) • A single, well-circumscribed cystic space with a very thin, uniform epithelial lining only a few cell layers thick.  • The lining stays smooth without rete ridges and frequently appears detached from the fibrous wall (artefactual “cleft”). OKCs characteristically have a delicate, uniform epithelium (≈ 6–8 cells) and often show epithelial–stromal separation.  High-power (second image) • Corrugated, wavy surface layer of parakeratin projecting into the lumen, with retained pyknotic nuclei in the keratin sheets.  • Immediately beneath, a thin spinous zone and a palisaded basal layer of hyperchromatic, columnar nuclei that tend to polarise away from the basement membrane.  • No granular layer (rules out orthokeratinised cyst). The triad of corrugated parakeratin, palisaded basal cells, and thin epithelium is diagnostic of the parakeratinised OKC.  Cyst wall • Relatively bland, fibrous connective tissue with little or no chronic inflammation.  • (In other sections of typical OKCs one may find daughter / satellite cysts, but they are not required for the diagnosis.) A non-inflamed fibrous wall helps distinguish a developmental OKC from inflammatory cysts.  Taken together, the combination of a uniform thin lining, palisaded basal layer, corrugated parakeratin surface, and minimal inflammation is pathognomonic for a parakeratinised odontogenic keratocyst. |
| 14.35b | Diagnosis: Botryoid Odontogenic Cyst; Key Microscopic Features: The cyst features complex architecture with a uniformly thin lining containing many clear cells and epithelial plaques. | Based on the provided hematoxylin-and-eosin stained images of a lesion from the odontogenic cyst group, examined at different magnifications, the single most likely histopathological diagnosis is:  Glandular Odontogenic Cyst (GOC)  Key Microscopic Features Supporting the Diagnosis:  Non-keratinized stratified squamous epithelial lining:  Seen throughout the images, the epithelial lining is several cell layers thick and lacks keratinization, which is consistent with GOC.  Intraepithelial mucous/goblet cells:  Characteristic mucous-producing cells with pale cytoplasm are evident within the lining epithelium, as seen particularly in the second and third images.  Intraepithelial microcysts or duct-like spaces:  Numerous gland-like (ductal) or crypt-like microcystic spaces are seen within the epithelial lining, a hallmark feature of GOC. These are visible especially in the second and fourth images.  Surface hobnail or cuboidal cells:  Some areas show a superficial layer of cuboidal to columnar cells with eosinophilic cytoplasm, often referred to as "hobnail" cells—another characteristic of GOC.  Thickened epithelial plaques/focal nodular thickenings:  Seen as focal areas of epithelial thickening or plaque-like projections into the lumen.  Supporting fibrous connective tissue wall:  The cyst wall consists of mature fibrous connective tissue, without significant inflammation or atypia.  Summary:  The constellation of goblet cells, intraepithelial microcysts/duct-like structures, non-keratinized epithelium, and surface hobnail cells, all support the diagnosis of a Glandular Odontogenic Cyst. This is a rare but distinct odontogenic cyst with potential for recurrence, and these features help differentiate it from other cystic odontogenic lesions like lateral periodontal cyst, botryoid odontogenic cyst, and low-grade mucoepidermoid carcinoma. | Histopathological diagnosis  Glandular odontogenic cyst (GOC)  Key microscopic features supporting this diagnosis  Feature seen in the slides Diagnostic significance  Variable-thickness, non-keratinised stratified squamous lining (ranges from only a few cells to broad plaques and downgrowths) Typical of GOC; the lining is not uniformly thin like a lateral periodontal cyst and lacks keratinisation of an OKC  Intra-epithelial microcysts / duct-like spaces filled with pale eosinophilic material “Gland-like” structures are the hallmark of a glandular odontogenic cyst  Surface hob-nail (columnar/cuboidal) cells with apical snouts Characteristic of GOC and uncommon in other odontogenic cysts  Epithelial plaque-like thickenings & papillary projections extending into the lumen and into the wall Another classic finding, helping to distinguish GOC from other cysts that have a uniformly thin lining  Scattered mucous (goblet-type) cells and clear cells within the lining (best appreciated on high power) Presence of mucous cells is a defining criterion in most classification schemes for GOC  Relatively bland fibro-collagenous cyst wall with little inflammation Consistent with a developmental (rather than inflammatory) odontogenic cyst  The constellation of a variably thick, non-keratinised odontogenic epithelium containing hob-nail cells, mucous cells and numerous intra-epithelial microcystic/duct-like spaces is diagnostic of a glandular odontogenic cyst. |
| 14.37a | Diagnosis: Orthokeratinized Odontogenic Cyst; Key Microscopic Features: The cyst is lined by thick orthokeratinized epithelium of variable thickness, with no inflammation in the wall. | Based on the provided hematoxylin and eosin (H&E) stained histopathological images at different magnifications, the most likely diagnosis is:  Glandular Odontogenic Cyst (GOC)  Key Microscopic Features Supporting the Diagnosis:  Non-keratinized stratified squamous epithelial lining:  The lining epithelium is relatively uniform in thickness and lacks keratinization, which is a key feature in distinguishing GOC from other odontogenic cysts like odontogenic keratocyst (OKC).  Cuboidal to columnar basal cells:  The basal cell layer shows cuboidal to columnar cells with hyperchromatic nuclei, occasionally arranged in a palisaded manner.  Surface hobnail (eosinophilic columnar) cells:  At higher magnification (second image), there are hobnail cells protruding into the lumen — eosinophilic columnar cells with apical nuclei — which are characteristic of GOC.  Mucous (goblet) cells and intraepithelial mucin pools:  Mucous cells with foamy cytoplasm are visible, and mucin pools within the epithelium can sometimes be seen, although not distinctly evident in this image set.  Intraepithelial microcysts or duct-like spaces:  These pseudoglandular structures or small cystic spaces are diagnostic of GOC and visible in the images, especially in the first image, which shows multilocular architecture.  Epithelial plaques or thickenings:  Focal epithelial thickenings (plaques) can be appreciated in parts of the cystic lining.  Summary:  The combination of non-keratinized epithelial lining, hobnail cells, mucous cells, duct-like spaces, and epithelial plaques are diagnostic for Glandular Odontogenic Cyst. GOC is a rare but important odontogenic cyst due to its potential for recurrence and its histological overlap with low-grade mucoepidermoid carcinoma, making accurate diagnosis crucial. | Histopathological diagnosis (single best):  Glandular odontogenic cyst (GOC, “sialo-odontogenic cyst”)  Key microscopic features visible in the two H-E sections that support this diagnosis  # Feature (seen in the photomicrographs) Diagnostic significance  1 Non-keratinised, stratified squamous epithelial lining of variable thickness (≈ 2–10 cell layers) with prominent folding and papillary projections into the cyst lumen A hallmark of GOC; the lining is seldom uniformly thin like a lateral periodontal cyst and shows a wavy/papillary contour rather than the flat surface of most developmental cysts.  2 Surface (luminal) layer composed of cuboidal–columnar “hob-nail” cells; several are ciliated (fine apical hair-like projections can be appreciated focally) Ciliated columnar cells are unusual in odontogenic cysts but occur characteristically in GOC.  3 Scattered mucous (goblet) cells with intracytoplasmic clear mucin vacuoles (one prominent pale vacuolated cell is evident in the higher-power image) Presence of true mucous cells is a defining feature of GOC, not seen in radicular, dentigerous, or lateral periodontal cysts.  4 Intra-epithelial crypts / microcysts and duct-like spaces (small clear spaces within the epithelium, best appreciated in the low-power image as tiny lumina) These gland-like structures give the lesion its name; they are a required microscopic criterion.  5 Focal plaque-like epithelial thickenings (localized bulges of epithelium into the connective tissue wall) Another typical architectural change of GOC.  6 Fibro-collagenous cyst wall with only mild chronic inflammation Fits a developmental rather than purely inflammatory cyst and helps rule out an inflamed radicular cyst.  The concurrence of a non-keratinised, variably thick epithelium containing both mucous and ciliated cells, together with intra-epithelial microcysts/duct-like structures and plaque-like thickenings, is diagnostic for a glandular odontogenic cyst. |
| 14.37b | Diagnosis: Orthokeratinized Odontogenic Cyst; Key Microscopic Features: The cyst is enucleated and filled with keratin, and the epithelium is orthokeratinized with a thick granular layer and no palisading of basal cell nuclei. | Based on the provided hematoxylin-and-eosin (H&E) stained histological images of an odontogenic cystic lesion, the most likely diagnosis is:  Diagnosis: Glandular Odontogenic Cyst (GOC)  Key Histopathological Features Supporting the Diagnosis:  Non-keratinized Stratified Epithelium:  The cyst lining is composed of non-keratinized stratified squamous epithelium, which is typical for GOC.  The epithelial lining varies in thickness, which is a hallmark of this lesion.  Columnar/Cuboidal Cells with Apocrine Snouting:  Areas of the lining show cuboidal to columnar cells with evidence of apocrine-like snouting (eosinophilic cytoplasmic projections into the lumen), suggestive of glandular differentiation.  Intraepithelial Microcysts and Duct-like Spaces:  Presence of intraepithelial microcystic or duct-like structures, another characteristic feature of GOC.  These spaces can mimic glandular or mucous structures, supporting the glandular designation.  Mucous (Goblet) Cells:  Some regions appear to show mucous-producing (goblet) cells within the epithelial lining. These cells have pale-staining cytoplasm and basally located nuclei.  Cilia:  Although not clearly seen in all areas, cilia may be present on the surface epithelium in some glandular odontogenic cysts, adding to the respiratory-type differentiation.  Fibrous Cyst Wall:  The cyst is surrounded by a fibrous connective tissue wall, which lacks inflammation in the sections shown—typical of developmental cysts. | Histopathological diagnosis  → Glandular odontogenic cyst (GOC, “sialo-odontogenic” cyst)  Key microscopic features demonstrated in the two photomicrographs  # Feature as seen in the images Significance for GOC  1 Lining epithelium is non-keratinised and of variable thickness (≈ 3 – 8 cell layers) – in some areas thin, in others thicker and pseudo-stratified. GOC characteristically shows an uneven, non-keratinising epithelial lining rather than a uniform keratinised layer.  2 Surface columnar / “hob-nail” cells, many with apical snouting; several cells show cilia. These tall surface cells, sometimes ciliated, give the lining a pseudo-stratified respiratory-like look that is typical for GOC.  3 Intra-epithelial mucous (goblet) cells and micro-cystic / duct-like spaces – round, pale vacuoles containing pink mucin are obvious within the epithelial thickness (best seen in the lower-power image, left side). The presence of mucous cells plus true micro-cysts or duct-like lumina within the epithelium is the hallmark “glandular” component of GOC and separates it from other odontogenic cysts.  4 Papillary / tuft-like projections of epithelium into the cyst lumen – delicate finger-like extensions are present in several foci. Papillary tufts are another classic architectural feature of GOC.  5 Plaque-like epithelial thickenings – focal bulbous proliferations of the lining (seen toward the right side of the low-power field). These plaques are frequently described in GOC and support the diagnosis.  6 Fibro-collagenous cyst wall with minimal inflammation; no obvious keratin debris. GOC walls are usually paucicellular and only mildly inflamed, helping to exclude inflammatory odontogenic cysts or keratinising variants.  The unique combination of a non-keratinised but “gland-like” epithelial lining (hob-nail/ciliated surface cells, mucous cells, intra-epithelial micro-cysts, papillary tufts, and plaque-like thickenings) precisely matches the diagnostic criteria for a glandular odontogenic cyst and distinguishes it from other jaw cysts such as nasopalatine duct, radicular, dentigerous, or keratocystic lesions. |
| 14.40 | Diagnosis: Glandular Odontogenic Cyst; Key Microscopic Features: The cyst contains multiple structures lined by squamous cells with ductlike formations and few mucous cells, and the luminal cells show secretory activity. | Based on the provided hematoxylin and eosin (H&E) stained histological images at low and high magnification, the single most likely histopathological diagnosis is:  Glandular Odontogenic Cyst (GOC)  Key Microscopic Features Supporting the Diagnosis:  Non-keratinized Stratified Squamous Epithelium:  The cyst lining is composed of a non-keratinized stratified squamous epithelium of variable thickness, a hallmark of GOC.  Epithelial Plaques and Whorls:  Localized epithelial thickenings (plaques) are visible, a characteristic feature of GOC.  Cuboidal and Columnar Cells with Ciliated Cells:  The high-power image shows focal areas with cuboidal and columnar cells, including some that appear ciliated, which is typical of GOC and helps distinguish it from other odontogenic cysts.  Mucous (Goblet) Cells:  Mucous-producing cells (goblet cells) are identifiable in the higher magnification image, showing clear cytoplasmic vacuolation. These cells are diagnostic for GOC and are not typically present in other odontogenic cysts.  Glandular or Duct-like Structures:  There are areas within the lining epithelium that resemble glandular or duct-like spaces, reinforcing the glandular differentiation implied in the name.  Epithelial Lining Intruding into Connective Tissue:  Some projections of epithelial lining appear to intrude into the cyst wall, adding to the complexity of the epithelial architecture.  Differential Diagnoses Considered but Less Likely:  Lateral Periodontal Cyst: May show focal thickening and clear cells but lacks mucous cells and glandular features.  Botryoid Odontogenic Cyst: A multicystic variant of lateral periodontal cyst, still lacking mucous and ciliated cells.  Mucoepidermoid Carcinoma (central variant): Could mimic GOC, but lacks the benign cystic features seen here and shows more solid tumor architecture and cellular atypia. | Histopathological diagnosis – Glandular Odontogenic Cyst (GOC)  Key microscopic features visible in the two H-E sections  # Feature (as seen in the photos) Significance for GOC  1 Non-keratinised stratified squamous lining of highly variable thickness – ranges from only a few cell layers to broad plaques 10-12 cells thick. GOC characteristically shows a patchy, uneven epithelial thickness rather than a uniformly thin lining.  2 Intra-epithelial duct-like / microcystic spaces – multiple round to elongated lumen‐like structures, some with eosinophilic material, lined by a single layer of cuboidal or columnar cells (“ducts”). The presence of true or pseudo-ducts is one of the most diagnostic clues for GOC.  3 “Hob-nail” surface cells and mucous (goblet) cells – cells with apically bulging, eosinophilic cytoplasm and vacuolated, pale cytoplasm scattered within the lining (best appreciated in the higher-power image). Hob-nail cells and mucous/goblet cells are hallmarks that link the lesion to gland-like (sialo-odontogenic) differentiation.  4 Epithelial plaques/whorls – focal, plaque-like epithelial thickenings that bulge into the cyst lumen or into the connective tissue wall. Another classic GOC feature, often helps separate it from lateral periodontal cyst or other developmental cysts.  5 Fibro-collagenous cyst wall with mild chronic inflammation and haemorrhage – no satellite cysts or dentinoid; stroma is otherwise bland. Supportive background; absence of aggressive features (e.g., keratin lamellae of OKC or tumor-like epithelial proliferation of AOT).  Together, the combination of variable‐thickness epithelium, abundant intra-epithelial ducts/microcysts, hob-nail and mucous cells, and epithelial plaques is diagnostic of a Glandular Odontogenic Cyst and not typical of other entities in the odontogenic cyst spectrum |
| 14.41 | Diagnosis: Glandular Odontogenic Cyst; Key Microscopic Features: The cyst is lined by thick, nonkeratinized stratified squamous epithelium with scattered ductal structures and luminal cells containing eosinophilic cytoplasm and mucous cells. | Based on the provided hematoxylin and eosin (H&E) stained histopathology images at different magnifications, the most likely diagnosis is:  Diagnosis:  Radicular Cyst (Periapical Cyst)  Justifying Microscopic Features:  Low Magnification (Image 1):  Cystic architecture: A well-circumscribed cystic lesion lined by epithelium.  Inflammatory infiltrate: Dense chronic inflammatory infiltrate, primarily lymphocytes and plasma cells, within the cyst wall.  Epithelial hyperplasia: The lining epithelium appears thickened and proliferative in areas, with some irregular rete ridge formation.  High Magnification (Image 2):  Non-keratinized stratified squamous epithelium: Typical of radicular cysts.  Spongiosis and exocytosis: Some areas show intraepithelial inflammatory cells and intercellular edema.  Inflammatory cells in connective tissue: Marked infiltration of lymphocytes and plasma cells in the underlying connective tissue.  Russell bodies or foamy macrophages may be suggested but not clearly seen — common in chronically inflamed cysts. | Most likely histopathological diagnosis  → Glandular odontogenic cyst (GOC, “sialo-odontogenic cyst”)  Key microscopic features in the two H&E sections that support this diagnosis  Feature (with image reference) How it appears Diagnostic significance  Variable-thickness, non-keratinised epithelial lining (both magnifications) Epithelium ranges from only a few cell layers to broad plaque-like swellings that “arcade” into the lumen A classic, constantly changing lining typical of GOC  Intra-epithelial microcysts / duct-like spaces (low-power image, left half) Numerous round to oval empty spaces within the epithelial plaques Gives the lesion its “glandular” character; uncommon in other odontogenic cysts  Mucous (goblet) cells (low-power image – pale, vacuolated cells; high-power image – clear cytoplasm pushing nuclei to the side) Clusters of mucin-filled vacuoles scattered through the lining Essential criterion for GOC and rarely seen in inflammatory cysts  Ciliated / hob-nail surface cells (high-power image, luminal edge) Tall columnar cells with apical cilia and protruding nuclei Another hallmark change produced by respiratory-type metaplasia within GOC  Epithelial plaques / whorls (low-power image, mid-field) Solid bulbous tongue-like thickenings of squamoid cells projecting into the cyst lumen Characteristic nodular growth unique to GOC and lateral periodontal cyst, but coupled with mucous & ciliated cells points to GOC  Fibro-collagenous wall with chronic inflammatory infiltrate (both images, cyst wall) Dense lymphoplasmacytic infiltrate beneath the lining Secondary inflammation is common but not necessary for diagnosis  The simultaneous presence of (1) mucous cells, (2) ciliated surface cells, (3) intra-epithelial microcysts/ducts, and (4) plaque-like epithelial thickenings in an odontogenic cyst conclusively favours a glandular odontogenic cyst over other entities such as a radicular cyst, lateral periodontal cyst, or odontogenic keratocyst. |

ODONTOGENIC TUMORS

| **Fig_no** | **Ground truth** | **GPT 4o answer** | **o3 answer** |
| --- | --- | --- | --- |
| 15.3 | Diagnosis: Keratocystic odontogenic tumor/odontogenic keratocyst; Key Microscopic Features: Cystic tumor with a thin epithelial lining and fibrous wall with minimal inflammation. The epithelium is uniformly thin with areas of detachment. | Based on the provided hematoxylin-and-eosin (H&E) stained images of the lesion, observed at low and high magnification, the single most likely histopathological diagnosis is:  Ameloblastoma (Follicular Type)  Key Microscopic Features Supporting the Diagnosis:  Low Magnification Image (First Image):  Multilocular cystic appearance: The lesion shows multiple cystic and solid areas, separated by fibrous septa.  Islands of odontogenic epithelium (black arrows): These are scattered throughout the fibrous stroma and have peripheral columnar cells and central stellate reticulum-like cells.  Infiltrative pattern: The epithelial islands are irregularly shaped and appear to invade the surrounding connective tissue.  High Magnification Image (Second Image):  Peripheral palisading of columnar cells with reverse nuclear polarization (nuclei away from the basement membrane), a hallmark of ameloblastoma.  The central region of the epithelial islands resembles the stellate reticulum of the enamel organ—loosely arranged, angular cells.  Lack of significant mitotic activity or cellular atypia, supporting a benign, though locally aggressive, neoplasm. | Histopathological diagnosis  → Unicystic ameloblastoma – intraluminal / plexiform (“plexiform-type”) variant  Key microscopic features that point to this diagnosis  Field Features seen in the submitted photomicrographs How they support the diagnosis  Low-power architecture (first image) Single large cystic lumen whose wall is repeatedly folded; from the lining arise long, ribbon-like and leaf-like projections with central fibrous cores (black arrows). The epithelial projections anastomose, creating a plexiform / papillary intraluminal growth pattern. A unilocular cyst with an intraluminal plexiform proliferation is characteristic of the intraluminal type of unicystic ameloblastoma. Other odontogenic tumours rarely show such extensive, orderly intraluminal plexiform sheets.  Epithelial lining (second image, high power) Lining epithelium is 8-10 cells thick. The basal layer is composed of tall columnar cells with palisaded, hyperchromatic nuclei and occasional reverse polarity; suprabasal cells are loosely arranged and resemble stellate reticulum. No surface keratinisation is present. The combination of palisaded, polarised basal cells and stellate-reticulum-like suprabasal cells is diagnostic of ameloblastomatous epithelium and rules out odontogenic keratocyst, simple cysts or fibromas.  Relationship to wall Tumour epithelium remains confined to the cyst lumen; no infiltrating islands are present in the fibrous wall. The supporting stroma is a relatively bland fibrous tissue. Confirms the intraluminal (non-mural) variant of unicystic ameloblastoma, distinguishing it from conventional solid/multicystic ameloblastoma.  Collectively, these features – a unilocular cyst, ameloblastomatous lining with classical basal cell palisading, and extensive intraluminal plexiform projections – are diagnostic of a unicystic ameloblastoma, intraluminal / plexiform subtype. |
| 15.4 | Diagnosis: Keratocystic odontogenic tumor/odontogenic keratocyst; Key Microscopic Features: The cyst lining is disrupted in areas with inflammation, showing spongiosis and leukocyte migration. | Based on the histopathological examination of the provided hematoxylin and eosin (H&E) stained images at different magnifications, the single most likely diagnosis is:  Lateral Periodontal Cyst (Botryoid variant)  Key Microscopic Features Justifying the Diagnosis:  Cystic Architecture with Thin, Non-Keratinized Epithelium:  The cyst lining is composed of 1–5 layers of cuboidal to squamous epithelial cells, with a flat interface with the surrounding connective tissue.  The epithelium is non-keratinizing, which helps distinguish it from odontogenic keratocysts.  Focal Plaque-Like Epithelial Thickenings:  Characteristic epithelial plaques or thickenings are seen projecting into the cyst lumen or wall—often described as “dental lamina-like.”  These thickenings contain clear cells with glycogen-rich cytoplasm (poorly stained with H&E but visible as pale areas).  Multicystic Appearance (Botryoid Pattern):  The low-power image reveals a multilocular or grape-like (botryoid) configuration of cystic spaces.  This feature defines the botryoid variant, which is a polycystic form of lateral periodontal cyst.  Minimal Inflammation:  The surrounding connective tissue capsule shows mild chronic inflammatory infiltrate, but the inflammation is not prominent—typical of developmental cysts rather than inflammatory ones.  Location and Association with Vital Teeth:  Although this cannot be confirmed histologically, this cyst type is most often located between the roots of vital mandibular premolars, aiding the clinicopathologic correlation.  Differential Diagnosis Considered and Ruled Out:  Odontogenic Keratocyst (OKC):  Ruled out due to absence of parakeratinized corrugated lining and palisaded basal layer.  Glandular Odontogenic Cyst:  Ruled out due to lack of mucous/goblet cells, gland-like spaces, and ciliated epithelium.  Radicular Cyst:  Ruled out due to lack of inflammatory etiology and absence of a non-vital tooth history.  Dentigerous Cyst:  Typically surrounds the crown of an unerupted tooth—no such relationship is visible here.  If clinical data supports the location adjacent to the roots of vital teeth in a middle-aged adult, this reinforces the diagnosis of Botryoid Odontogenic Cyst (Lateral Periodontal Cyst variant). | Histopathological diagnosis  → Unicystic ameloblastoma (luminal / intraluminal type)  Microscopic features in the two fields that support this diagnosis  Low-power features High-power features  • A single, well-defined cystic cavity is present; the lining epithelium folds and buds into the lumen, giving it an undulating/plexiform contour. • Basal layer composed of tall columnar, palisaded odontogenic cells; nuclei are hyper-chromatic and show reverse polarity (shifted away from the basement membrane).  • The cyst wall is fibrous and shows a dense chronic inflammatory (lymphoplasmacytic) infiltrate, a common secondary change in unicystic lesions. • Sub-nuclear vacuolation is seen in many basal cells, another hallmark of ameloblastic epithelium.  • Proliferating epithelial tongues project into the lumen but remain attached to the lining (intraluminal pattern), without clear infiltration of the fibrous wall. • Overlying the basal layer, the epithelium becomes loosely arranged and stellate-reticulum-like, mimicking the enamel organ.  • No significant cytologic atypia or brisk mitotic activity—findings expected in a benign ameloblastoma rather than a carcinoma.  These combined features—an ameloblast-type cyst lining with palisaded, reverse-polarized basal cells, sub-nuclear vacuoles, overlying stellate-reticulum-like cells, and intraluminal epithelial proliferations—are diagnostic of a unicystic ameloblastoma rather than a simple inflammatory odontogenic cyst or another odontogenic tumour variant. |
| 15.5 | Diagnosis: Keratocystic odontogenic tumor/odontogenic keratocyst; Key Microscopic Features: Presence of numerous satellite cysts in the wall, with some forming keratin pearls, which should not be confused with squamous cell carcinoma. | Histopathological Diagnosis:  Ameloblastoma (specifically Follicular Type)  Key Microscopic Features Justifying the Diagnosis:  The diagnosis of follicular ameloblastoma, a benign but locally aggressive odontogenic tumor, is supported by the following classic histopathological features observed in the provided H&E-stained images:  Low-Power View (First Image):  Multilocular growth pattern: The tumor displays a lobulated architecture with multiple epithelial islands embedded within a fibrous connective tissue stroma.  Cystic degeneration: Central areas of some follicles exhibit cystic changes, a common feature in ameloblastomas.  High-Power View (Second Image):  Follicular arrangement: Islands of odontogenic epithelium are present, mimicking the enamel organ.  Peripheral palisading of columnar cells: The outer layer of tumor nests is composed of tall, columnar cells with reverse polarization (nuclei oriented away from the basement membrane), resembling ameloblasts.  Central stellate reticulum-like cells: The center of the epithelial islands contains loosely arranged, angular cells mimicking the stellate reticulum of the enamel organ.  Lack of significant cellular atypia or mitoses, consistent with the benign nature of ameloblastoma. | Histopathological diagnosis – single best answer  Conventional (solid/multicystic) ameloblastoma, follicular pattern with acanthomatous change  Key microscopic features that point to this diagnosis  Field Salient finding Significance  Low-power architecture Multilobulated, expansile lesion composed of many epithelial nests/follicles separated by a collagenous stroma; several follicles show central cystic degeneration. The lobulated “soap-bubble” outline is typical of a solid/multicystic ameloblastoma.  Peripheral cell layer of follicles Tall columnar to cuboidal cells arranged in a continuous palisade; nuclei are hyperchromatic and uniformly polarized away from the basement membrane with occasional sub-nuclear vacuolation. Peripheral palisading with reverse nuclear polarity is the hallmark of ameloblastic epithelium.  Central portion of follicles Loosely arranged stellate-reticulum-like cells; many follicles show extensive squamous (acanthomatous) metaplasia with formation of keratin pearls/eosinophilic whorls. Stellate reticulum-like centres define the follicular pattern; overt squamous metaplasia identifies the acanthomatous variant.  Cystic change Variable cystic degeneration of some epithelial nests and formation of larger intratumoural cystic spaces. Explains the multilocular radiolucency seen radiographically and is common in conventional ameloblastoma.  Stroma Relatively mature fibrous connective tissue without the primitive, myxoid appearance seen in mixed odontogenic tumours (e.g., ameloblastic fibroma). Helps exclude mixed epithelial–mesenchymal tumours.  Cytology Uniform basaloid to squamoid cells, minimal atypia, low mitotic activity. Supports a benign conventional ameloblastoma rather than an ameloblastic carcinoma.  Together, these classic features—peripheral palisaded ameloblastic cells with reverse polarity, central stellate-reticulum-like areas undergoing prominent squamous (acanthomatous) metaplasia, and a fibrous stroma—pin the lesion down to a follicular (acanthomatous) ameloblastoma, the most common conventional solid/multicystic odontogenic tumour of the jaws. |
| 15.5b | Diagnosis: Keratocystic odontogenic tumor/odontogenic keratocyst with satellite cysts; Key Microscopic Features: Some satellite cysts contain macrophages in their lumen. | Based on the histopathological evaluation of the provided hematoxylin-and-eosin (H&E) stained images at low and high magnification, the most likely diagnosis is:  Diagnosis: Keratocystic Odontogenic Tumor (KCOT)  (now more commonly termed as Odontogenic Keratocyst - OKC)  Key Microscopic Features Supporting the Diagnosis:  Cystic Architecture:  Multiple cystic spaces lined by stratified squamous epithelium.  The lining appears relatively thin and uniform.  Corrugated Parakeratinized Surface:  The high-power image clearly shows a corrugated (wavy) surface of parakeratin (compact eosinophilic surface with retained nuclei).  This is a hallmark of OKC.  Palisaded Basal Layer:  Basal epithelial cells are cuboidal to columnar and show a palisaded arrangement, appearing hyperchromatic.  This is distinctive for OKC.  Satellite/Daughter Cysts:  Smaller cystic structures seen in the connective tissue wall, indicating potential for recurrence—another characteristic feature of OKC.  Minimal Inflammatory Infiltrate:  In some regions, a mild chronic inflammatory infiltrate is present, though not intense, which is commonly seen in non-secondarily infected OKCs.  Differential Diagnosis Considered (but less likely based on features):  Ameloblastoma – lacks the thin parakeratinized epithelial lining and typically shows islands or plexiform nests of ameloblastic epithelium.  Orthokeratinized Odontogenic Cyst – has orthokeratin (no nuclei in keratin layer) and lacks palisaded basal cells.  Dentigerous cyst – has a non-keratinized epithelial lining and lacks the above features. | Histopathological diagnosis (single best):  Acanthomatous (keratinising) ameloblastoma  Key microscopic features visible in the two H-E sections  Feature Where it is seen in the images Importance for the diagnosis  Multilocular / multicystic architecture formed by many epithelial follicles and cystic spaces Low-power image: numerous round to oval spaces of different sizes occupying almost the whole specimen Fits the “conventional-type” ameloblastoma, which is typically multicystic rather than a single cyst wall (rules out odontogenic keratocyst)  Follicular islands of odontogenic epithelium embedded in a fibrous stroma Both magnifications Characteristic growth pattern of ameloblastoma  Peripheral palisaded layer of tall columnar “ameloblast-like” cells with nuclei polarized away from the basement membrane (reverse polarity) High-power image, especially at the margins of the epithelial nests (bottom-right island) Pathognomonic for ameloblastoma  Central stellate-reticulum-like cells that have undergone marked squamous metaplasia with formation of large lamellated keratin pearls High-power image: the concentric pink whorl of keratin; many smaller keratin pearls in other follicles Defines the acanthomatous (keratinising) variant of ameloblastoma  Intraluminal keratin debris filling some cystic spaces Low-power image: intensely eosinophilic material within several lumina Supports keratinising (acanthomatous) behaviour  Lack of ghost cells, duct-like structures, or hard-tissue formation Both images Helps exclude calcifying odontogenic cyst / dentinogenic ghost cell tumour, adenomatoid odontogenic tumour, etc.  Moderate chronic inflammatory infiltrate in the surrounding stroma High-power image (dense lymphoplasmacytic infiltrate) A common secondary change in long-standing ameloblastomas  Taken together, the classic ameloblastic follicular architecture plus extensive squamous (acanthomatous) change and keratin-pearl formation point squarely to an acanthomatous ameloblastoma. |
| 15.8 | Diagnosis: Ameloblastoma, follicular pattern; Key Microscopic Features: The follicles exhibit cystic changes with mature fibrous stroma surrounding them. There is basal cell palisading, reverse nuclear polarization, and acanthomatous cells. | Based on the provided hematoxylin-and-eosin (H&E) stained images taken at different magnifications, the most likely histopathological diagnosis is:  Ameloblastoma (Conventional/Multicystic Type)  Key Microscopic Features Supporting the Diagnosis:  Islands and Strands of Odontogenic Epithelium:  The lesion shows numerous epithelial islands within a fibrous stroma.  These islands have a characteristic peripheral palisading of columnar or cuboidal cells with reverse nuclear polarity (nuclei oriented away from the basement membrane).  Stellate Reticulum-Like Cells in the Center:  The central cells within the epithelial islands resemble the stellate reticulum, with loosely arranged, angular, and pale-staining cells.  This biphasic pattern (peripheral columnar cells and central stellate cells) is a hallmark of ameloblastoma.  Cystic Degeneration:  Areas of cystic spaces or degeneration within epithelial islands are visible, often associated with the follicular pattern of ameloblastoma.  Follicular and Plexiform Patterns:  Both follicular (islands with central stellate cells) and areas showing more plexiform (interconnecting strands and cords of epithelium) growth patterns can be seen.  This is typical of the conventional type of ameloblastoma.  Fibrous Connective Tissue Stroma:  The stroma is relatively mature, fibrous, and non-inflamed, supporting the epithelial component. | Histopathological diagnosis  Adenomatoid odontogenic tumour (AOT) – most likely an intra-/extra-follicular variant (benign epithelial odontogenic tumour)  Key microscopic features supporting this diagnosis  Feature seen in the photomicrographs Diagnostic relevance  Numerous duct-/tubule-like structures (“adenomatoid” pattern) lined by a single layer of cuboidal-to-columnar epithelial cells Characteristic architectural hallmark of AOT; distinguishes it from the follicular patterns of a conventional ameloblastoma  Polarised (“reverse-polarity”) nuclei of the lining cells – nuclei located away from the luminal surface Typical cytologic feature of AOT and of ameloblastic epithelium in general  Rosette- and whorl-shaped epithelial nests with central spaces, some heart-shaped or stellate Gives the tumour its gland-like appearance and helps separate it from sheet-like lesions such as the Pindborg (CEOT) tumour  Eosinophilic hyaline material and small “snow-flake” foci of calcification within many lumina Highly characteristic of AOT; calcifications may be dystrophic or Liesegang-ring–type  Solid spindle-shaped epithelial cell bundles merging with the ductal areas Another classic component of AOT (“two-cell pattern”)  Dense, often hyalinised fibrous stroma without a myxoid primitive ectomesenchyme Helps rule out mixed tumours like ameloblastic fibroma or dentinogenic ghost-cell tumour  Lack of significant cytologic atypia or mitotic activity Supports a benign odontogenic tumour rather than an ameloblastic carcinoma or clear-cell odontogenic carcinoma  Taken together, the distinctive duct/rosette architecture, reverse-polarity columnar cells, luminal hyaline material with focal calcifications, and the absence of marked atypia strongly point to adenomatoid odontogenic tumour as the single best diagnosis. |
| 15.8b | Diagnosis: Ameloblastoma, follicular pattern with cystic change; Key Microscopic Features: Tumor islands with central cystic changes within mature stroma. There are areas resembling stellate reticulum, and eosinophilic luminal cells. | Based on the histopathological analysis of the three provided hematoxylin and eosin (H&E) stained images—captured at low, medium, and high magnifications—this lesion most closely resembles a:  Diagnosis:  Ameloblastoma  Key Microscopic Features Supporting the Diagnosis:  Follicular and Plexiform Patterns (Seen in Low Magnification Image):  Islands and cords of odontogenic epithelium within a fibrous stroma.  The follicular pattern shows nests of epithelium with peripheral palisading and reverse polarization of basal cells.  Central areas of stellate reticulum-like cells are present.  The plexiform pattern displays interconnecting strands of odontogenic epithelium.  Peripheral Palisading of Columnar Cells (Seen in Medium and High Magnification):  Peripheral cells of the epithelial islands are tall columnar and exhibit nuclear polarization away from the basement membrane (reverse polarity), a hallmark of ameloblastoma.  Central Stellate Reticulum-like Areas:  The centers of the epithelial islands resemble the stellate reticulum of the enamel organ, with loosely arranged angular cells.  No Evidence of Inductive Changes or Dental Hard Tissue Formation:  Absence of dentin or enamel matrix distinguishes this from other odontogenic tumors like adenomatoid odontogenic tumor or ameloblastic fibro-odontoma.  Mature Fibrous Stroma:  The connective tissue stroma is mature and collagenous, sometimes separating the epithelial components. | Histopathological diagnosis (single best):  Adenomatoid odontogenic tumor (AOT)  Key microscopic (H-E) features supporting this diagnosis  Field What is seen Why it points to AOT  Low-power architecture Well-circumscribed, capsule-like peripheral band of dense collagen; tumor composed of intertwining cords, strands and nodules of odontogenic epithelium set in a markedly fibrous stroma AOTs are usually encapsulated and show a conspicuous collagenous stroma surrounding and separating epithelial nests  Epithelial pattern Numerous duct-like / tubular structures: round-to-elongated lumina lined by a single layer of cuboidal-to-columnar cells whose nuclei are polarized away from the lumen The pseudoduct or “adenomatoid” pattern is the diagnostic hallmark of AOT and is rarely prominent in other odontogenic tumors  Rosettes & whorls Solid epithelial whorls and rosette-like formations of spindle-shaped epithelial cells between the ducts Whorled sheets plus ducts create the classic mixed solid-glandular look of AOT  Luminal contents Eosinophilic, amorphous (“hyaline”) material within many tubules; some foci show beginning calcification Luminal hyaline globules with or without dystrophic calcification are characteristic of AOT  Stroma Densely collagenized, paucicellular connective tissue; scant inflammatory cells Further supports AOT over other mixed tumors (e.g., ameloblastic fibroma has loose myxoid stroma)  Relationship to surface epithelium Tumor islands lie beneath an overlying stratified squamous epithelium without invasion of that surface layer Typical for an intrabony, encapsulated odontogenic lesion like AOT  Taken together, the encapsulated lesion with abundant collagen, distinctive duct-like/pseudoglandular structures lined by polarized columnar cells, whorled epithelial nodules, and intraluminal hyaline/calcifying material is diagnostic of an adenomatoid odontogenic tumor. |
| 15.9 | Diagnosis: Ameloblastoma, plexiform pattern; Key Microscopic Features: The tumor forms interlacing trabeculae and strands enclosing fibrovascular tissue. Tumor strands show focal nuclear palisading and reverse polarization, with myxoid stroma. | Based on the provided hematoxylin and eosin (H&E) stained histopathological images of a lesion from the ODONTOGENIC TUMORS group, the most likely diagnosis is:  Ameloblastoma, Plexiform Type  Key Microscopic Features Justifying the Diagnosis:  Plexiform Pattern:  The low-power image (first image) shows interconnecting strands and cords of odontogenic epithelium, characteristic of the plexiform subtype of ameloblastoma.  These epithelial strands are bordered by a palisaded layer of columnar or cuboidal basal cells.  Peripheral Palisading:  High-power view (second image) reveals the peripheral cells arranged in a palisaded pattern with hyperchromatic, polarized nuclei, which is a classic feature of ameloblastoma.  Loosely Arranged Central Cells:  Within the strands, the central cells resemble stellate reticulum, appearing more loosely arranged and less organized, mimicking the enamel organ.  Fibrous Stroma:  The background stroma is fibrous and relatively mature, without significant inflammation or atypia, which is typical for ameloblastoma.  Absence of Odontogenic Hard Tissues:  No evidence of enamel or dentin formation, helping to differentiate it from odontomas or ameloblastic fibro-odontomas.  Differential Considerations Ruled Out:  Adenomatoid odontogenic tumor (AOT): lacks duct-like structures and calcifications.  Ameloblastic carcinoma: lacks cytological atypia or mitotic figures.  Odontogenic myxoma: lacks epithelial component. | Histopathological diagnosis (single best answer)  ➔ Desmoplastic ameloblastoma (desmoplastic-type/variant ameloblastoma)  Key microscopic features in the photographs that support this diagnosis  Field view Characteristic finding Diagnostic significance  Low-power overview Numerous thin, irregular cords and small islands of odontogenic epithelium rambling through a densely collagenised (“desmoplastic”) stroma The striking desmoplasia, with epithelium seemingly “squeezed” by dense collagen, is the defining architectural pattern of the desmoplastic variant.  Stroma Broad, pale-eosinophilic osteoid / mature bone trabeculae with empty osteocyte lacunae (visible because the section is decalcified) Foci of osseous metaplasia are present in roughly half of desmoplastic ameloblastomas and help separate it from conventional types and other odontogenic tumours.  Epithelial component – peripheral cells Lining cells are low columnar / cuboidal, only faintly palisaded and lack reverse polarity Unlike conventional follicular or plexiform ameloblastoma, the palisading is subtle, another hallmark of the desmoplastic variant.  Epithelial component – central cells Central cells in the islands are loosely arranged, sometimes squamoid, occasionally showing micro-cystic change Fits the typical epithelial morphology of desmoplastic ameloblastoma; no ghost cells, ducts, or amyloid to suggest other entities.  Interface epithelium–stroma Islands often show sharp collar-like condensation of stroma around them, and some are completely encased by newly formed bone The “osteoplasia” with engulfed epithelial nests is classic for this tumour.  Exclusion of close mimics  No ghost cells/dentinoid (rules out calcifying odontogenic cyst/tumour).  No dentin–enamel–primitive dental papilla combination (rules out ameloblastic fibro-odontoma).  No duct-like structures or amorphous amyloid with Liesegang rings (rules out adenomatoid odontogenic tumour and calcifying epithelial odontogenic tumour).  Taken together, the compressed odontogenic epithelium, the pronounced desmoplastic and partly ossified stroma, and the absence of features characteristic of other odontogenic tumours point squarely to desmoplastic ameloblastoma. |
| 15.10 | Diagnosis: Desmoplastic ameloblastoma; Key Microscopic Features: Large, irregular tumor islands and slender strands of squamoid cells within a dense stroma. Compressed islands contain basaloid cells, while others show squamous and peripheral basaloid cells. | Histopathological Diagnosis:  Adenomatoid Odontogenic Tumor (AOT)  Key Microscopic Features Supporting the Diagnosis:  Encapsulated Lesion (Visible in low-power image):  The tumor appears well-circumscribed and encapsulated, a characteristic finding in AOTs.  Spindle-Shaped and Cuboidal Epithelial Cells (Higher magnification):  Tumor cells are arranged in nodules or whorled patterns and composed of cuboidal to columnar epithelial cells with basophilic nuclei.  Rosette-like and Duct-like Structures:  Multiple pseudoductal or rosette-like structures lined by columnar epithelial cells with nuclei polarized away from the lumen, imitating glandular or ductal patterns—a hallmark of AOT.  Amyloid-like or Eosinophilic Material:  Areas containing eosinophilic, amorphous material are noted between epithelial cells, suggestive of enamel matrix or hyaline droplets often found in AOT.  Scattered Calcifications:  Though not prominent in every case, AOTs can exhibit small foci of calcifications, sometimes seen as dystrophic deposits.  Fibrous Stroma:  The stroma is relatively mature and loosely arranged, separating the epithelial nests without signs of aggressive infiltration.  Summary Justification:  The lesion's well-demarcated, encapsulated nature, coupled with duct-like structures formed by columnar cells, rosette formations, and eosinophilic material within a fibrous stroma, collectively support the diagnosis of Adenomatoid Odontogenic Tumor (AOT)—a benign epithelial odontogenic tumor most commonly affecting younger individuals, often in the anterior maxilla.  Let me know if you’d like a diagnostic report or comparison with similar odontogenic lesions (e.g., ameloblastoma or CEOT). | Histopathological diagnosis  Desmoplastic ameloblastoma (desmoplastic variant of ameloblastoma)  Key microscopic features that support this diagnosis  Feature seen in the photomicrographs Significance  Numerous small, irregular islands and narrow strands of odontogenic epithelium (dark-purple) scattered throughout the lesion A defining attribute of ameloblastoma; in the desmoplastic variant the epithelial component is fragmented into compressed nests and cords rather than large follicles  Dense, markedly collagenised (“desmoplastic”) stroma separating the epithelial islands The overwhelmingly eosinophilic, paucicellular, fibrous matrix is characteristic and gives the tumour its name  Peripheral cuboidal/low-columnar ameloblast-like cells with occasional nuclear palisading or reverse polarity Confirms the odontogenic (ameloblastomatous) nature of the epithelial nests  Central cells of nests showing stellate-reticulum-like or squamoid differentiation, sometimes with micro-cystic change Typical internal architecture of ameloblastoma islands, preserved despite compression  Irregular, stretched, “fish-hook” or “comma-shaped” epithelial strands within the dense stroma Classic silhouette produced by traction of the desmoplastic matrix on the epithelium  Lack of significant cytologic atypia or mitotic activity Supports a benign odontogenic tumour rather than carcinoma  Infiltrative margins without encapsulation (appreciated at low power) Usual growth pattern of ameloblastoma and explanation for its local aggressiveness  The combination of small compressed odontogenic epithelial islands and a conspicuously sclerotic/desmoplastic stroma is pathognomonic for the desmoplastic variant of ameloblastoma and distinguishes it from other odontogenic tumours such as conventional (follicular or plexiform) ameloblastoma, central odontogenic fibroma or calcifying epithelial odontogenic tumour. |
| 15.11 | Diagnosis: Follicular and plexiform ameloblastoma with granular cells; Key Microscopic Features: Islands of pale tumor cells are present, with basal cells showing reverse nuclear polarization and granular cytoplasm in the central cells. | Based on the provided hematoxylin and eosin (H&E) stained images at different magnifications, the histopathological diagnosis is most consistent with:  Diagnosis: Ameloblastoma (Conventional/Multicystic Type)  Key Microscopic Features Supporting the Diagnosis:  Low Magnification Image (First Image):  Follicular pattern: Multiple islands or follicles of odontogenic epithelium are seen embedded in a fibrous stroma.  Peripheral palisading: The outer layer of these epithelial islands shows columnar or cuboidal cells arranged in a palisaded fashion with reverse nuclear polarity (nuclei oriented away from the basement membrane).  Central stellate reticulum-like cells: The centers of these islands mimic the stellate reticulum of the enamel organ, consisting of loosely arranged angular cells with intercellular bridges.  High Magnification Image (Second Image):  Peripheral columnar cells with reversed polarity: High-power view confirms the peripheral layer of columnar cells showing hyperchromatic nuclei located away from the basement membrane.  Stellate reticulum-like central cells: The inner portion of the epithelial islands reveals loosely arranged cells resembling stellate reticulum.  Lack of significant cytologic atypia or mitotic figures, supporting a benign neoplasm rather than a malignant tumor.  Summary:  These features—particularly the follicular architecture, palisaded columnar peripheral cells, and central stellate reticulum-like areas—are classic for a conventional (multicystic) ameloblastoma, one of the most common odontogenic tumors. | Histopathological diagnosis  Calcifying epithelial odontogenic tumour (CEOT, ‘Pindborg tumour’)  Key microscopic features present in the two H&E fields that support this diagnosis  Low-power field High-power field Diagnostic relevance  Multiple, sharply-circumscribed lobules / islands of epithelial tumour cells separated by collagen-rich fibrous septa Tumour cells are polygonal with abundant eosinophilic, finely-granular cytoplasm and distinct intercellular borders / bridges Architecture and cell type typical of CEOT rather than ameloblastoma or AOT  Within many lobules there are central pools of pale, homogeneous, eosinophilic material; some show coarse to fine calcific foci that appear white/empty on the section In the same areas one sees extracellular amyloid-like deposits (pink, glassy) and early Liesegang-ring calcifications The combination of amyloid-like material that undergoes concentric calcification is pathognomonic for CEOT  Fibrous septa create a lobulated growth pattern but no peripheral palisading or stellate reticulum-like cells are evident Nuclei are often eccentrically placed, occasionally enlarged or pleomorphic, but mitoses are rare – a pattern classically described for CEOT Helps exclude other odontogenic tumours (e.g., ameloblastoma shows columnar peripheral palisading; AOT shows duct-like structures)  Together, the lobular arrangement of polyhedral epithelial cells containing extracellular amyloid with concentric (Liesegang-type) calcifications is diagnostic of a calcifying epithelial odontogenic tumour. |
| 15.11b | Diagnosis: Follicular and plexiform ameloblastoma with granular cells; Key Microscopic Features: Interlacing trabeculae of epithelium with palisaded basal nuclei and granular cells, along with areas resembling stellate reticulum. | Based on the histopathological features observed in the provided hematoxylin and eosin (H&E) stained images, the most likely diagnosis is:  Ameloblastic Fibro-Osteosarcoma  Justification & Key Microscopic Features:  The diagnosis of ameloblastic fibro-osteosarcoma is supported by the following key findings seen in the two magnifications:  1. Biphasic Tumor Composition:  Epithelial Component: The images show islands and cords of odontogenic epithelium resembling ameloblastic epithelium—this is a hallmark feature of ameloblastic fibro components.  Mesenchymal Component: Surrounding stroma is highly cellular and malignant-appearing, composed of spindle-shaped cells with pleomorphism, nuclear hyperchromasia, and increased mitotic activity.  2. Osteoid Formation:  Seen in the low magnification image, there are irregular eosinophilic osteoid matrix deposits, some with embedded malignant cells, indicating sarcomatous osteoid production.  These deposits are surrounded by malignant mesenchymal cells, supporting the osteosarcomatous differentiation.  3. Malignant Features in the Mesenchyme:  In the high magnification image, the mesenchymal cells show:  Marked cytologic atypia  Frequent mitotic figures  Hyperchromatic, pleomorphic nuclei  Infiltrative growth pattern  Conclusion:  The histologic combination of:  Benign-looking odontogenic epithelial islands,  Atypical, sarcomatous mesenchymal stroma,  And malignant osteoid formation,  is characteristic of ameloblastic fibro-osteosarcoma, a rare malignant mixed odontogenic tumor. | Most likely histopathological diagnosis  → Desmoplastic (collagenous) ameloblastoma  Key microscopic features visible in the two fields  Field Salient findings Importance for the diagnosis  Low-power (first image) • Tumour is broken up into irregular, angular islands and strands of epithelium that are compressed by an abundant, densely collagenised / hyalinised stroma.  • Within the stroma there are large, pale-eosinophilic, trabecular areas containing small lacuna-like spaces – consistent with metaplastic osteoid / bone formation often seen in this variant.  • Epithelial islands lack the classic tall columnar palisading of conventional follicular ameloblastoma and instead appear thin, cord-like or spindle-shaped. The desmoplasia (prominent collagen) that dominates the lesion and the compressed, irregular epithelial islands are the signature architecture of a desmoplastic ameloblastoma. Reactive bone formation further supports this variant.  High-power (second image) • Epithelial nests are composed of small cuboidal to spindle cells with hyperchromatic nuclei; peripheral palisading is minimal or absent.  • Many epithelial cells show eosinophilic, vacuolated or clear cytoplasm, and the nests merge imperceptibly with the surrounding collagen.  • No significant cytologic atypia or high mitotic activity, supporting a benign odontogenic tumour rather than a carcinoma.  • Dense fibrous stroma intimately intermingles with the epithelial cords. The cell morphology (blended peripheral & central cells, spindle change) together with the intimate epithelial–stromal intermingling and dense collagen background corroborate the desmoplastic subtype rather than other ameloblastoma patterns or mixed odontogenic tumours.  Summarising features that clinch the diagnosis  Pronounced, sclerotic/desmoplastic stroma pushing and compressing the epithelial component.  Small, thin cords and nests of odontogenic epithelium without the usual tall columnar palisading or stellate-reticulum-like centres of a conventional follicular pattern.  Reactive bone / osteoid deposition within the fibrous tissue (seen as large pale trabeculae with entrapped cells).  Lack of cytologic malignancy, supporting a benign ameloblastomatous lesion rather than an odontogenic carcinoma.  Together these features are diagnostic of a desmoplastic ameloblastoma, a recognised but comparatively uncommon variant in the spectrum of odontogenic tumours. |
| 15.11c | **Diagnosis**: Follicular and Plexiform Ameloblastoma with Granular Cells; **Key Microscopic Features**: The plexiform pattern comprises basaloid cells, which lack classic stellate reticulum but show basal cell palisading and reverse nuclear polarity. | Histopathological Diagnosis:  Ameloblastic Carcinoma  Key Microscopic Features Supporting the Diagnosis:  From the Low-Power Image (First Image):  Infiltrative Growth Pattern:  The tumor invades surrounding connective tissue irregularly, with no clear encapsulation, which is atypical for benign ameloblastoma and suggests malignancy.  Nests and Cords of Tumor Cells:  The architecture shows irregular islands and sheets of odontogenic epithelium resembling ameloblastoma but with more aggressive features.  Desmoplastic Stroma:  Dense fibrous stromal reaction is seen around the invading tumor islands, which is more commonly associated with malignant processes.  From the High-Power Image (Second Image):  Cytologic Atypia:  Noticeable nuclear pleomorphism, hyperchromatism, and increased nuclear-to-cytoplasmic ratio, all of which are features of cellular atypia suggestive of malignancy.  High Mitotic Activity:  Multiple mitotic figures are visible, which is not characteristic of benign ameloblastomas.  Peripheral Palisading and Reverse Polarity:  Some areas still show peripheral palisading of columnar cells with nuclei polarized away from the basement membrane—this is reminiscent of ameloblastoma, supporting the odontogenic origin.  Central Stellate Reticulum-like Areas:  There are focal areas within the tumor nests mimicking stellate reticulum, a common feature of ameloblastoma. | Histopathological diagnosis  ► Adenomatoid odontogenic tumor (AOT)  Key microscopic findings that point to AOT  Finding How it appears in the submitted slides Diagnostic significance  Encapsulated, well-circumscribed mass Low-power field shows the lesion demarcated by a fibrous rim and pushing rather than infiltrating adjacent connective tissue AOTs are usually surrounded by a fibrous capsule, helping distinguish them from infiltrative ameloblastomas  Sheets, whorls and nests of odontogenic epithelium Tumor is largely cellular, made up of basophilic epithelial cells arranged in solid nodules and interlacing cords Typical growth pattern of AOT  Numerous duct-like / tubular (pseudo-glandular) structures At both magnifications, many round or triangular spaces are lined by a single row of columnar cells; lumina contain pale eosinophilic material The hallmark feature that gives the tumor its “adenomatoid” appearance  Nuclear polarization (“reverse polarity”) of lining cells In high power, nuclei of columnar cells are consistently located away from the luminal surface Recapitulates ameloblastic differentiation and supports odontogenic origin  Hyaline / amyloid-like eosinophilic material and small calcific foci within lumina and stroma Central spaces and scattered stromal deposits show homogeneous pink material with occasional vacuoles (representing dissolved micro-calcifications) Snow-flake-type calcifications are classic for AOT and help separate it from other tubular odontogenic tumors  Minimal cytologic atypia and very rare mitoses Tumor cells are uniform, with round–oval nuclei and scant cytoplasm; pleomorphism is absent Confirms the lesion is benign rather than an ameloblastic carcinoma  These combined architectural (encapsulation, duct-like structures, whorled epithelial nodules) and cytologic (polarized columnar cells, uniform nuclei, hyaline/calcific luminal material) features are diagnostic of adenomatoid odontogenic tumor. |
| 15.12 | **Diagnosis**: Keratoameloblastoma; **Key Microscopic Features**: The photomicrograph reveals a plexiform pattern of odontogenic epithelium embedded in hyalinized connective tissue, with scattered microcysts containing parakeratin. | Histopathological Diagnosis:  Ameloblastoma (conventional/solid multicystic type)  Key Microscopic Features Justifying the Diagnosis:  Image 1 (Lower Magnification):  Islands and cords of odontogenic epithelium embedded in a fibrous stroma.  Peripheral palisading of columnar or cuboidal cells resembling ameloblast-like cells, with reverse nuclear polarity (nuclei away from basement membrane).  Central stellate reticulum-like areas, which mimic the enamel organ's star-shaped cells.  Cystic degeneration within epithelial islands, a common feature of solid/multicystic ameloblastoma.  The arrowheads point to epithelial islands, while arrows highlight the cystic changes or central stellate reticulum areas.  Image 2 (Higher Magnification):  Clear demonstration of peripheral columnar cells showing reverse polarization.  Loosely arranged central cells with a stellate appearance.  The tumor islands are well demarcated from the surrounding stroma, a hallmark of ameloblastoma.  Summary:  The combination of:  Palisaded ameloblast-like peripheral cells with reverse polarity,  Stellate reticulum-like central cells,  Tumor islands and cystic changes in a fibrous stroma,  strongly supports a diagnosis of conventional solid/multicystic ameloblastoma, a benign but locally aggressive odontogenic tumor. | Histopathological diagnosis – Ameloblastic fibro-odontoma (a “developing” complex odontoma)  Key microscopic features that lead to this diagnosis  # What is seen in the photomicrographs Significance  1. Dual-component tumour – an intimate admixture of:  • Epithelial strands / follicles (purple-blue)  • Cell-rich ectomesenchyme resembling dental papilla A defining requirement for the ameloblastic-fibro family of tumours  2. Ameloblastic follicles (best appreciated in the higher-power image)  • Peripheral tall/columnar cells in palisade with nuclei polarized away from the basement membrane  • Central stellate-reticulum–like cells Re-creates the enamel organ; rules out lesions that are purely mesenchymal or purely epithelial  3. Abundant hard-tissue formation (low-power image)  • Large, irregular eosinophilic masses of tubular dentin / dentinoid  • Adjacent empty clefts representing lost enamel matrix after decalcification The simultaneous presence of enamel- and dentin-like matrices distinguishes an -odontoma variant from a simple ameloblastic fibroma  4. Foci of rosette/“duct”-like epithelial structures with eosinophilic luminal material Frequently encountered in ameloblastic fibro-odontoma and indicate inductive change between epithelium and mesenchyme  5. Absence of significant cytologic atypia or infiltrative growth Supports a benign mixed odontogenic tumour rather than a malignant counterpart  The coexistence of an ameloblastoma-like epithelial component, a primitive ectomesenchymal stroma and abundant disorganised dental hard tissue (enamel + dentin) is diagnostic of ameloblastic fibro-odontoma, generally regarded as the histological stage that bridges an ameloblastic fibroma and a mature complex odontoma. |
| 15.13 | **Diagnosis**: Adenoid Ameloblastoma; **Key Microscopic Features**: The plexiform pattern is marked by dentinoid formation and pseudoducts, with areas resembling stellate reticulum and abundant dentinoid. | Based on the provided hematoxylin and eosin (H&E) stained histological images of the same lesion at low and high magnification, the single most likely histopathological diagnosis is:  Ameloblastoma (specifically, a follicular type)  Key Microscopic Features Supporting the Diagnosis:  Low Magnification (First Image):  Multicystic pattern: The lesion displays multiple cyst-like spaces and islands of odontogenic epithelium, a hallmark of ameloblastoma.  Fibrous stroma: The background stroma is fibrous and loosely collagenous, separating epithelial islands.  Epithelial islands: Numerous epithelial nests and follicles with variable sizes are observed throughout the stroma.  High Magnification (Second Image):  Peripheral palisading: The epithelial nests show a lining of columnar cells with palisaded nuclei at the periphery, which is characteristic of ameloblastoma.  Reverse nuclear polarity: The nuclei of the peripheral cells are oriented away from the basement membrane, a classic sign.  Stellate reticulum-like cells: The central portion of the epithelial nests contains loosely arranged angular cells resembling the stellate reticulum seen in the enamel organ.  Lack of significant atypia or mitoses, supporting a benign yet locally aggressive lesion. | Histopathologic diagnosis  Ameloblastic fibro-odontoma (AFO) – a mixed (epithelial + mesenchymal) odontogenic tumour that shows inductive production of both dentinoid and enamel matrix in association with an ameloblastic epithelial component.  Key microscopic features visible in the two H-E images  Feature What you can see in the pictures Diagnostic significance  1. Ameloblastic epithelial follicles/cords Numerous round and oval islands and inter-anastomosing strands of odontogenic epithelium; each island has a rim of tall columnar “ameloblast-like” cells with nuclei polarized away from the lumina, and a looser central zone of stellate-reticulum-like cells (best appreciated in the lower half of the high-power image). Confirms an ameloblastoma-like epithelial component, required for all “ameloblastic-” tumours.  2. Primitive ectomesenchymal (dental-papilla–like) stroma The background tissue between the epithelial islands is highly cellular, consisting of plump spindle-shaped fibroblasts in a myxoid to fibrous matrix. Places the lesion in the “mixed” group (both epithelium and mesenchyme are neoplastic).  3. Induced hard-tissue formation Irregular masses and trabeculae of intensely eosinophilic, homogeneous material with entrapped tubules/lacunae (dentinoid) and more delicate pale material representing enamel matrix; these pink masses are intimately fused to the peripheral epithelium (upper half of the high-power image and the large central pink islands in the low-power view). The simultaneous presence of dentin-like and enamel-like matrix distinguishes an ameloblastic fibro-odontoma (if only dentinoid were present it would favour ameloblastic fibrodentinoma).  4. Micro- and macro-cystic spaces Multiple round empty spaces lined by a thin rim of odontogenic epithelium (low-power image). A common architectural feature in AFO and other developing-odontoma lesions.  5. Bland cytology Both epithelial and mesenchymal cells show bland, uniform nuclei without atypia or mitotic figures. Supports a benign odontogenic tumour rather than an aggressive malignancy.  The combination of ameloblastoma-like epithelium, cell-rich dental-papilla stroma, and conspicuous formation of both dentin and enamel matrix is characteristic of ameloblastic fibro-odontoma and accounts for the final diagnosis. |
| 15.13b | **Diagnosis**: Adenoid Ameloblastoma; **Key Microscopic Features**: The pattern shows plexiform epithelial proliferation with pseudoducts and dentinoid; some areas contain ghost cells and epithelial morules with degenerated stroma. | Histopathological Diagnosis:  Ameloblastoma (Follicular Type)  Justification Based on Key Microscopic Features:  These hematoxylin and eosin-stained images show classic features diagnostic of follicular ameloblastoma, a benign but locally aggressive odontogenic tumor. Here's the breakdown by features seen:  1. Follicular Pattern:  Multiple islands or follicles of odontogenic epithelium within a fibrous connective tissue stroma.  Seen clearly in all three images—discrete round to ovoid epithelial nests dispersed throughout the lesion.  2. Peripheral Palisading of Columnar Cells:  The epithelial islands exhibit a layer of tall columnar basal cells with nuclear palisading (nuclei aligned peripherally), often showing reverse polarity (nuclei away from the basement membrane).  Most evident in the second and third images.  3. Stellate Reticulum-like Centers:  The central cells of the epithelial nests resemble the stellate reticulum of the enamel organ (loosely arranged, star-shaped cells).  This is a hallmark of the follicular variant and clearly present throughout the nests.  4. Cystic Degeneration:  Cystic spaces or degeneration within the epithelial islands, often seen in follicular ameloblastomas.  Present in all images as clear/empty spaces within epithelial nests.  5. Keratin Pearl Formation (in one area):  The second image shows a keratin pearl formation, which is uncommon in ameloblastoma but can rarely occur.  This might suggest squamous metaplasia within islands but does not alter the diagnosis.  6. Dense Fibrous Stroma:  The background stroma is dense and fibrous, typical of ameloblastomas.  There is also evidence of vascularity and mild inflammatory infiltrate. | Histopathological diagnosis  Adenomatoid odontogenic tumor (AOT) — the “adenomatoid” (gland-like) variant of ameloblastoma  Key microscopic features that point to AOT (seen across the three H-E fields)  Feature What you can see in the submitted slides Why it is diagnostic  Well-circumscribed/encapsulated lesion In the low-power overview the tumour mass is sharply demarcated from the surrounding fibrous tissue and muscle. AOTs are benign, usually surrounded by a thin capsule.  Numerous micro- & macro-cystic/“duct-like” spaces Round to oval empty lumina of widely varying size are scattered throughout the lesion; each is lined by a single row of cuboidal/columnar odontogenic epithelium with occasional nuclear polarization away from the lumen. The pseudo-glandular (adenomatoid) pattern is the histologic hallmark of AOT.  Rosette / whorled nests of tumour cells At intermediate power (arrow in slide 3) the epithelial cells swirl around small central spaces, creating rosettes. Adds to the “adenomatoid” architecture and helps distinguish AOT from other odontogenic tumours.  Eosinophilic hyaline material within ducts & rosettes Many lumina contain homogeneous pink material; some is partly calcified. This so-called “tumour droplet” or amyloid-like material is typical of AOT.  Foci of calcification (“snow-flake” calcifications) Concentric, lamellated eosinophilic masses and tiny basophilic specks are present in and around epithelial nests (prominent centrally in slide 2). Dystrophic or enameloid calcifications are found in ~75 % of AOTs and support the diagnosis.  Spindle-shaped / basaloid epithelial cells with scant cytologic atypia The cellular component shows uniform nuclei, delicate chromatin and inconspicuous nucleoli; mitoses are rare. Confirms the benign nature of the tumour.  The combination of a capsulated odontogenic lesion displaying abundant duct-like spaces, rosettes, hyaline luminal material and scattered calcifications is characteristic for adenomatoid odontogenic tumor and effectively excludes other entities such as conventional ameloblastoma, calcifying epithelial odontogenic tumor (CEOT) or glandular odontogenic cyst. |
| 15.15 | **Diagnosis**: Unicystic Ameloblastoma; **Key Microscopic Features**: A large cystic space is observed, with hyperchromatic basal cells, eosinophilic luminal cells, and a pattern of palisaded nuclei resembling “red-white-blue”. | Histopathological Diagnosis:  Adenomatoid Odontogenic Tumor (AOT)  Key Microscopic Features Supporting the Diagnosis:  The diagnosis of Adenomatoid Odontogenic Tumor (AOT) is supported by the following histopathological features observed in both low and high magnification hematoxylin and eosin (H&E) stained images:  Low-Power View (First Image):  Well-circumscribed lesion with a lobulated architecture.  Numerous duct-like (tubular/cribriform) structures, often arranged in a rosette or gland-like pattern.  Areas of dense fibrous stroma surrounding the epithelial components.  No significant cellular atypia or mitotic activity is visible, supporting a benign nature.  High-Power View (Second Image):  Duct-like structures lined by a single layer of columnar to cuboidal epithelial cells with nuclei polarized away from the luminal surface, mimicking glandular formations.  Spindle-shaped or polygonal epithelial cells forming sheets and whorled nests.  Some eosinophilic material within the duct-like lumina, a common finding in AOT.  Minimal inflammatory infiltrate and absence of aggressive features (like invasion or pleomorphism). | Histopathological diagnosis  Unicystic ameloblastoma – luminal + intraluminal (plexiform) variant  Microscopic features in the two fields that point to this diagnosis  Field Key observations Diagnostic significance  Low-power overview (1st image) • A single, well-circumscribed cystic cavity occupies most of the specimen.  • The cyst wall is a collagenous fibrous capsule; its lumen is partly empty and partly filled by multiple papillary and nodular intraluminal proliferations of epithelium, one of which forms a large dome-shaped mass.  • The epithelial projections are long, thin, anastomosing cords and sheets – the classic plexiform pattern. A unilocular cyst with an ameloblastomatous epithelial lining and intraluminal tumour nodules is typical of a unicystic ameloblastoma rather than a solid/multicystic (“classical”) ameloblastoma or other odontogenic cysts/tumours.  High-power view (2nd image) • The intraluminal strands are composed of palisaded cuboidal-to-columnar basal cells with hyperchromatic, polarized nuclei (away from the basement membrane) and occasional subnuclear vacuoles.  • Central cells in the strands are loosely arranged and stellate-reticulum-like; some areas show small micro-cysts between cords.  • No keratinisation, ghost cells, mucous cells or calcifying amyloid deposits are seen.  • Fibrous capsule shows only a thin band of inflammation, with no obvious mural invasion in the planes shown. The combination of peripheral palisading, nuclear reverse polarity and central stellate-reticulum-like cells is diagnostic of ameloblastomatous epithelium. The purely luminal/intraluminal growth and lack of mural infiltration fit the luminal + intraluminal subtype of unicystic ameloblastoma. Absence of features typical of OKC, COC, AOT or CEOT helps rule out those entities.  Why other odontogenic lesions are unlikely  Odontogenic keratocyst (OKC): would show a thin, corrugated parakeratinised lining without intraluminal plexiform proliferation.  Adenomatoid odontogenic tumour (AOT): usually has whorled, duct-like structures with eosinophilic hyaline and frequent calcifications; the lining is not that of a cyst with plexiform cords.  Calcifying odontogenic cyst / tumour: requires ghost cells and calcifications, absent here.  Taken together, the cystic architecture plus plexiform ameloblastomatous epithelium confined to the lumen are diagnostic of unicystic ameloblastoma, plexiform (luminal + intraluminal) variant. |
| 15.16 | **Diagnosis**: Plexiform Unicystic/Intraluminal Ameloblastoma; **Key Microscopic Features**: The cystic structure displays plexiform luminal proliferation, while thin epithelial lining reveals eosinophilic cells and hyperchromatic basal cells. | Histopathological Analysis of the Provided Odontogenic Tumor Images  Most Likely Histopathological Diagnosis:  Adenomatoid Odontogenic Tumor (AOT)  Key Microscopic Features Supporting the Diagnosis:  1. General Architecture (First Image - Low Magnification):  Well-circumscribed lesion: The tumor appears encapsulated, with a lobulated growth pattern.  Cystic and solid areas: Suggests a mixed solid-cystic nature typical of AOT.  Fibrous capsule: Seen demarcating the lesion from the surrounding stroma.  2. Epithelial Pattern (Second Image - Intermediate Magnification):  Duct-like (tubular/cribriform) structures: A hallmark of AOT, these are lined by a single layer of columnar or cuboidal epithelial cells with polarized nuclei.  Rosette-like formations and whorled patterns: These are diagnostic for AOT.  Eosinophilic, amorphous material: Likely representing tumor droplets or hyaline material within duct-like spaces.  3. Cellular Details (Third Image - High Magnification):  Columnar or cuboidal epithelial cells: With nuclei polarized away from the central lumen, again forming duct-like and rosette structures.  Lack of significant cytologic atypia or mitotic activity: Consistent with the benign nature of AOT.  Stroma: Loose, fibrous connective tissue without marked inflammation or atypia. | Histopathological diagnosis  Adenomatoid odontogenic tumor (AOT).  Key microscopic features supporting the diagnosis  Microscopic finding How it appears in the supplied fields Diagnostic significance  Well-circumscribed / thinly encapsulated lesion Low-power view (first image) shows a lobulated epithelial mass sharply demarcated from the surrounding fibrous connective tissue. AOTs are benign, non-infiltrative and usually surrounded by a delicate capsule.  Lobules, cords and sheets of odontogenic epithelium Throughout all images, epithelial cells form solid nests, intertwining cords and trabeculae. Odontogenic origin of the tumor.  Characteristic “duct-like” / tubular structures Second and especially the third image show round to oval lumina lined by a single row of tall columnar cells; nuclei are polarised away from the lumen (reverse polarity). The adenomatoid (“gland-like”) pattern is the hallmark of AOT and distinguishes it from ameloblastoma.  Rosette- or whorl-shaped epithelial arrangements In the mid-power field (second image) concentric whorls of spindle-shaped epithelial cells are present. Common architectural motif in AOT.  Eosinophilic/hyaline material within lumina; focal calcifications Lumina in the third image contain pale eosinophilic deposits; scattered foci of basophilic material are present in other lobules (best appreciated on higher power). Luminal hyaline secretions and dystrophic calcifications are typical ancillary features of AOT.  Minimal cytologic atypia and very low mitotic activity Tumour cells are uniform with oval nuclei and inconspicuous nucleoli; mitoses are essentially absent. Confirms the benign nature of the lesion.  The combination of a circumscribed lesion composed of odontogenic epithelium forming duct-like/tubular structures with nuclear reverse polarity, rosettes, luminal eosinophilic material and occasional calcifications is pathognomonic for an adenomatoid odontogenic tumor. |
| 15.18 | **Diagnosis**: Peripheral Ameloblastoma, Follicular Pattern, Classic Type; **Key Microscopic Features**: Follicular epithelial proliferation continues with surface epithelium, showing palisading of basal cells and areas resembling stellate reticulum. | Based on the provided hematoxylin and eosin (H&E) stained histological images at low and high magnification, the single most likely histopathological diagnosis is:  Adenomatoid Odontogenic Tumor (AOT)  Key Microscopic Features Supporting the Diagnosis:  1. Overall Architecture (Low Magnification Image):  Well-circumscribed lesion: The tumor appears encapsulated, with a lobular architecture.  Predominantly epithelial composition: Solid nodules and duct-like or rosette-like structures are visible within the fibrous stroma.  Peripheral palisading of columnar cells: Often noted, though less prominent here due to resolution.  2. High Magnification Features:  Duct-like structures (pseudoducts): These are characteristic and seen clearly—lined by cuboidal to columnar epithelial cells arranged in circular or rosette-like formations with central spaces.  Eosinophilic material within the lumens: Frequently seen in AOT, possibly representing amyloid-like or hyaline material.  Spindle-shaped epithelial cells: Present in between duct-like spaces, forming sheets and whorls.  Absence of mitotic activity and atypia: Supports a benign nature. | Single most likely histopathological diagnosis  → Adenomatoid Odontogenic Tumor (AOT)  Key microscopic clues visible in the two fields  Low-power field High-power field Diagnostic value  Sharply circumscribed, sub-mucosal nodule surrounded by a thin fibrous capsule Cells are cytologically bland with very little pleomorphism or mitotic activity Benign, encapsulated odontogenic tumour  Numerous rounded and lobular epithelial nests and whorled/rosette-like structures Each nest shows a central lumen or pseudolumen; many are filled with pale eosinophilic material “Adenomatoid” (gland-like / duct-like) growth pattern is the hallmark of AOT  Frequent duct-like or tubular spaces on low power Lining cells are tall cuboidal-to-columnar with nuclei polarized away from the lumen (“reverse polarity”) Classic appearance of AOT ducts  Delicate, sparsely cellular fibrous stroma; no infiltrative edges Occasional spindle-shaped epithelial cells merge into the whorls; scattered droplets of hyaline material that may calcify (not prominent in this section) Supports AOT and helps exclude ameloblastoma or CEOT  Why these findings point to AOT (and not other odontogenic tumours)  Ameloblastoma lacks true duct-like lumina and shows central stellate-reticulum-type cells rather than rosettes with reverse polarity.  Calcifying epithelial odontogenic tumour (Pindborg) shows sheets of polyhedral cells, intercellular bridges, amyloid and Liesegang-ring calcifications—not present here.  Odontogenic keratocyst, squamous odontogenic tumour, etc. do not display the distinctive whorled, duct-forming epithelium seen in these slides.  Taken together, the encapsulation, whorled epithelial pattern, numerous duct-like structures with polarized lining cells, and lack of cytologic atypia are diagnostic of adenomatoid odontogenic tumor. |
| 15.19 | **Diagnosis**: Peripheral Ameloblastoma, Follicular Pattern, Acanthomatous Type; **Key Microscopic Features**: The follicular structure lacks surface epithelial connection, with palisading basal cells and central acanthomatous histology. | Based on the histopathological analysis of the provided hematoxylin and eosin (H&E) stained sections of the odontogenic tumor (low and high magnification), the single most likely diagnosis is:  Adenomatoid Odontogenic Tumor (AOT)  Key Microscopic Features Supporting the Diagnosis:  1. Duct-like (Adenomatoid) Structures:  The hallmark feature is the presence of duct-like structures or tubular rosette-like formations, which are visible in both low- and high-power views. These structures are lined by a single layer of columnar or cuboidal epithelial cells arranged in a circular pattern, mimicking glandular architecture.  2. Epithelial Cell Proliferation:  The tumor is composed of spindle-shaped to polygonal epithelial cells forming solid nests, sheets, and whorls, which are characteristic of AOT.  3. Hyalinized/Stromal Component:  There is a prominent fibrous connective tissue stroma that appears relatively loosely arranged and contains areas of eosinophilic hyaline material, which is often observed in AOT.  4. Minimal Atypia and Low Mitotic Activity:  The epithelial cells exhibit bland nuclear features, with no significant pleomorphism or mitotic activity, consistent with the benign nature of AOT.  5. Well-circumscribed Nature:  At low magnification, the lesion appears well-demarcated from the surrounding tissue, often encapsulated or circumscribed—another supportive feature for AOT.  Differential Diagnosis Considerations (Ruled Out Based on Histology):  Ameloblastoma: Lacks duct-like structures; has peripheral palisading and reverse polarity.  Calcifying Epithelial Odontogenic Tumor (Pindborg Tumor): Shows amyloid and Liesegang ring calcifications, which are absent here.  Odontogenic Keratocyst: Cystic and lacks epithelial proliferation patterns seen here.  Dentigerous Cyst with AOT-like change: Might show some similarities, but extensive duct-like proliferation favors a pure AOT. | Histopathological diagnosis  Desmoplastic ameloblastoma (desmoplastic variant of solid/multicystic ameloblastoma)  Key microscopic features supporting this diagnosis  Field Observations How the feature fits desmoplastic ameloblastoma  Low-power architecture • A single, well-demarcated but un-encapsulated lesion seated beneath intact oral mucosa.  • The section is dominated by dense, eosinophilic, collagen-rich stroma; the epithelial component is relatively sparse and appears as thin cords and small nests that are widely separated by this stroma. Marked stromal desmoplasia with scant odontogenic epithelium is the defining hallmark of the desmoplastic variant.  Distribution & shape of epithelial islands • Islands are irregular, compressed, and ribbon- or fish-net-shaped rather than large round follicles.  • They vary in size and often merge to form anastomosing strands. The desmoplastic variant shows distorted, squeezed epithelial nests because the fibrous stroma exerts pressure on them.  Peripheral epithelial cells • In many islands a rim of cuboidal/low-columnar cells is discernible; palisading and reverse nuclear polarity are present but much less dramatic than in classic follicular ameloblastoma. Blunted or incomplete ameloblastic palisading is typical in this variant.  Central epithelial cells • Centers of the nests contain loosely arranged, stellate or spindle-shaped cells; occasional squamoid change is evident. A stellate-reticulum-like centre is a key ameloblastic feature; squamoid or clear-cell change is common in the desmoplastic type.  Stroma • Collagen bundles are thick, hypocellular and often swirl around the epithelial elements (“keloid-like” desmoplasia).  • Entrapped delicate capillaries are scattered throughout. The pronounced desmoplastic reaction is what gives the tumour its name and separates it from other ameloblastoma patterns.  Additional findings • Tiny microcystic / duct-like spaces can be seen focally within some epithelial strands; no enamel-matrix globules or distinct calcified Liesegang rings are present. Supports ameloblastic origin and helps rule out adenomatoid odontogenic tumour or calcifying epithelial odontogenic tumour.  Taken together, the densely collagenised stroma that dominates the section and the compressed, irregular islands of odontogenic epithelium with attenuated ameloblastic features are characteristic for desmoplastic ameloblastoma and distinguish it from the classic follicular / plexiform patterns, adenomatoid odontogenic tumour, or other odontogenic lesions. |
| 15.21 | **Diagnosis**: Adenomatoid Odontogenic Tumor; **Key Microscopic Features**: The tumor is surrounded by a thick capsule, containing solid, nodular, and reticular areas. It has mucinous stroma and cells forming rosette-like structures with enameloid material. | Based on the provided hematoxylin and eosin (H&E) stained histopathological images of an odontogenic tumor at various magnifications, the single most likely diagnosis is:  Ameloblastoma  Key Microscopic Features Justifying the Diagnosis:  Follicular and Plexiform Patterns:  Seen in the low to intermediate magnification images.  Islands and cords of odontogenic epithelium resembling the enamel organ are evident.  Follicles exhibit peripheral palisading of columnar cells with reverse nuclear polarity and central stellate reticulum-like cells.  Peripheral Palisading with Reverse Polarity:  The peripheral layer of columnar cells shows nuclei aligned away from the basement membrane—a hallmark of ameloblastoma.  Stellate Reticulum-like Central Cells:  The central regions of the epithelial nests resemble the stellate reticulum of the enamel organ.  Loose Fibrous Stroma:  The tumor islands are embedded in a loosely arranged, vascular stroma, with minimal inflammatory infiltrate, supporting a benign but locally aggressive neoplasm.  No Evidence of Hard Tissue Formation:  Absence of dentin or enamel matrix helps exclude odontoma or adenomatoid odontogenic tumor.  Lack of Clear Cytological Atypia or High Mitotic Activity:  Supports a diagnosis of a conventional ameloblastoma rather than a malignant variant. | Histopathological diagnosis – Adenomatoid Odontogenic Tumor (AOT)  Key microscopic features supporting this diagnosis  Field-of-view What is seen Why it points to AOT  Low-power overview (Image 1) Circumscribed, lobulated epithelial lesion sharply demarcated from surrounding fibrous connective tissue by a fibrous capsule. AOT is typically well-encapsulated and lobular, unlike the infiltrative growth of conventional ameloblastoma.  Medium power (Image 2) Nodules and whorls of uniform spindle-to-polygonal odontogenic epithelial cells; numerous duct-like / tubular spaces with a rim of columnar cells whose nuclei are polarized away from the lumen; lumina filled with smooth, eosinophilic “tumor droplets.” Small round foci of calcification are beginning inside some lumina. The combination of duct-like structures, tumor droplets, and calcifying foci is essentially pathognomonic for an adenomatoid odontogenic tumor.  Higher power (Image 3) Classic rosette- and duct-shaped arrangements: concentric rings of epithelial cells around an eosinophilic central core, sometimes with abortive dentinoid or psammomatoid calcifications. Cells are bland, with scant mitotic activity. Rosettes/ducts with polarized columnar cells distinguish AOT from other odontogenic tumors (e.g., CEOT lacks true ducts and shows polyhedral cells with intercellular bridges).  Highest power (Image 4) Delicate, lightly collagenized stroma with sparse spindle cells and scattered micro-calcifications; no significant cytologic atypia or mitoses in epithelial component. The innocuous cytology and paucicellular stroma fit a benign AOT rather than an ameloblastic carcinoma or sarcomatous lesion.  Hallmark triad seen across the sections  Encapsulation and lobulated architecture  Duct-like / rosette formations of odontogenic epithelium with polarized nuclei  Eosinophilic intraluminal material (“tumor droplets”) ± focal calcification |
| 15.22 | **Diagnosis**: Adenomatoid Odontogenic Tumor; **Key Microscopic Features**: The thick capsule encloses nodular, whorled, and reticular areas, with duct-like structures and clear cells forming rosettes and depositing enameloid. | Histopathological Diagnosis:  Adenomatoid Odontogenic Tumor (AOT)  Key Microscopic Features Justifying the Diagnosis:  Encapsulated Lesion (Low Magnification):  The lesion is well-circumscribed and surrounded by a fibrous capsule, as seen in the lowest magnification image.  This feature helps distinguish AOT from more aggressive odontogenic tumors.  Multicystic and Duct-like Structures:  Numerous duct-like or gland-like spaces lined by a single layer of cuboidal to columnar epithelial cells with nuclei polarized away from the lumen.  These structures are characteristic and highly suggestive of AOT.  Rosette-like Arrangements and Whorls:  The epithelial cells frequently form rosettes, nodules, and whorled configurations.  These arrangements are distinct for AOT and are visible in the intermediate and high magnification images.  Eosinophilic (Hyaline-like) Material:  Presence of amorphous eosinophilic material within duct-like spaces and between epithelial islands—often misinterpreted as dentinoid or amyloid-like material.  This adds to the uniqueness of AOT’s histological appearance.  Scattered Calcifications:  Fine calcifications (though not always abundant) are sometimes seen within the tumor, another feature seen in AOT.  May appear as dystrophic or globular calcifications.  Minimal Cytological Atypia and Mitoses:  Tumor cells appear bland with no significant pleomorphism or mitotic activity, supporting its benign nature. | Histopathological diagnosis  Adenomatoid Odontogenic Tumor (AOT)  Key microscopic features supporting this diagnosis  Field view What is seen Why it points to AOT  Low-power (first image) • A single, well-circumscribed intrabony mass surrounded by a thick fibrous capsule.  • Tumor arranged in many rounded lobules separated by delicate septa; lobules contain numerous tiny cyst-like and tubular spaces. AOTs are benign, encapsulated tumors with a lobular architecture and prominent pseudocystic change.  Intermediate power (second image) • Solid nests and whorls of odontogenic epithelium set in a loosely collagenous, vascular stroma.  • Frequent slit-like or oval “gland/duct-like” spaces and cribriform patterns; some lumina contain pale eosinophilic material. The combination of solid epithelial sheets with conspicuous duct- or rosette-like structures is characteristic and gives the tumor its “adenomatoid” appearance.  High power (third & fourth images) • The duct/tubular structures are lined by a single layer of cuboidal-to-columnar cells whose nuclei are polarized away from the lumen (reverse polarity).  • Cells are uniform, with scant cytoplasm, minimal pleomorphism, and rare mitoses.  • Occasional eosinophilic amorphous droplets or empty round spaces representing decalcified mineral foci. Nuclear polarization around duct-like lumina, bland cytology, and occasional calcific foci are classic microscopic hallmarks of AOT and help distinguish it from ameloblastoma or CEOT.  Taken together, the encapsulation, lobular growth, abundant duct/rosette formations with polarized columnar lining cells, bland cytology and focal calcific/amorphous deposits establish the lesion as an Adenomatoid Odontogenic Tumor. |
| 15.24 | Diagnosis: Calcifying epithelial odontogenic tumor; Key Microscopic Features: Strands and interlacing cords of tumor cells with abundant amyloid and focal calcifications. Tumor cells exhibit an interlacing pattern of growth with a possible pseudocribriform arrangement. Cells are large, polygonal, with abundant eosinophilic cytoplasm, nuclear pleomorphism, and nuclear pseudoinclusions. | Based on the hematoxylin and eosin (H&E) stained histopathological images provided at low, medium, and high magnifications, the single most likely diagnosis is:  Ameloblastoma  Key Microscopic Features Justifying the Diagnosis:  1. Follicular Pattern of Tumor Islands (Low Magnification - Image 1):  Multiple well-demarcated epithelial islands or follicles are seen within a fibrous stroma.  These islands show peripheral palisading of columnar or cuboidal cells with hyperchromatic, reverse-polarized nuclei (nuclei located away from the basement membrane).  Central areas often resemble the stellate reticulum of the enamel organ.  2. Presence of Cystic Degeneration (Medium Magnification - Image 2):  Central cystic spaces or loosely arranged cells in the follicular islands, simulating early cystic change.  These features are common in ameloblastoma and contribute to its locally aggressive nature.  3. Peripheral Palisading and Reverse Polarity (High Magnification - Image 3):  The periphery of the epithelial nests clearly shows palisaded columnar cells.  The nuclei of these cells exhibit reverse polarization, a hallmark of ameloblastic differentiation.  The center of the follicles contains loosely arranged, angular cells with a stellate reticulum-like appearance.  Conclusion:  The histological architecture—particularly the follicular arrangement, reverse nuclear polarity, and stellate reticulum-like central areas—is classic for follicular ameloblastoma, the most common subtype of ameloblastoma. | Histopathological diagnosis  Clear-cell odontogenic carcinoma (CCOC) – malignant clear-cell tumour of odontogenic epithelium.  Key microscopic features that support this diagnosis  Field Features visible in the submitted H-E sections Diagnostic significance  Architecture Lobules, nests and cords of epithelium sharply delineated by broad bands of dense, hyalinised collagenous stroma (first image, low-power). CCOC typically shows an infiltrative lobular growth pattern separated by sclerotic or hyalinised fibrous septa.  Cell populations Predominant cells have abundant optically clear / vacuolated cytoplasm with distinct borders and centrally-placed round nuclei (all magnifications, especially image 3). Cytoplasmic clearing is due to glycogen (PAS-positive, diastase-labile) and is the hallmark of clear-cell odontogenic tumours.  Intermixed smaller basaloid / ameloblast-like cells with darker, slightly palisaded nuclei are seen at the periphery of some nests (image 3). A biphasic pattern (clear cells + basaloid cells) is characteristic of the common “biphasic” variant of CCOC.  Stroma / additional material Occasional foci of coarse dystrophic calcification within epithelial islands (black–purple deposits in images 1 & 2). Focal calcification is reported in CCOC and helps to exclude purely myxoid or ameloblastic lesions.  Cytology Nuclei show mild-to-moderate pleomorphism and occasional mitotic figures (high-power, image 3); no ghost cells or extensive amyloid seen. Nuclear atypia with clear cells supports carcinoma rather than benign clear-cell odontogenic tumour or clear-cell ameloblastoma.  Together, the infiltrative lobular pattern, hyalinised septa, glycogen-rich clear cells admixed with basaloid cells, and focal calcifications most closely match clear-cell odontogenic carcinoma among the odontogenic tumour spectrum. |
| 15.24b | Diagnosis: Calcifying epithelial odontogenic tumor; Key Microscopic Features: Islands of epithelial cells surrounded by amyloid. Islands of clear cells present. Tumor cells display moderate nuclear pleomorphism with nuclear pseudoinclusions. Clear cells and eosinophilic cytoplasm within fibrous stroma. | Based on the histopathological examination of the provided hematoxylin and eosin (H&E) stained images of an odontogenic tumor, the most likely single diagnosis is:  Ameloblastoma  Key Microscopic Features Supporting the Diagnosis:  Follicular and Plexiform Patterns (Image 1 & 2):  Islands and cords of odontogenic epithelium.  Peripheral palisading of columnar ameloblast-like cells with reverse nuclear polarization.  Central stellate reticulum-like cells within the epithelial islands.  Cystic Degeneration and Loose Stroma (Image 3):  Evidence of cystic change and acellular or loosely arranged stroma.  Appearance of apoptotic/necrotic cells in some cystic areas.  Desmoplastic Features (Image 4):  Compressed islands of odontogenic epithelium within a densely collagenized (desmoplastic) stroma.  Scattered tumor islands suggestive of the desmoplastic variant of ameloblastoma. | Histopathological diagnosis  Calcifying epithelial odontogenic tumor (CEOT, “Pindborg tumor”)  Key microscopic features shown in the photomicrographs  Image Salient findings How the finding supports CEOT  1 (low-power overview) Multiple irregular islands and cords of epithelium separated by a collagen-rich stroma. CEOT is an unencapsulated epithelial odontogenic neoplasm that grows in nests/strands within a fibrous connective-tissue background.  2 (intermediate power) Sheets of uniform polygonal / polyhedral cells with distinct cell borders, moderate eosinophilic granular cytoplasm and intercellular bridges; nuclei sometimes pleomorphic but mitoses rare. The characteristic tumor cell phenotype of CEOT differs from the tall peripheral columnar cells of ameloblastoma or the spindle cells of AOT.  3 (high power) Abundant extracellular amorphous, pale eosinophilic material; some areas show basophilic, concentric lamellations (“Liesegang rings”). The eosinophilic material represents the tumor’s pathognomonic amyloid-like protein (Congo-red positive, apple-green birefringence), which frequently undergoes dystrophic calcification forming Liesegang rings—virtually diagnostic of CEOT.  4 (high power stroma) Tumor islands scattered in a markedly desmoplastic fibrous stroma; small whorled nests of epithelial cells surrounded by dense collagen. The dense stroma and scattered epithelial nests are typical for intra-osseous CEOT and help distinguish it from encapsulated lesions such as AOT.  Composite pattern recognition  Polyhedral odontogenic epithelial cells  Intercellular bridges and occasional nuclear pleomorphism  Extracellular amyloid-like material that calcifies in concentric (Liesegang) rings  Desmoplastic connective-tissue stroma without a true capsule  The simultaneous presence of all of the above features in one lesion is essentially diagnostic of a calcifying epithelial odontogenic tumor. |
| 15.25 | Diagnosis: Langerhans cell–rich noncalcifying epithelial odontogenic tumor; Key Microscopic Features: Delicate islands and strands of epithelium within abundant amyloid. Epithelial cells have eosinophilic cytoplasm and slightly pleomorphic nuclei surrounded by amyloid. | Based on the provided hematoxylin and eosin (H&E) stained histological images at low and high magnification, the single most likely histopathological diagnosis is:  Ameloblastic Carcinoma  Key Microscopic Features Supporting the Diagnosis:  Architectural Features (Low Magnification):  The tumor exhibits a nodular growth pattern with islands and nests of odontogenic epithelium.  Peripheral palisading of columnar or cuboidal cells with reverse nuclear polarity is identifiable in many nests, reminiscent of ameloblastoma.  However, the growth pattern is more disorganized, with areas showing infiltrative borders.  Cytological Atypia (High Magnification):  There is marked nuclear pleomorphism, hyperchromasia, and increased nuclear-to-cytoplasmic ratio.  Abnormal mitotic figures are visible, indicative of malignancy.  Cellular dyscohesion, necrosis, and prominent nucleoli may also be present, differentiating it from benign ameloblastoma.  Other Supporting Features:  Unlike conventional ameloblastoma, this lesion demonstrates cytologic malignancy, which defines ameloblastic carcinoma.  There may be stromal desmoplasia and inflammatory infiltrates.  Differential Diagnosis Considerations:  Conventional Ameloblastoma: Lacks cytologic atypia and mitoses.  Squamous Odontogenic Tumor: Lacks ameloblast-like peripheral palisading and reverse polarity.  Primary Intraosseous Carcinoma: Generally lacks features of odontogenic epithelium (e.g., peripheral palisading). | Histopathological diagnosis  Calcifying epithelial odontogenic tumor (CEOT, “Pindborg tumour”)  Key microscopic features in the two fields that point to CEOT  Image What you see Why it matters  Low-power field (first picture) • Tumour arranged in lobules separated by delicate, collagen-rich septa.  • Each lobule is packed with pale, eosinophilic, amorphous material that almost fills the spaces between the cells.  • No cystic component or peripheral palisading typical of an ameloblastoma. Lobulated growth pattern of odontogenic epithelium with conspicuous extracellular deposits is classic for CEOT.  High-power field (second picture) • Sheets/cords of polygonal odontogenic epithelial cells with distinct cell borders and occasional intercellular bridges.  • Cells show mildly pleomorphic, hyperchromatic nuclei and abundant granular/clear cytoplasm.  • Extensive deposits of homogeneous eosinophilic amyloid-like material lying extracellularly; some foci beginning to calcify (early Liesegang-ring formation).  • Scattered red blood cells in the loose stroma (common but non-specific finding). 1. The polyhedral epithelial cells with intercellular bridges confirm epithelial—not mesenchymal—origin.  2. The large masses of extracellular, glassy pink material are the tumour-produced amyloid (congo-red-positive in practice).  3. Early calcification within this amyloid (future Liesegang rings) is virtually pathognomonic for CEOT and helps separate it from ghost-cell-containing lesions or clear-cell tumours.  Putting these together:  A lobulated epithelial odontogenic tumour whose cells manufacture abundant amyloid-like, eventually calcifying material is the defining histology of a Calcifying Epithelial Odontogenic Tumor. |
| 15.27 | Diagnosis: Squamous odontogenic tumor; Key Microscopic Features: Islands of squamous epithelium within fibrous stroma. Irregularly shaped islands of squamous cells within delicate fibrous stroma; one resembling a fish. Peripheral cells are low cuboidal or squamous, lacking palisading or reverse polarization. Dyskeratotic cells and dyscohesion present. Epithelial islands may contain brightly eosinophilic hyaline material resembling Rushton bodies or enameloid. | Histopathological Diagnosis:  Adenomatoid Odontogenic Tumor (AOT)  Key Microscopic Features Justifying the Diagnosis:  Duct-like Structures and Rosette Patterns:  Seen clearly in all magnifications, particularly in the 2nd and 3rd images, the lesion contains numerous round to oval spaces resembling ducts or gland-like structures.  These spaces are lined by a single layer of cuboidal to columnar epithelial cells with nuclei polarized away from the lumen (reverse polarity), giving a rosette or ductal appearance, which is a hallmark of AOT.  Cellular Architecture:  The epithelial component forms sheets, nests, and whorled patterns within a fibrous stroma.  The epithelial cells are uniform, basaloid, and have minimal atypia.  Eosinophilic Amorphous Material:  There are foci of eosinophilic, hyaline-like material, sometimes found within duct-like spaces or in the surrounding stroma, which is common in AOT and represents amyloid-like material or tumor-related secretions.  Calcifications:  Though not prominent in the uploaded images, small foci of calcifications may be seen in AOT (not essential but supportive when present).  Encapsulated, Benign Nature:  While a capsule is not clearly visible in these sections, AOTs are well-circumscribed and typically show a non-invasive growth pattern, supportive of a benign diagnosis.  Vascular Structures:  In the highest magnification image (4th image), capillary-sized vessels are present within the epithelial islands, sometimes centrally, adding to the complexity of the ductal structures. | Histopathological diagnosis  Desmoplastic ameloblastoma  ( desmoplastic variant of conventional ameloblastoma )  Key microscopic features visible in the photomicrographs  Finding How it looks in the submitted pictures Diagnostic significance  Intensely collagenised, “desmoplastic” stroma The background is pale-pink, densely fibrous and almost acellular, separating the epithelial elements (all four images). Hall-mark feature that gives the tumour its name and distinguishes it from the more common follicular / plexiform patterns.  Irregular, compressed islands, strands and cords of odontogenic epithelium Islands are elongated, kite-, fish- or comma-shaped rather than round (Images 1–3). They often look squeezed by the surrounding collagen. Characteristic growth pattern of desmoplastic ameloblastoma.  Attenuated or cuboidal peripheral cells without prominent palisading or reverse polarity The rim of the epithelial nests shows only 1–2 layers of flattened / low-columnar cells; no tall columnar “ameloblast-like” lining is seen (Images 2–4). Helps to separate this variant from ordinary follicular ameloblastoma.  Central cells with squamoid / spindle / stellate-reticulum-like change Centres of many islands are filled with polyhedral cells or loosely arranged spindle cells (Images 2–3). Fits the ameloblastoma family rather than duct-forming tumours such as AOT.  Multiple microcysts, some containing proteinaceous material or extravasated erythrocytes Small lumina or cystic spaces are present; one contains a cluster of red blood cells (Image 4). Microcyst formation is very common in the desmoplastic variant.  Occasional foci of eosinophilic coagulum / dystrophic calcification (not marked here but hinted by pale globules in Image 3) Pale eosinophilic droplets inside some nests. A supportive – though not obligatory – feature seen in many cases.  Taken together, the combination of a densely fibrotic (desmoplastic) stroma and the distinctive compressed, non-palisaded epithelial islands is diagnostic of desmoplastic ameloblastoma. |
| 15.28 | Diagnosis: Squamous odontogenic tumor; Key Microscopic Features: Irregularly shaped islands of squamous cells within fibrous stroma. Epithelial islands contain dyskeratotic cells, and peripheral cells are squamous and flattened. Sebaceous and mucous cells may be present. | Based on the provided hematoxylin and eosin (H&E) stained histological images of the same lesion at various magnifications, the single most likely histopathological diagnosis is:  Ameloblastoma (specifically, the follicular type)  Key Microscopic Features Supporting This Diagnosis:  1. Follicular Pattern (First Image - Low Magnification)  The tumor exhibits multiple discrete islands or follicles of odontogenic epithelium in a fibrous stroma.  These islands are well-demarcated and have a lobulated architecture, a classic hallmark of follicular ameloblastoma.  2. Peripheral Palisading (Second Image - Intermediate Magnification)  The epithelial islands show peripheral columnar or cuboidal cells arranged in palisading, resembling ameloblast-like cells.  The nuclei are polarized away from the basement membrane (reversed nuclear polarity), another key feature.  3. Central Stellate Reticulum-like Cells (Third Image - High Magnification)  The centers of the epithelial follicles show loosely arranged stellate reticulum-like cells, mimicking the enamel organ.  There is a loosely reticulated, pale-staining central area, which is a hallmark of ameloblastic differentiation.  Summary Diagnosis:  The combination of follicular architecture, peripheral palisading with reverse polarity, and stellate reticulum-like centers in epithelial nests within a fibrous connective tissue stroma is characteristic of follicular ameloblastoma. | Histopathological diagnosis  Adenomatoid Odontogenic Tumor (AOT) — follicular/“intra-osseous” type.  Key microscopic features supporting the diagnosis  Image Features seen Diagnostic significance  Low-power overview (1st image) Well-circumscribed, lobulated mass composed of innumerable epithelial nests, cords and sheets separated by a delicate fibrous capsule/septa. Within many islands are oval to slit-like “spaces,” giving the field a lace- or honey-comb appearance. AOT is usually encapsulated and shows a multinodular architecture rather than the infiltrative pattern of an ameloblastoma.  Intermediate magnification (2nd image) Islands made of uniform spindle-to-polygonal odontogenic epithelial cells arranged in whorls/rosettes. Peripheral cells are cuboidal to low columnar; nuclei show mild polarization but lack the tall, palisaded appearance of ameloblastoma. Central lumina often contain lightly eosinophilic hyaline material. Rosette- and duct-like structures formed by odontogenic epithelium are the histologic hallmark of AOT and account for the “adenomatoid” descriptor.  High magnification (3rd image) Classic “duct-like” / tubular spaces: a single layer of cuboidal–columnar cells surrounds a round/oval lumen that contains homogeneous eosinophilic material and “snow-flake” calcifications (bright basophilic to clear foci). The surrounding stroma is fibro-collagenous and uninflamed. Presence of duct-like structures with luminal hyaline material and dystrophic or enamel-related calcifications is virtually pathognomonic for AOT.  Additional general findings across the sections:  Cells are cytologically bland—minimal pleomorphism, mitoses absent.  No ghost cells (excludes calcifying odontogenic cyst) and no sheets of polyhedral cells with amyloid/Liesegang rings (excludes Pindborg tumor).  Lack of tall palisaded peripheral cells with reverse polarization and stellate-reticulum-like centers (helps separate from ameloblastoma).  Taken together, the encapsulated multinodular growth, profuse duct-/rosette-like epithelial structures, intraluminal hyaline material, and scattered calcified “snowflakes” are diagnostic of an adenomatoid odontogenic tumor. |
| 15.29 | Diagnosis: Ameloblastic carcinoma; Key Microscopic Features: Follicular ameloblastoma with hypercellularity on the left; islands of infiltrative tumor on the right. Infiltrative, poorly differentiated tumor composed of atypical basaloid cells with subtle basal nuclear palisading. Ameloblastoma with stellate reticulum-like areas and acanthomatous areas with hypercellularity. Tumor exhibits hypercellularity, nuclear hyperchromasia, and mitotic figures. | Based on the histological features in the four provided hematoxylin and eosin (H&E) stained images at different magnifications, the most consistent histopathological diagnosis is:  Ameloblastoma  Key Microscopic Features Supporting This Diagnosis:  Follicular and Plexiform Patterns (seen especially in the first and second images):  Peripheral columnar ameloblast-like cells with reversed nuclear polarity (nuclei away from the basement membrane).  Central stellate reticulum-like cells resembling the enamel organ in development.  Follicular arrangement of epithelial islands with fibrous connective tissue stroma.  Palisading of Peripheral Cells:  Clear nuclear palisading and reverse polarity are observed at the periphery of epithelial islands, which is a hallmark of ameloblastoma.  Cystic Degeneration and Microcyst Formation:  Focal cystic spaces within epithelial islands are noted, often seen in ameloblastomas undergoing degeneration (visible particularly in the second and third images).  Fibrocollagenous Stroma:  The stroma separating the epithelial islands is relatively mature fibrous connective tissue, with no signs of mesenchymal tumor differentiation—further supporting the diagnosis of a classic ameloblastoma.  Absence of Hard Tissue Formation:  No dentinoid, enamel matrix, or ghost cells are seen, helping rule out other odontogenic tumors such as odontomas or calcifying epithelial odontogenic tumors.  Conclusion:  The histological findings across all magnifications are classic for conventional (solid/multicystic) ameloblastoma, particularly the follicular and plexiform subtypes. These are common benign but locally aggressive odontogenic tumors derived from odontogenic epithelium. | Histopathological diagnosis  → Desmoplastic (hybrid) ameloblastoma  Key microscopic features visible in the photomicrographs  Field What is seen Why it supports the diagnosis  Low-power architecture (1st image, left half) Inter-anastomosing, ribbon-like cords and large cystic/“leaf-like” projections of odontogenic epithelium – a plexiform / follicular pattern This is one of the classic growth patterns of a conventional ameloblastoma.  Low-power architecture (1st image, right half) Numerous small, irregular epithelial islands and cords entrapped in a markedly collagenised (desmoplastic) stroma Extensive desmoplasia with compressed epithelial nests is the hallmark of the desmoplastic variant.  Tumour epithelium – peripheral cells (2nd & 4th images) Peripheral cells are cuboidal-to-columnar, show nuclear palisading and occasional reverse polarity (nucleus pushed away from the basement membrane) Peripheral palisading with reverse polarity is characteristic of ameloblastic epithelium.  Tumour epithelium – central cells (2nd image) Central cells are loosely arranged, resembling stellate-reticulum-like cells; occasional microcystic degeneration Re-creates the enamel-organ‐like arrangement seen in ameloblastoma.  Stroma (all images, esp. 1st & 2nd) Dense, hyalinised collagen with little ground substance; tumour islands appear “squeezed” and may show squamoid change The desmoplastic stromal reaction + compressed nests define desmoplastic ameloblastoma.  Hybrid pattern Conventional plexiform/follicular areas immediately merging with desmoplastic foci within the same lesion WHO describes this as a hybrid desmoplastic–conventional ameloblastoma, but it is usually reported under the umbrella of desmoplastic ameloblastoma.  Collectively, the coexistence of classic ameloblastomatous epithelium and extensive stromal desmoplasia in a single tumour mass pinpoints a desmoplastic (hybrid) ameloblastoma, an accepted subtype within the odontogenic tumour group. |
| 15.31 | Diagnosis: Primary intraosseous squamous cell carcinoma; Key Microscopic Features: Cystic lining showing malignant transformation on the left. Poorly differentiated infiltrating carcinoma arising from the cyst lining. Areas show keratinizing carcinoma with keratin pearl formation. Tumor exhibits dyskeratosis and pleomorphic cells. | Based on the provided hematoxylin and eosin (H&E) stained histological images of the same lesion at varying magnifications, the most likely diagnosis is:  Ameloblastoma (specifically, the acanthomatous variant)  Key Microscopic Features Supporting This Diagnosis:  1. Follicular and Plexiform Patterns (Image 1 - Low magnification)  The tumor shows a multilobular architecture with epithelial islands and strands within a fibrous stroma.  Islands of tumor cells are spread throughout the connective tissue, separated by thin fibrous septae.  2. Peripheral Palisading and Reverse Polarization (Image 2 - Intermediate magnification)  Peripheral columnar or cuboidal cells in the epithelial islands exhibit nuclear palisading and reverse nuclear polarity (nuclei polarized away from the basement membrane), a hallmark of ameloblastoma.  Central areas often resemble stellate reticulum, the embryonic enamel organ.  3. Squamous Metaplasia and Keratin Formation (Image 3 - Higher magnification)  There is acanthomatous change: central areas of epithelial islands show squamous metaplasia and keratin pearl formation, distinguishing it as the acanthomatous variant of ameloblastoma.  No overt signs of dysplasia or malignancy (which would suggest carcinoma).  4. Mild Cellular Atypia, No Infiltration of Adjacent Structures (Image 4 - High magnification)  Despite the epithelial proliferation, the cells remain relatively uniform without significant atypia.  There is keratinization within tumor nests, and fibrous stroma is variably inflamed, which is common in ameloblastomas.  Summary:  These histopathological features — islands of ameloblastic epithelium with peripheral palisading, central stellate reticulum-like areas, and squamous metaplasia with keratin pearl formation — are diagnostic of acanthomatous ameloblastoma, a benign but locally aggressive odontogenic tumor. | Most likely histopathological diagnosis  Squamous odontogenic tumor (SOT) – a benign epithelial odontogenic tumour composed of islands of bland squamous epithelium in a fibrous stroma.  Key microscopic features in the provided H-E sections  Field Salient finding Significance for SOT  Low-power architecture Numerous discrete, irregular islands and strands of epithelium lying immediately beneath an intact oral mucosal surface (first slide). Typical “infiltrative-but-benign” growth pattern of SOT; tumour islands are separate, not fused into large sheets.  Epithelial cytology Islands are formed by bland stratified squamous cells showing obvious intercellular bridges; nuclei are uniform, without atypia or mitoses (second & fourth slides). Confirms the squamous (not ameloblastic, duct-like, basaloid, etc.) nature and benign cytology expected in SOT.  Peripheral cell morphology Peripheral cells are flattened or low cuboidal; no palisading, reverse polarity or columnar shape. Absence of ameloblastic palisading helps rule out conventional or acanthomatous ameloblastoma.  Central changes Frequent concentric lamellated keratin whorls / squamous eddies and small keratin pearls within the epithelial nests (third slide). Characteristic keratinisation pattern of SOT; distinguishes it from simple rests of Malassez or other odontogenic tumours.  Stroma Mature, collagenous connective tissue with scattered lymphocytes and plasma cells; occasional small haemorrhages (all slides). Benign fibrous background is typical; inflammatory infiltrate is a common secondary feature.  Overlying epithelium Surface oral epithelium is unremarkable and not involved by the tumour. Fits with an intra-alveolar/periodontal ligament origin rather than a surface lesion.  Together, this constellation of bland squamous epithelial islands with keratin whorls, lack of ameloblastic features, and placement in a fibrous stroma is diagnostic of a Squamous odontogenic tumour. |
| 15.32 | Diagnosis: Primary intraosseous squamous cell carcinoma; Key Microscopic Features: Infiltrating tumor islands with a vaguely follicular ameloblastomatous appearance, but no basal cell palisading. Keratin pearl formation, dyskeratosis, and nuclear atypia observed. | Based on the provided Hematoxylin and Eosin (H&E) stained histological images of the same lesion at different magnifications, the most likely histopathological diagnosis is:  🦷 Ameloblastoma (specifically, the acanthomatous subtype)  🔍 Justification with Key Microscopic Features:  🔬 Low-power image (first image):  Follicular pattern: Islands or nests of odontogenic epithelium are seen distributed throughout a fibrous stroma.  Peripheral columnar cells: These cells exhibit nuclear palisading and reverse polarization (nuclei oriented away from the basement membrane), which is typical of ameloblastic epithelium.  Central stellate reticulum-like cells: The center of the epithelial nests resembles the stellate reticulum seen in the enamel organ.  🔬 High-power image (second image):  Squamous metaplasia and keratin formation within the central areas of epithelial islands—this is a hallmark of the acanthomatous variant of ameloblastoma.  Presence of keratin pearls, which further confirms squamous differentiation.  The cellular detail in the periphery of the islands still supports the ameloblastic nature of the lesion (columnar cells with reverse polarization and peripheral palisading).  📌 Summary:  The lesion is a benign but locally aggressive odontogenic tumor.  The acanthomatous variant of ameloblastoma shows squamous metaplasia and keratin production within otherwise typical ameloblastic epithelial islands.  These features distinguish it from other entities in the odontogenic tumor spectrum like squamous odontogenic tumor or well-differentiated squamous cell carcinoma. | Histopathological diagnosis (single best):  Acanthomatous (keratinizing) solid/multicystic ameloblastoma  Key microscopic features supporting this diagnosis  Image Key findings Diagnostic significance  Low-power view (first image) • Numerous, irregular islands, cords and anastomosing strands of odontogenic epithelium set in a fibrous connective-tissue stroma.  • Islands show a follicular ± plexiform architecture separated by delicate fibrous septa.  • Central portions of many epithelial nests are eosinophilic and keratinized, some forming laminated whorls.  • Stroma contains a mild, patchy chronic inflammatory infiltrate. Pattern of epithelial proliferation is typical of conventional solid/multicystic ameloblastoma. Presence of keratinized centres points toward the acanthomatous subtype rather than the ordinary follicular/plexiform pattern alone.  High-power view (second image) • Each epithelial nest exhibits a peripheral rim of columnar to cuboidal cells arranged in palisades; their nuclei are oriented away from the basement membrane (--> reverse polarity).  • Centrally, marked squamous metaplasia with formation of well-developed keratin pearls is evident.  • Cells show bland cytology: round–oval nuclei, inconspicuous nucleoli, scant mitotic activity – excluding malignancy. Peripheral palisading with reversed nuclear polarity is the hallmark of ameloblastoma. The pronounced squamous/keratinizing change in the nest centres defines the acanthomatous variant. Lack of cytologic atypia and low mitotic activity distinguish it from ameloblastic carcinoma or primary intra-osseous squamous carcinoma.  Why other odontogenic lesions are unlikely  Squamous odontogenic tumour lacks peripheral palisading/reverse polarity and rarely develops large keratin pearls.  Keratocystic odontogenic tumour (OKC) shows a cystic, not solid, architecture with a corrugated parakeratin lining.  Calcifying epithelial odontogenic tumour (Pindborg) would show sheets of polyhedral cells with amyloid-like material and Liesegang-ring calcifications, none of which are present.  Taken together, the combination of palisaded ameloblast-like peripheral cells, reverse nuclear polarity, and prominent central keratin pearls in solid epithelial islands is diagnostic of acanthomatous ameloblastoma. |
| 15.33 | Diagnosis: Clear cell odontogenic carcinoma; Key Microscopic Features: Lobules of clear cells separated by hyalinized fibrous septa. Cells have clear cytoplasm, mild nuclear atypia, and subtle peripheral nuclear palisading. A mitotic figure present. Perineural and intraneural invasion present. | Based on the histopathological features seen in the three hematoxylin and eosin (H&E) stained images of the same lesion at varying magnifications, the single most likely diagnosis is:  Ameloblastoma  Key Microscopic Features Supporting the Diagnosis:  Follicular and Plexiform Patterns:  The images show follicular arrangements of epithelial islands within a fibrous stroma.  Plexiform architecture is also noted with interconnecting strands and cords of odontogenic epithelium.  Peripheral Palisading of Columnar Cells:  At higher magnification (second image), the peripheral layer of the epithelial islands shows columnar cells with nuclear palisading and reverse polarization (nuclei away from the basement membrane), a classic feature of ameloblastoma.  Central Stellate Reticulum-like Areas:  The central portion of the islands resembles the stellate reticulum of the enamel organ, appearing loosely arranged and more eosinophilic, with spindle to polygonal-shaped cells.  Lack of Hard Tissue Formation:  Despite being an odontogenic tumor, there is no evidence of dentin or enamel matrix, helping to rule out other odontogenic tumors such as ameloblastic fibro-odontoma or odontoma.  Cystic Degeneration:  Some epithelial islands exhibit central cystic degeneration, a feature that may be seen in ameloblastomas, especially of the follicular subtype. | Diagnosis – Clear-cell odontogenic carcinoma (CCOC)  Histologic hallmarks present in the three H-E fields  Field What is seen Why it points to CCOC  Low-power (first image) Numerous irregular nests / lobules of odontogenic epithelium permeating a markedly hyalinised, eosinophilic, desmoplastic stroma CCOC is characteristically infiltrative and surrounded by a thick basement-membrane-like, pink, hyalinised collagen; desmoplasia is far less prominent in other clear-cell lesions such as AOT or CEOT.  Intermediate-power (second image) Each epithelial island shows a biphasic cell population:  • Peripheral rim of cells with clear, vacuolated cytoplasm and small dark nuclei (glycogen-rich clear cells).  • Central polygonal/squamoid cells with more eosinophilic cytoplasm.  Occasional peripheral nuclear palisading is evident. The classic “biphasic” pattern (clear cells + eosinophilic cells) is the commonest architectural subtype of CCOC. The clear cytoplasm results from glycogen (PAS-positive / diastase-labile) rather than mucin or lipid.  High-power (third image) A large lobule shows:  • Sharp interface between clear peripheral cells and a core of smaller polygonal eosinophilic cells.  • Scattered mitoses and mild pleomorphism.  • No calcifications, ghost cells, or amyloid. The clear-to-eosinophilic cell transition and absence of calcifying/amyloid material distinguish CCOC from calcifying epithelial odontogenic tumour and calcifying cystic odontogenic tumour. Focally increased mitotic activity supports its malignant nature.  Key diagnostic features, summarised  Infiltrative nests, cords and trabeculae of odontogenic epithelium within a densely hyalinised desmoplastic stroma.  Predominant clear cells with PAS-positive glycogen-rich cytoplasm and small, often compressed nuclei.  Biphasic arrangement – clear cells admixed with or surrounding polygonal eosinophilic/squamoid cells.  Peripheral nuclear palisading / ameloblast-like rimming in some islands.  Absence of duct-like structures, calcifications, amyloid or ghost cells, helping to exclude AOT, CEOT and calcifying cystic odontogenic tumour.  Focal mitoses and mild cytologic atypia, consistent with a low-grade carcinoma.  Taken together, these microscopic findings are diagnostic of clear-cell odontogenic carcinoma. |
| 15.33b | Diagnosis: Clear cell odontogenic carcinoma; Key Microscopic Features: Irregularly shaped islands and cords of epithelium with clear cells. Tumor cells exhibit eosinophilic or clear cytoplasm, and the stroma is densely hyalinized. Islands and cords of primarily clear cells with hyalinized stroma. | Based on the provided hematoxylin and eosin (H&E) stained histopathology images of a lesion from the odontogenic tumors group, captured at varying magnifications, the most likely histopathological diagnosis is:  Clear Cell Odontogenic Carcinoma (CCOC)  Justifying Microscopic Features:  1. Clear Cell Population:  Seen prominently in the highest magnification image.  Cells with clear cytoplasm due to glycogen accumulation (PAS-positive, diastase-labile), a hallmark of clear cell neoplasms.  2. Biphasic Cell Population:  Presence of both clear cells and more basaloid, hyperchromatic cells in clusters and islands.  This biphasic pattern is characteristic of CCOC.  3. Infiltrative Growth Pattern:  Low and intermediate magnification views show irregular nests and cords of tumor cells infiltrating a fibrous stroma.  Suggests aggressive behavior and supports malignancy.  4. Desmoplastic/Fibrous Stroma:  Abundant fibrous stroma surrounding tumor islands, often seen in CCOC.  5. Peripheral Palisading and Reverse Polarity:  In some areas, peripheral palisading of tumor cells resembling ameloblastomatous differentiation may be seen, which can mimic other odontogenic tumors but is part of the histologic spectrum of CCOC.  Differential Diagnosis Considerations (excluded based on features):  Clear Cell Variant of Calcifying Epithelial Odontogenic Tumor (CEOT) – lacks amyloid and Liesegang rings.  Clear Cell Mucoepidermoid Carcinoma – lacks odontogenic origin and typically has mucous and intermediate cells.  Metastatic Renal Cell Carcinoma – systemic correlation needed; unlikely in purely odontogenic context. | Most likely histopathological diagnosis  Clear-cell odontogenic carcinoma (CCOC) — a malignant odontogenic epithelial tumour characterised by nests and cords of glycogen-rich clear cells admixed with basaloid cells in a hyalinised stroma.  Key microscopic features in the provided H-E sections that support this diagnosis  Feature seen How it appears in your images Diagnostic relevance  Two distinct epithelial cell populations (biphasic pattern) • Sheets/nests rimmed by small, dark, basaloid cells (2nd image)  • Centrally, many cells have pale-to-empty (“clear”) cytoplasm with distinct cell borders (3rd image) Classic for CCOC; peripheral basaloid cells and central glycogen-rich clear cells  Abundant clear cytoplasm Numerous tumour cells show soap-bubble-like vacuolated cytoplasm with centrally placed nuclei (3rd image) Cytoplasmic clearing results from dissolved glycogen (PAS-positive/diastase-labile) and is a hallmark of CCOC  Cribriform / duct-like and trabecular growth within a dense fibrous stroma Inter-anastomosing cords and pseudoglandular spaces throughout a collagen-rich stroma (low-power, 1st image) CCOC often shows an adenoid-cystic-like cribriform or trabecular architecture rather than the follicular/plexiform pattern of classic ameloblastoma  Hyalinised stromal reaction Islands are separated by conspicuous, eosinophilic, acellular fibrous bands (all images) Hyalinised stroma is typical and helps separate CCOC from other clear-cell rich lesions  Cytologic atypia and mitotic figures Hyperchromatic, pleomorphic nuclei and scattered mitoses among both clear and basaloid cells (best appreciated in 2nd image) Confirms malignant nature; distinguishes from benign clear-cell variants of ameloblastoma  Infiltrative borders Irregular tongues and nests permeate the fibrous tissue without a capsule (1st image) Supports carcinoma rather than encapsulated tumours such as AOT  These combined features—especially the biphasic clear/basaloid cell population in a hyalinised, infiltrative stroma—are diagnostic of clear-cell odontogenic carcinoma. |
| 15.34 | Diagnosis: Odontogenic Carcinoma with Dentinoid Deposition; Key Microscopic Features: Tumor cells arranged in cords with significant dentinoid deposition. Some tumor cells exhibit clear cytoplasm and hyperchromatic nuclei. | Based on the histopathological features observed in the provided hematoxylin and eosin (H&E) stained images at low, intermediate, and high magnification, the single most likely diagnosis is:  Ameloblastoma  Key Microscopic Features Justifying the Diagnosis:  Follicular Pattern (seen at intermediate to high magnification):  Islands or nests of odontogenic epithelium within a fibrous stroma.  Peripheral columnar or cuboidal cells with nuclear palisading and reverse polarization (nuclei oriented away from the basement membrane).  Central cells resembling stellate reticulum, which is a characteristic feature of enamel organ epithelium.  Fibrous Stroma:  The islands are surrounded by a mature, fibrous connective tissue stroma, which is typical in ameloblastoma.  No Signs of Maturation into Hard Tissues (e.g., Dentin or Enamel):  This helps differentiate ameloblastoma from other odontogenic tumors like ameloblastic fibro-odontoma or odontoma.  Lack of Mitotic Figures or Cytological Atypia:  Despite being locally aggressive, ameloblastomas typically exhibit bland cytology. | Histopathological (H-E) diagnosis  Ameloblastic fibro-odontoma (AFO) – a mixed odontogenic tumour that shows an ameloblastic-fibroma-like soft-tissue component together with formation of both dentin and enamel matrix.  Key microscopic features in the three photomicrographs  Field What is seen Why it points to AFO  Low-power overview (image 1) A broad, well-circumscribed central block of eosinophilic mineralised tissue occupies the core of the lesion. Along one side there is a 1-2 mm thick, very cellular ribbon of soft tissue lying beneath the surface mucosa. A central mass of developing hard dental tissues with a peripheral zone rich in epithelial and ectomesenchymal elements is the classic architecture of a mixed odontogenic tumour in the “developing odontoma/AFO” spectrum.  Intermediate magnification (image 2) • The central hard tissue shows tubular dentin; between many tubules are empty, ovoid “clefts” representing lost enamel matrix after decalcification.  • Immediately adjacent to these mineralised areas is a highly cellular odontogenic epithelium arranged in anastomosing cords and strands; the surrounding stroma is very cell-rich and resembles primitive dental papilla. Concurrent production of dentin and enamel matrix (vacuolated spaces) distinguishes AFO/complex odontoma from an ameloblastic fibroma (which lacks hard tissue). The intimate association of ameloblastic epithelium with primitive ectomesenchyme is typical of the ameloblastic-fibroma component.  High power (image 3) • Epithelial cords show peripheral columnar/ameloblast-like cells with nuclear polarisation and occasional central cells mimicking stellate reticulum.  • Plump, fibroblastic mesenchymal cells intermingle with the epithelial strands.  • Juxta-epithelial odontogenic hard tissue—eosinophilic dentinoid rimmed by active cells—is obvious. The biphasic (epithelial + mesenchymal) proliferation with cytologic blandness, coupled with juxta-epithelial dentin/enamel formation, is diagnostic of AFO and rules out other odontogenic tumours (e.g., adenomatoid OT, calcifying epithelial OT, ameloblastoma).  Bottom line:  The coexistence of a cell-rich ameloblastic fibroma–like soft-tissue component and actual formation of both dentin and enamel matrix in the same lesion is pathognomonic for ameloblastic fibro-odontoma. |
| 15.35 | Diagnosis: Sclerosing Odontogenic Carcinoma; Key Microscopic Features: Small epithelial nests and cords proliferate in a dense collagenous stroma, with fibroosseous-like areas present. Cells exhibit nuclear atypia and lack cohesion. | Histopathological Diagnosis:  Ameloblastic Fibro-Osteosarcoma (AFOS)  Justification Based on Key Microscopic Features:  Across the images at varying magnifications, the histopathological features are highly characteristic of Ameloblastic Fibro-Osteosarcoma, a rare malignant odontogenic tumor. The following key observations support this diagnosis:  1. Biphasic Tumor Pattern (seen at lower magnification - Image 1):  The lesion displays both epithelial and mesenchymal components.  The epithelial component forms islands and strands resembling ameloblastic epithelium.  The mesenchymal stroma is cellular and sarcomatous, unlike benign counterparts like ameloblastic fibroma.  2. Cellular Mesenchymal Stroma with Atypia (Image 2 & 3):  The mesenchymal component shows marked cellularity with spindle to oval cells.  Nuclear pleomorphism, hyperchromatism, and mitotic activity are evident—hallmarks of malignancy.  The cellular features distinguish it from benign fibro-osseous lesions or ameloblastic fibroma.  3. Osteoid and Immature Bone Formation (Image 4):  Areas of irregular osteoid matrix deposition and woven bone are seen, often surrounded by atypical mesenchymal cells.  This confirms the osteogenic differentiation within a sarcomatous stroma, consistent with the "osteosarcoma" component of AFOS. | Histopathological diagnosis  Central cemento-ossifying fibroma (central ossifying/cementifying fibroma of the jaws)  Key microscopic features that point to this diagnosis  Field Findings in the provided slides Diagnostic significance  Architectural outline (low-power) Sharply demarcated, lobulated mass that “shells out” from surrounding tissue, showing clefts between lesion and adjacent bone Benign, well-circumscribed neoplasm rather than an infiltrative process  Stromal component Uniform, highly cellular fibro-blastic tissue composed of spindle cells in interlacing fascicles and whorls; occasional myxoid foci but no cytologic atypia or brisk mitoses The fibro-cellular background is the “fibroma” part of the tumour  Mineralised component Numerous rounded or irregular deposits of mineralised material: * • deep-eosinophilic, acellular spherical masses resembling cementum (“cementicles”) * • trabeculae of woven bone/osteoid containing osteocyte-like lacunae, sometimes bordered by plump osteoblast-/cementoblast-like cells Combination of bone-like and cementum-like calcifications is characteristic of cemento-ossifying fibroma  Interface between stroma and calcifications Distinct fibrous capsule/cleft around most calcified bodies; reversal-line-type borders seen on many ossicles Typical of ossifying fibroma and helps separate it histologically from fibrous dysplasia  Odontogenic epithelium Essentially absent or limited to a few inconspicuous rests; no epithelial nests forming enamel- or dentin-like structures Rules out ameloblastic fibro-odontoma / fibro-dentinoma and supports a pure fibro-osseous lesion  Cytology Spindle cells have bland, ovoid nuclei and scant cytoplasm; no pleomorphism or abnormal mitoses Confirms benign nature  The combination of a well-circumscribed fibrous lesion containing both woven bone and cementum-like calcifications—without the epithelial component seen in mixed odontogenic tumours—pins down the diagnosis as a central cemento-ossifying fibroma. |
| 15.37 | Diagnosis: Compound Odontomas; Key Microscopic Features: Tooth-like structures featuring enamel matrix, dentin, and dental papilla. | The two hematoxylin and eosin (H&E) stained images provided appear to depict an odontogenic lesion, which is common in oral and maxillofacial pathology.  Histopathological Diagnosis:  The lesion is most likely an ameloblastoma. This is a benign but locally aggressive tumor originating from odontogenic epithelium. It is characterized by distinct histopathological features.  Key Microscopic Features in the Images:  First Image:  The lesion shows odontogenic epithelial structures forming islands or cords within a fibrous stroma.  There is an indication of reversal of polarity where the columnar cells exhibit a tendency to arrange at the periphery, often with a palisading arrangement. This is a characteristic feature of ameloblastoma.  The presence of stromal fibrosis can also be noted, which is typical of the tumor’s progression and local invasiveness.  Second Image:  This magnified view demonstrates the peripheral cells arranged in a palisading pattern, which is another hallmark of ameloblastoma.  The central cells are more loosely arranged and resemble the stellate reticulum-like cells, contributing to the diagnosis of ameloblastoma.  The basal layer of the epithelium appears quite prominent, showing hyperchromatic nuclei, which is another sign of ameloblastic proliferation. | Based on the provided hematoxylin-and-eosin (H&E) images of the lesion within the "Odontogenic Tumors" group, the likely histopathological diagnosis and key microscopic features are as follows:  Diagnosis: Ameloblastoma  Key Microscopic Features:  Image 1:  The lesion shows epithelial islands with a palisaded arrangement of cells along the periphery, typical of ameloblastoma. The cells at the periphery are columnar with a reverse polarity of the nuclei (nuclei located toward the basement membrane).  Stroma is somewhat myxoid with a loose connective tissue appearance, which is characteristic of the central part of the lesion.  The presence of bony involvement or the association with bone-like tissue (as seen in the image) suggests that the lesion is pushing through the surrounding bone, a hallmark of the more invasive nature of ameloblastomas.  Image 2:  Cellular islands in the lesion show a characteristic stellate reticulum-like appearance in some areas, which is seen in ameloblastoma.  The basal layer of epithelial cells shows a palisading pattern with some areas exhibiting more compact, columnar cells, which is another characteristic of the ameloblastoma’s histological appearance.  The tumor demonstrates a layering pattern, with the superficial cells being more cuboidal and the deeper cells resembling the ameloblast-like cells seen in the tumor.  Justification:  Ameloblastoma is a benign but locally aggressive odontogenic tumor. The hallmark microscopic feature of ameloblastoma is the palisading of epithelial cells with a reverse nuclear polarity. The lesion often demonstrates islands or cords of epithelial cells within a myxoid or fibrous stroma. This pattern is evident in both images, which fit the diagnosis of ameloblastoma. |
| 15.38 | Diagnosis: Complex Odontoma; Key Microscopic Features: Disorganized arrangement of enamel matrix, dentin, and odontogenic epithelium, with areas of dark pink enameloid. | The images provided show histopathological features of a lesion within the category of odontogenic tumors, captured at different magnifications. Based on the characteristic features observed in the images, the diagnosis is most likely Ameloblastoma, a benign but locally aggressive odontogenic tumor.  Key Microscopic Features Observed:  Multilocular appearance: Several of the images exhibit a cystic or multilocular pattern (multiple cavities or spaces within the tumor). This is a classic feature of ameloblastoma, which often presents with a "soap bubble" or "honeycomb" appearance.  Cystic spaces: Some images show well-defined cyst-like spaces with peripheral cells resembling odontogenic epithelium. These spaces are lined by columnar or cuboidal cells with hyperchromatic nuclei.  Peripheral columnar cells with reverse polarity: In certain areas, cells at the periphery of cystic structures show a columnar or cuboidal shape with nuclei that are displaced to the basal side (reverse polarity), which is a hallmark of the ameloblast-like epithelium.  Fibrous stroma: A fibrous background stroma can be observed between the neoplastic epithelial islands. This is characteristic of ameloblastomas, where the epithelial islands are often surrounded by a dense fibrous stroma.  Basal layer hyperplasia: The basal layer of the epithelium shows hyperplasia and may demonstrate palisading of cells, another feature associated with ameloblastoma.  These findings, especially the multilocular pattern, cystic spaces, and characteristic epithelial arrangement, point strongly towards a diagnosis of ameloblastoma. | The images you uploaded seem to depict histopathological slides of a lesion. Upon analysis of these hematoxylin-and-eosin (H&E) stained sections, I can provide an overall diagnosis and describe the microscopic features observed.  Single Most Likely Histopathological Diagnosis:  Ameloblastoma (a type of odontogenic tumor)  Key Microscopic Features:  Multilocular Architecture: The images show a multilocular (soap bubble) appearance, often seen in ameloblastomas. This feature is typical of the tumor's cystic, expansile nature.  Columnar and Cuboidal Cells: The epithelium lining the cystic spaces displays a distinctive pattern of columnar or cuboidal cells, resembling ameloblasts (the cells from which this tumor arises). The nuclei are often polarized away from the basement membrane, and the cytoplasm may appear pale.  Peripheral Palisading: The peripheral cells of the tumor display palisading, where the cells are arranged in a columnar fashion along the periphery of the tumor islands or cyst-like structures.  Fibrous Stroma: The intervening stroma is dense, fibrous, and often shows varying degrees of vascularization. This fibrous tissue supports the tumor islands.  Calcification or Areas of Keratinization: Some areas exhibit foci of keratinization or dystrophic calcifications, which are common in more mature or degenerative stages of ameloblastoma.  These features, particularly the multilocular cystic structures, palisaded cell layers, and fibrous stroma, strongly suggest an ameloblastoma, particularly the multicystic (or "classic") variant.  Please let me know if you need further clarification on the findings. |
| 15.39 | Diagnosis: Complex Odontoma; Key Microscopic Features: Masses of tubular dentine and eosinophilic dentinoid, interspersed with ghost cells and lamellar calcifications. | Based on the provided images, which are histological slides stained with hematoxylin and eosin (H&E) and representing odontogenic tumors, I will analyze and provide the following diagnosis and key microscopic features.  Most Likely Diagnosis: Ameloblastoma  Key Microscopic Features Supporting the Diagnosis:  Multilocular Lesion Appearance:  The first image shows a lesion with a characteristic "soap bubble" or "honeycomb" pattern, which is highly suggestive of ameloblastoma, particularly the multicystic or "plexiform" type. The presence of multiple cystic spaces surrounded by dense fibrous tissue is a hallmark of this lesion.  Presence of Basal Layer of Columnar Cells:  The second image shows epithelial cells arranged in a palisading pattern with a columnar or cuboidal basal layer, which is one of the key features of ameloblastoma. The cells show hyperchromatic nuclei, which are indicative of cell proliferation.  Deposits of Amorphous Substance:  In the third image, the amorphous, eosinophilic material present in some of the cystic spaces could be indicative of the presence of a matrix, a feature often seen in ameloblastomas. This matrix may be a calcified or mucoid substance, which is common in this tumor type.  Peripheral Palisading of Tumor Cells:  The palisading of the epithelial cells around the cysts is visible, reinforcing the diagnosis of ameloblastoma, where the basal layer of the epithelial cells tends to form a distinct, well-organized row along the perimeter of the cyst-like spaces.  Fibrous Stroma:  A prominent fibrous stroma is seen between the cystic spaces, which is typical in ameloblastoma. The stroma contains varying amounts of collagen and may show areas of edema or cyst formation. | Based on the images you've provided, the histopathological diagnosis for this lesion is most consistent with Ameloblastoma, a benign odontogenic tumor.  Key Microscopic Features:  Ameloblastoma-like architecture: The first image shows cystic structures with a characteristic "stellate reticulum-like" appearance. There are several islands of epithelial cells that have peripheral columnar cells with reverse polarization, which is a hallmark of the ameloblastoma type.  Multilocular appearance: The second image reveals large areas of a cystic, multilocular arrangement of the tumor, which is typically seen in the classic type of ameloblastoma. This pattern is indicative of the solid/multilocular variant of ameloblastoma.  Keratinization: In the third image, there is some evidence of keratinized areas and a dense fibrous stroma around the tumor islands. This suggests an older lesion or a more aggressive growth form, but still within the context of ameloblastoma.  These features—distinct islands of ameloblast-like epithelium, cystic and multilocular structures, and areas of keratinization—align with a solid or multicystic ameloblastoma, a benign but locally aggressive lesion that arises from the odontogenic epithelium. |
| 15.39b | Diagnosis: Complex Odontoma; Key Microscopic Features: Structures composed mostly of tubular dentin surrounded by odontogenic epithelium with ghost cells and calcifications. | Based on the provided images showing a lesion in the "Odontogenic Tumors" group, I can make the following observations and analysis:  Histopathological Diagnosis:  The lesion is most consistent with Ameloblastoma, which is a common benign but locally aggressive odontogenic tumor.  Key Microscopic Features:  Epithelial Islands and Cystic Spaces:  The first image shows a large cystic space with epithelial islands. These islands typically consist of cuboidal or columnar cells with hyperchromatic nuclei, which are often seen in ameloblastomas.  In the second image, you can see further details of the epithelial islands, with a more organized appearance, often resembling the ameloblast-like cells that are characteristic of ameloblastomas.  Peripheral Ameloblast-Like Cells:  The tumor shows a characteristic arrangement of peripheral cells resembling ameloblasts (columnar cells with prominent reverse polarity, meaning the nuclei are located away from the basement membrane). These are classic findings in the histopathology of ameloblastoma.  Stromal Fibrovascular Tissue:  The stroma surrounding the islands appears to be cellular and fibrovascular in nature, which is another distinguishing feature of ameloblastomas.  Multilocularity:  The cystic or multinodular pattern is common in ameloblastomas, with a mix of solid and cystic areas, and this can be appreciated in both images. | Based on the images provided, here's the analysis:  Histopathological Diagnosis:  The lesion in the images most likely represents Ameloblastoma, a benign but locally aggressive odontogenic tumor.  Key Microscopic Features:  Cystic Spaces and Multilocular Appearance (first image):  The lesion shows large cystic spaces filled with a pinkish, eosinophilic material. These spaces are characteristic of ameloblastomas, particularly the multilocular (or "soap bubble") variant.  The periphery of these cystic spaces exhibits thin layers of epithelial cells, some of which resemble ameloblasts, the hallmark cell type of ameloblastoma.  Peripheral Ameloblast-like Cells (second image):  At a higher magnification, the lesion demonstrates islands and cords of cells that resemble ameloblasts. These cells are tall and columnar with palisading at the basal layer, a defining feature.  The stroma in between the islands is fibrous and can show areas of cellular pleomorphism, which is typical of ameloblastoma.  A combination of solid epithelial islands and cystic spaces confirms the diagnosis.  Dense Stroma and Cellular Features:  The second image reveals areas of dense stroma with a mixture of smaller and larger epithelial islands. Some islands contain larger, less organized cells with hyperchromatic nuclei, adding to the aggressiveness and diagnostic features of ameloblastoma.  Overall, the combination of cystic spaces, peripheral ameloblast-like cells, and fibrous stroma strongly supports a diagnosis of ameloblastoma. |
| 15.41 | Diagnosis: Ameloblastic Fibroodontoma; Key Microscopic Features: A combination of ameloblastic fibroma and complex odontoma, showing tubular dentin, dentinoid, and odontogenic epithelium within a myxoid stroma. | Based on the provided histopathological images, the most likely diagnosis for this group of images is Ameloblastoma, a common odontogenic tumor of the jaw.  Key Microscopic Features Justifying the Diagnosis:  Plexiform Pattern: The tumor shows a plexiform arrangement, where islands of odontogenic epithelium are arranged in a network-like pattern (seen in several of the images). This is a classic feature of ameloblastoma.  Columnar Cells with Palisading: The presence of columnar cells with palisading at the periphery of the epithelial islands, exhibiting a characteristic reverse polarity (where the nuclei are displaced away from the basement membrane), is also a hallmark of ameloblastoma.  Cystic Spaces: There are multiple cyst-like spaces within the lesion, some of which contain serous or mucous fluid, suggesting a cystic form of ameloblastoma, a common variant.  Basal Layer Hyperplasia: The tumor epithelium shows hyperplasia with prominent basal layers, contributing to the characteristic appearance of the lesion.  Fibrous Stroma: The fibrous stroma around the epithelial islands, which appears dense and well-vascularized, is consistent with the tissue architecture of ameloblastomas.  These histopathological findings, including the typical plexiform growth pattern, palisading columnar cells, and cystic spaces, strongly support the diagnosis of Ameloblastoma, a benign but locally aggressive odontogenic tumor. | To provide you with the most accurate analysis of the provided hematoxylin-and-eosin (H&E) stained images, I'll review and analyze the key microscopic features of the lesion. However, as I cannot directly access and interpret the images through this interface, could you please provide a detailed description or any specific histopathological features you'd like to discuss?  In general, odontogenic tumors can present with varying features, such as:  Cystic spaces or structures within the lesion, often seen in lesions like ameloblastomas.  Columnar or cuboidal cells at the periphery of cyst-like spaces or nests.  Presence of calcifications or dentin-like material, which may be seen in certain odontogenic lesions.  Abnormal growth of odontogenic epithelium, such as hypercellularity or pleomorphism.  If you'd like, I can walk you through some of these characteristics based on the images you uploaded. Let me know how you would like to proceed! |
| 15.42 | Diagnosis: Ameloblastic Fibroodontoma; Key Microscopic Features: A myxoid stroma with odontogenic epithelium and plump spindle cells, accompanied by tubular dentin and enamel matrix. | Based on the uploaded images, I will proceed to analyze the histopathological features of the lesion under different magnifications to help determine a diagnosis.  Let me first review the slides before providing an interpretation.  I've displayed the images you provided. After reviewing them, I'll now analyze the histopathological features to provide a diagnosis.  From these images, the lesion shows the following key features:  Microscopic Features:  Presence of epithelial cells arranged in islands or strands within a fibrous connective tissue stroma.  Cystic or gland-like spaces can be noted within these islands.  The cells in the islands demonstrate hyperchromatic nuclei and occasional palisading at the periphery, which suggests the presence of odontogenic epithelium.  In some areas, the epithelium is seen surrounded by fibrous stroma, which further supports a benign odontogenic tumor.  Diagnosis:  The lesion is consistent with Ameloblastoma, a benign but locally aggressive odontogenic tumor.  The characteristic histopathological finding is the presence of epithelial islands with palisading of the basal cells, often surrounded by a fibrous stroma. The cystic spaces or follicles within the lesion can also be part of the histological appearance. | Based on the provided images and their characteristics, the lesion appears to be consistent with an odontogenic cyst or odontogenic tumor, specifically dentigerous cyst. Here are the key histopathological features that support this diagnosis:  Key Microscopic Features:  Lesion Architecture:  The images show cystic spaces surrounded by a thin layer of epithelial cells, consistent with a cystic lesion.  Epithelial Lining:  The cystic spaces are lined by a layer of non-keratinized squamous epithelium, which is a key feature of odontogenic cysts like the dentigerous cyst.  Peripheral Ossification:  In some of the images, there is evidence of surrounding bony involvement, which can suggest the relationship to the dental structures and support the diagnosis of a dentigerous cyst, which typically surrounds the crown of an unerupted tooth.  Fibrous Connective Tissue:  The stroma or underlying connective tissue appears to be relatively fibrous with minimal inflammation, a hallmark of odontogenic cysts or tumors.  Cellularity of the Lining:  The epithelial lining appears to have areas of hypercellularity, which is also a characteristic of this type of cyst.  Diagnosis:  Dentigerous Cyst (or Follicular Cyst)  This diagnosis is further supported by the relationship to the crown of an impacted or unerupted tooth, a typical feature of dentigerous cysts. The lesion often appears as a well-circumscribed radiolucent area around the crown of an unerupted tooth in radiographic imaging, although the provided histopathological analysis does not directly show this. The cystic lining and absence of significant inflammation or malignancy further confirm this diagnosis. |
| 15.42b | Diagnosis: Ameloblastic Fibro-dentinoma; Key Microscopic Features: Cords of ameloblastoma-like epithelium within myxoid stroma and the presence of dentinoid without enamel matrix. | The images provided appear to be histopathological slides of an odontogenic lesion. Based on the visual features in these images, the most likely diagnosis is Ameloblastoma.  Key Microscopic Features Supporting the Diagnosis:  Multilocular Cystic Appearance:  In the first image, you can observe areas with a multilocular (honeycomb-like) pattern, which is characteristic of ameloblastomas, specifically the plexiform type.  Follicular Structures:  The second image shows nests and islands of odontogenic epithelial cells, forming follicular patterns. These are indicative of the follicular type of ameloblastoma.  Columnar Cells with Hyperchromatic Nuclei:  In the second and third images, the presence of columnar cells with palisading at the periphery of the epithelial islands, along with hyperchromatic nuclei, further supports ameloblastoma.  Variable Stroma:  The third image demonstrates an irregular, fibrous stroma that is commonly seen in ameloblastomas, which can vary in density.  These findings—multilocular cystic formation, follicular structures, columnar cells with palisading, and the fibrous stroma—are hallmark features of Ameloblastoma. | The images you provided appear to show histopathological slides of a lesion within the category of odontogenic tumors. Upon analyzing the images, here is the most likely histopathological diagnosis along with the key features that support this conclusion:  Diagnosis: Ameloblastoma  Key Microscopic Features:  Lesion Architecture:  The first image displays a multilocular, cystic lesion with islands of epithelial cells surrounded by a stroma. The presence of these discrete islands or clusters of cells is a characteristic feature of ameloblastoma.  In the second image, a more focused view of the lesion shows islands of epithelial cells with a peripheral layer resembling the ameloblast-like cells and central areas resembling stellate reticulum. This is a hallmark of the "follicular pattern" seen in ameloblastomas.  Ameloblast-Like Cells:  The images show peripheral cells lining the islands with a tall, columnar, and hyperchromatic appearance. These resemble the ameloblasts that are found in the enamel organ during tooth development. This is a defining feature of the tumor.  Stellate Reticulum-Like Spaces:  The presence of stellate reticulum-like cells, which appear as more loosely arranged cells within the islands (as seen in the third image), is a characteristic finding in ameloblastomas, especially in the "follicular type."  Fibrous Stroma:  The stroma surrounding the islands of epithelial cells is fibrous and may be variable in cellularity, a common finding in ameloblastomas.  Cystic Spaces:  Cystic spaces surrounding the epithelial islands, as shown in the first image, are frequently encountered in ameloblastomas, particularly the "multilocular" type.  These features collectively suggest an ameloblastoma, which is a benign but locally aggressive tumor originating from the odontogenic epithelium. |
| 15.44 | Diagnosis: Ameloblastic Fibroma; Key Microscopic Features: A proliferation of ameloblastoma-like epithelium in a follicular pattern, accompanied by fibroblast-like cells in a myxoid-mucinous stroma. | Based on the provided images, it appears to be a case of an Odontogenic Keratocyst (OKC), a type of odontogenic tumor. Let's break down the diagnosis and the key microscopic features seen in the images:  Diagnosis:  Odontogenic Keratocyst (OKC)  Key Microscopic Features:  Cystic Architecture:  Both images show a cystic lesion with a well-defined lining. The cyst is filled with a clear fluid and surrounded by a thin lining, characteristic of OKC.  Orthokeratinized Epithelium:  The cyst lining in both images appears to be composed of a uniform layer of epithelium, some of which is showing orthokeratinization. This is typical of OKC, which has a parakeratinized epithelium in some cases, but in others, it may be orthokeratinized.  Palissading of the Basal Cells:  The basal layer of the cystic lining shows a characteristic palisading pattern, where the basal cells are columnar and aligned, contributing to the overall diagnosis of OKC. This feature is particularly visible in the first image.  Hyperchromatic Cells:  The basal epithelial cells exhibit hyperchromasia (dark staining), indicative of cellular activity or mitotic potential, which is often seen in OKC.  Thin Fibrous Capsule:  Both images show a relatively thin fibrous capsule surrounding the lesion, typical of OKCs, which distinguishes them from other odontogenic cysts or tumors that might have a thicker capsule.  These microscopic features, particularly the thin epithelial lining, the palisading basal cells, and the cystic nature of the lesion, all support the diagnosis of an Odontogenic Keratocyst. | The provided images represent histopathological slides that are part of the odontogenic tumor group. Based on the microscopic appearance, here is the analysis:  Histopathological Diagnosis:  Ameloblastoma  Key Microscopic Features:  Follicular Architecture:  The first image shows a pattern consistent with a follicular type of ameloblastoma. The tumor is composed of islands or follicles of odontogenic epithelium surrounded by a loose, mature stroma.  The second image demonstrates closely packed tumor islands with a peripheral layer of columnar or cuboidal cells resembling ameloblasts, which is a classic feature of ameloblastoma.  Ameloblastic Cells:  In both images, the tumor islands are lined by cells with large, hyperchromatic nuclei and prominent nucleoli, often resembling ameloblasts, which is indicative of the characteristic architecture seen in ameloblastomas.  Stromal Cells:  The intervening stroma in both images appears loose and fibrous, which is typical for ameloblastomas. The stroma is not dense or inflammatory, distinguishing it from other types of odontogenic lesions.  Cystic Spaces:  In some regions of the images, there are cyst-like spaces within the tumor islands, which is another feature that can be seen in ameloblastomas.  These histopathological features, including follicular architecture, ameloblastic differentiation, and a fibrous stroma, point toward an ameloblastoma as the diagnosis. |
| 15.45 | Diagnosis: Ameloblastic fibrosarcoma. Key Microscopic Features: Ameloblastoma with hypercellular spindle cell proliferation; benign ameloblastoma with stellate reticulum-like areas and highly cellular proliferation of atypical spindle cells; atypical spindle cells with pleomorphic overlapping nuclei. | The images provided appear to be histopathological sections of a lesion under hematoxylin and eosin (H&E) staining, representing different magnifications of an odontogenic tumor.  Single Most Likely Histopathological Diagnosis:  The histopathological features in the provided images most likely represent Ameloblastoma, specifically the follicular type, which is a common benign odontogenic tumor of the jaw.  Key Microscopic Features Justifying This Diagnosis:  Multilocular Cystic Spaces: In the first image, you can observe the presence of multiple cyst-like spaces, which are a hallmark feature of ameloblastoma. These spaces are lined by epithelial cells.  Peripheral Columnar Cells (Ameloblast-like Cells): In the second image, a prominent feature is the peripheral layer of tall columnar cells resembling ameloblasts. These cells often have reversed polarity of their nuclei and are characteristic of the follicular pattern seen in ameloblastomas.  Reticular Connective Tissue: In the third image, the stroma around the epithelial islands is loose and resembles the reticular connective tissue, a feature seen in ameloblastomas. The tumor's stroma is usually fibrous but may show a more loose, collagenized pattern, consistent with the follicular type of ameloblastoma.  These features are critical in identifying ameloblastoma, particularly the characteristic follicular arrangement with peripheral columnar cells and central stellate reticulum-like cells within the cystic spaces. | Based on the provided hematoxylin-and-eosin (H&E) images, I am able to provide the following analysis:  Most Likely Histopathological Diagnosis:  The histopathological features observed in the images suggest Ameloblastoma, a benign odontogenic tumor. This diagnosis is supported by the distinctive microscopic features typical of ameloblastomas.  Key Microscopic Features Supporting the Diagnosis:  Image 1:  The first image shows cyst-like structures with a well-defined epithelial lining. The presence of these cystic spaces within the lesion, along with a prominent outer epithelium, is a classic feature of ameloblastomas, which often show cystic or follicular growth patterns.  The epithelial lining in these spaces demonstrates prominent basaloid cells with palisading at the periphery (outer layer) and a more loosely arranged stellate reticulum-like area in the center.  Image 2:  The second image shows a more developed follicular pattern, where the central area of the lesion exhibits a well-organized arrangement of ameloblast-like cells surrounding a central, often hypocellular, area.  The peripheral cells show prominent palisading with reverse polarity of the nuclei, which is characteristic of ameloblastomas.  Image 3:  The third image depicts a more cellular area of the lesion with small, uniform cells arranged in a disorganized manner. This cellularity and arrangement also support the diagnosis of an ameloblastoma, which can display varying degrees of cellular differentiation.  Conclusion:  The presence of cystic spaces, follicular patterns, reverse polarity of nuclei, and a palisading layer of columnar or cuboidal cells at the periphery of the epithelial islands all point towards an ameloblastoma, a benign yet locally aggressive odontogenic tumor. |
| 15.47 | Diagnosis: Calcifying cystic odontogenic tumor/Calcifying odontogenic cyst. Key Microscopic Features: Cyst lined by basaloid cell lining with many cell-like structures; epithelial proliferation with many cell-like structures; lining epithelium containing few cell-like structures and slightly palisaded bottom layer cells; typical cell-like structures with eosinophilic cytoplasm and a space where the nucleus should be. | Based on the images provided, the lesion in question appears to be consistent with a Central Odontogenic Fibroma (COF), which is a benign tumor commonly found in the jaw. The analysis is based on the characteristic features seen in the different magnifications.  Key Microscopic Features Supporting the Diagnosis:  Fibrous Stroma:  The tumor displays a well-developed, fibrous stroma, which is characteristic of Central Odontogenic Fibroma. The stroma is composed of dense collagen fibers that appear pale pink in H&E staining.  Odontogenic Epithelium:  Small islands of odontogenic epithelium can be seen scattered within the fibrous stroma, with some showing ameloblast-like cells or basaloid epithelium. These features are indicative of odontogenic tumor processes.  Minimal to No Mineralized Material:  Unlike other odontogenic tumors, such as the odontoma, there is little to no evidence of mineralized tissue (like enamel or dentin), making COF distinct.  Lack of Inflammation:  There is a lack of significant inflammation, which can often be present in other odontogenic lesions like the odontogenic cyst or periapical granuloma.  Well-defined Borders:  The tumor has well-defined margins, indicating a benign nature, which is typical of COF. Malignant lesions tend to show more infiltrative or irregular borders.  Cellularity:  The lesion has relatively low cellularity, with a mix of fibroblasts and odontogenic epithelial elements embedded in the fibrous tissue.  These features are consistent with the Central Odontogenic Fibroma, which is typically well-circumscribed and characterized by fibrous tissue interspersed with odontogenic epithelium, without extensive mineralization. | Based on the images provided, I can offer an analysis of the histopathological features. Let's go step by step to assess these characteristics:  Diagnosis:  The images depict a lesion consistent with Adenomatoid Odontogenic Tumor (AOT).  Key Microscopic Features Justifying the Diagnosis:  Lesion Architecture:  The tumor exhibits well-circumscribed, cystic-like areas, which are often seen in AOTs. The cystic spaces may contain fluid and epithelial lining.  The Presence of Columnar Epithelial Cells:  One of the hallmark features of AOT is the presence of duct-like structures or columnar epithelial cells arranged in a rosette or tubular pattern, visible in some images. These cells resemble the cells of the odontogenic epithelium and are characteristic of AOT.  Pseudocyst Formation:  The images suggest the formation of pseudocystic spaces, commonly seen in AOT. These spaces are often lined by a layer of cuboidal or columnar epithelial cells and sometimes contain eosinophilic material.  Stromal Fibrosis:  The stroma around the epithelial cells is often fibrous, and this is evident in the images, where a fibrous connective tissue matrix surrounds the tumor islands, contributing to the lesion's characteristic appearance.  Calcifications or Dystrophic Calcification:  Although not universally present in every case, AOT often contains calcifications, which may be faintly seen in the images as areas of dense staining.  These features are diagnostic and point to AOT, a benign odontogenic tumor typically characterized by its distinctive architectural features and cellular patterns, which are visible in these images. |
| 15.48 | Diagnosis: Calcifying cystic odontogenic tumor/Calcifying odontogenic cyst. Key Microscopic Features: Cystic lesion with many cell-like structures and dystrophic calcifications; ameloblastomatous areas with basal cell nuclear palisading and stellate reticulum-like areas; juxtaepithelial dentinoid deposition; dentinoid encircling cell-like structures with early dystrophic calcifications. | Based on the examination of the provided hematoxylin and eosin (H&E) stained histopathological images of the same lesion at varying magnifications, the single most likely histopathological diagnosis is:  Ameloblastoma  Key Microscopic Features Supporting This Diagnosis:  Follicular and Plexiform Patterns (Image 1 & 2):  Islands and cords of odontogenic epithelium are evident within a fibrous connective tissue stroma.  The follicular areas show peripheral palisading of columnar cells with reverse nuclear polarity (nuclei away from the basement membrane), mimicking ameloblasts.  The centers of the epithelial islands contain loosely arranged, stellate reticulum-like cells.  Cystic Degeneration (Image 1 & 3):  Areas within the epithelial islands exhibit cystic changes, common in ameloblastoma, especially in follicular variants.  Some follicles appear to be breaking down centrally, forming microcystic spaces.  Palisading Basal Cell Layer (Image 2):  Clear palisading and hyperchromatic nuclei at the periphery of the epithelial islands.  Reverse polarization is a hallmark characteristic of ameloblastic epithelium.  Vascular and Stromal Features (Image 3 & 4):  The tumor stroma is mature and fibrous.  Some areas exhibit hemorrhage and eosinophilic coagulum, likely from cystic degeneration.  Image 4 demonstrates more acellular, hyalinized stroma and degenerating epithelial islands, consistent with longstanding ameloblastoma changes. | Histopathological diagnosis  Calcifying odontogenic cyst – “calcifying cystic odontogenic tumour” (Gorlin cyst)  Key microscopic features in the provided fields  Image Diagnostic findings How the finding supports the diagnosis  1 – low-power overview Irregular lobules and strands of odontogenic epithelium embedded in a collagenous fibrous wall; wide empty spaces are the cystic lumen. A cystic architecture with mural ameloblast-like epithelial proliferation is the expected overall pattern of a calcifying odontogenic cyst (COC).  2 – high-power of epithelial islands Follicular nests show: 1️⃣ a single layer of columnar/palisaded basal cells with hyperchromatic nuclei exhibiting reverse polarity, and 2️⃣ loosely arranged central cells resembling stellate reticulum. This ameloblastomatous epithelial phenotype is typical for the lining/proliferations seen in COC.  3 – cystic lumen A large cystic space lined by thin ameloblast-like epithelium; within the lumen and along the wall are eosinophilic masses and free epithelial clumps. COC is characteristically unicystic; intraluminal epithelial proliferations or ghost-cell masses are common.  4 – ghost cells & dentinoid Numerous pale eosinophilic, anucleate ghost cells; some are beginning to calcify. Adjacent is homogeneous eosinophilic dentinoid-like material and focal concentric (Liesegang-ring) calcifications. Ghost cells (with or without calcification) together with dentinoid deposition are the pathognomonic hallmarks of a calcifying odontogenic cyst.  Putting it all together  Cystic lesion with ameloblast-like lining ± intraluminal/mural proliferations  Ghost cells that frequently calcify  Associated dentinoid / calcified material in the capsule  Ameloblastomatous epithelial islands in the wall  This constellation is unique to the calcifying odontogenic cyst (calcifying cystic odontogenic tumour/Gorlin cyst) and distinguishes it from other odontogenic tumours such as conventional ameloblastoma, ameloblastic fibro-odontoma or CEOT. |
| 15.49 | Diagnosis: Dentinogenic ghost cell tumor. Key Microscopic Features: Tumor produces abundant dentinoid and infiltrates adjacent bone; tumor epithelium with ameloblastomatous appearance, palisading of basal cells and stellate reticulum-like areas; polygonal tumor cells transitioning into cells with eccentric nuclei and dentinoid deposition. Melanin is present. | Based on the provided H&E-stained histological images at increasing magnifications, the most appropriate histopathological diagnosis is:  Ameloblastoma  Key Microscopic Features Supporting the Diagnosis:  Follicular Pattern of Tumor Islands:  The tumor is composed of islands and nests of odontogenic epithelium, embedded in a fibrous stroma.  These islands show peripheral palisading of columnar cells with reverse polarization (nuclei displaced away from the basement membrane), resembling pre-ameloblasts.  Stellate Reticulum-Like Cells in the Center:  The central areas of the epithelial islands contain loosely arranged stellate or star-shaped cells, mimicking the stellate reticulum of the enamel organ.  Cystic Degeneration and Vacuolization:  Some epithelial islands exhibit central cystic or vacuolated degeneration, which is common in ameloblastomas.  No Evidence of Hard Tissue Formation:  Unlike odontomas or some variants of ameloblastic fibro-odontomas, there is no dental hard tissue (dentin or enamel matrix) formation visible in these sections.  Bone Invasion:  The images show tumor infiltration into bone, indicating the locally aggressive nature typical of ameloblastoma. | Histopathological diagnosis  → Dentinogenic ghost-cell tumour (solid variant of the calcifying odontogenic cyst)  Key microscopic features supporting this diagnosis  Field Findings in the submitted slides Diagnostic significance  Odontogenic epithelium Numerous solid nests, cords and sheets of ameloblast-like epithelium with a rim of tall columnar cells showing nuclear palisading and reverse polarity; centres contain looser, stellate-reticulum-type cells Typical epithelial component of ghost-cell lesions and distinguishes it from simple fibrous or reactive processes  Ghost cells Within many epithelial nests the central cells have lost nuclei, appear swollen, homogeneously eosinophilic and outline-preserved – classical ghost cells. Foci of dystrophic calcification are beginning within some of these areas Ghost cells are the hallmark of calcifying odontogenic lesions; their presence in abundance, in a predominantly solid tumour, favours a dentinogenic ghost-cell tumour rather than a purely cystic (“COC”) lesion  Dentinoid / osteoid-like matrix Broad, irregular masses of densely eosinophilic, acellular material lie immediately adjacent to, and sometimes engulf, the epithelial islands; the material shows tubule-like clefts and lacunae resembling dysplastic dentin Juxta-epithelial dentinoid deposition is characteristic of the dentinogenic variant and helps separate it from other ghost-cell entities and from ameloblastoma  Stroma Moderately cellular fibrous connective tissue with scattered spindle cells; no primitive myxoid stroma as would be expected in an ameloblastic fibroma/fibro-odontoma A relatively mature fibrous stroma is typical for DGCT  Calcification Small basophilic foci within ghost-cell clusters and within the dentinoid masses Frequent but not obligatory; supports a ghost-cell lesion  Taken together—the solid ameloblast-like epithelial component, the abundance of ghost cells, and the prominent production of dentinoid—these features are diagnostic of a dentinogenic ghost-cell tumour rather than an adenomatoid odontogenic tumour, ameloblastic fibro-odontoma, or other mixed odontogenic lesions. |
| 15.50 | Diagnosis: Ghost cell odontogenic carcinoma. Key Microscopic Features: Infiltrative cellular proliferation of basaloid cells with many cell-like structures; hypercellular and atypical odontogenic epithelium with palisaded bottom layer cell nuclei. | Histopathological Diagnosis:  Ameloblastoma (specifically, the acanthomatous subtype)  Key Microscopic Features Supporting the Diagnosis:  Follicular Architecture:  Seen in both images, especially the first: islands or nests of odontogenic epithelium in a fibrous connective tissue stroma.  Peripheral palisading of columnar cells resembling ameloblasts (hyperchromatic, reverse polarization of nuclei away from the basement membrane).  Central areas mimicking stellate reticulum (loosely arranged, star-shaped cells).  Acanthomatous Changes:  Prominent in both images but especially in the first one, where squamous metaplasia and keratin formation are evident within the epithelial islands.  Formation of keratin pearls, which is a hallmark of the acanthomatous ameloblastoma subtype. These are round, eosinophilic (pink-staining) concentric layers of keratinized cells.  Lack of Connective Tissue Invasion:  Although ameloblastoma is locally aggressive, it typically shows well-demarcated nests without stromal invasion into deeper tissues like a carcinoma would.  Absence of Ghost Cells or Dentinoid:  Helps rule out other odontogenic tumors like calcifying epithelial odontogenic tumor (CEOT) or calcifying odontogenic cyst (COC). | Histopathological diagnosis (single best):  Acanthomatous (keratinising) ameloblastoma – a squamous/keratin-rich variant of solid-multicystic ameloblastoma.  Key microscopic features in the two H&E sections that support this diagnosis  Feature How it appears in the images Significance  Islands & cords of odontogenic epithelium Numerous nests/sheets of basaloid cells infiltrating a fibrous stroma (left half of first image & left half of second image). Typical architectural pattern of solid ameloblastoma.  Peripheral ameloblast-like cell layer Rim of tall columnar cells showing nuclear palisading; nuclei are polarized away from the basement membrane toward the centre/lumen (best appreciated around the slit-like lumen in the second image). Hallmark of ameloblastoma.  Squamous (acanthomatous) metaplasia of the central cells Central portions of many tumour islands have converted to mature squamous cells with abundant eosinophilic cytoplasm (both images). Defines the acanthomatous variant.  Conspicuous keratin pearls / lamellated keratin masses Large, pink, concentrically layered keratin pearls occupying the centre of tumour islands and microcystic spaces (bold pink whorls in the right half of the first image and the circular whorl in the second image). Keratin-pearl formation is the key diagnostic clue for the keratinising/acanthomatous form.  Microcysts filled with keratin debris Pale eosinophilic, cyst-like spaces filled with keratin and ghost squames in the first image (bottom-left island). Common in keratin-rich ameloblastomas.  Lack of significant cytologic atypia or high mitotic activity Nuclei are fairly uniform; mitoses are rare. Supports a benign ameloblastoma rather than an ameloblastic carcinoma.  Taken together, the classic ameloblastic architecture combined with extensive squamous metaplasia and keratin-pearl production pinpoints the lesion as acanthomatous (keratinising) ameloblastoma within the spectrum of odontogenic tumours. |
| 15.51 | Diagnosis: Peripheral dentinogenic ghost cell tumor. Key Microscopic Features: Tumor in continuity with surface epithelium; epithelium resembling ameloblastoma with reverse nuclear polarization, stellate reticulum-like areas, and many cell-like structures; cell-like structures and dentinoid; dentinoid encircling cell-like structures and melanin present. | Based on the provided hematoxylin and eosin (H&E) stained histopathological images at various magnifications, the most likely diagnosis is:  Diagnosis:  Cemento-Ossifying Fibroma (COF)  Key Microscopic Features Supporting the Diagnosis:  Well-demarcated Lesion (Low Power View - Image 1):  The lesion is well-circumscribed and separated from the adjacent bone and soft tissue, which is a hallmark of a benign, encapsulated tumor.  The lesion is located beneath intact surface epithelium.  Fibrous Stroma (Images 2–4):  The tumor shows a cellular fibrous stroma composed of spindle-shaped fibroblastic cells.  The stroma appears moderately cellular, consistent with a fibro-osseous lesion.  Mineralized Components:  Cementum-like calcifications: Rounded, basophilic (purple-staining) structures that resemble cementum are scattered throughout the lesion.  Bone-like trabeculae: Eosinophilic, irregular bony trabeculae with osteocyte-like cells in lacunae are also seen (particularly in Images 3 and 4), supporting a mixed ossifying component.  These features are characteristic of a lesion with both ossifying and cementifying elements.  Lack of Cellular Atypia or Mitotic Activity:  No significant cytologic atypia or high mitotic rate is observed, which is consistent with a benign process. | Most likely histopathological diagnosis  → Cemento-ossifying fibroma (benign fibro-osseous / mesenchymal odontogenic tumour)  Key microscopic features that support this diagnosis  Field-of-view Salient findings Diagnostic significance  Low-power overview (Image 1) Sharply circumscribed, expansile mass immediately beneath an intact stratified squamous mucosa; a thin peripheral fibrous “capsule” separates the lesion from adjacent tissue. Well-demarcated border is characteristic of cemento-ossifying fibroma and helps distinguish it from fibrous dysplasia (which blends imperceptibly with surrounding bone).  Intermediate magnification (Image 2) Highly cellular, fibro-vascular stroma containing interlacing bundles of spindle cells; scattered trabeculae and irregular globules of eosinophilic calcified material are embedded within it. The biphasic pattern of fibrous tissue plus mineralised matrix is the defining feature of fibro-osseous lesions.  Higher magnification (Image 3) Calcified deposits show two patterns: ① curvilinear woven bone trabeculae with osteoblastic rimming and prominent reversal lines, and ② more basophilic, cementum-like spherical/ovoid masses (“cementicles”). Mixed bone- and cementum-type mineralisation is typical of a cemento-ossifying rather than a pure ossifying or cementifying variant. Reversal lines give the characteristic “mosaic” appearance.  Highest magnification (Image 4) The stromal spindle cells are bland, with scant cytoplasm and no cytologic atypia or increased mitoses; occasional dilated capillaries and focally extravasated red cells are present. Confirms the benign nature of the lesion and rules out malignant fibro-osseous tumours or sarcomas.  Synthesis of features  Well-circumscribed, capsule-like border separating the lesion from host bone.  Cellular fibrous stroma composed of uniform spindle fibroblasts.  Mixed mineralised product – interwoven trabeculae of woven bone and rounded cementum-like bodies, often with reversal lines.  Absence of significant atypia, mitoses or odontogenic epithelial component (excludes ameloblastic fibro-odontoma/complex odontoma).  Together these findings are diagnostic of a cemento-ossifying fibroma of the jaws, a benign odontogenic mesenchymal tumour that originates from periodontal ligament–derived cells capable of forming both bone and cementum. |
| 15.53 | Diagnosis: Primordial odontogenic tumor. Key Microscopic Features: Proliferation of spindle and stellate cells with mucinous stroma surfaced by columnar epithelial cells; epithelial component resembling ameloblastoma with stellate reticulum-like areas and basal cells with reverse nuclear polarization, with an underlying cambium layer of spindle and stellate cells; rudimentary organization into a tooth germ; nests of epithelial cells with clear cytoplasm and enameloid-like material; marked proliferation of epithelium forming cystic tooth germ-like structures with abundant eosinophilic enameloid; epithelium exhibiting palisaded nuclei with reverse nuclear polarization, cellular stroma, cambium zone, enamel matrix, enameloid, and dentinoid; interlacing trabeculae of epithelium encircling hypercellular stroma with plump fibroblasts. | Histopathological Diagnosis:  Ameloblastoma  Key Microscopic Features Supporting the Diagnosis:  The provided hematoxylin and eosin (H&E) stained images show classic features of ameloblastoma, a benign but locally aggressive odontogenic tumor. The diagnosis is based on the following key histological findings:  1. Follicular and Plexiform Patterns (Seen in Images 1, 2, 5, and 7):  Follicular Pattern: Islands or nests of odontogenic epithelium resembling enamel organ with:  Peripheral palisading of columnar ameloblast-like cells with reverse nuclear polarity (nuclei away from the basement membrane).  Central areas resembling stellate reticulum.  Plexiform Pattern: Interconnecting cords and strands of epithelium embedded in a fibrous stroma.  2. Cystic Changes (Image 3):  Large central cystic space lined by odontogenic epithelium, often seen in ameloblastoma variants (especially unicystic ameloblastoma).  Peripheral palisading and basal cell layer typical of ameloblastoma lining.  3. Mural Invasion (Image 4):  Tumor islands invading into the fibrous wall from the epithelial lining, consistent with a mural unicystic ameloblastoma, a variant of unicystic ameloblastoma with intramural proliferation.  4. Hypercellular Stroma and Vascularity (Image 6):  Evidence of loosely arranged mesenchymal tissue with prominent vascularity, sometimes showing hemorrhage, typical of ameloblastoma's stroma.  5. Absence of Hard Tissue Formation:  No evidence of dentin or enamel matrix, helping distinguish ameloblastoma from odontoma or ameloblastic fibro-odontoma. | Histopathological diagnosis  Adenomatoid odontogenic tumor (AOT) – most probably the extra-/intra-mucosal (extrafollicular) variant.  Key microscopic features in the supplied slides that support this diagnosis  Image (approx.) Salient finding Diagnostic significance  1 & 2 A well-delineated epithelial proliferation immediately beneath oral mucosa; cellular but bland spindle/oval odontogenic epithelial cells form solid sheets and whorls. AOT is a benign, encapsulated epithelial odontogenic tumour; the spindle-cell whorls are typical.  3 A unilocular, sharply circumscribed cystic space surrounded by a thick fibrous capsule with an inner rim of tumour epithelium. AOT is characteristically encapsulated and may partly or completely line a cystic cavity (follicular or extrafollicular pattern).  4 Numerous round to ovoid intensely eosinophilic “tumour droplets”/hyaline masses, many showing concentric dystrophic calcification (“snow-flake” calcifications), embedded within nests of tumour cells. The presence of eosinophilic amyloid-like material that often calcifies is virtually pathognomonic for AOT.  5 Low-power view of a lobulated lesion with multiple slit-like, gland-/duct-like spaces interspersed among solid epithelial nodules. The glandular/pseudoductal architecture gives the tumour its name (“adeno-”), differentiating it from ameloblastoma and other odontogenic tumours.  6 & 7 High-power views of the duct-like/papillary structures: columnar cells line a central lumen, nuclei are polarized away from the lumen; intervening stroma is scant and delicate. Polarized nuclei around duct-like spaces and papillary infoldings are classic for AOT; absence of cytologic atypia confirms benign nature.  Collectively, the combination of:  a well-encapsulated lesion,  spindle- and polygonal odontogenic epithelial cells arranged in solid whorls, rosettes and duct-like/tubular structures with nuclear polarization,  ample eosinophilic hyaline (“tumour droplet”) material with focal dystrophic calcifications,  and occasional cystic change  precisely matches the histologic picture of an Adenomatoid odontogenic tumor and rules out close mimics such as ameloblastoma, calcifying odontogenic cyst/tumor, or ameloblastic fibro-odontoma. |
| 15.55 | Diagnosis: Central cementoossifying fibroma. Key Microscopic Features: Proliferation of spindle cells with woven bone and cementum deposition; trabeculae of woven bone with osteocytes and acellular globular cementum droplets. | Based on the provided hematoxylin and eosin (H&E) stained histological images at different magnifications, the most likely diagnosis is:  Diagnosis: Ameloblastoma (conventional / solid multicystic type)  Key Histopathological Features:  1. Follicular Pattern (seen especially in higher magnification image):  Nests or islands of odontogenic epithelium.  Peripheral layer of columnar or palisaded ameloblast-like cells with reverse polarization (nuclei displaced away from the basement membrane).  Central areas resembling stellate reticulum (loose, star-shaped cells in a loosely arranged network).  2. Cystic Degeneration:  Evident in the lower magnification image as multiple cystic spaces within the lesion, which is typical of the solid/multicystic ameloblastoma.  3. Fibrous Stroma:  Moderately cellular connective tissue stroma separating epithelial islands.  4. No evidence of hard tissue formation (rules out odontoma, ameloblastic fibro-odontoma, etc.). | Histopathological diagnosis (single best fit)  Calcifying epithelial odontogenic tumor (Pindborg tumor)  Key microscopic features visible in the two fields  Feature How it appears in the provided images Diagnostic significance  Sheets / nests of epithelial cells The lesional tissue is densely cellular; individual tumour cells are polygonal-to-spindle with distinct borders and are arranged in solid sheets and whorled nests. (Best appreciated in the low-power overview.) An epithelial (rather than mesenchymal) odontogenic tumour.  Abundant extracellular eosinophilic, homogeneous (“amyloid-like”) material Large irregular pink blocks permeate the tumour islands (seen in both images, especially centre and bottom left of the high-power field). Characteristic amyloid-like product produced by CEOT cells, confirmable with Congo-red.  Concentric lamellated calcifications (Liesegang rings) A perfectly round, onion-skin calcification with a central nidus is present near the top of the high-power image; smaller rings are scattered elsewhere. Pathognomonic for CEOT; develop within the amyloid deposits.  Mild–moderate nuclear pleomorphism & prominent intercellular bridges Tumour cell nuclei vary slightly in size/shape; delicate bridges are visible between adjacent cells at high power. Typical cytology of CEOT; helps separate it from other epithelial odontogenic tumours.  Well-circumscribed border against native bone In the low-power view the tumour is cleanly demarcated from the subjacent mandibular bone by a fibrous capsule. CEOTs are usually well-outlined intrabony masses.  The combination of polyhedral odontogenic epithelium + abundant amyloid-like material + Liesegang ring calcifications is distinctive for a calcifying epithelial odontogenic tumour and is not reproduced by other lesions in the odontogenic-tumour group. |
| 15.56 | Diagnosis: Central cementoossifying fibroma and central giant cell granuloma. Key Microscopic Features: Proliferation of spindle cells with osteoid deposition and giant cell clusters; spindle cells with globular acellular cementum-like spherules and woven bone, hyalinized stroma; spindle and stellate cells depositing globular hematoxyphilic acellular cementum droplets and osteoid; presence of central giant cell granuloma. | Based on the histopathological features observed in the provided H&E-stained images of the same lesion at different magnifications, the most likely diagnosis is:  🦷 Calcifying Epithelial Odontogenic Tumor (CEOT)  (also known as Pindborg tumor)  🔍 Key Microscopic Features Supporting the Diagnosis:  Sheets and islands of polyhedral epithelial cells  These cells have well-defined borders and eosinophilic cytoplasm.  Nuclei often show pleomorphism but mitotic activity is usually low.  Prominent intercellular bridges  Characteristic of the epithelial component and visible in higher magnification (third image).  Liesegang ring calcifications  These are concentric, lamellated calcified structures, pathognomonic for CEOT.  Clearly seen in multiple images as eosinophilic, ring-like deposits (especially second and fourth images).  Amyloid-like eosinophilic extracellular material  Appears pink and amorphous, often in association with calcifications.  Congo red staining can confirm this material by showing apple-green birefringence under polarized light (not shown here but diagnostically relevant).  Fibrous stroma  Moderately cellular fibrous connective tissue stroma surrounds the epithelial islands.  🧠 Summary:  The combination of epithelial sheets, amyloid-like material, and Liesegang ring-type calcifications is diagnostic of Calcifying Epithelial Odontogenic Tumor (CEOT). This is a rare benign but locally aggressive odontogenic tumor that most commonly arises in the posterior mandible of adults. | Histopathological diagnosis –  Central (cemento-)ossifying fibroma – a benign fibro-osseous odontogenic tumour.  Key microscopic features that point to this diagnosis  Feature seen in the four H&E fields Why it favours a cemento-ossifying fibroma  Sharply circumscribed, expansile nodule composed of cellular fibrous tissue that is clearly separated from the surrounding bone (best appreciated in the lowest-power image) Ossifying fibroma grows as a well-demarcated, sometimes encapsulated mass, unlike the ill-defined borders of fibrous dysplasia.  Moderately cellular stroma of bland spindle/stellate fibroblasts with a whorled pattern Characteristic fibroblastic background of a fibro-osseous lesion.  Mixture of two types of mineralised product scattered throughout the stroma: 1) irregular trabeculae or plates of woven bone/osteoid (pink, rimmed by osteoblast-like cells) and 2) round to ovoid basophilic, acellular cementum-like spherules (“cementicles”) (purple-blue) – see the medium- and high-power images The coexistence of bony trabeculae and cementum-like globules is the signature microscopic pattern of a cemento-ossifying fibroma.  Reversal lines and occasional lamellation inside the calcified masses and plump rimming cells at their periphery (high power) Indicates active, neoplastic deposition of bone/cementum rather than inert dystrophic calcification.  Absence of sheets of polygonal epithelial cells or amyloid (rules out calcifying epithelial odontogenic tumour), and very scant or no odontogenic epithelium (rules out ameloblastic fibro-odontoma/central odontogenic fibroma) Helps narrow the differential to a purely mesenchymal fibro-osseous tumour.  Together, these findings—well-demarcated fibrocellular tissue containing both woven bone and cementum-like spherical calcifications—are diagnostic of a central (cemento-)ossifying fibroma. |
| 15.57 | Diagnosis: Cementoossifying fibroma. Key Microscopic Features: Proliferation of spindle and stellate cells in hyalinized collagenous stroma with few cementum spherules; benign spindle and stellate cells with cementicle deposition. | Based on the provided hematoxylin and eosin (H&E) stained histological images at different magnifications, the single most likely histopathological diagnosis is:  Desmoplastic Ameloblastoma  Key Microscopic Features Supporting the Diagnosis:  1. Dense Collagenized Stroma (Desmoplasia):  The stroma is highly collagenous and densely fibrous, which is a hallmark of desmoplastic ameloblastoma. This feature is prominently seen in both low- and high-power images as a pale pink background with scattered spindle cells.  2. Scattered Islands and Strands of Odontogenic Epithelium:  Small, irregular, compressed islands and strands of odontogenic epithelium are scattered within the dense stroma. These are more noticeable in the high-power image.  The epithelial cells may show peripheral nuclear palisading and reverse polarity (though subtle here), consistent with ameloblastic differentiation.  3. Lack of Classic Follicular or Plexiform Pattern:  Unlike conventional ameloblastomas, desmoplastic ameloblastomas lack large follicular structures. Instead, they present as small epithelial nests and cords embedded within the fibrotic stroma.  4. Compressed Appearance of Epithelial Islands:  The epithelial islands appear "squeezed" by the dense stroma, giving them a stellate or irregular configuration.  Summary:  These features—dense desmoplastic stroma, small epithelial islands or strands with odontogenic characteristics, and absence of classical ameloblastoma architecture—are consistent with Desmoplastic Ameloblastoma, a rare histological variant of ameloblastoma with a predilection for the anterior jaws and distinctive histology. | Histopathological diagnosis (single best):  Central odontogenic fibroma – simple (epithelium-poor) type  Key microscopic features that support this diagnosis  Low-power findings High-power findings Diagnostic relevance  Diffusely collagenised, pale-pink stroma forming broad, interlacing/whorled bundles Hypocellular lesion – only scattered bland, spindle- to stellate-shaped fibroblasts with wavy nuclei A mature, fibrous connective-tissue matrix with very low cellularity is characteristic of the “simple / epithelium-poor” variant of odontogenic fibroma  No obvious odontogenic hard tissues (no dentin, cementum or enamel matrix) and no bone trabeculae Absence of cytologic atypia, pleomorphism or mitotic activity Helps exclude ossifying fibroma, cemento-ossifying lesions and sarcomas  Only a few thin-walled vascular channels; lesion otherwise homogeneous No or only extremely rare rests of odontogenic epithelium (none seen in the fields provided) Distinguishes the simple form from the epithelium-rich (WHO-type) odontogenic fibroma and from mixed epithelial–mesenchymal tumours (e.g., ameloblastic fibroma)  Smooth pushing borders rather than infiltrative pattern (appreciated on low power) Collagen bundles separated by delicate myxoid clefts but not a predominantly myxoid matrix Separates it from odontogenic myxoma/fibromyxoma, which is far more gelatinous and basophilic  Taken together, the densely collagenised, hypocellular stroma with scattered innocuous fibroblasts and virtual absence of odontogenic epithelium or mineralised tissue conforms most closely to a central odontogenic fibroma of the simple / epithelium-poor subtype within the spectrum of odontogenic tumours of the jaws. |
| 15.57b | Diagnosis: Cementoossifying Fibroma; Key Microscopic Features: Cellular spindle cell proliferation with osteoid and cementicles deposition. Cementicles have a peripheral eosinophilic rim of Sharpey fibers. | Based on the provided hematoxylin and eosin (H&E) stained histological images of an odontogenic lesion at low and high magnification, the most likely diagnosis is:  Ameloblastic Fibro-Odontoma (AFO)  Key Microscopic Features Supporting the Diagnosis:  Biphasic Pattern:  The lesion shows both epithelial and mesenchymal components, a hallmark of mixed odontogenic tumors.  The epithelial component appears as odontogenic epithelial islands or cords with peripheral palisading, resembling ameloblastic epithelium.  The mesenchymal component resembles a primitive dental papilla, composed of loose, myxoid stroma with spindle-shaped cells.  Presence of Dental Hard Tissues:  There are eosinophilic, calcified materials resembling dentin and enamel matrix.  These areas often have tubular structures indicative of dentinoid, along with enamel matrix-like material which is less structured and more amorphous.  These features differentiate AFO from ameloblastic fibroma (which lacks calcified dental tissue).  Well-Circumscribed Appearance:  The low-power image suggests a relatively well-demarcated lesion, which is typical for AFO.  Differential Diagnosis Considerations:  Ameloblastic Fibroma: Lacks dental hard tissues.  Odontoma: More mature and disorganized calcified dental tissues without prominent soft tissue epithelial-mesenchymal interaction.  Ameloblastoma: Typically lacks the primitive mesenchyme and hard tissue formation seen in AFO. | Histopathological diagnosis (single best):  Conventional / central cemento-ossifying fibroma (benign mesenchymal odontogenic tumour)  Key microscopic features visible in the two H-E fields  Field Feature seen Significance for the diagnosis  Low-power (first image) • Sharply circumscribed mass composed of a moderately cellular fibrous stroma peppered with numerous, well-demarcated calcified islands of varying size and shape (ovoid, curvilinear, lobulated). A well-defined, tumour-like growth pattern is typical of a cemento-ossifying fibroma and helps separate it from the more diffuse fibro-osseous dysplasias.  • Calcified foci show two morphologies: (a) broad trabeculae that mimic woven/early lamellar bone, and (b) rounded spherules. The coexistence of bony trabeculae and cementum-like globules (“cementicles”) is characteristic; hence the combined term cemento-ossifying fibroma.  High-power (second image) • Rounded, intensely eosinophilic globules with faint concentric lamellation and occasional entrapped lacuna-like spaces—cementum-like deposits. These acellular cementicles are a hallmark of the lesion.  • Irregular, lightly basophilic/eosinophilic trabeculae containing osteocyte-like lacunae but showing little or no osteoblastic rimming—immature bone. Confirms the “ossifying” component.  • Intervening stroma formed by interlacing spindle-shaped fibroblasts and delicate collagen bundles; vascular but not markedly myxoid. Fits the classic fibro-osseous stroma of COF; cellularity is lower than in juvenile variants.  • No odontogenic epithelial islands, ghost cells, amyloid, or Liesegang-ring calcifications. Their absence helps exclude lesions such as ameloblastic fibro-odontoma, calcifying epithelial odontogenic tumour, and calcifying odontogenic cyst. |
| 15.61 | Diagnosis: Cementoosseous Dysplasia; Key Microscopic Features: Small bone fragments and cellular fibrous tissue surround a sclerotic cementoosseous mass. Spindle cells deposit woven bone trabeculae and hematoxyphilic cementicles. | Histopathological Diagnosis:  Ameloblastic Fibro-Ossifying Odontoma  Key Microscopic Features Supporting the Diagnosis:  Biphasic Tumor with Odontogenic Epithelium and Ectomesenchyme:  The presence of odontogenic epithelium arranged in islands, strands, and cords resembling the enamel organ (seen especially in higher magnification images).  Surrounding this epithelium is a primitive myxoid to cell-rich ectomesenchymal stroma mimicking the dental papilla.  Formation of Dental Hard Tissues:  Prominent areas of dentinoid and cementoid material are seen.  Multiple foci of immature woven bone and calcified trabeculae are evident, consistent with an ossifying component.  Some sections show dentin-like matrix formation within the connective tissue stroma.  No Cytologic Atypia or Malignant Features:  The cells are cytologically bland, without mitotic activity or nuclear pleomorphism.  Transition Zones:  There are gradual transitions from the cellular ectomesenchyme to areas of mineralized matrix (bone/dentin), a hallmark of odontogenic hamartomatous/benign tumor growth.  Organized Growth:  The tumor architecture, especially in low-power views, shows lobulated masses separated by fibrous septa, which is consistent with ameloblastic fibro-odontoma/fibro-ossifying lesions.  Rationale:  This lesion combines elements of:  Ameloblastic fibroma (epithelium + ectomesenchyme)  Ossifying odontoma (mineralized tissue: dentin, cementum, bone)  Hence, Ameloblastic Fibro-Ossifying Odontoma, a rare hybrid tumor in the odontogenic spectrum, best fits this constellation of histological features. | Histopathological diagnosis — Central cemento-ossifying fibroma (central ossifying / cemento-ossifying fibroma)  Key microscopic features that point to this diagnosis  Field What is seen in the photomicrographs Why it matters  Circumscription At low power the biopsy fragments are sharply demarcated from the surrounding decalcified bone and surrounded by a fibrous capsule-like edge A well-defined border is typical for a true neoplasm such as a cemento-ossifying fibroma and helps separate it from fibrous dysplasia, which merges imperceptibly with native bone  Fibro-cellular stroma The inter-trabecular background is highly cellular, made of plump spindle/stellate fibroblasts arranged in storiform and whorled patterns with delicate vascular channels A cellular, actively proliferating fibrous stroma is expected in this tumour and contrasts with the relatively hypocellular stroma of mature cemento-osseous dysplasia  Dual mineralised products Multiple types of calcified material are present: Irregular, curvilinear trabeculae and broad sheets of eosinophilic woven bone/osteoid with occasional osteoblastic rimming (pink) and rounded, basophilic acellular masses with reversal lines that resemble cementum (purple) The admixture of bone-like and cementum-like tissue is the hallmark of a cemento-ossifying fibroma; the components often coalesce as the lesion matures  Reversal / resting lines Dark, scalloped basophilic lines outline many calcified islands Indicates repeated deposition and resorption typical of a neoplastic cemento-osseous process  Absence of odontogenic epithelium No nests or sheets of ameloblastic or squamous epithelium are identified in any field Helps rule out mixed odontogenic tumours such as ameloblastic fibro-odontoma or calcifying epithelial odontogenic tumour  Lack of aggressive cytology Spindle cells show bland, uniform nuclei without atypia or elevated mitotic activity Favors a benign fibro-osseous neoplasm rather than an osteosarcoma or other malignancy  Taken together—a well-circumscribed, highly cellular fibrous lesion producing both bone- and cementum-like calcifications with characteristic reversal lines and no epithelial component—these findings are diagnostic of central cemento-ossifying fibroma, the prototypical benign fibro-osseous odontogenic tumour of periodontal-ligament origin. |
| 15.62 | Diagnosis: Cementoosseous Dysplasia; Key Microscopic Features: A long-standing lesion with a large sclerotic cementoosseous mass adjacent to lamellar cortical bone. Cellular spindle cells are next to dense cementum-like material. | Histopathological Diagnosis:  Cemento-Ossifying Fibroma (COF)  Key Microscopic Features Supporting the Diagnosis:  Well-Circumscribed Lesion (Low Power Image - Image 1):  The lesion appears well-demarcated from the surrounding bone, a classic feature of benign fibro-osseous lesions such as COF.  A fibrous capsule-like boundary can be seen, separating it from adjacent trabecular bone.  Fibrous Stroma (Medium Power - Image 2):  The stroma is composed of cellular fibrous connective tissue.  The presence of spindle-shaped fibroblasts in a collagenous background is evident.  Varying degrees of vascularity are present, typical of this lesion.  Mineralized Components (Higher Power - Image 3):  Numerous areas of mineralization are seen:  Cementum-like material: Round to ovoid basophilic acellular deposits.  Bone-like trabeculae: Some show osteoblastic rimming, confirming active bone formation.  The presence of both bone and cementum-like material is diagnostic for cemento-ossifying fibroma.  Lack of Atypia or Malignant Features:  No cellular atypia, mitotic activity, or necrosis is observed, consistent with a benign neoplasm.  Differential Diagnosis Considerations (and why they are less likely):  Fibrous Dysplasia: Typically lacks well-demarcated borders and shows more irregular, curvilinear ("Chinese character") bony trabeculae without osteoblastic rimming.  Cementoblastoma: Would show fusion with the root of a tooth, and usually more aggressive features.  Ossifying Fibroma: Cemento-ossifying fibroma is essentially a subtype of ossifying fibroma, showing both bone and cementum-like material, as seen here. | Histopathological diagnosis  → Benign cementoblastoma (“true cementoma”)  Microscopic features supporting this diagnosis  Field Key findings Why it points to cementoblastoma  Low-power architecture Well-circumscribed, lobulated lesion composed almost entirely of a solid, intensely eosinophilic calcified mass that is fused to the surrounding bone; a narrow fibro-osseous peripheral zone is present. A sharply defined, predominantly mineralised mass that blends with adjacent bone is typical of cementoblastoma rather than the more scattered calcifications of ossifying fibroma or cemento-osseous dysplasia.  Nature of the calcified tissue Broad, irregular sheets of mineralised material with deep basophilic reversal lines and occasional entrapped lacunae (cementocytes). The cementum-like quality (dense, basophilic reversal lines, cementocytes) distinguishes cementoblastoma from dentin-producing lesions such as odontogenic fibrodentinoma or fibro-odontoma.  Peripheral rimming cells A cuff of plump, basophilic cementoblast-like cells intimately lines the outer surface of the calcified mass; occasional multinucleated cells may be present. Active rimming by cementoblasts is a defining feature of cementoblastoma and is not seen in non-neoplastic fibro-osseous lesions.  Fibrovascular interface Vascular, cellular connective tissue wedges between the calcified lobules, containing numerous capillaries and scattered inflammatory cells without cytologic atypia. A fibrovascular stroma investing cementum-like trabeculae is expected in cementoblastoma; lack of atypia supports benign nature.  Relationship to tooth/root (Root not present in the section, but the peripheral fusion of the mass to pre-existing bone and its circumscription strongly suggest origin from the tooth’s periodontal ligament.) Cementoblastoma is characteristically fused to the tooth root; even when the root is not in the section, the pattern of attachment at the bony margin reflects this behaviour.  Diagnostic synthesis  The combination of a circumscribed lesion made up of dense cementum-like tissue with prominent reversal lines, actively rimming cementoblasts, and a vascular connective-tissue interface is classic for benign cementoblastoma and distinguishes it from other odontogenic and fibro-osseous tumours of the jaws. |
| 15.62b | Diagnosis: Cementoosseous Dysplasia; Key Microscopic Features: A long-standing lesion with a cementoosseous mass and cellular fibrous tissue. Osteoid and woven bone deposition are present, with occasional osteoclasts. | Based on the provided hematoxylin-and-eosin (H&E) stained histopathology images at low and high magnifications, the single most likely diagnosis is:  Central Ossifying Fibroma (COF)  Key Microscopic Features Supporting This Diagnosis:  1. Fibro-osseous architecture (seen in both images):  The lesion shows a well-demarcated border (especially visible at low power), which is characteristic of central ossifying fibroma.  There is a proliferation of fibrous connective tissue interspersed with irregularly shaped bony trabeculae or cementoid material, consistent with a fibro-osseous lesion.  2. Bone formation:  Trabeculae of woven bone or cementum-like spherules are present.  The bony trabeculae often have a mixture of immature and more mature appearance, sometimes rimmed by osteoblasts (though not prominent here).  3. Cellular stroma:  The stroma is moderately to highly cellular, composed of spindle-shaped fibroblasts in a collagenous background.  The cells show minimal atypia and no mitotic activity, consistent with a benign lesion.  4. Lack of features of malignancy:  There are no signs of malignant transformation such as cellular pleomorphism, high mitotic rate, or invasion into adjacent tissue.  Differential Diagnoses Considered but Less Likely:  Fibrous Dysplasia: Typically more poorly circumscribed and shows “Chinese character” woven bone; lacks clear border seen here.  Cemento-ossifying fibroma (variant): This term is now often unified under central ossifying fibroma, especially when both bone and cementum-like material are present.  Osteoblastoma or Cementoblastoma: These are more vascular and cellular with prominent osteoblastic rimming and sometimes aggressive growth.  Juvenile Ossifying Fibroma: More aggressive, occurs in younger individuals, and histologically shows more cellular stroma and mitotic figures. | Histopathological diagnosis (single best):  Central (cemento-)ossifying fibroma  Key microscopic features supporting this diagnosis  Field Findings visible in the images Significance  Overall architecture (low-power view) • Well-circumscribed, expansile lesion sharply demarcated from the surrounding native bone.  • Composed of a fibrous stroma that is peppered with numerous discrete, variably sized mineralised trabeculae. A sharply outlined, fibro-osseous mass is characteristic of an ossifying fibroma rather than a diffuse fibrous dysplasia.  Nature of the mineralised product • Irregular, curved trabeculae of woven bone / osteoid showing osteocyte lacunae.  • Occasional rounded or globular masses resembling immature cementum. An admixture of bone-like and cementum-like material is typical of a cemento-ossifying fibroma.  Osteoblastic activity • Many trabeculae are rimmed by a single layer of plump osteoblasts. Active deposition of new bone within a neoplastic fibro-osseous lesion.  Stromal component • Moderately cellular spindle-cell fibroblastic stroma with a whorled / storiform pattern.  • No significant cytologic atypia or mitotic activity. The bland but cellular fibrous tissue is a defining component; absence of atypia supports a benign lesion.  Interface with host bone • Abrupt transition between lesion and pre-existing cortical bone, without blending. A helpful feature that distinguishes ossifying fibroma from non-neoplastic fibro-osseous conditions.  Taken together, the constellation of a well-demarcated lesion composed of cellular fibrous tissue producing both woven bone and cementum-like material with osteoblastic rimming is diagnostic of a central (cemento-)ossifying fibroma, an odontogenic-origin benign fibro-osseous neoplasm. |
| 15.66 | Diagnosis: Odontogenic Myxoma; Key Microscopic Features: Proliferation of spindle cells in a myxoid-mucinous stroma with islands of odontogenic epithelium. Tumor cells may have trailing cytoplasmic processes and benign nuclei. | Based on the provided hematoxylin-and-eosin (H&E) stained images of the same lesion at different magnifications, the most likely histopathological diagnosis is:  Ameloblastoma (Follicular Type)  Key Microscopic Features Supporting the Diagnosis:  1. Islands of Odontogenic Epithelium:  Seen clearly in the second image (higher magnification), the lesion contains epithelial islands that resemble the enamel organ.  These islands are composed of a peripheral layer of columnar or palisaded ameloblast-like cells with reverse nuclear polarity (nuclei away from the basement membrane).  The center of the epithelial islands resembles the stellate reticulum of the enamel organ.  2. Mature Fibrous Stroma:  The epithelial islands are set in a mature, fibrous connective tissue stroma, as seen in the first and third images. This supports a benign but locally aggressive neoplasm rather than a malignant one.  3. Lack of Significant Atypia or Mitoses:  The cells appear relatively uniform without high mitotic activity or pleomorphism, typical of benign odontogenic tumors such as ameloblastoma.  4. Spindle Cell Stroma with Loose Arrangement (in some areas):  The fourth image shows a more loosely arranged connective tissue area with spindle-shaped cells, sometimes seen in the stromal component of ameloblastomas. | Histopathological diagnosis  Central odontogenic fibroma – WHO-type (epithelium-rich odontogenic fibroma)  Microscopic features that support the diagnosis  Image # Key findings Significance  1 (low-power overview) • Well-circumscribed, lobulated mass composed of sweeping, whorled bundles of pale-eosinophilic collagen with very low mitotic activity.  • Scattered, sharply demarcated dark-purple nests embedded in the stroma. A benign, fibrous odontogenic tumour with focal epithelial elements rather than an infiltrative ameloblastoma or a purely myxoid lesion.  2 (high power on one nest) • Small island of odontogenic epithelium: peripheral cuboidal/low-columnar cells and centrally looser, polygonal cells.  • No true palisading, reverse nuclear polarity, or stellate-reticulum-type spaces; nuclei bland, mitoses absent. Islands of “inactive” odontogenic epithelium are the hallmark of the WHO-type central odontogenic fibroma; lack of overt ameloblastic features helps exclude desmoplastic ameloblastoma.  3 (intermediate power) • Collagenous stroma is relatively cellular with evenly spaced, bland spindle or stellate fibroblasts.  • Mild focal myxoid change but no extensive gelatinous matrix. Dense collagen with only focal myxoid zones differentiates it from odontogenic myxoma/myxofibroma, which is far more myxoid and less collagenised.  4 (intermediate power) • Concentric and interlacing bundles of collagen create a storiform/whorled pattern.  • Cells are uniform, with inconspicuous nucleoli, and there is no atypia or necrosis. Confirms the benign fibroblastic nature of the tumour and helps rule out desmoplastic fibroma of bone (which is more infiltrative and lacks odontogenic epithelium).  Collectively, the combination of a mature fibro-collagenous stroma, scattered but unequivocal nests/cords of bland odontogenic epithelium, and the absence of enamel-organ–type palisading or extensive myxoid matrix is diagnostic of a central odontogenic fibroma, WHO-type. |
| 15.67 | Diagnosis: Odontogenic Myxoma with Dystrophic Calcifications; Key Microscopic Features: Tumor infiltrates bone and exhibits dystrophic calcifications. Spindle cells are embedded in collagenous and mucinous stroma. | Histopathological Analysis  Based on the provided hematoxylin and eosin (H&E) stained images, the single most likely histopathological diagnosis is:  🦷 Ameloblastic Fibro-Odontoma (AFO)  Justification & Key Microscopic Features  The diagnosis is supported by the following characteristic microscopic findings visible in the two images:  🔍 Low-Magnification Features (First Image):  Biphasic Tumor Composition:  Epithelial Component: Islands and cords of odontogenic epithelium resembling dental lamina or enamel organ.  Mesenchymal Component: Primitive, cellular ectomesenchymal tissue resembling dental papilla.  Odontoma-like Structures:  Presence of dentinoid and enamel matrix-like material.  These areas appear eosinophilic and acellular in varying degrees, consistent with developing dental hard tissues, including enamel and dentin.  🔬 High-Magnification Features (Second Image):  Epithelial Islands:  Peripheral palisading columnar cells with reverse nuclear polarity (resembling ameloblasts).  Central stellate reticulum-like cells, characteristic of ameloblastic epithelium.  Dental Hard Tissue Formation:  Clearly evident dentin-like matrix and enamel matrix, with adjacent ghost-like cells in enamel matrix areas.  Loose Mesenchyme:  The fibrous stroma appears primitive and loose, resembling dental papilla, consistent with the fibroblastic component of AFO.  ✅ Final Summary:  The presence of both ameloblastoma-like epithelial features and dental hard tissue formation (odontoma-like), within a primitive ectomesenchymal stroma, is the hallmark of Ameloblastic Fibro-Odontoma.  This entity is part of the spectrum of mixed odontogenic tumors and is most common in children and adolescents. | Histopathological diagnosis  Complex odontoma (a hamartomatous odontogenic tumour composed of a disorganised mass of dental hard tissues).  Key microscopic features supporting this diagnosis  Field finding How it appears in the two H-E micrographs Diagnostic significance  Irregular, haphazard masses of intensely eosinophilic hard tissue Large pink–purple islands and trabeculae occupy most of the low-power image; the material shows a smooth outer contour and swirling internal pattern typical of mature dentin Confirms a dental hard-tissue lesion rather than a purely epithelial tumour  Central “empty” or pale areas within the hard-tissue islands Many islands contain cleft-like white spaces where enamel has been dissolved out during decalcification Indicates the presence of enamel matrix, a prerequisite for an odontoma  Peripheral basophilic/blue granular rims on some islands A thin darker rim surrounds parts of several islands (best seen in the higher-power view) Represents cementum-like tissue, completing the tri-layered enamel–dentin–cementum structure characteristic of odontomas  Absence of organised tooth (denticle) morphology No distinct crown–root units or lamellar arrangement; instead a single amorphous block of mixed hard tissues Distinguishes a complex odontoma from a compound odontoma (which shows miniature teeth)  Sparse odontogenic epithelium entrapped at the periphery Small rosette-like epithelial nests adjacent to a dentin island in the high-power image Common but not essential; indicates odontogenic origin but the cellular component is minimal compared with mixed tumours such as ameloblastic fibro-odontoma  Fibrous capsule / follicular stroma enveloping the mass Pale, collagen-rich connective tissue surrounds and infiltrates between the calcified islands Expected in an odontoma and explains the circumscribed radiographic outline of the lesion  The combination of mature but disorganised dental hard tissues (dentin ± enamel ± cementum) without recognisable tooth forms and only scant epithelial/mesenchymal components is diagnostic of a complex odontoma. |
| 15.69 | Diagnosis: Central Odontogenic Fibroma; Key Microscopic Features: Proliferation of densely collagenous fibrous tissue with odontogenic rests. Epithelial rests show eosinophilic cytoplasm with hyaline globules. | Based on the provided hematoxylin and eosin (H&E) stained images of an odontogenic lesion at varying magnifications, the most likely histopathological diagnosis is:  Adenomatoid Odontogenic Tumor (AOT)  Key Microscopic Features Justifying the Diagnosis:  Spindle-shaped epithelial cells in a whorled pattern:  Seen across low-power images (first and third images).  These cells form sheets and strands, commonly in a fibrous stroma.  Duct-like or gland-like structures ("adenomatoid" appearance):  Evident in higher magnification views (second and fourth images).  Characterized by rosette-like or duct-like spaces lined by columnar or cuboidal cells with polarized nuclei.  Calcified deposits or eosinophilic amorphous material:  While not very prominent in the images provided, these are often seen in AOT and may appear as spherical calcifications.  Minimal cellular atypia and low mitotic activity:  Supporting a benign nature.  Summary of Diagnosis:  AOT is a benign epithelial odontogenic tumor, commonly affecting young individuals, and often associated with impacted teeth (particularly canines). The histologic hallmark is the presence of duct-like structures surrounded by odontogenic epithelium in a spindle cell background. | Histopathological diagnosis  Central (intra-osseous) odontogenic fibroma – WHO/epithelium-rich type  Key microscopic features visible in the photomicrographs  Feature What you can see in the provided fields  Fibro-cellular collagenous stroma The low-power views show a solid mass of interlacing and swirling bundles of bland spindle-shaped fibroblasts embedded in a pink, relatively mature collagen matrix. The pattern is storiform to fascicular rather than myxoid, supporting a fibroma rather than a myxoma.  Scattered odontogenic epithelial rests At higher magnification multiple, small islands, strands and rosette/“duct”-like clusters of basaloid epithelial cells are dispersed through the stroma. The cells have uniform, darkly staining nuclei, scant cytoplasm and no peripheral palisading or reverse polarity (i.e., they are “inactive-looking”), exactly as described for the WHO-type odontogenic fibroma.  Lack of ameloblastic or stellate-reticulum differentiation The epithelial nests do not show the tall columnar, polarized cells with central stellate reticulum typical of an ameloblastic fibroma, nor the solid lobular epithelial architecture of an adenomatoid odontogenic tumour, helping to exclude those entities.  Absence of significant mineralised material No woven bone trabeculae, cementum-like droplets or dystrophic calcifications are appreciable in these sections, which argues against ossifying/cemento-ossifying lesions and against the calcifying epithelial odontogenic tumour.  Bland cytology & low mitotic activity Both stromal and epithelial components are cytologically bland with no atypia or increased mitoses, in keeping with a benign fibrous odontogenic tumour.  These combined findings — a fibrous spindle-cell background riddled with innocuous odontogenic epithelial nests and occasional duct-like structures — are characteristic of a central odontogenic fibroma, epithelium-rich (WHO) variant. |
| 15.69b | Diagnosis: Central Odontogenic Fibroma; Key Microscopic Features: Tumor contains both small and large epithelial rests, with clear cells in the centers. | Histopathological Analysis of the Provided Images  Single Most Likely Diagnosis:  Desmoplastic Ameloblastoma  Key Microscopic Features Supporting the Diagnosis:  Desmoplastic Stroma:  The hallmark of desmoplastic ameloblastoma is a densely collagenized, fibrous (desmoplastic) stroma, which is prominently seen in both images.  The stroma appears eosinophilic (pink) and acellular with a compressed, fibrotic appearance, separating epithelial islands.  Compressed Epithelial Islands and Strands:  The tumor islands are small, irregular, and often appear compressed or “squeezed” by the surrounding dense stroma.  These islands contain cells resembling ameloblastic epithelium, though often lacking the classic palisading and reverse polarization seen in conventional ameloblastoma.  Scattered Follicular Patterns:  Some epithelial islands retain a vague follicular pattern with central stellate reticulum-like cells.  In the higher magnification image, you can see peripheral columnar or cuboidal cells with central areas containing loosely arranged cells.  Lack of Cyst Formation:  Unlike conventional follicular or plexiform ameloblastoma, this variant shows minimal to no cystic change.  Occasional Microcystic or Duct-like Spaces:  In some areas, you may observe small duct-like or microcystic spaces, a feature occasionally noted in desmoplastic ameloblastoma.  Summary:  The images demonstrate a tumor composed of irregular odontogenic epithelial islands in a prominent desmoplastic stroma, without classic follicular architecture or cystic spaces. These features are characteristic of desmoplastic ameloblastoma, a rare variant of ameloblastoma known for its unique histologic appearance and anatomic predilection for the anterior jaws. | Histopathological diagnosis – Desmoplastic ameloblastoma  (a desmoplastic/“collagen-rich” variant of conventional ameloblastoma)  Key microscopic features in the photographs that point to this diagnosis  Feature seen Diagnostic significance  1. Broad expanses of densely eosinophilic, paucicellular fibrous stroma (marked “desmoplasia”) Hallmark of the desmoplastic variant; the fibrous component dominates and compresses the epithelial element.  2. Numerous small, irregular islands, strands and cords of odontogenic epithelium scattered through the stroma Ameloblastomatous epithelial proliferation is present but greatly reduced in volume and distorted by the dense collagen.  3. Compressed nests show peripheral cuboidal/low-columnar cells with darker nuclei and pale rim (rudimentary palisading) Represents the attenuated ameloblast-like outer cell layer typical of ameloblastoma, though less conspicuous in this variant.  4. Central portions of the islands are solid or micro-cystic, sometimes with stellate-reticulum-like or squamoid cells and small lumina Internal architecture that separates ameloblastoma from purely fibrous odontogenic lesions.  5. Occasional thin, hyalinised basement-membrane–like rim around epithelial nests Often produced by the pressure of the collagenous stroma in desmoplastic ameloblastoma.  6. Absence of sheets of epithelial cells, amyloid, Liesegang rings or prominent calcifications Helps exclude calcifying epithelial odontogenic tumour, adenomatoid odontogenic tumour, etc.  7. No myxoid ground substance, but rather tightly packed collagen bundles swirling between nests Distinguishes it from an odontogenic myxoma, and from the more lax fibrous tissue of a central odontogenic fibroma.  The combination of dense “keloid-like” collagen interlaced with compressed, cystic/follicular islands of odontogenic epithelium is characteristic of desmoplastic ameloblastoma and not seen in other odontogenic tumours. |
| 15.69c | Diagnosis: Central Odontogenic Fibroma with Giant Cells; Key Microscopic Features: Features multinucleated osteoclast-type giant cells. There are also islands of odontogenic epithelium and a central giant cell lesion. | Single Most Likely Histopathological Diagnosis:  Ameloblastic Fibroma  Key Microscopic Features Supporting the Diagnosis:  The provided hematoxylin and eosin (H&E) stained histological sections (at low and higher magnifications) exhibit the classic features of Ameloblastic Fibroma, a benign mixed odontogenic tumor:  1. Biphasic Tumor Pattern:  Epithelial Component:  Presence of narrow cords and small islands of odontogenic epithelium resembling the dental lamina.  The epithelium has peripheral columnar or cuboidal cells, occasionally showing palisading, and central stellate reticulum-like areas, mimicking enamel organ epithelium.  Mesenchymal Component:  Composed of a cellular, primitive ectomesenchyme that resembles the dental papilla.  This stroma is loose and myxoid to fibroblastic in appearance, with plump, stellate or ovoid cells distributed in a delicate fibrous matrix.  2. Absence of Hard Tissue Formation:  Unlike lesions such as ameloblastic fibro-odontoma or odontoma, there is no evidence of enamel or dentin formation, helping rule out these other mixed tumors.  3. Well-Circumscribed Growth Pattern:  The tumor margins are generally well-demarcated, and the lesion appears non-infiltrative, consistent with benign behavior.  4. Lack of Cytologic Atypia or Mitoses:  Both epithelial and mesenchymal components appear cytologically benign, without mitotic activity or significant pleomorphism. | Histopathological diagnosis  Central odontogenic fibroma – “simple / epithelium-poor” subtype  Microscopic features that lead to this diagnosis  Image level What is seen Why it matters  Low-power overview (image 1) • Lobulated, relatively well-demarcated mass composed of pale-pink, collagen-rich tissue.  • The tumour is entirely mesenchymal; no obvious epithelial component or mineralised material is seen.  • Cellular density is moderate and fairly uniform throughout, arranged in sweeping bundles and whorled foci. Odontogenic myxoma would look much looser and basophilic (myxoid); ossifying-fibrous lesions would show woven bone or cementum-like calcifications. The collagen-rich, purely fibroblastic appearance narrows the differential to the simple variant of central odontogenic fibroma (COF) or a desmoplastic fibroma.  Intermediate / high power (image 2) • Spindle-shaped fibroblasts with slender, tapering nuclei arranged in short interlacing fascicles and storiform whorls.  • Scant cytoplasm, inconspicuous nucleoli, virtually no mitotic activity or atypia.  • Background of dense, eosinophilic collagen; focal myxoid change is minimal.  • Occasional thin-walled vessels and areas of haemorrhage; no calcified material.  • No infiltrative, “pushing-through-bone-trabeculae” pattern typical of desmoplastic fibroma is appreciable in the sampled tissue. These findings match the “epithelium-poor / simple” COF described in the WHO classification: a benign odontogenic tumour consisting of mature fibrous tissue with plump fibroblasts, arranged in fascicles or storiform sheets and lacking (or containing only scant) odontogenic epithelium and calcifications.  PMC  BioMed Central  Key points distinguishing COF from close mimics  Odontogenic myxoma / fibromyxoma – much looser, gelatinous, basophilic stroma with stellate cells.  Desmoplastic fibroma (aggressive fibromatosis of bone) – shows long sweeping fascicles that permeate and entrap native bone trabeculae; margins are typically infiltrative rather than lobulated as seen here.  ResearchGate  WHO-type (epithelium-rich) COF – would display conspicuous strands/nests of inactive odontogenic epithelium and often dysplastic dentin or cementum-like calcifications, none of which are present in the provided sections.  Taken together, the purely fibroblastic, collagen-rich lesion with benign cytology, storiform/fascicular architecture and absence of epithelium or mineralised material is most consistent with a central odontogenic fibroma, simple (epithelium-poor) variant. |
| 15.70 | Diagnosis: Central Odontogenic Fibroma, Amyloid-rich Variant; Key Microscopic Features: Densely collagenous fibrous tissue with small islands of odontogenic epithelium and amyloid globules. | Based on the histopathological features observed in the two hematoxylin and eosin (H&E) stained images at different magnifications, the single most likely diagnosis is:  Odontogenic Myxoma  Key Microscopic Features Supporting the Diagnosis:  Loose, Myxoid Stroma:  The lesion is composed of an abundant myxoid (mucoid) extracellular matrix with a pale, basophilic appearance under H&E stain, which is a hallmark of odontogenic myxoma.  Spindle-shaped and Stellate Cells:  Scattered within the myxoid background are spindle-shaped and stellate (star-shaped) fibroblastic cells with hyperchromatic nuclei, embedded in the loose connective tissue.  Lack of Significant Atypia or Mitoses:  The cells do not show marked atypia or significant mitotic activity, consistent with a benign lesion.  Minimal or Absent Epithelial Component:  No overt odontogenic epithelial rests or islands are seen in the provided fields, which is typical since odontogenic myxomas often lack an epithelial component or have only scattered remnants.  Vascularity and Fibrous Septae:  Some thin-walled blood vessels and fibrous septa are visible in the matrix, often organizing the tumor into lobules or strands. | Histopathological diagnosis (single best):  Central odontogenic fibroma – “simple / epithelium-poor” type  Microscopic features visible in the two H-E fields that support this diagnosis  Feature How it appears in the photographs Significance  Cell-rich fibrous stroma arranged in whorls and interlacing fascicles Both the low-power and high-power views show sweeping, wavy bundles of pale-pink collagen with spindle-shaped fibroblasts, creating a storiform / swirling architecture. Indicates a benign fibro-proliferative lesion rather than a myxomatous or epithelial tumor.  Plump, bland spindle or stellate fibroblasts Nuclei are elongated, evenly-chromatic, with inconspicuous nucleoli and virtually no mitotic activity. Fits the bland cytology expected in an odontogenic fibroma.  Rounded to ovoid cementoid / dentinoid calcifications (“cementicles”) scattered through the stroma Numerous homogeneous, pale‐purple to eosinophilic globules of varying size are embedded within the collagen bundles. Foci of cementum-like or dysplastic dentin are a classic, almost diagnostic, clue in central odontogenic fibroma.  Sparse, small clusters of odontogenic epithelium (inactive‐looking) Tiny nests/cords of basophilic cells are present in a few foci (best appreciated in the high-power image at the lower left and centre). They lack peripheral palisading or stellate reticulum. Even a scant epithelial component confirms that the fibrous lesion is of odontogenic origin.  Absence of features that would favour other entities No myxoid background (rules out odontogenic myxoma); no sheets of polygonal epithelial cells with amyloid (rules out CEOT); no bone trabeculae with osteoblastic rimming (rules out ossifying fibroma). Helps narrow the diagnosis to odontogenic fibroma.  Taken together—the highly cellular, whorled fibrous tissue, the scattered cementoid globules, and the presence (albeit minimal) of odontogenic epithelial rests—point squarely to a central odontogenic fibroma, simple/epithelium-poor type.s |
| 15.71 | Diagnosis: Granular cell odontogenic tumor; Key Microscopic Features: Clusters of granular cells are present alongside epithelial islands and globular calcifications within dense collagenous stroma. | Histopathological Diagnosis:  Ameloblastoma (specifically, the desmoplastic variant)  Key Microscopic Features Supporting the Diagnosis:  Desmoplastic Stroma:  All three images show a prominent dense collagenous (fibrous) stroma, which compresses the epithelial islands. This dense stroma is characteristic of the desmoplastic variant of ameloblastoma.  Compressed Odontogenic Epithelial Islands:  The epithelial islands are small, irregular, and scattered throughout the fibrous stroma.  They appear compressed and stretched, often lacking the classic peripheral palisading seen in conventional follicular ameloblastoma.  These islands often show a central area of loosely arranged cells, sometimes resembling stellate reticulum.  Epithelial Cell Morphology:  The epithelial cells are cuboidal to columnar, and although palisading is not prominent, they are still identifiable as odontogenic.  Some islands show squamoid appearance or cyst-like changes, which can occur in desmoplastic ameloblastomas.  Lack of Classic Ameloblastoma Features:  There is an absence of large cystic spaces and well-defined follicular or plexiform patterns, distinguishing it from other subtypes.  Instead, a more infiltrative growth pattern with narrow cords and strands of epithelium is seen. | Histopathological diagnosis  Desmoplastic ameloblastoma  (the “desmoplastic” or “collagen-rich” variant of ameloblastoma)  Key microscopic features in the slides that point to this diagnosis  Feature seen How it appears in the provided fields Diagnostic significance  Marked desmoplasia Vast areas of densely eosinophilic, hyalinised collagen that occupy more space than the epithelial component (pink background dominating every image). Hall-mark of the desmoplastic variant; conventional ameloblastoma shows only scant fibrous stroma.  Small, compressed odontogenic epithelial islands & strands Numerous irregular nests, cords and comma-shaped islands of basophilic cells are scattered singly within the collagen bundles rather than forming large follicles. The collagen “squeezes” the epithelium into these distinctive thin, distorted islands.  Peripheral cuboidal/low-columnar cells with minimal or absent palisading The edges of many epithelial islands show a rim of slightly darker nuclei, but without the neat tall palisading and reverse nuclear polarity of a typical follicular ameloblastoma. Loss of classic palisading is expected in the desmoplastic subtype.  Central cells showing squamoid / spindle / stellate-reticulum-like change and micro-cysts Many islands have pale central zones; some contain small lumina or cyst-like spaces filled with eosinophilic material. Micro-cyst formation and squamoid metaplasia are characteristic.  Foci of hyaline / calcified material Eosinophilic droplets and occasional basophilic calcified globules are present inside epithelial nests and lumina. Focal calcification and stromal hyalinisation are commonly seen in this variant.  Whorled pattern of stroma and epithelium At low power the lesion shows swirling, storiform arrangements of collagen with epithelial islands “trapped” within the whorls. Creates the distinctive low-power appearance that helps separate this entity from other odontogenic tumours.  Taken together—the overwhelmingly dense, whorled collagenous stroma, the numerous tiny distorted epithelial islands/cords with very subtle ameloblastic features, and scattered micro-cystic/hyaline foci—form the classic picture of desmoplastic ameloblastoma. |
| 15.72 | Diagnosis: Granular cell odontogenic tumor; Key Microscopic Features: Sheets of granular cells surround dentinoid, with some islands of odontogenic epithelium showing dystrophic calcifications. | Based on the three provided hematoxylin and eosin (H&E) stained histological images of the same lesion at different magnifications, the single most likely histopathological diagnosis is:  Clear Cell Odontogenic Carcinoma (CCOC)  Key Microscopic Features Justifying the Diagnosis:  Predominantly Clear Cells:  The tumor is composed mainly of cells with clear cytoplasm, which is a hallmark of CCOC.  The clear appearance is due to cytoplasmic glycogen (PAS-positive, diastase-labile), although this would require special stains for confirmation.  Polygonal Cell Shape with Distinct Cell Borders:  The tumor cells are polygonal to oval with well-demarcated borders and round to oval nuclei, many with prominent nucleoli.  Nuclear Pleomorphism and Hyperchromatism:  Some cells show nuclear pleomorphism and mild-to-moderate hyperchromatism, consistent with malignancy.  Hyalinized Stromal Areas and Vascular Invasion:  In the second image, there is prominent hyalinized fibrous stroma and tumor cells invading around blood vessels, supporting a malignant odontogenic tumor.  Duct-like or Glandular Structures and Clusters:  In the third image, areas suggestive of duct-like or pseudo-glandular structures are present, occasionally seen in CCOC.  The presence of clusters of clear cells and entrapped remnants of odontogenic epithelium is also noted.  Absence of Classic Ameloblastomatous Features:  Unlike ameloblastoma, there's no evidence of peripheral palisading, reverse polarity, or stellate reticulum-like areas.  This helps distinguish it from clear cell variants of ameloblastoma.  Differential Diagnosis to Consider (But Less Likely Here):  Clear Cell Ameloblastoma: Typically shows ameloblastomatous differentiation which is not evident here.  Metastatic Clear Cell Tumors (e.g., renal cell carcinoma): Need to be excluded clinically/radiologically.  Calcifying Epithelial Odontogenic Tumor (CEOT) with clear cells: Usually contains amyloid-like material and calcifications, not seen here. | Histopathological diagnosis  Calcifying epithelial odontogenic tumour (CEOT, “Pindborg tumour”)  Key microscopic features visible in the three H-E photomicrographs  Feature How it appears in the supplied images Diagnostic significance  Sheets/islands of polyhedral epithelial cells with distinct borders All three fields show tightly packed polygonal cells with crisp cell membranes. The tumour epithelium of CEOT is composed of polyhedral odontogenic cells rather than the columnar ameloblast-type cells seen in classic ameloblastoma.  Abundant granular, eosinophilic cytoplasm Cytoplasm is packed with fine eosinophilic granules, giving the cells a “sugar-coated” look (best appreciated in the first and third images). Granular cytoplasm (rich in lysosomes) is a hallmark of CEOT and helps separate it from AOT or clear-cell lesions.  Prominent intercellular bridges Delicate “spider-web” bridges can be traced between adjacent cells at higher power. Confirms squamoid/odontogenic epithelial nature of the cells.  Extracellular amyloid-like, eosinophilic hyaline material Second image shows large, irregular pools of pink, homogeneous material and smaller droplets dispersed among tumour cells. CEOT uniquely produces an odontogenic amyloid (odontogenic ameloblastic protein).  Calcifications forming concentric “Liesegang rings” Within the amyloid masses in the second and third images are round to ovoid calcified bodies, some showing concentric laminations. The progressive calcification of amyloid into Liesegang rings is virtually pathognomonic for CEOT.  Occasional clear cells/vacuolated foci Third image (right) demonstrates clusters of optically clear cells, reflecting cytoplasmic glycogen/degeneration. A recognised but variable component of CEOT.  The concurrence of polygonal granular epithelial cells, extracellular amyloid that undergoes concentric calcification, and characteristic Liesegang rings is diagnostic for a calcifying epithelial odontogenic tumour. |
| 15.73 | Diagnosis: Peripheral odontogenic fibroma; Key Microscopic Features: The stroma contains non-encapsulated proliferating spindle cells and calcifications, with odontogenic epithelium containing clear cells. | Histopathological Diagnosis:  Central Odontogenic Fibroma (COF) – WHO type.  Key Microscopic Features Justifying the Diagnosis:  1. Low-power View (First Image):  Well-circumscribed lesion expanding the jaw, without infiltrative borders.  Fibrous stroma with scattered epithelial islands/nests visible even at this magnification.  The lesion extends between bony trabeculae, indicating intraosseous origin.  2. Intermediate-power View (Second Image):  Interwoven collagenous stroma that is relatively cellular, with scattered odontogenic epithelial rests.  The epithelium appears inactive, non-keratinizing, and arranged in small nests or strands – a hallmark of odontogenic origin.  No features of atypia or malignant transformation are present.  Presence of dentinoid or cementoid material – these eosinophilic globules in the stroma suggest inductive interaction, supporting the diagnosis.  3. High-power View (Third Image):  Stroma is fibroblastic and contains numerous plump spindle-shaped fibroblasts.  Numerous odontogenic epithelial nests with uniform, basaloid to cuboidal cells.  Occasional calcifications and entrapped epithelial rests. | Histopathological diagnosis  Peripheral (WHO-type) odontogenic fibroma  Key microscopic features supporting the diagnosis  Image Findings Significance  Low-power (whole section) • Well-circumscribed, submucosal, dome-shaped mass beneath an intact, acanthotic stratified squamous mucosa with elongated rete ridges.  • Lesion composed predominantly of fibrous connective tissue of variable cellularity. A polypoid gingival tumour separated from surface epithelium is typical for the peripheral counterpart of odontogenic fibroma.  Intermediate magnification • Numerous, irregular globules and islands of intensely eosinophilic to basophilic calcified material (cementum-/dentin-like) scattered through the stroma. Odontogenic fibroma (WHO type) characteristically contains cementoid or dysplastic dentin deposits; they help distinguish it from purely fibrous reactive lesions.  High magnification • Small strands, cords and nests of inactive-appearing odontogenic epithelium with basophilic nuclei embedded in the fibrous matrix.  • Fibro-cellular stroma with plump spindle/stellate fibroblasts and delicate collagen bundles; occasional myxoid foci. The intimate admixture of odontogenic epithelium with a fibrous stroma is a defining feature of odontogenic fibroma. Presence of both epithelium and mineralised product rules out peripheral ossifying fibroma (reactive) and other odontogenic tumours such as ameloblastic fibroma.  Putting the features together  A gingival (extra-osseous) lesion showing a fibro-cellular connective-tissue proliferation that harbours inactive odontogenic epithelial rests and deposits of cementum-/dentin-like calcification is diagnostic of a peripheral odontogenic fibroma (WHO type). |
| 15.75 | Diagnosis: Cementoblastoma; Key Microscopic Features: The tumor's periphery shows parallel trabeculae of cementum, with large cementoblasts proliferating between these cementum seams. | Based on the histopathological analysis of the provided hematoxylin-and-eosin (H&E) stained images at different magnifications, the most likely diagnosis is:  Calcifying Epithelial Odontogenic Tumor (CEOT) — also known as Pindborg Tumor  Key Microscopic Features Supporting the Diagnosis:  Sheets and islands of polygonal epithelial cells:  The tumor is composed of polyhedral epithelial cells with well-defined borders.  Cells exhibit eosinophilic (pink-staining) cytoplasm and prominent intercellular bridges.  Pleomorphism and prominent nucleoli:  Nuclei may appear pleomorphic, with some atypical features, but mitoses are usually rare.  Nucleoli are often prominent in these cells.  Amyloid-like extracellular material:  There is abundant extracellular, homogeneous, eosinophilic material resembling amyloid, characteristic of CEOT.  This material is typically Congo red positive and shows apple-green birefringence under polarized light (not shown in H&E but implied by the eosinophilic deposits).  Liesegang ring calcifications:  Concentric, ring-like calcifications (Liesegang rings) are seen within the amyloid-like material.  These appear as round, basophilic calcifications scattered throughout the lesion.  Lobular growth pattern:  The tumor shows a lobular architecture with islands and sheets of tumor cells separated by fibrous septa, best appreciated in the lower magnification image. | Histopathological diagnosis  Complex odontoma (disorganized hamartomatous odontogenic hard-tissue lesion)  Key microscopic features supporting this diagnosis  Field What is seen Why it points to a complex odontoma  Low power (first image) A well–circumscribed, almost encapsulated mass composed predominantly of solid eosinophilic hard-tissue matrix with scattered islands of soft tissue A mature odontoma is essentially a tooth-like mass; complex type shows a single amorphous block rather than many miniature teeth  Hard-tissue matrix Broad areas of tubular dentin – pink, with parallel odontoblastic tubules cut longitudinally and transversely (second image) The presence of dentinal tubules confirms true dentin production rather than nonspecific calcification  Enamel component Numerous empty/cleft-like white spaces and basophilic outlines where enamel matrix was dissolved during decalcification (images 1–3) In decalcified H-E sections of odontomas the enamel matrix is lost, leaving these characteristic voids  Cementum Patchy basophilic globules apposed to dentin in some foci (image 3) Irregular cementum deposition is typical in complex odontomas  Odontogenic epithelium & pulp-like tissue Small nests/cords of odontogenic epithelial cells and loose fibro-vascular tissue interposed between hard tissues (images 1 & 3) Residual enamel organ / dental papilla components are obligatory in an odontoma  Lack of organisation into tooth-like structures No orderly crown-root morphology or separate denticles are present (contrast with compound odontoma) Confirms the complex subtype  Taken together, the disorganised admixture of dentin, enamel matrix voids and cementum without formation of miniature teeth, plus residual odontogenic epithelium, is diagnostic of a complex odontoma rather than other mixed odontogenic tumours such as ameloblastic fibro-odontoma, dentinogenic ghost-cell tumour or calcifying epithelial odontogenic tumour. |
| 15.76 | Diagnosis: Cementoblastoma; Key Microscopic Features: The tumor is attached to the tooth root, showing resorption, and has fused with the dentin; the encapsulated mass of cementum contains cementoblasts in an organized array at the periphery. | Histopathological Analysis of the Provided H&E Images  Single Most Likely Histopathological Diagnosis:  Ameloblastic Fibro-Osteoma (AFO)  Justification and Key Microscopic Features:  Across the four provided images, the lesion shows features diagnostic of ameloblastic fibro-osteoma, an intermediate lesion in the spectrum of odontogenic tumors, often considered a transitional form between ameloblastic fibroma and complex odontoma.  Key Features Identified:  1. Odontogenic Epithelium (Epithelial Component):  Present as nests and cords of odontogenic epithelium dispersed within the mesenchymal background.  These epithelial islands resemble the enamel organ with peripheral columnar/ameloblast-like cells and central stellate reticulum-like areas (better visualized at higher magnifications, particularly the last image).  2. Mesenchymal Component (Ectomesenchyme):  Composed of cell-rich, primitive, loose ectomesenchymal stroma, mimicking dental papilla.  The mesenchyme appears myxoid to cellular with fibroblastic features.  3. Hard Tissue Formation:  Prominent feature in this case:  Irregular woven bone or osteoid-like material is seen (visible clearly in images 1, 2, and 3).  Some areas show immature dentinoid or cementoid material.  The combination of epithelial, mesenchymal, and calcified tissue is a defining hallmark of ameloblastic fibro-osteoma.  4. Lack of Significant Maturation or Organization:  Unlike odontomas, where dental hard tissue is more organized into tooth-like structures, in AFO the bone/dentin is disorganized.  This supports a diagnosis of AFO over odontoma.  Differential Diagnoses Considered and Ruled Out:  Diagnosis Reason Ruled Out  Ameloblastic fibroma Lacks significant calcified tissue; this case shows abundant mineralized matrix.  Complex odontoma Would show more organized tooth-like structures; this lesion is immature and disorganized.  Odontogenic myxoma Lacks epithelial component and hard tissue formation.  Ossifying fibroma Typically lacks odontogenic epithelium. | Histopathological diagnosis – Complex odontoma  Microscopic clues that lead to this diagnosis  Field What you can see Why it matters  Low-power overview (Img 1) A well-circumscribed mass composed almost entirely of a pink, calcified tissue perforated by innumerable small round/elongate voids; surrounded by a thin fibrous capsule. A solitary, capsulated block of dental hard tissue favours an odontoma rather than an infiltrative true neoplasm.  Intermediate magnification (Img 2 & 3) The calcified matrix shows countless, evenly spaced parallel channels that run through the tissue. Adjacent to the mass is loose fibrous “pulp-like” tissue separated by a capsule. The channels are classic dentinal tubules – the hallmark of dentin. A pulp-like zone and capsule are typical of odontomas.  High magnification (Img 4) Tubular dentin with entrapped dentinal lacunae/canaliculi; occasional eosinophilic “ghost” spaces where enamel matrix was lost during decalcification; cementum-like basophilic lines rim some areas; no orderly crown-root architecture. A haphazard mixture of dentin (± cementum, lost enamel) without the anatomical organisation of a normal tooth identifies the lesion as a complex rather than a compound odontoma. Absence of odontogenic epithelium or primitive ectomesenchyme rules out ameloblastic fibro-odontoma or related tumours.  Key diagnostic features in summary  Dominant mass of tubular dentin – numerous dentinal tubules visible throughout the eosinophilic matrix.  Irregular, disorganised arrangement of dental hard tissues with no tooth-like shape (distinguishes it from compound odontoma).  Cleared “voids” where enamel matrix was present before decalcification.  Peripheral cementum-like deposits and a delicate fibrous capsule separating the lesion from surrounding bone/soft tissue.  Lack of invasive growth, atypia, or significant odontogenic epithelium, supporting a hamartomatous odontoma rather than an odontogenic neoplasm such as cementoblastoma or calcifying epithelial odontogenic tumour.  Taken together, these findings are classic for a complex odontoma – a hamartomatous odontogenic tumour composed of a chaotic mass of enamel, dentin and cementum with predominance of dentin. |

NONODONTOGENIC CYSTS, CYST-LIKE LESIONS, AND MYOSPHERULOSIS

| **Fig_no** | **Ground truth** | **GPT 4o answer** | **o3 answer** |
| --- | --- | --- | --- |
| 16.2 | Diagnosis: Nasopalatine duct cyst; Key Microscopic Features: The cyst lining is uniformly thin, with inflammation causing epithelial proliferation (indicated by arrows). The epithelium transitions to pseudostratified columnar, ciliated in some areas, and squamous in others. | Based on the histopathological images provided, they appear to show a non-odontogenic cyst or cyst-like lesion in the oral and maxillofacial region. The most likely diagnosis is Adenomatoid Odontogenic Tumor (AOT), which fits within the spectrum of non-odontogenic cysts and cyst-like lesions.  Key Microscopic Features:  Cystic Structure: The images show cyst-like areas with epithelial lining, which is characteristic of non-odontogenic cysts.  Epithelial Lining: In the second image, the epithelial lining is prominent, exhibiting a columnar or cuboidal appearance that is typical of certain odontogenic lesions.  Fibrous Capsule: The third image, marked with arrows, shows a thick fibrous capsule surrounding the cystic spaces, which is often seen in benign cysts and cyst-like lesions.  Cellular Arrangement: The cells within the lining appear somewhat organized with prominent nuclei and some degree of cellular stratification, which can be indicative of an odontogenic cyst or tumor.  Granulation Tissue/Inflammatory Cells: In some areas, inflammation or granulation tissue is seen, possibly due to irritation or secondary changes within the cystic space.  Given these features, Adenomatoid Odontogenic Tumor (AOT) is a strong candidate diagnosis. This lesion is often characterized by cystic structures with an epithelial lining that shows a range of cellular differentiation, and it often presents with a fibrous capsule and may be associated with a small degree of inflammation. | Most Likely Histopathological Diagnosis:  The lesion depicted in the images most likely represents Adenomatoid Odontogenic Cyst (AOC), a nonodontogenic cyst that is often found in the maxillofacial region.  Key Microscopic Features:  Cyst Lining:  The cyst is lined by columnar epithelial cells with areas of cuboidal epithelium. This epithelial lining is characteristic of adenomatoid odontogenic cysts.  Micropapillary Pattern:  There are areas showing papillary projections from the epithelial lining, which are indicative of an adenomatoid structure often seen in this type of cyst. This is consistent with the papillary or glandular appearance in some nonodontogenic cysts.  Resinous Materials:  Some regions show pink staining and dense collagen fibers, which could be suggestive of the fibrous capsule of the cyst.  Inflammation and Keratinization:  In certain images, there are signs of inflammatory cells in the surrounding tissue, which are typically seen in reactive processes of odontogenic or nonodontogenic cysts.  Cystic Spaces:  The cyst's central area appears hollow or fluid-filled, surrounded by the epithelial lining and some stroma.  Supporting Features:  The cyst shows well-defined boundaries, with epithelial proliferation that forms glandular-like structures, a hallmark of this type of lesion.  These microscopic characteristics help support the diagnosis of Adenomatoid Odontogenic Cyst. |
| 16.3 | Diagnosis: Nasopalatine duct cyst; Key Microscopic Features: The cyst lining is focally present with a thick basement membrane, and neurovascular bundles are seen in the wall (indicated by arrows). The epithelium is nonkeratinized and flattened. | Based on the provided hematoxylin-and-eosin (H&E) images of the lesion, let's proceed with the analysis.  Single Most Likely Histopathological Diagnosis:  The lesion most likely represents a Non-Odontogenic Cyst, possibly a Nasopalatine Duct Cyst (NPDC), given its location and appearance in the images.  Key Microscopic Features That Support the Diagnosis:  Cystic Structure: The lesion appears to be a cystic structure, with a well-defined, irregular lumen surrounded by a fibrous connective tissue capsule. This is consistent with non-odontogenic cysts.  Epithelium: The cyst lining consists of a layer of cuboidal to columnar epithelium. There may be focal areas of stratification or thickening, which are characteristic of cysts like the NPDC.  Inflammatory Cell Infiltrate: In some areas, there may be mild to moderate inflammatory infiltrate, which could include lymphocytes, plasma cells, and neutrophils. This is commonly seen in the lining of cysts and in reaction to either irritation or secondary infection.  Lumen: The cyst's lumen may contain a mix of necrotic debris, cellular components, and mucin. This suggests that the cyst is active, as opposed to a simple benign cyst that may be devoid of such materials.  Fibrous Capsule: A thick, dense fibrous capsule around the cyst, which is noted in these images, is typical for NPDCs and other non-odontogenic cysts.  Location: Given that this type of cyst can be found in the midline of the maxilla, often near the nasopalatine canal, the cyst's presentation on the images might correlate with this anatomical area.  Together, these histopathological features suggest a non-odontogenic cyst, with Nasopalatine Duct Cyst being the most likely diagnosis in this case. | The two images you provided show histological sections of a lesion, and based on the features observed, we can attempt a diagnosis.  Single most histopathological diagnosis:  The images likely represent a Radicular Cyst (Periapical Cyst), a common nonodontogenic cyst. This diagnosis is supported by the following key microscopic features:  Key Microscopic Features:  Cystic Structure:  Both images show well-defined, cystic spaces lined by epithelial cells. These cysts are typically filled with clear fluid or debris.  Epithelial Lining:  The cysts show a thin epithelial lining, which is a characteristic of radicular cysts. The lining may exhibit varying degrees of hyperplasia, often showing a non-keratinized squamous epithelium.  Inflammatory Cell Infiltrate:  The adjacent cystic area shows a chronic inflammatory infiltrate, including lymphocytes and plasma cells, which is common in radicular cysts, often associated with the root of a non-vital tooth.  Fibrous Capsule:  There is a fibrous capsule surrounding the cyst, indicative of a cystic structure that results from inflammation or necrosis due to a chronic periapical infection.  Peripheral Proliferation:  In some areas, you may observe areas where the epithelium shows reactive changes or thickening, which could suggest a reaction to chronic irritation.  Justification for Diagnosis:  The histopathological presentation of a cyst with an epithelial lining, surrounded by a fibrous capsule, and associated with chronic inflammation fits the characteristics of a radicular cyst. These cysts typically form as a result of inflammation associated with the non-vital tooth's root and can be seen radiographically as a radiolucent lesion at the apex of the tooth.  Let me know if you'd like any further analysis or if you would like to focus on a different aspect of the histopathology. |
| 16.6 | Diagnosis: Surgical ciliated cyst; Key Microscopic Features: The cyst is lined with uniformly thin epithelium, with mild chronic inflammation present in the wall. The epithelium consists of ciliated, pseudostratified columnar cells interspersed with mucous cells. | ased on the provided images, here's the analysis for the lesion in the context of nonodontogenic cysts, cyst-like lesions, and myospherulosis:  Single Most Histopathological Diagnosis:  Radicular Cyst (also known as Periapical Cyst)  Key Microscopic Features:  Cystic Lumen: Both images depict a cystic lesion, with a well-defined lumen filled with fluid or other contents.  Epithelial Lining: The cyst is lined by a non-keratinized stratified squamous epithelium (seen in both high and low magnification). This is characteristic of radicular cysts, which typically show a uniform layer of epithelial cells.  Fibrous Capsule: The outer wall of the cyst shows dense fibrous connective tissue, which is indicative of the capsule surrounding the lesion.  Chronic Inflammation: The connective tissue surrounding the cyst shows areas of chronic inflammation with plasma cells, lymphocytes, and histiocytes, common in radicular cysts due to their inflammatory origin.  Peripheral Palisading: The epithelium lining the cyst demonstrates palisading of the basal layer (which may be noted as a key feature of this lesion), contributing to the diagnosis of a radicular cyst.  Justification for Diagnosis:  Classic Histological Pattern: The radicular cyst is frequently seen at the apex of non-vital teeth, and its histopathology involves a cystic cavity lined by epithelium, with chronic inflammatory cells in the surrounding connective tissue.  Lesion Location: While the location of the lesion is not directly shown in the image, the morphology observed (epithelial lining, fibrous capsule) is consistent with radicular cysts seen in the oral cavity, which are a result of chronic periapical inflammation. | The images you've provided appear to show histopathological features of a lesion that may belong to the nonodontogenic cysts, cyst-like lesions, and myospherulosis group in oral and maxillofacial pathology.  Most Likely Diagnosis:  Given the appearance of the tissue in both images, the most likely histopathological diagnosis could be Adenomatoid Odontogenic Tumor (AOT) or a Non-odontogenic cyst like Nasopalatine duct cyst or Nasolabial cyst, which also fits the pattern of cystic structures with epithelial lining.  Key Microscopic Features:  Cystic Spaces:  Both images depict cyst-like areas surrounded by epithelial lining.  The first image shows a more organized structure with stratified epithelial lining (perhaps cuboidal or columnar).  The second image appears to show a more complex cystic structure with varying wall thickness.  Epithelial Lining:  The epithelial lining appears to be thin in some areas but is more organized and compact in others. This could be indicative of a non-odontogenic cyst or a benign neoplasm like an AOT, where the epithelial lining can vary in thickness.  Cellular Stroma:  The cyst walls in both images are accompanied by a stroma of mesenchymal-like tissue with possible focal areas of mild inflammation, which could point towards an inflammatory or reactive process.  In some regions, we can observe dense collagen or a fibrous background.  Absence of Dental Elements:  Notably, there is no clear evidence of tooth development or dental tissue, which would help distinguish an odontogenic lesion from a non-odontogenic lesion.  These features together suggest that the lesion might be one of the nonodontogenic cysts or a benign cystic tumor like AOT. The distinct epithelial lining and cystic presentation are important markers to support this diagnosis.  If more detailed features are needed, reviewing additional diagnostic information or images from other angles could help further clarify the exact diagnosis. |
| 16.8 | Diagnosis: Simple bone cyst; Key Microscopic Features: Small fibrovascular tissue and bone fragments are curetted from the empty cyst cavity during surgery. The walls contain fibrovascular tissue and viable bone. | From the images provided, we can observe the histopathological features characteristic of a non-odontogenic cyst, likely a nasopalatine duct cyst (NPDC), also known as an incisive canal cyst.  Key Histopathological Features:  Cystic spaces: Both images show distinct cystic spaces that are surrounded by a fibrous wall, which is typical of non-odontogenic cysts like NPDC.  Epithelial lining: The cysts in these images exhibit epithelial linings with both columnar and squamous cells. This lining is a key feature of NPDC, as it often displays varying types of epithelium in different areas.  Fibrous connective tissue: The surrounding connective tissue is well-developed, often observed with blood vessels and a fibrovascular stroma, contributing to the appearance of a cyst with a well-defined wall.  Chronic inflammatory cells: Inflammatory cells, especially in the deeper portions of the wall, are visible in the second image. This is common in NPDC due to chronic irritation or infection.  Smooth and rounded cyst margins: The smooth contours of the cysts and the arrangement of tissue seen in these high magnifications are indicative of a benign, non-invasive cystic lesion.  Based on these features, nasopalatine duct cyst (NPDC) appears to be the most likely diagnosis. | Based on the two provided hematoxylin-and-eosin (H&E) images of a lesion within the category of nonodontogenic cysts, cyst-like lesions, and myospherulosis in oral and maxillofacial pathology, I will analyze the key features and histopathological diagnosis.  Single Most Histopathological Diagnosis:  Peripheral Ossifying Fibroma (POF) or Peripheral Cementifying Fibroma (PCF)  Key Microscopic Features Justifying the Diagnosis:  Lesion Morphology:  The first image displays a lesion with varying cellular architecture, including areas of abundant fibrous tissue and scattered bone or calcified material. This is characteristic of peripheral ossifying or cementifying fibromas.  The second image reveals more defined bone or cementum-like structures embedded within the fibrous tissue, indicative of ossifying or cementifying processes.  Fibrous Stroma:  Both images show a prominent fibrous stroma. The fibrous tissue is generally loose and appears to interlace around the calcified areas. This is a hallmark of peripheral fibromas (whether ossifying or cementifying).  Calcified Components:  The second image shows areas of mineralization that resemble bone or cementum-like material. These calcified components can range from small bone trabeculae to cementum-like deposits, a typical feature in POF and PCF.  Cellular Elements:  The presence of mesenchymal cells, often resembling fibroblasts, scattered within the fibrous stroma, as seen in both images, supports a fibrous lesion, reinforcing the diagnosis of peripheral fibromas.  Inflammatory Cells:  There might be scattered inflammatory cells, though this is not as prominent in the provided images, which can still be a feature in these lesions, particularly if they are irritated or secondary to trauma. |
| 16.9 | Diagnosis: Simple bone cyst associated with cementoosseous dysplasia; Key Microscopic Features: The cyst is surrounded by fibrovascular tissue with a proliferation of spindle cells. Cementoosseous material forms globular masses within the cyst-like space. Spindle cell proliferation includes the deposition of woven bone and cementum droplets (cementicles). | The provided images show histopathological sections of a lesion that likely falls within the group of nonodontogenic cysts, cyst-like lesions, or myospherulosis.  Single Most Likely Histopathological Diagnosis:  Calcifying Epithelial Odontogenic Cyst (CEOC) / Pindborg Tumor  Key Microscopic Features:  Cystic Spaces and Calcifications:  In several images, there are areas with cystic spaces filled with amorphous material and calcified deposits. The calcification seen in these images is a characteristic feature of this type of lesion.  Palpable Epithelial Lining:  The cystic spaces are lined with epithelium showing features like squamous differentiation, which is often observed in these types of lesions. The epithelial lining appears somewhat thickened in certain areas.  Amorphous Material and Hyaline Appearance:  The lesion shows areas of hyaline and amorphous material, which might resemble amyloid-like material and are commonly present in calcifying epithelial odontogenic cysts.  Odontogenic Epithelium Features:  There are islands of odontogenic epithelium and ghost cells (cells that have lost their cytoplasm, leaving only their outlines), which are hallmarks in such lesions.  Fibrous Stroma with Calcifications:  The stroma surrounding the cysts is fibrous and exhibits some calcified structures, often found in calcifying odontogenic cysts and related lesions.  The microscopic features observed across the various magnifications support the diagnosis of a Calcifying Epithelial Odontogenic Cyst (CEOC), primarily due to the presence of calcifications, cystic structures, and specific epithelial changes. | Based on the histopathological images provided, here's an analysis of the lesion:  Single Most Likely Histopathological Diagnosis:  The lesion likely corresponds to a globulomaxillary cyst or periapical cyst, which are both examples of nonodontogenic cysts. These cysts are not derived from dental tissue, but can occur in the maxillary region. The features visible in the histopathology are consistent with a cystic lesion with specific microscopic characteristics.  Key Microscopic Features:  Cystic Architecture:  The images show a cystic structure with a well-defined lining.  In several images, there is the presence of a thick fibrous capsule surrounding the cyst.  Epithelial Lining:  The cyst lining exhibits stratified squamous epithelium, which is characteristic of these types of cysts.  In some sections, the epithelium appears thin and flattened, without significant hyperplasia, which is often seen in the lining of nonodontogenic cysts.  Inflammatory Cells:  Inflammatory infiltrates can be seen around the cyst wall in the form of lymphocytes, plasma cells, and histiocytes, suggesting chronic inflammation.  The presence of these cells is typical for cysts that are of a nonodontogenic origin.  Bone and Fibrous Tissue:  The surrounding areas show fibrous connective tissue and scattered bone-like structures or areas of bone resorption.  This could be related to the cyst’s interaction with the surrounding bone, which may show reactive changes.  Fluid-filled Spaces:  Some of the cystic spaces appear to have clear or homogenous material, possibly indicating a serous or mucoid fluid component. |
| 16.11 | Diagnosis: Hematopoietic bone marrow defect; Key Microscopic Features: The bone fragments are viable and include hematopoietic marrow. Megakaryocytes, erythrocyte precursors, and myeloid precursors are observed. | Based on the provided hematoxylin-and-eosin images of the lesion in the context of nonodontogenic cysts, cyst-like lesions, and myospherulosis group in oral and maxillofacial pathology, here is the analysis:  Single Most Histopathological Diagnosis:  The most likely diagnosis for the lesion in these images is a Buccal Bifurcation Cyst (BBC), a type of non-odontogenic cyst commonly seen in the mandible, especially around the first permanent molar.  Key Microscopic Features:  Lesion Location:  The lesion appears to be in the vicinity of bone, with features suggestive of cystic change near the tooth, often associated with the molar. In the higher magnification, the lesion's proximity to the bone and tooth surfaces is visible.  Cystic Spaces:  The presence of multiple large cystic spaces within the lesion, as seen in the images, is a characteristic feature. The cysts are lined by a single layer of epithelial cells.  Inflammation and Granulation Tissue:  There is abundant chronic inflammatory infiltrate within the cystic lining, consistent with a reactive process. This infiltrate is largely composed of lymphocytes, plasma cells, and histiocytes, a feature commonly found in nonodontogenic cysts.  Epithelial Lining:  The cysts are lined by a non-keratinized epithelium, often columnar or cuboidal, which can exhibit areas of hyperplasia. This feature helps differentiate this lesion from odontogenic cysts like the dentigerous cyst.  Bone Resorption:  At the lower magnification, there is evidence of bone resorption, a key feature in the presence of a cystic lesion that is in close proximity to the bone, leading to cortical thinning or even perforation.  Microscopic Lymphoid Follicles:  In certain sections, the presence of lymphoid follicles and a mixed inflammatory infiltrate suggests the lesion is reactive and further points to the inflammatory origin of the cyst. This can be a hallmark of BBC, which is often associated with reactive changes due to the inflammatory environment.  These histopathological features align with the diagnosis of a Buccal Bifurcation Cyst and help in differentiating it from other cystic lesions in the oral cavity. | Based on the provided histopathological images of the lesion, the diagnosis appears to most likely be a myospherulosis, which falls under the group of nonodontogenic cysts and cyst-like lesions.  Key microscopic features justifying this diagnosis:  Presence of foamy, vacuolated cells: The first image shows areas with large clear spaces (fat-like structures) and clusters of cells that have a foamy appearance, indicating fat or lipid-laden cells. These foamy cells are characteristic of myospherulosis.  Peripherally placed small rounded structures: The second image zooms in on the cells and demonstrates these fat-filled structures surrounded by densely packed cellular material. The rounded, centrally clear vacuoles, which appear like empty spaces (fat cells), are a hallmark feature of this condition.  Dense inflammatory infiltrate: Both images show a mix of inflammatory cells, primarily lymphocytes and plasma cells, within a fibrous stroma. This pattern is also consistent with myospherulosis, which is known to be associated with chronic inflammation.  Bone and fibrous tissue involvement: The first image includes areas of bone-like tissue at the periphery, which is a common finding when myospherulosis occurs in close proximity to bone, often resulting from a traumatic injury.  Together, these microscopic features support a diagnosis of myospherulosis, characterized by lipid-laden cells (resembling fat or vacuolated structures), chronic inflammation, and the potential for bone involvement or adjacency. |
| 16.15 | Diagnosis: Myospherulosis; Key Microscopic Features: Clear spaces contain yellow-brown particulate material surrounded by a histiocytic and giant cell reaction, with fibrous and hyalinized stroma. Spherical and crescent-shaped bodies, along with smaller fragments of similarly pigmented debris, are present. Clear spaces of various sizes contain yellow-brown material with marked fibrosis and mild chronic inflammation. One spherical brown body shows an endobody (indicated by an arrow). | Based on the provided hematoxylin and eosin-stained images of the lesion, the histopathological features suggest a diagnosis of myospherulosis, which is a cyst-like lesion associated with the presence of myospherules, or foreign body granulomas.  Key Microscopic Features:  Presence of Myospherules:  The most characteristic feature is the presence of myospherules, which are round, clear spaces surrounded by a collection of macrophages and foreign-body giant cells.  These structures are seen in multiple images, often surrounded by fibrosis.  Foreign Body Reaction:  The lesion exhibits signs of a foreign body reaction, indicated by the presence of multinucleated giant cells and inflammatory cells surrounding the myospherules.  Cyst-Like Spaces:  The cystic spaces are outlined with dense fibrous tissue and are observed in several images. These spaces may contain cellular debris, and their walls are often reactive.  Fibrosis and Inflammation:  Surrounding the cystic spaces are areas of fibrosis and chronic inflammation, with scattered lymphocytes and plasma cells. The reactive tissue can cause the lesion to appear multinodular.  These findings collectively suggest myospherulosis, which is often associated with the use of certain foreign substances in the oral cavity and can resemble a benign cystic lesion, but with distinct histopathological features. | Based on the images provided and the histopathological features presented, the diagnosis points toward Myospherulosis, a cyst-like lesion. Myospherulosis is characterized by the presence of spherical or globular aggregates of foreign material in tissue, and the lesions typically show the following histopathological features:  Key Microscopic Features Justifying the Diagnosis:  Spherical Collections of Foreign Material:  The images display spherical or oval bodies, predominantly at higher magnification, surrounded by a fibro-inflammatory reaction. These entities appear as "spherules" or "myospherules," which are a hallmark of myospherulosis. They contain variable material, often including hemosiderin (iron), which is seen as dark brown pigmentation.  Granulomatous Inflammation:  There is a marked inflammatory response surrounding these spherules, which is often granulomatous in nature, indicated by a mixed infiltrate of macrophages and multinucleated giant cells. This is evident in the second image, where multinucleated giant cells can be seen surrounding the spherules.  Hemosiderin Deposition:  Hemosiderin-laden macrophages (brownish pigment) are present, suggesting previous hemorrhage or breakdown of blood products within the lesion. This is seen in the second and fourth images.  Fibrous Tissue and Cyst-like Structures:  The lesion is encased in fibrous tissue, which is characteristic of cyst-like lesions, as shown in the first and third images, where the fibrous capsule surrounding the spherules is evident.  Histological Arrangement:  The spherules are often separated by a well-defined fibrous tissue stroma, creating a multilocular or cystic appearance. This arrangement is visible in the first image, where the spherules are separated by the fibrous matrix.  These histopathological findings strongly support the diagnosis of Myospherulosis, a benign condition often seen in the oral and maxillofacial region following trauma or foreign body implantation. |

NONODONTOGENIC INTRAOSSEOUS TUMORS AND INFLAMMATORY/REACTIVE CONDITIONS

| **Fig_no** | **Ground truth** | **GPT 4o answer** | **o3 answer** |
| --- | --- | --- | --- |
| **17.7** | Diagnosis: Primary nonbacterial sclerosing osteomyelitis; Key Microscopic Features: Interlacing trabeculae of bone with medullary fibrosis; Parallel seams of woven bone with some osteoblastic rimming and abundant fibrous tissue with peritrabecular clefting suggestive of fibrous dysplasia; Areas exhibit prominent fibrosis with acute and chronic inflammation; Note the areas of delicate fibrous tissue and osteoid with irregular bone fragments may be mistaken for fibrous dysplasia in a small biopsy; Atypical reactive bone with plump osteocytes is often present. | Central Ossifying Fibroma (COF)  Key Microscopic Features Supporting the Diagnosis:  Well-demarcated lesion:  The lesion shows a clear demarcation from the surrounding bone, which is characteristic of a neoplastic process such as a benign fibro-osseous lesion.  Fibrocellular stroma:  The background stroma is moderately to highly cellular with spindle-shaped fibroblasts in a whorled or storiform pattern.  Presence of mineralized material:  There are numerous trabeculae and spherical structures of mineralized tissue. Two types of mineralization are seen:  Trabecular woven or lamellar bone (some with osteoblastic rimming).  Cementum-like basophilic spherules (cementoid calcifications).  Lack of significant inflammation or atypia:  No features of malignancy (e.g., atypical mitotic figures, pleomorphism) or overt inflammation dominate the fields.  Pattern of mineralization:  The mineralized material appears embedded in the fibroblastic stroma and is distributed in a somewhat concentric or peripheral pattern, consistent with ossifying fibroma rather than other fibro-osseous lesions.  Differential Diagnostic Considerations (but ruled out based on features):  Fibrous Dysplasia:  Usually not well-demarcated and blends with surrounding bone; lacks capsule-like border.  Bony trabeculae are typically irregular and "Chinese character" shaped without osteoblastic rimming.  Cemento-Osseous Dysplasia:  Often occurs in tooth-bearing areas, especially the anterior mandible.  Usually multifocal and not as well-demarcated; less cellular stroma.  Osteoblastoma or Cementoblastoma:  These show more prominent osteoblastic or cementoblastic rimming with a different growth pattern and clinical correlation. | Histopathological diagnosis (single best fit)  Juvenile trabecular ossifying fibroma (JTOF) — the trabecular (non-psammomatoid) variant of juvenile ossifying fibroma, a benign but locally aggressive fibro-osseous neoplasm of the jaws.  Key microscopic features supporting this diagnosis  Field Findings visible in the submitted H&E sections  Overall architecture • Well-circumscribed, expansile lesion composed of innumerable interlacing, slender trabeculae of osteoid / woven bone that branch and anastomose, creating a lace-like pattern.  • Trabeculae are evenly distributed throughout a cellular fibrous stroma, producing the striking “fish-net” appearance seen at low power.  Nature of the bony trabeculae • Trabeculae are long, curved or angular (“Chinese-character”) strands rather than spherical bodies.  • Prominent osteoblastic rimming with plump active osteoblasts lines many trabeculae, confirming newly formed woven bone.  • Occasional transition to more mature lamellar bone at the periphery.  Stromal component • Inter-trabecular spaces contain a highly cellular fibroblastic stroma of uniform spindle cells with scant collagen.  • No atypia or increased mitoses.  Absence of other elements • No odontogenic epithelium, cementicles, or psammoma-like calcified bodies (helps exclude odontogenic tumors and the psammomatoid JO variant).  Circumscription • Sharp demarcation from surrounding native bone (best appreciated on the low-power image), a feature typical of ossifying fibroma and unlike fibrous dysplasia.  Together, the combination of a sharply circumscribed lesion, a very cellular fibrous stroma, and numerous elongated, osteoblast-rimmed woven-bone trabeculae is characteristic of juvenile trabecular ossifying fibroma. |
| **17.8** | Diagnosis: Medication-related osteonecrosis; Key Microscopic Features: Nonviable bony sequestrum (dislodged by nonsurgical sequestrectomy) with ragged resorptive defects surrounded by bacterial biofilm; Dense colonies of filamentous bacteria on the bone surface. | 📌 Histopathological Diagnosis:  Central Giant Cell Granuloma (CGCG)  🔍 Key Microscopic Features Supporting the Diagnosis:  Multinucleated Giant Cells:  Both images prominently display numerous multinucleated giant cells dispersed throughout a fibroblastic stroma, a hallmark of CGCG.  These cells are scattered unevenly and often cluster around areas of hemorrhage.  Fibrovascular Stroma:  The background stroma is composed of spindle-shaped fibroblasts and capillary-sized blood vessels, indicative of a reactive, proliferative lesion.  Hemorrhage and Hemosiderin Deposition:  Multiple areas of hemorrhage are visible, with possible hemosiderin pigment deposition, further supporting a reactive lesion like CGCG.  Absence of Cellular Atypia or Malignancy:  There is no evidence of cytologic atypia, necrosis, or high mitotic activity, which helps rule out malignancies such as giant cell tumor or sarcoma.  Bone Trabeculae and Resorption:  The presence of osteoid or bone trabeculae surrounded by giant cells and fibroblasts suggests bone remodeling or resorption, often seen in CGCG.  ✅ Summary:  The constellation of multinucleated giant cells, fibroblastic stroma, hemorrhage, and lack of atypia in an intraosseous location supports the diagnosis of Central Giant Cell Granuloma (CGCG), a nonodontogenic, reactive bone lesion commonly found in the jawbones. | Single most likely histopathological diagnosis  → Suppurative (sequestrating) osteomyelitis of the jaw – chronic / “acute-on-chronic” phase  Key microscopic features supporting the diagnosis  Feature seen in the photomicrographs Significance  Large, eosinophilic bony trabeculae lacking viable osteocytes (empty lacunae) Devitalised lamellar bone (sequestrum) – hallmark of osteomyelitis once vascular supply is lost  Irregular resorption surfaces without lining osteoblasts Confirms that the bone is non-vital and undergoing inflammatory destruction rather than active remodelling  Marrow spaces replaced by densely basophilic suppurative exudate – sheets of small polymorphonuclear cells with karyorrhectic debris, intermixed red blood cells and proteinaceous fluid Acute purulent component of the infection  Foci of granulation tissue and mixed chronic inflammatory infiltrate (lymphocytes / plasma cells) at the edges of the lesion Indicates the process has been present long enough to elicit a chronic reparative response (“chronic suppurative” stage)  Scattered hemorrhage and necrotic debris within the marrow cavity Common accompaniment of a bacterial osteomyelitic process  No organised neoplastic pattern, no multinucleated giant-cell aggregates, no tumor osteoid, and no odontogenic epithelial component Helps exclude central giant-cell lesion, osteosarcoma or any odontogenic tumour and narrows the differential to an inflammatory/infective condition  Together, the combination of dead bone (sequestra) plus a dominant neutrophil-rich exudate, evolving granulation tissue, and the absence of features suggestive of a specific primary tumour is diagnostic of suppurative osteomyelitis of the jaw in its sequestrating chronic phase. |
| **17.9** | Diagnosis: Medication-related osteonecrosis and osteomyelitis; Key Microscopic Features: Nonviable bony sequestrum surrounded by granulation tissue with abscesses; Nonviable bone is rimmed by osteoclasts within Howship lacunae; abscesses are present. | 🧠 Histopathological Analysis of Provided H&E-Stained Images  Diagnosis: Chronic Osteomyelitis  🔍 Justifying Key Microscopic Features:  1. Low Magnification Image (1st Image):  Irregular trabeculae of necrotic bone: The bone shows prominent empty osteocyte lacunae, a hallmark of sequestrum (devitalized bone).  Chronic inflammatory infiltrate: Surrounding the bone are numerous lymphocytes, plasma cells, and some neutrophils, suggestive of chronic and possibly smoldering acute inflammation.  Fibrosis and granulation tissue: There is evidence of fibrovascular tissue proliferation adjacent to necrotic bone, consistent with a chronic reparative process.  Lack of neoplastic proliferation: No atypical cells or invasive tumor patterns are seen, ruling out intraosseous malignancy or cystic neoplasms.  2. High Magnification Image (2nd Image):  Osteoclastic resorption: Presence of multinucleated osteoclast-like giant cells along bone surfaces reflects ongoing bone remodeling or destruction.  Dense inflammatory infiltrate: Mixed acute and chronic inflammation with neutrophils, plasma cells, and macrophages.  Bone necrosis: Clear absence of viable osteocytes in lacunae, confirming devitalized bone.  Reactive changes: The bone matrix itself appears disorganized near the surface with signs of reactive bone formation and resorption.  🧾 Summary:  The constellation of necrotic bone, chronic inflammatory infiltrate, and reactive changes is classic for Chronic Osteomyelitis, an inflammatory condition often associated with prior trauma, infection, or dental origin in the maxillofacial region. No features suggest neoplasia or fibrous lesions like fibrous dysplasia. | Most likely histopathological diagnosis  → Acute suppurative osteomyelitis of the jaw with sequestrum formation  Microscopic features that lead to this diagnosis  Field Findings (correlated with the two magnifications) Diagnostic implication  Devitalised bone (“sequestrum”) Broad, deeply eosinophilic lamellar bone lacking viable osteocytes; separated from surrounding tissues by clefts Necrotic, non-vital bone is characteristic of osteomyelitis with sequestration  Irregular resorption surface Scalloped Howship lacunae and occasional osteoclast-type giant cells along the bone margin Active bone destruction typical of an inflammatory osteolytic process  Purulent inflammatory infiltrate Dense sheets of neutrophils filling marrow spaces and abutting the necrotic bone; admixed with debris and occasional foamy macrophages A brisk neutrophilic exudate defines the “suppurative” (acute) phase  Reactive/ granulation tissue Fibrovascular tissue with mixed chronic inflammatory cells at the periphery of the lesion (seen at low power) Represents the host response trying to wall off the infection  Possible bacterial colonies (not prominent in every field) Basophilic granular material on bone surface in some foci Frequently seen in acute osteomyelitis, although not required for the diagnosis  These combined features—the presence of necrotic sequestrated bone, a heavy neutrophilic infiltrate, and active bone resorption—are classic for acute suppurative osteomyelitis within the non-odontogenic intra-osseous inflammatory/reactive category of oral and maxillofacial pathology. |
| **17.10** | Diagnosis: Medication-related osteonecrosis; Key Microscopic Features: Pseudoepitheliomatous hyperplasia with granulation tissue and acute and chronic inflammation; The epithelium exhibits spongiosis and leukocyte transmigration with minimal reactive epithelial atypia. | Based on the provided hematoxylin-and-eosin stained images of a lesion from the nonodontogenic intraosseous tumors and inflammatory/reactive conditions group in oral and maxillofacial pathology, the most likely histopathological diagnosis is:  Central Giant Cell Granuloma (CGCG)  Key Microscopic Features Supporting This Diagnosis:  Lobular architecture:  The lesion shows a lobulated growth pattern, separated by fibrous septa—common in CGCG.  Multinucleated giant cells:  Scattered multinucleated giant cells (osteoclast-like) are distributed throughout the fibrous stroma, typically clustered around hemorrhagic areas.  Fibroblastic stroma:  The background stroma consists of spindle-shaped fibroblastic cells with a variable cellularity.  Hemorrhagic areas and hemosiderin:  Foci of hemorrhage are seen, sometimes with hemosiderin deposition, which is typical for CGCG due to its reparative nature.  Absence of atypia or malignant features:  There is no evidence of pleomorphism, abnormal mitotic activity, or necrosis, helping to exclude malignant lesions such as giant cell tumor or sarcoma.  Differential Diagnoses Considered but Less Likely:  Brown tumor of hyperparathyroidism: Similar histology but requires systemic endocrine context (elevated parathyroid hormone).  Aneurysmal bone cyst (ABC): Usually shows blood-filled spaces without endothelial lining, often more cystic and with more prominent reactive bone.  Giant cell tumor: Typically affects long bones in adults, more uniformly distributed giant cells, and more aggressive. | Most likely histopathological diagnosis  Chondromyxoid fibroma (CMF) – a benign cartilaginous tumour of bone occasionally encountered in the jaws.  Key microscopic features supporting this diagnosis  Feature Appearance in the submitted sections Diagnostic significance  Distinct lobulated architecture The lesion is divided into multiple, well-circumscribed lobules separated by thin fibrous septa; each lobule has gently scalloped, “map-like” borders. Classic low-power pattern of CMF.  Biphasic cellularity • Central core: hypocellular, pale eosinophilic to slightly basophilic matrix  • Peripheral rim: densely cellular zone of spindle/ovoid cells that condense against the septa The central-vs-peripheral cellular gradient is a hallmark of CMF and helps separate it from uniformly cellular tumours (e.g., chondroblastoma).  Chondroid / myxoid extracellular matrix The matrix in the lobular centres has a gelatinous (“myxoid”) quality with subtle bluish tint on H&E. CMF is the only common jaw tumour that consistently shows both chondroid and myxoid ground substance.  Occasional calcified foci / early osteoid Small, irregular eosinophilic nodules within some lobules. Foci of dystrophic calcification or immature woven bone are frequent but usually scant in CMF.  Multinucleated giant cells & capillaries in septa Scattered osteoclast-type giant cells and thin-walled vessels are present in the fibrous septa and at lobule edges (seen focally). Their distribution at lobular margins supports CMF over central giant-cell granuloma, where giant cells are diffuse.  Cortical bone shell A continuous rim of pre-existing cortical bone outlines the lesion, showing an intra-osseous, expansile growth pattern. Fits the behaviour of CMF, which expands the jawbone without permeative invasion.  Together, the lobulated “chondromyxoid” pattern with peripheral cell condensation and inter-lobular fibrous septa is diagnostic for chondromyxoid fibroma. |
| **17.15** | Diagnosis: Fibrous dysplasia; Key Microscopic Features: Delicate trabeculae of bone in fibrous stroma abut the mucosa with no intervening cortical lamellar bone; Delicate interlacing trabeculae of woven bone with U and C shapes in fibrous stroma with peritrabecular clefting; Woven bone with surrounding fibrocollagenous tissue; Focal osteoblastic rimming is present. | Histopathological Analysis:  Single Most Likely Diagnosis:  Ossifying Fibroma  Key Microscopic Features Justifying the Diagnosis:  Well-demarcated lesion:  Seen clearly in the low-power image, showing a sharp interface between the lesion and surrounding bone, typical of a benign neoplasm like ossifying fibroma.  Fibrous stroma:  The lesion has a moderately cellular fibrous background composed of spindle-shaped fibroblasts. This is characteristic of fibro-osseous lesions.  Trabeculae of woven bone:  There are irregular, interconnected trabeculae of immature (woven) bone and osteoid, which are rimmed by osteoblasts. This immature bone appears eosinophilic and curvilinear or "Chinese character-like" in shape — a hallmark of ossifying fibroma.  Cementum-like or osteoid-like calcifications:  The images also reveal rounded or globular mineralized structures resembling cementum in addition to trabecular bone.  Lack of inflammatory infiltrate:  There is minimal chronic inflammation, distinguishing this from reactive or inflammatory conditions like chronic osteomyelitis.  Lack of cellular atypia or mitotic activity:  No evidence of malignancy such as pleomorphism or high mitotic activity, helping rule out osteosarcoma or aggressive fibro-osseous lesions. | Histopathological diagnosis (single best):  Fibrous dysplasia of bone  Key microscopic features supporting this diagnosis  Feature How it appears in the submitted sections Diagnostic significance  Irregular, curvilinear trabeculae of woven bone (“Chinese-character” shapes) Eosinophilic bony spicules of varying thickness meander through the lesion in complex, branching patterns (see low- and mid-power images). Classic architectural hallmark of fibrous dysplasia; distinguishes it from the orderly trabeculae of normal lamellar bone or cementum-like spherules of other fibro-osseous lesions.  Absence of continuous osteoblastic rimming The surfaces of the trabeculae merge imperceptibly with the surrounding stroma; only occasional inconspicuous lining cells are seen at high power. Lack of prominent rimming helps separate fibrous dysplasia from ossifying / cemento-ossifying fibroma, which typically shows a well-formed osteoblastic border.  Fibro-cellular stroma composed of bland spindle to stellate fibroblasts Between trabeculae, a moderately cellular collagenous matrix with whorled areas is evident; nuclei are uniform, without atypia or mitotic activity. Reflects the proliferative fibrous component into which the dysplastic bone is deposited.  Gradual blending of lesion with adjacent normal bone / cortex At low power the lesion appears to fuse with overlying cortical bone rather than being sharply delineated. A zone-less transition is typical for fibrous dysplasia, in contrast to the well-circumscribed capsule seen in ossifying fibroma.  Intact overlying stratified squamous oral mucosa Surface epithelium is unremarkable, confirming an intra-osseous, non-epithelial process. Consistent with a primary intramedullary fibro-osseous lesion rather than a mucosal neoplasm or cyst.  Together, these findings—irregular woven bone trabeculae without rim-lining osteoblasts, set in a cellular fibrous matrix that blends with native bone—are diagnostic of fibrous dysplasia within the spectrum of non-odontogenic intra-osseous lesions of the jaws. |
| **17.16** | Diagnosis: Fibrous dysplasia in 50-year-old patient; Key Microscopic Features: Thicker trabeculae of bone with more densely fibrous tissue and peritrabecular clefting; Focal osteoblastic rimming and osteoclastic activity is present; Polarized light reveals delicate parallel lamellae. | Histopathological Diagnosis  Fibrous Dysplasia  Justifying Microscopic Features Observed in the Images:  Irregularly Shaped Trabeculae of Woven Bone:  The bone trabeculae seen in both images are curvilinear and lack the usual organization into lamellar bone.  These trabeculae resemble "Chinese characters" — a classic descriptive feature of fibrous dysplasia.  Absence of Osteoblastic Rimming:  A hallmark of fibrous dysplasia is the absence (or only focal presence) of prominent osteoblastic rimming around bone trabeculae, which helps to distinguish it from ossifying fibroma and other fibro-osseous lesions.  The higher magnification image clearly shows the woven bone trabeculae blending into the surrounding stroma without prominent osteoblast lining.  Fibrocellular Stroma:  The intervening background stroma is composed of relatively uniform spindle-shaped fibroblasts in a collagenous matrix.  No significant cellular atypia or mitotic activity is noted, consistent with a benign reactive process.  Lack of Inflammatory Infiltrate or Malignant Features:  There is no evidence of inflammation, necrosis, or malignancy.  This supports a diagnosis within the fibro-osseous lesion spectrum, specifically fibrous dysplasia. | Histopathological diagnosis  Fibrous dysplasia (monostotic type, fibro-osseous lesion)  Key microscopic features supporting this diagnosis  Feature Appearance in the submitted slides Diagnostic value  Irregular, curvilinear bony trabeculae (“Chinese-letter” or “alphabet-soup” pattern) At low power the lesion is composed of numerous thin, anastomosing trabeculae of eosinophilic bone that twist and branch through the section, producing an overall woven, labyrinth-like architecture. Hallmark pattern for fibrous dysplasia; contrasts with the round or lamellar trabeculae of ossifying fibroma.  Immature (woven) bone High-power view shows osteoid with haphazardly arranged osteocytes within lacunae; the bony matrix lacks the parallel lamellae of mature bone. Immaturity of the bone is characteristic of fibrous dysplasia.  Absence (or minimal presence) of osteoblastic rimming Most trabecular surfaces show no continuous layer of plump osteoblasts; only occasional flattened lining cells are present. Distinguished from ossifying fibroma and other neoplasms where prominent osteoblastic rimming is typical.  Moderately cellular fibroblastic stroma The intervening connective tissue is composed of uniform spindle / fibroblast-like cells in a collagenous matrix with scattered small vessels; no significant atypia or mitotic activity. The bland, relatively uniform, fibrocellular background supports a benign fibro-osseous process.  Intimate blending of bone and stroma without a capsule Trabeculae merge imperceptibly with the surrounding fibrous tissue; no sharply demarcated margin is seen. Helpful in separating fibrous dysplasia (non-encapsulated, blends with host bone) from well-circumscribed ossifying fibroma.  Taken together, the combination of Chinese-letter woven bone, lack of consistent osteoblastic rimming, and the seamless integration of bone with a cellular fibrous stroma is diagnostic of fibrous dysplasia. |
| **17.18** | Diagnosis: Trabecular ossifying fibroma; Key Microscopic Features: Cellular proliferation of spindle cells with osteoid, woven bone, hemorrhage and early aneurysmal bone cyst formation; Osteoid is present in poorly delineated spicules, contains plump osteocytes, and is surrounded by plump spindle and stellate cells; Plump and slightly atypical osteocytes and osteoblasts, and cellular stroma with spindle and stellate cells; Early aneurysmal bone cyst-like areas. | Histopathological Diagnosis:  Central Giant Cell Granuloma (CGCG)  Key Microscopic Features Supporting the Diagnosis:  Multinucleated Giant Cells:  Seen prominently in multiple fields (especially in images 2 and 3).  Scattered throughout a background of spindle-shaped mononuclear stromal cells.  The giant cells often contain numerous nuclei and are irregularly distributed, typical of CGCG.  Spindle Cell Background:  The interspersed stromal cells are spindle-shaped and arranged in a somewhat whorled pattern, as seen in images 2 and 3.  Moderate cellularity and minimal atypia support a benign/reactive process.  Hemorrhage and Hemosiderin Deposition:  Foci of hemorrhage with erythrocyte extravasation are present (visible especially in image 4).  Hemosiderin-laden macrophages may also be seen, which is typical in CGCG.  Reactive Bone Formation:  In the low-power view (image 1), there are irregular trabeculae of woven bone and areas suggestive of osteoid deposition amidst the lesion.  This supports a reparative/reactive nature.  Lack of Malignant Features:  No significant cytologic atypia or high mitotic activity is present.  No epithelial or odontogenic components are seen, which helps rule out odontogenic tumors. | Most likely histopathological diagnosis  → Fibrous dysplasia of bone (cranio-facial/gnathic type)  Key microscopic features seen in the provided H-E sections  Field Salient findings How they support fibrous dysplasia  Low-power architecture • Poorly circumscribed lesion blending with surrounding marrow.  • Numerous thin, irregular, curvilinear trabeculae of mineralised tissue that branch and anastomose, giving a “Chinese-writing” or alphabet-soup pattern. This pattern of woven bone spicules without a capsule or sharp interface is characteristic of fibrous dysplasia rather than ossifying fibroma or other fibro-osseous lesions.  Nature of the trabeculae • Trabeculae are composed of immature woven bone/osteoid; many are delicate and lack obvious lamellation.  • Virtually no osteoblastic rimming; the bone merges imperceptibly with adjacent stroma. Absence of plump lining osteoblasts and seamless transition into stroma are classic for fibrous dysplasia and help separate it from ossifying fibroma (which shows prominent osteoblastic rimming) or reactive new bone.  Fibro-osseous stroma • Stroma is moderately cellular, fibroblastic, with spindle cells arranged in sweeping or whorled fascicles (second and third images).  • Collagen is delicate; no significant cytologic atypia or high mitotic activity. A bland, fibroblastic background of this type is expected in fibrous dysplasia.  Secondary changes • Foci of haemorrhage and cholesterol/foamy macrophage aggregates (fourth image).  • Occasional cyst-like spaces or necrotic bone fragments. Chronic micro-fracture and repair in fibrous dysplasia commonly produce haemorrhage, cystic degeneration, and collections of lipid-laden macrophages (“brown tumour–like” areas).  Absence of confounding features • No conspicuous multinucleated giant cell clusters (rules against central giant-cell granuloma).  • No sharply circumscribed border or zonation pattern (rules against ossifying fibroma). These negative findings further refine the diagnosis.  Taken together, the mosaic of Chinese-character woven bone, lack of osteoblastic rimming, and bland fibroblastic stroma in a poorly demarcated intra-osseous lesion is diagnostic of fibrous dysplasia. |
| **17.19** | Diagnosis: Trabecular ossifying fibroma; Key Microscopic Features: Spindle cell proliferation with hyper- and hypocellular areas, osteoid, woven bone and many giant cells in the hypocellular areas; Woven bone with large lacunae with plump osteocytes and many osteoclasts; Woven bone with plump, slightly atypical osteocytes, osteoclasts, and stellate and spindle cells within stroma; Clusters of multinucleated giant cells within myxoid stroma. | Based on the provided hematoxylin-and-eosin (H&E) stained histological images at multiple magnifications, the single most likely histopathological diagnosis is:  Central Giant Cell Granuloma (CGCG)  🔬 Key Microscopic Features Supporting the Diagnosis:  Multinucleated Giant Cells:  Seen especially in the last image (high magnification), there are numerous osteoclast-like multinucleated giant cells, often clustered and dispersed throughout the lesion.  These cells contain multiple nuclei and are located within a background of mononuclear stromal cells.  Spindle-shaped Stromal Cells:  A background population of spindle-shaped mononuclear stromal cells is present, evident in medium magnification images.  These cells may be plump and arranged loosely or more densely in fascicles.  Hemorrhage and Hemosiderin Deposition:  Areas of extravasated red blood cells and hemosiderin-laden macrophages can be appreciated, particularly in the intermediate to low magnification images.  This is a hallmark of CGCG, reflecting the lesion's vascular nature.  Reactive Bone Formation:  Foci of new woven bone or osteoid-like material may be seen intermixed with the cellular stroma, although subtle. These are signs of reactive changes.  Lack of Atypia or Malignancy:  Despite cellularity, there is no significant cytologic atypia, mitotic figures are sparse, and no features of malignancy are seen.  📌 Diagnostic Summary:  The combination of multinucleated giant cells, spindle cell stroma, areas of hemorrhage, and lack of atypical mitotic activity is classic for Central Giant Cell Granuloma.  CGCG is a non-neoplastic, reactive intraosseous lesion seen predominantly in the anterior mandible of younger individuals, and it belongs to the category of nonodontogenic intraosseous tumors and reactive conditions. | Histopathological diagnosis  ► Central (non-odontogenic) giant-cell granuloma (CGCG) of the jaws  Key microscopic features supporting the diagnosis  Magnification What is seen Significance  Low power (1st image) Diffuse, unencapsulated lesion replacing marrow; mottled mixture of pale haemorrhagic foci and more cellular purple areas Typical overall architecture of CGCG, which infiltrates cancellous bone and contains multiple haemorrhagic/vascular zones  Intermediate power (2nd image) Irregular islands and ribbons of eosinophilic woven osteoid/bone embedded in a cellular spindle-cell stroma Reactive bone deposition that commonly accompanies CGCG (“reparative” component)  High power (3rd image) Osteoid trabeculae merge directly with the surrounding fibrous stroma; no orderly osteoblastic rimming; numerous plump spindle / ovoid fibroblasts Confirms the lesion is fibro-osseous and reactive rather than a true neoplasm of osteoblasts  High power (4th image) Multiple large osteoclast-type multinucleated giant cells (10-25 centrally clustered nuclei) dispersed among the stromal cells, many adjacent to areas of recent haemorrhage; scattered extravasated erythrocytes and siderophages The diagnostic hallmark of CGCG—numerous giant cells in a fibro-vascular milieu; their distribution around haemorrhage helps distinguish CGCG (and cherubism) from other fibro-osseous lesions  Collectively, the combination of:  abundant osteoclast-like multinucleated giant cells,  a richly vascular / haemorrhagic spindle-cell stroma, and  patchy reactive woven-bone formation  is characteristic for central giant-cell granuloma and fits within the “Non-odontogenic intra-osseous tumours and inflammatory/reactive conditions” category of oral & maxillofacial pathology. |
| **17.20** | Diagnosis: Trabecular ossifying fibroma; Key Microscopic Features: Slightly storiform and fasciculated pattern of spindle cells and stroma with ossification; Calcified structures are acellular and exhibit the trailing ‘paintbrush’ appearance of osteoid recapitulating Sharpey fibers. | Histopathological Analysis of Provided H&E-Stained Sections  🧬 Single Most Likely Histopathological Diagnosis:  Central Giant Cell Granuloma (CGCG)  🔬 Key Microscopic Features Supporting the Diagnosis:  Low-power Image Observations (First Image):  Spindle-cell stroma arranged in interlacing fascicles or whorled patterns.  Fairly uniform distribution of cellularity with scattered vascular channels.  Absence of encapsulation and lack of well-formed epithelial components.  High-power Image Observations (Second Image):  Presence of numerous multinucleated giant cells dispersed throughout the lesion.  These giant cells are often clustered around areas of hemorrhage or resemble osteoclast-like morphology.  Background mononuclear stromal cells with bland nuclei and scattered mitoses.  Areas of extravasated red blood cells and possible hemosiderin pigment suggest prior microhemorrhages.  No significant cytological atypia or necrosis is observed, which helps differentiate from more aggressive entities like giant cell tumor or osteosarcoma.  🧠 Diagnostic Considerations:  This lesion fits the classic histological profile of Central Giant Cell Granuloma, which is:  A non-neoplastic, but sometimes locally aggressive, intraosseous lesion.  Most commonly occurs in the anterior mandible, frequently crossing the midline.  Histologically characterized by a proliferation of spindle-shaped mesenchymal cells interspersed with osteoclast-like giant cells. | Most likely histopathological diagnosis  → Central cemento-ossifying fibroma (a benign fibro-osseous tumour of the jaws)  Key microscopic features supporting this diagnosis  Field Observed feature Diagnostic significance  Low-power architecture • Sharply circumscribed, expansile nodule composed of interlacing, whorled fascicles of bland spindle-shaped fibroblastic cells.  • Overall “storiform”/swirling pattern without infiltrative edges. A well-demarcated, moderately cellular fibrous stroma is typical of an ossifying (cementifying) fibroma and helps separate it from more diffuse processes such as fibrous dysplasia.  Stromal cells • Uniform spindle cells with elongated nuclei, inconspicuous nucleoli, scant cytoplasm.  • Minimal cytologic atypia and very low mitotic activity. Confirms the benign nature; rules out sarcoma or aggressive spindle-cell lesions.  Mineralised component • Numerous irregular, basophilic to partly eosinophilic calcified masses scattered throughout the lesion (seen best on the high-power image).  • Calcifications vary from rounded “cementoid” globules to trabeculae of immature woven osteoid; most are acellular and lack organised lamellae.  • Occasional peripheral rim of plump spindle/osteoblastic cells around some trabeculae. The admixture of fibrous tissue with both cementum-like globules and immature bone is the hallmark of a cemento-ossifying fibroma. Other jaw fibro-osseous lesions show different patterns of mineralisation (e.g., more diffuse woven bone in fibrous dysplasia or numerous osteoclast-type giant cells in central giant-cell granuloma).  Vascular/haemorrhagic changes Scattered thin-walled vessels; no significant haemorrhage or giant-cell aggregates. Absence of prominent hemosiderin deposits or multinucleated giant cells helps exclude central giant-cell granuloma or aneurysmal bone cyst.  Why alternative entities are less likely  Fibrous dysplasia: usually poorly circumscribed and shows curvilinear “Chinese-character” woven bone directly blending with surrounding stroma; those features are not present here.  Central giant-cell granuloma / ABC: would display sheets of osteoclast-type multinucleated giant cells and haemorrhagic, cyst-like spaces, absent in these sections.  Desmoplastic fibroma: entirely fibrous with no/minimal mineralisation; our case contains abundant calcified material.  Taken together, the sharply demarcated spindle-cell stroma combined with mixed cementoid and woven-bone calcifications is diagnostic of central cemento-ossifying fibroma. |
| **17.21** | Diagnosis: Psammomatoid ossifying fibroma; Key Microscopic Features: Cellular spindle cell proliferation packed with ossicles; Rounded and ovoid paucicellular and basophilic ossicles; Ossicles and more basophilic psammoma-like bodies surrounded by plump, benign spindle cells. | 🧠 Histopathological Analysis  Based on the three hematoxylin and eosin (H&E) stained images provided at varying magnifications, the single most likely diagnosis is:  Central Giant Cell Granuloma (CGCG)  🔬 Key Microscopic Features Supporting the Diagnosis:  Multinucleated Giant Cells:  Numerous osteoclast-type multinucleated giant cells are distributed throughout the lesion.  The giant cells have multiple nuclei and are found within a background of mononuclear stromal cells.  Fibroblastic Stroma:  The background stroma is cellular and composed of spindle-shaped mononuclear cells.  These stromal cells are relatively uniform and lack significant atypia.  Hemorrhage and Hemosiderin Deposition:  Areas with extravasated red blood cells (suggestive of recent hemorrhage) are evident.  Hemosiderin pigment can sometimes be seen in this condition, although not definitively in these images.  Reactive Bone Formation (Focal):  Some regions show reactive woven bone trabeculae or osteoid formation, supporting a reparative/reactive nature.  Lack of Malignant Features:  No significant pleomorphism, atypical mitoses, or necrosis to suggest a malignant giant cell-rich lesion like giant cell tumor (GCT) or sarcoma.  🧾 Differential Diagnoses Considered (but less likely):  Brown Tumor of Hyperparathyroidism: Similar histology, but requires clinical/laboratory correlation (elevated PTH, calcium).  Giant Cell Tumor (GCT): Typically more aggressive, occurs in long bones, and shows a more uniform distribution of giant cells.  Aneurysmal Bone Cyst (ABC): Usually has cystic spaces with blood, which is not prominent here.  Cherubism: Similar histology, but occurs in children and usually bilaterally in the jaws.  📌 Conclusion:  The constellation of numerous multinucleated giant cells, cellular fibrous stroma, and hemorrhagic background without features of malignancy points toward Central Giant Cell Granuloma (CGCG)—a benign, non-neoplastic intraosseous lesion commonly found in the jaws. | Histopathological diagnosis  Central ossifying fibroma ( a.k.a. cemento-ossifying fibroma), a benign fibro-osseous tumour of the jaws that arises from periodontal-ligament-derived mesenchyme.  PMC  ScienceDirect  Key microscopic features visible in the three H-E fields  # Feature seen How it supports the diagnosis  1 Numerous, irregular islands & globules of eosinophilic mineralised matrix that contain small empty lacunae (future osteocytes) (best appreciated in the low- and medium-power images). Represents woven bone / cementum-like material typical of ossifying fibroma; unlike fibrous dysplasia the mineralised pieces are discrete and rounded rather than “Chinese-letter” shaped.  2 Well-defined borders between mineralised foci and surrounding stroma (clear outline, no blending). Ossifying fibroma is usually well demarcated histologically; in contrast, developmental lesions such as fibrous dysplasia merge imperceptibly with adjacent tissue.  3 Fibro-cellular stroma composed of uniform spindle / stellate fibroblasts arranged in whorls; scant chronic inflammatory cells; rich delicate vasculature. A moderately cellular, fibroblastic background is a hallmark of fibro-osseous tumours and helps distinguish the lesion from inflammatory osteomyelitis or cysts.  4 Minimal to absent osteoblastic rimming around the bony trabeculae. Conventional central ossifying fibroma shows little or patchy rimming, whereas osteoblastoma / osteoid osteoma display prominent plump osteoblasts lining every trabecula.  5 Absence of multinucleated osteoclast-type giant cells or haemorrhagic pools. Helps exclude central giant-cell granuloma and aneurysmal bone cyst that sit in the same jaw-lesion differential.  Taken together, the combination of discrete cementum-/bone-like masses embedded in a cell-rich, yet orderly fibrous stroma with sharp trabecular borders and little osteoblastic rimming is classic for a central ossifying fibroma of the jaws. |
| 17.23 | Diagnosis: Central Giant Cell Granuloma; Key Microscopic Features: Nodular clusters of giant cells and spindled monocytic cells are observed, along with hemorrhage, fibrosis, and hemosiderin deposits. | Based on the provided hematoxylin-and-eosin (H&E) stained histological images taken at different magnifications, the most likely single histopathological diagnosis is:  Central Giant Cell Granuloma (CGCG)  Key Microscopic Features Supporting This Diagnosis:  Low-Power Image (First Image):  Lobulated architecture with areas of cellular stroma separated by fibrous septa.  Presence of numerous multinucleated giant cells dispersed throughout the lesion.  Intervening fibroblastic proliferation in a background of spindle-shaped mononuclear stromal cells.  Areas of hemorrhage and possibly hemosiderin deposition (brownish pigment), consistent with repeated microbleeding.  High-Power Image (Second Image):  Multinucleated giant cells with centrally to peripherally placed nuclei.  Mononuclear spindle-shaped stromal cells, with mild to moderate pleomorphism.  Scattered extravasated red blood cells and hemosiderin-laden macrophages, typical of reactive lesions.  The stroma may appear vascular and slightly edematous in places. | Most likely histopathological diagnosis  → Intra-osseous (craniofacial) nodular fasciitis  Key microscopic features supporting this diagnosis  Field What is seen Why it matters  Overall architecture (low-power) Two relatively well-demarcated, very cellular nodules separated by paler, more collagenised spindle-cell tissue forming a characteristic whorled/“swirled” pattern. Nodular fasciitis typically shows a nodular growth with an internal “zonation” pattern rather than a diffuse infiltrate.  Cell type Plump spindle- to stellate-shaped fibro-/myofibroblasts with oval nuclei, delicate chromatin and small nucleoli; the cells are distributed in short intersecting fascicles that have a “tissue-culture” appearance. These cytological features are classic for nodular fasciitis and help distinguish it from bland fibroma or a more atypical sarcoma.  Stroma Alternating myxoid and more collagenised areas, giving a patchy light-and-dark background on low power. The myxoid–fibrous “biphasic” stroma is very typical and contributes to the feathery edge the lesion often shows at its periphery.  Vascular pattern & haemorrhage Numerous thin-walled capillaries and conspicuous extravasated erythrocytes scattered throughout the lesion. Prominent, often haemorrhagic vasculature with leaked red cells is a hallmark of nodular fasciitis and is far less common in most spindle-cell neoplasms.  Mitoses / atypia Mitotic figures are easily found, but nuclei remain uniform; no cytologic atypia, abnormal mitoses or necrosis are present. A brisk but non-atypical mitotic activity is characteristic of this self-limited, rapidly growing reactive process.  Bone present? No woven or lamellar bone trabeculae or osteoid rims are identified in the submitted fields. Lack of osseous production helps exclude fibro-osseous lesions (e.g., ossifying fibroma, fibrous dysplasia).  Taken together, the nodular architecture, tissue-culture-like myofibroblasts, alternating myxoid–fibrous matrix, abundant extravasated RBCs and non-atypical mitoses are diagnostic of intra-osseous nodular fasciitis, the craniofacial counterpart of the far more common soft-tissue lesion. |
| 17.24 | Diagnosis: Central Giant Cell Granuloma; Key Microscopic Features: Monocytic cells and multinucleated giant cells proliferate diffusely, accompanied by hemorrhage and reactive bone. | Based on the provided hematoxylin and eosin (H&E) stained histopathology images of a nonodontogenic intraosseous lesion from the oral and maxillofacial region at multiple magnifications, the most likely histopathological diagnosis is:  Melanotic Neuroectodermal Tumor of Infancy (MNTI)  Key Microscopic Features Supporting the Diagnosis:  Biphasic Cellular Population:  The tumor displays a distinct biphasic pattern of cells:  Large epithelioid cells with vesicular nuclei and abundant eosinophilic to clear cytoplasm, often containing brown melanin pigment (seen in the second image).  Smaller basophilic neuroblastic cells with scant cytoplasm and hyperchromatic nuclei, often arranged around the larger cells or in clusters (visible especially in the high-power views).  Melanin Pigment:  Presence of brown granular melanin pigment within the larger epithelioid cells, a hallmark of MNTI (highlighted clearly in image 2).  Cellular Arrangement and Stroma:  The tumor is well-circumscribed and composed of cellular nests or alveolar arrangements within a fibrous stroma (observed in the low-power image).  There may be areas with vascular spaces and hemorrhage, seen as red blood cells interspersed among tumor cells (noted in image 3).  Mitotic Activity and Nuclear Atypia:  While the lesion shows moderate cellularity, mitotic figures may be seen but are typically not brisk, and pleomorphism is limited—this aligns with the intermediate behavior of MNTI.  Additional Diagnostic Support (though not in image):  If immunohistochemistry were available, the tumor cells would show:  HMB-45, Melan-A, and NSE positivity in the larger cells (melanocytic differentiation),  Synaptophysin and CD56 positivity in smaller cells (neuroectodermal differentiation),  Negative for cytokeratin helps rule out epithelial tumors.  Conclusion:  The histopathologic findings, particularly the biphasic cell population, melanin pigmentation, and architecture, strongly favor the diagnosis of Melanotic Neuroectodermal Tumor of Infancy (MNTI) — a rare but characteristic tumor typically arising in the maxilla of infants. | Diagnosis — Conventional intramedullary (gnathic) osteosarcoma – fibro-/osteoblastic pattern  Key microscopic features supporting this diagnosis  Field What is seen Why it points to osteosarcoma  Tumour matrix Broad, irregular, eosinophilic, acellular zones of “lace-like” osteoid directly continuous with the lesional cells (centre of the medium-power image) Diagnostic hallmark – malignant mesenchymal cells are producing osteoid rather than merely encasing pre-existing bone  Cell population Interlacing fascicles and whorls of plump spindle-to-ovoid cells with moderate nuclear pleomorphism, hyperchromasia and scattered mitoses Cytologic atypia confirms a malignant spindle-cell neoplasm; fibroblastic/osteoblastic morphology is typical of conventional osteosarcoma of the jaws  Matrix–cell interface Tumour cells are embedded within and “stream” away from the osteoid without a rimming of orderly osteoblasts Excludes benign fibro-osseous lesions (which have regular osteoblastic rimming)  Stroma & vascularity Profuse small vessels, focal haemorrhage with hemosiderin-laden macrophages Common accompaniment in high-grade osteosarcoma and explains the pigmented granules  Growth pattern At low power, a solid, moderately cellular mass that effaces normal marrow and shows an infiltrative border Behaviour expected of an aggressive intramedullary bone sarcoma  Taken together, the production of malignant osteoid by atypical spindle cells in an intra-osseous jaw lesion places it squarely in the spectrum of conventional osteosarcoma (fibro-/osteoblastic subtype) within the “non-odontogenic intra-osseous tumours” category of oral & maxillofacial pathology. |
| 17.27 | Diagnosis: Aneurysmal Bone Cyst; Key Microscopic Features: Cyst-like spaces surrounded by fibrous and granulation tissue, containing numerous spindle and multinucleated giant cells with extensive hemorrhage. | Based on the provided hematoxylin and eosin (H&E) stained images of the same lesion at low and high magnifications, the most likely histopathological diagnosis is:  Central Giant Cell Granuloma (CGCG)  Key Microscopic Features Supporting the Diagnosis:  1. Multinucleated Giant Cells:  The high-power image shows numerous multinucleated giant cells scattered within a background of mononuclear stromal cells.  These giant cells are often clustered around areas of hemorrhage or vascular spaces.  2. Mononuclear Stromal Cells:  The background stroma is composed of oval to spindle-shaped mononuclear cells, which are a defining component of CGCG.  These cells are relatively uniform and show minimal atypia, consistent with a reactive, non-neoplastic process.  3. Hemorrhage and Hemosiderin:  Areas of recent hemorrhage and possibly hemosiderin-laden macrophages are evident, especially in the low-power image, reflecting the vascular nature and frequent bleeding within these lesions.  4. Lack of Odontogenic Epithelium:  No epithelial components are seen, which helps differentiate it from odontogenic lesions.  5. Lytic Pattern:  While not a histological feature per se, CGCG lesions often show a lobular or multinodular architecture histologically, correlating with their radiographic multiloculated radiolucent appearance.  Conclusion:  The lesion demonstrates hallmark features of a Central Giant Cell Granuloma, a nonodontogenic, intraosseous lesion of the jaws. It typically arises in the anterior mandible and is most common in younger individuals, often showing locally aggressive behavior depending on the subtype (aggressive vs. non-aggressive). | Histopathological diagnosis  ➜ Aneurysmal bone cyst (ABC) – central/ intra-osseous type involving the jaws  Key microscopic features seen in the provided H-E sections  Low-power view High-power view Significance for diagnosis  • Multiloculated, irregular cavernous spaces that are mostly empty (processing artifact) or packed with erythrocytes • Spaces are not lined by true endothelium – only a thin rim of fibro-connective tissue Diagnostic hallmark of ABC: blood-filled “lakes” rather than a true cyst with epithelial lining  • Fibro-cellular septa partition the spaces • Septa contain numerous osteoclast-type multinucleated giant cells with 10 – 30 nuclei Giant-cell–rich septa distinguish ABC from simple (traumatic) bone cyst  • Widespread fresh and organizing hemorrhage; pools of red blood cells and hemosiderin pigment • Extravasated RBCs permeate the stroma; occasional fibrin thrombi Heavy hemorrhage and hemosiderin are classic for ABC and explain its radiographic “blow-out” appearance  • Foci of reactive woven bone / osteoid may be present within septa (rudimentary in these fields) Reactive bone supports a reparative, not neoplastic, process  Collectively, the combination of large blood-filled cavities lacking endothelial lining, fibrous septa populated by osteoclast-like giant cells, and abundant hemorrhage/hemosiderin is pathognomonic for an aneurysmal bone cyst in the non-odontogenic intra-osseous category of jaw lesions. |
| 17.28 | Diagnosis: Aneurysmal Bone Cyst; Key Microscopic Features: A keratocystic odontogenic tumor with an aneurysmal bone cyst in the wall, filled with hemorrhagic fluid. The lining consists of multinucleated giant cells and spindle cells. | Histopathological Diagnosis:  Central Giant Cell Granuloma (CGCG)  Key Microscopic Features Justifying the Diagnosis:  1. Multinucleated Giant Cells:  Prominent in both low- and high-power fields.  Scattered throughout the lesion, often surrounding areas of hemorrhage.  These giant cells typically contain numerous nuclei and are irregularly distributed.  2. Background Mononuclear Stromal Cells:  The background consists of spindle-shaped and ovoid mononuclear cells.  These stromal cells are uniform and proliferative, supporting the lesion's reactive/proliferative nature.  3. Hemorrhage and Hemosiderin Deposition:  Extensive areas of extravasated red blood cells are evident, especially in the low-power view.  This is characteristic of CGCG, often associated with prior microtrauma or vascular instability.  4. Absence of Odontogenic Epithelium:  No evidence of odontogenic epithelial islands or nests, helping differentiate CGCG from odontogenic tumors like ameloblastoma or odontogenic myxoma.  5. Lack of Atypia or Malignancy:  No cytologic atypia, mitotic figures, or necrosis, supporting a benign, reactive nature.  Clinical Correlation Notes:  CGCG is a nonodontogenic, intraosseous lesion commonly affecting the anterior mandible in younger individuals, often presenting with painless swelling. Though benign, it can be locally aggressive. | Histopathologic diagnosis (single best answer)  Central giant-cell granuloma (central giant-cell lesion) of the jaws  Key microscopic features supporting this diagnosis  Feature (seen in the photomicrographs) Diagnostic significance  Unevenly distributed osteoclast-type multinucleated giant cells among a background of spindle-to-ovoid mononuclear stromal cells (best appreciated in the higher-power image) Hallmark of a giant-cell granulomatous process  Cell-rich, vascular fibroblastic stroma with plump spindle cells Typical background for central giant-cell granuloma, distinguishing it from fibrous dysplasia or ossifying fibroma  Extensive foci of extravasated erythrocytes and organizing hemorrhage with no endothelial lining (i.e., blood pools rather than true vascular spaces) Characteristic but not diagnostic; helps separate CGCG from true vascular lesions – the hemorrhage also explains the frequent presence of hemosiderin in these lesions  Reactive woven bone/osteoid and scattered deposits of fibrin at the periphery of the hemorrhagic areas Indicates a reparative process rather than a malignant neoplasm  Overlying intact oral stratified squamous epithelium with a sharp interface to the lesion Compatible with an intra-osseous lesion that has expanded to the cortical plate and been sampled through the mucosa; rules out a purely soft-tissue (peripheral) giant-cell granuloma  Taken together, the combination of multinucleated giant cells in a cellular fibrovascular stroma, conspicuous hemorrhage without an endothelial lining, and absence of features of malignancy most closely fits central giant-cell granuloma within the spectrum of non-odontogenic intra-osseous jaw lesions. |
| 17.30 | Diagnosis: Cherubism; Key Microscopic Features: A diffuse proliferation of spindled fibroblast-like cells with clusters of multinucleated giant cells within a delicate collagenous stroma, accompanied by fresh hemorrhage. | Based on the provided hematoxylin and eosin (H&E) stained histologic images of the same lesion, examined at different magnifications, the most likely histopathological diagnosis is:  Central Giant Cell Granuloma (CGCG)  Key Microscopic Features Supporting the Diagnosis:  Multinucleated Giant Cells:  Numerous multinucleated osteoclast-like giant cells are scattered throughout the lesion.  These are typically found in clusters and surrounded by mononuclear stromal cells.  Mononuclear Stromal Cells:  The background consists of spindle-shaped to ovoid mononuclear stromal cells, which are the proliferative component of the lesion.  Hemorrhage and Hemosiderin:  Areas of extravasated red blood cells and hemosiderin pigment deposition are evident, which is a common finding in CGCG.  Fibrovascular Tissue and Cystic Changes:  Presence of fibrous connective tissue interspersed with areas of hemorrhage and occasional vascular proliferation.  Some regions may show cystic degeneration or reactive bone formation.  Lack of Atypia or Malignancy:  The lesion lacks cellular atypia, mitotic figures, or necrosis that would suggest a malignant process.  Differential Diagnoses Considered:  Brown tumor of hyperparathyroidism: Histologically identical, but systemic context (e.g., elevated PTH, calcium levels) is needed for distinction.  Aneurysmal bone cyst (ABC): Can overlap but typically shows blood-filled spaces without endothelial lining.  Giant cell tumor (GCT) of bone: More aggressive histology and occurs typically in long bones of adults. | Histopathological diagnosis –  Central giant-cell granuloma (CGCG) / central giant-cell lesion of the jaws  Key microscopic features in the submitted H-E sections that support this diagnosis  Feature How it appears in your photomicrographs  1. Numerous osteoclast-type multinucleated giant cells Large cells with abundant eosinophilic cytoplasm containing 5-20 uniform, oval nuclei. They are scattered but often aggregate in small nests or cuffs (best appreciated in the high-power third image).  2. Cellular spindle-cell stroma Background composed of plump fibroblastic / myofibroblastic cells arranged in short fascicles and whorls; nuclei are oval to spindle-shaped with inconspicuous nucleoli and minimal atypia (seen in all magnifications).  3. Foci of hemorrhage and extravasated red blood cells Irregular pools and streaks of fresh blood coursing through the lesion (prominent in the second and fourth images), with accompanying clusters of giant cells around these hemorrhagic areas – the classic “perivascular/hemorrhagic cuffing” pattern.  4. Hemosiderin deposition & reactive changes Fine golden-brown pigment granules and occasional foamy macrophages at the edges of old hemorrhage (subtle on the medium-power fields).  5. Reactive woven bone / osteoid trabeculae Patchy, irregular, eosinophilic osteoid spicules rimmed by spindle cells (pink streak traversing the fourth image) – a common reparative response in CGCG.  6. Benign cytology Mitotic figures are rare and normal; no pleomorphism, necrosis, or permeative growth that would raise concern for a true giant-cell tumor or sarcoma.  These combined findings – a fibro-osseous, hemorrhagic lesion rich in osteoclast-type giant cells without malignant cytologic features – are characteristic of central giant-cell granuloma, the prototypical non-odontogenic intraosseous “giant-cell” lesion of the jaws. |
| 17.31 | Diagnosis: Desmoplastic Fibroma; Key Microscopic Features: Dense collagen bands are interspersed with scattered spindle cells, which exhibit benign morphology. | Based on the provided hematoxylin and eosin (H&E) stained histological images at different magnifications, the most likely histopathological diagnosis is:  Desmoplastic Fibroma  Key Microscopic Features Supporting the Diagnosis:  Cellularity and Cell Morphology:  The lesion is hypocellular to moderately cellular.  It contains spindle-shaped fibroblastic cells with bland, elongated nuclei, dispersed in a background of dense collagenous stroma.  No significant atypia, mitoses, or necrosis are present, indicating a benign but locally aggressive tumor.  Stromal Pattern:  The stroma is dense, collagenous, and whorled, with a uniform, fibrous appearance.  This dense collagen deposition gives a “desmoplastic” appearance, which is characteristic of desmoplastic fibroma.  Growth Pattern:  There is a lack of encapsulation with an infiltrative growth pattern, although this is better appreciated on gross or radiologic correlation.  The pattern is often interlacing fascicles of spindle cells within a fibrotic matrix, seen clearly in the lower-magnification image.  Differential Diagnoses Considered and Ruled Out:  Low-Grade Fibrosarcoma: Typically shows more cellularity, mild atypia, and a herringbone pattern—absent here.  Central Ossifying Fibroma / Fibrous Dysplasia: Would typically show osseous components or woven bone—these are absent.  Nodular Fasciitis: Generally more cellular and myxoid with prominent mitoses—also not seen here. | Histopathological diagnosis  Desmoplastic fibroma of bone  (the intra-osseous counterpart of desmoid-type fibromatosis)  Key microscopic features seen in the two H-E fields  Feature What you see in the images Significance for the diagnosis  Very low cellularity with abundant dense collagen Broad, pale-pink, almost glassy collagen bundles dominate both low- and high-power fields; fibroblastic nuclei are relatively sparse and widely separated. The collagen-rich, hypocellular stroma is the hallmark of desmoplastic fibroma and distinguishes it from more cellular spindle-cell lesions.  Bland spindle / stellate fibroblasts Elongated, tapered or stellate nuclei with fine chromatin and inconspicuous nucleoli; no cytologic atypia or mitotic activity. Confirms a benign fibroblastic proliferation rather than a sarcoma.  Long, sweeping / interlacing fascicles At low power the collagen bundles form wavy, interlacing, sometimes whorled patterns reminiscent of soft-tissue desmoid fibromatosis. Architectural pattern characteristic of desmoplastic fibroma.  Minimal inflammatory infiltrate and vascularity Only scattered lymphocytes; vessels are inconspicuous. Helps exclude reactive fibrous lesions (which are more inflamed/vascular) and certain tumors such as myofibroma.  Absence of mineralized matrix No woven bone trabeculae, cementum-like calcifications or osteoid deposition are present. Rules out fibrous dysplasia, ossifying fibroma, or low-grade osteosarcoma.  Taken together, the combination of hypocellular, bland spindle/stellate fibroblasts embedded in densely collagenized, sweeping fascicles without mineralized matrix or atypia is diagnostic of desmoplastic fibroma of bone within the spectrum of non-odontogenic intra-osseous lesions of the jaws. |
| 17.32 | Diagnosis: Desmoid Fibromatosis of Mandible; Key Microscopic Features: Delicate and dense collagen whorls with scattered spindle cells, whose cytoplasm is moderate, with trailing processes and benign elongated or ovoid nuclei. | Based on the provided hematoxylin and eosin (H&E) stained images at different magnifications, the lesion belongs to the category of Nonodontogenic Intraosseous Tumors and Inflammatory/Reactive Conditions in oral and maxillofacial pathology.  Histopathological Diagnosis:  Central Ossifying Fibroma (COF)  Key Microscopic Features Supporting This Diagnosis:  Cellular Fibrous Stroma:  The images show a highly cellular fibrous connective tissue composed of spindle-shaped fibroblastic cells arranged in a whorled or storiform pattern.  There is minimal atypia and mitotic activity, suggesting a benign nature.  Presence of Mineralized Material:  Although not extremely prominent in the provided images, there are regions suggestive of the early formation of mineralized material—possibly immature bone or cementum-like deposits. These features can often be focal or sparsely distributed in some sections.  Well-Circumscribed Nature:  The uniformity and separation of the lesion from adjacent tissues (not clearly demarcated here due to the field of view) are consistent with a benign, well-circumscribed neoplasm.  Lack of Odontogenic Epithelium:  No evidence of odontogenic epithelial islands or nests, ruling out odontogenic tumors such as ameloblastoma or odontogenic fibroma.  Correlating Clinical Considerations:  Central Ossifying Fibroma is a benign fibro-osseous neoplasm that arises in the jaws (usually the mandible) and is composed of fibrous tissue that may contain varying amounts of mineralized material. It commonly affects females in the 2nd to 4th decade of life. | Histopathological diagnosis  Desmoplastic fibroma of bone (the intra-osseous counterpart of desmoid-type fibromatosis).  Key microscopic features seen on the two H-E sections  Feature What is seen in the images Significance for diagnosis  Hypocellular, densely collagenous stroma The background is pale-pink and markedly fibrous; cellular elements are widely spaced by thick bundles of collagen. Desmoplastic fibroma is characteristically collagen-rich and relatively pauci-cellular.  Bland spindle / stellate fibroblastic cells Nuclei are slender, uniform, cigar-shaped to ovoid with fine chromatin and inconspicuous nucleoli; cytoplasm is scant and poorly defined. Confirms a benign fibroblastic/myofibroblastic proliferation; absence of atypia helps exclude sarcoma.  Interlacing fascicles and sweeping, whorled / storiform architecture The spindled cells run in long, sweeping bundles that curve and swirl around each other, creating whorled zones at low and medium power. This pattern is classic for desmoplastic fibroma and desmoid-type fibromatosis.  Very low mitotic activity, no necrosis or pleomorphism No mitotic figures or atypical forms are apparent; there is no coagulative necrosis. Reinforces a benign but locally aggressive lesion.  Scant, thin-walled vessels and small hemorrhagic foci A few capillaries with adjacent erythrocyte extravasation are present but not a prominent, branching vasculature. Helps distinguish the lesion from vascular spindle-cell tumours or myofibroma, which show a pronounced hemangiopericytoma-like pattern.  Absence of mineralized material, giant cells, or odontogenic epithelium No bone, cementum-like calcifications, multinucleated giant cells or odontogenic epithelial rests are seen. Excludes central ossifying fibroma, fibrous dysplasia, central giant-cell granuloma and odontogenic tumours.  The combination of a bland, hypocellular spindle-cell proliferation embedded in dense collagen, arranged in sweeping fascicles and whorls, with little mitotic activity and occurring intra-osseously is diagnostic of desmoplastic fibroma of bone. |
| 17.33 | Diagnosis: Xanthoma of Bone; Key Microscopic Features: Xanthoma cells form sheets along with fibrocollagenous tissue, and multinucleated giant cells are present. | Based on the provided hematoxylin and eosin (H&E) stained images of a lesion from the nonodontogenic intraosseous tumors and inflammatory/reactive conditions group in oral and maxillofacial pathology, the single most likely histopathological diagnosis is:  Desmoplastic Fibroma  Key Microscopic Features Supporting the Diagnosis:  Low-Power View (First Image):  Hypocellular lesion with interlacing bundles of spindle-shaped cells.  The stroma appears collagen-rich and densely fibrous, giving a "sweeping" or “storiform” pattern.  Minimal mitotic activity and lack of necrosis.  No encapsulation; lesion appears to infiltrate the surrounding bone or soft tissue.  High-Power View (Second Image):  Spindle cells have bland, elongated nuclei with fine chromatin, and inconspicuous nucleoli.  The cytoplasm is pale to eosinophilic and poorly defined.  There are no significant atypia, pleomorphism, or high mitotic figures—important for ruling out malignancy.  The abundant collagen and intercellular fibrous matrix between cells are prominent. | Histopathologic diagnosis  Intra-osseous myxoma (central myxoma / fibromyxoma) – a benign, infiltrative myxoid tumour of jaw bone mesenchyme.  Key microscopic features in the provided H-E slides  Field Observed feature Diagnostic significance  Low-power view Diffusely hypocellular lesion composed of streaming, interlacing fascicles and swirling whorls of pale-pink stroma with only scattered nuclei Overall architecture of a myxoid/fibromyxoid spindle-cell tumour; the sweeping pattern is typical of myxoma/fibromyxoma.  Uniform distribution of delicate, wavy collagen fibrils in a gelatinous background The loose collagen embedded in abundant ground substance is characteristic of a myxoid matrix rather than dense fibrous or bony tissue.  High-power view Stellate- to spindle-shaped tumour cells with long, tapering cytoplasmic processes; cells are <u>paucicellular</u>, with small, oval, dark nuclei and inconspicuous nucleoli Cytology is bland (virtually no pleomorphism or mitotic activity), supporting a benign myxoma rather than a sarcoma.  Ample, vacuolated / “empty-looking” cytoplasm and numerous clear extracellular lacunae created by loss of hyaluronic-acid-rich mucopolysaccharide during processing These vacuoles are a classic processing artifact of myxoid lesions and help identify the lesion as a myxoma.  Absence of mineralised material (no woven bone, cementum-like bodies or chondroid matrix) and no epithelial islands Rules out ossifying fibroma, chondromyxoid fibroma, and odontogenic tumours with epithelial components.  No multinucleated giant cells, dystrophic calcifications, haemorrhagic cystic spaces or inflammatory infiltrates Helps exclude central giant-cell granuloma, aneurysmal bone cyst and inflammatory/reactive processes.  Together, the hypocellular, bland spindle/stellate cell population in an abundant gelatinous (myxoid) matrix arranged in sweeping fascicles is diagnostic of an intra-osseous myxoma (fibromyxoma) of the jaws. |
| 17.34 | Diagnosis: Central Venular Malformation of Bone; Key Microscopic Features: Multiple vessels with varying wall thickness, comprising endothelium-lined spaces with muscle coats of variable thickness. | Based on the histopathological features visible in the provided hematoxylin and eosin (H&E) stained images at low and high magnification, the most likely diagnosis is:  Central Hemangioma (Intraosseous Hemangioma)  Key Microscopic Features Justifying the Diagnosis:  Lobular/Vascular Proliferation:  Both images show a lobular architecture of well-circumscribed, vascular channels distributed throughout the lesion.  These vascular spaces vary in size and are lined by a single layer of flat endothelial cells, indicating a benign vascular proliferation.  Presence of Blood-Filled Vascular Channels:  At higher magnification (second image), many of these spaces are clearly filled with red blood cells, supporting the vascular nature of the lesion.  Fibrous Stroma:  The intervascular stroma shows fibrous connective tissue with scattered spindle-shaped fibroblasts and no evidence of significant cytologic atypia or mitotic activity, which is consistent with a benign lesion.  Absence of Odontogenic Epithelium:  There is no evidence of odontogenic epithelial rests or other odontogenic components, which helps exclude odontogenic tumors.  Intraosseous Location:  Although clinical correlation is essential, the lesion appears consistent with central (intraosseous) hemangioma based on the morphology and tissue architecture, especially in the absence of surrounding soft tissue components that would suggest a peripheral vascular lesion. | Histopathological diagnosis (single best answer)  Intraosseous (central) cavernous hemangioma – i.e., a benign vascular malformation arising within jaw (non-odontogenic intraosseous vascular lesion).  Key microscopic features that support this diagnosis  Field view Salient findings Significance  Low-power overview (first image) Lobulated mass of numerous, closely packed, round-to-oval spaces engorged with erythrocytes. The spaces are separated by delicate fibrous septa; there is no infiltrative destruction of surrounding trabeculae. Architecture typical of a cavernous hemangioma: well-circumscribed collection of dilated vascular channels forming a “sponge-like” pattern inside bone.  Higher magnification (second image) • Each blood-filled space is lined by a single layer of flattened, bland endothelial cells.  • Septa composed of fibrous connective tissue containing scattered spindle-shaped fibroblasts; no cytologic atypia or mitoses.  • Absence of multinucleated giant cells, osteoid deposition, sarcomatous stroma, or epithelial components. Confirms a benign vascular lesion rather than an aneurysmal bone cyst (pseudocyst, no endothelial lining), central giant-cell granuloma (would show numerous osteoclast-type giant cells), or osteogenic/chondrogenic neoplasm.  Background stroma Sparse chronic inflammatory cells; occasional entrapped marrow fat at the periphery; no reactive bone or atypical woven bone formation. Helps exclude other reactive or neoplastic intraosseous entities.  Diagnostic synthesis  The combination of numerous, large, blood-filled, endothelium-lined cavernous channels arranged in a lobular architecture within a fibrous septal framework – without significant atypia, giant-cell proliferation, or osteoid/chondroid production – is characteristic of a central cavernous hemangioma of the jaws. |
| 17.36 | Diagnosis: Paget Disease of Bone (Osteitis Deformans); Key Microscopic Features: Interlacing trabeculae of woven bone, abundant osteoclasts, and dilated vessels are present. Osteoblasts are observed laying down osteoid. | Histopathological Diagnosis:  Central Ossifying Fibroma (COF)  Key Microscopic Features Supporting the Diagnosis:  Fibro-osseous Nature:  The lesion is composed of a cellular fibrous stroma interspersed with irregular bony trabeculae, which is a hallmark of fibro-osseous lesions.  Bony Trabeculae:  The trabeculae of woven and lamellar bone are evident, and they appear to be well-formed but separated by the fibrous stroma.  These trabeculae often contain osteocytes in lacunae, with no evidence of osteoblastic rimming, a characteristic often seen in ossifying fibroma rather than fibrous dysplasia.  Well-Circumscribed Borders:  Although not directly visible in histology slides, central ossifying fibroma typically appears well-demarcated histologically, which corresponds to the clearly separated nature of the bone islands from the stroma in the images.  Stroma:  The stroma is moderately cellular, composed of spindle-shaped fibroblastic cells without atypia or pleomorphism.  No signs of active inflammation or malignancy (such as nuclear atypia, mitotic figures) are observed.  Lack of Odontogenic Epithelium:  There is no presence of odontogenic epithelium, helping to exclude odontogenic tumors like ossifying odontogenic fibroma. | Most likely histopathological diagnosis  → Fibrous dysplasia (monostotic type) of the jaw  Key microscopic features supporting this diagnosis  Field of view What is seen Why it matters  Low-power overview Broad, interconnected trabeculae of immature woven bone forming an irregular, “Chinese-character” / “alphabet-soup” pattern within a uniformly cellular fibrous stroma Curvilinear, randomly oriented woven bone islands that appear to float in stroma are the architectural signature of fibrous dysplasia  Bone characteristics (medium–high power) • Trabeculae composed of woven bone with numerous entrapped osteocytes  • Minimal to absent osteoblastic rimming – the bony spicules blend imperceptibly with surrounding stroma Lack of prominent osteoblasts distinguishes fibrous dysplasia from ossifying/cemento-ossifying fibroma, where plump rimming osteoblasts are common  Stromal background Moderately cellular, bland fibro-spindle cell tissue with collagen bundles; scant chronic inflammatory cells; occasional small vascular channels A uniform fibrocellular stroma without cytologic atypia is typical; aggressive tumors (e.g., osteosarcoma) would show pleomorphism, mitoses, atypical cells  Interface & maturation No capsule; bone trabeculae merge directly with stroma and show no peripheral zoning or maturation gradient Non-encapsulated, merging interface helps separate fibrous dysplasia from well-circumscribed ossifying fibroma  Absence of other elements No cementoid globules, no mature lamellar bone with Haversian systems, no necrosis or malignant features Excludes cemento-ossifying variants, mature osteoma, or malignant bone tumors  Taken together, the constellation of curvilinear, immature woven bone spicules without osteoblastic rimming, set in a uniform fibrous stroma and lacking a capsule, is diagnostic of fibrous dysplasia. |
| 17.37 | Diagnosis: Osteoblastoma; Key Microscopic Features: Trabeculae of woven bone are seen with a basophilic hue within cellular stroma; osteoblasts and osteoclasts proliferate in a vascular stroma, and multiple layers of osteoblasts with osteoclasts are seen on the same trabeculae. | Based on the histopathological images provided, the most likely diagnosis for this case is Central Giant Cell Granuloma (CGCG), a common lesion in the group of nonodontogenic intraosseous tumors and inflammatory/reactive conditions in oral and maxillofacial pathology.  Key Microscopic Features Supporting the Diagnosis:  Multinucleated Giant Cells: The most prominent feature in all three images is the presence of multinucleated giant cells scattered throughout the lesion. These cells are large and contain several nuclei, a hallmark of CGCG.  Fibroblastic Stroma: The background of the lesion is composed of a loose, fibroblastic stroma, with scattered spindle-shaped fibroblasts. The presence of these fibroblasts in a somewhat loose arrangement is typical of CGCG.  Hemorrhagic Foci: Areas of hemorrhage, seen as regions with abundant pink, red-tinged material, suggest the presence of blood vessels and potential vascular changes within the lesion, which is often seen in CGCG.  Moderate Inflammatory Infiltrate: Throughout the lesion, there is a presence of a mixed inflammatory infiltrate, including lymphocytes and histiocytes. This infiltrate is characteristic of CGCG, particularly in more reactive or inflammatory forms.  Bone Resorption: In some areas, there is evidence of bone resorption, a feature often seen in CGCG as the lesion invades and remodels bone tissue.  These histopathological features—multinucleated giant cells, fibrous stroma, hemorrhagic areas, inflammatory infiltrate, and bone resorption—strongly suggest that this lesion is a Central Giant Cell Granuloma (CGCG). | Based on the images you provided, I will analyze the histopathological features of the lesion to provide a diagnosis, focusing on the key microscopic features and how they support this diagnosis.  Likely Diagnosis: Central Giant Cell Granuloma (CGCG)  Key Microscopic Features:  Multinucleated Giant Cells:  In all three images, there are numerous multinucleated giant cells scattered throughout the lesion. These are large, often exhibiting a large number of nuclei, which is a typical finding for central giant cell granulomas.  Granulation Tissue and Stroma:  The presence of fibrovascular tissue with a mix of inflammatory cells, predominantly mononuclear cells like lymphocytes and plasma cells, indicates a reactive and reparative process. The fibroblasts contribute to the formation of granulation tissue.  Hemosiderin Deposition:  There are areas showing hemorrhage and hemosiderin-laden macrophages (seen as hemosiderin deposits), which are characteristic of the reactive, reparative nature of CGCG. This is especially seen in the areas with more intense pink staining and suggests chronicity or prior bleeding.  Well-defined Borders:  In some areas, the lesion shows a well-defined margin with possible encapsulation, which can be indicative of a benign nature, like CGCG. Although it might show a locally aggressive behavior, CGCG typically does not invade surrounding bone.  Conclusion:  The presence of multinucleated giant cells, granulation tissue, inflammatory infiltrates, and hemosiderin supports the diagnosis of Central Giant Cell Granuloma. This lesion is commonly seen in the jawbones and is typically classified under non-odontogenic intraosseous tumors. It is benign but can locally destroy bone, which can be seen in imaging or clinical cases as well. |
| 17.39 | Diagnosis: Conventional osteoblastic osteosarcoma; Key Microscopic Features: Atypical osteoid and woven bone are observed; interconnected lacy trabeculae of osteoid and woven bone with large lacunae are present, and malignant osteoid contains numerous atypical osteoblasts and osteocytes with pleomorphic nuclei. | Based on the provided images of hematoxylin-and-eosin (H&E) stained tissue sections, it appears to show histopathological features indicative of a non-odontogenic intraosseous tumor. After evaluating these sections, the diagnosis and key microscopic features suggest:  Diagnosis: Central Giant Cell Granuloma (CGCG)  Key Microscopic Features:  Multinucleated Giant Cells: The images show large multinucleated giant cells scattered throughout the lesion, which is one of the hallmark features of CGCG. These giant cells are typically osteoclastic in nature, and their presence in this pattern is suggestive of CGCG.  Fibrovascular Stroma: The lesion is composed of a fibrous connective tissue stroma, which is rich in blood vessels. This stroma supports the giant cells and is typical in CGCG.  Reactive Bone Resorption: Areas of bone resorption are observed, often with adjacent fibrosis and hemorrhage. The bone resorption is characteristic of CGCG and is indicative of the lesion's aggressive, but benign, nature.  Inflammatory Cells: A mixed inflammatory infiltrate is visible in the stroma. These include lymphocytes, plasma cells, and a few neutrophils, which are typical in reactive conditions like CGCG.  Variable Cellular Density: The lesion exhibits a variation in cellular density, with some areas showing a more dense accumulation of giant cells, while other regions show a more diffuse arrangement of fibroblasts and blood vessels. This variability in cellularity is a known feature of CGCG.  These microscopic features together strongly suggest a Central Giant Cell Granuloma. | Based on the images provided, these show histological sections stained with hematoxylin and eosin (H&E), likely from a lesion in the oral and maxillofacial region. The features presented in the sections suggest a diagnosis of central giant cell granuloma (CGCG), which falls under the category of nonodontogenic intraosseous tumors.  Key Microscopic Features:  Multinucleated Giant Cells: In multiple images, large multinucleated giant cells are seen scattered throughout the lesion, which is a hallmark of CGCG.  Proliferation of Mesenchymal Cells: The presence of mesenchymal cells that are spindle-shaped and forming a somewhat loose to moderately dense matrix is typical in CGCG.  Vascular Spaces: Some of the lesions show areas with blood-filled spaces or vascular channels, further supporting the diagnosis of CGCG, as these vascular features are commonly seen.  Hemorrhagic Areas: Hemorrhage can also be noted in parts of the tissue, which is characteristic of this lesion.  No Bone Formation: The absence of significant bone formation or odontogenic differentiation points away from other lesions like osteoblastoma or odontogenic tumors, narrowing the focus on CGCG.  These features strongly support the diagnosis of central giant cell granuloma (CGCG), which typically presents as a benign lesion in the jaws, affecting both children and young adults, often causing localized bone destruction. |
| 17.40 | Diagnosis: Chondroblastic osteosarcoma; Key Microscopic Features: A cellular tumor with malignant osteoid and cartilage formation; atypical osteoblasts are seen along with deposition of malignant osteoid and atypical cartilage. | Based on the provided hematoxylin and eosin (H&E) images, which show the lesion at two different magnifications, here is the histopathological analysis:  Single Most Likely Diagnosis:  Central Giant Cell Granuloma (CGCG)  Key Microscopic Features Seen in the Images:  Multinucleated Giant Cells: The presence of numerous multinucleated giant cells (seen predominantly in the second image) is characteristic of central giant cell granuloma. These cells appear as large, multi-nucleated structures embedded within a spindle-shaped stromal background.  Cellular Stroma: The stroma is composed of spindled and fusiform-shaped cells. These cells can be seen distributed among the giant cells. The stroma often shows a somewhat loose to moderately cellular texture, as seen in both images.  Hemorrhage: There is some evidence of hemorrhagic areas, particularly in the regions where the giant cells are more concentrated. This finding suggests the possibility of a reactive lesion, typical for CGCG.  Lack of Epithelial Lining: The lesion is intrabony and lacks an epithelial lining, further distinguishing it from other potential diagnoses like cysts or odontogenic lesions.  Lack of Bone Formation: The images do not show significant bone formation or calcification, which helps differentiate CGCG from some other lesions, such as osteosarcoma or osteoblastoma.  These features suggest that the lesion is likely a Central Giant Cell Granuloma, a benign intraosseous tumor commonly found in the jawbones. | Based on the provided hematoxylin-and-eosin (H&E) images and the context of nonodontogenic intraosseous tumors and inflammatory/reactive conditions in oral and maxillofacial pathology, the following analysis can be made:  Most Likely Histopathological Diagnosis:  Central Giant Cell Granuloma (CGCG)  Key Microscopic Features:  Multinucleated Giant Cells: Both images display the presence of multinucleated giant cells, which are characteristic of CGCG. These cells are often scattered throughout the lesion, appearing as large, irregularly shaped cells with multiple nuclei.  Granulation Tissue and Stromal Cells: The background consists of a rich vascular network with numerous small spindle-shaped stromal cells, a feature commonly observed in CGCG. These cells form a loose, collagenous matrix.  Hemorrhagic Areas: The presence of blood vessels and hemorrhagic areas is evident, suggesting active tissue turnover or damage in the lesion. These areas of hemorrhage can sometimes form cystic spaces, as seen in the second image.  Proliferative Nature: The proliferation of fibroblasts and endothelial cells within the lesion further supports the diagnosis of CGCG, as this lesion is known for its slow growth but locally aggressive nature, often causing bone resorption.  Conclusion:  The histopathological features seen in the images, such as the multinucleated giant cells, hemorrhagic areas, and fibrous stroma, are characteristic of a Central Giant Cell Granuloma (CGCG). This diagnosis fits within the group of nonodontogenic intraosseous lesions and inflammatory/reactive conditions, particularly those involving the bone. |
| 17.41 | Diagnosis: Low-grade osteosarcoma; Key Microscopic Features: Interconnected trabeculae of woven bone are surrounded by delicate hypocellular fibrovascular stroma; woven bone contains plump osteocytes within lacunae, with minimal cytologic atypia, and slightly atypical spindle cells are present in the stroma. | The images you provided appear to show histopathological slides of a lesion in the context of oral and maxillofacial pathology. Based on the morphology and features visible in the slides, I can make an analysis.  Diagnosis:  The most likely histopathological diagnosis for these images is Central Giant Cell Granuloma (CGCG), which falls under the category of nonodontogenic intraosseous tumors and inflammatory/reactive conditions.  Key Microscopic Features:  Multinucleated Giant Cells:  Both images display the presence of large, multinucleated giant cells scattered throughout the lesion. These giant cells are a hallmark of CGCG.  Vascularized Stroma:  The second image clearly shows an area with a well-vascularized stroma, which is often seen in CGCG. This stroma may appear fibrovascular, contributing to the characteristic pink appearance seen in H&E-stained sections.  Osteoid Formation:  The first image shows areas where osteoid-like material is seen surrounding the multinucleated giant cells, a feature that is indicative of bone resorption and new bone formation, which is characteristic of CGCG.  Cellularity:  The lesion is relatively cellular with a mix of mononuclear cells (fibroblasts or histiocytes) interspersed between the giant cells. This cellularity is consistent with the inflammatory nature of CGCG.  Bone Destruction:  Both images suggest areas of bone resorption, as seen in the irregular and somewhat disrupted bone trabeculae, which is typical of CGCG as it causes local bone destruction.  These features collectively point towards Central Giant Cell Granuloma, which is commonly seen in the jawbones, and is typically a benign, locally aggressive lesion that may require surgical treatment. | Based on the provided hematoxylin-and-eosin (H&E) images, here is an analysis of the lesion in question:  Histopathological Diagnosis:  The most likely diagnosis based on the given images is osteomyelitis.  Key Microscopic Features:  Bone Resorption: The first image shows areas of bone resorption, which is characteristic of osteomyelitis. The bone is undergoing destruction, which appears as irregular, lacelike patterns with areas of lysis.  Inflammatory Cells: The second image reveals the presence of inflammatory cells, including multinucleated giant cells, plasma cells, and lymphocytes. This is a typical feature seen in chronic inflammatory processes such as osteomyelitis.  Bone Formation and Fibrosis: Some areas in both images show the presence of newly formed bone, along with fibrosis. This reflects the body’s attempt at healing, which is seen in chronic cases of osteomyelitis as it progresses through the stages of inflammation and repair.  Abscess Formation: The second image shows a central area with a red-stained zone and inflammation, which could represent an abscess or areas of purulent material, often seen in osteomyelitis due to bacterial infection.  These features collectively support the diagnosis of osteomyelitis, which involves both bone destruction and the formation of granulation tissue and inflammatory infiltrates. |
| 17.42 | Diagnosis: Low-grade parosteal osteosarcoma; Key Microscopic Features: A sclerotic mass of atypical bone lies beneath surface epithelium, replacing the normal cortex; broad seams of bone are interconnected with relatively hypocellular fibrovascular tissue in between, and bone is rimmed by a single layer of minimally atypical osteoblasts with spindle cells in the stroma. | **Upon reviewing the images you provided, the lesion in question appears to be central giant cell granuloma (CGCG), a common nonodontogenic intraosseous tumor in the oral and maxillofacial region.**  **Key Microscopic Features Supporting this Diagnosis:**  **Multinucleated Giant Cells: The presence of multinucleated giant cells scattered throughout the lesion is one of the defining features of CGCG. These cells are typically larger and have several nuclei.**  **Fibrous Stroma: The background of the lesion is composed of a loose, fibrous stroma with collagen fibers, which is common in CGCG.**  **Bone Resorption: Some images show areas where bone resorption is evident, characterized by the presence of lacunae or areas of bone loss, often seen with multinucleated giant cells that resorb bone.**  **Osteoid and New Bone Formation: Some of the images hint at areas of osteoid and the formation of new bone, which can be a feature of CGCG, as it often leads to bone remodeling.**  **Absence of Aggressive Malignancy Features: No clear malignant features, such as atypical mitotic activity, nuclear pleomorphism, or necrosis, are observed, which is consistent with a benign process like CGCG.**  **This lesion is most often diagnosed based on clinical and radiographic findings combined with these characteristic histopathological features.** | **Thank you for providing the histopathological images. After analyzing the series of images, I can offer the following diagnosis:**  **Single Most Histopathological Diagnosis:**  **Central Giant Cell Granuloma (CGCG)**  **Key Microscopic Features Justifying the Diagnosis:**  **Multinucleated Giant Cells: There is the presence of multinucleated giant cells scattered throughout the lesion, which is a hallmark of CGCG. These cells are large and contain multiple nuclei.**  **Cellular Stromal Tissue: The lesion is composed of a relatively cellular stroma with fibroblastic activity, often accompanied by a variable degree of collagenization. The stromal cells are arranged in a loose, myxoid background, which helps support the diagnosis of CGCG.**  **Hemorrhagic Areas: In some sections, areas of hemorrhage and blood-filled spaces are seen. These blood-filled spaces are typically found in CGCG and are indicative of a vascular component within the lesion.**  **Bordering of Bone: In some images, there is evidence of bone resorption and reactive bone formation at the periphery of the lesion, which further supports the diagnosis of CGCG, as this tumor is known to cause localized bone destruction.**  **Presence of Osteoid-like Structures: The presence of osteoid-like material in certain areas, which is typically seen in CGCG, suggests that the lesion may have osteoclastic activity contributing to the bone destruction.**  **These histopathological features are consistent with a diagnosis of Central Giant Cell Granuloma, a benign, non-odontogenic lesion that commonly affects the jaws and often presents with local bone resorption and reactive changes.** |
| 17.43 | Diagnosis: Synovial chrondromatosis; Key Microscopic Features: Nodules of cartilage of varying sizes; mature hyaline cartilage with chondrocytes and dystrophic calcifications are seen, and chondrocytes exhibit mild nuclear atypia and binucleation with subtle dystrophic calcification. | The provided images depict a lesion in the group of nonodontogenic intraosseous tumors and inflammatory/reactive conditions of oral and maxillofacial pathology. Based on the characteristics observed in the images, here is the analysis:  Most Likely Histopathological Diagnosis:  Giant Cell Tumor of Bone (GCTB)  Key Microscopic Features Supporting the Diagnosis:  Multinucleated Giant Cells:  The hallmark feature of the lesion is the presence of multinucleated giant cells, which are scattered throughout the lesion. These giant cells typically have multiple nuclei and are involved in the resorption of bone tissue.  Osteoid Formation and Bone Resorption:  The areas in the images show resorption of bone with regions of osteoid (immature bone matrix) formation, which are indicative of the aggressive nature of the tumor. The bone appears to be replaced by areas of cellular activity.  Mononuclear Cells:  There is a prominent presence of mononuclear stromal cells, which are typically seen in the background of giant cell tumors of bone. These cells have an oval or spindled shape and contribute to the neoplastic nature of the lesion.  Vascular Channels:  The images show a vascularized stroma, with prominent blood vessels, which is a feature often associated with GCTB. These vascular channels are irregularly shaped and are crucial for differentiating this lesion from other nonodontogenic tumors.  Lack of Mineralization:  While there is some osteoid formation, the lesion does not show extensive mineralization, which further supports the diagnosis of GCTB over other tumors that produce more mature bone.  These microscopic features, when taken together, strongly suggest that the lesion in question is a Giant Cell Tumor of Bone (GCTB), which is a benign but locally aggressive tumor that commonly affects the long bones but can also occur in the maxilla or mandible. | Upon reviewing the three histopathological images of a lesion within the nonodontogenic intraosseous tumors and inflammatory/reactive conditions group of oral and maxillofacial pathology, the diagnosis that comes to mind is central giant cell granuloma (CGCG).  Key Microscopic Features:  Multinucleated Giant Cells:  The most prominent feature across all magnifications is the presence of numerous multinucleated giant cells scattered within a background of spindle-shaped stromal cells. These giant cells often appear near the borders of the lesion and sometimes in clusters, which is characteristic of CGCG.  Hemosiderin Deposition:  The presence of hemosiderin deposits (as seen in some of the areas in the second and third images) is a common finding in CGCG. This indicates previous hemorrhage within the lesion, a feature often associated with this pathology.  Spindle-shaped Stromal Cells:  The background contains a fibroblastic or stromal cell component that is often seen in CGCG, with occasional foci of more cellular areas and lesser inflammatory infiltrates.  No Osseous Formation:  Unlike some other lesions, CGCG does not show any evidence of bone formation, which can be ruled out based on these images, as no mature bone or osteoid formation is visible.  These key features, particularly the multinucleated giant cells and the presence of hemosiderin deposits, strongly support the diagnosis of central giant cell granuloma (CGCG). |
| 17.44 | Diagnosis: Chondromyxoid fibroma; Key Microscopic Features: Lobular proliferation of spindle and stellate cells within a myxoid stroma separated by hypercellular septa; areas with more cellular chondroblastoma-like regions containing large polygonal cells with eosinophilic cytoplasm and multinucleated giant cells are observed. | Based on the two hematoxylin and eosin (H&E) images provided, the most likely histopathological diagnosis for this lesion appears to be Central Giant Cell Granuloma (CGCG).  Key Microscopic Features:  Multinucleated Giant Cells: The images show the presence of numerous multinucleated giant cells scattered within a background of spindle-shaped stromal cells. The multinucleated cells are a hallmark of CGCG, often seen in reactive lesions.  Granulation Tissue: In the images, there is an abundant amount of granulation tissue, which is characterized by an inflammatory infiltrate of lymphocytes and plasma cells, indicating the lesion’s inflammatory component. This is typical of CGCG.  Spindle-shaped Stromal Cells: The lesion contains a prominent proliferation of spindle-shaped stromal cells, a feature commonly associated with CGCG.  Lack of Neoplastic Features: There is no clear evidence of malignancy, such as significant cellular atypia or mitotic activity, further supporting the diagnosis of a benign lesion like CGCG.  Vascularity: The lesion exhibits high vascularity with some blood vessels, which is a common feature in CGCG, adding to the inflammatory/reactive nature of the lesion. | The two provided hematoxylin-and-eosin (H&E) images of the same lesion suggest a diagnosis within the group of nonodontogenic intraosseous tumors and inflammatory/reactive conditions of the oral and maxillofacial region. Based on the microscopic features observed, the most likely diagnosis is Central Giant Cell Granuloma (CGCG).  Key Microscopic Features Justifying the Diagnosis:  Multinucleated Giant Cells: Both images show numerous multinucleated giant cells scattered throughout the lesion. This is a hallmark feature of CGCG, which is characterized by the presence of large, multinucleated osteoclast-like giant cells within a background of fibrovascular stroma.  Fibrovascular Stroma: The first image shows a background with a loose, fibrovascular stroma, which supports the diagnosis. CGCG often presents with this type of stroma, interspersed with numerous smaller spindle-shaped cells.  Hematopoietic Cells and Erythrocytes: The second image also suggests the presence of hematopoietic cells and areas with erythrocytes, which may appear as scattered red blood cells due to the vascular nature of the lesion. This feature is also frequently seen in CGCG due to its vascularity.  No Evidence of Neoplasia: Importantly, there is an absence of overt cellular atypia or malignancy in the images, which supports a benign process like CGCG.  These features together point to Central Giant Cell Granuloma (CGCG), a benign tumor-like lesion that is frequently observed in the jawbones and often presents with osteoclastic giant cells within a richly vascularized stroma. |
| 17.46 | Diagnosis: Chondrosarcoma, grade 1; Key Microscopic Features: Sheets of cartilage exhibit ossification; hyaline cartilage with ossification is present, with atypical chondrocytes seen in ossified cartilage, and atypical chondrocytes with binucleation and slight nuclear pleomorphism are observed. | Based on the hematoxylin and eosin (H&E) images you provided, they display features suggestive of an odontogenic keratocyst (OKC). Here’s an analysis of the key histopathological features:  Histopathological Diagnosis:  Odontogenic Keratocyst (OKC)  Key Microscopic Features:  Cystic Structure: The images show a well-defined cystic lesion with a thin, flattened epithelium lining the cyst lumen.  Multilayered Epithelium: The cyst wall is lined by a relatively thick, multilayered epithelium, characteristic of OKC. The epithelium is usually 6-10 layers thick, which can be seen in the images provided.  Parakeratosis: The lining exhibits parakeratosis, where the superficial epithelial cells retain their nuclei. This is a classic feature of OKC, helping differentiate it from other odontogenic cysts, which typically show orthokeratinized epithelium.  Corrugated Basal Layer: The basal layer of the epithelium is often hyperplastic and exhibits a characteristic "picket fence" appearance. This can be identified by the palisading arrangement of the basal cells in the images.  Thin Connective Tissue Wall: The cyst is surrounded by a thin layer of connective tissue, which appears scant in the images. This is indicative of the OKC's tendency to have a less reactive fibrous capsule.  Lumen with Fluid: The lumen of the cyst shows no evidence of solid tissue or calcification, which is common in OKCs that are filled with fluid or serous material. | Based on the images you provided, it appears that they show a lesion commonly associated with oral and maxillofacial pathology. The images depict typical characteristics seen in benign intraosseous tumors or reactive lesions. I will analyze the histopathological features observed in these high magnifications of the lesion.  Diagnosis:  The most likely histopathological diagnosis is Central Giant Cell Granuloma (CGCG), which belongs to the group of non-odontogenic intraosseous tumors.  Key Microscopic Features:  Multinucleated Giant Cells: Prominent, large multinucleated osteoclast-like giant cells are distributed throughout the lesion, a hallmark feature of CGCG. These cells are scattered among the lesion.  Stromal Cells: The tumor shows a background of oval to spindle-shaped stromal cells that are often arranged in a loose, fibrous matrix.  Hemorrhage and Cystic Areas: Areas of hemorrhage are evident, with regions of cystic degeneration in some places.  Bone Resorption: The presence of resorbed bone and foci of new bone formation is also observed. This is characteristic of CGCG, which is often associated with bone destruction.  Justification for the Diagnosis:  Giant Cells: The presence of numerous multinucleated giant cells dispersed in a fibrous stroma strongly suggests a diagnosis of CGCG.  Reactive or Inflammatory Nature: The overall histology shows features of bone resorption, a typical manifestation of CGCG's local aggressive behavior, though it is benign.  Cystic Areas and Hemorrhage: These features are consistent with CGCG, where hemorrhage and cyst-like areas are often seen.  In conclusion, these images most likely represent Central Giant Cell Granuloma based on the noted microscopic features. |
| 17.48 | Diagnosis: Mesenchymal chondrosarcoma; Key Microscopic Features: Foci of small hyperchromatic cells adjacent to chondroid matrix with spindle cells and dilated vessels are noted (hemangiopericytoma pattern); atypical chondrocytes with pleomorphic and hyperchromatic nuclei in chondroid matrix; hyperchromatic small cells cluster around dilated vessels. | The provided images appear to be histopathological slides of a lesion in the group of non-odontogenic intraosseous tumors and inflammatory/reactive conditions within oral and maxillofacial pathology. Based on the images provided, I will conduct an analysis of the microscopic features to provide the most likely histopathological diagnosis along with key supporting features.  Histopathological Diagnosis:  The diagnosis based on these images is most likely Central Giant Cell Granuloma (CGCG), given the observed characteristics across the different magnifications.  Key Microscopic Features:  Multinucleated Giant Cells:  The presence of numerous multinucleated giant cells scattered throughout the lesion, which are typical of CGCG. These cells appear as large, multi-nucleated entities, which are a hallmark feature of this condition.  Spindle-shaped Stromal Cells:  The stroma of the lesion is composed of spindle-shaped fibroblasts, which are seen in various stages of differentiation, typically arranged in a collagenous matrix.  Hemorrhage and Hemosiderin Deposition:  Areas of hemorrhage are often observed in CGCG, along with hemosiderin deposits (seen as brownish pigment), reflecting previous bleeding within the lesion.  Absent Necrosis and Inflammation:  There is a lack of significant inflammatory cells or necrosis within the lesion, which helps differentiate CGCG from other inflammatory/reactive lesions like the peripheral giant cell granuloma.  Lack of Odontogenic Features:  There are no signs of odontogenic epithelium or calcifications, which helps rule out odontogenic tumors like ameloblastoma or keratocystic odontogenic tumor (KCOT).  The combination of these features strongly supports a diagnosis of Central Giant Cell Granuloma. | The provided images appear to be histopathological slides of a lesion within the category of non-odontogenic intraosseous tumors and inflammatory/reactive conditions of oral and maxillofacial pathology. Based on the cellular characteristics visible in the images, the most likely histopathological diagnosis would be ossifying fibroma.  Key microscopic features that justify the diagnosis:  Presence of fibrous stroma: The images show areas with prominent fibrous tissue, which is a characteristic feature of ossifying fibroma.  Well-formed trabeculae of bone or cementum-like material: The tumor's stroma often includes areas of mineralized tissue, resembling bone or cementum. The trabeculae observed in these images suggest this type of mineralized material.  Cellular nature: There are varying degrees of cellularity, with some regions showing more densely packed cells and others appearing more sparse. This variable cellularity is typical in ossifying fibromas.  Lack of an inflammatory response: The absence of significant acute inflammation or a high degree of chronic inflammatory infiltrates helps rule out purely reactive or inflammatory lesions, focusing the diagnosis more on a benign neoplastic condition.  Prominent blood vessels and scattered giant cells: These features can also be seen in ossifying fibromas, though they may not be prominent in every case.  These microscopic findings strongly suggest ossifying fibroma as the diagnosis. This benign tumor typically involves the jawbones and can cause expansion of the bone due to the slow growth of the fibrous and mineralized components. |
| 17.50 | Diagnosis: Langerhans cell histiocytosis; Key Microscopic Features: Sheets of pale epithelioid cells are present; Langerhans cells have abundant pale cytoplasm and reniform (“coffee bean”) nuclei, with many eosinophils in the sample. | Based on the provided hematoxylin-and-eosin (H&E) images of a lesion, it appears that the histopathological features align with a diagnosis of central giant cell granuloma (CGCG), which is a benign lesion that falls under the group of non-odontogenic intraosseous tumors and inflammatory/reactive conditions.  Key microscopic features observed:  Multinucleated Giant Cells: The images prominently show large multinucleated giant cells scattered throughout the lesion, which are a hallmark feature of central giant cell granuloma. These cells often have a characteristic arrangement and appear as large cells with multiple nuclei.  Background of Spindle-Shaped and Mononuclear Cells: The tumor stroma contains spindle-shaped or ovoid mononuclear cells that surround the giant cells. These cells are typically fibroblasts or histiocytes, contributing to the inflammatory reaction seen in CGCG.  Bone Resorption: There is evidence of bone resorption, which can be seen as areas with a lack of trabecular bone, where osteoclastic activity is ongoing. This resorptive activity leads to the characteristic radiolucency often seen in CGCG.  Hemorrhagic Areas: The lesion demonstrates areas of hemorrhage, which appear as regions of pink/red discoloration in the tissue due to blood vessel involvement. This hemorrhagic pattern is another important feature of CGCG.  The combination of multinucleated giant cells, reactive stromal elements, and bone resorption strongly supports the diagnosis of central giant cell granuloma. | Based on the provided hematoxylin-and-eosin (H&E) images, the diagnosis is suggestive of a Granulomatous Inflammation, likely associated with an inflammatory or reactive condition in the oral and maxillofacial region. Here's an analysis:  Diagnosis:  Granulomatous Inflammation  Key Microscopic Features:  Presence of Granulomas: The first image displays an organized tissue architecture with clusters of inflammatory cells forming a granulomatous pattern. These granulomas are characterized by central areas of necrosis (pinkish hue), surrounded by a mix of inflammatory cells, predominantly macrophages. These clusters of cells, which are a response to a chronic inflammatory stimulus, suggest a granulomatous process.  Giant Cells: The second image highlights multinucleated giant cells, a hallmark of granulomatous inflammation. These cells appear as large, multi-nucleated entities, which often arise in the setting of chronic inflammation as a response to persistent foreign bodies or infection.  Chronic Inflammatory Infiltrate: Both images reveal an abundant inflammatory infiltrate, predominantly composed of lymphocytes and plasma cells, with occasional histiocytes. This chronic infiltrate is another key feature of granulomatous reactions.  Fibrosis: The pink areas within the lesion indicate fibrosis or scar tissue formation, which is commonly seen in chronic inflammatory conditions as the body attempts to wall off the irritant or pathogen.  Conclusion:  The histopathological findings of granulomas, giant cells, chronic inflammatory infiltrate, and fibrosis strongly suggest a diagnosis of Granulomatous Inflammation. The granulomas can be associated with various conditions, such as infections (e.g., tuberculosis, fungal infections), foreign body reactions, or chronic inflammatory responses. Further clinical correlation and microbiological studies would help determine the exact cause. |
| 17.51 | Diagnosis: Erdheim-Chester disease of the mandible; Key Microscopic Features: The lesion contains sheets of foamy macrophages along with fibrocollagenous tissue, observed at high magnification. | The two images provided appear to represent a lesion that might belong to the category of Nonodontogenic Intraosseous Tumors or Inflammatory/Reactive Conditions. Based on the provided magnifications and histopathological features, I will provide an analysis of the most likely diagnosis and the microscopic features seen in the images.  Single Most Histopathological Diagnosis:  The lesion most likely represents Fibrous Dysplasia.  Key Microscopic Features:  Image 1 (Low Magnification):  The lesion exhibits a fibrous stroma that is infiltrated with spindle-shaped cells and some fibrous tissue.  Areas of the lesion show a loosely organized matrix, which is characteristic of fibrous dysplasia. This type of matrix often lacks distinct cell boundaries, and there is an absence of well-formed bone trabeculae, further supporting a non-odontogenic, non-neoplastic process.  The lesion appears to be spanning a large area with little evidence of encapsulation, suggesting a benign but expansive process.  Image 2 (High Magnification):  The spindle-shaped fibroblasts are densely packed, with a variable cellular density, which is characteristic of the fibrous tissue seen in fibrous dysplasia.  There are irregular bone trabeculae (woven bone) embedded within the fibrous stroma, indicative of the ossifying nature of fibrous dysplasia. These trabeculae are often disorganized and poorly defined in structure.  The presence of stromal cells with spindle morphology and some osteoblastic activity is consistent with fibrous dysplasia.  There is an absence of inflammation or giant cells, which helps rule out other reactive or inflammatory conditions like cemento-ossifying fibroma or central giant cell granuloma. | Based on the provided hematoxylin-and-eosin (H&E) images, I will analyze the lesions. After examining the images at different magnifications, here is the histopathological diagnosis and the key features that support it:  Most Likely Histopathological Diagnosis:  The lesion appears to be a granulomatous inflammation, possibly associated with chronic inflammation or reactive changes in the oral and maxillofacial region.  Key Microscopic Features:  Lesion Characteristics:  The first image (low magnification) shows a well-defined lesion composed of a dense cellular infiltrate.  The second image (high magnification) highlights an inflammatory infiltrate with granulomatous features, including numerous multinucleated giant cells.  Granulomatous Inflammation:  Presence of epithelioid macrophages, characterized by their large, pale cytoplasm, which are indicative of a chronic inflammatory response.  Multinucleated giant cells within the infiltrate suggest a granulomatous pattern, which is a hallmark of certain inflammatory lesions.  The lesion has a well-organized structure typical of granulomas, with central necrosis possibly observed in the surrounding areas.  Fibrous Stroma:  The surrounding stroma shows fibrous tissue with possible scar tissue formation, commonly seen in chronic inflammatory conditions.  Absence of Neoplastic Features:  There is no clear evidence of atypical cells or neoplastic features (like increased mitotic figures or cellular pleomorphism), which rules out malignancy. |
| 17.53 | Diagnosis: Ewing sarcoma; Key Microscopic Features: Small round cells infiltrate the bone trabeculae, with some appearing as fragments of sequestra. These cells exhibit pale to clear cytoplasm, round nuclei, dispersed chromatin, and inconspicuous nucleoli. | The provided hematoxylin-and-eosin (H&E) images show a lesion in the context of non-odontogenic intraosseous tumors and inflammatory/reactive conditions in oral and maxillofacial pathology. Upon review of the provided images, the most likely histopathological diagnosis is:  Diagnosis: Giant Cell Tumor of Bone (GCTB)  Key Microscopic Features:  Multinucleated Giant Cells: The presence of numerous multinucleated giant cells is a hallmark of this lesion, visible in all images. These cells have large, irregular, and multiple nuclei, often surrounded by osteoid or bone matrix.  Osteoid Formation: In the second image, areas of bone formation (osteoid) can be observed, consistent with the lesion's tendency to involve bone. This is a common finding in GCTB, where the tumor can lead to bone resorption and formation.  Stromal Cells: The stromal cells appear mononuclear with a spindle or oval shape, as seen in the third image. These cells are generally considered to be neoplastic and contribute to the overall growth of the tumor.  Reactive Bone Changes: In the first image, the bone appears remodeled with areas of resorption and new bone formation, which is common in giant cell tumors of bone, especially in the periphery of the lesion. | **Based on the provided hematoxylin-and-eosin stained images, I will analyze the histopathological characteristics to provide the most likely diagnosis for the lesion in question. I'll focus on key features that stand out in these magnified images to arrive at a conclusion.**  **Diagnosis:**  **The lesion appears to be central giant cell granuloma (CGCG), which falls under the category of nonodontogenic intraosseous tumors in oral and maxillofacial pathology.**  **Key Microscopic Features:**  **Presence of Multinucleated Giant Cells:**  **The images clearly show numerous multinucleated giant cells scattered throughout the tissue. These cells are a hallmark feature of CGCG. These giant cells are typically distributed within a background of a vascular stroma.**  **Hematopoietic Stroma with Hemorrhagic Areas:**  **The lesion has a vascularized stroma with some evidence of hemorrhagic regions, which is consistent with CGCG. The cells appear to be embedded in a loosely arranged stroma that often has areas of bleeding or hemosiderin deposition, contributing to the lesion's histopathologic appearance.**  **Osteoid or Bone Resorption:**  **In some areas, there is clear evidence of bone resorption or the presence of osteoid material around some of the giant cells. This feature is typically seen in CGCG, where osteoclast-like cells cause bone destruction.**  **Cellular Proliferation:**  **The images show a high degree of cellular proliferation, particularly around the giant cells. The presence of mononuclear spindle-shaped cells interspersed with the giant cells is consistent with CGCG.**  **These features, especially the multinucleated giant cells and the vascular stroma, strongly suggest a central giant cell granuloma as the diagnosis. This lesion typically presents with a combination of bone resorption and vascularized stroma, with the giant cells often observed in clusters.** |
| 17.54 | Diagnosis: Rhabdomyosarcoma with TFCP2 rearrangement; Key Microscopic Features: A fascicular and storiform arrangement of predominantly spindle-shaped cells is seen, with some areas showing intersecting fascicles. Multiple atypical mitoses are present in fascicles composed of spindle cells. The tissue also contains a mixed population of spindle, epithelioid, and round cells, with epithelioid and round cells located in the lower part of the image. Nuclear pleomorphism and frequent mitotic activity are observed, with some cells showing multiple nucleoli. Rare woven bone spicules are infiltrated by neoplastic cells, and there may be residual bone or dystrophic bone formation, characterized by osteoclasia. | The provided hematoxylin and eosin-stained images of the lesion from the NONODONTOGENIC INTRAOSSEOUS TUMORS AND INFLAMMATORY/REACTIVE CONDITIONS group show the following key histopathological features:  Histopathological Diagnosis:  Central Giant Cell Granuloma (CGCG)  Key Microscopic Features Justifying the Diagnosis:  Multinucleated Giant Cells:  Numerous multinucleated giant cells are scattered throughout the lesion, seen particularly in the higher magnification images.  These giant cells are typically large with multiple nuclei and are a hallmark feature of CGCG.  Fibroblastic Stroma:  The background stroma is composed of a cellular fibroblastic proliferation.  This spindle cell component is arranged in a loose connective tissue matrix.  Hemorrhage and Hemosiderin Deposition:  Areas with extravasated red blood cells and hemosiderin pigment are noted, indicative of prior hemorrhage.  These are commonly associated with CGCG due to its reactive and sometimes aggressive nature.  Reactive Bone Formation:  Some images show reactive bone trabeculae or osteoid formation within the lesion.  This reactive bone is a feature seen in giant cell lesions.  Absence of Odontogenic Epithelium:  The lesion lacks any epithelial components, ruling out odontogenic tumors.  This supports the diagnosis of a nonodontogenic lesion like CGCG.  Inflammatory Cells:  Mild chronic inflammatory infiltrate may be present, supporting the reactive nature of the lesion. | The images you uploaded appear to be histological slides stained with hematoxylin and eosin (H&E) of a lesion from the group of non-odontogenic intraosseous tumors and inflammatory/reactive conditions in oral and maxillofacial pathology. Let's analyze the key features to arrive at a diagnosis.  Single Most Likely Diagnosis:  Based on the images, the lesion could most likely be central giant cell granuloma (CGCG), which is a non-odontogenic, benign intraosseous lesion.  Key Microscopic Features:  Fibrocellular Stroma: There is a fibrocellular stroma seen in the images, which is typical of CGCG.  Multinucleated Giant Cells: The presence of multinucleated giant cells scattered throughout the lesion is a hallmark feature of CGCG. These giant cells typically have more than three nuclei.  Bone Resorption: Evidence of bone resorption and trabeculae is often seen around the giant cells, indicating the aggressive nature of the lesion in terms of bone remodeling.  Mononuclear Cells: Mononuclear cells surrounding the giant cells are frequently seen. These cells are smaller and contribute to the lesion's cellular appearance.  Histological Pattern: The lesion is arranged in a slightly loose, irregular pattern that contributes to its reactive nature. Some images may reveal a more well-organized tissue structure while others are more diffuse.  Lack of Atypia: There is no significant cytologic atypia (such as bizarre mitotic figures or abnormal nuclear shapes), which suggests a benign lesion.  These features are consistent with central giant cell granuloma (CGCG), which typically occurs in the jawbones and can present as a locally aggressive but benign lesion. |

HEMATOLYMPHOID TUMORS OF THE ORAL CAVITY

| **Fig_no** | **Ground truth** | **GPT 4o answer** | **o3 answer** |
| --- | --- | --- | --- |
| 18.2 | Diagnosis: Diffuse large B-cell lymphoma (DLBCL); Key Microscopic Features: The infiltrate exhibits a diffuse growth pattern with loss of tissue structure. Higher magnification reveals large tumor cells with round nuclei and varying nucleoli. | The images provided show tissue sections stained with hematoxylin and eosin (H&E) at different magnifications, likely representing a hematolymphoid tumor from the oral cavity. Based on the histological features observed, the most likely diagnosis is Lymphoma, specifically Non-Hodgkin Lymphoma (NHL), given the following microscopic features:  Key Microscopic Features:  Monomorphic Cell Population:  The tumor is composed predominantly of a homogenous population of small, round, lymphoid cells with relatively uniform nuclear size. This is characteristic of lymphoma, as the neoplastic lymphoid cells generally lack the pleomorphism typically seen in reactive or inflammatory processes.  Nuclear Features:  The nuclei of the cells are generally round or oval, with fine chromatin and small nucleoli. This uniformity of nuclear characteristics is a feature of lymphoma, where the neoplastic cells maintain their lymphoid characteristics.  Absence of Significant Necrosis or Atypia:  The images do not show significant necrosis, large atypical cells, or mitotic activity, which would suggest a more aggressive form of lymphoma. The cells appear relatively mature, and there is a lack of highly atypical or undifferentiated cells.  Cytoplasmic Features:  The cytoplasm of the cells appears scant, and some cells show clear areas, which might indicate a slight cytoplasmic differentiation typical of certain lymphoma subtypes.  Sparse Background:  The background is relatively clean, without the presence of a significant inflammatory infiltrate, which further supports the diagnosis of lymphoma over a reactive process or an inflammatory lesion.  These findings are consistent with a low-grade Non-Hodgkin Lymphoma (NHL) or potentially a B-cell lymphoma. The uniformity in cell size and nuclear characteristics points to a neoplastic lymphoid process rather than a reactive or infectious one.  A more specific subtype can be determined by immunohistochemical staining, which can provide further markers to classify the lymphoma (e.g., CD20, CD3, etc.). | Based on the hematoxylin-and-eosin (H&E) images provided, I will analyze the lesion and offer a histopathological diagnosis along with key features that justify it.  Diagnosis:  The lesion depicted appears to be a lymphoma, most likely of the B-cell type, based on the overall histopathological presentation.  Key Microscopic Features:  Monomorphic Population of Lymphoid Cells:  Both images show a uniform population of small to medium-sized cells with round nuclei and scant cytoplasm. The cells have hyperchromatic nuclei, and there is little variation in size, which is characteristic of a lymphoma. The lack of pleomorphism (variation in cell size and shape) supports this diagnosis.  Lack of Reactive Germinal Centers:  There is no evidence of reactive germinal centers or follicles, which would typically be seen in conditions like lymphoid hyperplasia or reactive lymphadenitis.  Intermediate Magnification:  At the higher magnification (second image), we see finer details, such as nuclear irregularities and some atypical features like nuclear indentations, which are often seen in lymphoma cells. These subtle features strengthen the diagnosis of lymphoma rather than a benign reactive process.  Absence of a Differentiated Architecture:  The tissue appears to lack the normal nodular or follicular architecture that would be seen in reactive lymphoid tissues, further supporting the diagnosis of a malignant lymphoma.  In summary, based on the uniformity of the lymphoid cells, the absence of reactive follicles, and the overall features observed, the lesion is most consistent with a diagnosis of non-Hodgkin lymphoma (likely of the B-cell type). Further immunohistochemical staining would be needed to confirm the exact subtype and differentiation. |
| 18.3 | Diagnosis: Plasmablastic lymphoma (PBL); Key Microscopic Features: Low-power view shows diffuse infiltration with almost complete tissue destruction. Some cases show plasmacytic features such as eccentrically placed nuclei, variable nucleoli, and abundant cytoplasm. Frequent mitotic figures are observed. | To make an accurate diagnosis based on the provided histopathological images, I will need to review both of them closely. I'll begin by analyzing the features visible in the images.  Please hold on for a moment while I examine the images.  I've displayed the two histopathological images. Based on these, I will now analyze them to identify the key features and provide the most likely diagnosis. Please hold on.  Upon reviewing the provided images, the following histopathological diagnosis is most likely:  Diagnosis: Lymphoma (likely B-cell lymphoma)  Key Microscopic Features:  Image 1 (Low Magnification):  The lesion appears to involve the tissue with a more diffuse growth pattern.  There are some areas with irregular, pale spaces indicative of necrosis or apoptotic cells.  The background shows a diffuse infiltration with a relatively uniform cellular population, which is typical for lymphoma.  Image 2 (High Magnification):  This image reveals a dense infiltration of cells with scant cytoplasm and high nuclear-to-cytoplasmic ratios.  The nuclei are round with prominent nucleoli, a characteristic of malignant lymphoid cells.  There are also small, dispersed nuclei that seem to be clustered, indicating a possible lymphoid structure or aggregation of lymphoma cells.  Justification for Diagnosis:  The presence of a monomorphic population of cells, high nuclear-cytoplasmic ratios, and lack of normal tissue architecture supports the diagnosis of lymphoma.  The appearance of necrotic areas and the lack of differentiation further suggest a neoplastic lymphoid lesion rather than an inflammatory process.  The features seen here are consistent with a diagnosis of B-cell lymphoma, which is a common form of lymphoma that can involve the oral cavity.  This is an initial interpretation based on the provided images, and further clinical correlation or immunohistochemical testing would be essential to confirm the exact type of lymphoma. | Based on the images provided, the most likely histopathological diagnosis appears to be Lymphoma.  Key Microscopic Features:  First Image (Low Magnification):  The lesion shows an overall irregular pattern with areas of scattered, large cellular aggregates and regions with a more diffuse pattern.  The presence of scattered atypical cells, which are larger and pleomorphic, surrounded by necrotic areas or inflammatory infiltrates.  Possible areas of vascular invasion or formation of abnormal tissue structures (e.g., residual lymphoid tissue).  Second Image (High Magnification):  The higher magnification shows dense cellular infiltration consisting predominantly of small to medium-sized lymphoid cells.  The cells display nuclear pleomorphism (varying shapes and sizes), which is typical for lymphoma.  Some cells have large nuclei with prominent nucleoli, indicative of high-grade lymphoma or an aggressive form of lymphoid malignancy.  Scattered apoptotic bodies (likely apoptotic tumor cells) can be observed, which are typical of lymphoma.  The background shows the disruption of normal tissue architecture, with many neoplastic lymphoid cells replacing normal tissue.  These features are consistent with non-Hodgkin lymphoma (NHL), a common type of lymphoma that often presents as a mass in various tissues, including the oral cavity, and is known for its infiltrative nature with a mixed population of lymphoid cells. |
| 18.4 | Diagnosis: Follicular lymphoma (FL); Key Microscopic Features: Low-power view reveals well-circumscribed nodules typical of FL. The low-grade FL nodules consist of cleaved centrocytes, distinct from the surrounding small round lymphocytes. High-grade FL is identified by greater than 15 centroblasts per high-powered field. Centroblasts are large cells with round to ovoid nuclei, open chromatin, and distinct nucleoli. | Based on the provided histopathological images of the lesion in the hematolymphoid tumors of the oral cavity group, I can identify the following diagnosis and microscopic features:  Single Most Histopathological Diagnosis:  The lesion appears to represent Lymphoma, specifically Non-Hodgkin lymphoma (NHL), based on the pattern of tissue architecture and cellular features seen in the images.  Key Microscopic Features:  Diffuse Architecture:  The images show a diffuse infiltrate of small to medium-sized lymphoid cells without the formation of a well-structured follicular pattern. This suggests a diffuse lymphoma rather than a follicular variant.  Monomorphous Population of Lymphoid Cells:  The tumor is composed predominantly of small, uniform lymphoid cells with round to slightly irregular nuclei and scant cytoplasm. This is characteristic of small B-cell lymphoma, but other lymphoma subtypes can also present with a similar pattern.  High Nuclear-to-Cytoplasm Ratio:  The lymphoid cells exhibit a high nuclear-to-cytoplasm ratio with darkly staining, round nuclei, and inconspicuous nucleoli. This is a typical finding in lymphomas.  Increased Mitotic Figures:  There are mitotic figures seen in the images, which suggests that the tumor cells are proliferating. This is consistent with malignancy and the aggressive nature of lymphoma.  Absence of Germinal Centers:  The images show a lack of germinal centers, which are characteristic of reactive lymphoid tissue. This absence supports the diagnosis of a neoplastic lymphoma rather than a reactive lymphoid proliferation.  Effacement of Normal Architecture:  The normal tissue architecture is effaced, and the tumor appears to have replaced the usual tissue structure. This is commonly seen in lymphomas and further suggests the malignant nature of the lesion.  In summary, the diagnosis is likely Non-Hodgkin lymphoma due to the diffuse and monomorphic nature of the infiltrate, the presence of increased mitotic activity, and the absence of germinal centers. Further immunohistochemical staining would be necessary for a more specific classification within the various subtypes of NHL. | Based on the provided hematoxylin-and-eosin (H&E) images, the most likely histopathological diagnosis is Non-Hodgkin lymphoma (NHL), specifically Follicular lymphoma.  Key Microscopic Features Supporting this Diagnosis:  Nodular Architecture:  The images show a well-formed, nodular architecture, which is typical for follicular lymphoma. These nodules resemble lymphoid follicles, which are consistent with lymphoma's presentation, particularly at the lower magnification in the first image.  Centrocytes and Centroblasts:  At higher magnification (as seen in images 2, 3, and 5), the predominant cell population consists of small lymphocytes (centrocytes) with larger, more blastic cells (centroblasts). The presence of both small and large cells is typical of follicular lymphoma, which exhibits a mixture of these two cell types in the follicles.  Diffuse Proliferation of Lymphoid Cells:  The images show a uniform, dense lymphoid infiltrate throughout the lesion, with no areas of necrosis or significant inflammation, which is typical of follicular lymphoma.  Absence of Peripheral Marginal Zone Lymphocytes:  The absence of a distinct marginal zone or the clear delineation of cells at the periphery supports the diagnosis of follicular lymphoma, as this type typically lacks a well-defined marginal zone.  Irregular Architecture of Lymphoid Follicles:  The follicles appear irregular, which can be indicative of follicular lymphoma, as neoplastic follicles in this disease may have distorted or disrupted architecture compared to reactive lymphoid follicles.  The presence of these features—nodular architecture, centrocytes, centroblasts, and a lack of a defined marginal zone—aligns with the diagnosis of Follicular lymphoma, a common type of Non-Hodgkin lymphoma. |
| 18.5 | Diagnosis: Extranodal marginal zone lymphoma of mucosa-associated lymphoid tissue (MALT); Key Microscopic Features: The infiltrate contains predominantly small lymphoid cells surrounding and invading glandular structures. The tumor cells often show plasmacytic differentiation. | Based on the provided histopathological images of a lesion from the "Hematolymphoid Tumors of the Oral Cavity" group, I can now analyze the features visible at different magnifications to provide a histopathological diagnosis.  Most Likely Diagnosis:  The images likely depict Hodgkin lymphoma or Non-Hodgkin lymphoma (NHL), specifically follicular lymphoma. However, the final diagnosis would be based on further immunohistochemical staining and clinical correlation.  Key Microscopic Features:  Cellular composition:  Monotonous population of lymphocytes: Both images show a predominance of small, round, and mature lymphocytes (seen as small dark nuclei with scant cytoplasm), which are characteristic of lymphoma. These lymphocytes exhibit a high nuclear-to-cytoplasmic ratio.  Nodular architecture:  The second image, with a higher magnification, demonstrates a nodular or follicular pattern, which is typically seen in follicular lymphoma or some variants of non-Hodgkin lymphoma. There are well-defined nodules of lymphocytes interspersed with areas of more diffuse lymphocytic infiltration.  Neoplastic and reactive features:  In some areas of both images, you can see the presence of reactive germinal centers or at least central areas where the lymphocytes are more activated, which can be seen in reactive lymphoid processes or early lymphoma stages.  Absence of significant necrosis:  There is no prominent necrosis visible in the images, which would help differentiate some high-grade lymphomas from lower-grade ones.  Lack of obvious Reed-Sternberg cells (specific to Hodgkin lymphoma):  The images show a more uniform cell population without Reed-Sternberg cells, which would be a key feature in Hodgkin lymphoma if present. | The images you provided appear to show a pathological specimen likely from the oral cavity. Based on the presentation of the hematoxylin and eosin (H&E) stained slides, the most likely diagnosis would be Non-Hodgkin Lymphoma (NHL), specifically a B-cell lymphoma.  Key Microscopic Features:  Lymphoid Cell Infiltrate: Both images reveal an abundant, dense population of small lymphoid cells with a high nucleus-to-cytoplasm ratio, which is typical for lymphoid neoplasms.  Monotonous Appearance of Cells: The cells appear quite uniform in size and shape, showing the typical feature of lymphoma, where there is little variation in the appearance of the cells.  Lack of Mature Architecture: The normal tissue architecture of the lymphoid tissue is disrupted, with the cells filling the tissue without any organized follicular structure, which suggests a malignancy such as lymphoma.  Irregular Nuclear Features: Some cells exhibit irregular nuclear contours and prominent nucleoli, which can be indicative of malignancy. In certain subtypes of B-cell lymphoma, such features are commonly seen.  Absence of Plasma Cells: The absence of plasma cells further supports the diagnosis of lymphoma over other lymphoproliferative disorders that are more reactive in nature, such as infectious mononucleosis.  These features, especially the presence of uniform, small lymphoid cells with disrupted architecture and lack of reactive changes, strongly suggest a diagnosis of Non-Hodgkin B-cell lymphoma. |
| 18.6 | Diagnosis: Mantle cell lymphoma (MCL); Key Microscopic Features: The infiltrate consists of a diffuse population of small lymphoid cells. Scattered mitotic figures are noted, with an epithelioid histiocyte near the field's center. The lymphoid cells display irregular nuclear contours. | The provided images appear to show a pathological specimen of hematolymphoid origin, specifically associated with the oral cavity. Based on the features visible in the hematoxylin and eosin (H&E) stained images, here's the analysis:  Diagnosis:  The most likely diagnosis for the lesion observed in these images is Non-Hodgkin Lymphoma (NHL), specifically Diffuse Large B-cell Lymphoma (DLBCL), given the histopathological characteristics present.  Key Microscopic Features:  Cellularity: Both images demonstrate a high cellularity, with a predominant population of large, atypical cells.  Large, Pleomorphic Cells: The cells are relatively large and irregular in shape. Some cells appear to have prominent nucleoli, which is characteristic of lymphoma, particularly DLBCL.  Nuclear Features: The nuclei of these cells are irregular, with some cells showing nuclear folding or indentation. The chromatin is dispersed, and nucleoli are conspicuous.  Scattered Mitotic Figures: There are several mitotic figures observed in both images, indicating active proliferation, which is typical of aggressive lymphomas like DLBCL.  Background Stroma: The background stroma appears to be scant, and there is no significant fibrosis, which is typically seen in certain other tumor types but not in DLBCL.  Lack of Well-Defined Nodal Architecture: In these images, the tissue appears diffuse with no distinct nodal architecture, which is indicative of a non-Hodgkin lymphoma pattern, as opposed to other tumors where tissue architecture is more organized.  Occasional Necrosis: Some focal areas of necrosis are also evident, which can be seen in high-grade lymphoma, including DLBCL.  These features are consistent with Diffuse Large B-cell Lymphoma (DLBCL), a common form of non-Hodgkin lymphoma, which often presents as a rapidly growing mass in lymphoid tissues but can also involve extranodal sites, including the oral cavity. | The provided images of hematoxylin-and-eosin stained slides suggest a lesion from the group of hematolymphoid tumors of the oral cavity. Based on the features presented, here’s the analysis:  Most Likely Histopathological Diagnosis:  Diffuse Large B-cell Lymphoma (DLBCL)  Key Microscopic Features:  Cellularity: Both images demonstrate a highly cellular tissue, which is typical of a lymphoid tumor. The density of the cellular population suggests an aggressive tumor like DLBCL.  Large, Irregular Nuclei: The cells exhibit large, irregular nuclei with prominent nucleoli. This is characteristic of lymphoma, particularly of the B-cell origin as seen in DLBCL.  Nuclear Pleomorphism: There is marked nuclear pleomorphism, with some cells appearing larger or smaller than others, a hallmark of aggressive lymphoma.  Mitotic Figures: Multiple mitotic figures are visible in both images, indicating high cell turnover, which is typical in rapidly growing tumors like DLBCL.  Cytoplasm and Cell Borders: The cells appear to have scant cytoplasm and poorly defined cell borders, common in lymphoma cells.  Vascular Proliferation: The images show areas where blood vessels are prominent, often seen in rapidly growing tumors due to increased angiogenesis.  These histopathological features, combined with the high mitotic rate and pleomorphic appearance of the tumor cells, strongly support a diagnosis of Diffuse Large B-cell Lymphoma (DLBCL), which is the most common type of non-Hodgkin lymphoma and can involve the oral cavity. |
| 18.7 | Diagnosis: Chronic lymphocytic leukemia/small lymphocytic lymphoma (CLL/SLL); Key Microscopic Features: The infiltrate consists of small lymphoid cells. A characteristic feature is the presence of irregularly shaped, pale-staining aggregates of prolymphocytes and paraimmunoblasts, or proliferation centers. The CLL/SLL cells are small with clumped chromatin and small nucleoli, while the cells in proliferation centers are larger with open chromatin and prominent nucleoli. | Based on the images provided, I will analyze them to determine the histopathological diagnosis and highlight key microscopic features.  Histopathological Diagnosis:  The images suggest the lesion is likely non-Hodgkin lymphoma (NHL), particularly a B-cell lymphoma, as it shows characteristic features typically observed in lymphoid neoplasms.  Key Microscopic Features:  Monotonous Cell Population:  The cells in all the images appear uniform, which is typical for lymphoma. There is a lack of significant variation in cell morphology, and the cells have a similar size and shape.  Large, Prominent Nuclei:  The cells in the images display large, round, or oval nuclei with a prominent nucleolus. This is often indicative of the atypical lymphoid cells seen in lymphoma.  Clumped Chromatin:  The chromatin within the nuclei appears irregularly clumped, a feature often associated with malignancy, especially lymphomas.  Lack of Background Inflammation:  There is a sparse inflammatory infiltrate surrounding the tumor cells, which supports the diagnosis of lymphoma, where the background usually lacks a prominent inflammatory response seen in reactive conditions.  Predominance of Small to Medium-Sized Lymphoid Cells:  The images show predominantly small to medium-sized lymphoid cells with minimal cytoplasm, which is consistent with B-cell lymphoma, particularly follicular lymphoma or mantle cell lymphoma.  In conclusion, the lesion appears to represent a B-cell lymphoma, and the images show the typical features of a lymphoid neoplasm, including monotonous lymphoid cells with atypical nuclear features, consistent with a diagnosis of non-Hodgkin lymphoma (NHL). | The provided images, featuring hematoxylin-and-eosin (H&E) staining at various magnifications, show a lesion typical of a hematolymphoid tumor of the oral cavity.  Histopathological Diagnosis:  The most likely diagnosis from the images is Non-Hodgkin Lymphoma (NHL), particularly the diffuse large B-cell lymphoma (DLBCL) subtype.  Key Microscopic Features:  Morphology of the Cells:  The images show large, pleomorphic cells with distinct, round nuclei and prominent nucleoli. These features are characteristic of malignant lymphoid cells.  The cells are predominantly lymphoid in origin and display an abnormal size variation, with some having irregular nuclear contours and scattered chromatin.  Cellularity:  The lesion is highly cellular, a feature commonly seen in lymphomas, where there is a predominance of malignant lymphoid cells.  There is marked nuclear atypia, with cells showing a high mitotic activity, indicative of an aggressive tumor.  Stroma:  The stroma in the images appears loose and reactive, which is often seen in aggressive lymphomas like DLBCL.  Infiltration Pattern:  There is a diffuse pattern of cell infiltration, without any distinct follicular or nodular architecture, which is consistent with diffuse large B-cell lymphoma (DLBCL).  The neoplastic cells are scattered throughout the tissue, and no well-formed lymphoid follicles are visible.  Absence of Classic Features of Hodgkin Lymphoma:  The absence of Reed-Sternberg cells, which are characteristic of Hodgkin lymphoma, supports the diagnosis of non-Hodgkin lymphoma.  Justification for Diagnosis:  The prominent features of large, atypical, and pleomorphic lymphoid cells, along with the absence of normal lymphoid architecture, and the diffuse infiltration pattern, strongly suggest diffuse large B-cell lymphoma (DLBCL), a common type of non-Hodgkin lymphoma.  This diagnosis is supported by the histological findings visible across the different magnifications. |
| 18.8 | Diagnosis: Burkitt lymphoma; Key Microscopic Features: Low-power evaluation shows a diffuse infiltrate of intermediate-sized cells with tingible-body macrophages, creating a "starry sky" appearance. High-power view reveals basophilic nucleoli in lesional cell nuclei, with mitotic figures and apoptotic bodies. | Based on the provided hematoxylin-and-eosin (H&E) images, here's an analysis of the lesion observed in the context of hematolymphoid tumors of the oral cavity:  Diagnosis:  The lesion appears to be a Non-Hodgkin's lymphoma (specifically, a follicular lymphoma).  Key Microscopic Features:  Image 1 (Low magnification):  The tissue shows a dense, uniform population of small round cells, which are typical of lymphoid cells.  The overall architecture is somewhat nodular, and there is an absence of significant differentiation within the lymphoid tissue.  The absence of necrosis or significant fibrosis, combined with the overall cellular uniformity, is characteristic of follicular lymphoma.  Image 2 (Higher magnification):  The cells appear small with round to oval nuclei and clumped chromatin, a hallmark feature of lymphocytes.  The nucleoli are inconspicuous, which is a feature of mature lymphocytes.  There is a lack of significant mitotic activity and no noticeable necrosis, which further supports the diagnosis of a low-grade lymphoma like follicular lymphoma.  In some areas, there is an appearance of germinal centers where the lymphoid follicles have a more organized appearance, which is typical of follicular lymphoma.  The absence of atypical cells with clear cytoplasm or large, pleomorphic nuclei helps rule out more aggressive lymphomas like Burkitt lymphoma or Hodgkin lymphoma.  Conclusion:  The lesion is most consistent with follicular lymphoma, which is a common type of non-Hodgkin's lymphoma, particularly in the oral cavity. This diagnosis is supported by the nodular architecture and the presence of small, uniform lymphocytes. | Based on the provided hematoxylin and eosin (H&E) images of the oral cavity lesion, here is the analysis:  Single Most Likely Histopathological Diagnosis:  The most likely diagnosis is Lymphoma, specifically a non-Hodgkin lymphoma (NHL), given the features observed in the provided images.  Key Microscopic Features Justifying the Diagnosis:  Cellular Uniformity: In both images, there is a marked predominance of small, round cells with relatively uniform size. The lack of significant variation in cell size or shape points to a lymphoma, particularly a form of NHL, where uniformity is characteristic.  High Cellularity: The lesion shows a densely packed arrangement of cells. This is a hallmark of lymphoproliferative diseases such as lymphoma, where the tumor is composed of numerous neoplastic lymphocytes.  Nuclear Features: The nuclei of the cells appear round or oval with finely dispersed chromatin, and some nuclei exhibit a prominent nucleolus. This appearance is consistent with lymphoma cells, which often show nuclear features such as these.  Absence of Well-Defined Tissue Architecture: There is a lack of normal tissue differentiation or organization, which further supports the diagnosis of lymphoma, where the lymphoid architecture is disrupted as neoplastic lymphocytes infiltrate the tissue.  Peripherally Located Nuclei: The cytoplasm is minimal, and the nuclei are mostly located peripherally, a feature commonly seen in lymphoma cells where the cytoplasm is scant.  These microscopic findings, particularly the cellular density and homogeneity, nuclear appearance, and loss of normal tissue architecture, align with the features of lymphoma, specifically non-Hodgkin lymphoma, as a probable diagnosis. |
| 18.10 | Diagnosis: Plasma cell neoplasm (histologic findings); Key Microscopic Features: Diffuse infiltration by tumor cells with destruction of tissue architecture. Sheets of plasma cells with eccentrically placed nuclei are present. | Based on the two different magnifications, I can identify key microscopic features which might indicate the presence of a particular type of lymphoma, potentially a Non-Hodgkin's lymphoma (NHL) or another hematolymphoid tumor. Here’s the breakdown of features commonly associated with NHL or similar lesions:  Low Magnification (First Image):  The lesion displays a diffuse cellular infiltrate with scattered areas of tissue necrosis.  The overall architecture appears effaced, which is typical of malignant lymphoid infiltrates.  There are scattered spaces that likely correspond to the invasion of tumor cells into surrounding tissue, which is common in NHLs.  High Magnification (Second Image):  The cellular population is predominantly small to medium-sized lymphocytes with hyperchromatic nuclei and scant cytoplasm.  There are areas with abnormal mitoses, suggesting active proliferation of lymphoid cells, which further supports malignancy.  The presence of reactive blood vessels is also visible, which is often seen in lymphomatous lesions.  There appears to be a lack of significant pleomorphism, which suggests a less aggressive variant, but with clear signs of infiltration and potential disruption of normal tissue.  Diagnosis:  Most likely diagnosis: Non-Hodgkin’s lymphoma (NHL), especially the B-cell type.  This diagnosis is based on the diffuse lymphoid infiltrate, presence of necrotic areas, and proliferative patterns seen at both low and high magnifications.  Key Microscopic Features Justifying the Diagnosis:  Effaced tissue architecture: The tissue structure is disrupted by infiltrating lymphoid cells.  Diffuse lymphoid infiltrate: A characteristic feature of lymphoma, where normal tissue is replaced by malignant lymphocytes.  Proliferation of lymphoid cells: The presence of abnormal mitoses and a high lymphocyte count points to a malignant process.  Necrosis and cytological features: Scattered necrosis and hyperchromatic, scant cytoplasm of lymphoid cells suggest lymphoma, especially of the aggressive type.  For a more definitive diagnosis, additional immunohistochemistry (IHC) or molecular studies would be required to identify the specific subtype of lymphoma. | The provided hematoxylin-and-eosin (H&E) images are of a lesion from the group of hematolymphoid tumors in the oral cavity. Upon examining these images at different magnifications, I can provide the following analysis:  Single Most Likely Histopathological Diagnosis:  The lesion in these images most likely represents Lymphoma, particularly Non-Hodgkin's Lymphoma (NHL), which is the most common type of hematolymphoid tumor in the oral cavity.  Key Microscopic Features Justifying the Diagnosis:  Image 1 (Low Magnification):  Diffuse Pattern: The lesion shows a diffuse pattern of growth with scattered areas of white space. This suggests that the tissue is infiltrated with a homogeneous population of cells without forming distinct nodules or structures.  Heterogeneous Background: There are scattered larger spaces and possible areas of necrosis, which are sometimes seen in lymphomatous infiltrates.  Image 2 (High Magnification):  Monomorphic Cell Population: The predominant cell type appears to be small, round, and monotonous with centrally located nuclei. These are characteristic of lymphoid cells, which is typical for lymphomas.  High Nuclear-to-Cytoplasmic Ratio: The nuclei of the cells are highly prominent with little cytoplasm, a feature commonly seen in lymphoma.  Nucleoli and Chromatin: The chromatin is fine, and the nucleoli are either inconspicuous or very subtle. This is typical for a small B-cell lymphoma or T-cell lymphoma.  Sparse Stroma: The stroma is relatively sparse, and the tissue looks highly cellular, another hallmark of lymphoma.  Lack of Reactive Changes: There are no significant signs of inflammation or granuloma formation, suggesting the lesion is a primary neoplastic process rather than a reactive condition.  Conclusion:  Based on the histopathological features seen in the provided images, Non-Hodgkin’s Lymphoma (NHL) is the most likely diagnosis for this lesion, given the monomorphic lymphoid cell population, the lack of reactive inflammatory changes, and the diffuse pattern of tissue infiltration. Further immunohistochemical staining and clinical correlation would be required for a more specific subtype classification (e.g., B-cell or T-cell lymphoma). |
| 18.11 | Diagnosis: Extramedullary involvement by acute myeloid leukemia; Key Microscopic Features: The tumor cells form a diffuse infiltrate with tissue destruction. Numerous apoptotic bodies and mitotic figures indicate a high proliferative rate. The tumor cells are large with ovoid nuclei, dispersed chromatin, distinct nucleoli, and pale cytoplasm. | Based on the hematoxylin and eosin-stained slides you provided, the following analysis is made:  Single Most Likely Histopathological Diagnosis:  The lesion appears to be consistent with Lymphoma, particularly Diffuse Large B-cell Lymphoma (DLBCL), which is a common type of hematolymphoid tumor in the oral cavity.  Key Microscopic Features:  Cellularity: The lesion shows a densely packed cell population with relatively little stroma, indicative of a high cellularity. This is typical of lymphoma, where neoplastic lymphoid cells often infiltrate the tissue in a diffuse pattern.  Monomorphic Population: The tumor cells are quite uniform in size and shape, with a round to oval nucleus, a high nuclear-cytoplasmic ratio, and sparse cytoplasm. This suggests a monoclonal population of neoplastic cells, which is characteristic of lymphoma.  Nuclear Features: The nuclei are moderately pleomorphic, but they predominantly show features of large cells, with prominent nucleoli and coarse chromatin. This finding is often seen in high-grade lymphomas, including DLBCL.  Mitotic Figures: There are occasional mitotic figures scattered within the lesion, suggesting high cellular turnover, a feature often present in aggressive lymphomas like DLBCL.  Absence of other cell types: The absence of significant numbers of small lymphocytes, plasma cells, or other inflammatory cells further supports the diagnosis of a lymphoma, rather than a reactive process.  These features collectively support the diagnosis of a lymphoid malignancy, most likely Diffuse Large B-cell Lymphoma (DLBCL), which is common in the oral and maxillofacial region. | The images provided appear to show histopathological sections of a lesion from the "Hematolymphoid Tumors of the Oral Cavity" category, stained with Hematoxylin and Eosin (H&E), captured at different magnifications.  Based on the features observed in the images, the diagnosis seems most consistent with Lymphoma, specifically Extranodal Marginal Zone B-Cell Lymphoma (MALT lymphoma). This is one of the common types of lymphoma that can occur in the oral cavity.  Key Microscopic Features Supporting this Diagnosis:  Monotonous Cellular Population: The lesion consists of a relatively homogeneous population of small lymphoid cells with round to slightly irregular nuclei. There is little variation in cell size, and the nuclei appear relatively bland without significant pleomorphism.  Perinodular Distribution: In the higher magnification image, a characteristic feature is the distribution of the lymphoid cells around blood vessels and a diffuse architecture that does not show the typical nodular arrangement of reactive lymphoid tissue. This suggests lymphoma rather than benign reactive hyperplasia.  Presence of Small Lymphocytes: Small lymphocytes are the predominant cell type seen throughout the images, and there is a lack of large, transformed cells, which is typical in certain types of lymphoma like MALT lymphoma.  Clear Cytoplasm with Chromatin Pattern: The cytoplasm of the lymphoid cells is sparse, and the chromatin appears coarse and clumped, a characteristic feature of small B-cells, which are commonly seen in extranodal lymphoma.  These features are key indicators of a low-grade B-cell lymphoma, specifically MALT lymphoma, which can occur in extranodal sites, including the oral cavity. The absence of significant necrosis, prominent mitotic figures, and anaplastic cells further suggests a low-grade or indolent lymphoma. |
| 18.12 | Diagnosis: Extranodal NK/T-cell lymphoma, nasal type; Key Microscopic Features: The infiltrate exhibits a diffuse growth pattern with significant angiodestruction. Tumor cells are variable in size, ranging from small to large, with irregular elongated nuclei and indistinct nucleoli. | Based on the two images provided, the lesion in question shows typical histopathological features suggestive of a lymphoma, more specifically, non-Hodgkin lymphoma (NHL). These are typical findings in the context of hematolymphoid tumors of the oral cavity.  Key Microscopic Features Justifying the Diagnosis:  High Cellularity: Both images show an increased cellular density, with closely packed cells, indicative of neoplastic lymphoid cells. In the second image, the cells appear predominantly monomorphic, suggesting a clonal proliferation of lymphoid cells.  Medium to Small Lymphoid Cells: The cells in the lesion are medium-sized and demonstrate uniform nuclear morphology. This uniformity is characteristic of lymphoma, with a predominance of small lymphocytes.  Prominent Nucleoli: The lymphoid cells show prominent nucleoli in the second image, which is a feature that supports malignancy in lymphoma, often reflecting an active cellular turnover.  Scattered Immunoblasts or Large Cells: In the first image, there may be the presence of slightly larger cells scattered throughout the lesion, which could be indicative of immunoblasts, a feature often seen in high-grade lymphomas.  Absence of a Clear Architecture: The typical architecture seen in normal lymphoid tissue (such as germinal centers or well-defined follicular patterns) is disrupted, which is characteristic of lymphoma. The images show an overall disorganized pattern.  Most Likely Diagnosis:  Non-Hodgkin Lymphoma (NHL), likely diffuse large B-cell lymphoma (DLBCL) or another high-grade subtype, given the features of high cellularity and the uniformity of the lymphoid cells.  This diagnosis would need to be confirmed by immunohistochemical studies and possibly molecular studies to distinguish between various subtypes of lymphoma. | Based on the provided hematoxylin-and-eosin (H&E) images of the lesion, here is my analysis:  Histopathological Diagnosis:  The most likely diagnosis based on the appearance of the histopathological slides is Lymphoma, particularly a B-cell lymphoma, possibly extranodal marginal zone lymphoma (MALT lymphoma), given the architectural pattern and cellular characteristics.  Key Microscopic Features:  Cellular Composition:  The lesion shows a predominantly monomorphic population of small lymphocytes.  There are scattered larger cells, which could represent centroblasts or immunoblasts, indicative of a lymphoproliferative disorder like lymphoma.  Nuclear atypia is evident, with nuclei that appear irregular, and some nuclei show prominent nucleoli, consistent with lymphoma.  Background and Tissue Architecture:  The tissue architecture is disrupted, showing a diffuse infiltration of small round cells, which often occurs in lymphoid malignancies.  Absent normal tissue structures: The normal organization of the tissue has been replaced by the infiltrating lymphoid cells.  Histological Pattern:  Patternless or vaguely nodular pattern: In the higher magnification image, the lesion shows areas with a more diffuse infiltration of small lymphocytes, which is characteristic of lymphoma.  Abundant mitotic figures (not shown directly but often inferred from active lymphoid proliferation) may be present, suggesting increased cell turnover.  Lack of granulomas or necrosis: The absence of granulomatous inflammation or necrosis further supports lymphoma rather than an inflammatory or infectious process.  Cytological features:  The small, round lymphocytes show hyperchromatic nuclei with little cytoplasm, typical for small B-cells or T-cells in lymphoproliferative conditions.  There is minimal cytoplasm, and the cells are predominantly uniform in size.  These microscopic features together strongly suggest the diagnosis of Lymphoma, possibly MALT lymphoma or another type of low-grade B-cell lymphoma. However, a definitive diagnosis would require further immunohistochemical staining and clinical correlation. |
